# Enantioselective Aziridination of Unactivated Terminal Alkenes Using a Planar Chiral Rh(III) Indenyl Catalyst

**DOI:** 10.1021/jacs.3c10637

**Published:** 2024-01-03

**Authors:** Patrick Gross, Hoyoung Im, David Laws, Bohyun Park, Mu-Hyun Baik, Simon B. Blakey

**Affiliations:** † Department of Chemistry, Emory University, Atlanta, Georgia 30322, United States; ‡ Department of Chemistry, Korea Advanced Institute of Science and Technology (KAIST), Daejeon 34141, Republic of Korea; § Center for Catalytic Hydrocarbon Functionalizations, Institute for Basic Science (IBS), Daejeon 34141, Republic of Korea

## Abstract

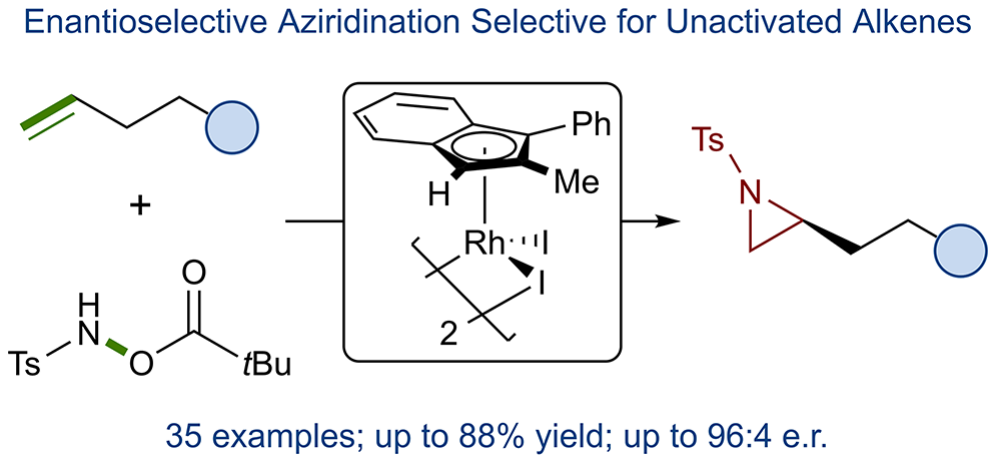

Chiral aziridines are important structural motifs found in natural products and various target molecules. They serve as versatile building blocks for the synthesis of chiral amines. While advances in catalyst design have enabled robust methods for enantioselective aziridination of activated olefins, simple and abundant alkyl-substituted olefins pose a significant challenge. In this work, we introduce a novel approach utilizing a planar chiral rhodium indenyl catalyst to facilitate the enantioselective aziridination of unactivated alkenes. This transformation exhibits a remarkable degree of functional group tolerance and displays excellent chemoselectivity favoring unactivated alkenes over their activated counterparts, delivering a wide range of enantioenriched high-value chiral aziridines. Computational studies unveil a stepwise aziridination mechanism in which alkene migratory insertion plays a central role. This process results in the formation of a strained four-membered metallacycle and serves as both the enantio- and rate-determining steps in the overall reaction.

## Introduction

Aziridines are valuable strained three-membered nitrogen-containing heterocycles known for their utility as nitrogen building blocks.^1^ Chiral aziridines command an elevated level of interest as they serve as excellent chiral building blocks and intermediates for the stereoselective incorporation of nitrogen while allowing for downstream diversification through regio- and stereoselective opening of the strained ring.^2−4^ Nucleophilic ring opening with or without Lewis acid activation is the most frequently employed method for aziridine diversification and can afford access to chiral amines, amino alcohols, amino acids, and other chiral nitrogen motifs.^5,6^ The ease with which chiral aziridines can be diversified has made them desirable targets and has driven the development of synthetic methods to access them in an expedient fashion.^7−9^

**Scheme 1:**
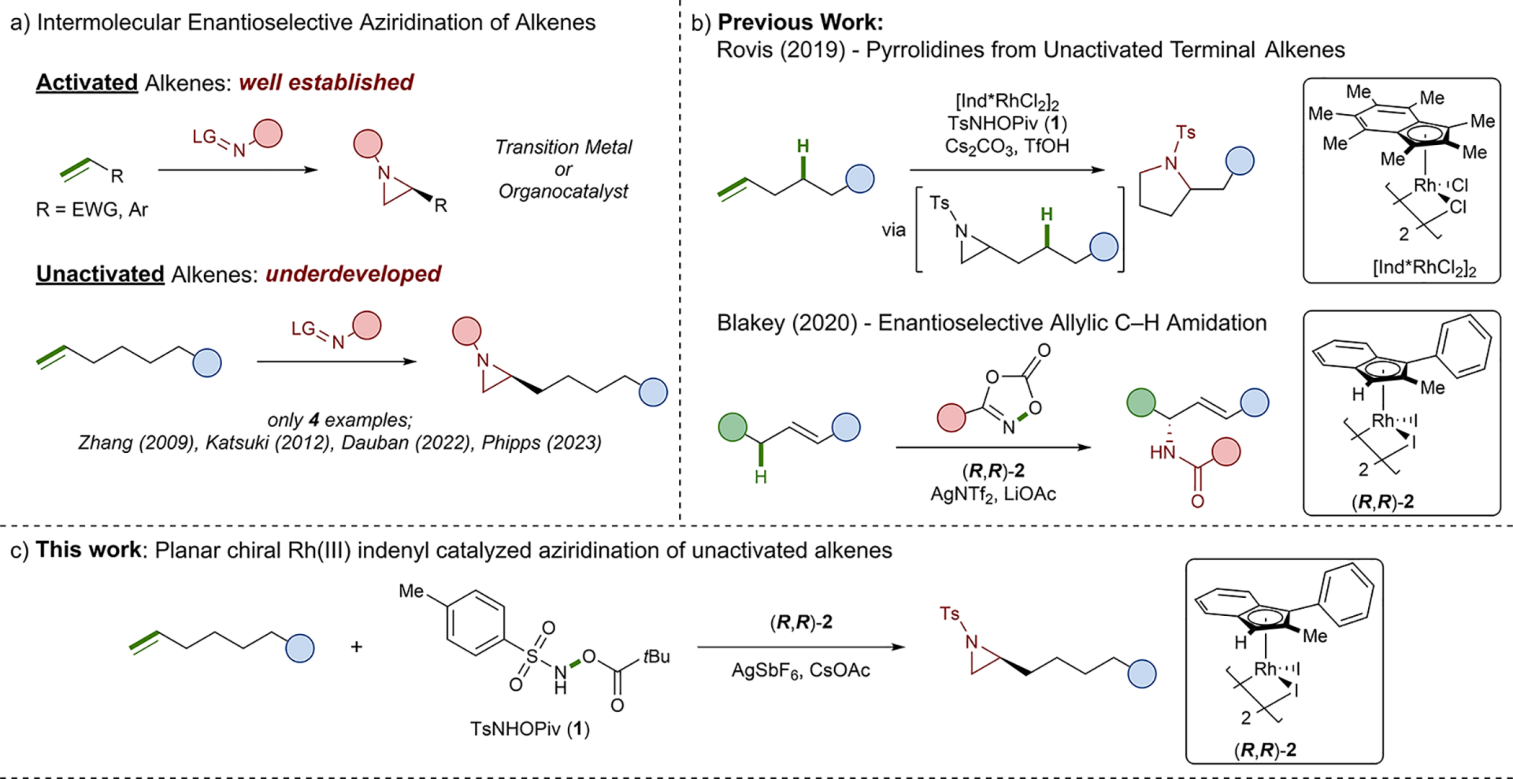
Functionalization of Activated and Unactivated Alkenes

Stereoselective synthesis of aziridines can be achieved through three distinct synthetic disconnections: (1) intramolecular condensation of chiral haloamines or amino alcohols, (2) stereoselective addition of carbon sources to imines, or (3) stereoselective addition of nitrene equivalents to alkenes. Of these, the addition of a nitrene equivalent across an alkene is the most attractive, due to the simplicity of the disconnection and the availability of alkene building blocks. While catalytic methods have been developed using organocatalysts,^10^ transition metal-catalyzed methods have remained the predominant means of accessing chiral aziridines through this disconnection. Following the seminal work by Evans^11,12^ and Jacobsen,^13^ the development of Cu(II)-catalyzed methodologies using discreet or in situ-generated hypervalent imino-iodinane nitrene sources has received the most attention.^14−18^ Since then, other methodologies have been developed using Ru(II),^19−21^ Co(II),^22,23^ Rh(II),^24−26^ Fe(II),^27^ Mn(III),^28^ and Ag(I),^29^ along with a variety of unique ligand designs and nitrene sources, enabling both intra- and intermolecular stereoselective aziridinations. However, these methods have focused on the aziridination of activated alkenes such as styrenes and α,β-unsaturated systems (Scheme 1a, top). The stereoselective aziridination of unactivated alkenes with alkyl substitutions remains challenging (Scheme 1a, bottom).^30^

As a result, only four methods that are capable of conducting intermolecular enantioselective aziridinations on unactivated alkenes have been reported. A Co(II) porphyrin catalyst developed by Zhang was shown to aziridinate a variety of styrenyl substrates along with a small number of unactivated substrates.^31^ A report by Katsuki using a Ru(II)[salen] catalyst also showed the aziridination of a small number of unactivated alkenes alongside activated systems.^32^ In 2022, Dauban reported on the asymmetric aziridination of tetra-substituted styrenes using a chiral dirhodium(II) tetracarboxylate catalyst and showed that unactivated alkenes could also function in this system.^33^ In a recent report, Phipps showed the use of an achiral dirhodium(II) catalyst in conjunction with a chiral cationic organocatalyst and a pendant hydroxyl directing group for the aziridination of both activated and unactivated alkenes with a variety of substitution patterns.^34^ These methods represent significant advances in enantioselective aziridination technology, but there still exists room for further development. While each of these methods can conduct aziridinations on unactivated systems, their focus still lies with activated substrates, with unactivated systems often providing lower yields and enantioselectivities. Furthermore, each of these systems relies on either azide or in situ-generated imino-iodinane nitrene sources, which can present safety and chemoselectivity liabilities.

We recognized the possibility of developing a broadly applicable asymmetric unactivated alkene aziridination method building from a report by Rovis and coauthors for the synthesis of pyrrolidines from unactivated terminal alkenes (Scheme 1b, top).^35^ Therein, the use of [Ind*RhCl_2_]_2_ in combination with a hydroxylamine nitrogen transfer reagent **1** and TfOH allowed for formal [4 + 1] cycloadditions. During their investigation, they determined that the transformation proceeded via an isolatable aziridine intermediate, demonstrating a previously unprecedented mode of reactivity for a Rh(III) catalyst. Additionally, they noted that the indenyl ligand was critical for reactivity, with the analogous [Cp*RhCl_2_]_2_ catalyst resulting in significantly reduced yields. Inspired by this precedent, we sought to render the aziridination enantioselective through the use of our planar chiral Rh(III) indenyl catalyst **2**, developed in the context of an enantioselective allylic C–H amidation reaction (Scheme 1b, bottom).^36,37^ Herein, we report the development of an enantioselective aziridination method targeting unactivated alkenes using a simple hydroxylamine in combination with a planar chiral Rh(III) indenyl catalyst (Scheme 1c). Furthermore, we detail our computational investigations into the mechanism of this reaction.

## Results and Discussion

### Reaction Optimization

**Figure 1:**
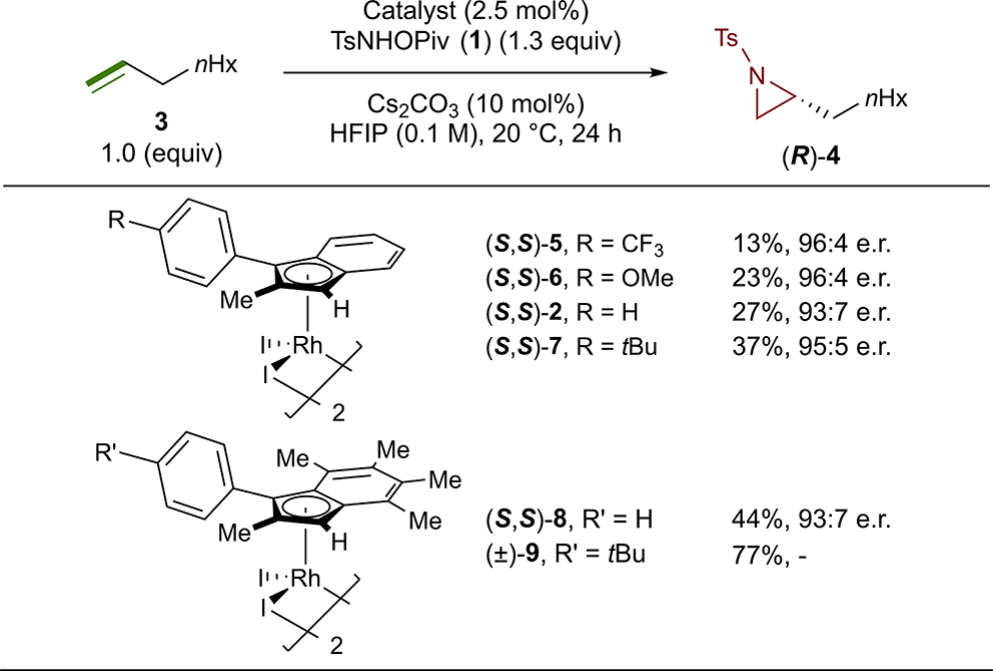
Screening of electronically varied planar chiral Rh(III) indenyl catalysts. Reactions were performed on a 0.10 mmol scale. Isolated yields are reported, and enantiomeric ratios were determined by chiral HPLC on an AD-H column (3% isopropanol in hexanes).

We began our investigation by exploring the effect of indenyl ligand electronics on catalyst reactivity in the aziridination of 1-nonene (**3**) to form aziridine (***R***)-**4**.^38^ We selected our previously developed planar chiral catalyst **2** as well as four electronically tuned variants **5**–**8** (Figure 1). Catalysts **5**–**8** are accessible in two steps via racemic Rh(I)(COD) intermediates, which can be separated via chiral preparative HPLC to afford enantiopure catalysts (see the Supporting Information for details). Subjecting **3** to the conditions previously reported by Rovis in the presence of a CF_3_-substituted catalyst (***S***,***S***)-**5** (2.5 mol %) provided the aziridine (***R***)-**4** in a 13% yield with excellent enantioselectivity (96:4 e.r.) (Figure 1). Using methoxy-substituted catalyst (***S***,***S***)-**6** improved the yield of (***R***)-**4** to 23% while maintaining excellent enantioselectivity (96:4 e.r.). We believe the out-of-plane orientation of the phenyl moiety prevents the methoxy substituent from donating electron density to the indene via resonance, and it functions as an electron-withdrawing group through inductive effects. Catalyst (***S***,***S***)-**2** further improved the yield of (***R***)-**4** to 27% while still achieving high enantioselectivity (93:7 e.r.). Using the more electron-rich catalyst (***S***,***S***)-**7** improved the yield of (***R***)-**4** to 37% (95:5 e.r.). A further increase in yield was achieved when the pentamethylated catalyst (***S***,***S***)-**8** was used, providing (***R***)-**4** in a 44% yield with the same excellent enantioselectivity (95:5 e.r.). To capitalize on this reactivity trend, we designed a more electron-rich catalyst (±)-**9**, which significantly improved the yield of **4** to 77% (Figure 1). Unfortunately, (±)-**9** could only be accessed in its racemic form as design elements, which made this catalyst so effective, prevented the separation of the planar chiral Rh(I) intermediate whether through chiral preparative HPLC or other planar chiral catalyst resolution strategies.^39^ We note that under these initial conditions, no halide abstracting additive, commonly required when using this family of catalysts, was included. We therefore hypothesize that this trend in catalyst reactivity could stem from the ability of the electron-donating catalysts **7**–**9** to better facilitate the dissociation of the iodide ligands by stabilizing the resulting cationic Rh(III) species.

**Figure 2:**
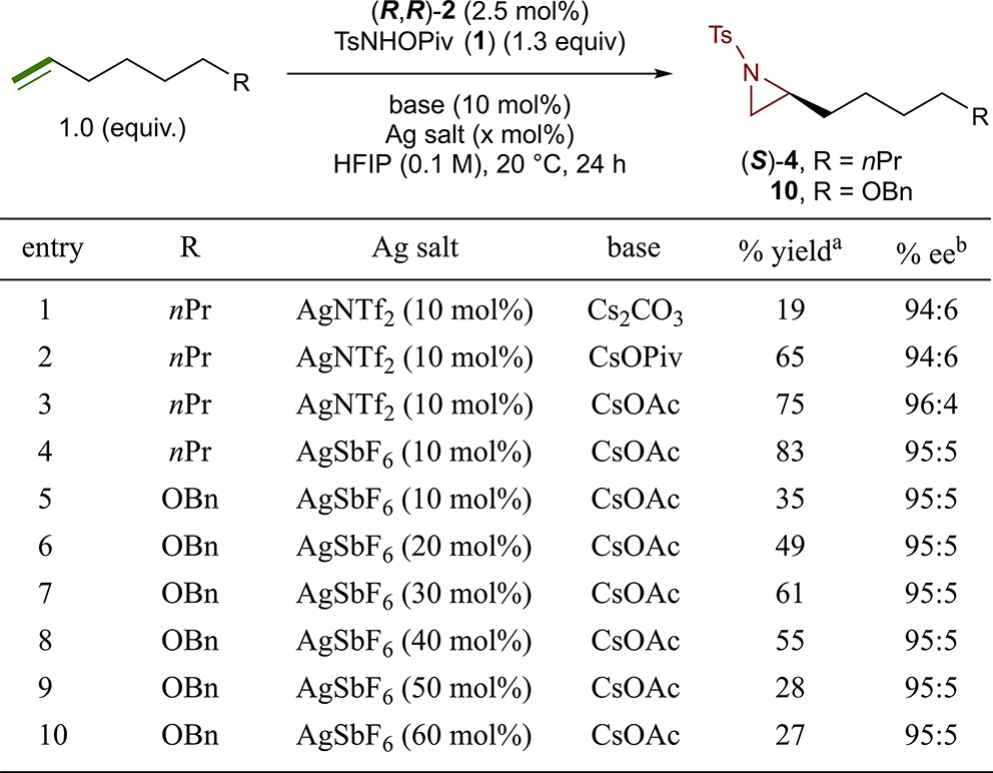
Optimization of the Ag salt variable. Reactions were run on a 0.10 mmol scale. ^*a*^ Isolated yields. ^*b*^ Enantiomeric ratios were determined by chiral HPLC (see the Supporting Information for details).

To explore this possibility, we investigated the use of Ag halide scavengers to activate the catalyst. Although our first attempt using catalyst (***R***,***R***)-**2** in combination with AgNTf_2_ (10 mol %) in the aziridination of 1-nonene did not prove successful and lead to a reduction in yield (19%, 94:6 e.r.) (entry 1, Figure 2), a change in base from Cs_2_CO_3_, to CsOPiv proved to be key, delivering an improved yield of 65% while maintaining excellent enantioselectivity (95:5 e.r.) (entry 2, Figure 2). Final improvements to the reaction were made by using AgSbF_6_ (10 mol %) and CsOAc (10 mol %), allowing for the formation of (***S***)-**4** in an 83% yield (95:5 e.r.) (entry 4, Figure 2). We noted that for some substrates, such as benzyl-protected 5-hexen-1-ol (entry 5, Figure 2), these initial conditions were less effective. Based on our previous observations that loading of the silver additive could impact the reaction yield, we systematically investigated this variable. For this more challenging substrate, we discovered that a loading of 30 mol % of AgSbF_6_ provided aziridine **10** in a 61% yield with the same excellent enantioselectivity (95:5 e.r.) (entry 6, Figure 2). Loadings of AgSbF_6_ above or below 30 mol % were found to be detrimental to the yield of **10** (entry 6, 8–9, Figure 2). At this time, we do not have an explanation for this observation, and we speculate that the extra loading of Ag may be assisting in recovering the catalyst from off pathway decomposition.

### Scope and Limitations

**Figure 3:**
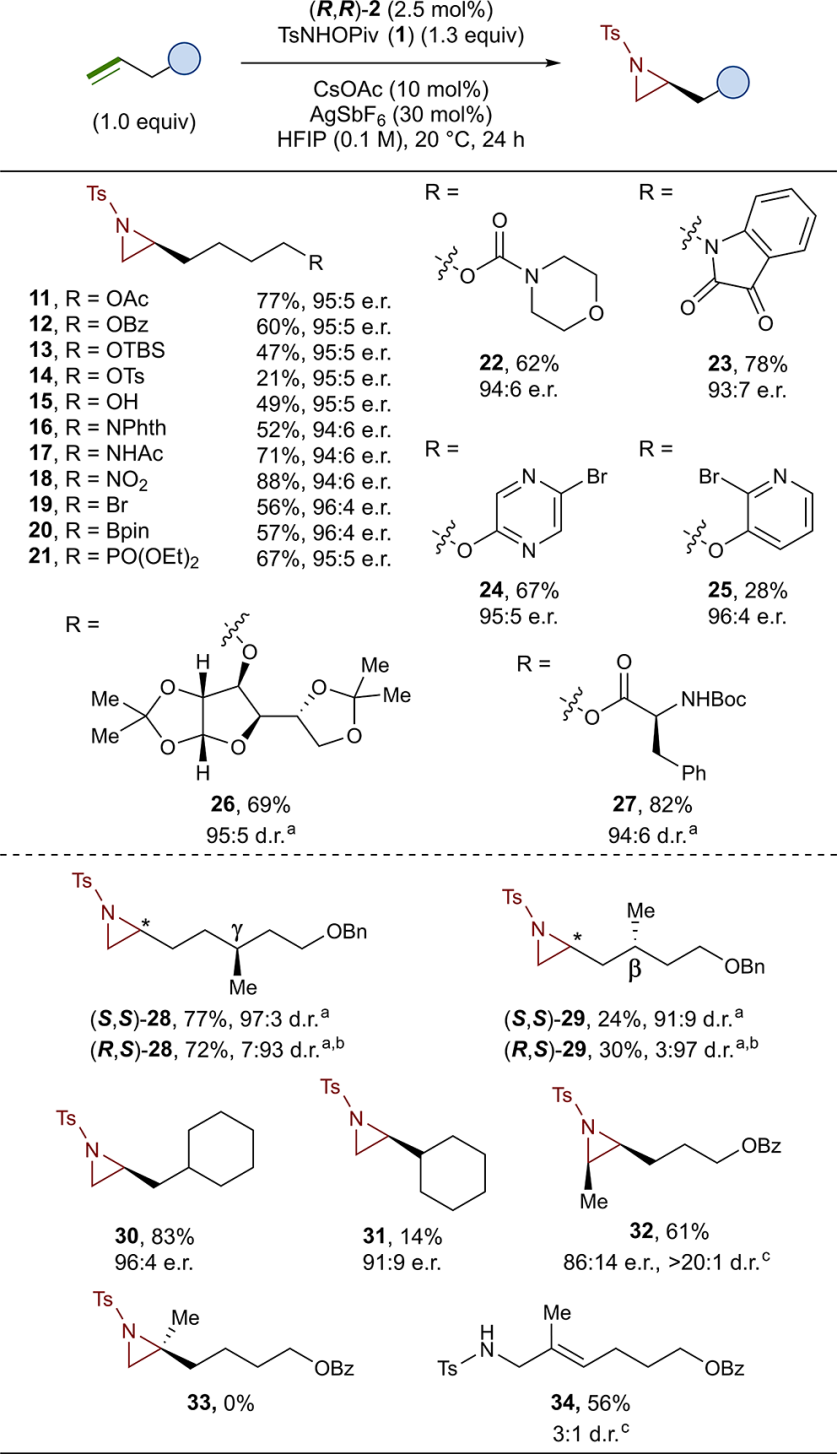
Scope of functionalized alkene substrates. Reactions were run on a 0.10 mmol scale. Isolated yields are reported, and enantiomeric ratios were determined by chiral HPLC (see the Supporting Information for details). ^a^Diastereomeric ratio was determined by chiral HPLC. ^b^(***S***,***S***)-**2** was used as the precatalyst. ^c^Diastereomeric ratio was determined by integration of the crude ^1^H NMR spectra.

With the optimized conditions in hand, we investigated a variety of alkene substrates with functionalized alkyl chains. We first examined a series of 5-hexen-1-ol derivates, which were well tolerated and generally provided the aziridines **11**–**14** in good yields (21–77%) with excellent enantioselectivities (95:5 e.r.) (Figure 3). The unprotected 5-hexen-1-ol was also tolerated and gave aziridine **15** in a 49% yield and 95:5 e.r. Phthalimide-protected alkyl amine was found to be robust, with aziridine **16** being formed in a 52% yield and 94:6 e.r. The free N–H of an N-alkyl acetamide did not inhibit the reaction and provided aziridine **17** in a 71% yield with excellent enantioselectivity (94:6 e.r.). A nitro substituent, an alkyl halide, a boronic ester, and a phosphonate ester were all well tolerated, giving aziridines **18**–**21** in good yields (56–88%) with excellent enantioselectivities (94:6–96:4 e.r.). With good tolerance for a variety of heteroatoms established, we next explored more complicated substrates with various heterocycles appended. Aziridines **22**–**25** with an appended morpholine carbamate, isatin, diazine, and pyridine were all synthesized in good yields (28–78%), and high enantioselectivities were maintained in all cases (93:7–95:5 e.r.). Aziridination of protected d-glucofuranose and *L*-phenyl alanine derivates were also successful, providing aziridines **26** and **27** in a 69% yield, 95% d.r., and an 82% yield, 94:6 d.r., respectively.

Having established a broad functional group scope, we next explored substrate steric effects, beginning with matched and mismatched substrate catalyst pairings in the aziridination of *L*-citronellol-derived alkenes. A stereogenic center at the γ-position relative to the alkene does not significantly impact the reaction, allowing both diastereomers to be accessed in high yields with excellent catalyst control for both the matched diastereomer (***S***,***S***)-**28** (77%, 97:3 d.r.) and the mismatched diastereomer (***R,S***)-**28** (72%, 7:93 d.r.). Moving the stereogenic center to the β-position reduces the yield of the corresponding aziridines but does not impact the catalyst-controlled diastereoselectivity in either the matched (***R,S***)-**29** (30%, 3:97 d.r.) or mismatched (***S***,***S***)-**29** (24%, 91:9 d.r.) catalyst substrate pairings. Aziridination of allyl cyclohexane provided aziridine **30** in a good yield and with excellent enantioselectivity, demonstrating that substitutions at the beta position can be tolerated through rigidification. Substitutions at the α-position remain challenging, with the aziridination of vinyl cyclohexane providing aziridine **31** in a reduced yield of 14% (91:9 e.r.). The aziridination of disubstituted alkenes was also investigated with *cis*-aziridine **32** forming in a 61% yield, with a > 20:1 d.r. and 86:14 e.r. from the corresponding *Z*-alkene, while the *E*-substituted alkene was found to be unreactive. Aziridination of a 1,1-disubstituted alkene did not provide the desired aziridine **33** and instead afforded the terminal allylic amine **34** in a 56% yield (3:1 d.r).

**Figure 4:**
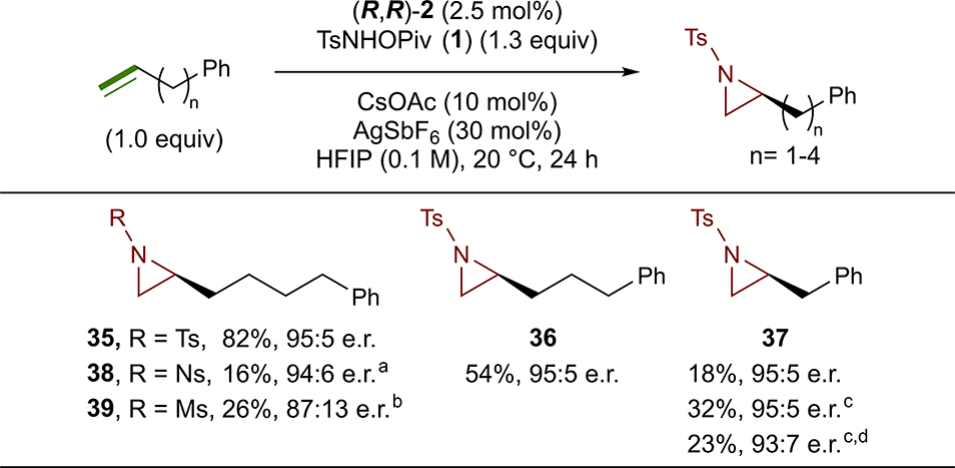
Scope of varied alkyl chain length substrates. Reactions were run on a 0.10 mmol scale. Isolated yields are reported, and enantiomeric ratios were determined by chiral HPLC (see the Supporting Information for details). ^a^NsNHOPiv was used as the nitrogen source. ^b^MsNHOPiv was used as the nitrogen source. ^c^(***R,R***)-**8** was used as the precatalyst. ^d^Reaction was run at 60 °C.

We continued our investigation into substrate steric effects by exploring the effect of the alkyl chain length. As demonstrated by many of the substrates shown above, a carbon chain length of four does not negatively affect the aziridination, and aziridine **35** was formed in an excellent yield (83%, 95:5 e.r.) (Figure 4). Shortening of the chain length to three carbons still allowed for the formation of aziridine **36** in a 54% yield and 95:5 e.r. Further shortening of the alkyl chain to one methylene unit led to the formation of aziridine **37** in a significantly reduced yield of 18% while still maintaining excellent enantioselective control (95:5 e.r.). The yield of **37** could be improved to 32% using pentamethylated catalyst (***R,R***)-**8** at room temperature. Attempts to further increase the yield by running the reaction at elevated temperature were not successful (60 °C, 23%, 93:7 e.r.). Comparison of the chiral HPLC trace of **37** to standards of (***R***)-**37** and (***S***)-**37** prepared through intramolecular condensation of commercially available chiral amino alcohols allowed for the stereochemical assignment of (*S*) to **37** and other substrates by analogy. We note at this time that both Ns (**38**)- and Ms (**39**)-substituted versions of **35** can be synthesized, however, in significantly reduced yields of 16% and 26%, respectively, and in the case of **39**, a reduction in enantioselectivity (87:13 e.r.) is observed. We also note that the chiral aziridine products may be deprotected using a variety of methods reported in the literature.^40−43^

**Figure 5:**
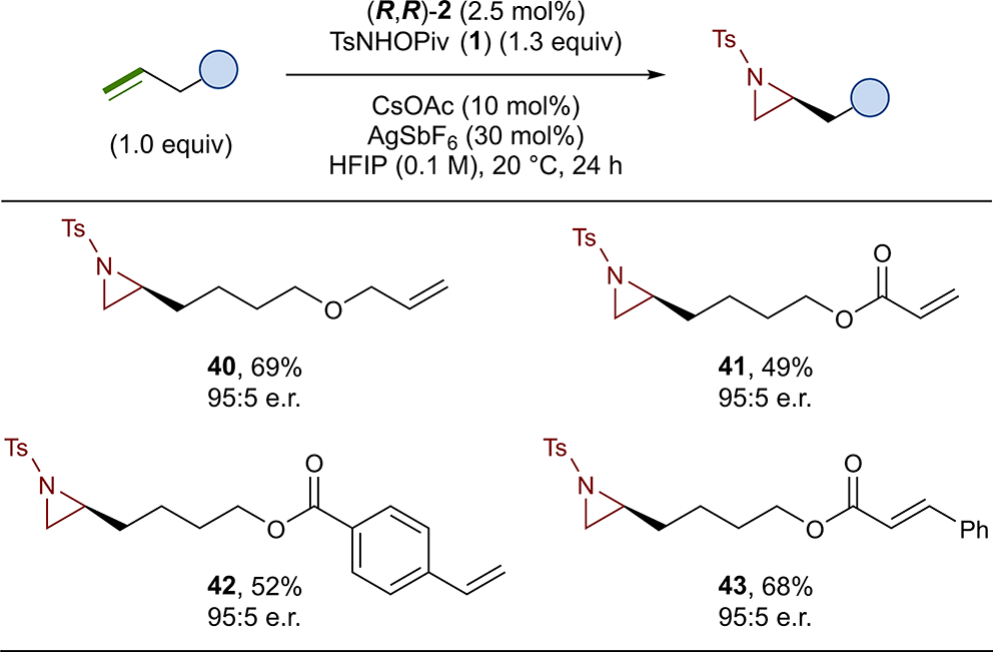
Scope of competition substrates containing activated and unactivated alkenes. Reactions were run on a 0.10 mmol scale. Isolated yields are reported, and enantiomeric ratios were determined by chiral HPLC (see the Supporting Information for details).

Next, we investigated the effect of alkene electronics by conducting competition reactions with substrates containing both activated and unactivated alkenes. Astonishingly, the terminal unactivated alkene was selectively aziridinated in the presence of an ally ether (**40**, 69%, 95:5 e.r.), an acrylic ester (**41**, 49%, 95:5 e.r.), a styrene (**42**, 52%, 95:5 e.r.), and a cinnamate (**43**, 68%, 95:5 e.r.) (Figure 5). In all four competition reactions, aziridination occurred exclusively at the unactivated alkene, with the activated alkenes remaining intact, demonstrating a previously unknown level of selectivity in an enantioselective aziridination method.

### Computational Mechanism Study

**Scheme 2:**
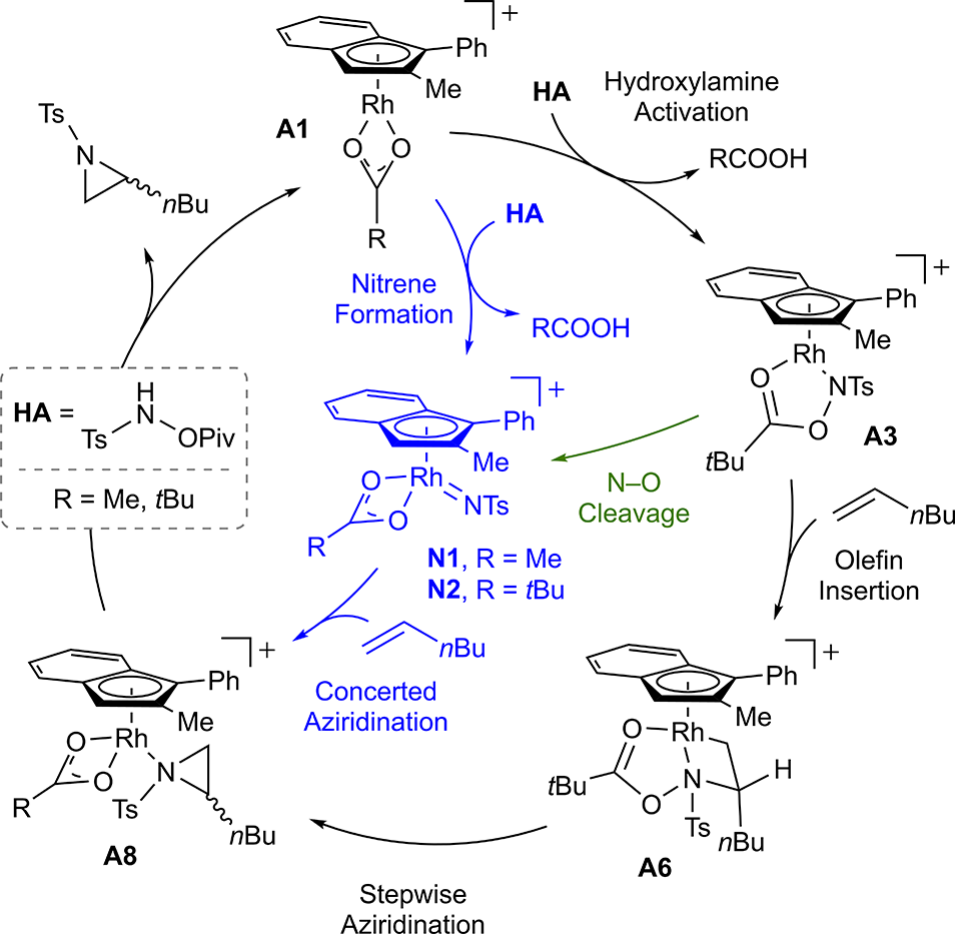
Plausible Reaction Pathways for Aziridine Formation

To gain a comprehensive understanding of the mechanism, density functional theory (DFT) calculations at the B3LYP-D3/cc-pVTZ(-f)/LACV3P//B3LYP-D3/6-31G**/LACVP level of theory^44−49^ were conducted.^50^ Our investigation began with the 16-electron complex **A1** using 1-hexene and CsOAc as the reactant and base, respectively. Two plausible mechanistic scenarios were considered, as depicted in Scheme 2: the first scenario, depicted by the black trace, initiates with the formation of an amide, followed by subsequent olefin insertion. The second scenario, illustrated by the blue and green traces, follows a pathway through nitrene formation involving the concerted creation of both C–N bonds. We ruled out the possibility of a direct concerted metalation–deprotonation (CMD) of the olefin due to its unrealistically high energy demand (see the Supporting Information for details).

**Figure 6:**
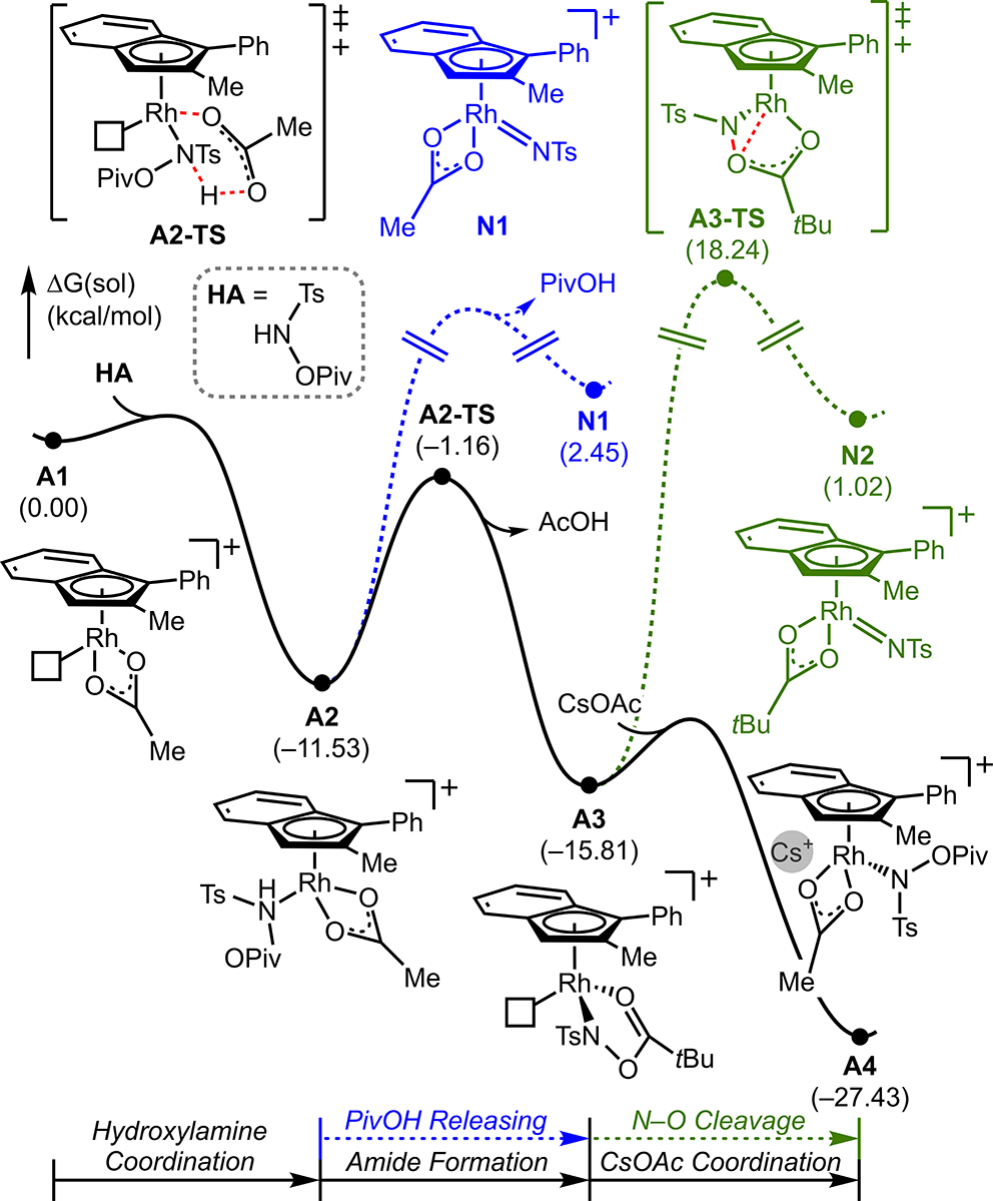
Free energy profile for the formation of **A4** from **A1**. Blue and green traces represent direct nitrene formation via N–O bond cleavage.

As shown in Figure 6, the coordination of hydroxylamine **HA** to **A1** exergonically gives an 18-electron complex **A2**. Subsequently, intramolecular deprotonation of **A2** takes place, resulting in the liberation of acetic acid and the formation of a metal-amide complex, **A3**, characterized by a reaction barrier of 10.4 kcal/mol. Then, the 5-coordinated complex **A3** engages an additional base, forming a cesium-bound complex, **A4**. Notably, **A4** serves as a resting intermediate, associated with an energy change of −11.6 kcal/mol.^51^

**Figure 7:**
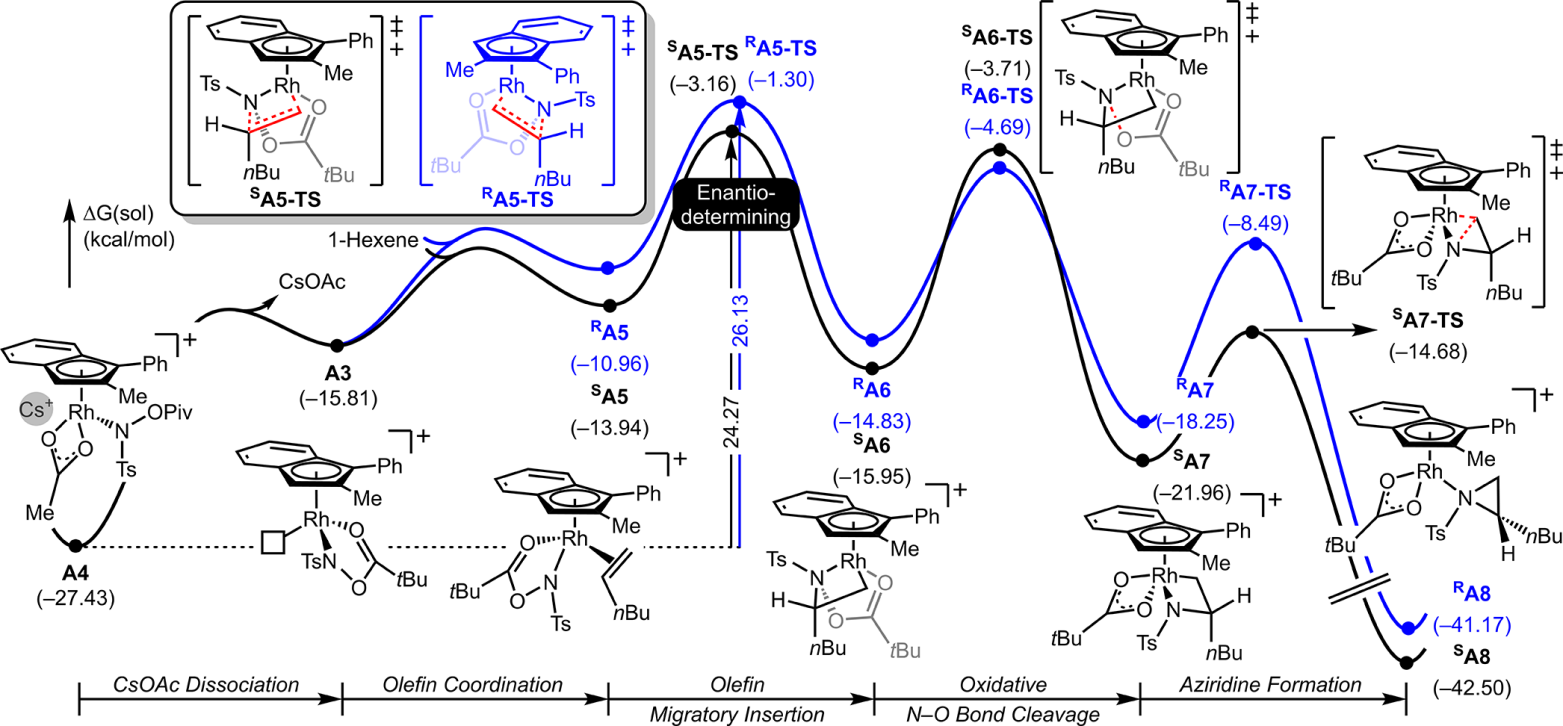
Energy profile of the olefin insertion step and aziridination step. Structures for the (*R*) pathways are omitted in this figure.

During the formation of **A4**, two intermediates **A2** and **A3** have the potential to form nitrene intermediates, **N1** and **N2**, as illustrated by the blue and green traces in Figure 6. While the formation of **N1** involves an intramolecular acid-base reaction, both pathways necessitate oxidative N–O bond cleavage to yield pivalate and nitrene. However, the resulting metal-nitrene intermediates, **N1** and **N2**, exhibit free energies of 2.4 and 1.0 kcal/mol, respectively. These energy levels are even higher than the likely rate-determining transition state in the alternative mechanism (**^R^A5-TS** in Figure 7). These consistently high energies suggest that the reaction does not proceed via the nitrene intermediate. Additional details are provided in the Supporting Information.

Turning our attention back to the resting intermediate, **A4**, we delved deeper into the subsequent stages of catalysis, specifically the phase where olefin insertion leads to aziridine formation. Because **A4** is saturated, it releases CsOAc and reverts to 16-electron complex **A3**, which can then react with an olefin. Due to the asymmetric structure of **A3**, it presents two distinct vacant sites for the olefin, and this leads to an enantioselective C–N bond formation (**^S^A5** and **^R^A5**). This observation aligns with the experimental results, indicating a 19–24 times higher yield of the (*S*)-product compared to that of the (*R*)-product (Figure 2, entries 3 and 4). Notably, our calculations estimate the energy difference between the two transition states, ^**S**^**A5**-**TS** and **^R^A5-TS**, to be 1.9 kcal/mol. Moreover, this migratory insertion (first C–N bond formation) was found to have the highest activation energy through the catalytic cycle. It likely serves as both the enantio- and rate-determining transition step.

**Figure 8:**
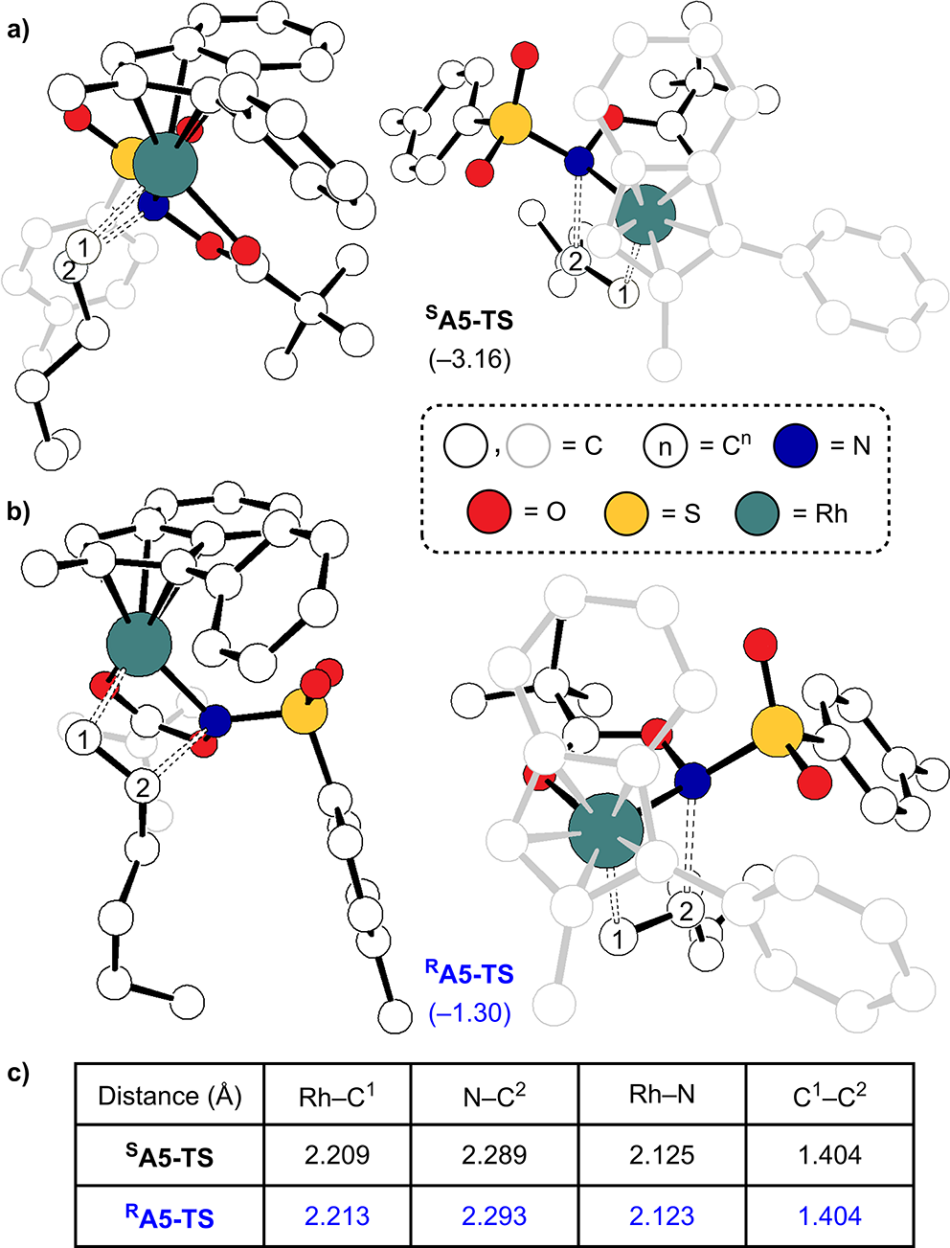
Structures of (a) **^S^A5-TS**, (b) **^R^A5-TS**, and (c) distances between the atoms involved in the olefin insertion step. All hydrogen atoms in the structures have been omitted for clarity.

The stereoselectivity of the reaction arises from interactions among the substrate, the sulfonamide, and the phenyl substituent on the indenyl ligand of the catalyst. Our computational studies demonstrate that during C–N bond formation from **A5** to **A7**, the tosyl group and the alkyl chain on the olefin are both positioned within the same quadrant. In the context of (*S*)-product formation, the tosyl and alkyl groups adopt an orientation opposite that of the phenyl substituent, spanning from **^S^A5** to **^S^A7**. Conversely, for the formation of the (*R*)-product, two substituents are located in the same vicinity as the phenyl substituent. Figure 8a and b presents the optimized structures of **^S^A5-TS** and **^R^A5-TS** as representative examples. Clearly, this distinction leads to a variation in the steric interaction. Both distortion-interaction analysis^52,53^ and the energy decomposition analysis^54−59^ indicate that the primary contribution to the energy difference between **^S^A5-TS** and **^R^A5-TS** arises from steric interactions, accounting for the difference of 1.9 kcal/mol (Figure 8a). Further details can be found in the Supporting Information.

After the migratory insertion, intermediate **A6** undergoes subsequent oxidative N–O bond cleavage, forming Rh(V) intermediate **A7**. Leveraging the electron-deficient environment in **A7** as a driving force, reductive C–N bond formation readily takes place with step barriers of 7.3 and 9.8 kcal/mol for **^S^A7-TS** and **^R^A7-TS**, respectively. Finally, the dissociation of the aziridine product from **A8** regenerates the pivalate analogue of active catalyst **A1**, which can initiate a new catalytic cycle.

## Conclusions

Herein, we report an enantioselective aziridination reaction using a planar chiral rhodium indenyl catalyst and unravel the origin of the enantioselectivity by DFT calculations. The reaction provides a wide range of enantioenriched aziridines and shows remarkable selectivity for unactivated olefins in the presence of activated olefin motifs. The mild reaction conditions and easy preparation of the catalyst represent additional features and contribute to the versatility of the reaction. Computational studies revealed that the activation of hydroxylamine occurs prior to olefin engagement. Both nitrene and π-allyl complex formation are less favorable than the formation of the amide intermediate. The amide intermediate undergoes an olefin insertion step, which is both an enantio- and rate-determining step. The enantioselectivity arises from the difference in the steric clash between the olefin and amide intermediate, which favors the formation of (*S*)-aziridine from the olefin-bound intermediate. The fact that the simple planar chiral indenyl catalyst can induce such high levels of enantioselectivity in both the previously reported allylic amidation reaction and this mechanistically distinct aziridination reaction suggests that it is a versatile catalyst platform with significant potential for further development.

## Supporting Information Available

Experimental procedures, characterization data, spectra, and details of the computational studies (PDF)
Cartesian coordinates (XYZ)

## Supplementary Material

ja3c10637_si_001.pdf

ja3c10637_si_002.xyz
