# Supplementary material for: Enantioselective Aziridination of Unactivated Terminal Alkenes Using a Planar Chiral Rh(III) Indenyl Catalyst
Source: J Am Chem Soc. 2024 Jan 3;146(2):1447–54. doi: 10.1021/jacs.3c10637 (PMC10797617; doi:10.1021/jacs.3c10637)
Supplement: Supplementary file 1 — ja3c10637_si_001.pdf [file ja3c10637_si_001.pdf]

Supporting Information

for

Enantioselective Aziridination of Unactivated Terminal Alkenes Using a Planar Chiral Rh(III) Indenyl Catalyst

Patrick Gross[1], Hoyoung Im[2,3], David Laws III[1], Bohyun Park[3,2], Mu-Hyun Baik[3,2,*], and Simon B. Blakey[1,*]

[1]Department of Chemistry, Emory University, Atlanta, Georgia 30322, USA

[2]Department of Chemistry, Korea Advanced Institute of Science and Technology (KAIST), Daejeon 34141, Republic of Korea

[3]Center for Catalytic Hydrocarbon Functionalizations, Institute for Basic Science (IBS), Daejeon 34141, Republic of Korea

*email: sblakey@emory.edu

*email: mbaik2805@kaist.ac.kr

Table of Contents

1. General Information 2
2. Catalyst Preparation 3
3. Preparation of Starting Materials 11
3.1. Synthesis of Nitrogen Sources 12
3.2. Synthesis of Alkene Substrates 13
4. Enantioselective Aziridinations 19
5. Determination of Aziridine Product Stereochemistry 30
6. Computational Data 33
6.1. Computational Details 33
6.2. Olefin Concerted Metalation-Deprotonation (CMD) Pathway 35
6.3. Calculated Nitrene Formation Pathways 36
6.4. Analysis of Olefin Migratory Insertion Step 37
6.5. Solvation Modeling of Cs^+ and Explicit Solvent Molecules 39
6.6. Computed Energy Components for Optimized Structures 40
6.7. Vibrational Frequencies (in cm^{-1}) of the Optimized Structures 42
7. References 50
8. NMR and HPLC Data 52

1. General Information

All reactions were conducted under nitrogen atmosphere with anhydrous solvents in oven- or flame-dried glassware using standard Schlenk technique, unless otherwise stated. Anhydrous dichloromethane (DCM), diethyl ether (Et_2O), tetrahydrofuran (THF), and were obtained by passage through activated alumina using a *Glass Contours* solvent purification system. 1,1,1,3,3,3-hexaflouroisopropanol (HFIP) was distilled over activated 4Å molecular sieves and stored over activated 4Å molecular sieves. Solvents for workup, extraction, and column chromatography were used as received from commercial suppliers without further purification. All catalysts were stored and weighed in a nitrogen-filled glovebox. All other chemicals were purchased from Millipore Sigma, Strem Chemicals, Oakwood Chemicals, Alfa Aesar, TCI, Combi Blocks, or Ambeed and used as received without further purification, unless otherwise stated.

^{1}H and ^{13}C nuclear magnetic resonance (NMR) spectra were recorded on a Varian Inova 600 spectrometer (600 MHz ^{1}H, 151 MHz ^{13}C), ^{13}C 600 spectrometer (600 MHz ^{1}H, 151 MHz ^{13}C), a Varian Inova 500 spectrometer (500 MHz ^{1}H, 126 MHz ^{13}C), a Bruker 400 spectrometer (400 MHz ^{1}H, 126 MHz), and a Varian Inova 400 spectrometer (400 MHz ^{1}H, 126 MHz ^{13}C). Chemical shifts δ values were reported in parts per million (ppm) relative to $CHCl_3$ (7.26 ppm for ^{1}H, 77.16 ppm for ^{13}C) for $CDCl_3$, relative to C_6H_6 ((7.16 ppm for ^{1}H, 128.06 ppm for ^{13}C) for C_6D_6, relative to DMSO (2.50 ppm for ^{1}H, 39.52 ppm for ^{13}C) for DMSO-d_6. Coupling constants (J values) were reported in Hz and multiplicities were indicated using the following abbreviations: s = singlet, d = doublet, t = triplet, q = quartet, qn = quintet, m = multiplet, br = broad. High resolution mass spectra (HRMS) were obtained using a Thermo Electron Corporation Finigan LTQFTMS (at the Mass Spectrometry Facility, Emory University). High Pressure Liquid Chromatography (HPLC) was performed on an Agilent 1100 series HPLC utilizing CHIRALPAK® AD-H, AS-H, CHIRALCEL® OD-H and OJ-H 4.6 x 150 mm analytical columns or on an Agilent 1260 Infinity II series HPLC utilizing CHIRALPAK® IA, IB, IH, IJ, and IK 4.6 x 150 mm analytical columns. Semi preparative HPLC was performed on an Agilent 1260 Infinity II series preparative HPLC using a CHIRALCEL® OD-H 20 x 250 mm column. Optical rotations were measured on a PerkinElmer 341 polarimeter. Analytical thin layer chromatography (TLC) was performed on precoated glass-backed Silicycle SiliaPureR 0.25 mm silica gel 60 plates and visualized with UV light or ethanolic *p*-anisaldehyde. Silica gel column chromatography was performed using Silicycle SiliaFlashR F60 silica gel (40- 63 μm). Flash column chromatography was performed using Silicycle SiliaFlashR F60 silica gel (40- 63 μm) on a Biotage Isolera One system. Preparatory TLC was performed on precoated glass backed Silicycle SiliaPureR 1.0 mm silica gel 60 plates.

2. Catalyst Preparation

$[Ind^{*}RhCl_2]_2$ catalyst was synthesized following a reported literature procedure[1].

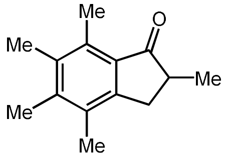

2,4,5,6,7-pentamethyl-2,3-dihydro-1H-inden-1-one (**S1**): Using an adapted procedure from by O'Hare[2]. Under a N_2 atmosphere, dry CH_2Cl_2 (50 mL) was added to 250 mL round bottom flask equipped with a stir bar. Oxalyl chloride (3.0 mL 34.4 mmol, 1.1 equiv.), methacrylic acid (2.9 mL, 34.4 mmol, 1.1 equiv.), and five drops of DMF were sequentially added and the reaction was left to stir vigorously overnight at room temperature. The reaction was placed in an ice bath before quickly adding $AlCl_3$ (5.7 g, 43.0 mmol, 1.4 equiv.). After five minutes, 1,2,3,4-tetramethylbenzene (4.5 mL, 30.2 mmol, 1.0 equiv.) was slowly added rapidly turning the reaction to a dark red orange. The reaction was allowed to stir at room temperature for an additional 4 hours before pouring into a (1:1) HCl: ice slurry. The resulting biphasic solution was extracted with CH_2Cl_2 3x. The combined organic layers were washed with DI H_2O 3x and dried over Na_2SO_4. The solvent was removed under reduced pressure and the crude product purified using silica gel column chromatography (0-5% EtOAc in hexanes) to afford **S1** (5.79 g, 98% yield) as an off-white solid. **^{1}H NMR** (400 MHz, $CDCl_3$) δ 3.22 (dd, *J* = 16.8, 8.0 Hz, 1H), 2.69 – 2.58 (m, 4H), 2.51 (dd, *J* = 16.8, 4.0 Hz, 1H), 2.29 (s, 3H), 2.24 (d, *J* = 2.4 Hz, 6H), 1.29 (d, *J* = 7.4 Hz, 3H). **^{13}C NMR** (151 MHz, $CDCl_3$) δ 211.18, 150.57, 142.06, 135.29, 134.54, 131.13, 130.74, 42.53, 33.68, 17.14, 16.94, 15.50, 15.28, 13.93. **HRMS** (+APCI) calculated for $C_{14}H_{19}O$ $[M+H]^+$ 203.14304, found 203.1427

General Procedure A for Ligand Synthesis

A three neck round bottom flask equipped with a stir bar and a condenser was placed under a N_2 atmosphere. The corresponding indanone (1.0 equiv.) was transferred as a solution in THF (0.3-0.5M) and the reaction vessel was placed in an ice bath to cool to 0 °C. Once cooled, the corresponding Grignard reagent (3.0 equiv.) was slowly added to the reaction. Once the addition was complete the ice bath was removed and replaced with a heating block to reflux the reaction for 16 hours. The reaction was cooled to room temperature and placed in an ice bath before carefully quenching with DI H_2O. Concentrated HCl was added to the reaction vessel and stirred for an additional 24 hours at room temperature. The layers of the biphasic solution were separated, and the aqueous layer extracted with Et_2O 3x. The combined organic layers were sequentially washed with $NaHCO_3$, DI H_2O and brine before being dried over $MgSO_4$. The solvent was removed under reduced pressure and the resulting crude product purified via silica gel column chromatography (0-5% EtOAc in hexanes).

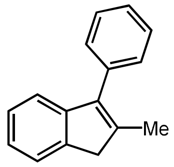

2-methyl-3-phenyl-1H-indene **(S2)** Prepared according to **General Procedure A** using 2-methylindan-1-one[3] (3.94 g, 26.9 mmol, 1.0 equiv) and phenyl magnesium bromide (36 mL, 2.0M, 75 mmol, 2.8 equiv.). (**S2**) (4.78 g, 86% yield) Spectroscopic data for **S2** matches those previously reported in the literature[4].

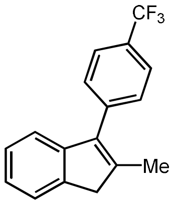

2-methyl-3-(4-(trifluoromethyl)phenyl)-1H-indene **(S3)** Prepared according to **General Procedure A** using 2-methylindan-1-one[3] (0.290 g, 2.0 mmol, 1.0 equiv) and freshly prepared (4-(trifluoromethyl)phenyl)magnesium bromide (1.09 g, 4.4 mmol, 2.2 equiv.). (**S3**) (0.361 g, 66% yield) ^{1}**H NMR** (600 MHz, $CDCl_3$) δ 7.73 (d, *J* = 8.2 Hz, 2H), 7.52 (d, *J* = 8.2 Hz, 1H), 7.49 – 7.44 (m, 1H), 7.28 – 7.22 (m, 1H), 7.22 – 7.14 (m, 2H), 3.50 (s, 2H), 2.15 (s, 3H). ^{13}**C NMR** (151 MHz, $CDCl_3$) δ 145.83, 142.42, 142.17, 139.47, 137.69, 129.59, 129.14 (q, *J* = 32.2 Hz), 126.47, 125.55 (q, *J* = 3.7 Hz), 124.49, 124.47 (q, *J* = 271.9 Hz), 123.70, 43.40, 14.95. ^{19}**F NMR** (564 MHz, $CDCl_3$) δ -62.40. **HRMS** (+APCI) calculated for $C_{17}H_{14}F_3$ $[M+H]^+$ 275.10421, found 275.10404.

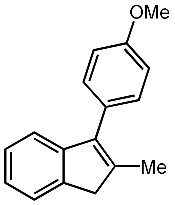

3-(4-methoxyphenyl)-2-methyl-1H-indene **(S4)** Prepared according to **General Procedure A** using 2-methylindan-1-one[3] (0.500 g, 3.42 mmol, 1.0 equiv) and freshly prepared (4-methoxyphenyl)magnesium bromide (1.45 g, 6.84 mmol, 2.0 equiv.). (**S4**) (0.537 g, 66% yield) ^{1}**H NMR** (600 MHz, $CDCl_3$) δ 7.44 (d, *J* = 7.3 Hz, 1H), 7.36 (d, *J* = 8.4 Hz, 2H), 7.25 (d, *J* = 4.2 Hz, 2H), 7.17 (dq, *J* = 7.8, 4.0 Hz, 1H), 7.02 (d, *J* = 8.6 Hz, 2H), 3.88 (s, 3H), 3.45 (s, 2H), 2.15 (s, 3H). ^{13}**C NMR** (151 MHz, $CDCl_3$) δ 158.69, 146.71, 142.55, 140.15, 138.25, 130.37, 127.95, 126.26, 124.01, 123.49, 119.36, 113.99, 55.41, 43.14, 15.00. **HRMS** (+APCI) calculated for $C_{17}H_{17}O$ $[M+H]^+$ 237.12739, found 237.12732.

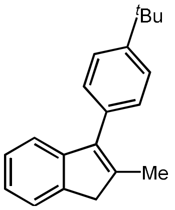

3-(4-(tert-butyl)phenyl)-2-methyl-1H-indene **(S5)** Prepared according to **General Procedure A** 2-methylindan-1-one[3] (0.500 g, 3.42 mmol, 1.0 equiv) and freshly prepared (4-(tert-butyl)phenyl)magnesium bromide (3.25 g, 13.7 mmol, 4.0 equiv.). (**S5**) (0.440 g, 49% yield) ^{1}**H NMR** (600 MHz, $CDCl_3$) δ 7.49 (d, *J* = 8.3 Hz, 2H), 7.44 (d, *J* = 7.3 Hz, 1H), 7.36 (d, *J* = 8.3 Hz, 2H), 7.29 (d, *J* = 7.5 Hz, 1H), 7.24 (t, *J* = 7.4 Hz, 1H), 7.16 (td, *J* = 7.3, 1.2 Hz, 1H), 3.46 (s, 2H), 2.17 (s, 3H), 1.40 (s, 9H). ^{13}**C NMR** (151 MHz, $CDCl_3$) δ 149.85, 146.63, 142.58, 140.45, 138.50, 132.56, 128.89, 126.22, 125.40, 123.98, 123.47, 119.56, 43.25, 34.76, 31.56, 15.10. **HRMS** (+APCI) calculated for $C_{20}H_{23}$ $[M+H]^+$ 263.17943, found 263.17932.

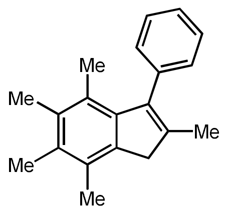

2,4,5,6,7-pentamethyl-3-phenyl-1H-indene **(S6)**: Prepared according to **General Procedure A** using **S1** (0.608 g, 3.0 mmol, 1.0 equiv) and phenyl magnesium bromide (11.5 mL, 0.8 M, 9.2 mmol, 3.0 equiv.). (**S6**) (0.415 g, 57% yield) ^{1}**H NMR** (400 MHz, $CDCl_3$) δ 7.42 (tt, J = 8.1, 1.6 Hz, 4H), 7.35 (t, J = 7.3 Hz, 1H), 7.27 – 7.22 (m, 2H), 3.35 (s, 2H), 2.33 (s, 3H), 2.29 (s, 3H), 2.20 (s, 3H), 1.92 (s, 3H), 1.83 (s, 3H). ^{13}**C NMR** (101 MHz, $CDCl_3$) δ 141.48, 140.80, 140.44, 139.68, 139.11, 134.17, 130.88, 129.70, 128.67, 128.23, 126.80, 126.69, 42.28, 16.56, 16.38, 16.28, 16.20, 14.84. **HRMS** (+APCI) calculated for $C_{20}H_{23}$ $[M+H]^+$ 263.17943, found 263.18007.

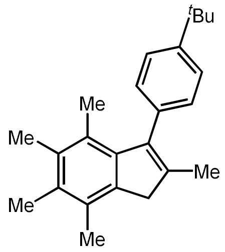

3-(4-(tert-butyl)phenyl)-2,4,5,6,7-pentamethyl-1H-indene **(S7)**: Prepared according to **General Procedure A** using **S1** (1.17 g, 5.8 mmol, 1.0 equiv) and (4-(tert-butyl)phenyl)magnesium bromide (23 mL, 0.75 M, 17 mmol, 3.0 equiv.). (**S7**) (0.987 g, 53% yield) ^{1}**H NMR** (400 MHz, $CDCl_3$) δ 7.41 (d, J = 8.3 Hz, 2H), 7.16 (d, J = 8.3 Hz, 2H), 3.33 (s, 2H), 2.33 (s, 3H), 2.28 (s, 3H), 2.20 (s, 3H), 1.93 (s, 3H), 1.84 (s, 3H), 1.39 (s, 9H). ^{13}**C NMR** (101 MHz, $CDCl_3$) δ 149.47, 141.63, 140.74, 140.36, 139.14, 136.38, 134.14, 130.75, 129.25, 128.59, 126.93, 125.00, 42.25, 34.67, 31.62, 16.55, 16.41, 16.27, 16.19, 14.96. **HRMS** (+APCI) calculated for $C_{24}H_{31}$ $[M+H]^+$ 319.24203, found 319.24142

General Procedure B for catalyst complexation

In a nitrogen filled glovebox, indene ligand (2.2 equiv.), KO^{t}Bu (3.0 equiv.) and $[Rh(COD)Cl]_2$ (1.0 equiv.) were weighed out into a 15 mL vial equipped with a stir bar. The vial was sealed with a Teflon septum screw capped and brought out of the box. Outside the glovebox, anhydrous THF (0.3-0.5M) was added, and the reaction was stirred overnight under a N_2 balloon at room temperature. The reaction was filtered through a celite pipette plug flushing with hexanes and the filtrate concentrated under reduced pressure. The resulting crude product was columned via silica gel column chromatography using deacidified silica gel (1% Et_3N in hexanes, flushing with 100% hexanes before loading and eluting the yellow product band with 100% hexanes) to provide the Rh(I)Indenyl complex. Separation of the planar chiral enantiomers was conducted via chiral semipreparative HPLC.

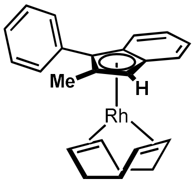

(±)-1,5-cyclooctadiene(η^5-2-methyl-3-phenylinden-1H-yl)rhodium(I)(±-**S8**). Prepared according to **General Procedure B** using *2-methyl-3-phenyl-1H-indene*[4] (120 mg, 0.58 mmol, 2.2 equiv.), KO^{t}Bu (97 mg, 0.86 mmol, 3.2 equiv.), and $[Rh(COD)Cl]_2$ (128 mg, 0.26 mmol, 1.0 equiv.). (±-**S8**) (0.2069 g, 96% yield) yellow oil. Spectroscopic data for **(±)-S8** matches those previously reported in the literature.[4]

Chiral Resolution: **Analytical HPLC**: (OD-H column 0% 2-propanol in hexanes, 1.0 mL/min) (***(S)*-S8**): t_1 = 14.3 min, (***(R)*-S8**): t_2 = 21.1 min, **Semi-prep HPLC**: 20 x 250 mm Chiracel OD-H column, 0% 2-Propanol in Hexanes, 20 mL/min 900 μL injections of 22 mg/mL solutions were made to resolve the complex.

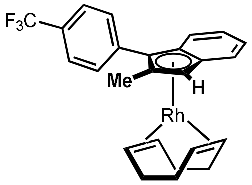

(±)-1,5-cyclooctadiene(η^5-2-methyl-3-(4-(trifluoromethyl)phenyl)inden-1H-yl)rhodium(I) (±-**S9**). Prepared according to **General Procedure B** using **S3** (175 mg, 0.64 mmol, 2.1 equiv.), KO^{t}Bu (85 mg, 0.76 mmol, 2.5 equiv.), and $[Rh(COD)Cl]_2$ (150 mg, 0.30 mmol, 1.0 equiv.). (±-**S9**) (0.0852 g, 75% yield). **^{1}H NMR** (600 MHz, C_6D_6) δ 7.45 (d, J = 8.2 Hz, 2H), 7.27 (d, J = 7.8 Hz, 2H), 7.19 (ddt, J = 9.2, 8.2, 1.1 Hz, 2H), 7.11 – 7.05 (m, 2H), 4.62 (s, 1H), 3.82 (qd, J = 4.8, 2.2 Hz, 2H), 3.62 (ddd, J = 8.0, 5.6, 2.5 Hz, 2H), 2.23 (d, J = 1.3 Hz, 3H), 1.87 – 1.77 (m, 4H), 1.71 – 1.60 (m, 4H). **^{13}C NMR** (151 MHz, C_6D_6) δ 139.67, 129.68, 125.73 (q, J = 3.8 Hz), 124.32 (q, J = 271.6 Hz), 123.57, 122.78, 119.77, 117.37, 112.81 (d, J_{C-Rh} = 2.1 Hz), 112.49 (d, J_{C-Rh} = 2.8 Hz), 108.08 (d, J_{C-Rh} = 5.0 Hz), 94.09 (d, J_{C-Rh} = 3.8 Hz), 77.50 (d, J_{C-Rh} = 4.8 Hz), 72.51 (d, J_{C-Rh} = 13.7 Hz), 69.67 (d, J_{C-Rh} = 13.7 Hz), 31.78, 31.64, 14.69. **^{19}F NMR** (564 MHz, C_6D_6) δ -61.98. **HRMS** (+APCI) calculated for $C_{25}H_{24}F_3Rh$ $[M]^+$ 484.08797, found 484.0894.

Chiral Resolution: **Analytical HPLC** (Chiracel AD-H column 100% Hexanes, 1.0 mL/min) (***(S)*-S9**): t_1 = 4.6 min, (***(R)*-S9**): t_2 = 7.1 min, **Semi-prep HPLC** (20 x 250 mm Chiracel OD-H column, 100% Hexanes, 10 mL/min 250 μL injections of 20 mg/mL solutions were made to resolve the complex.

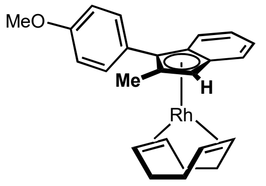

(±)-1,5-cyclooctadiene(η^5-3-(4-methoxyphenyl)-2-methylinden-1H-yl)rhodium(I)(±-**S10**): Prepared according to **General Procedure B** using **S4** (53 mg, 0.23 mmol, 2.2 equiv.), KO^{t}Bu (39 mg, 0.35 mmol, 3.4 equiv.), and $[Rh(COD)Cl]_2$ (59 mg, 0.12 mmol, 1.0 equiv.). (±-**10**) (0.0731 g, 80% yield). **^{1}H NMR** (600 MHz, C_6D_6) δ 7.43 – 7.39 (m, 1H), 7.38 (d, *J* = 8.2 Hz, 2H), 7.25 – 7.21 (m, 1H), 7.13 – 7.08 (m, 2H), 6.88 (d, *J* = 8.2 Hz, 2H), 4.68 (s, 1H), 3.94 (td, *J* = 7.5, 3.4 Hz, 2H), 3.77 (tt, *J* = 7.6, 3.1 Hz, 2H), 3.37 (s, 3H), 2.38 (s, 3H), 1.96 – 1.83 (m, 4H), 1.78 – 1.65 (m, 4H). **^{13}C NMR** (151 MHz, C_6D_6) δ 158.78, 130.77, 127.43, 123.15, 122.41, 119.66, 117.96, 114.42, 112.97 (d, $J_{C–Rh}$ = 2.3 Hz), 112.07 (d, $J_{C–Rh}$ = 2.7 Hz), 107.69 (d, $J_{C–Rh}$ = 5.0 Hz), 96.06 (d, $J_{C–Rh}$ = 3.7 Hz), 76.77 (d, $J_{C–Rh}$ = 4.9 Hz), 72.03 (d, $J_{C–Rh}$ = 13.7 Hz), 69.12 (d, $J_{C–Rh}$ = 13.8 Hz), 54.86, 31.91, 31.78, 14.82. **HRMS** (+APCI) calculated for $C_{25}H_{27}ORh$ $[M]^+$ 446.11115, found 446.1111.

Chiral Resolution: **Analytical HPLC:** Chiracel OD-H column 100% Hexanes, 1.0 mL/min ((***S***)-**10**): t_1 = 34.2 min, ((***R***)-**10**): t_2 = 50.9 min, **Semi-prep HPLC**: 20 x 250 mm Chiracel OD-H column, 100% Hexanes, 20 mL/min 900 µL injections of 20 mg/mL solutions were made to resolve the complex

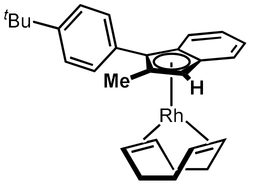

(±)-1,5-cyclooctadiene(η^5-3-(4-(tert-butyl)phenyl-2-methylinden-1H-yl)rhodium(I)(±-**S11**). Prepared according to **General Procedure B** using **S5** (67 mg, 0.25 mmol, 2.2 equiv.), KO^{t}Bu (41mg, 0.37 mmol, 3.2 equiv.), and $[Rh(COD)Cl]_2$ (68 mg, 0.14 mmol, 1.0 equiv.). (±-**11**) (0.0852 g, 78% yield). **^{1}H NMR** (600 MHz, C_6D_6) δ 7.47 – 7.45 (m, 2H), 7.45 – 7.41 (m, 1H), 7.41 – 7.37 (m, 2H), 7.24 – 7.20 (m, 1H), 7.12 – 7.06 (m, 2H), 4.67 (s, 1H), 3.94 (dp, *J* = 7.2, 3.0 Hz, 2H), 3.77 (ddt, *J* = 7.8, 6.2, 1.9 Hz, 2H), 2.40 (d, *J* = 1.4 Hz, 3H), 1.92 – 1.82 (m, 4H), 1.75 – 1.63 (m, 4H), 1.29 (s, 9H). **^{13}C NMR** (151 MHz, C_6D_6) δ 149.19, 132.57, 129.47, 125.77, 123.18, 122.43, 119.68, 113.04 (d, $J_{C–Rh}$ = 2.3 Hz), 112.18 (d, $J_{C–Rh}$ = 2.7 Hz), 107.81 (d, $J_{C–Rh}$ = 5.0 Hz), 96.04 (d, $J_{C–Rh}$ = 3.8 Hz), 76.95 (d, $J_{C–Rh}$ = 4.9 Hz), 72.09 (d, $J_{C–Rh}$ = 13.8 Hz), 69.19 (d, $J_{C–Rh}$ = 13.6 Hz), 34.62, 31.81 (d, $J_{C–Rh}$ = 2.2 Hz), 31.50, 14.91. **HRMS** (+APCI) calculated for $C_{28}H_{33}Rh$ $[M]^+$ 472.16318, found 472.16426.

Chiral Resolution: **Analytical HPLC**: Chiracel OD-H column 100% Hexanes, 0.5 mL/min ((***S***)-**11**): t_1 = 13.0 min, ((***R***)-**11**): t_2 = 16.7 min, **Semi-prep HPLC**: 20 x 250 mm Chiracel OD-H column, 100% Hexanes, 20 mL/min 900 µL injections of 20 mg/mL solutions were made to resolve the complex.

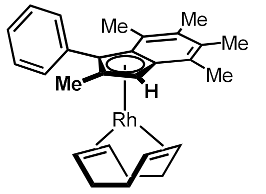

(±)-1,5-cyclooctadiene(η[5]-2,4,5,6,7-pentamethyl-3-phenylinden-1H-yl)rhodium(I)(±-S**12**). Prepared according to **General Procedure B** using **S6** (115 mg, 0.44 mmol, 2.2 equiv.), KO^{t}Bu (67 mg, 0.60 mmol, 3.0 equiv.), and $[Rh(COD)Cl]_2$ (118 mg, 0.24 mmol, 1.0 equiv.). (**±-12**) (0.109 g, 58% yield). **^{1}H NMR** (600 MHz, C_6D_6) δ 7.77 (d, J = 7.7 Hz, 1H), 7.36 – 7.30 (m, 1H), 7.27 – 7.21 (m, 1H), 4.83 (s, 1H), 4.10 (tt, J = 7.8, 3.1 Hz, 2H), 3.88 (tt, J = 7.9, 2.9 Hz, 2H), 2.29 (s, 3H), 2.18 (s, 3H), 2.13 (d, J = 1.2 Hz, 3H), 2.12 (s, 3H), 1.99 (s, 3H), 1.99 – 1.88 (m, 4H), 1.84 – 1.73 (m, 4H). **^{13}C NMR** (151 MHz, C_6D_6) δ 137.80, 132.56, 131.04, 130.39, 126.78, 123.60, 122.87, 111.43 (d, J_{C-Rh} = 2.7 Hz), 109.15 (d, J_{C-Rh} = 2.4 Hz), 107.78 (d, J_{C-Rh} = 4.9 Hz), 99.37 (d, J_{C-Rh} = 4.2 Hz), 75.64 (d, J_{C-Rh} = 4.8 Hz), 69.25 (d, J_{C-Rh} = 13.8 Hz), 68.95 (d, J_{C-Rh} = 13.9 Hz), 32.56, 31.98, 18.14, 16.56, 16.51, 16.24, 13.84. **HRMS** (+APCI) calculated for $C_{28}H_{33}Rh$ $[M]^+$ 472.16318, found 472.16342.

Chiral Resolution: **Analytical HPLC** (Chiracel OD-H column 100% Hexanes, 1.0 mL/min) ((***R*)-12**): t_1 = 4.5 min, ((***S*)-12**): t_2 = 5.7 min, **Semi-prep HPLC** (20 x 250 mm Chiracel OD-H column, 100% Hexanes, 20 mL/min 250 µL injections of 20 mg/mL solutions were made to resolve the complex.

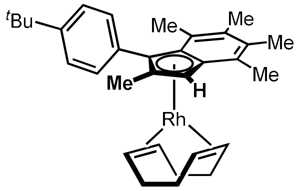

(±)-1,5-cyclooctadiene(η[5]-3-(4-(tert-butyl)phenyl)-2,4,5,6,7-pentamethylinden-1H-yl)rhodium(I) (±-**S13**). Prepared according to **General Procedure B** using **S7** (115 mg, 0.44 mmol, 2.2 equiv.), KO^{t}Bu (64 mg, 0.60 mmol, 3.0 equiv.), and $[Rh(COD)Cl_2]$ (118 mg, 0.24 mmol, 1.0 equiv.). (±-**S13**) (0.108 g, 58% yield). **^{1}H NMR** (600 MHz, $CDCl_3$) δ 7.75 (dd, J = 8.0, 1.9 Hz, 1H), 7.45 (dd, J = 8.0, 2.1 Hz, 1H), 7.29 (qd, J = 8.1, 2.0 Hz, 2H), 4.85 (s, 1H), 4.14 (td, J = 7.5, 3.7 Hz, 2H), 3.92 (ddd, J = 10.6, 7.7, 2.9 Hz, 2H), 2.30 (s, 3H), 2.19 (s, 3H), 2.17 (d, J = 1.2 Hz, 3H), 2.12 (s, 3H), 2.04 (s, 3H), 2.03 – 1.99 (m, 2H), 1.99 – 1.91 (m, 2H), 1.86 – 1.76 (m, 4H), 1.29 (s, 9H). **^{13}C NMR** (151 MHz, $CDCl_3$) δ 149.39, 134.78, 132.32, 130.82, 130.32, 125.00, 124.91, 123.74, 122.84, 111.41 (d, J_{C-Rh} = 2.6 Hz), 109.26 (d, J_{C-Rh} = 2.6 Hz), 107.77 (d, J_{C-Rh} = 4.6 Hz), 99.21 (d, J_{C-Rh} = 4.3 Hz), 75.63 (d, J_{C-Rh} = 4.9 Hz), 69.12 (d, J_{C-Rh} = 13.8 Hz), 68.98 (d, J_{C-Rh} = 13.8 Hz), 34.61, 32.58, 32.06, 31.57, 18.20, 16.56, 16.52, 16.25, 14.02. **HRMS** (+APCI) calculated for $C_{32}H_{42}Rh$ $[M+H]^+$ 529.23361, found 529.2339.

The planar chiral enantiomers of (±-**13**) were not found to separate sufficiently on chiral HPLC to allow for the clean chiral resolution of this complex.

General Procedure C for Precatalysts Synthesis

With no precautions against air or moisture exposure, I_2 crystals (2.5 euqiv.) were added to an 8 dram vial equipped with a stir bar and containing a solution of resolved (*S,S*)-Ind^{X}Rh(I)(COD) complex (1.0 equiv.) in Et_2O (0.03M). The vial was capped to prevent solvent evaporation and the black solution was stirred for 24 hours. The reaction was filtered through a Buchner funnel washing with excess Et_2O until the filtrate was clear. The fine black powder was carefully collected from the filter paper to give the (*S,S*)-[Ind^{X}Rh(III)I_2]$_2$ precatalyst which was stored in a nitrogen filled glovebox.

(*R,R*)-[Ind^{X}Rh(III)I_2]$_2$ precatalysts and (±)-[Ind^{X}Rh(III)I_2]$_2$ precatalysts were synthesized in the same manner.

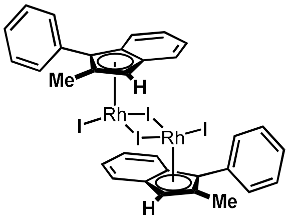

(S,S)-(η^5-2-methyl-3-phenylinden-1H-yl) rhodium(III) diiodide dimer (**(*S,S*)-2**): Prepared according to **General Procedure C** using (*S*)-**S8** (97 mg, 0.23 mmol, 1.0 equiv.) and I_2 (148 mg, 0.58 mmol, 2.5 equiv.). **(*S,S*)-2** (120 mg, 93% yield) Fine black powder. Spectroscopic data for **(*S,S*)-2** matches those previously reported in the literature[4].

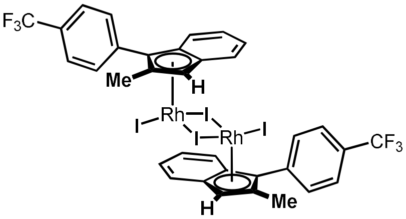

(S,S)-(η^5-2-methyl-3-(4-(trifluoromethyl)phenyl)inden-1H-yl) rhodium(III) diiodide dimer (**(*S,S*)-5**): Prepared according to **General Procedure C** using **(*S*)-S9** (49 mg, 0.10 mmol, 1.0 equiv.) and I_2 (64 mg, 0.25 mmol, 2.5 equiv.). **(*S,S*)-5** (37 mg, 58% yield) Fine black powder. **^{1}H NMR** (600 MHz, DMSO-d$_6$) δ 8.06 (d, *J* = 8.0 Hz, 4H), 7.88 (d, *J* = 8.0 Hz, 4H), 7.71 (d, *J* = 8.5 Hz, 2H), 7.63 (t, *J* = 7.5 Hz, 2H), 7.59 (t, *J* = 7.6 Hz, 2H), 7.44 (d, *J* = 8.4 Hz, 2H), 6.54 (s, 2H), 2.27 (s, 6H). **^{13}C NMR** (151 MHz, DMSO-d$_6$) δ 134.06, 133.73, 132.67, 129.01 (q, *J* = 31.9 Hz), 127.62, 125.39, 125.31 (q, *J* = 4.0 Hz), 124.18 (q, *J* = 272.1 Hz), 112.10 (d, $J_{C–Rh}$ = 5.4 Hz), 107.09 (d, $J_{C–Rh}$ = 3.7 Hz), 104.34 (d, $J_{C–Rh}$ = 4.1 Hz), 92.91 (d, $J_{C–Rh}$ = 5.6 Hz), 78.28 (d, $J_{C–Rh}$ = 6.7 Hz), 13.46. **^{19}F NMR** (565 MHz, DMSO-d$_6$) δ -61.09. **HRMS** (+APCI) calculated for $C_{17}H_{12}F_3{}^{127}I_3{}^{103}Rh$ [M-$C_{17}H_{12}IRh$]$^-$ 756.70857, found 756.70865

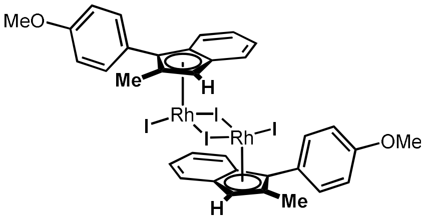

(S,S)-(η^5-3-(4-methoxyphenyl)-2-methylinden-1H-yl) rhodium(III) diiodide dimer (**(*S,S*)-6**): Prepared according to **General Procedure C** using **(*S*)-S10** (52 mg, 0.12 mmol, 1.0 equiv.) and I_2 (74 mg, 0.29 mmol, 2.5 equiv.). **(*S,S*)-6** (51 mg, 71% yield) Fine black powder. **^{1}H NMR** (600 MHz, DMSO-d$_6$) δ 7.80 (d, *J* = 8.7 Hz, 4H), 7.70 – 7.65 (m, 2H), 7.58 (tt, *J* = 6.7, 5.2 Hz, 4H), 7.47 – 7.41 (m, 2H), 7.06 (d, *J* = 8.7 Hz, 4H), 6.43 (s, 2H), 3.83 (s, 6H), 2.23 (s, 6H). **^{13}C NMR** (151 MHz, DMSO-d$_6$) δ 159.64, 133.48, 132.35, 132.21, 127.83, 125.55, 120.95, 113.98, 110.83 (d, $J_{C–Rh}$ = 5.4 Hz), 107.29 (d, $J_{C–Rh}$ = 3.9 Hz), 103.45 (d, $J_{C–Rh}$ = 4.7 Hz), 95.23 (d, $J_{C–Rh}$ = 5.6 Hz), 77.58 (d, $J_{C–Rh}$ = 7.2 Hz), 55.23, 13.58. **HRMS** (+APCI) calculated for $C_{17}H_{14}OI_2Rh$ [M-$C_{17}H_{16}OI_2Rh$]$^-$ 590.81725, found 590.81689

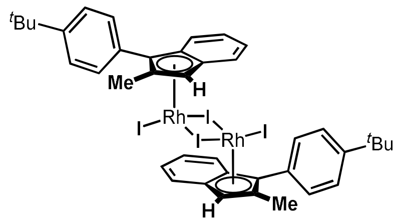

(S,S)-(η^5-3-(4-(tert-butyl)phenyl-2-methylinden-1H-yl) rhodium(III) diiodide dimer (**(*S,S*)-7**): Prepared according to **General Procedure C** using **(*S*)-S11** (43 mg, 0.90 mmol, 1.0 equiv.) and I_2 (57 mg, 0.23 mmol, 2.5 equiv.). **(*S,S*)-7** (39 mg, 72% yield) Fine black powder. **^{1}H NMR** (600 MHz, DMSO-d$_6$) δ 7.79 (d, *J* = 8.3 Hz, 4H), 7.68 (dd, *J* = 8.4, 1.2 Hz, 2H), 7.62 – 7.58 (m, 2H), 7.58 – 7.55 (m, 2H), 7.51 (d, *J* = 8.0 Hz, 4H), 7.45 (d, *J* = 8.5 Hz, 2H), 6.44 (s, 2H), 2.25 (s, 6H), 1.34 (s, 18H). **^{13}C NMR** (151 MHz, DMSO-d$_6$) δ 151.29, 133.48, 130.55, 127.75, 125.67, 125.28, 111.32 (d, J_{C-Rh} = 5.7 Hz), 107.36 (d, J_{C-Rh} = 3.8 Hz), 103.62 (d, J_{C-Rh} = 4.5 Hz), 94.93 (d, J_{C-Rh} = 5.9 Hz), 77.63 (d, J_{C-Rh} = 7.0 Hz), 34.59, 31.03, 13.74. **HRMS** (+APCI) calculated for $C_{20}H_{21}IRh$ [M- $C_{20}H_{21}I_3Rh$]$^+$ 490.97375, found 490.9738

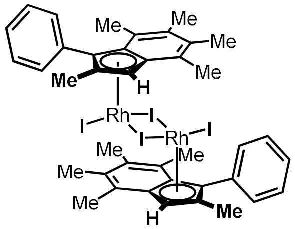

(S,S)-(η^5-2,4,5,6,7-pentamethyl-3-phenylinden-1H-yl) rhodium(III) diiodide dimer (**(*S,S*)-8**): Prepared according to **General Procedure C** using **(*S*)-S12** (32 mg, 0.069 mmol, 1.0 equiv.) and I_2 (44 mg, 0.17 mmol, 2.5 equiv.). **(*S,S*)-8** (42 mg, 98% yield) Fine black powder. ^{1}H NMR (600 MHz, DMSO) δ 8.27 – 8.22 (m, 2H), 7.48 – 7.41 (m, 4H), 7.40 – 7.35 (m, 2H), 7.29 – 7.24 (m, 2H), 6.44 (s, 2H), 2.46 (s, 6H), 2.18 (s, 6H), 2.11 (s, 6H), 2.02 (s, 6H), 1.87 (s, 6H). ^{13}C NMR (151 MHz, DMSO) δ 142.65, 141.60, 133.53, 131.02, 130.69, 129.88, 129.22, 128.47, 128.08, 127.73, 112.53 (d, J_{C-Rh} = 6.0 Hz), 107.15 (d, J_{C-Rh} = 2.9 Hz), 104.92 (d, J_{C-Rh} = 3.7 Hz), 95.91 (d, J_{C-Rh} = 6.9 Hz), 75.08 (d, J_{C-Rh} = 7.0 Hz), 18.25, 17.77, 17.46, 17.27, 13.97. **HRMS** (+APCI) calculated for $C_{40}H_{42}I_3Rh_2$ [M-I]$^+$ 1108.85251, found 1108.85571

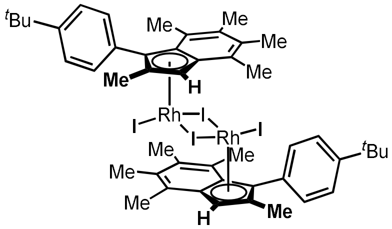

(η^5-3-(4-(tert-butyl)phenyl)-2,4,5,6,7-pentamethylinden-1H-yl) rhodium(III) diiodide dimer (**9**): Prepared according to **General Procedure C** using **S13** (97 mg, 0.23 mmol, 1.0 equiv.) and I_2 (148 mg, 0.58 mmol, 2.5 equiv.). **9** (120 mg, 93% yield) Fine black powder. **^{1}H NMR** (600 MHz, DMSO) δ 8.16 (dd, *J* = 8.2, 2.1 Hz, 1H), 7.48 (dd, *J* = 8.2, 2.1 Hz, 1H), 7.39 (dd, *J* = 8.1, 2.1 Hz, 1H), 7.18 (dd, *J* = 8.1, 2.0 Hz, 1H), 6.42 (s, 1H), 2.46 (s, 3H), 2.18 (s, 3H), 2.11 (s, 3H), 2.01 (s, 3H), 1.88 (s, 3H), 1.33 (s, 9H). **^{13}C NMR** (151 MHz, DMSO) δ 150.82, 142.51, 141.62, 133.11, 130.90, 129.52, 129.09, 127.97, 124.84, 124.45, 112.55 (d, J_{C-Rh} = 5.7 Hz), 107.34 (d, J_{C-Rh} = 2.6 Hz), 104.78 (d, J_{C-Rh} = 3.5 Hz), 96.06 (d, J_{C-Rh} = 6.7 Hz), 74.83 (d, J_{C-Rh} = 6.9 Hz), 34.47, 31.12, 18.30, 17.72, 17.44, 17.24, 14.02. **HRMS** (+APCI) calculated for $C_{48}H_{58}I_3Rh_2$ [M-I]$^+$ 1220.97772, found 1220.98175.

3. Preparation of Starting Materials

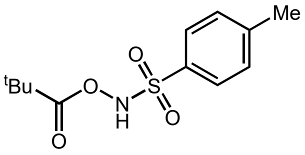

1

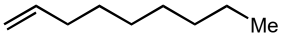

3

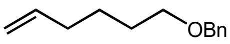

S14

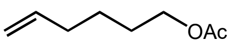

S15

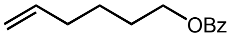

S16

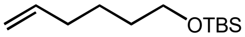

S17

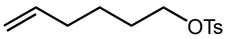

S18

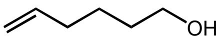

S19

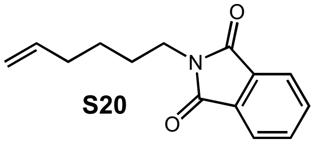

S20

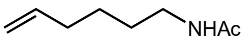

S21

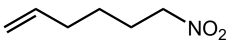

S22

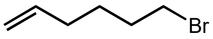

S23

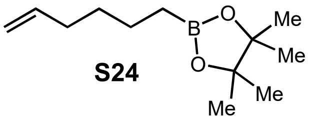

S24

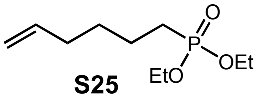

S25

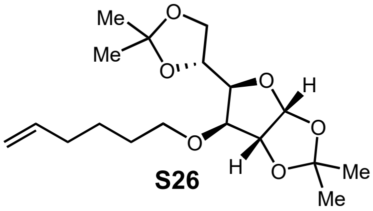

S26

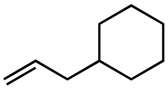

S27

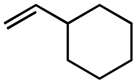

S28

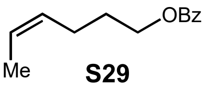

S29

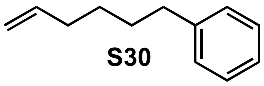

S30

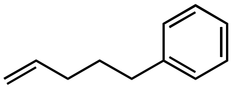

S31

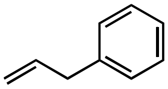

S32

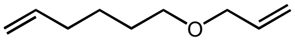

S33

3, **S19**, **S23**, **S27**, **S28**, and **S32** were obtained commercially and used without further purification.

1,[5] **S14**,[6] **S15**,[7] **S16**,[8] **S17**,[9] **S18**,[10] **S20**,[11] **S21**,[12] **S22**,[13] **S24**,[14] **S25**,[15] **S26**,[16] **S29**,[17] **S30**,[18] **S30**,[19] and **S33**[20] were synthesized following reported literature procedures.

3.1. Synthesis of Nitrogen Sources

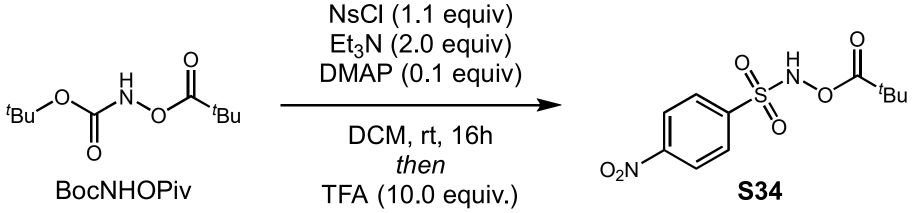

4-nitro-N-(pivaloyloxy)benzenesulfonamide (**S34**): To a solution of BocNHOPiv (0.55 g, 2.5 mmol, 1.0 equiv.) and DMPA (0.040 g, 0.33 mmol, 0.13 equiv.) in DCM (10 mL) was added Et_3N (0.70 mL, 5.0 mmol, 2.0 equiv.) followed by the addition of NsCl (0.61 g, 2.8 mmol, 1.1 equiv.) . The solution was left to stir at room temperature for 16 hours. To the solution, TFA (2.0 mL, 25.0 mmol, 10.0 equiv.) was slowly added and the solution was continued to stir for one hour. The reaction was then diluted with DCM and washed with 1M HCl once. The aqueous layer was extracted a further two times with DCM before the combined organic layers were dried over $MgSO_4$ and the solvent was removed under reduced pressure. The crude product was purified using silica gel column chromatography (10% EtOAc in Hexanes) to obtain **S34** as an off white solid (0.423 g, 55 % yield). **^{1}H NMR** (400 MHz, $CDCl_3$) δ 9.08 (s, 1H), 8.42 (d, J = 8.9 Hz, 2H), 8.15 (d, J = 8.9 Hz, 2H), 1.16 (s, 9H).**^{13}C NMR** (101 MHz, $CDCl_3$) δ 176.75, 151.21, 141.64, 130.35, 124.52, 38.47, 26.90. **HRMS** (+APCI) calculated for $C_{11}H_{13}O_6N_2S$ $[M-H]^-$ 301.04998, found 301.0487.

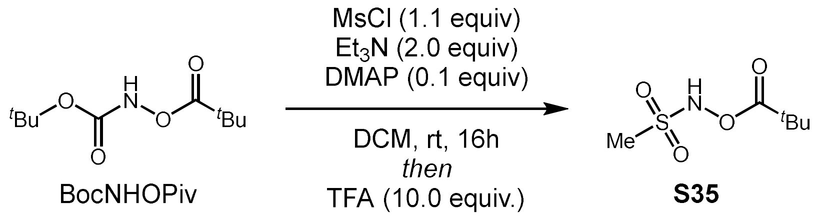

N-(pivaloyloxy)methanesulfonamide (**S35**): To a solution of BocNHOPiv (0.55 g, 2.5 mmol, 1.0 equiv.) and DMPA (0.030 g, 0.25mmol, 0.10 equiv.) in DCM (10 mL) was added Et_3N (0.70 mL, 5.0 mmol, 2.0 equiv.) followed by the addition of MsCl (0.21 mL, 2.7 mmol, 1.1 equiv.) . The solution was left to stir at room temperature for 16 hours. To the solution TFA, (2.0 mL, 25.0 mmol, 10.0 equiv.) was slowly added and the solution was continued to stir for one hour. The reaction was then diluted with DCM and washed with 1M HCl once. The aqueous layer was extracted a further two times with DCM before the combined organic layers were dried over $MgSO_4$ and the solvent was removed under reduced pressure. The crude product was purified using silica gel column chromatography (10% EtOAc in Hexanes) to obtain **S35** as a white solid (0.226 g, 46 % yield). **^{1}H NMR** (400 MHz, $CDCl_3$) δ 8.71 (s, 1H), 3.07 (s, 3H), 1.32 (s, 9H).**^{13}C NMR** (101 MHz, $CDCl_3$) δ 177.16, 38.99, 38.56, 26.98. **HRMS** (+APCI) calculated for $C_6H_{12}O_4NS$ $[M-H]^-$ 194.0493, found 194.0494.

3.2. Synthesis of Alkene Substrates

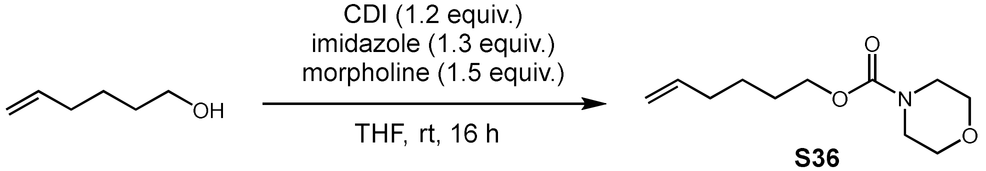

S36

hex-5-en-1-yl morpholine-4-carboxylate (**S36**): To a solution of CDI (0.98 g, 6.0 mmol, 1.2 equiv.) in THF (10 mL) was added hex-5-en-1-ol (0.60 mL, 5.0 mmol, 1.0 equiv.) and solution was stirred for two hours at room temperature. After which, imidazole (0.45 g, 6.6 mmol, 1.3 equiv.) and morpholine (0.66 mL, 7.6 mmol, 1.5 equiv.) were added sequentially before letting the reaction stir for 16 hours. The reaction was diluted with EtOAc and washed with 1M HCl twice, DI H_2O once, and sat. brine once. The organic layer was dried over anhydrous Na_2SO_4, and the solvent removed under reduced pressure to obtain **S36** as colorless oil that did not require further purification (1.10 g, quant.). ^{1}**H NMR** (400 MHz, $CDCl_3$) δ 5.79 (ddt, *J* = 16.9, 10.3, 6.6 Hz, 1H), 5.01 (dt, *J* = 17.1, 1.7 Hz, 1H), 4.96 (ddd, *J* = 10.2, 2.2, 1.1 Hz, 1H), 4.09 (t, *J* = 6.6 Hz, 2H), 3.65 (s, 4H), 3.46 (t, *J* = 4.8 Hz, 4H), 2.08 (q, *J* = 7.2 Hz, 2H), 1.70 – 1.59 (m, 2H), 1.52 – 1.38 (m, 2H). ^{13}**C NMR** (101 MHz, $CDCl_3$) δ 155.60, 138.42, 114.83, 66.63, 65.52, 44.01, 33.34, 28.43, 25.23. **HRMS** (+APCI) calculated for $C_{11}H_{20}O_3N$ $[M+H]^+$ 214.14377, found 214.14361

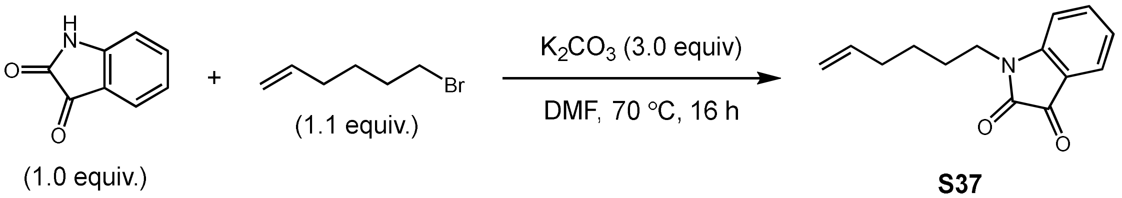

(1.0 equiv.)

S37

1-(hex-5-en-1-yl)indoline-2,3-dione (**S37**): Indoline-2,3-dione (0.77, 5.2 mmol, 1.0 equiv.) and K_2CO_3 (2.1 g, 15.4 mmol, 3.0 equiv.) were added to a flame dried 24mL reaction vial equipped with a stir bar and the atmosphere was exchanged with N_2 three times. DMF (10 mL) was added to the vial and the reaction stirred for 5 minutes before adding 6-bromohex-1-ene (0.84 mL, 6.0 mmol, 1.1 equiv.). The reaction was placed in an aluminium heating block and stirred at 70 °C for 16 hours. The reaction was cooled to room temperature and diluted with EtOAc before washing with DI H_2O four times. The organic layer was dried over anhydrous Na_2SO_4, and the solvent removed under reduced pressure. The crude product was purified using silica gel column chromatography (20% EtOAc in Hexanes) to obtain **S37** as a colorless oil (1.104 g, 92 % yield). ^{1}**H NMR** (400 MHz, $CDCl_3$) δ 7.66 – 7.53 (m, 2H), 7.11 (td, *J* = 7.5, 0.8 Hz, 1H), 6.89 (dt, *J* = 7.8, 0.8 Hz, 1H), 5.77 (ddt, *J* = 16.9, 10.2, 6.7 Hz, 1H), 5.07 – 4.92 (m, 2H), 3.77 – 3.67 (m, 2H), 2.15 – 2.08 (m, 2H), 1.79 – 1.65 (m, 2H), 1.55 – 1.40 (m, 2H).^{13}**C NMR** (101 MHz, $CDCl_3$) δ 183.76, 158.26, 151.13, 138.45, 138.08, 125.63, 123.77, 117.72, 115.36, 110.26, 40.20, 33.33, 26.74, 26.17. **HRMS** (+APCI) calculated for $C_{14}H_{16}O_2N$ $[M+H]^+$ 230.11756, found 230.11746

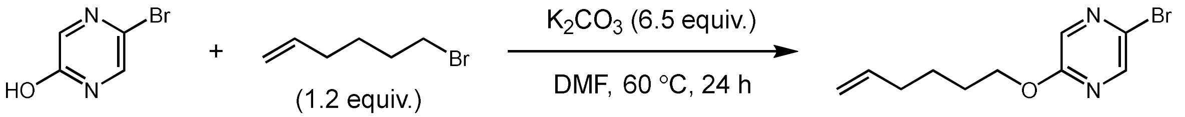

(1.0 equiv.)

S38

2-bromo-5-(hex-5-en-1-yloxy)pyrazine (**S38**): Following a modified procedure[21], 5-bromopyrazin-2-ol (0.88, 5.0 mmol, 1.0 equiv.) and K_2CO_3 (4.5 g, 33 mmol, 6.5 equiv.) were added to a flame dried 22 mL reaction vial equipped with a stir bar and the atmosphere was exchanged with N_2 three times. DMF (10 mL) was added to the vial and the reaction stirred for 20 minutes before adding 6-bromohex-1-ene (0.80 mL, 6.0 mmol, 1.5 equiv.). The reaction was placed in an aluminium heating block and stirred at 60 °C for 24 hours. The reaction was cooled to room temperature and diluted with EtOAc before washing with DI H_2O three times and sat. LiCl once. The organic layer was dried over anhydrous Na_2SO_4 and the solvent removed under reduced pressure. The crude product was purified using silica gel column chromatography (5% EtOAc in Hexanes)

to obtain **S38** as a colorless oil (0.64 g, 50 % yield). **^{1}H NMR** (400 MHz, $CDCl_3$) δ 8.16 (d, *J* = 1.3 Hz, 1H), 7.99 (d, *J* = 1.4 Hz, 1H), 5.81 (ddt, *J* = 17.0, 10.2, 6.7 Hz, 1H), 5.03 (dq, *J* = 17.1, 1.7 Hz, 1H), 4.97 (ddt, *J* = 10.2, 2.2, 1.2 Hz, 1H), 4.29 (t, *J* = 6.6 Hz, 2H), 2.12 (dtd, *J* = 7.6, 6.3, 1.4 Hz, 2H), 1.79 (dq, *J* = 8.1, 6.6 Hz, 2H), 1.54 (p, *J* = 8.0 Hz, 2H). **^{13}C NMR** (101 MHz, $CDCl_3$) δ 159.82, 142.94, 138.44, 135.58, 129.98, 115.05, 67.13, 33.47, 28.26, 25.28. **HRMS** (+APCI) calculated for $C_{10}H_{14}ON_2Br$ $[M+H]^+$ 257.0284, found 257.02895

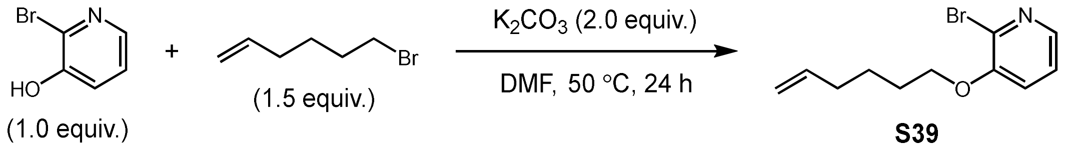

S39

2-bromo-3-(hex-5-en-1-yloxy)pyridine (**S39**): Following a modified procedure[21], 2-bromopyridin-3-ol (0.89, 5.1 mmol, 1.0 equiv.) and K_2CO_3 (1.4 g, 10 mmol, 2.0 equiv.) were added to a flame dried 22 mL reaction vial equipped with a stir bar and the atmosphere was exchanged with N_2 three times. DMF (10 mL) was added to the vial and the reaction stirred for 20 minutes before adding 6-bromohex-1-ene (1.0 mL, 7.5 mmol, 1.5 equiv.). The reaction was placed in an aluminum heating block and stirred at 50 °C for 24 hours. The reaction was cooled to room temperature and diluted with EtOAc before washing with DI H_2O three times and sat. LiCl once. The organic layer was dried over anhydrous Na_2SO_4 and the solvent removed under reduced pressure. The crude product was purified using silica gel column chromatography (10% EtOAc in Hexanes) to obtain **S39** as a colorless oil (1.19 g, 91 % yield). **^{1}H NMR** (400 MHz, $CDCl_3$) δ 7.96 (dd, *J* = 4.6, 1.6 Hz, 1H), 7.19 (dd, *J* = 8.1, 4.6 Hz, 1H), 7.11 (dd, *J* = 8.1, 1.6 Hz, 1H), 5.83 (ddt, *J* = 16.9, 10.2, 6.6 Hz, 1H), 5.05 (dq, *J* = 17.1, 1.7 Hz, 1H), 4.98 (ddt, *J* = 10.2, 2.2, 1.3 Hz, 1H), 4.04 (t, *J* = 6.3 Hz, 2H), 2.15 (qt, *J* = 6.6, 1.4 Hz, 2H), 1.87 (dq, *J* = 8.4, 6.4 Hz, 2H), 1.62 (dq, *J* = 10.1, 7.5 Hz, 2H). **^{13}C NMR** (101 MHz, $CDCl_3$) δ 152.61, 141.21, 138.46, 133.31, 123.46, 119.61, 115.08, 69.26, 33.41, 28.44, 25.31. **HRMS** (+APCI) calculated for $C_{11}H_{15}ONBr$ $[M+H]^+$ 256.03315, found 256.03311

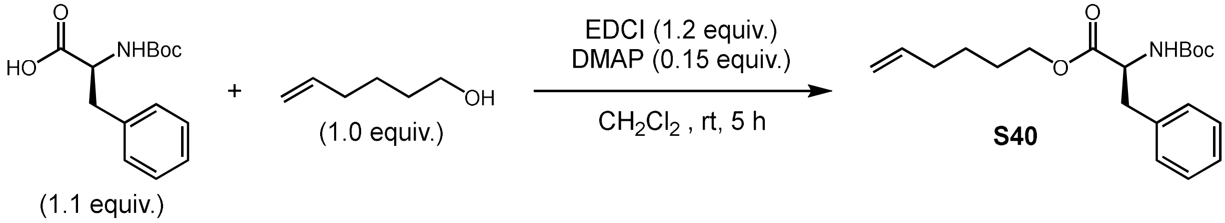

S40

hex-5-en-1-yl (tert-butoxycarbonyl)-L-phenylalaninate (**S40**): In a 24 mL reaction vial equipped with a stir bar under an N_2 atmosphere, hex-5-en-1-ol (0.42 mL, 3.5 mmol, 1.0 equiv.) was added to a solution of (tert-butoxycarbonyl)-L-phenylalanine (1.0 g, 3.8 mmol, 1.1 equiv.) and DMAP (0.06 g, 0.5 mmol, 0.14 equiv.) in CH_2Cl_2 (15 mL). After stirring for five minutes EDCI (0.81 g, 4.3 mmol, 1.2 equiv.) was added and the reaction stirred at room temperature for five hours until complete consumption of the starting material was observed via TLC. The reaction was diluted with EtOAc and washed with 1M HCl, sat. $NaHCO_3$, and brine. The organic layer was dried over anhydrous Na_2SO_4 and the solvent removed under reduced pressure to give **S40** as colorless oil that did not require further purification. (1.21 g, 99% yield.). **^{1}H NMR** (400 MHz, $CDCl_3$) δ 7.33 – 7.20 (m, 3H), 7.13 (d, *J* = 6.6 Hz, 2H), 5.77 (ddt, *J* = 16.9, 10.2, 6.7 Hz, 1H), 5.09 – 4.88 (m, 3H), 4.57 (q, *J* = 6.1 Hz, 1H), 4.09 (td, *J* = 6.7, 2.5 Hz, 2H), 3.08 (t, *J* = 6.0 Hz, 2H), 2.05 (q, *J* = 7.3 Hz, 2H), 1.69 – 1.52 (m, 2H), 1.45 – 1.34 (m, 11H). **^{13}C NMR** (101 MHz, $CDCl_3$) δ 172.11, 155.20, 138.33, 136.21, 129.46, 128.64, 127.12, 115.07, 79.99, 65.40, 54.60, 38.60, 33.35, 28.44, 28.01, 25.18. **HRMS** (+APCI) calculated for $C_{20}H_{30}O_4N$ $[M+H]^+$ 348.21693, found 348.21711. $[\alpha]_D^{22}$ +28.2° (*c* = 7.3, $CHCl_3$)

Synthetic sequence for *(S)-(((3-methylhept-6-en-1-yl)oxy)methyl)benzene* (**S44**):

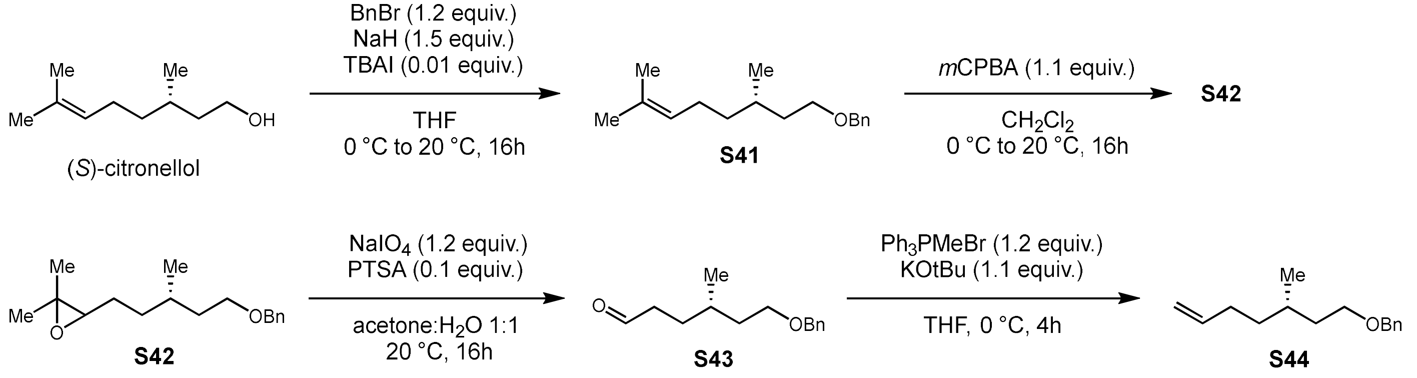

(S)-(((3,7-dimethyloct-6-en-1-yl)oxy)methyl)benzene (**S41**)[22]:

To a 250 mL round bottom flask equipped with a stir bar NaH (60% wt, 1.8 g, 45 mmol, 1.8 equiv.) was added and the atmosphere exchanged with N_2 3x followed by the addition of THF (100 mL). The reaction was placed in an ice bath and (*S*)-citronellol (4.6 mL, 25 mmol, 1.0 equiv.) was slowly added. The reaction was stirred in the ice bath for one hour after which BnBr (3.6 mL, 30 mmol, 1.2 equiv.) and TBAI (92 mg, 0.25mmol, 0.01 euqiv.) were added. The reaction was warmed to room temperature and stirred overnight. After which, the reaction was again placed in an ice bath and quenched with sat. NH_4Cl. The reaction was extracted with EtOAc and washed with DI H_2O and sat. brine. The combined organic layers were dried over anhydrous Na_2SO_4 and solvent removed under reduced pressure to obtain **S41** which was used crude in the next reaction.

3-((S)-5-(benzyloxy)-3-methylpentyl)-2,2-dimethyloxirane (**S42**)[23]:

A 100 mL round bottom flask equipped with a stir bar and containing solution of **S41** (4.25 g, 17.3 mmol, 1.0 equiv.) in CH_2Cl_2 (60 mL) was placed in an ice bath. Once cooled, mCPBA (77 wt%, 4.34 g, 19 mmol, 1.1 equiv.) was added and the reaction left to stir at room temperature overnight. The reaction was diluted with CH_2Cl_2 and washed with sat. $NaHCO_3$. The organic layer was dried over Na_2SO_4 and the solvent removed to yield **S42** which was used crude in the next reaction.

(S)-6-(benzyloxy)-4-methylhexanal (**S43**)[23]:

To solution of **S42** (3.94 g, 15.0 mmol, 1.0 equiv.) in acetone (40 mL) in a 250mL round bottom flask equipped with a stir bar was sequentially added $NaIO_4$ (3.8 g, 18 mmol, 1.2 equiv.), DI H_2O (40 mL), and PTSA (0.328 g, 1.5 mmol, 0.1 equiv.). The reaction mixture was left to stir open to air at room temperature overnight. After which the reaction was filtered to remove the white solid that had formed washing with hexanes. The filtrate was washed with DI H_2O and the organic layer was dried over anhydrous Na_2SO_4. The solvent removed to obtain **S43** which was used without further purification.

(S)-(((3-methylhept-6-en-1-yl)oxy)methyl)benzene (**S44**)[23]:

A 250 mL round bottom flask equipped with a stir bar and containing a solution of $MePPh_3Br$ (6.44 g, 18 mmol, 1.2 equiv.) in THF (80 mL) was placed in an ice bath. Once cooled, added KO^{t}Bu (1.93 g, 17 mmol, 1.1 equiv.) and the yellow suspension was stirred in the ice bath for two hours. After which, a solution of **S43** (3.31 g, 15.0 mmol, 1.0 equiv.) in THF (20 mL) was added slowly and continued ot be stirred in the ice bath. After an additional two hours the reaction was filtered and the filtrate concentrated under recued pressure. The crude product was purified by silica gel column chromatography (0-5% EtOAc in hexanes) to yield **S44** as a colorless oil (1.75g, 32% yield over 4 steps). **^{1}H NMR** (400 MHz, $CDCl_3$) δ 7.38 – 7.26 (m, 5H), 5.81 (ddt, *J* = 16.9, 10.2, 6.6 Hz, 1H), 5.00 (dq, *J* = 17.1, 1.7 Hz, 1H), 4.93 (ddt, *J* = 10.2, 2.3, 1.2 Hz, 1H), 4.51 (s, 2H), 3.51 (ddd, *J* = 9.3, 6.8, 3.0 Hz, 2H), 2.16 – 1.97 (m, 2H), 1.76 – 1.54 (m, 2H), 1.52 – 1.34 (m, 2H), 1.23 (dddd, *J* = 13.4, 9.7, 7.8, 5.8 Hz, 1H), 0.90 (d, *J* = 6.5 Hz, 3H). **^{13}C NMR** (101 MHz, $CDCl_3$) δ 139.34,

138.82, 128.49, 127.75, 127.62, 114.27, 73.05, 68.78, 36.82, 36.41, 31.40, 29.59, 19.63. **HRMS** (+APCI) calculated for $C_{15}H_{23}O$ $[M+H]^+$ 219.17434, found 219.17457. $[\alpha]_D^{22}$ -1.5° (c = 2.1, $CHCl_3$)

Synthetic sequence for *(S)-(((3-methylhex-5-en-1-yl)oxy)methyl)benzene* (**S50**):

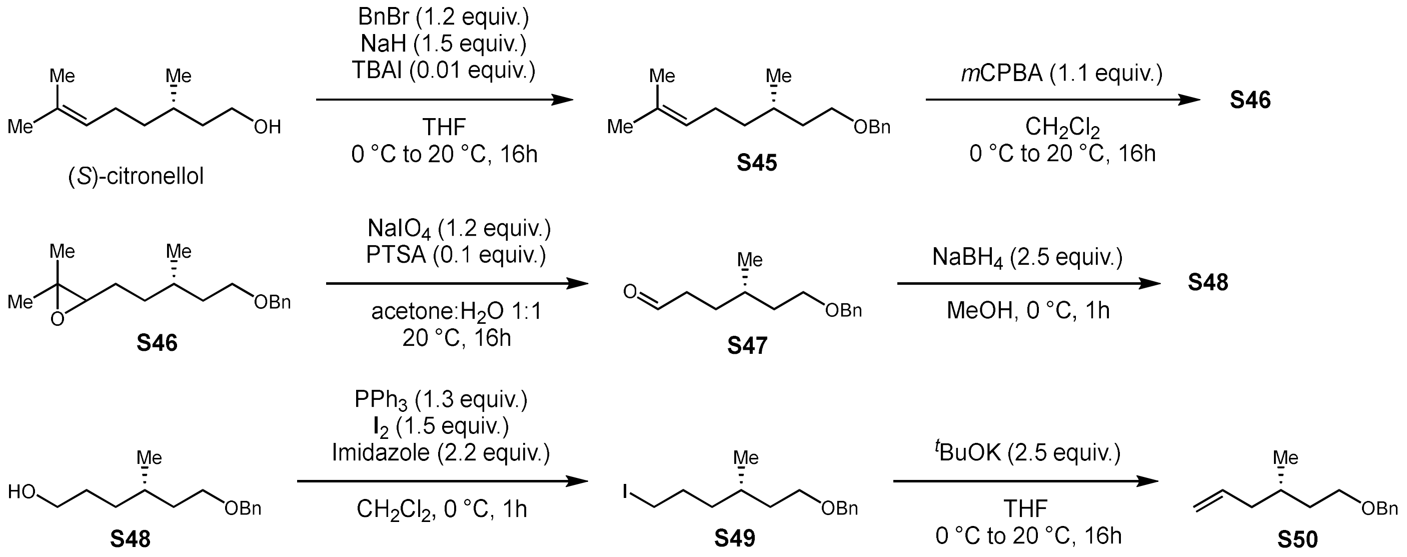

(S)-(((3,7-dimethyloct-6-en-1-yl)oxy)methyl)benzene (**S45**)[22]:

To a 250 mL round bottom flask equipped with a stir bar NaH (60% wt, 2.1 g, 53 mmol, 2.1 equiv.) was added and the atmosphere exchanged with N2 3x followed by the addition of THF (100 mL). The reaction was placed in an ice bath and (*S*)-citronellol (4.6 mL, 25 mmol, 1.0 equiv.) was slowly added. The reaction was stirred in the ice bath for one hour after which BnBr (3.4 mL, 29 mmol, 1.1 equiv.) and TBAI (105 mg, 0.28 mmol, 0.01 euqiv.) were added. The reaction was warmed to room temperature and stirred overnight. After which, the reaction was again placed in an ice bath and quenched with sat. NH_4Cl. The reaction was extracted with EtOAc and washed with DI H2O and sat. brine. The combined organic layers were dried over anhydrous Na_2SO_4 and solvent removed under reduced pressure to obtain **S45** which was used crude in the next reaction.

3-((S)-5-(benzyloxy)-3-methylpentyl)-2,2-dimethyloxirane (**S46**)[23]:

A 100 mL round bottom flask equipped with a stir bar and containing solution of **S45** (4.89 g, 19.8 mmol, 1.0 equiv.) in CH_2Cl_2 (60 mL) was placed in an ice bath. Once cooled, mCPBA (77 wt%, 4.96 g, 22 mmol, 1.1 equiv.) was added and the reaction left to stir at room temperature overnight. The reaction was diluted with CH_2Cl_2 and washed with sat. $NaHCO_3$. The organic layer was dried over Na_2SO_4 and the solvent removed to yield **S46** which was used crude in the next reaction.

(S)-6-(benzyloxy)-4-methylhexanal (**S47**)[23]:

To solution of **S46** (5.21 g, 19.8 mmol, 1.0 equiv.) in acetone (60 mL) in a 250mL round bottom flask equipped with a stir bar was sequentially added $NaIO_4$ (5.1 g, 23 mmol, 1.2 equiv.), DI H_2O (60 mL), and PTSA (0.38 g, 2.0 mmol, 0.1 equiv.). The reaction mixture was left to stir open to air at room temperature overnight. After which the reaction was filtered to remove the white solid that had formed washing with hexanes. The filtrate was washed with DI H_2O and the organic layer was dried over anhydrous Na_2SO_4. The solvent removed to obtain **S47** which was used without further purification.

(S)-6-(benzyloxy)-4-methylhexan-1-ol (**S48**)[24]:

A 250 mL round bottom flask equipped with a stir bar and containing a solution of **S47** (4.37 g, 19.8 mmol, 1.0 equiv.) in MeOH (100 mL) was placed in an ice bath. Once cooled, $NaBH_4$ (1.87 g, 49 mmol, 2.5 equiv.) was slowly added,

and the reaction was left to stir in the ice bath for one hour. The solvent was removed under reduced pressure and the remaining residue redissolved in EtOAc before being sequentially washed with DI H_2O and sat. brine. The organic layer was dried over anhydrous Na_2SO_4 and the solvent removed under reduced pressure to yield **S48**which was sufficiently clean to use in the next step without further purification.

(S)-(((6-iodo-3-methylhexyl)oxy)methyl)benzene (**S49**)[24]:

To 100 mL three neck round bottom flask equipped with a stir bar imidazole (1.84 g, 27 mmol, 2.0 equiv.) and PPh_3 (4.51 g, 17 mmol, 1.3 equiv.) were added. The atmosphere was exchanged with N_2 three times and a solution of **S48** (3.0 mL, 13.5 mmol, 1.0 equiv.) in CH_2Cl_2 (60mL) was added. The reaction vessel was placed in an ice bath and I_2 crystals (4.46 g, 17 mmol, 1.3 equiv.) were added in small portions. The reaction was left to stir in the ice bath for one hour before being quenched with sat. $Na_2S_2O_3$ (20mL). The biphasic reaction was extracted with CH_2Cl_2, and the combines organic layers washed with sat. brine. The organic layer was dried over anhydrous $MgSO_4$ and the solvent removed under reduced pressure. Hexanes was added to the crude residue and the resulting solid was filtered off using short silica plug flushing with more 5% EtOAc in hexanes. The solvent was removed from the filtrate to yield **S49** which was immediately used crude in the next step.

(S)-(((3-methylhex-5-en-1-yl)oxy)methyl)benzene (**S50**)[25]:

A 100 mL two neck round bottom flask containing **S49** (4.49 g, 13.5 mmol, 1.0 equiv.)) in THF (50mL) was placed in an ice bath. To the reaction, KO^{t}Bu (3.89 g, 34 mmol, 2.5 equiv.) was slowly added forming a white suspension. The reaction was removed from the ice bath and left to stir at room temperature overnight. The reaction was quenched using sat. NH_4Cl and extracted three times with Et_2O. The combined organic layers were then washed with sat. $Na_2S_2O_3$ and sat. brine. The organic layer was dried over anhydrous $MgSO_4$ the solvent removed under reduced pressure. The crude product was purified by silica gel column chromatography (5% EtOAc in hexanes) to yield **S50** as a colorless oil (2.715 g, 54% yield over 6 steps). **^{1}H NMR** (400 MHz, $CDCl_3$) δ 7.39 – 7.32 (m, 4H), 7.32 – 7.26 (m, 1H), 5.78 (ddt, *J* = 16.2, 11.2, 7.1 Hz, 1H), 5.01 (ddt, *J* = 5.1, 2.3, 1.3 Hz, 1H), 4.98 (t, *J* = 1.3 Hz, 1H), 4.51 (d, *J* = 0.8 Hz, 2H), 3.51 (ddd, *J* = 9.3, 6.7, 3.0 Hz, 2H), 2.09 (dddt, *J* = 13.8, 6.8, 5.4, 1.3 Hz, 1H), 1.92 (dtt, *J* = 13.8, 7.3, 1.2 Hz, 1H), 1.76 – 1.62 (m, 2H), 1.50 – 1.36 (m, 1H), 0.90 (d, *J* = 6.5 Hz, 3H). **^{13}C NMR** (101 MHz, $CDCl_3$) δ 138.79, 137.41, 128.49, 127.75, 127.62, 115.97, 73.04, 68.69, 41.57, 36.38, 29.91, 19.56. **HRMS** (+APCI) calculated for $C_{14}H_{21}O$ $[M+H]^+$ 205.15869, found 205.15888. $[\alpha]_D^{22}$ +2.6° (*c* = 3.6, $CHCl_3$)

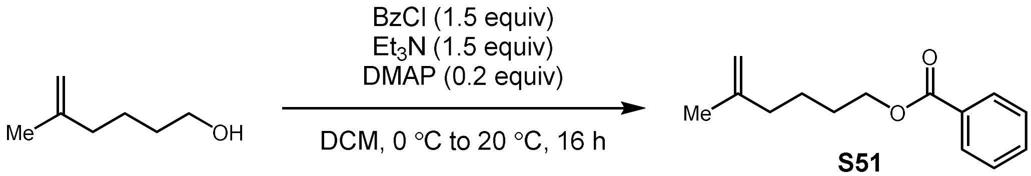

S51

5-methylhex-5-en-1-yl benzoate (**S51**): To a solution of 5-methylhex-5-en-1-ol (0.68 mL, 5.0 mmol, 1.0 equiv)) and DMAP (0.130 g, 1.0 mmol, 0.2 equiv.) in DCM (10 mL) was added Et_3N (1.0 mL, 7.5 mmol, 1.5 equiv.). The reaction was placed in an ice bath and BzCl (0.87 mL, 7.5 mmol, 1.5 equiv.) was added dropwise at 0 °C. The reaction was allowed to warm to room temperature and stirred for 16 hours. The reaction was quenched with the addition of DI H_2O and the layers were separated followed by extraction of the aqueous phase with DCM three times. The combined organic layers were dried over $MgSO_4$ and the solvent was removed under reduced pressure. The crude product was purified using silica gel column chromatography (0-2% EtOAc in Hexanes) to obtain **S51** as a colorless oil (1.062 g, 97%). **^{1}H NMR** (400 MHz, $CDCl_3$) δ 8.09 – 8.00 (m, 2H), 7.60 – 7.52 (m, 1H), 7.48 – 7.39 (m, 2H), 4.76 – 4.71 (m, 1H), 4.71 – 4.66 (m, 1H), 4.33 (t, J = 6.6 Hz, 2H), 2.09 (t, J = 7.5 Hz, 2H), 1.83 – 1.74 (m, 2H), 1.73 (s, 3H), 1.66 – 1.54 (m, 2H).**^{13}C NMR** (101 MHz, $CDCl_3$) δ 166.82, 145.56, 132.97, 130.59, 129.67, 128.47, 110.37, 65.07, 37.45, 28.43, 24.06, 22.44. **HRMS** (+APCI) calculated for $C_{14}H_{19}O_2$ $[M+H]^+$ 219.13796, found 219.13805.

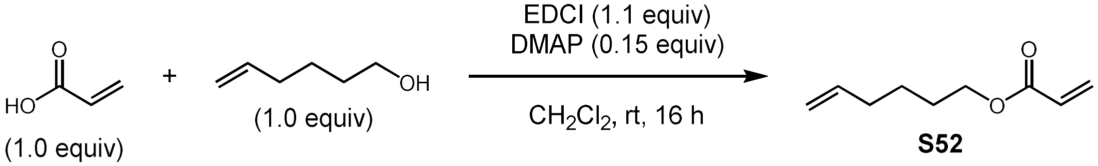

hex-5-en-1-yl acrylate (**S52**): In a 24 mL reaction vial equipped with a stir bar under an N_2 atmosphere, hex-5-en-1-ol (0.60 mL, 5.0 mmol, 1.0 equiv.) was added to a solution of acrylic acid (0.35 mL, 5.1 mmol, 1.0 equiv.) and DMAP (0.08 g, 0.7 mmol, 0.12 equiv.) in CH_2Cl_2 (15 mL). After stirring for five minutes EDCI (1.17 g, 6.0 mmol, 1.2 equiv.) was added and the reaction stirred at room temperature overnight until complete consumption of the starting material was observed via TLC. The reaction was diluted with CH_2Cl_2 and washed with 1M HCl, sat. $NaHCO_3$, DI H_2O, and brine. The organic layer was dried over anhydrous Na_2SO_4 and the solvent removed under reduced pressure. The crude product was purified using silica gel column chromatography (10% EtOAc in Hexanes) to obtain **S52** as a colorless oil (0.250 g, 32 % yield) **^{1}H NMR** (400 MHz, $CDCl_3$) δ 6.39 (dd, *J* = 17.4, 1.5 Hz, 1H), 6.11 (dd, *J* = 17.3, 10.4 Hz, 1H), 5.86 – 5.72 (m, 2H), 5.01 (dq, *J* = 17.1, 1.7 Hz, 1H), 4.96 (ddt, *J* = 10.2, 2.2, 1.2 Hz, 1H), 4.15 (t, *J* = 6.6 Hz, 2H), 2.09 (tdt, *J* = 7.8, 6.7, 1.4 Hz, 2H), 1.75 – 1.62 (m, 2H), 1.53 – 1.40 (m, 2H). **^{13}C NMR** (101 MHz, $CDCl_3$) δ 166.44, 138.46, 130.66, 128.70, 114.97, 64.59, 33.41, 28.16, 25.31. **HRMS** (+APCI) calculated for $C_9H_{15}O_2$ $[M+H]^+$ 155.10666, found 155.1069.

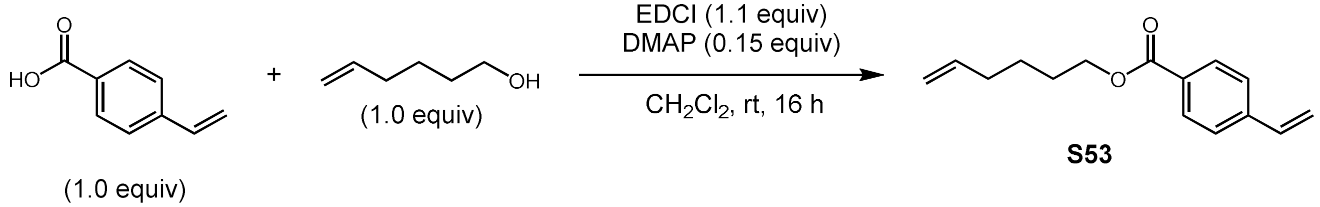

hex-5-en-1-yl 4-vinylbenzoate (**S53**): In a 24 mL reaction vial equipped with a stir bar under an N_2 atmosphere, hex-5-en-1-ol (0.60 mL, 5.0 mmol, 1.0 equiv.) was added to a solution of 4-vinylbenzoic acid (0.76 g, 5.1 mmol, 1.0 equiv.) and DMAP (0.08 g, 0.7 mmol, 0.15 equiv.) in CH_2Cl_2 (15 mL). After stirring for five minutes EDCI (1.10 g, 5.8 mmol, 1.2 equiv.) was added and the reaction stirred at room temperature overnight until complete consumption of the starting material was observed via TLC. The reaction was diluted with CH_2Cl_2 and washed with 1M HCl, sat. $NaHCO_3$, DI H_2O, and brine. The organic layer was dried over anhydrous Na_2SO_4 and the solvent removed under reduced pressure. The crude product was purified using silica gel column chromatography (10% EtOAc in Hexanes) to obtain **S53** as a colorless oil (0.722g, 67 % yield) **^{1}H NMR** (400 MHz, $CDCl_3$) δ 8.00 (d, *J* = 8.4 Hz, 2H), 7.46 (d, *J* = 8.1 Hz, 2H), 6.75 (dd, *J* = 17.6, 10.9 Hz, 1H), 5.91 – 5.75 (m, 2H), 5.38 (dd, *J* = 10.9, 0.7 Hz, 1H), 5.04 (dq, *J* = 17.1, 1.6 Hz, 1H), 4.98 (ddt, *J* = 10.2, 2.2, 1.2 Hz, 1H), 4.32 (t, *J* = 6.6 Hz, 2H), 2.13 (tdt, *J* = 7.8, 6.7, 1.4 Hz, 2H), 1.85 – 1.73 (m, 2H), 1.62 – 1.49 (m, 2H). **^{13}C NMR** (101 MHz, $CDCl_3$) δ 166.56, 141.96, 138.50, 136.16, 129.98, 129.69, 126.21, 116.56, 115.02, 65.00, 33.46, 28.30, 25.44. **HRMS** (+APCI) calculated for $C_{15}H_{19}O_2$ $[M+H]^+$ 231.13796, found 231.13813.

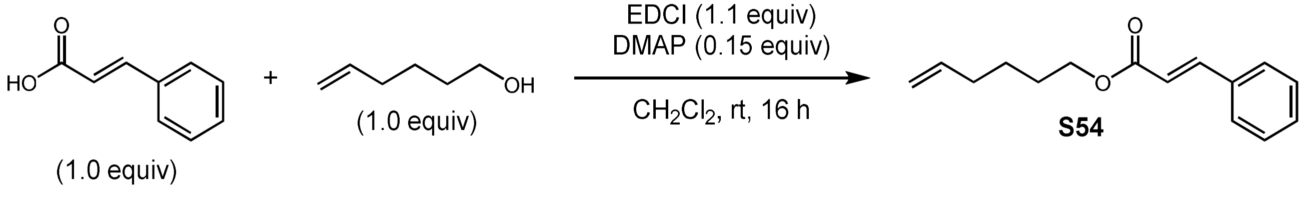

hex-5-en-1-yl cinnamate (**S54**): In a 24 mL reaction vial equipped with a stir bar under an N_2 atmosphere, hex-5-en-1-ol (0.60 mL, 5.0 mmol, 1.0 equiv.) was added to a solution of *trans*-cinnamic acid (0.79 g, 5.3 mmol, 1.1 equiv.) and DMAP (0.07 g, 0.7 mmol, 0.12equiv.) in CH_2Cl_2 (15 mL). After stirring for five minutes EDCI (1.18 g, 6.0 mmol, 1.2 equiv.) was added and the reaction stirred at room temperature overnight until complete consumption of the starting material was observed via TLC. The reaction was diluted with CH_2Cl_2 and washed with 1M HCl, sat. $NaHCO_3$, DI H_2O, and brine. The

organic layer was dried over anhydrous Na_2SO_4 and the solvent removed under reduced pressure. The crude product was purified using silica gel column chromatography (10% EtOAc in Hexanes) to obtain **S54** as a colorless oil (0.766 g, 66 % yield) **^{1}H NMR** (400 MHz, $CDCl_3$) δ 7.57 (d, *J* = 16.0 Hz, 1H), 7.46 – 7.37 (m, 2H), 7.31 – 7.23 (m, 3H), 6.33 (d, *J* = 16.0 Hz, 1H), 5.70 (ddt, *J* = 16.9, 10.2, 6.7 Hz, 1H), 4.92 (dq, *J* = 17.1, 1.7 Hz, 1H), 4.86 (ddt, *J* = 10.2, 2.3, 1.2 Hz, 1H), 4.10 (t, *J* = 6.6 Hz, 2H), 2.00 (tdt, *J* = 7.7, 6.6, 1.4 Hz, 2H), 1.67 – 1.55 (m, 2H), 1.46 – 1.34 (m, 2H). **^{13}C NMR** (101 MHz, $CDCl_3$) δ 167.20, 144.75, 138.50, 134.56, 130.36, 129.00, 128.18, 118.33, 114.98, 64.61, 33.45, 28.28, 25.36. **HRMS** (+APCI) calculated for $C_{15}H_{19}O_2$ $[M+H]^+$ 231.13796, found 231.13814.

4. Enantioselective Aziridinations

General Procedure D: Optimization of Enantioselective Aziridination using 1-nonene (3)

An oven-dried 4dram reaction vial, with Teflon tape wrapped threads, and equipped with an oven dried stir bar was brought into the glovebox. The nitrene source **1** (0.13 mmol, 1.3 equiv.), base, silver salt additive and catalyst (2.5 mol%) were added to the reaction vial. The vial was sealed with a Teflon septum screw cap and brought out of the box to complete the reaction. Under an N_2 atmosphere, HFIP (1.0 mL, 0.1M) was added directly to the reaction vial followed by 1-nonene **3** (17.0 µL ,0.10 mmol, 1.0 equiv.) using a micro syringe. The reaction was left to stir at the indicated temperature under a N_2 balloon for the indicated time. After which, the crude reaction was filtered through a Celite pipette plug using DCM. The solvent was removed under reduced pressure and a crude NMR sample was prepared using dibromomethane as an internal standard. The crude material purified via preparative TLC (5% EtOAc in hexanes) to yield the corresponding aziridine product which was analyzed via chiral HPLC (AD-H column 3% 2-propanol in hexanes, 1.0 mL/min) to determine the enantiomeric ratio.

General Procedure E: Aziridination Procedure for substrates with <u>known</u> densities.

An oven-dried 4dram reaction vial, with Teflon tape wrapped threads, and equipped with an oven dried stir bar was brought into the glovebox. The nitrene source **1** (0.13 mmol, 1.3 equiv.), CsOAc (0.01mmol, 0.1 equiv.), $AgSbF_6$ (0.30 mmol, 0.3 equiv.), and **2** (2.5 mol%) were added to the reaction vial. The vial was sealed with a Teflon septum screw cap and brought out of the box to complete the reaction. Under an N_2 atmosphere, HFIP (1.0 mL, 0.1M) was added directly to the reaction vial followed by the olefin (0.10 mmol, 1.0 equiv.) using a micro syringe. The reaction was left to stir at room temperature under a N_2 balloon for 24 hours. After 24 hours the crude reaction was filtered through a Celite pipette plug using DCM to flush. The solvent was removed under reduced pressure and the crude material purified via preparative TLC using the indicated eluent to yield the corresponding aziridine product.

General Procedure F: Aziridination Procedure for substrates with <u>unknown</u> densities.

An oven-dried 4dram reaction vial, with Teflon tape wrapped threads, and equipped with an oven dried stir bar was brought into the glovebox. The nitrene source **1** (0.13 mmol, 1.3 equiv.), CsOAc (0.01mmol, 0.1 equiv.), $AgSbF_6$ (0.30 mmol, 0.3 equiv.), and **2** (2.5 mol%) were added to the reaction vial. The vial was sealed with a Teflon septum screw cap and brought out of the box to complete the reaction. The olefin (0.10 mmol 1.0 equiv.) was added as a stock solution in HFIP (1.0 mL, 0.1M) and the reaction was left to stir at room temperature under a N_2 balloon for 24 hours. After 24 hours the crude reaction was filtered through a Celite pipette plug using DCM to flush. The solvent was removed under reduced pressure and the crude material purified via preparative TLC using the indicated eluent to yield the corresponding aziridine product.

All racemic reactions were conducted using $[Ind^*RhCl_2]_2$ as the catalyst using either **General Procedure E** or **F**.

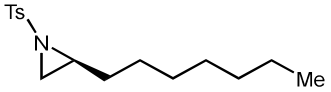

(S)-2-heptyl-1-tosylaziridine (**4**): Prepared using **General Procedure D** using 1-nonene (**3**) and (***R***,***R***)-**2**. Purified via preparative TLC using (5% EtOAc in Hexanes) to provide **4** (0.0245 g, 83%yield, 95:5 e.r.) as colorless oil. **^{1}H NMR** (600 MHz, $CDCl_3$) δ 7.82 (d, *J* = 8.2 Hz, 2H), 7.33 (d, *J* = 8.2 Hz, 2H), 2.71 (tt, *J* = 7.5, 4.8 Hz, 1H), 2.64 (d, *J* = 7.0 Hz, 1H), 2.44 (s, 3H), 2.05 (d, *J* = 4.6 Hz, 1H), 1.53 (ddd, *J* = 9.6, 6.6, 3.4 Hz, 1H), 1.36 – 1.09 (m, 12H), 0.86 (t, *J* = 7.3 Hz, 3H). **^{13}C NMR** (151 MHz, $CDCl_3$) δ 144.53, 135.36, 129.74, 128.13, 40.64, 33.91, 31.76, 29.20, 29.11, 26.89, 22.74, 21.75, 14.20. **HRMS** (+APCI) calculated for $C_{16}H_{26}O_2NS$ $[M+H]^+$ 296.16788, found 296.16864. **HPLC** (AD-H column 3% 2-propanol in hexanes, 1.0 mL/min) t_M = 11.9 min t_m = 10.5 min, 95:5 e.r.

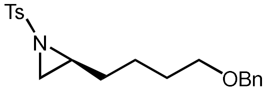

(S)-2-(4-(benzyloxy)butyl)-1-tosylaziridine (**10**): Prepared using **General Procedure F** using **S16** and (***R***,***R***)-**2**. Purified via preparative TLC using (20% EtOAc in Hexanes) to provide **10** (0.0218 g, 61% yield, 95:5 e.r.) as colorless oil. **^{1}H NMR** (600 MHz, $CDCl_3$) δ 7.82 (d, *J* = 8.3 Hz, 2H), 7.38 – 7.27 (m, 7H), 4.46 (s, 2H), 3.38 (t, *J* = 6.4 Hz, 2H), 2.73 (tt, *J* = 7.0, 4.8 Hz, 1H), 2.62 (d, *J* = 6.9 Hz, 1H), 2.42 (s, 3H), 2.05 (d, *J* = 4.6 Hz, 1H), 1.65 – 1.48 (m, 4H), 1.43 – 1.31 (m, 3H). **^{13}C NMR** (151 MHz, $CDCl_3$) δ 144.55, 138.64, 135.29, 129.76, 128.51, 128.11, 127.73, 73.04, 70.05, 40.34, 33.92, 31.21, 29.26, 23.64, 21.75. **HRMS** (+APCI) calculated for $C_{20}H_{26}O_3N^{32}S$ $[M+H]^+$ 360.16279, found 360.16254. **HPLC** (AD-H column 5% 2-propanol in hexanes, 1.0 mL/min) t_M = 22.2 min t_m = 18.7 min, 95:5 e.r.

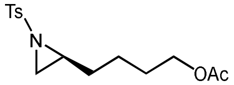

(S)-4-(1-tosylaziridin-2-yl)butyl acetate (**11**): Prepared using **General Procedure F** using **S15** and (***R***,***R***)-**2**. Purified via preparative TLC using (20% EtOAc in Hexanes) to provide **11** (0.0240 g, 77% yield, 95:5 e.r.) as colorless oil. **^{1}H NMR** (400 MHz, $CDCl_3$) δ 7.82 (d, *J* = 8.3 Hz, 2H), 7.38 – 7.30 (m, 2H), 3.95 (t, *J* = 6.6 Hz, 2H), 2.73 (tt, *J* = 7.1, 4.5 Hz, 1H), 2.63 (d, *J* = 7.0 Hz, 1H), 2.44 (s, 3H), 2.06 (d, *J* = 4.6 Hz, 1H), 2.03 (s, 3H), 1.68 – 1.48 (m, 3H), 1.38 – 1.27 (m, 3H). **^{13}C NMR** (101 MHz, $CDCl_3$) δ 171.24, 144.69, 135.15, 129.78, 128.11, 64.17, 40.10, 33.92, 30.98, 28.05, 23.40, 21.77, 21.10. **HRMS** (+APCI) calculated for $C_{15}H_{22}O_4NS$ $[M+H]^+$ 312.12641, found 312.12625. **HPLC** (AD-H column 8% 2-propanol in hexanes, 1.0 mL/min) t_M = 15.1 min t_m = 13.4 min, 95:5 e.r.

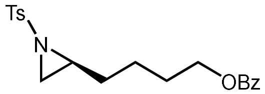

(S)-4-(1-tosylaziridin-2-yl)butyl benzoate (**12**): Prepared using **General Procedure F** using **S16** and (***R***,***R***)-**2**. Purified via preparative TLC using (7:2:1, Hex:EtOAc:Et_3N) to provide **12** (0.0225 g, 60% yield, 95:5 e.r.) as colorless oil. **^{1}H NMR** (400 MHz, $CDCl_3$) δ 8.02 (d, *J* = 7.0 Hz, 2H), 7.82 (d, *J* = 8.3 Hz, 2H), 7.57 (t, *J* = 7.4 Hz, 1H), 7.45 (t, *J* = 7.6 Hz, 2H), 7.30 (d, *J* = 7.6 Hz, 2H), 4.20 (td, *J* = 6.5, 1.4 Hz, 2H), 2.75 (tt, *J* = 7.1, 4.5 Hz, 1H), 2.66 (d, *J* = 6.9 Hz, 1H), 2.40 (s, 3H), 2.09 (d, *J* = 4.5 Hz, 1H), 1.78 – 1.58 (m, 3H), 1.47 – 1.31 (m, 3H). **^{13}C NMR** (101 MHz, $CDCl_3$) δ 166.69, 144.73, 135.15, 133.08, 130.41, 129.79, 129.66, 128.51, 128.12, 64.66, 40.20, 33.92, 31.03, 28.19, 23.57, 21.74. **HRMS** (+APCI) calculated for $C_{20}H_{24}O_4NS$ $[M+H]^+$ 374.14206, found 374.1431. **HPLC** (IA column 10% 2-propanol in hexanes, 1.0 mL/min) t_M = 17.5 min t_m = 15.6 min, 95:5 e.r.

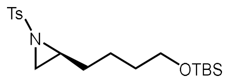

(S)-2-(4-((tert-butyldimethylsilyl)oxy)butyl)-1-tosylaziridine (**13**): Prepared using **General Procedure F** using **S17** and (***R***,***R***)-**2**. Purified via preparative TLC using (20% EtOAc in Pentane) to provide **13** (0.0181 g, 47% yield, 95:5 e.r.) as colorless oil. **^{1}H NMR** (600 MHz, $CDCl_3$) δ 7.82 (d, *J* = 8.3 Hz, 2H), 7.33 (dd, *J* = 8.6, 0.7 Hz, 2H), 3.51 (t, *J* = 6.4 Hz, 2H), 2.77 – 2.67 (m, 1H), 2.63 (d, *J* = 7.0 Hz, 1H), 2.44 (s, 3H), 2.06 (d, *J* = 4.6 Hz, 1H), 1.61 – 1.52 (m, 1H), 1.49 – 1.42 (m, 2H), 1.41 – 1.34 (m, 1H), 1.34 – 1.27 (m, 2H), 0.88 (s, 10H), 0.02 (s, 6H). **^{13}C NMR** (151 MHz, $CDCl_3$) δ 144.53, 135.36, 129.77, 128.12, 62.86, 40.47, 33.91, 32.27, 31.22, 26.09, 23.23, 21.77, 18.47, -5.18. **HRMS** (+APCI) calculated for $C_{19}H_{34}O_3NSSi$ $[M+H]^+$ 384.20232, found 384.2021. **HPLC** (OJ-H column 5% 2-propanol in hexanes, 1.0 mL/min) t_M = 6.0 min t_m = 8.8 min, 95:5 e.r.

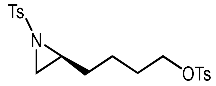

(S)-4-(1-tosylaziridin-2-yl)butyl 4-methylbenzenesulfonate (**14**): Prepared using **General Procedure F** using **S18** and (***R***,***R***)-**2**. Purified via preparative TLC using (60% EtOAc in Hexanes) to provide **14** (0.0087 g, 21% yield, 95:5 e.r.) as colorless oil. **^{1}H NMR** (400 MHz, $CDCl_3$) δ 7.80 (d, *J* = 8.3 Hz, 2H), 7.77 (d, *J* = 8.3 Hz, 2H), 7.38 – 7.30 (m, 4H), 3.92 (t, *J* = 6.3 Hz, 2H), 2.67 (tt, *J* = 7.0, 4.5 Hz, 1H), 2.59 (d, *J* = 7.0 Hz, 1H), 2.45 (s, 6H), 2.02 (d, *J* = 4.5 Hz, 1H), 1.66 – 1.52 (m, 4H), 1.37 – 1.20 (m, 4H). **^{13}C NMR** (101 MHz, $CDCl_3$) δ 144.94, 144.82, 135.08, 133.18, 130.02, 129.86, 128.11, 128.00, 70.16, 39.81, 33.85, 30.67, 28.25, 22.95, 21.79. **HRMS** (+APCI) calculated for $C_{20}H_{26}O_5NS_2$ $[M+H]^+$ 424.12469, found 424.12426. **HPLC** (IA column 30% 2-propanol in hexanes, 1.0 mL/min) t_M = 14.5 min t_m = 13.5x min, 95:5 e.r

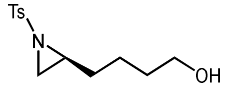

(S)-4-(1-tosylaziridin-2-yl)butan-1-ol (**15**): Prepared using **General Procedure E** using hex-5-en-1-ol and (***R***,***R***)-**2**. Purified via preparative TLC using (60% EtOAc in Hexanes) to provide **15** (0.0132 g, 49% yield, 95:5 e.r.) as colorless oil. **^{1}H NMR** (400 MHz, $CDCl_3$) δ 7.82 (d, *J* = 8.3 Hz, 2H), 7.34 (dd, *J* = 8.6, 0.8 Hz, 2H), 3.56 (td, *J* = 6.4, 5.0 Hz, 2H), 2.75 (tt, *J* = 7.0, 4.6 Hz, 1H), 2.62 (d, *J* = 7.0 Hz, 1H), 2.44 (s, 3H), 2.06 (d, *J* = 4.6 Hz, 1H), 1.68 – 1.57 (m, 1H), 1.57 – 1.47 (m, 2H), 1.39 – 1.33 (m, 3H).**^{13}C NMR** (101 MHz, $CDCl_3$) δ 144.68, 135.16, 129.80, 128.14, 62.66, 40.23, 34.04, 32.05, 31.09, 23.19, 21.78.**HRMS** (+APCI) calculated for $C_{13}H_{20}O_3NS$ $[M+H]^+$ 270.11584, found 270.11587. (IK column 30% 2-propanol in hexanes, 1.0 mL/min) t_M = 16.5 min t_m = 19.3 min, 95:5 e.r.

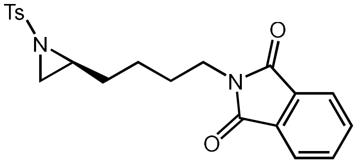

(S)-2-(4-(1-tosylaziridin-2-yl)butyl)isoindoline-1,3-dione (**16**): Prepared using **General Procedure F** using **S20** and (***R***,***R***)-**2**. Purified via preparative TLC using (20% EtOAc in Hexanes) to provide **16** (0.0209 g, 52% yield, 94:6 e.r.) as colorless oil. **^{1}H NMR** (400 MHz, CDCl3) δ 7.87 – 7.78 (m, 4H), 7.71 (dd, J = 5.5, 3.0 Hz, 2H), 7.34 (s, 2H), 3.63 – 3.54 (m, 2H), 2.71 (tt, J = 7.2, 4.7 Hz, 1H), 2.63 (d, J = 6.9 Hz, 1H), 2.44 (s, 3H), 2.07 (d, J = 4.5 Hz, 1H), 1.69 – 1.54 (m, 3H), 1.44 – 1.18 (m, 4H). **^{13}C NMR** (101 MHz, CDCl3) δ 168.49, 144.65, 135.17, 134.08, 132.20, 129.82, 128.11, 123.34, 40.15, 37.60, 33.83, 30.82, 28.05, 24.06, 21.79. **HRMS** (+APCI) calculated for $C_{21}H_{23}O_4N_2S$ $[M+H]^+$ 399.1373, found 399.13749 **HPLC** (IA column 35% 2-propanol in hexanes, 1.0 mL/min) t_M = 15.0 min t_m = 12.6 min, 94:6 e.r.

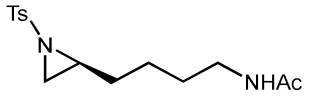

(S)-N-(4-(1-tosylaziridin-2-yl)butyl)acetamide (**17**): Prepared using **General Procedure F** using **S21** and (***R***,***R***)-**2**. Purified via preparative TLC using (100% EtOAc) to provide **17** (0.0221 g, 71%yield, 94:6 e.r.) as colorless oil. **^{1}H NMR** (400 MHz, $CDCl_3$) δ 7.80 (d, *J* = 8.4 Hz, 2H), 7.34 (d, *J* = 8.1 Hz, 2H), 5.70 (s, 1H), 3.17 (hept, *J* = 6.6 Hz, 2H), 2.81 – 2.69 (m, 1H), 2.57 (d, *J* = 7.0 Hz, 1H), 2.44 (s, 3H), 2.05 (d, *J* = 4.6 Hz, 1H), 1.96 (d, *J* = 0.9 Hz, 3H), 1.73 – 1.61 (m, 1H), 1.56 – 1.45 (m, 2H), 1.43 – 1.20 (m, 4H).. **^{13}C NMR** (101 MHz, $CDCl_3$) δ 170.35, 144.77, 135.03, 129.84, 128.09, 39.87, 39.40, 34.33, 30.75, 28.62, 24.31, 23.39, 21.78. **HRMS** (+APCI) calculated for $C_{15}H_{23}O_3N_2S$ $[M+H]^+$ 311.14239, found 311.14236. **HPLC** (IH column 30% acetonitrile in H_2O, 0.75 mL/min) t_M = 20.5 min t_m = 18.0 min, 94:6 e.r.

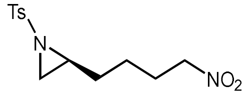

(S)-2-(4-nitrobutyl)-1-tosylaziridine (**18**): Prepared using **General Procedure F** using **S22** and (***R***,***R***)-**2**. Purified via preparative TLC using (40% EtOAc in Hexanes) to provide **18** (0.0261 g, 88% yield, 94:6 e.r.) as colorless oil. **^{1}H NMR** (400 MHz, CDCl3) δ 7.81 (d, J = 8.3 Hz, 2H), 7.35 (d, J = 7.8 Hz, 2H), 4.28 (td, J = 6.9, 2.3 Hz, 2H), 2.71 (ddt, J = 8.4, 7.1, 4.3 Hz, 1H), 2.64 (d, J = 7.0 Hz, 1H), 2.46 (s, 3H), 2.07 (d, J = 4.4 Hz, 1H), 2.01 – 1.89 (m, 2H), 1.78 – 1.65 (m, 1H), 1.40 – 1.22 (m, 3H). **^{13}C NMR** (101 MHz, CDCl3) δ 144.94, 134.93, 129.87, 128.12, 39.57, 33.86, 30.56, 26.60, 23.80, 21.78. **HRMS** (+APCI) calculated for $C_{13}H_{19}O_4N_2S$ $[M+H]^+$ 299.10600, found 299.10601 **HPLC** (IB column 40% 2-propanol in hexanes, 1.0 mL/min) t_M = 11.8 min t_m = 11.2 min, 94:6 e.r.

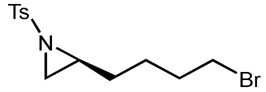

(S)-2-(4-bromobutyl)-1-tosylaziridine (**19**): Prepared using **General Procedure E** using 6-bromohex-1-ene (**S23**) and (***R***,***R***)-**2**. Purified via preparative TLC using (7:2:1, Hex:EtOAc:Et_3N) to provide **19** (0.0196 g, 59%yield, 96:4 e.r.) as colorless oil. **^{1}H NMR** (400 MHz, $CDCl_3$) δ 7.82 (d, *J* = 8.3 Hz, 2H), 7.35 (d, *J* = 7.8 Hz, 2H), 3.29 (td, *J* = 6.7, 1.6 Hz, 2H), 2.71 (tt, *J* = 7.1, 4.5 Hz, 1H), 2.64 (d, *J* = 7.0 Hz, 1H), 2.45 (s, 3H), 2.07 (d, *J* = 4.5 Hz, 1H), 1.85 – 1.70 (m, 2H), 1.69 – 1.57 (m, 1H), 1.46 – 1.21 (m, 3H).**^{13}C NMR** (101 MHz, $CDCl_3$) δ 144.74, 135.14, 129.84, 128.13, 40.04, 33.84, 33.36, 32.01, 30.54, 25.60, 21.79. **HRMS** (+APCI) calculated for $C_{13}H_{19}O_2NBrS$ $[M+H]^+$ 332.03144, found 332.03157. **HPLC** (IH column 20% 2-propanol in hexanes, 1.0 mL/min) t_M = 23.5 min t_m = 21.5 min, 96:4 e.r.

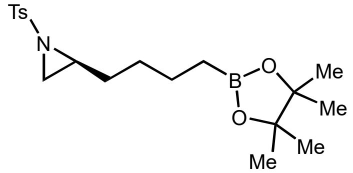

(S)-2-(4-(4,4,5,5-tetramethyl-1,3,2-dioxaborolan-2-yl)butyl)-1-tosylaziridine (**20**): Prepared using **General Procedure F** using **S24** and (***R***,***R***)-**2**. Purified via preparative TLC using (10% Acetone in Pentane) to provide **21** (0.0216 g, 57% yield, 96:4 e.r.) as colorless oil. **^{1}H NMR** (400 MHz, $CDCl_3$) δ 7.81 (d, *J* = 8.3 Hz, 2H), 7.32 (d, *J* = 7.8 Hz, 2H), 2.71 (tt, *J* = 7.1, 4.9 Hz, 1H), 2.62 (d, *J* = 7.0 Hz, 1H), 2.44 (s, 3H), 2.05 (d, *J* = 4.6 Hz, 1H), 1.58 – 1.44 (m, 1H), 1.41 – 1.27 (m, 3H), 1.23 (s, 14H), 0.66 (t, *J* = 7.8 Hz, 2H). **^{13}C NMR** (101 MHz, $CDCl_3$) δ 144.52, 135.37, 129.75, 128.10, 83.08, 40.60, 33.89, 31.18, 29.48, 24.94, 23.62, 21.77. **HRMS** (+APCI) calculated for $C_{19}H_{31}O_4NBS$ $[M+H]^+$ 379.20977, found 379.20968 **HPLC** (IH column 5% 2-propanol in hexanes, 1.0 mL/min) t_M = 18.7 min t_m = 16.8 min, 96:4 e.r.

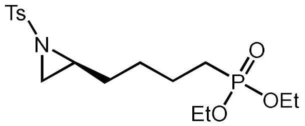

diethyl (S)-(4-(1-tosylaziridin-2-yl)butyl)phosphonate (**21**): Prepared using **General Procedure F** using **S25** and (***R***,***R***)**-2**. Purified via preparative TLC using (80% EtOAc in Hexanes) to provide **21** (0.0259 g, 67%yield, 95:5 e.r.) as colorless oil. **^{1}H NMR** (400 MHz, $CDCl_3$) δ 7.80 (d, J = 8.3 Hz, 2H), 7.33 (d, J = 8.3 Hz, 2H), 4.15 – 3.98 (m, 4H), 2.71 (tt, J = 6.7, 4.9 Hz, 1H), 2.60 (d, J = 6.9 Hz, 1H), 2.43 (s, 3H), 2.04 (d, J = 4.5 Hz, 1H), 1.70 – 1.48 (m, 5H), 1.40 – 1.25 (m, 9H). **^{13}C NMR** (101 MHz, $CDCl_3$) δ 144.62, 135.17, 129.79, 128.09, 61.60, 61.54, 39.95, 33.97, 30.94, 30.92, 27.93, 27.76, 26.32, 24.91, 22.12, 22.07, 21.75, 16.62, 16.56. **^{31}P NMR** (162 MHz, $CDCl_3$) δ 31.80 (tp, J = 17.0, 9.0 Hz). **HRMS** (+APCI) calculated for $C_{17}H_{29}O_5NPS$ [M+H]$^+$ 390.14986, found 390.14991. **HPLC** (IH column 50% acetonitrile in water, 0.75 mL/min) t_M = 10.2 min t_m = 9.3 min, 95:5 e.r.

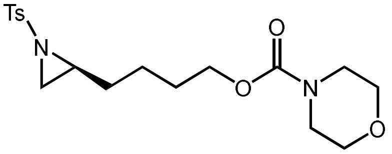

(S)-4-(1-tosylaziridin-2-yl)butyl morpholine-4-carboxylate (**22**): Prepared using **General Procedure F** using **S36** and (***R***,***R***)**-2**. Purified via preparative TLC using (50% EtOAc in Hexanes) to provide **22** (0.0236 g, 62%yield, 94:6 e.r.) as colorless oil. **^{1}H NMR** (400 MHz, $CDCl_3$) δ 7.81 (d, J = 8.3 Hz, 2H), 7.33 (d, J = 8.1 Hz, 2H), 3.99 (t, J = 6.6 Hz, 2H), 3.64 (d, J = 4.7 Hz, 4H), 3.45 (d, J = 4.9 Hz, 4H), 2.74 (tt, J = 6.9, 4.5 Hz, 1H), 2.61 (d, J = 7.0 Hz, 1H), 2.44 (s, 3H), 2.05 (d, J = 4.6 Hz, 1H), 1.68 – 1.53 (m, 3H), 1.41 – 1.29 (m, 3H). **^{13}C NMR** (101 MHz, $CDCl_3$) δ 155.47, 144.60, 135.06, 129.69, 127.99, 66.61, 65.14, 39.91, 33.94, 30.90, 28.35, 23.31, 21.68. **HRMS** (+APCI) calculated for $C_{18}H_{27}O_5N_2S$ [M+H]$^+$ 383.16352, found 383.16338. **HPLC** (IH column 50% 2-propanol in hexanes, 1.0 mL/min) t_M = 17.8 min t_m = 15.4 min, 94:6 e.r.

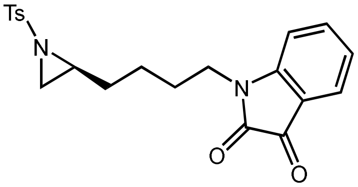

(S)-1-(4-(1-tosylaziridin-2-yl)butyl)indoline-2,3-dione (**23**): Prepared using **General Procedure F** using **S37** and (***R***,***R***)**-2**. Purified via preparative TLC using (50% EtOAc in Hexanes) to provide **23** (0.0310 g, 78 % yield, 93:7 e.r.) as colorless oil. **^{1}H NMR** (400 MHz, $CDCl_3$) δ 7.79 (d, J = 8.3 Hz, 2H), 7.59 (d, J = 7.6 Hz, 2H), 7.33 (d, J = 7.8 Hz, 2H), 7.11 (td, J = 7.6, 0.8 Hz, 1H), 6.90 (dt, J = 7.6, 0.9 Hz, 1H), 3.66 (hept, J = 7.2 Hz, 2H), 2.74 (tt, J = 7.1, 4.5 Hz, 1H), 2.58 (d, J = 6.9 Hz, 1H), 2.44 (s, 3H), 2.05 (d, J = 4.6 Hz, 1H), 1.70 (ddd, J = 13.2, 9.1, 6.1 Hz, 3H), 1.48 – 1.30 (m, 3H).**^{13}C NMR** (101 MHz, $CDCl_3$) δ 183.63, 158.24, 150.94, 144.78, 138.56, 134.95, 129.85, 128.07, 125.58, 123.83, 117.66, 110.34, 39.96, 39.67, 34.08, 30.75, 26.61, 24.26, 21.78.**HRMS** (+ESI) calculated for $C_{21}H_{23}O_4N_2S$ [M+H]$^+$ 399.1373, found 399.13772. **HPLC** (IH column 50% acetonitrile in water, 0.75 mL/min) t_M = 21.2 min t_m = 19.6 min, 93:7 e.r.

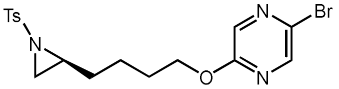

(S)-2-bromo-5-(4-(1-tosylaziridin-2-yl)butoxy)pyrazine (**24**): Prepared using **General Procedure F** using **S38** and (***R***,***R***)**-2**. Purified via preparative TLC using (7:2:1, Hex:EtOAc:Et_3N) to provide **24** (0.0284 g, 67%yield, 95:5 e.r.) as colorless oil. **^{1}H NMR** (400 MHz, $CDCl_3$) δ 8.15 (d, J = 1.4 Hz, 1H), 7.97 (d, J = 1.3 Hz, 1H), 7.82 (d, J = 8.3 Hz, 2H), 7.33 (d, J = 8.0

Hz, 2H), 4.18 (t, J = 6.5 Hz, 2H), 2.76 (tt, J = 7.0, 4.6 Hz, 1H), 2.63 (d, J = 6.9 Hz, 1H), 2.07 (d, J = 4.6 Hz, 1H), 1.80 – 1.59 (m, 3H), 1.51 – 1.30 (m, 3H).**^{13}C NMR** (101 MHz, $CDCl_3$) δ 159.66, 144.67, 142.92, 135.50, 135.16, 130.08, 129.78, 128.12, 66.80, 40.04, 33.98, 31.02, 28.13, 23.43, 21.76. **HRMS** (+APCI) calculated for $C_{17}H_{21}O_3N_3BrS$ $[M+H]^+$ 426.04815, found 426.04952. **HPLC** (IA column 10% 2-propanol in hexanes, 1.0 mL/min) t_M = 19.8 min t_m = 18.5 min, 95:5 e.r.

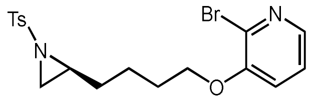

(S)-2-bromo-3-(4-(1-tosylaziridin-2-yl)butoxy)pyridine (**25**): Prepared using **General Procedure F** using **S39** and (***R***,***R***)-**2**. Purified via preparative TLC using (50% EtOAc in Hexanes) to provide **25** (0.0120 g, 28 % yield, 96:4 e.r.) as colorless oil. **^{1}H NMR** (400 MHz, $CDCl_3$) δ 8.00 (dd, J = 4.6, 1.5 Hz, 1H), 7.85 (d, J = 8.3 Hz, 2H), 7.35 (d, J = 7.7 Hz, 2H), 7.22 (dd, J = 8.1, 4.6 Hz, 1H), 7.10 (dd, J = 8.1, 1.6 Hz, 1H), 3.96 (td, J = 6.2, 2.0 Hz, 2H), 2.81 (ddt, J = 7.7, 7.0, 4.6 Hz, 1H), 2.66 (d, J = 7.0 Hz, 1H), 2.41 (s, 3H), 2.12 (d, J = 4.6 Hz, 1H), 1.84 (ddd, J = 13.8, 7.3, 6.1 Hz, 2H), 1.79 – 1.68 (m, 1H), 1.62 – 1.36 (m, 3H).**^{13}C NMR** (101 MHz, $CDCl_3$) δ 152.45, 144.66, 141.32, 135.12, 133.20, 129.83, 128.14, 123.52, 119.55, 68.99, 40.10, 34.01, 30.96, 28.28, 23.60, 21.74.**HRMS** (+ESI) calculated for $C_{18}H_{22}O_3N_2BrS$ $[M+H]^+$ 425.0529, found 425.05288. **HPLC** (IJ column 50% 2-propanol in hexanes, 1.5 mL/min) t_M = 19.6 min t_m = 17.3 min, 96:4 e.r.

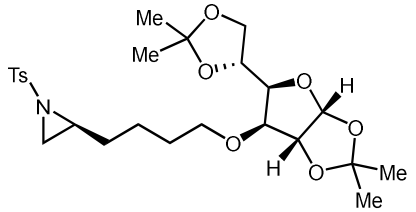

(S)-2-(4-(((3aR,5R,6S,6aR)-5-((R)-2,2-dimethyl-1,3-dioxolan-4-yl)-2,2-dimethyltetrahydrofuro[2,3-d][1,3]dioxol-6-yl)oxy)butyl)-1-tosylaziridine (**26**): Prepared using **General Procedure F** using **S26** and (***R***,***R***)-**2**. Purified via preparative TLC using (20% EtOAc in Hexanes) to provide **26** (0.0355 g, 69%yield, 95:5 d.r.) as colorless oil. **^{1}H NMR** (400 MHz, $CDCl_3$) δ 7.81 (d, J = 8.3 Hz, 2H), 7.33 (d, J = 8.2 Hz, 2H), 5.85 (d, J = 3.7 Hz, 1H), 4.49 (d, J = 3.8 Hz, 1H), 4.26 (dt, J = 7.7, 5.9 Hz, 1H), 4.13 – 4.01 (m, 2H), 3.97 (dd, J = 8.6, 5.7 Hz, 1H), 3.80 (d, J = 3.0 Hz, 1H), 3.52 (dt, J = 9.1, 6.3 Hz, 1H), 3.44 (dt, J = 9.3, 6.3 Hz, 1H), 2.74 (tt, J = 6.4, 4.6 Hz, 1H), 2.59 (d, J = 7.0 Hz, 1H), 2.44 (s, 3H), 2.04 (d, J = 4.6 Hz, 1H), 1.62 – 1.50 (m, 3H), 1.48 (s, 3H), 1.41 (s, 3H), 1.39 – 1.33 (m, 3H), 1.32 (s, 3H), 1.31 (s, 3H). **^{13}C NMR** (101 MHz, $CDCl_3$) δ 144.59, 135.27, 129.77, 128.10, 111.87, 109.05, 105.36, 82.63, 82.25, 81.26, 72.58, 70.24, 67.38, 40.11, 34.00, 31.07, 29.16, 26.96, 26.94, 26.37, 25.55, 23.46, 21.76. **HRMS** (+APCI) calculated for $C_{25}H_{38}O_8NS$ $[M+H]^+$ 512.23126, found 512.23097 **HPLC** (OJ-H column 20% 2-propanol in hexanes, 1.0 mL/min) t_M = 12.3 min t_m = 17.7 min, 95:5 d.r.

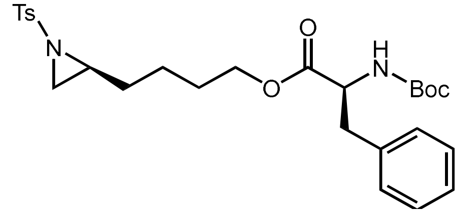

4-((S)-1-tosylaziridin-2-yl)butyl (tert-butoxycarbonyl)-L-phenylalaninate (**27**): Prepared using **General Procedure F** using **S40** and (***R***,***R***)-**2**. Purified via preparative TLC using (30% EtOAc in Hexanes) to provide **27** (0.0423 g, 82% yield, 94:6 d.r.) as a yellow oil. **^{1}H NMR** (400 MHz, $CDCl_3$) δ 7.81 (d, J = 8.3 Hz, 2H), 7.33 (d, J = 8.0 Hz, 2H), 7.32 – 7.19 (m, 3H), 7.12 (d, J = 6.5 Hz, 2H), 4.99 (d, J = 8.3 Hz, 1H), 4.54 (dt, J = 8.4, 6.2 Hz, 1H), 3.97 (t, J = 6.6 Hz, 2H), 3.05 (ddd, J = 20.9, 14.0, 6.4 Hz, 2H), 2.71 (tt, J = 7.2, 4.8 Hz, 1H), 2.62 (d, J = 7.0 Hz, 1H), 2.43 (s, 3H), 2.05 (d, J = 4.5 Hz, 1H), 1.64 – 1.46 (m, 3H), 1.41 (s, 9H), 1.36 – 1.19 (m, 3H). **^{13}C NMR** (101 MHz, $CDCl_3$) δ 172.03, 155.16, 144.70, 136.14, 135.12, 129.79, 129.40, 128.62, 128.09, 127.10, 79.99, 64.99, 54.56, 39.91, 38.56, 33.90, 30.86, 28.40, 27.86, 23.24, 21.74. **HRMS**

(+ESI) calculated for $C_{27}H_{36}O_6N_2NaS$ $[M+Na]^+$ 539.21863, found 539.21969. **HPLC** (IH column 60% acetonitrile in water, 0.75 mL/min) t_M = 16.6 min t_m = 15.5 min, 94:6 d.r.

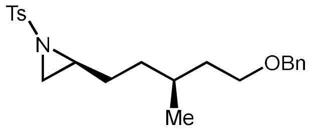

(S)-2-((S)-5-(benzyloxy)-3-methylpentyl)-1-tosylaziridine **(*S,S*)-28**: Prepared using **General Procedure F** using **S44** and **(*R*,*R*)-2**. Purified via preparative TLC using (7:2:1, Hex:EtOAc:Et$_3$N) to provide **(*S,S*)-28** (0.0300 g, 77% yield, 97:3 d.r.) as colorless oil. **^{1}H NMR** (400 MHz, CDCl$_3$) δ 7.82 (d, J = 8.3 Hz, 2H), 7.39 – 7.27 (m, 7H), 4.48 (s, 2H), 3.44 (tq, J = 6.1, 2.8 Hz, 2H), 2.68 (tt, J = 7.2, 4.6 Hz, 1H), 2.62 (d, J = 7.0 Hz, 1H), 2.43 (s, 3H), 2.05 (d, J = 4.5 Hz, 1H), 1.59 – 1.46 (m, 3H), 1.41 – 1.18 (m, 3H), 1.09 – 0.96 (m, 1H), 0.80 (d, J = 6.4 Hz, 3H). **^{13}C NMR** (101 MHz, CDCl$_3$) δ 144.54, 138.70, 135.26, 129.73, 128.48, 128.14, 127.74, 127.65, 73.05, 68.44, 40.68, 36.66, 33.95, 33.94, 29.48, 28.89, 21.73, 19.37. **HRMS** (+APCI) calculated for $C_{22}H_{30}O_3NS$ $[M+H]^+$ 388.19409, found 388.19394. **HPLC** (IH column 15% 2-propanol in hexanes, 1.0 mL/min) t_M = 17.0min t_m = 19.8 min, 97:3 d.r.

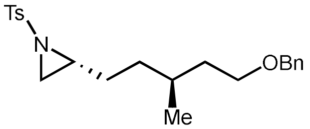

(R)-2-((S)-5-(benzyloxy)-3-methylpentyl)-1-tosylaziridine **(*R,S*)-28**: Prepared using **General Procedure F** using **S44** and **(*S*,*S*)-2**. Purified via preparative TLC using (7:2:1, Hex:EtOAc:Et$_3$N) to provide **(*R,S*)-28** (0.0280 g, 72% yield, 7:93 d.r.) as colorless oil. **^{1}H NMR** (400 MHz, CDCl$_3$) δ 7.82 (d, J = 8.3 Hz, 2H), 7.39 – 7.26 (m, 7H), 4.47 (s, 2H), 3.43 (td, J = 6.8, 6.3, 2.7 Hz, 2H), 2.68 (tt, J = 7.2, 4.7 Hz, 1H), 2.62 (d, J = 7.0 Hz, 1H), 2.44 (s, 3H), 2.05 (d, J = 4.5 Hz, 1H), 1.65 – 1.43 (m, 3H), 1.40 – 1.15 (m, 3H), 1.07 (dddd, J = 13.3, 10.9, 7.4, 5.4 Hz, 1H), 0.80 (d, J = 6.5 Hz, 3H). **^{13}C NMR** (101 MHz, CDCl$_3$) δ 144.54, 138.69, 135.31, 129.74, 128.49, 128.14, 127.73, 127.66, 73.06, 68.46, 40.75, 36.51, 33.99, 33.89, 29.49, 28.90, 21.75, 19.57. **HRMS** (+APCI) calculated for $C_{22}H_{30}O_3NS$ $[M+H]^+$ 388.19409, found 388.19386. **HPLC** (IH column 15% 2-propanol in hexanes, 1.0 mL/min) t_M = 19.7 min t_m = 17.2 min, 7:93 d.r.

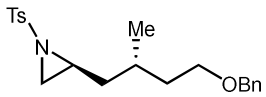

(S)-2-((S)-4-(benzyloxy)-2-methylbutyl)-1-tosylaziridine **(*S,S*)-29**: Prepared using **General Procedure F** using **S50** and **(*R*,*R*)-2**. Purified via preparative TLC using (7:2:1, Hex:EtOAc:Et$_3$N) to provide **(*S,S*)-29** (0.0090 g, 24% yield, 9:91 d.r.) as colorless oil. **^{1}H NMR** (400 MHz, CDCl$_3$) δ 7.82 (d, J = 8.3 Hz, 2H), 7.38 – 7.26 (m, 7H), 4.46 (s, 2H), 3.43 (td, J = 6.6, 2.6 Hz, 2H), 2.79 (tdd, J = 7.1, 5.8, 4.6 Hz, 1H), 2.64 (d, J = 6.9 Hz, 1H), 2.42 (s, 3H), 2.04 (d, J = 4.6 Hz, 1H), 1.73 – 1.53 (m, 3H), 1.51 – 1.37 (m, 2H), 1.35 – 1.22 (m, 2H), 0.89 (d, J = 6.6 Hz, 3H). **^{13}C NMR** (101 MHz, CDCl$_3$) δ 144.56, 138.66, 135.32, 129.80, 129.77, 128.52, 128.10, 127.72, 127.68, 73.07, 68.31, 38.85, 38.81, 36.84, 34.40, 28.84, 21.77, 19.31. **HRMS** (+APCI) calculated for $C_{21}H_{28}O_3N^{32}S$ $[M+H]^+$ 374.17844, found 374.1784. **HPLC** (IK column 15% 2-propanol in hexanes, 1.0 mL/min) t_M = 22.3 min t_m = 19.9 min, 9:91 d.r.

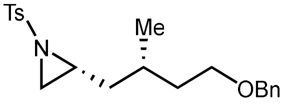

(R)-2-((S)-4-(benzyloxy)-2-methylbutyl)-1-tosylaziridine (***R,S***)**-29**: Prepared using **General Procedure F** using **S50** and (***S,S***)**-2**. Purified via preparative TLC using (7:2:1, Hex:EtOAc:Et_3N) to provide (***R,S***)**-29** (0.0110 g, 30%yield, 97:3 d.r.) as colorless oil. **^{1}H NMR** (400 MHz, $CDCl_3$) δ 7.82 (d, J = 8.3 Hz, 2H), 7.38 – 7.27 (m, 7H), 4.47 (s, 2H), 3.51 – 3.33 (m, 2H), 2.81 (tdd, J = 6.9, 6.0, 4.6 Hz, 1H), 2.60 (d, J = 7.0 Hz, 1H), 2.43 (s, 3H), 1.99 (d, J = 4.6 Hz, 1H), 1.75 – 1.60 (m, 2H), 1.51 – 1.31 (m, 3H), 0.90 (d, J = 6.6 Hz, 3H). **^{13}C NMR** (101 MHz, $CDCl_3$) δ 144.55, 138.65, 135.35, 129.80, 128.53, 128.10, 127.76, 127.70, 73.11, 68.27, 38.95, 38.77, 36.13, 33.88, 28.91, 21.78, 19.74. **HRMS** (+APCI) calculated for $C_{21}H_{28}O_3NS$ $[M+H]^+$ 374.17844, found 374.17833. **HPLC** (IK column 15% 2-propanol in hexanes, 1.0 mL/min) t_M = 19.9 min t_m = 22.4 min, 97:3 d.r.

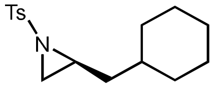

(S)-2-(cyclohexylmethyl)-1-tosylaziridine (**30**): Prepared using **General Procedure F** using allylcyclohexane (**S27**) and (***R***,***R***)**-2**. Purified via preparative TLC using (20% EtOAc in Hexanes) to provide **30** (0.0247 g, 83% yield, 96:4 e.r.) as a colorless oil. **^{1}H NMR** (400 MHz, $CDCl_3$) δ 7.83 (d, J = 8.3 Hz, 2H), 7.34 (d, J = 7.7 Hz, 2H), 2.80 – 2.72 (m, 1H), 2.65 (d, J = 7.0 Hz, 1H), 2.44 (s, 3H), 2.03 (d, J = 4.6 Hz, 1H), 1.72 – 1.50 (m, 5H), 1.39 – 1.22 (m, 2H), 1.21 – 0.99 (m, 4H), 0.91 – 0.75 (m, 2H). **^{13}C NMR** (101 MHz, $CDCl_3$) δ 144.57, 135.32, 129.77, 128.14, 39.25, 39.13, 36.20, 34.01, 33.51, 32.70, 26.42, 26.24, 26.10, 21.77. **HRMS** (+APCI) calculated for $C_{16}H_{24}O_2NS$ $[M+H]^+$ 294.15223, found 294.15201. **HPLC** (IK column 10% 2-propanol in hexanes, 1.0 mL/min) t_M = 21.1 min t_m = 19.0 min, 96:4 e.r.

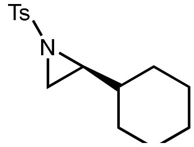

(S)-2-cyclohexyl-1-tosylaziridine (**31**): Prepared using **General Procedure E** using vinylcyclohexane (**S28**) and (***R***,***R***)**-2**. Purified via preparative TLC using (20% EtOAc in Hexanes) to provide **31** (0.0039 g, 14%yield, 91:9 e.r.) as colorless oil. **^{1}H NMR** (400 MHz, $CDCl_3$) δ 7.82 (d, J = 8.3 Hz, 2H), 7.33 (d, J = 7.9 Hz, 2H), 2.60 (d, J = 7.0 Hz, 1H), 2.53 (td, J = 7.2, 4.6 Hz, 1H), 2.45 (s, 3H), 2.10 (d, J = 4.6 Hz, 1H), 1.74 – 1.45 (m, 6H), 1.27 – 0.85 (m, 7H). **^{13}C NMR** (101 MHz, $CDCl_3$) δ 144.53, 135.27, 129.74, 128.23, 45.31, 39.54, 32.81, 30.34, 29.77, 26.16, 25.70, 25.52, 21.81. **HRMS** (+APCI) calculated for $C_{15}H_{22}O_2NS$ $[M+H]^+$ 280.13658, found 280.13654. **HPLC** (AS-H column 5% 2-propanol in hexanes, 1.0 mL/min) t_M = 22.9 min t_m = 18.2 min, 91:9 e.r.

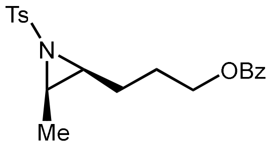

3-((2S,3R)-3-methyl-1-tosylaziridin-2-yl)propyl benzoate (**32**): Prepared using **General Procedure F** using **S29** and (***R***,***R***)-**2**. Purified via preparative TLC using (7:2:1, Hex:EtOAc:Et_3N) to provide **32** (0.0226 g, 61% yield, 86:14 e.r., >20:1 d.r.) as colorless oil. **^{1}H NMR** (400 MHz, $CDCl_3$) δ 8.00 (dd, J = 8.4, 1.4 Hz, 2H), 7.82 (d, J = 8.3 Hz, 2H), 7.61 – 7.51 (m, 1H), 7.49 – 7.40 (m, 2H), 7.32 (dd, J = 8.7, 0.8 Hz, 2H), 4.33 – 4.17 (m, 2H), 2.95 (dq, J = 7.3, 5.9 Hz, 1H), 2.81 (ddd, J = 8.3, 7.3, 4.9 Hz, 1H), 2.40 (s, 3H), 1.77 – 1.61 (m, 3H), 1.56 – 1.45 (m, 1H), 1.22 (d, J = 5.9 Hz, 3H). **^{13}C NMR** (101 MHz, $CDCl_3$) δ 166.55, 144.51, 135.45, 133.12, 130.25, 129.78, 129.63, 128.51, 127.97, 64.16, 44.46, 40.47, 26.59, 23.31, 21.73,

12.11. **HRMS** (+APCI) calculated for $C_{20}H_{24}O_4N^{32}S$ $[M+H]^+$ 374.14206, found 374.14319. **HPLC** (IB column 10% 2-propanol in hexanes, 1.0 mL/min) t_M = 16.9 min t_m = 15.4 min, 86:14 e.r. >20:1 d.r.

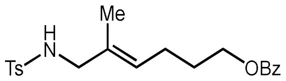

5-methyl-6-((4-methylphenyl)sulfonamido)hex-4-en-1-yl benzoate (**34**): Prepared using **General Procedure F** using **S51** and (***R***,***R***)-**2**. Purified via preparative TLC using (20% EtOAc in Hexanes) to provide **34** (0.0215 g, 56% yield) as a colorless oil. **^{1}H NMR** (400 MHz, $CDCl_3$) δ 8.06 – 7.98 (m, 2H), 7.75 – 7.69 (m, 2H), 7.59 – 7.53 (m, 1H), 7.44 (dd, J = 8.5, 7.0 Hz, 2H), 7.30 (d, J = 8.1 Hz, 2H), 5.35 – 5.20 (m, 1H), 4.44 (s, 1H), 4.26 (t, J = 6.5 Hz, 2H), 3.44 (d, J = 6.4 Hz, 2H), 2.42 (s, 3H), 2.11 (q, J = 7.4 Hz, 2H), 1.81 – 1.71 (m, 2H), 1.56 (s, 3H).**^{13}C NMR** (101 MHz, $CDCl_3$) δ 166.71, 143.50, 137.18, 133.08, 131.60, 130.41, 129.79, 129.63, 128.52, 127.33, 127.25, 64.34, 51.14, 28.38, 24.33, 21.65, 14.44. **HRMS** (+APCI) calculated for $C_{21}H_{26}O_4N^{32}S$ $[M+H]^+$ 388.15771, found 388.15732.

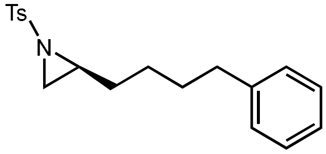

(S)-2-(4-phenylbutyl)-1-tosylaziridine (**35**): Prepared using **General Procedure F** using **S30** and (***R***,***R***)-**2**. Purified via preparative TLC using (20% EtOAc in Hexanes) to provide **35** (0.0215 g, 82% yield, 94:6 e.r.) as colorless oil. **^{1}H NMR** (400 MHz, $CDCl_3$) δ 7.82 (d, *J* = 8.3 Hz, 2H), 7.33 (d, *J* = 7.9 Hz, 2H), 7.30 – 7.23 (m, 2H), 7.22 – 7.15 (m, 1H), 7.12 (d, *J* = 6.7 Hz, 2H), 2.72 (tt, *J* = 7.3, 4.8 Hz, 1H), 2.63 (d, *J* = 7.0 Hz, 1H), 2.52 (t, *J* = 7.7 Hz, 2H), 2.45 (s, 3H), 2.06 (d, *J* = 4.6 Hz, 1H), 1.64 – 1.48 (m, 3H), 1.44 – 1.21 (m, 3H). **^{13}C NMR** (101 MHz, $CDCl_3$) δ 144.55, 142.32, 135.30, 129.77, 128.45, 128.41, 128.11, 125.87, 40.42, 35.79, 33.93, 31.31, 30.92, 26.54, 21.77.**HRMS** (+APCI) calculated for $C_{19}H_{24}O_2NS$ $[M+H]^+$ 330.15223, found 330.15195. **HPLC** (AD-H column 5% 2-propanol in hexanes, 1.0 mL/min) t_M = 16.8 min t_m = 13.2 min, 94:6 e.r.

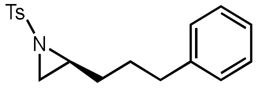

(S)-2-(3-phenylpropyl)-1-tosylaziridine (**36**): Prepared using **General Procedure F** using S**31** and (***R***,***R***)-**2**. Purified via preparational TLC using (20% EtOAc in Hexanes) to provide 36 (0.0170 g, 54%yield, 94:6 e.r.) as colorless oil. **^{1}H NMR** (400 MHz, $CDCl_3$) δ 7.83 (d, *J* = 8.3 Hz, 2H), 7.32 (d, *J* = 8.0 Hz, 2H), 7.29 – 7.21 (m, 2H), 7.21 – 7.11 (m, 1H), 7.11 – 7.01 (m, 2H), 2.79 – 2.69 (m, 1H), 2.64 (d, *J* = 6.9 Hz, 1H), 2.55 (t, *J* = 7.5 Hz, 2H), 2.42 (s, 3H), 2.06 (d, *J* = 4.5 Hz, 1H), 1.68 – 1.48 (m, 3H), 1.39 – 1.24 (m, 1H). **^{13}C NMR** (101 MHz, $CDCl_3$) δ 144.64, 141.78, 135.24, 129.78, 128.43, 128.13, 125.98, 40.29, 35.19, 33.93, 30.88, 28.47, 21.75. **HRMS** (+APCI) calculated for $C_{18}H_{22}O_2NS$ $[M+H]^+$ 316.13658, found 316.13643. **HPLC** (IA column 10% 2-propanol in hexanes, 1.0 mL/min) t_M = 10.1 min t_m = 8.9 min, 94:6 e.r.

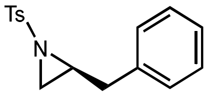

(S)-2-benzyl-1-tosylaziridine (**37**): Prepared using **General Procedure E** using allylbenzene (**S32**) and (***R***,***R***)-**2**. Purified via preparative TLC using (7:2:1, Hex:EtOAc:Et_3N) to provide **37** (0.0051 g, 18% yield, 95:5 e.r.) as white crystalline solid. **^{1}H NMR** (400 MHz, $CDCl_3$) δ 7.68 (d, *J* = 8.3 Hz, 2H), 7.21 (d, *J* = 7.9 Hz, 2H), 7.16 (dd, *J* = 4.9, 2.0 Hz, 3H), 7.04 (td, *J* = 4.9, 4.2, 3.2 Hz, 2H), 2.95 (tt, *J* = 7.0, 4.9 Hz, 1H), 2.81 (dd, *J* = 14.5, 5.2 Hz, 1H), 2.74 – 2.65 (m, 2H), 2.42 (s, 3H), 2.16 (d, *J* = 4.5 Hz, 1H). **^{13}C NMR** (101 MHz, $CDCl_3$) δ 144.45, 137.14, 135.00, 129.73, 128.86, 128.60, 128.02, 126.65, 41.32,

37.64, 32.97, 21.77. **HRMS** (+APCI) calculated for $C_{16}H_{18}O_2N^{32}S$ $[M+H]^+$ 288.10528, found 288.10601. **HPLC** (IA column 5% 2-propanol in hexanes, 1.0 mL/min) t_M = 14.7 min t_m = 13.0 min, 95:5 e.r.

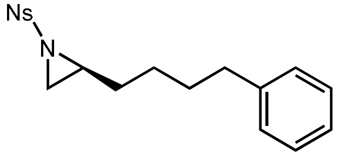

(S)-1-((4-nitrophenyl)sulfonyl)-2-(4-phenylbutyl)aziridine (**38**): Prepared using **General Procedure F** using **S30** and (***R***,***R***)-**2**. Purified via preparative TLC using (20% EtOAc in Hexanes) to provide **38** (0.0057 g, 16% yield, 94:6 e.r.) as a colorless oil. **^{1}H NMR** (400 MHz, $CDCl_3$) δ 8.36 (d, J = 8.9 Hz, 2H), 8.13 (d, J = 8.8 Hz, 2H), 7.30 – 7.24 (m, 2H), 7.21 – 7.15 (m, 1H), 7.14 – 7.08 (m, 2H), 2.88 (tt, J = 7.2, 4.8 Hz, 1H), 2.74 (d, J = 7.0 Hz, 1H), 2.55 (t, J = 7.6 Hz, 2H), 2.15 (d, J = 4.7 Hz, 1H), 1.69 – 1.53 (m, 3H), 1.46 – 1.26 (m, 3H).**^{13}C NMR** (101 MHz, $CDCl_3$) δ 150.69, 144.21, 142.05, 129.36, 128.48, 128.44, 126.00, 124.37, 41.38, 35.70, 34.73, 31.28, 30.80, 26.48. **HRMS** (+APCI) calculated for $C_{18}H_{21}O_4N_2S$ $[M+H]^+$ 361.12165, found 361.12133. **HPLC** (IB column 20% 2-propanol in hexanes, 1.0 mL/min) t_M = 22.8 min t_m = 19.7 min, 94:6 e.r.

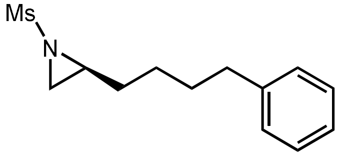

(S)-1-(methylsulfonyl)-2-(4-phenylbutyl)aziridine (**39**): Prepared using **General Procedure F** using **S30** and (***R***,***R***)-**2**. Purified via preparative TLC using (20% EtOAc in Hexanes) to provide **39** (0.0067 g, 26% yield, 87:13 e.r.) as a colorless oil. **^{1}H NMR** (400 MHz, $CDCl_3$) δ 7.31 – 7.26 (m, 2H), 7.21 – 7.14 (m, 3H), 3.01 (s, 3H), 2.72 (tt, J = 7.0, 4.9 Hz, 1H), 2.63 (t, J = 7.6 Hz, 2H), 2.59 (d, J = 7.0 Hz, 1H), 2.09 (d, J = 4.6 Hz, 1H), 1.75 – 1.56 (m, 3H), 1.55 – 1.45 (m, 3H).**^{13}C NMR** (101 MHz, $CDCl_3$) δ 142.27, 128.54, 128.49, 125.96, 39.77, 39.61, 35.85, 33.55, 31.37, 31.08, 26.63.**HRMS** (+APCI) calculated for $C_{13}H_{20}O_2NS$ $[M+H]^+$ 254.12093, found 254.12082. **HPLC** (IB column 20% 2-propanol in hexanes, 1.0 mL/min) t_M = 11.3 min t_m = 10.0 min, 87:13 e.r.

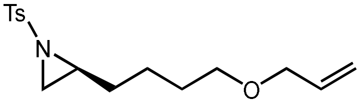

(S)-2-(4-(allyloxy)butyl)-1-tosylaziridine (**40**): Prepared using **General Procedure F** using **S31** and (***R***,***R***)-**2**. Purified via preparative TLC using (7:2:1, Hex:EtOAc:Et_3N) to provide **40** (0.0214 g, 69 % yield, 95:5 e.r.) as colorless oil. **^{1}H NMR** (400 MHz, $CDCl_3$) δ 7.82 (d, *J* = 8.3 Hz, 2H), 7.33 (d, *J* = 8.2 Hz, 2H), 5.89 (ddt, *J* = 17.2, 10.3, 5.6 Hz, 1H), 5.25 (dq, *J* = 17.3, 1.7 Hz, 1H), 5.16 (dq, *J* = 10.3, 1.4 Hz, 1H), 3.92 (dt, *J* = 5.6, 1.4 Hz, 2H), 3.33 (t, *J* = 6.5 Hz, 2H), 2.73 (tt, *J* = 7.0, 4.8 Hz, 1H), 2.62 (d, *J* = 7.0 Hz, 1H), 2.44 (s, 3H), 2.06 (d, *J* = 4.6 Hz, 1H), 1.64 – 1.46 (m, 3H), 1.45 – 1.27 (m, 3H).**^{13}C NMR** (101 MHz, $CDCl_3$) δ 144.55, 135.30, 135.06, 129.77, 128.13, 116.92, 71.96, 70.05, 40.35, 33.92, 31.24, 29.27, 23.64, 21.77.**HRMS** (+ESI) calculated for $C_{16}H_{24}O_3NS$ $[M+H]^+$ 310.14714, found 310.14709. **HPLC** (IH column 20% 2-propanol in hexanes, 1.0 mL/min) t_M = 14.0 min t_m = 18.1 min, 95:5 e.r.

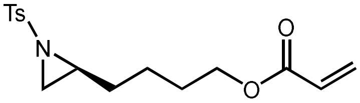

(S)-4-(1-tosylaziridin-2-yl)butyl acrylate (**41**): Prepared using **General Procedure F** using **S52** and (***R***,***R***)-**2**. Purified via preparative TLC using (7:2:1, Hex:EtOAc:Et_3N) to provide **41** (0.0159 g, 49 % yield, 95:5 e.r.) as colorless oil. **^{1}H NMR** (400 MHz, $CDCl_3$) δ 7.82 (d, *J* = 8.3 Hz, 2H), 7.33 (d, *J* = 7.9 Hz, 2H), 6.39 (dd, *J* = 17.3, 1.5 Hz, 1H), 6.10 (dd, *J* = 17.3,

10.4 Hz, 1H), 5.82 (dd, J = 10.4, 1.5 Hz, 1H), 4.04 (t, J = 6.6 Hz, 2H), 2.73 (tt, J = 7.0, 4.6 Hz, 1H), 2.64 (d, J = 7.0 Hz, 1H), 2.44 (s, 3H), 2.07 (d, J = 4.5 Hz, 1H), 1.69 – 1.53 (m, 3H), 1.41 – 1.28 (m, 3H). **^{13}C NMR** (101 MHz, $CDCl_3$) δ 166.32, 144.70, 135.23, 130.79, 129.79, 128.59, 128.13, 64.24, 40.13, 33.89, 30.99, 28.08, 23.44, 21.76. **HRMS** (+ESI) calculated for $C_{16}H_{22}O_4NS$ $[M+H]^+$ 324.12641, found 324.1266. **HPLC** (IA column 10% 2-propanol in hexanes, 1.0 mL/min) t_M = 13.6 min t_m = 12.5 min, 95:5 e.r.

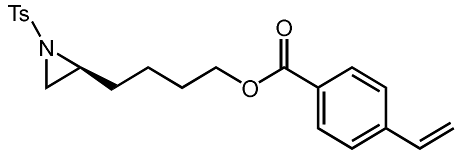

(S)-4-(1-tosylaziridin-2-yl)butyl 4-vinylbenzoate (**42**): Prepared using **General Procedure F** using **S53** and (***R***,***R***)**-2**. Purified via preparative TLC using (7:2:1, Hex:EtOAc:Et_3N) to provide **42** (0.0206 g, 52 % yield, 95:5 e.r.) as colorless oil. **^{1}H NMR** (400 MHz, $CDCl_3$) δ 7.98 (d, J = 8.4 Hz, 2H), 7.82 (d, J = 8.3 Hz, 2H), 7.47 (d, J = 8.2 Hz, 2H), 7.30 (dd, J = 8.6, 0.7 Hz, 2H), 6.76 (dd, J = 17.6, 10.9 Hz, 1H), 5.87 (dd, J = 17.6, 0.8 Hz, 1H), 5.39 (dd, J = 10.9, 0.7 Hz, 1H), 4.20 (td, J = 6.6, 1.4 Hz, 2H), 2.75 (tt, J = 7.1, 4.5 Hz, 1H), 2.66 (d, J = 7.0 Hz, 1H), 2.41 (s, 3H), 2.08 (d, J = 4.5 Hz, 1H), 1.76 – 1.60 (m, 3H), 1.46 – 1.33 (m, 4H). **^{13}C NMR** (101 MHz, $CDCl_3$) δ 166.46, 144.71, 142.10, 136.15, 135.24, 130.00, 129.83, 129.80, 129.56, 128.14, 126.26, 116.67, 64.64, 40.19, 33.93, 31.04, 28.21, 23.58, 21.74. **HRMS** (+ESI) calculated for $C_{22}H_{26}O_4NS$ $[M+H]^+$ 400.15771, found 400.158. **HPLC** (IH column 70% acetonitrile in water, 0.75 mL/min) t_M = 12.0 min t_m = 11.0 min, 95:5 e.r.

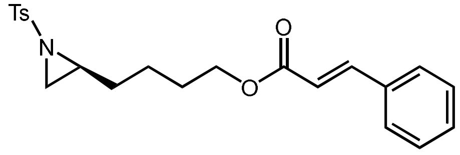

(S)-4-(1-tosylaziridin-2-yl)butyl cinnamate (**41**): Prepared using **General Procedure F** using **S54** and (***R***,***R***)**-2**. Purified via preparative TLC using (7:2:1, Hex:EtOAc:Et_3N) to provide **41** (0.0272 g, 68 % yield, 95:5 e.r.) as colorless oil. **^{1}H NMR** (400 MHz, $CDCl_3$) δ 7.83 (d, J = 8.3 Hz, 2H), 7.68 (d, J = 16.0 Hz, 1H), 7.57 – 7.48 (m, 2H), 7.44 – 7.35 (m, 3H), 7.32 (dd, J = 8.6, 0.8 Hz, 2H), 6.42 (d, J = 16.0 Hz, 1H), 4.10 (td, J = 6.6, 0.8 Hz, 2H), 2.75 (tt, J = 6.9, 4.5 Hz, 1H), 2.65 (d, J = 7.0 Hz, 1H), 2.43 (s, 3H), 2.08 (d, J = 4.6 Hz, 1H), 1.73 – 1.57 (m, 3H), 1.44 – 1.31 (m, 3H). **^{13}C NMR** (101 MHz, $CDCl_3$) δ 167.08, 144.90, 144.69, 135.24, 134.51, 130.44, 129.78, 129.04, 128.20, 128.13, 118.18, 64.25, 40.15, 33.92, 31.01, 28.18, 23.48, 21.75. **HRMS** (+ESI) calculated for $C_{22}H_{26}O_4NS$ $[M+H]^+$ 400.15771, found 400.15807. **HPLC** (IA column 70% acetonitrile in water, 0.75 mL/min) t_M = 16.7 min t_m = 19.2 min, 95:5 e.r.

5. Determination of Aziridine Product Stereochemistry

Stereochemistry of *2-benzyl-1-tosylaziridine* **37** was assigned by synthesizing *(R)-2-benzyl-1-tosylaziridine* from *(R)-2-amino-3-phenylpropan-1-ol* and synthesizing *(S)-2-benzyl-1-tosylaziridine* from *(S)-2-amino-3-phenylpropan-1-ol* then comparing HPLC data to the reaction HPLC data for the same substrate, shown below.

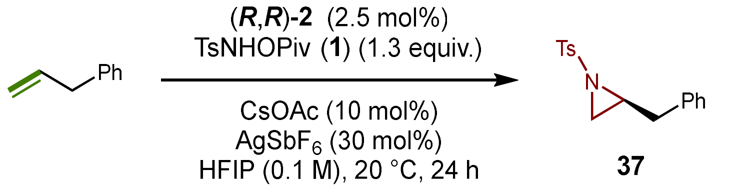

37
18% yield, 95:5 e.r.
t_M = 14.7 min t_m = 13.0 min

(S)-2-benzyl-1-tosylaziridine (**37**): See **Enantioselective Aziridine Scope** for full experimental. **HPLC** (IA column 5% 2-propanol in hexanes, 1.0 mL/min) t_M = 14.7 min t_m = 13.0 min, 95:5 e.r.

(±)-2-benzyl-1-tosylaziridine (±-**37**):

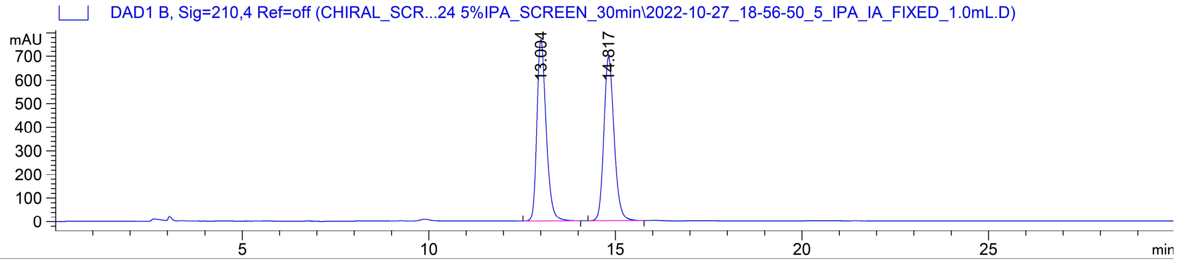

Signal 2: DAD1 B, Sig=210,4 Ref=off

Peak #	RetTime [min]	Type	Width [min]	Area [mAU*s]	Height [mAU]	Area %
1	13.004	BB	0.2642	1.32316e4	769.17761	49.9148
2	14.817	BB	0.2935	1.32768e4	694.65857	50.0852

Totals : 2.65083e4 1463.83618

(*S*)-2-benzyl-1-tosylaziridine (**37**):

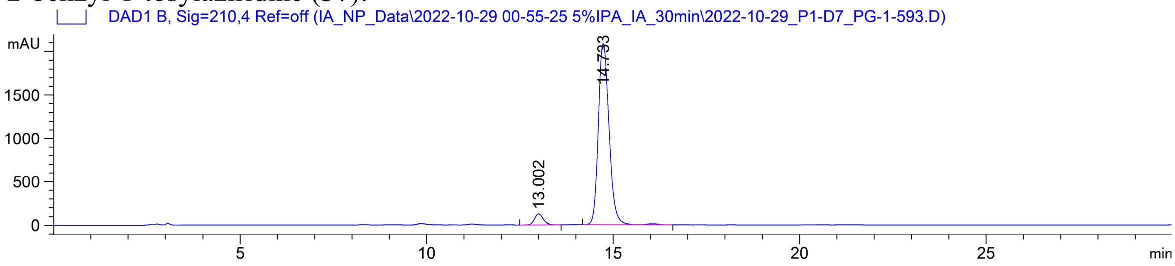

Signal 2: DAD1 B, Sig=210,4 Ref=off

Peak #	RetTime [min]	Type	Width [min]	Area [mAU*s]	Height [mAU]	Area %
1	13.002	BB	0.2471	2112.04272	127.29562	4.8152
2	14.733	BV R	0.2419	4.17496e4	2081.45728	95.1848

Totals : 4.38617e4 2208.75290

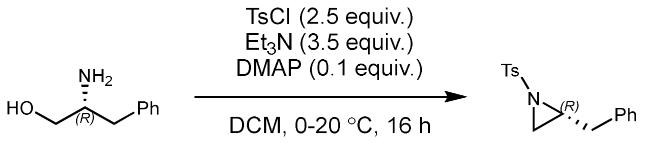

(*R*)-37
19% yield, single enantiomer
t_R = 12.9 min

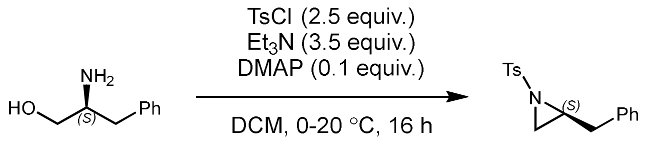

(*S*)-37
50% yield, single enantiomer
t_R = 14.6 min

Synthesis of chiral 2-benzyl-1-tosylaziridine from amino alcohols[26]: To a solution of chiral amino alcohol (0.76 g, 5.0 mmol, 1.0 equiv.) in dry CH_2Cl_2 (20 mL) at 0 °C was added Et_3N (2.4 mL, 18 mmol, 3.5 equiv.), and TsCl (2.4 g, 13 mmol, 2.5 equiv.). After 10 minutes, DMAP (61 mg, 0.5 mmol, 0.1 equiv.) was added and the reaction was allowed to stir under a N2 balloon at room temperature overnight. After which, the reaction was quenched DI H_2O and extracted with CH_2Cl_2 three times. The combined organic layers were dried Na_2SO_4 and the solvent removed under reduced pressure. The crude product was purified using silica gel column chromatography (5-10% EtOAc) and further purified via recrystallization from EtOAc and hexanes to yield (***R***)-**37** or (***S***)-**37** as a white solid.

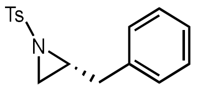

(R)-2-benzyl-1-tosylaziridine ((***R***)-**37**): (0.275 g, 19%yield, single enantiomer) **^{1}H NMR** (400 MHz, $CDCl_3$) δ 7.69 (d, *J* = 8.3 Hz, 2H), 7.21 (d, *J* = 8.0 Hz, 2H), 7.16 (dd, *J* = 4.9, 2.0 Hz, 3H), 7.09 – 6.99 (m, 2H), 2.95 (tt, *J* = 7.0, 4.8 Hz, 1H), 2.81 (dd, *J* = 14.5, 5.2 Hz, 1H), 2.75 – 2.64 (m, 2H), 2.42 (s, 3H), 2.16 (d, *J* = 4.5 Hz, 1H). **^{13}C NMR** (101 MHz, $CDCl_3$) δ 144.45, 137.13, 134.93, 129.72, 128.85, 128.59, 128.01, 126.63, 41.33, 37.63, 32.96, 21.77. **HRMS** (+APCI) calculated for $C_{16}H_{18}O_2N^{32}S$ $[M+H]^+$ 288.10528, found 288.10601. **HPLC** (IA column 5% 2-propanol in hexanes, 1.0 mL/min) t_R = 12.9 min $[\alpha]_D^{22}$ -16.3° (*c* = 2.3, $CHCl_3$)

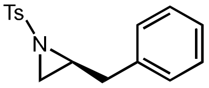

(S)-2-benzyl-1-tosylaziridine ((***S***)-**37**)[26] (0.712 g, 50%yield) **^{1}H NMR** (400 MHz, $CDCl_3$) δ 7.68 (d, *J* = 8.3 Hz, 2H), 7.21 (d, *J* = 7.9 Hz, 2H), 7.19 – 7.11 (m, 3H), 7.09 – 7.00 (m, 2H), 2.95 (tt, *J* = 7.1, 4.9 Hz, 1H), 2.81 (dd, *J* = 14.4, 5.2 Hz, 1H), 2.74 – 2.64 (m, 2H), 2.42 (s, 3H), 2.17 (d, *J* = 4.5 Hz, 1H). **^{13}C NMR** ^{13}C NMR (101 MHz, $CDCl_3$) δ 144.45, 137.13, 134.93, 129.72, 128.84, 128.58, 128.00, 126.63, 41.33, 37.62, 32.96, 21.77. **HPLC** (IA column 5% 2-propanol in hexanes, 1.0 mL/min) t_R= 14.6 $[\alpha]_D^{22}$ 16.6° (*c* = 2.3, $CHCl_3$)

(R)-2-benzyl-1-tosylaziridine ((***R***)-**37**):

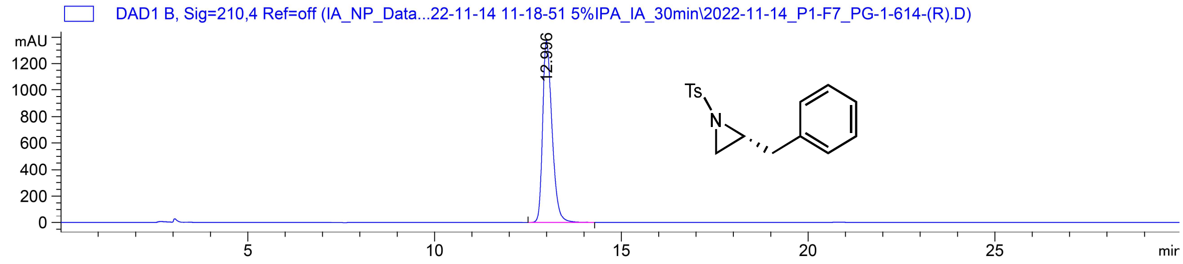

Signal 2: DAD1 B, Sig=210,4 Ref=off

Peak #	RetTime [min]	Type	Width [min]	Area [mAU*s]	Height [mAU]	Area %
1	12.996	BB	0.2626	2.39854e4	1370.93250	100.0000

Totals : 2.39854e4 1370.93250

(S)-2-benzyl-1-tosylaziridine ((***S***)-**37**):

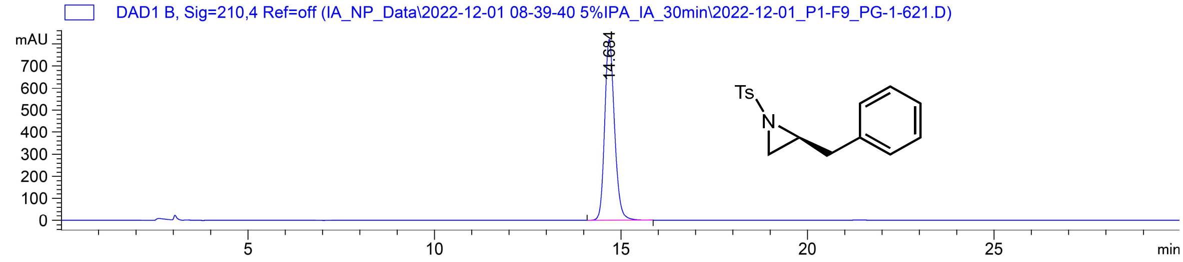

Signal 2: DAD1 B, Sig=210,4 Ref=off

Peak #	RetTime [min]	Type	Width [min]	Area [mAU*s]	Height [mAU]	Area %
1	14.684	BB	0.2811	1.50120e4	820.03436	100.0000

Totals : 1.50120e4 820.03436

6. Computational Data
6.1. Computational Details

All calculations except for the energy decomposition analysis, were conducted using DFT[27] as implemented in the Jaguar 9.1 suite[28] of ab initio quantum chemistry programs with B3LYP[29-30] levels of theory including Grimme's D3 dispersion correction.[31-32] Geometry optimizations proceeded using the 6-31G** basis set, with LACVP effective core potential[33-35] for rhodium, and cesium atoms. Analytical vibrational frequencies within the harmonic approximation were calculated using the 6-31G** basis to confirm proper convergence to well-defined minima or saddle points on the potential energy surface. Solvation energies were calculated using a self-consistent reaction field (SCRF)[36] approach based on accurate numerical solutions of the Poisson-Boltzmann equation and were performed with the 6-31G** basis at the optimized gas phase geometry with the dielectric constant of $\varepsilon = 16.7$ for hexafluoro-2-propanol. As is the case for all continuum models, the solvation energies are subject to empirical parametrization of the atomic radii that are used to generate the solute surface. The standard set of optimized radii in Jaguar was used for H (1.150 Å), C (1.900 Å), N (1.600 Å), O (1.600 Å), S (1.900 Å), Rh (1.464 Å), and Cs (2.259 Å).[37] The energies of the optimized structures were reevaluated by additional single point calculations on each optimized geometry using a triple-ζ quality cc-pVTZ(-f) basis set for main group elements,[38] and LACV3P effective core potential for rhodium and cesium atoms. The Gibbs free energies in solution phase G(sol) were computed with the following protocol.

$$G(sol) = G(gas) + G^{solv} \quad (1)$$

$$G(gas) = H(gas) - TS(gas) \quad (2)$$

$$H(gas) = E(SCF) + ZPE \quad (3)$$

$$\Delta E(SCF) = \Sigma E(SCF) \text{ for products} - \Sigma E(SCF) \text{ for reactants} \quad (4)$$

$$\Delta G(sol)^\circ = \Sigma G(sol) \text{ for products} - \Sigma G(sol) \text{ for reactants} \quad (5)$$

$$\Delta G(sol) = \Delta G(sol)^\circ + RT\ln(Q) \quad (6)$$

G(gas) is the free energy in gas phase; G^{solv} is the free energy of solvation; H(gas) is the enthalpy in gas phase; T is the temperature (293.15K); S(gas) is the entropy in gas phase; E(SCF) is "raw" electronic energy as computed from the SCF procedure which is the self-consistent field energy, and ZPE is the zero point energy. The entropy we refer to is specifically vibrational/rotational/translational entropy of the solute(s), and the entropy of the solvent is implicitly comprised in the continuum solvation model.

In the realm of Density Functional Theory (DFT) calculations, it is common practice to consider only a single molecule of each reacting species, a simplification that fails to account for concentration effects.[39] To address this limitation, we employ Eq. 6, wherein ΔG(sol)° represents the 'raw' Gibbs free energy obtained from the calculation, Q denotes the reaction quotient, and ΔG(sol) signifies the concentration-corrected Gibbs free energy. The calculation of Q hinges on the relative number of substrate molecules compared to the number of rhodium species involved in the reaction.

$$\text{CsOAc} \rightleftharpoons \text{Cs}^+ + \text{AcO}^- \quad \Delta G^\circ_1 = -4.19 \text{ (kcal/mol)} (7)$$

$$\text{Cs}^+ + \text{AcO}^- + \textbf{HA} \rightleftharpoons \text{CsNTsOpiv} + \text{AcOH} \qquad \Delta G^\circ_2 = -3.01 \text{ (kcal/mol)} (8)$$

In a previous study conducted by the Lebel group,[40] it was proposed that the deprotonation of hydroxylamine is feasible in the presence of carboxylate, a proposition that holds true for **HA**. The formation of CsNTsOPiv is exergonic by 7.2 kcal/mol, implying that the predominant state of **HA** is deprotonated, while the majority of acetate exists in the form of acetic acid. Notably, the chemical species described in Eq. 8 are recognized to exert a concentration effect on the formation of reaction intermediates. Utilizing the initial substrate concentrations depicted in Figure 2 and the energy difference derived from Eq. 8, we have successfully calculated the equilibrium concentrations of each species as follows:

$$[Cs^+]_0 = 1.0\times10^{-2}\ (M),\quad [AcO^-]_0 = 5\times10^{-3}\ (M),\quad [\mathbf{HA}]_0 = 1.3\times10^{-1}\ (M),\quad T = 293.15\ (K)$$

$$[Cs^+]_0 = [Cs^+] + [CsNTsOPiv] \qquad (9)$$

$$[\mathbf{HA}]_0 = [\mathbf{HA}] + [CsNTsOPiv] \qquad (10)$$

$$[AcO^-]_0 = [AcO^-] + [AcOH] \qquad (11)$$

$$[CsNTsOPiv][AcOH]/[Cs^+][AcO^-][\mathbf{HA}] = \exp(-\Delta G°_2/RT) \qquad (12)$$

As acetate coordinates with the rhodium catalyst to generate the active catalyst **A1**, we made the assumption that only half of the initial concentration of acetate, which is 5.0×10^{-3} M, would be involved in the equilibrium. Additionally, we approximated $[\mathbf{HA}]_0$ to be approximately equal to $[\mathbf{HA}]$ since the concentration of HA is sufficiently large to remain unaffected by the equilibrium. Upon solving Eq. 12, we obtained the following values:

$$[Cs^+] = 5.12\times10^{-3}\ (M)$$

$$[AcO^-] = 1.95\times10^{-4}\ (M)$$

$$[CsNTsOpiv] = [AcOH] = 4.81\times10^{-3}\ (M)$$

All other steps involving **HA**, AcOH, and 1-hexene are similarly adjusted using Eq. 6, taking into account the concentrations previously mentioned and illustrated in Figure 2.

For species involving explicit solvent molecules, the concentration of solvent in the reaction mixture was assumed to be same to that of pure HFIP. The concentration of HFIP was obtained from the density (1.596 g/mL) and molar mass of HFIP (168.04 g/mol), and calculated to be 9.50 M. This value was used for the concentration correction for the free energy of $\mathbf{A4_{HFIP}}$.

The energy decomposition analysis[41-42] was conducted as implemented in the Amsterdam Density Functional (ADF) 2019 suite of ab initio quantum chemistry programs.[43] Calculations were carried out using B3LYP-D3 functional, and triple-ζ quality TZ2P basis set[44-45] without frozen core. The scalar level of zeroth-order regular approximation (ZORA)[46-47] was applied for relativistic effect correction.

6.2. Olefin Concerted Metalation-Deprotonation (CMD) Pathway

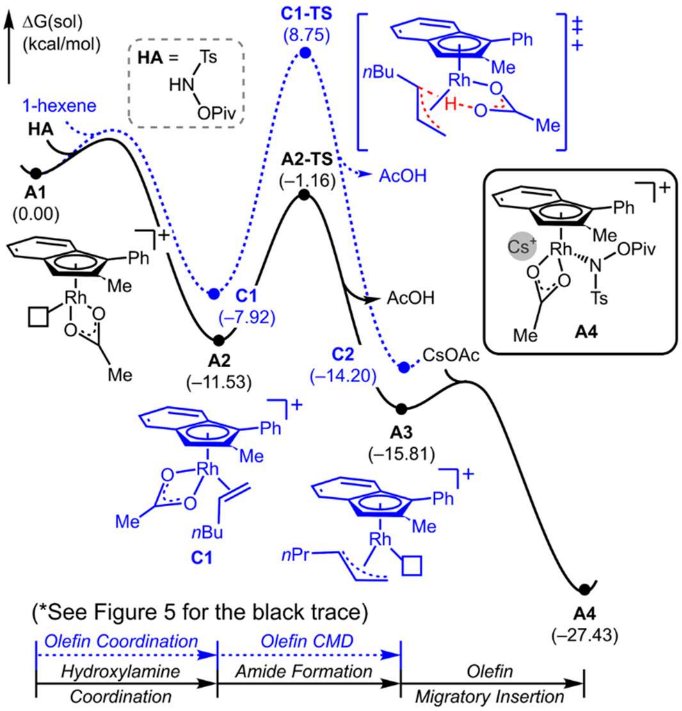

Figure S1. Energy profile of olefin CMD pathway

In Figure S1, we present an alternative pathway known as the olefin CMD pathway. In this route, the olefin substrate 1-hexene coordinates with the active catalyst **A1**, resulting in the formation of intermediate **C1**. Subsequent to this coordination, the olefin undergoes CMD facilitated by carboxylate, culminating in the generation of the 16 e$^-$ π-allyl complex **C2**. This complex can further engage with **HA**. Importantly, the CMD step involving the olefin presents a notably higher activation energy barrier of 16.7 kcal/mol, while the amide formation step requires a substantially lower energy input of only 10.4 kcal/mol. This significant energy difference strongly favors the hydroxylamine activation pathway.

6.3. Calculated Nitrene Formation Pathways

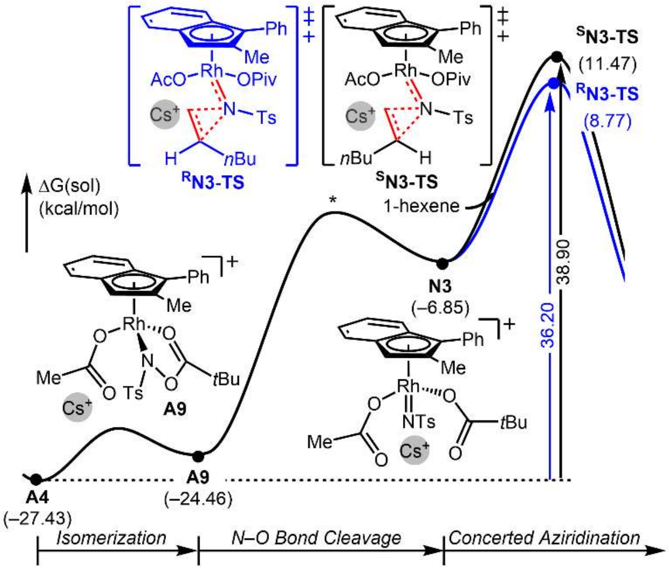

Figure S2. Energy profile of Concerted aziridination of Cs-nitrene species

Our next consideration involved the potential involvement of Cs^+ in the formation of a nitrene intermediate. Given the crucial role of N–O bond cleavage in nitrene formation, the presence of Cs^+ could potentially facilitate this process. To explore this possibility, we investigated the potential for nitrene formation starting from the resting state **A4**.

The isomerization of **A4** leads to the formation of **A9**, where rhodium coordinates with the pivaloyl group, replacing κ^2-acetate with κ^1-acetate. Subsequent to this rearrangement, the N–O bond cleavage of **A9** yields a relatively stable Rh(V)-nitrene species, denoted as **N3**. While the concerted aziridination of **N3** through the **N3-TS** transition state can yield the desired product, it is noteworthy that the energy barrier for both **^{S}N3-TS** and **^{R}N3-TS** is approximately 35 kcal/mol, significantly higher than that observed in the olefin insertion mechanism. Furthermore, the predicted selectivity is inversed to the observed selectivity.

6.4. Analysis of Olefin Migratory Insertion Step

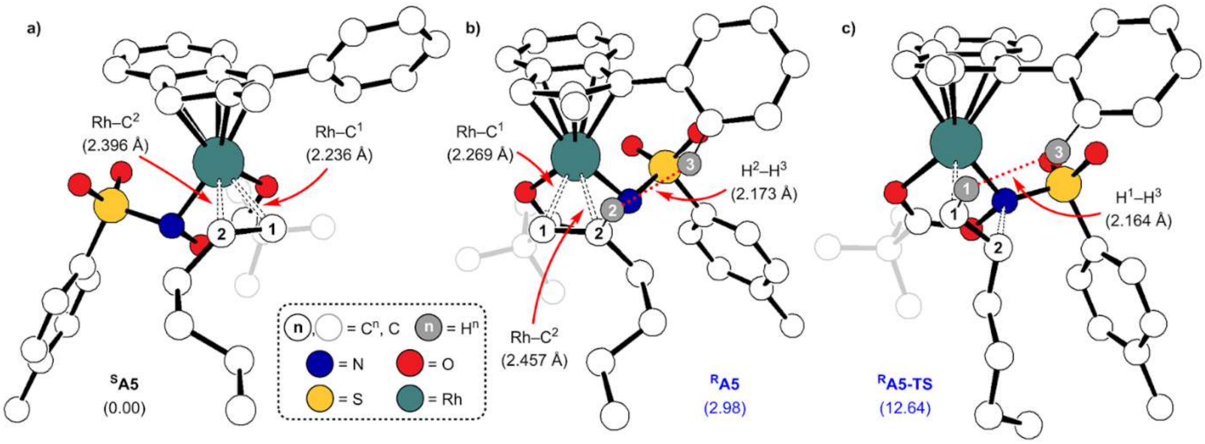

Figure S3. Depictions of a) ^{S}**A5**, b) ^{R}**A5**, c) ^{R}**A5-TS**. The relative energies of these structures and interatomic distances are provided within parentheses. Hydrogen atoms not contributing to steric interactions are omitted for clarity.

Upon comparing the intermediate structures of ^{S}**A5** and ^{R}**A5**, notable differences arise in the Rh–C^1 and Rh–C^2 bond lengths. Specifically, in ^{S}**A5**, the Rh–C^1 bond length is 2.236 Å, which is shorter than that of ^{R}**A5** (2.269 Å). Furthermore, ^{S}**A5** exhibits a shorter bond length for Rh–C^2 bond (2.396 Å) which is 2.269 Å in ^{R}**A5** (Figure S3). These distinctions can be attributed to a steric clash between the phenyl substituent on **A3** and the olefin substrate, as evidenced by the close proximity of hydrogen atoms from the olefin and phenyl group in ^{R}**A5** (H^2–H^3 = 2.173 Å). Remarkably, ^{R}**A5-TS** also demonstrates a similar steric clash (H^1–H^3 = 2.164 Å) which is avoided in ^{S}**A5-TS**.

Consequently, one might anticipate variations in the Rh–C^1 and N–C^2 distances, impacting the Rh–C^1 and N–C^2 bond formation processes. However, to our surprise, the distances involving Rh–C^1, N–C^2, Rh–N, and C^1–C^2 appear nearly identical between ^{S}**A5-TS** and ^{R}**A5-TS**. This observation suggests that the differences in energy barriers are less likely to arise from orbital interactions between **A3** and the olefin substrate.

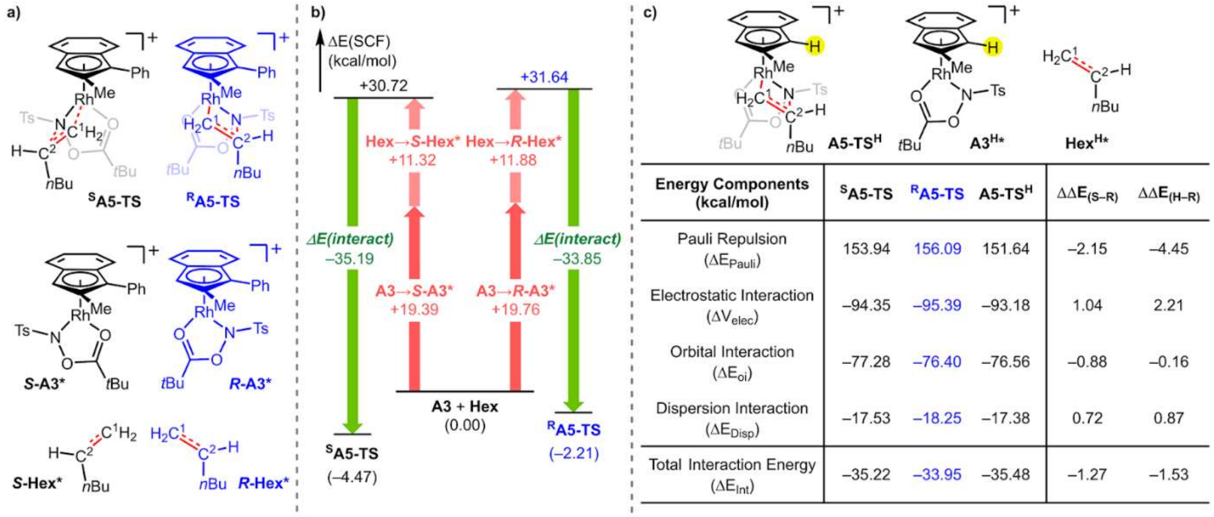

Energy Components (kcal/mol)	^{S}A5-TS	^{R}A5-TS	A5-TS^H	$\Delta\Delta E_{(S-R)}$	$\Delta\Delta E_{(H-R)}$
Pauli Repulsion (ΔE_{Pauli})	153.94	156.09	151.64	−2.15	−4.45
Electrostatic Interaction (ΔV_{elec})	−94.35	−95.39	−93.18	1.04	2.21
Orbital Interaction (ΔE_{oi})	−77.28	−76.40	−76.56	−0.88	−0.16
Dispersion Interaction (ΔE_{Disp})	−17.53	−18.25	−17.38	0.72	0.87
Total Interaction Energy (ΔE_{Int})	−35.22	−33.95	−35.48	−1.27	−1.53

Figure S4. a) Fragments used in the distortion-interaction analysis of ^{S}**A5-TS** and ^{R}**A5-TS**, b) energy components in distortion-interaction analysis, and c) Fragments used for energy decomposition analysis of **A5-TS^{H}**, and components for energy decomposition analysis of ^{S}**A5-TS**, ^{R}**A5-TS**, and **A5-TS^{H}**.

We undertook a comprehensive investigation of two transition states, namely ^{S}**A5-TS** and ^{R}**A5-TS**, in order to elucidate the underlying cause of the disparity in energy barriers. To achieve this, we employed Distortion-Interaction analyses on both

^{S}**A5-TS** and ^{R}**A5-TS**. These transition states were divided into two distinct components: one originating from **A3**, denoted as **A3***, and the other arising from the approaching 1-hexene(**Hex**), denoted as **Hex***, as illustrated in Figure 8b.

Our analysis unveiled that ^{S}**A5-TS** necessitates 0.9 kcal/mol less energy to reach compared to ^{R}**A5-TS**. In detail, ^{S}**A5-TS** has an advantage of 1.3 kcal/mol greater interaction relative to ^{R}**A5-TS**. Given the significance of interaction energy in favoring ^{S}**A5-TS** over ^{R}**A5-TS**, we conducted an energy decomposition analysis to pinpoint the primary factor contributing to the disparity in energy barriers (see Computational details for details on energy decomposition analysis).

The results of this analysis revealed that two factors, Pauli repulsion (ΔE_{Pauli}) play a pivotal role in favoring ^{S}**A5-TS**, contributing –2.2 kcal/mol. The minor contribution from orbital interaction (ΔE_{Oi}) strengthens the barrier difference for 0.9 kcal/mol, favoring ^{S}**A5-TS**. These components overwhelmingly outweigh the unfavorable interactions, which include 1.0 kcal/mol of Electrostatic interaction (ΔV_{elec}) and 0.7 kcal/mol of Dispersion interaction (ΔE_{Disp}). Collectively, these factors contribute to a net advantage of 1.3 kcal/mol favoring ^{S}**A5-TS** over ^{R}**A5-TS**.

Furthermore, to reinforce the idea that the ΔE_{Pauli} difference arises from the presence of the phenyl group on the Ind ligand, we performed an energy decomposition analysis on **A5-TS^{H}**, which substitutes the phenyl group on the Ind ligand with hydrogen. In comparing each term between ^{R}**A5-TS** and **A5-TS^{H}**, we observed that ΔE_{Pauli} was the most substantial factor, favoring **A5-TS^{H}** by –4.5 kcal/mol, whereas the other factors had a smaller impact on interaction energy. This comparison underscores the dominant role of ΔE_{Pauli}, primarily induced by the phenyl group on the Ind ligand, as the primary contributor to the barrier difference.

6.5. Solvation Modeling of Cs^+ and Explicit Solvent Molecules

The implicit solvation model used in this study does not explicitly describe the hydrogen bonding between solute and solvent, which may mislead that the implicit solvation model cannot account for hydrogen bonding with solvent. However, these interactions are incorporated by training the model with training set which should contain hydrogen bonding. Thus, implicit solvent model will describe the explicit interaction with solvent as good as other interactions such as van der Waals interactions and electrostatic forces.

Nontheless, we conducted additional calculations to find out the possible species that may influence the overall reaction. We expect the solvation of transition states would be minimal, since the lifetime of these transient species would be too short for solvent molecules to rearrange to optimal solvation shell. Thus, the additional calculations with explicit solvent molecules were only done with intermediates.

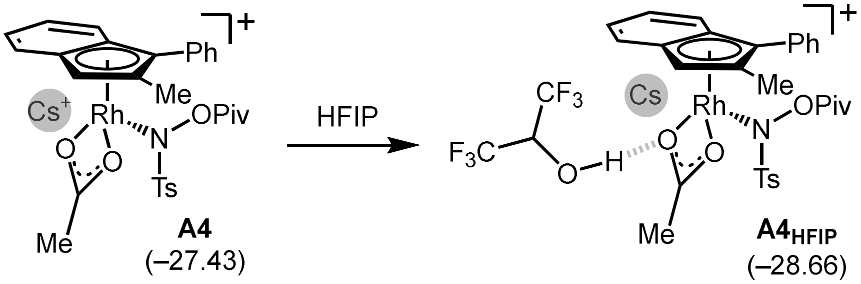

Figure S5. Additional calculation using an explicit solvent molecule with **A4**

The reaction intermediates, **A3**, **A4**, ^{S}**A5**, and ^{R}**A5** were calculated with additional explicit HFIP molecules. The majority of the conformers with HFIP molecule show higher energy compared to those without HFIP molecule, the intermediate **A4** with HFIP molecule denoted by **A4**$_{\mathbf{HFIP}}$, deviates from the trend, which is stabilized for 1.2 kcal/mol compared to **A4**. This is expected to be the effect of hydrogen bonding between HFIP and tosyl group of **A4**, and electrostatic interaction between HFIP and Cs^+.

Meanwhile, the accuracy of modeling the Cs^+ containing intermediate **A4** may have problem, because It is challenging to obtain the accurate solvation energy of Cs^+ ion. The cluster-continuum solvation model might be the alternative for simple implicit solvation model. The primary goal of the computational study is to provide mechanistic insight, we considered that providing the upper limit of the barrier would be proper for the readers. In this context, including the **A4** would be proper. For same reason, the cluster-continuum solvation model might be less appropriate, since these frozen structures of cluster will not properly reflect the fluctional behavior of solvent molecules surrounding the Cs^+ ion. The optimized Cs^+ cluster structure will overestimate the solvation energy of Cs^+ ion which will overestimate the energy of **A4**, and underestimate the barrier. Thus, we chose to use simple implicit solvation model for simplicity and consistency. The challenges of the cluster-continuum models can be found from the reference 39.

6.6. Computed Energy Components for Optimized Structures

Table S1. Computed Energy Components for Optimized Structures

	E(SCF) (eV)	ZPE (kcal/mol)	S(gas) (cal/mol K)	G^{Solv} (kcal/mol)
	B3LYP-D3 /cc-pVTZ(-f)/LACV3P	B3LYP-D3 /6-31G**/LACVP	B3LYP-D3 /6-31G**/LACVP	B3LYP-D3 /6-31G**/LACVP
1-hexene	–6420.366	104.22	85.57	–1.38
**HA**	–33241.971	177.05	145.21	–11.95
CsOAc	–6762.369	31.17	86.97	–34.95
AcOH	–6236.253	38.90	68.82	–8.16
PivOH	–9446.948	92.55	86.69	–7.50
**A1**	–26004.538	183.05	145.80	–46.43
**A2**	–59248.373	361.73	241.72	–41.10
**A2-TS**	–59247.748	358.53	239.00	–42.75
**A3**	–53011.362	321.46	212.22	–42.53
**A3-TS**	–53009.725	319.12	215.58	–42.91
**A4**	–59776.178	353.83	247.94	–51.59
**$A4_{HFIP}$**	-81276.820	393.81	304.81	-50.87
^{S}**A5**	–59432.325	427.27	255.19	–40.61
^{R}**A5**	–59432.212	427.19	253.16	–40.76
^{S}**A5-TS**	–59431.922	426.85	249.48	–40.38
^{R}**A5-TS**	–59431.824	426.73	250.21	–40.45

**A5-TS^H**	–53142.237	375.94	221.14	–41.66
**^SA6**	–59432.727	428.92	245.75	–37.77
**^RA6**	–59432.667	428.65	246.00	–37.70
**^SA6-TS**	–59432.052	426.84	247.90	–38.39
**^RA6-TS**	–59431.985	426.62	254.29	–38.81
**^SA7**	–59432.632	428.15	254.76	–42.56
**^RA7**	–59432.516	428.26	250.07	–43.01
**^SA7-TS**	–59432.341	427.27	252.29	–41.84
**^RA7-TS**	–59432.164	427.23	242.66	–42.51
**^SA8**	–59433.743	429.06	247.57	–40.50
**^RA8**	–59433.674	428.92	248.82	–40.26
**A9**	–59775.947	354.06	250.83	–53.33
**C1**	–32426.035	289.13	187.21	–42.71
**C1-TS**	–32425.210	285.72	182.94	–42.91
**C2**	–26189.155	248.73	159.62	–41.15
**N1**	–49799.619	266.73	195.81	–47.44
**N2**	–53010.437	320.33	213.06	–45.66
**N3**	–59775.135	352.59	253.44	–52.21
**^SN3-TS**	–66195.459	458.33	287.42	–51.14
**^RN3-TS**	–66195.499	458.03	292.28	–51.18

6.7. Vibrational Frequencies (in cm^{-1}) of the Optimized Structures

```
==============================
1-hexene
==============================
  77.01   98.76  134.45  182.26  260.05  362.30
 365.75  469.70  654.31  751.39  811.41  908.34
 936.02  939.06  951.73 1028.39 1036.76 1059.41
1066.73 1130.53 1203.60 1255.68 1281.77 1324.32
1331.17 1343.94 1368.32 1413.10 1430.39 1465.42
1497.36 1503.74 1512.86 1514.41 1523.93 1729.40
3003.23 3016.96 3024.57 3035.21 3045.30 3056.98
3076.39 3105.07 3109.45 3128.88 3146.59 3230.92
==============================
HA
==============================
  18.42   21.93   37.91   57.49   60.79   79.17
 110.79  150.53  173.16  198.06  221.60  228.47
 251.05  277.92  280.28  289.10  319.98  332.85
 338.47  350.03  372.32  390.21  413.85  418.09
 445.41  482.39  504.17  519.46  572.78  606.63
 644.79  646.17  670.30  714.58  763.45  814.22
 817.27  832.08  859.40  879.77  942.13  951.43
 962.46  977.14  986.90  990.03 1021.24 1033.94
1040.85 1062.95 1065.12 1067.03 1096.13 1132.33
1154.58 1157.03 1226.62 1236.33 1236.54 1259.00
1306.26 1331.02 1344.84 1359.25 1418.96 1421.80
1427.69 1445.88 1448.96 1457.81 1491.12 1497.97
1501.16 1501.97 1504.54 1514.74 1518.80 1536.29
1539.12 1628.65 1655.05 1813.03 3040.96 3047.86
3051.91 3056.73 3103.75 3117.65 3127.89 3131.76
3132.20 3139.30 3143.89 3151.59 3187.06 3188.43
3213.42 3223.51 3359.95
==============================
CsOAc
==============================
  32.10   60.54  140.80  161.43  443.30  614.05
 643.36  912.08 1018.75 1052.29 1358.37 1422.59
1481.40 1494.80 1675.24 3041.14 3107.16 3141.55
==============================
AcOH
==============================
  72.48  421.63  541.15  584.78  679.63  867.45
1002.43 1069.26 1220.08 1355.66 1422.95 1482.41
1488.47 1858.77 3067.90 3134.24 3186.71 3752.54
==============================
PivOH
==============================
  45.99  218.26  242.40  263.64  287.81  295.81
 357.58  367.95  386.20  516.45  576.58  616.56
 730.48  787.30  870.93  951.55  960.67  983.85
1056.68 1062.67 1157.28 1239.74 1253.76 1281.87
1366.55 1414.18 1422.62 1451.52 1492.36 1500.07
1505.72 1515.25 1520.00 1539.90 1838.35 3043.55
3045.59 3053.79 3113.63 3115.29 3127.06 3135.36
3137.37 3140.66 3746.45
==============================
A1
==============================
  22.95   52.65   67.63   75.21   77.14   79.14
  90.43  116.00  123.45  150.04  155.63  186.77
 219.39  236.91  273.35  283.97  316.37  334.15
 349.87  397.97  417.80  446.90  461.39  481.61
 487.23  496.69  537.86  557.65  595.93  607.56
 621.97  650.93  658.90  696.62  707.03  716.44
 719.23  777.84  782.58  857.64  871.39  887.13
 919.62  925.57  954.70  963.28  989.92  993.92
 998.25 1011.94 1012.72 1023.73 1028.62 1030.27
1039.05 1055.49 1061.64 1068.46 1123.98 1139.80
1179.63 1196.00 1206.46 1222.23 1228.30 1243.71
1324.90 1327.44 1353.25 1373.68 1391.90 1401.55
1421.32 1427.54 1433.34 1471.26 1472.18 1486.23
1486.68 1493.62 1496.89 1500.69 1519.04 1524.93
1541.72 1589.38 1624.00 1638.89 1651.01 3040.63
3057.72 3113.40 3126.07 3163.24 3176.89 3192.34
3195.54 3204.35 3206.50 3213.21 3215.87 3221.57
3225.68 3235.35 3241.09
==============================
A2
==============================
  13.42   21.02   23.25   27.41   32.59   43.77
  47.05   58.00   60.94   63.80   75.36   79.06
  81.43   91.06   94.54  102.20  112.03  114.55
 117.53  127.81  132.51  157.79  164.73  169.19
 178.13  188.00  209.07  210.39  217.03  231.42
 237.23  240.07  268.96  272.28  280.05  285.33
 287.76  301.48  309.21  312.43  321.51  326.45
 341.05  347.21  366.02  387.02  396.18  407.01
 413.60  420.79  440.82  443.77  467.54  467.69
 481.21  488.17  494.17  498.52  513.63  539.73
 547.14  564.14  572.89  606.10  610.87  617.26
 627.10  643.10  648.60  656.23  657.95  688.09
 699.73  705.78  709.42  722.66  725.22  762.20
 776.61  784.12  814.53  820.84  829.59  847.93
 854.91  874.11  884.36  901.21  906.68  926.69
 950.42  954.69  957.64  960.86  965.83  978.41
 987.30  987.56  991.19  991.48  996.49 1016.54
1019.06 1023.02 1023.53 1024.37 1028.94 1029.72
1038.31 1049.83 1060.96 1062.22 1063.16 1065.56
1074.64 1088.93 1107.84 1122.88 1141.76 1152.60
1160.03 1176.58 1189.64 1193.37 1203.72 1222.53
1227.76 1228.17 1234.30 1237.44 1245.42 1255.93
1297.09 1317.79 1329.43 1342.93 1348.90 1355.36
1361.59 1374.24 1390.17 1395.21 1405.69 1421.36
1424.98 1425.43 1428.35 1429.62 1445.97 1450.01
1456.24 1456.58 1476.17 1487.59 1490.57 1491.87
1496.38 1498.22 1498.58 1499.98 1503.34 1503.74
1504.40 1514.91 1515.78 1518.62 1532.72 1535.33
1549.92 1561.39 1581.36 1622.02 1633.43 1646.19
1651.29 1660.34 1817.98 3047.45 3053.34 3055.00
3059.37 3059.93 3062.61 3112.06 3128.26 3130.64
3135.04 3137.27 3138.21 3139.44 3141.22 3141.30
3142.53 3154.08 3173.71 3189.40 3194.80 3195.57
3197.35 3199.34 3204.47 3212.91 3213.47 3216.42
3218.72 3229.30 3235.82 3255.66 3262.88 3267.79
==============================
A2-TS
==============================
-659.11   18.53   25.86   28.85   29.54   33.29
  38.02   51.50   52.07   61.03   64.82   66.62
  71.97   74.21   78.70   83.36   96.64  101.91
 127.96  138.87  151.74  157.71  168.93  174.54
 179.19  189.99  208.38  217.05  225.79  228.97
 233.87  238.00  239.58  272.80  277.98  282.86
 286.49  293.97  298.40  309.89  317.86  327.47
 329.43  335.45  351.72  365.79  369.17  389.48
 410.74  413.89  418.89  443.32  449.28  461.89
 477.70  478.90  497.23  501.52  520.35  533.82
 541.69  554.41  585.02  606.19  607.81  622.83
 625.55  627.23  643.85  652.59  658.29  671.35
 701.48  705.35  711.16  717.23  729.39  753.47
 776.81  783.89  785.28  815.67  827.75  829.43
 853.97  854.26  872.31  875.27  882.02  898.21
 922.14  941.63  951.59  959.11  961.91  976.60
 979.95  985.09  987.19  989.14  989.22  999.28
1012.50 1020.87 1022.88 1023.57 1024.93 1030.34
1030.85 1039.94 1044.19 1052.18 1061.38 1061.96
1063.11 1068.63 1077.37 1090.08 1126.09 1140.46
1143.82 1159.93 1176.01 1193.86 1204.75 1224.07
1227.68 1230.69 1236.59 1238.10 1239.06 1257.00
1290.89 1322.84 1326.80 1328.41 1345.46 1353.50
1361.12 1376.94 1388.78 1396.44 1414.21 1417.24
```

1423.75 1426.05 1428.14 1429.96 1446.73 1452.83
1468.07 1472.15 1484.38 1485.23 1491.38 1492.27
1492.67 1498.11 1498.37 1499.13 1502.45 1503.34
1505.94 1515.46 1518.62 1524.49 1534.38 1536.64
1543.57 1591.33 1623.28 1627.43 1645.04 1647.98
1649.28 1654.15 1717.12 1877.36 3046.04 3048.74
3049.91 3053.25 3059.13 3065.80 3110.47 3125.71
3129.57 3131.69 3135.34 3136.49 3138.60 3139.27
3141.25 3152.48 3179.58 3191.93 3192.50 3197.47
3199.26 3201.48 3201.74 3208.39 3212.44 3216.89
3221.24 3223.26 3227.22 3230.68 3239.09 3252.50
==============================
A3
==============================
20.78 28.80 30.86 32.90 39.38 46.15
58.88 61.88 65.12 77.74 81.58 84.76
100.14 121.61 127.74 151.43 156.53 164.06
192.20 203.79 207.41 222.81 231.63 232.96
242.56 245.93 258.41 277.91 280.47 285.15
290.05 299.48 310.54 324.99 336.47 341.90
348.08 382.41 392.43 413.02 418.09 421.44
435.08 442.52 451.91 466.05 479.00 480.16
494.08 500.60 538.31 544.93 555.32 557.38
588.23 608.29 618.84 622.64 644.19 653.04
656.45 658.68 700.67 707.39 720.80 729.98
758.26 764.13 778.69 784.62 802.13 817.23
829.93 847.38 854.75 855.33 877.33 885.07
903.95 922.19 945.97 963.55 965.92 977.78
980.29 983.74 984.95 988.99 990.66 1005.86
1007.80 1021.98 1022.49 1023.94 1030.13 1031.06
1038.75 1051.80 1055.98 1061.87 1064.05 1065.62
1084.81 1124.38 1139.57 1140.95 1158.16 1175.84
1192.27 1204.41 1224.13 1225.57 1227.35 1229.68
1237.11 1238.94 1240.11 1259.07 1315.74 1323.05
1327.58 1343.10 1349.20 1353.56 1360.07 1376.35
1394.78 1415.90 1418.64 1426.95 1428.63 1430.14
1446.64 1456.84 1463.10 1483.32 1489.66 1492.28
1496.25 1498.33 1500.20 1501.93 1504.39 1506.21
1515.60 1518.97 1520.18 1534.84 1536.99 1543.74
1589.35 1624.42 1625.31 1627.95 1646.56 1649.67
1654.57 3045.32 3048.22 3050.57 3055.49 3061.46
3108.68 3128.46 3130.90 3133.99 3137.17 3137.83
3142.39 3146.94 3156.00 3190.14 3192.41 3193.81
3194.92 3200.15 3201.92 3203.36 3209.20 3212.49
3217.04 3222.57 3222.85 3223.55 3235.50 3256.25
==============================
A3-TS
==============================
-99.45 20.71 23.06 31.74 34.96 40.26
52.31 53.80 58.85 64.12 68.28 76.53
86.35 102.54 105.97 122.91 133.45 143.40
153.68 163.60 172.85 188.26 194.76 219.54
230.91 235.86 240.44 263.68 269.20 277.86
286.11 287.07 289.17 298.62 319.81 322.69
332.63 350.88 367.25 376.48 391.00 409.24
414.78 418.06 431.42 443.21 456.14 465.03
481.73 488.86 492.90 530.42 531.96 533.80
558.82 600.50 609.81 625.83 634.55 639.83
648.98 652.30 662.73 701.28 704.34 708.44
715.72 721.11 770.03 782.64 790.44 812.23
813.60 829.09 850.52 851.81 868.75 876.50
894.49 908.12 921.54 949.43 959.19 960.41
980.37 983.37 985.99 990.58 993.48 994.57
1014.42 1019.81 1021.08 1021.32 1027.64 1030.33
1035.33 1055.29 1060.11 1060.97 1061.98 1064.74
1066.43 1124.04 1130.03 1140.29 1160.23 1172.51
1191.20 1204.64 1220.82 1226.51 1228.69 1230.80
1237.35 1238.15 1254.17 1260.08 1281.43 1323.51
1327.19 1343.29 1351.39 1359.62 1374.28 1397.78
1407.32 1410.12 1418.64 1425.65 1426.54 1429.53
1448.50 1458.76 1472.57 1481.74 1490.69 1491.26
1494.98 1495.58 1497.16 1501.37 1502.93 1504.69
1506.91 1515.83 1517.19 1528.80 1532.39 1540.09
1545.81 1585.59 1605.85 1627.71 1635.00 1640.95
1654.27 3043.47 3050.12 3051.03 3055.65 3055.85
3107.46 3121.39 3126.11 3126.48 3129.41 3139.45
3140.15 3142.03 3144.43 3154.45 3192.98 3195.57
3196.49 3196.52 3205.29 3210.13 3212.02 3219.63
3221.06 3223.65 3228.36 3236.49 3242.02 3250.47
==============================
A4
==============================
20.61 25.40 34.65 36.56 40.42 51.18
56.69 59.32 62.21 66.28 69.34 73.95
77.73 81.46 85.33 91.25 97.74 102.35
108.20 117.25 126.18 132.54 141.87 154.74
158.45 170.13 182.34 191.37 198.23 217.73
222.86 229.08 233.91 237.00 253.21 263.54
274.73 282.46 288.68 293.85 299.26 303.93
317.65 323.93 337.39 341.00 351.08 375.35
392.10 392.67 410.38 417.90 419.97 436.91
446.35 459.03 464.13 470.97 484.73 490.03
495.99 539.11 546.43 549.23 560.43 584.82
603.95 608.36 621.77 626.92 641.86 651.02
657.54 665.25 685.27 704.19 710.32 720.02
726.53 766.33 773.37 778.69 796.01 812.43
823.51 833.57 842.92 859.86 864.21 872.79
880.89 894.40 916.03 920.25 947.63 952.99
959.70 960.07 977.61 982.66 983.87 989.34
990.35 991.58 1012.31 1015.46 1017.34 1018.84
1022.48 1025.92 1032.11 1033.03 1053.30 1055.01
1062.01 1063.80 1065.17 1068.64 1084.15 1118.82
1132.61 1138.65 1140.18 1157.51 1172.50 1188.59
1202.05 1220.73 1225.11 1226.36 1226.95 1236.51
1237.05 1262.36 1283.74 1296.63 1328.00 1329.53
1345.17 1353.66 1354.42 1368.25 1386.06 1397.66
1414.12 1420.66 1422.43 1428.62 1432.16 1443.70
1452.48 1461.42 1467.44 1475.93 1484.60 1490.31
1492.75 1494.73 1497.95 1499.99 1500.82 1503.04
1505.25 1505.46 1511.13 1515.21 1518.95 1536.62
1537.50 1547.15 1571.54 1583.34 1624.57 1633.98
1652.99 1656.46 1660.22 1809.65 3044.77 3045.72
3053.75 3055.41 3059.90 3067.04 3105.25 3114.98
3123.16 3128.87 3131.13 3132.70 3143.00 3144.70
3147.03 3148.68 3165.54 3166.71 3183.52 3186.10
3188.34 3193.60 3196.08 3208.91 3209.76 3216.71
3218.02 3229.16 3231.54 3240.03 3242.09 3254.92

$A4_{HFIP}$
==============================
11.48 14.92 25.01 27.07 29.96 36.56
39.02 42.98 45.01 49.28 50.26 52.76
59.85 59.94 62.58 71.05 72.77 73.17
77.87 79.29 84.97 85.91 94.24 97.85
105.70 107.45 112.56 124.28 130.30 140.75
142.99 156.36 160.93 168.56 170.23 173.47
197.45 200.10 217.41 226.90 230.96 231.57
237.22 243.53 256.78 263.90 273.18 278.06
283.48 288.11 292.30 296.64 299.05 303.48
321.98 323.66 326.64 338.30 341.29 349.48
352.17 375.17 392.11 393.89 409.79 417.81
418.80 433.51 448.99 456.13 458.80 461.59
470.65 480.69 489.05 494.69 506.70 526.04
537.79 538.24 546.25 549.00 559.94 584.72
602.48 607.03 609.79 621.15 627.22 641.89
653.77 657.02 664.83 671.17 680.83 691.60
701.76 709.34 719.19 721.92 731.07 765.96
774.94 780.77 795.36 811.65 820.96 826.82
832.16 856.02 858.30 872.94 887.64 892.45
901.55 904.88 915.81 919.44 947.17 954.93
957.79 959.51 976.47 983.17 987.03 988.88
990.07 994.92 1012.60 1016.99 1019.76 1020.36
1024.52 1031.68 1032.48 1034.81 1052.05 1053.22
1061.37 1063.47 1064.90 1068.98 1084.63 1107.30
1119.11 1131.96 1138.42 1140.46 1154.54 1158.16
1172.40 1190.54 1192.51 1201.43 1221.59 1225.72
1226.62 1227.27 1235.56 1236.70 1237.29 1250.72
1261.69 1285.93 1292.18 1295.48 1318.10 1326.03
1329.31 1345.25 1350.81 1352.89 1355.07 1368.23

1387.61 1393.01 1400.15 1414.65 1421.34 1422.58
1423.58 1428.81 1444.18 1452.61 1462.79 1465.97
1474.26 1484.57 1490.24 1492.27 1493.73 1494.91
1496.56 1498.62 1500.80 1504.01 1505.30 1508.02
1511.96 1515.60 1519.00 1537.12 1537.29 1545.46
1557.20 1580.54 1624.85 1631.92 1652.99 1653.42
1658.42 1808.09 3044.54 3046.91 3052.94 3055.16
3058.88 3059.85 3065.89 3105.12 3116.20 3125.89
3126.62 3131.87 3132.12 3141.95 3143.49 3143.65
3147.00 3164.48 3170.52 3184.75 3185.20 3189.56
3197.09 3197.35 3209.68 3213.97 3214.16 3228.63
3231.91 3238.55 3242.30 3256.80 3257.97 3474.14
==============================
^{S}A5
==============================
10.53 13.92 25.61 31.53 33.21 38.33
42.05 44.69 51.48 57.37 66.22 67.69
73.15 82.43 85.76 89.16 91.72 111.61
123.13 132.04 139.67 144.99 160.36 171.48
183.79 188.85 202.26 208.36 211.58 228.45
229.32 236.58 241.28 258.57 260.66 264.29
271.12 275.43 279.35 280.40 291.36 300.63
309.50 315.84 335.45 341.55 342.68 345.46
383.14 394.73 407.79 408.36 414.47 415.45
420.91 431.39 437.16 450.30 467.77 475.99
477.67 486.84 494.28 540.38 544.99 546.50
556.90 565.85 586.79 608.94 610.93 622.53
645.31 646.47 654.09 658.09 691.08 709.38
718.04 722.66 751.41 762.36 770.20 772.97
773.98 780.90 803.27 820.20 828.22 838.57
845.04 853.74 856.35 859.34 870.51 881.45
903.15 920.57 931.99 938.48 943.74 956.53
962.40 966.45 972.61 974.21 981.71 981.89
989.36 991.62 995.19 1004.94 1009.96 1010.78
1017.57 1019.40 1022.10 1024.43 1031.86 1039.19
1044.58 1054.67 1063.32 1064.28 1064.65 1065.95
1074.75 1080.36 1121.43 1124.18 1133.84 1140.80
1155.57 1176.24 1193.85 1199.05 1204.41 1219.26
1223.88 1226.84 1229.61 1236.67 1239.14 1247.32
1251.57 1261.50 1265.67 1295.01 1304.64 1314.95
1329.78 1335.69 1340.36 1340.99 1349.40 1351.65
1354.94 1356.86 1376.14 1401.79 1410.45 1418.14
1423.47 1424.88 1429.08 1434.67 1435.07 1445.07
1450.28 1456.05 1464.10 1486.77 1490.50 1492.11
1497.92 1500.17 1500.57 1500.80 1501.80 1503.85
1504.45 1508.39 1511.13 1514.09 1514.92 1517.28
1519.58 1522.24 1536.73 1536.87 1551.67 1583.08
1591.27 1627.15 1631.96 1645.83 1652.17 1652.73
1659.35 3018.92 3026.27 3040.90 3043.04 3048.96
3050.84 3053.76 3055.52 3058.26 3062.50 3078.60
3106.63 3110.21 3117.92 3120.43 3127.03 3130.86
3132.01 3132.86 3134.60 3138.54 3142.15 3145.71
3153.90 3157.03 3188.30 3189.17 3190.59 3198.42
3201.05 3204.51 3209.41 3218.16 3218.45 3218.49
3221.75 3226.71 3234.18 3250.99 3252.69 3260.28
==============================
^{R}A5
==============================
14.58 17.24 26.78 31.55 33.26 38.19
44.06 52.13 59.78 61.61 69.32 71.92
75.43 82.92 86.87 87.85 99.26 102.54
113.77 131.43 145.71 148.51 161.07 174.71
182.67 186.98 197.80 199.16 211.40 225.72
230.08 233.38 243.50 244.87 257.71 260.99
269.38 273.83 275.43 282.29 296.18 305.80
313.99 315.03 334.30 336.50 342.59 344.76
379.90 393.84 394.21 408.21 413.94 415.45
418.87 433.03 437.01 453.76 476.30 478.22
484.09 493.55 494.16 528.56 541.41 545.61
558.13 569.55 587.48 610.09 613.63 625.85
641.19 647.24 650.10 659.74 707.25 709.64
714.36 725.31 752.56 758.96 767.57 772.85
777.62 778.79 797.10 810.65 830.30 840.12
841.31 855.66 856.37 857.86 859.55 876.65
890.49 923.31 935.57 940.70 942.09 945.91
958.28 961.69 974.97 977.55 985.70 986.65
989.54 991.18 993.31 1007.85 1011.92 1013.43
1018.64 1020.48 1021.06 1023.12 1030.69 1036.04
1046.15 1055.81 1063.03 1064.00 1064.28 1065.41
1069.60 1081.55 1120.88 1121.32 1132.83 1144.90
1154.34 1176.44 1191.22 1196.04 1202.04 1220.13
1222.72 1227.25 1229.62 1231.28 1235.18 1245.50
1253.93 1261.04 1266.98 1298.68 1312.78 1330.21
1332.54 1337.16 1339.14 1340.52 1341.21 1355.62
1355.91 1356.57 1366.71 1404.85 1410.24 1418.24
1423.92 1424.32 1428.76 1433.62 1435.13 1443.47
1455.13 1456.21 1466.70 1486.61 1490.33 1494.37
1495.85 1499.63 1499.84 1501.59 1502.23 1502.64
1504.18 1505.30 1508.51 1514.17 1515.05 1515.17
1519.47 1522.23 1535.94 1537.28 1546.58 1584.88
1596.70 1626.50 1633.46 1646.00 1651.06 1651.37
1660.01 3018.15 3026.41 3041.33 3043.32 3048.74
3051.84 3052.16 3054.18 3058.41 3068.70 3080.57
3106.21 3110.78 3114.48 3120.59 3121.01 3124.24
3129.49 3134.66 3135.13 3144.16 3146.21 3148.21
3160.80 3163.92 3183.88 3186.31 3188.10 3189.23
3193.55 3200.18 3203.92 3214.59 3215.19 3217.35
3222.62 3223.16 3237.47 3250.75 3252.74 3264.86
==============================
^{S}A5-TS
==============================
-178.30 15.27 23.16 29.47 33.23 37.14
41.48 46.37 49.61 54.88 59.60 66.63
72.32 73.19 80.01 85.36 99.73 105.72
113.21 119.30 136.01 143.72 155.94 156.40
170.72 174.09 181.41 198.91 206.79 212.68
220.43 233.86 243.32 248.12 256.20 259.53
273.03 277.37 278.51 289.22 298.83 306.23
313.13 314.59 334.69 339.35 342.69 353.91
390.40 402.45 408.41 411.13 417.71 419.85
434.24 438.27 447.17 461.97 464.56 468.30
481.24 485.68 492.12 496.95 535.40 550.44
552.56 557.19 582.67 599.78 604.15 627.68
637.24 647.56 649.32 656.84 688.48 713.83
718.08 727.49 760.41 763.70 772.27 775.35
778.56 785.32 812.44 820.08 832.47 837.28
851.35 859.61 869.81 874.34 880.33 885.45
897.48 917.67 936.00 947.66 953.74 954.41
959.77 961.07 971.45 973.69 977.25 980.44
985.05 987.38 988.96 991.80 1015.28 1015.51
1017.82 1022.09 1023.62 1031.41 1036.96 1037.54
1052.24 1058.50 1063.03 1064.62 1065.95 1068.57
1073.90 1084.82 1108.25 1122.29 1135.05 1139.42
1154.47 1175.43 1189.58 1203.69 1203.91 1216.89
1223.15 1226.24 1228.27 1232.77 1235.94 1240.20
1246.09 1254.99 1271.47 1288.54 1312.00 1317.12
1321.01 1327.70 1336.44 1340.46 1346.18 1350.99
1354.33 1373.18 1380.33 1386.27 1386.83 1419.45
1421.13 1424.80 1427.37 1433.47 1437.21 1439.77
1443.08 1454.74 1462.67 1484.25 1491.97 1494.89
1497.78 1498.34 1499.22 1499.92 1500.66 1501.80
1503.95 1508.20 1513.39 1513.55 1514.82 1516.47
1519.47 1522.53 1535.56 1536.08 1543.69 1550.84
1587.84 1623.99 1632.98 1644.87 1651.96 1659.28
1665.50 3032.87 3035.62 3042.16 3043.58 3048.90
3050.32 3051.48 3057.55 3062.21 3076.07 3085.51
3108.18 3111.15 3115.37 3120.13 3122.98 3124.72
3131.08 3134.33 3136.74 3137.50 3140.00 3143.64
3144.05 3150.10 3186.75 3188.26 3188.84 3189.64
3195.57 3198.35 3205.27 3213.76 3214.41 3215.52
3218.46 3222.01 3223.93 3235.10 3241.47 3263.34
==============================
^{R}A5-TS
==============================
-184.00 15.56 25.40 27.31 33.55 35.42
38.43 41.41 49.16 49.87 54.31 65.93
67.51 75.81 86.71 91.23 96.04 108.30
116.40 127.05 139.41 146.25 152.66 159.67
170.95 173.99 179.45 185.37 198.80 211.35
222.32 230.89 232.12 238.81 249.96 259.17

275.83 278.18 280.24 289.58 299.05 305.21
312.73 316.70 334.13 335.31 340.55 348.37
382.80 392.80 404.30 410.65 415.95 421.61
438.64 442.36 450.13 464.72 467.95 471.78
482.36 485.51 497.42 499.36 539.61 544.95
551.97 557.78 582.14 601.84 604.79 628.41
642.44 647.81 651.06 653.88 690.15 711.54
718.89 731.02 756.16 761.89 769.89 772.96
779.67 784.34 812.32 819.55 831.75 832.92
846.76 860.01 863.32 875.73 880.83 884.12
899.31 918.45 931.29 946.09 946.90 952.21
961.33 961.51 968.44 976.12 979.79 980.29
983.89 987.41 988.10 988.75 1011.56 1014.31
1016.21 1020.08 1023.78 1028.11 1033.95 1035.83
1050.89 1058.14 1062.82 1063.63 1064.65 1070.05
1074.59 1086.01 1107.37 1120.95 1135.45 1139.88
1154.05 1175.62 1188.09 1202.06 1203.77 1219.27
1222.59 1225.51 1228.46 1231.40 1235.64 1240.28
1242.20 1255.32 1271.48 1287.44 1319.20 1321.28
1324.73 1327.60 1334.44 1340.76 1344.74 1353.78
1355.19 1367.49 1379.86 1386.24 1386.40 1419.37
1421.28 1424.68 1425.19 1428.36 1435.04 1438.18
1443.40 1455.12 1471.39 1484.84 1491.85 1494.20
1496.58 1497.65 1498.32 1498.73 1499.54 1501.65
1504.14 1504.31 1513.16 1514.34 1515.41 1518.95
1519.61 1522.54 1535.87 1536.41 1542.85 1547.58
1585.67 1624.46 1634.68 1644.56 1652.15 1660.27
1669.94 3032.16 3034.03 3043.92 3045.89 3049.27
3051.05 3053.79 3058.81 3064.56 3075.85 3084.05
3107.33 3113.25 3120.00 3121.12 3123.15 3125.62
3133.09 3134.02 3136.14 3139.52 3142.34 3143.98
3147.07 3163.37 3181.03 3185.12 3188.87 3189.65
3195.01 3199.11 3205.69 3212.41 3214.41 3215.08
3221.32 3221.69 3222.27 3232.59 3246.84 3250.18

==============================

A5-TS^{H}

==============================

-171.66 9.00 20.41 29.77 33.39 40.50
43.32 47.00 55.05 58.14 62.24 68.56
86.75 90.64 105.38 110.44 121.78 136.53
146.41 156.54 158.33 172.62 174.40 183.77
194.00 211.41 219.07 227.81 231.63 250.17
256.32 261.82 276.30 282.51 289.71 297.99
304.96 311.35 314.40 336.45 339.25 345.95
369.77 391.42 401.34 410.41 412.01 417.45
439.44 443.16 447.68 463.65 465.12 469.61
481.99 486.55 494.79 532.72 536.40 552.91
582.30 592.82 602.24 606.00 642.22 646.50
652.16 711.68 722.92 760.77 764.88 770.85
774.39 786.22 795.16 815.25 820.92 833.64
851.40 860.54 871.13 877.49 884.05 885.39
890.50 896.77 939.43 947.28 952.06 957.44
960.66 962.18 974.57 977.61 978.63 985.34
988.44 989.12 1009.20 1016.06 1021.63 1026.31
1030.72 1035.42 1050.55 1059.40 1062.97 1064.17
1070.18 1073.86 1085.62 1106.98 1139.17 1140.82
1154.29 1160.05 1197.48 1198.99 1204.41 1222.87
1229.56 1230.78 1232.26 1235.94 1242.40 1254.92
1272.36 1284.81 1287.46 1317.08 1321.34 1335.01
1340.53 1344.85 1349.00 1354.41 1379.43 1385.31
1386.15 1392.80 1419.24 1423.90 1425.44 1428.91
1434.98 1437.67 1443.20 1455.18 1474.04 1492.12
1493.50 1497.31 1498.26 1499.10 1499.29 1499.91
1501.34 1503.29 1511.17 1513.32 1515.24 1516.38
1516.81 1519.51 1522.06 1535.49 1536.18 1545.57
1591.09 1624.35 1645.67 1651.17 1658.80 3033.53
3036.50 3043.40 3045.21 3049.53 3051.15 3051.67
3060.34 3063.06 3075.81 3086.34 3107.85 3111.70
3117.21 3121.89 3123.77 3126.00 3132.88 3134.71
3136.69 3139.39 3141.67 3143.39 3143.84 3146.30
3186.64 3188.46 3190.17 3197.84 3213.38 3214.77
3219.94 3222.87 3237.31 3243.81 3250.53 3270.01

==============================

^{S}A6

==============================

16.40 28.69 32.28 34.52 35.60 43.13
46.59 57.85 60.87 63.50 66.68 70.20
70.58 79.01 85.29 102.32 118.23 124.55
131.71 145.67 156.29 164.76 173.75 176.91
183.90 198.74 202.90 216.31 225.53 227.33
236.47 241.36 248.48 254.50 261.52 274.60
277.98 278.55 290.48 294.36 300.48 312.67
325.99 340.72 343.81 347.96 359.54 395.09
397.89 406.77 409.19 416.26 418.88 429.84
437.80 455.03 459.10 476.47 481.87 486.19
500.12 517.70 535.88 557.40 559.20 567.84
589.50 613.93 615.19 623.04 630.09 644.48
658.89 659.36 666.77 708.59 711.00 717.74
726.26 746.97 761.21 763.08 774.66 782.27
789.82 807.96 815.90 828.13 833.82 847.35
856.48 861.09 868.28 885.48 885.91 911.74
913.17 924.45 928.58 943.54 952.15 953.54
961.53 970.49 978.57 981.05 986.52 989.13
990.25 990.96 1009.51 1013.23 1013.85 1016.99
1023.82 1027.47 1030.69 1046.75 1052.42 1056.62
1060.81 1062.66 1064.91 1067.09 1074.46 1087.94
1092.44 1115.34 1121.64 1132.43 1146.99 1151.15
1157.79 1159.95 1173.30 1191.41 1195.03 1202.73
1216.44 1225.39 1226.52 1227.42 1238.33 1242.01
1251.51 1256.16 1291.24 1302.26 1307.58 1321.81
1326.03 1339.50 1342.19 1348.56 1351.03 1362.98
1364.47 1371.79 1375.51 1384.75 1391.08 1412.96
1419.41 1424.48 1425.47 1426.05 1436.59 1439.09
1446.11 1454.79 1464.28 1478.67 1487.23 1490.81
1496.35 1497.88 1498.36 1499.71 1501.02 1502.08
1502.60 1504.06 1507.81 1513.90 1514.78 1515.47
1515.97 1519.45 1521.51 1533.12 1535.86 1551.51
1610.65 1621.72 1633.92 1647.47 1647.79 1660.64
1736.58 3016.71 3033.03 3036.89 3045.82 3050.43
3052.62 3054.46 3055.08 3059.86 3066.34 3074.67
3077.17 3086.49 3105.48 3112.02 3115.71 3116.64
3127.78 3128.62 3132.36 3134.19 3139.83 3141.63
3144.26 3146.36 3148.96 3171.29 3188.17 3195.01
3195.18 3195.78 3196.20 3205.06 3208.34 3211.18
3216.93 3219.16 3224.01 3226.95 3229.54 3250.21

==============================

^{R}A6

==============================

18.58 25.92 33.00 34.82 38.18 41.24
43.60 49.06 58.89 65.73 69.04 73.58
81.89 91.23 93.61 102.88 119.30 123.94
131.72 137.71 145.81 155.64 168.85 172.84
178.27 192.65 198.74 207.64 225.91 226.51
230.79 237.51 241.94 244.53 260.76 277.66
278.17 281.97 290.78 295.69 301.17 309.92
323.88 337.77 339.57 348.64 357.33 386.66
396.62 398.54 407.32 416.29 425.32 427.52
440.70 454.14 464.60 477.96 481.53 484.74
506.01 516.68 534.90 552.03 557.65 566.36
587.60 604.32 613.59 623.01 630.83 639.21
649.49 658.27 660.71 691.10 707.64 723.13
723.89 749.71 760.03 762.48 774.92 783.75
789.53 807.05 811.31 830.43 831.49 835.74
852.05 858.92 869.70 884.68 885.83 908.77
911.05 918.86 928.65 943.15 950.86 952.72
961.61 973.33 978.96 981.02 986.60 989.46
991.15 994.15 1009.82 1011.13 1011.99 1015.58
1021.29 1025.13 1030.83 1043.40 1053.25 1054.26
1061.22 1063.13 1063.89 1064.86 1071.79 1086.54
1090.26 1112.06 1120.75 1127.90 1147.95 1151.50
1157.27 1158.88 1174.82 1188.42 1194.20 1201.33
1216.13 1223.73 1225.90 1227.12 1236.40 1239.69
1250.75 1255.72 1289.86 1301.84 1313.28 1323.20
1323.79 1339.59 1342.47 1349.70 1353.23 1365.27
1367.52 1368.27 1377.20 1378.05 1391.21 1411.29
1419.91 1424.52 1425.55 1426.48 1426.61 1436.27
1446.10 1454.85 1467.77 1481.37 1485.47 1490.87
1494.90 1495.30 1498.82 1500.00 1501.04 1501.21
1502.39 1503.95 1504.34 1514.04 1514.99 1515.35
1517.16 1519.67 1521.74 1532.70 1536.06 1547.12

1604.16 1621.31 1634.27 1646.43 1648.02 1660.44
1736.93 3014.89 3032.65 3036.80 3048.15 3053.40
3053.91 3055.06 3057.15 3060.42 3066.33 3074.46
3076.25 3090.97 3105.80 3110.94 3117.71 3120.45
3128.40 3133.43 3133.57 3134.63 3141.02 3141.42
3144.81 3148.41 3165.79 3169.09 3186.31 3193.08
3194.08 3195.19 3196.26 3205.18 3205.95 3214.44
3215.85 3220.00 3227.44 3227.46 3237.89 3239.13
==============================
^{S}A6-TS
==============================
-26.89 12.15 22.43 27.60 28.61 37.03
42.11 46.65 50.27 63.94 68.29 73.28
75.00 78.22 87.42 99.20 101.54 109.56
117.40 126.12 139.38 141.81 150.01 157.38
170.71 173.48 179.98 188.67 201.01 219.15
224.60 234.56 240.68 243.39 252.42 271.06
276.29 278.39 281.33 288.99 291.08 309.65
316.97 327.21 331.92 340.47 348.43 379.84
392.16 398.81 407.33 419.08 421.05 428.50
435.32 447.24 450.97 453.10 472.80 479.90
498.03 506.15 516.70 541.90 548.49 550.04
556.41 569.91 607.01 616.52 627.86 637.65
641.39 650.93 655.57 657.96 696.64 708.41
718.70 740.43 750.91 760.00 768.28 778.43
780.00 800.01 804.72 807.29 828.76 831.78
854.46 856.18 867.93 878.40 879.83 883.10
897.42 903.90 925.16 945.10 948.49 952.83
956.41 973.06 976.01 980.27 982.83 988.26
992.29 992.72 1008.63 1010.66 1015.08 1016.53
1023.20 1025.10 1028.50 1045.57 1047.33 1053.23
1055.95 1062.22 1063.15 1064.37 1065.10 1083.01
1084.87 1104.53 1122.59 1136.32 1139.94 1143.93
1160.57 1174.75 1182.96 1189.18 1203.26 1219.44
1225.70 1226.89 1227.72 1236.72 1237.91 1243.58
1247.28 1265.28 1265.70 1286.80 1303.11 1304.09
1309.06 1326.02 1339.08 1343.96 1346.70 1350.98
1351.39 1361.79 1371.49 1387.76 1389.35 1408.47
1412.86 1421.69 1425.61 1425.85 1436.74 1437.96
1446.85 1450.37 1459.31 1464.69 1481.92 1488.60
1494.52 1495.45 1498.36 1498.66 1499.28 1502.77
1503.35 1504.52 1509.40 1512.80 1513.27 1514.44
1516.02 1516.34 1522.74 1532.23 1536.85 1546.42
1559.66 1602.58 1620.68 1630.81 1639.27 1647.45
1657.36 3012.48 3015.75 3030.09 3036.08 3043.87
3044.26 3047.91 3053.66 3054.32 3056.62 3073.69
3079.15 3105.19 3105.30 3109.68 3115.15 3116.95
3117.57 3120.92 3126.12 3131.78 3138.91 3140.45
3153.23 3155.55 3169.65 3190.12 3193.05 3194.01
3195.85 3196.78 3201.28 3206.50 3213.39 3215.27
3222.44 3223.88 3225.29 3230.35 3235.24 3242.66
==============================
^{R}A6-TS
==============================
-20.54 5.96 8.11 16.56 25.65 29.36
32.93 33.82 44.68 46.53 54.25 70.63
72.96 76.83 83.37 93.18 111.42 116.68
121.06 125.93 139.70 142.63 154.07 164.73
169.20 170.64 178.24 189.68 214.43 217.94
233.33 233.74 235.89 244.84 255.63 262.88
273.72 278.20 287.35 294.13 296.67 313.69
316.74 321.56 332.40 337.38 348.62 358.20
386.90 393.77 406.39 415.40 417.98 428.10
436.06 443.38 448.19 456.99 474.27 482.69
503.55 510.25 516.12 543.14 551.23 552.56
559.38 569.06 605.67 614.45 621.43 638.94
639.46 648.68 654.49 659.19 697.15 705.23
711.49 748.51 755.30 764.47 772.79 777.52
780.75 801.73 805.23 813.77 828.88 831.83
850.87 853.29 859.49 874.37 880.24 885.32
892.81 895.47 920.72 946.30 946.76 950.41
955.38 974.82 978.56 982.37 984.92 985.70
989.91 992.29 1003.81 1011.57 1012.24 1016.00
1017.90 1024.91 1030.69 1044.07 1044.89 1051.91
1055.91 1059.52 1059.99 1064.88 1067.63 1081.03
1085.98 1114.30 1123.48 1134.63 1137.07 1141.35
1159.50 1176.87 1178.83 1187.12 1203.94 1220.38
1226.58 1226.70 1229.92 1235.96 1237.69 1238.77
1240.91 1261.36 1263.21 1272.82 1302.91 1314.90
1320.22 1325.43 1331.54 1345.04 1349.16 1350.88
1353.78 1358.94 1370.84 1388.15 1389.23 1408.71
1412.32 1413.75 1418.14 1427.67 1428.37 1436.79
1445.66 1448.15 1468.04 1470.49 1481.75 1488.43
1493.61 1494.91 1497.25 1497.61 1498.33 1502.47
1504.66 1505.46 1506.04 1511.38 1513.57 1515.68
1517.68 1522.16 1523.00 1532.60 1535.56 1539.50
1587.48 1601.98 1619.70 1624.56 1642.87 1646.64
1652.87 3012.03 3015.33 3028.56 3037.36 3042.72
3045.37 3046.75 3053.90 3054.05 3056.03 3072.74
3091.94 3105.32 3105.85 3108.11 3114.13 3117.80
3117.91 3122.56 3126.08 3136.40 3138.87 3139.56
3141.39 3146.99 3162.87 3191.59 3193.84 3194.77
3194.83 3196.37 3200.34 3209.41 3211.34 3215.49
3217.90 3221.44 3221.86 3242.26 3244.86 3249.19
==============================
^{S}A7
==============================
12.68 16.62 19.61 26.81 29.30 35.43
36.79 39.57 43.29 50.36 57.13 61.80
72.21 78.57 91.53 105.03 117.34 117.60
123.90 133.90 136.54 140.87 156.83 174.38
181.77 188.20 198.10 206.30 219.08 228.82
241.59 248.28 262.80 263.45 268.77 269.54
277.17 282.10 283.25 292.57 297.92 299.29
314.19 324.84 334.85 348.27 353.38 367.28
381.63 391.43 401.91 412.74 417.25 421.38
431.76 439.12 454.81 466.97 479.77 484.26
493.69 504.43 507.77 540.04 550.53 557.91
564.55 580.86 610.73 612.25 617.01 624.86
641.22 646.11 658.64 661.23 708.86 711.10
719.03 730.64 757.67 775.90 778.55 784.98
789.61 801.81 811.57 815.56 823.49 829.20
856.42 861.88 876.47 886.18 888.20 911.68
912.66 926.63 950.72 952.64 954.56 958.82
965.09 975.48 985.83 986.73 987.07 988.74
992.46 1002.59 1016.13 1021.93 1022.20 1026.01
1031.34 1032.49 1036.35 1037.42 1054.83 1055.65
1056.26 1060.30 1063.93 1065.44 1073.52 1079.12
1091.57 1125.77 1126.76 1134.83 1141.22 1153.09
1156.84 1183.40 1186.75 1191.75 1206.30 1224.71
1225.06 1231.03 1234.42 1236.17 1246.29 1247.53
1254.97 1258.66 1282.49 1291.43 1321.93 1325.63
1329.17 1336.19 1342.98 1355.96 1356.79 1363.04
1377.38 1387.69 1396.92 1399.77 1409.59 1410.34
1420.17 1427.10 1427.71 1428.92 1436.06 1437.21
1446.04 1461.17 1469.10 1477.69 1481.93 1491.43
1497.68 1498.40 1499.33 1499.54 1500.49 1504.41
1504.94 1507.60 1511.05 1513.75 1515.41 1516.24
1517.79 1518.32 1522.40 1523.48 1534.72 1544.59
1548.18 1590.86 1620.91 1628.54 1637.74 1648.58
1655.11 3011.26 3028.46 3038.23 3041.54 3043.80
3044.56 3050.08 3050.62 3051.97 3055.02 3059.26
3071.01 3106.40 3108.11 3115.58 3120.21 3121.60
3122.53 3123.60 3126.88 3130.36 3136.97 3139.27
3141.45 3143.93 3164.72 3190.34 3192.05 3194.00
3200.97 3202.59 3208.30 3214.20 3215.46 3217.21
3222.17 3222.28 3230.72 3233.24 3238.05 3283.10
==============================
^{R}A7
==============================
15.04 25.16 26.35 33.14 36.71 37.97
41.32 49.11 50.67 52.84 65.46 72.03
82.58 83.67 92.05 101.93 111.16 120.78
122.29 129.74 141.88 149.40 170.37 178.44
181.21 185.04 186.91 197.45 214.87 228.29
239.04 242.08 252.55 269.00 269.76 274.73
280.13 284.42 285.97 288.64 300.08 305.63
314.92 318.17 328.08 343.01 348.25 374.57
383.95 390.82 403.38 412.70 415.78 420.80
427.00 434.46 453.13 458.09 475.43 479.50

486.52 503.02 512.89 541.67 549.38 555.52
559.90 572.95 599.75 610.91 617.54 625.11
642.94 644.87 659.80 662.75 707.57 710.23
713.35 728.56 760.71 776.93 783.29 784.04
788.42 797.67 811.30 816.51 826.97 831.24
858.19 864.12 866.30 884.30 889.47 912.01
918.65 922.50 948.20 959.77 962.95 963.34
971.33 978.23 984.29 984.56 986.78 987.01
996.33 1000.96 1015.31 1020.11 1020.37 1022.52
1026.46 1029.50 1032.99 1043.58 1044.54 1055.31
1058.08 1059.31 1063.75 1067.22 1081.36 1086.34
1103.67 1119.40 1130.90 1136.63 1137.86 1148.85
1155.32 1179.05 1192.78 1198.65 1206.55 1220.36
1224.03 1230.75 1233.41 1236.93 1250.77 1251.91
1254.11 1262.50 1286.67 1294.88 1312.49 1325.80
1327.07 1332.65 1342.03 1355.73 1357.11 1357.71
1380.99 1387.89 1393.02 1396.21 1411.31 1411.90
1418.43 1427.66 1428.54 1430.80 1435.19 1437.49
1445.81 1457.04 1462.31 1467.60 1485.62 1489.99
1490.30 1496.76 1497.13 1498.89 1500.75 1504.87
1504.96 1505.40 1509.76 1512.23 1515.30 1516.89
1516.99 1517.74 1522.02 1525.22 1535.53 1545.64
1548.67 1601.36 1621.65 1621.87 1638.69 1649.07
1653.29 3013.32 3024.42 3039.24 3045.34 3046.37
3047.23 3051.63 3052.37 3056.03 3056.39 3063.49
3070.71 3106.63 3108.30 3117.41 3119.76 3122.87
3126.23 3127.65 3130.69 3135.38 3136.39 3136.97
3137.62 3144.87 3188.55 3189.21 3189.39 3195.27
3202.91 3205.80 3211.50 3213.52 3215.60 3219.41
3222.47 3227.30 3232.92 3240.52 3247.48 3260.60

==============================

^{S}A7-TS

==============================

-271.05 15.71 20.61 22.34 24.42 24.76
29.48 39.60 41.32 44.44 59.94 65.60
67.11 80.27 84.25 94.84 105.93 114.55
122.45 128.22 137.04 147.74 153.88 157.36
174.48 179.74 182.68 196.47 208.68 213.01
226.96 237.68 247.93 251.66 266.75 270.19
274.18 280.83 288.97 297.62 299.64 303.27
311.94 315.84 317.91 333.35 347.96 353.01
368.88 381.23 392.86 399.95 412.28 414.82
421.37 426.43 437.36 460.50 464.09 477.55
480.38 490.46 491.91 510.77 533.98 542.43
554.63 558.52 577.82 607.42 612.22 622.44
642.48 643.78 654.15 655.93 703.59 708.82
711.51 722.95 723.57 761.55 776.37 781.75
790.59 797.61 812.31 815.35 818.50 826.71
855.75 856.01 872.89 881.13 882.24 889.69
911.40 923.21 931.65 945.41 949.27 958.76
965.44 974.22 983.32 987.26 987.35 989.34
992.76 999.79 1003.62 1015.45 1016.25 1021.84
1022.47 1022.55 1030.24 1030.78 1031.10 1048.87
1055.69 1057.35 1059.66 1064.14 1066.49 1083.22
1087.16 1124.39 1124.95 1136.25 1143.68 1157.70
1185.40 1190.22 1193.84 1198.60 1205.10 1224.18
1225.70 1230.29 1234.64 1236.32 1245.70 1253.36
1258.21 1260.27 1283.07 1302.01 1312.94 1329.97
1330.98 1331.69 1343.34 1344.26 1355.68 1358.03
1376.59 1389.81 1398.40 1401.43 1409.31 1412.97
1420.57 1428.92 1430.59 1433.13 1436.16 1436.74
1445.88 1457.44 1462.32 1464.68 1486.00 1490.89
1496.80 1497.67 1498.44 1499.03 1502.36 1503.95
1504.94 1506.17 1509.95 1513.20 1514.38 1515.81
1516.93 1520.21 1523.44 1534.61 1535.12 1546.62
1553.39 1579.46 1622.72 1630.33 1647.60 1648.99
1656.87 3010.13 3028.29 3038.63 3044.13 3044.70
3049.12 3050.86 3052.16 3055.39 3072.02 3077.69
3087.82 3107.30 3108.82 3118.24 3119.50 3122.31
3124.26 3129.40 3132.03 3135.62 3137.05 3138.02
3156.26 3162.58 3171.82 3191.03 3192.31 3193.30
3199.27 3201.88 3206.79 3212.35 3213.09 3218.15
3219.57 3220.34 3228.12 3231.36 3285.97 3307.81

==============================

^{R}A7-TS

==============================

-207.82 7.58 18.05 24.12 31.81 34.16
39.90 44.66 49.81 52.23 54.64 66.28
77.53 87.75 91.85 98.73 103.14 119.66
123.09 126.11 136.53 148.00 148.94 164.54
171.13 178.90 181.71 183.51 193.16 213.10
220.22 227.64 238.15 254.31 257.52 266.54
272.40 278.41 284.77 288.07 291.77 298.29
306.00 316.15 323.89 336.58 339.40 358.51
367.61 374.58 393.13 411.71 416.52 418.12
425.81 436.27 444.27 462.55 464.45 483.74
486.40 492.05 496.30 520.32 533.37 546.42
555.17 557.03 582.70 608.26 621.67 628.06
639.39 642.56 653.28 655.85 703.75 709.64
719.08 721.24 725.72 764.25 766.76 780.72
786.62 788.32 806.18 811.74 818.69 829.60
858.19 858.92 866.35 876.53 883.78 900.30
908.54 920.59 940.77 948.07 953.12 958.77
960.77 975.90 978.12 985.06 986.44 990.48
993.40 998.34 1006.04 1014.47 1015.62 1017.76
1020.66 1021.91 1029.23 1030.67 1041.25 1055.97
1058.05 1058.61 1062.96 1063.74 1065.15 1078.86
1088.05 1124.90 1126.20 1131.36 1142.50 1156.07
1179.76 1184.88 1192.57 1200.83 1205.14 1222.54
1224.15 1232.50 1232.91 1233.81 1242.15 1245.08
1259.83 1262.71 1284.67 1303.05 1319.07 1323.16
1326.12 1329.31 1342.48 1346.08 1356.09 1357.68
1372.32 1388.49 1391.83 1398.04 1403.48 1413.38
1417.40 1418.22 1422.30 1428.11 1428.65 1436.09
1444.59 1451.58 1457.69 1465.55 1486.34 1490.44
1494.67 1496.88 1498.77 1499.46 1499.70 1501.77
1503.61 1505.10 1507.26 1512.97 1515.46 1516.82
1520.36 1520.84 1523.44 1533.45 1534.28 1543.34
1577.13 1592.88 1622.17 1626.38 1642.13 1648.90
1653.77 3009.93 3023.51 3038.65 3045.67 3046.10
3050.94 3051.70 3054.97 3057.91 3070.77 3073.56
3079.83 3107.18 3108.79 3118.65 3118.82 3125.68
3128.42 3128.98 3129.86 3134.30 3136.79 3139.06
3142.72 3151.21 3172.03 3189.75 3192.92 3196.63
3204.49 3206.72 3214.77 3217.15 3219.85 3220.67
3226.82 3229.21 3231.97 3252.57 3260.29 3308.13

==============================

^{S}A8

==============================

11.51 19.10 26.12 31.90 38.87 43.78
54.07 60.95 66.49 71.22 73.46 80.21
80.97 82.60 90.58 108.13 113.45 120.72
137.15 142.10 147.49 158.27 167.79 172.24
177.67 189.37 196.82 206.02 214.28 222.30
237.77 238.83 242.20 262.57 265.69 278.56
280.61 288.14 293.28 296.96 301.38 304.47
317.98 322.65 330.27 340.23 345.32 361.58
379.54 393.51 396.68 413.67 414.25 417.52
424.41 440.71 443.52 464.24 473.73 482.91
484.61 494.75 513.44 537.54 541.79 549.86
560.26 564.89 608.89 614.94 624.58 639.40
643.29 646.50 659.02 664.80 704.78 708.21
724.96 725.98 761.54 775.57 781.90 791.36
797.00 807.83 816.62 821.24 822.18 846.54
853.12 865.70 872.47 881.92 886.66 891.40
918.10 922.57 923.74 952.04 960.90 964.44
969.21 974.54 984.22 984.74 987.39 989.37
989.88 1005.17 1015.32 1019.83 1022.61 1023.09
1027.72 1028.93 1033.44 1045.42 1053.89 1057.70
1062.20 1066.04 1066.33 1084.18 1086.04 1111.74
1121.85 1123.72 1139.95 1141.52 1160.28 1166.99
1179.08 1186.19 1190.32 1204.31 1207.02 1221.00
1227.14 1229.01 1236.35 1237.20 1243.99 1255.59
1256.92 1263.26 1275.97 1299.65 1323.76 1327.21
1328.31 1330.92 1343.22 1345.11 1352.63 1361.19
1375.36 1389.91 1393.25 1404.00 1410.07 1416.12
1422.66 1427.94 1432.57 1436.67 1439.34 1439.69
1446.22 1460.15 1481.84 1487.08 1490.62 1493.35
1496.39 1497.36 1499.55 1501.02 1501.43 1503.94
1504.67 1507.54 1509.95 1511.50 1514.58 1516.56

1517.55 1518.45 1523.01 1534.17 1538.52 1543.76
1550.37 1580.62 1626.20 1630.93 1649.24 1650.21
1657.79 3023.04 3023.89 3041.62 3042.38 3044.07
3046.64 3054.48 3055.15 3062.85 3073.04 3096.17
3108.02 3112.36 3116.31 3119.83 3121.14 3129.36
3132.25 3133.67 3139.33 3140.18 3141.14 3147.25
3148.22 3171.92 3179.45 3189.61 3192.41 3193.70
3196.11 3200.56 3204.12 3209.35 3211.50 3217.54
3219.89 3222.15 3229.76 3233.64 3249.34 3271.11

==============================

^{R}A8

==============================

17.18 23.59 24.25 35.74 39.69 43.85
48.67 50.48 54.77 63.87 73.23 79.20
86.58 87.47 94.21 101.22 109.83 115.28
134.37 139.76 146.77 151.16 156.64 161.85
169.64 171.21 187.47 199.51 202.40 222.15
230.07 240.81 243.80 246.28 250.74 275.97
281.43 288.64 290.51 292.97 294.96 304.17
305.62 329.37 334.70 337.40 339.92 362.73
375.27 383.75 399.14 408.75 422.58 424.46
435.16 439.42 444.94 465.37 467.24 480.36
487.81 495.67 498.71 538.08 545.34 547.22
560.03 562.27 607.96 621.15 625.20 633.90
645.50 650.70 657.12 664.28 700.61 708.46
722.84 725.65 756.85 768.89 785.10 790.11
798.10 814.80 816.67 824.09 838.07 854.58
857.78 866.57 872.10 881.51 889.44 901.92
914.82 922.08 923.96 951.35 959.80 960.27
971.87 980.70 985.22 987.86 989.25 990.52
999.24 1000.80 1014.20 1017.51 1020.97 1022.94
1030.49 1031.80 1039.50 1047.36 1056.07 1061.52
1063.36 1064.37 1068.09 1085.28 1087.70 1106.50
1122.37 1125.67 1140.08 1142.52 1156.83 1162.57
1175.59 1182.01 1192.16 1200.36 1204.51 1221.73
1228.18 1230.35 1235.75 1238.07 1243.56 1255.40
1256.74 1258.96 1276.55 1301.67 1324.84 1327.30
1331.06 1332.26 1344.76 1349.49 1354.00 1360.25
1372.65 1387.82 1396.03 1406.28 1409.00 1415.79
1424.11 1426.08 1428.63 1430.97 1436.22 1446.86
1447.90 1457.28 1464.11 1488.51 1492.07 1493.79
1494.56 1499.07 1500.15 1501.19 1503.43 1503.98
1505.12 1508.90 1510.79 1515.21 1516.61 1517.41
1518.54 1519.14 1523.44 1536.45 1538.61 1547.47
1571.46 1589.57 1624.12 1629.94 1648.31 1649.86
1657.88 3019.14 3023.71 3041.51 3045.18 3046.64
3050.99 3054.57 3057.07 3063.03 3069.26 3081.24
3110.04 3111.46 3120.02 3122.28 3125.24 3125.55
3126.16 3131.87 3136.68 3140.51 3142.43 3146.49
3151.74 3163.20 3163.73 3193.08 3193.59 3193.94
3200.31 3204.29 3209.32 3213.59 3216.14 3219.30
3221.12 3224.70 3232.45 3239.61 3251.39 3263.55

==============================

A9

==============================

13.91 19.12 27.78 29.31 33.98 41.14
46.88 47.71 52.82 62.13 72.31 75.56
78.05 80.75 87.41 92.81 101.07 109.25
113.60 123.16 126.98 128.78 142.16 165.76
168.87 181.31 192.03 207.94 215.63 216.96
219.56 233.00 241.84 252.25 254.74 265.55
270.83 278.67 289.71 294.70 294.97 303.76
310.47 324.95 339.19 346.39 368.19 378.90
390.35 397.44 416.60 417.66 421.03 432.35
437.21 449.66 453.32 466.13 489.59 505.86
523.63 537.33 547.60 553.79 556.59 586.15
604.08 612.88 614.07 622.45 641.96 649.65
655.12 656.26 665.12 696.30 712.08 720.58
730.08 739.89 771.20 779.41 787.71 819.65
826.11 833.82 840.81 855.99 863.57 870.20
877.22 891.71 914.70 932.35 951.65 956.59
962.18 967.50 975.18 978.61 984.85 986.78
992.73 995.40 1012.72 1013.95 1017.15 1023.36
1023.66 1032.42 1033.67 1034.59 1054.33 1057.07
1062.28 1065.65 1066.24 1066.87 1094.51 1120.75
1137.24 1140.74 1167.22 1171.99 1189.29 1201.90
1220.66 1227.41 1231.74 1236.47 1239.56 1241.49
1244.07 1263.07 1291.08 1311.41 1327.69 1343.05
1345.41 1349.02 1352.36 1367.33 1372.92 1394.07
1405.10 1412.67 1417.74 1421.81 1428.39 1437.32
1444.34 1448.34 1460.41 1477.27 1487.01 1489.30
1491.89 1493.51 1499.97 1502.92 1505.11 1505.28
1508.77 1511.91 1517.31 1518.79 1519.32 1541.97
1545.52 1550.38 1584.76 1624.24 1630.07 1654.24
1654.72 1659.55 1660.23 1674.81 3042.36 3043.43
3045.11 3053.18 3058.54 3059.76 3105.05 3123.19
3128.15 3129.75 3131.09 3132.74 3137.06 3138.61
3139.42 3146.96 3164.02 3186.20 3187.13 3188.61
3194.95 3195.41 3206.16 3206.91 3207.71 3211.46
3216.66 3222.50 3224.11 3227.90 3261.52 3278.79

==============================

C1

==============================

18.19 30.60 40.87 42.98 51.65 56.59
63.41 79.79 83.21 97.20 98.53 113.23
127.95 134.88 142.04 154.51 173.48 180.37
194.85 206.30 226.99 231.81 258.75 265.10
270.76 283.73 290.48 320.38 347.61 350.87
393.19 400.31 415.53 438.30 455.13 465.88
471.21 478.29 491.15 510.20 544.91 553.82
607.49 617.72 622.89 647.08 657.34 682.14
702.48 714.68 730.84 755.45 775.45 779.72
782.83 815.70 856.16 862.43 881.99 902.69
906.37 919.10 936.73 945.24 949.87 959.48
979.31 983.98 989.59 990.67 1006.36 1013.09
1015.41 1017.34 1020.69 1022.87 1025.75 1034.11
1048.70 1058.99 1061.96 1069.64 1079.20 1120.79
1121.75 1142.50 1178.39 1191.38 1204.26 1207.40
1220.15 1225.28 1243.20 1258.78 1275.92 1296.80
1318.30 1327.71 1328.53 1343.25 1353.54 1360.95
1367.54 1395.34 1399.48 1404.68 1424.35 1434.34
1436.72 1451.18 1457.22 1461.54 1479.11 1483.87
1491.60 1496.21 1498.98 1502.27 1503.20 1507.69
1512.13 1514.09 1514.12 1522.82 1545.60 1558.89
1576.82 1584.75 1629.68 1646.78 1656.34 3022.39
3030.34 3042.11 3051.57 3055.64 3058.55 3064.83
3076.31 3111.94 3119.82 3123.09 3129.02 3134.42
3157.41 3163.83 3165.65 3175.96 3189.19 3197.40
3201.94 3206.42 3212.60 3215.78 3222.79 3223.41
3231.79 3252.17 3253.27

==============================

C1-TS

==============================

-804.36 20.27 28.47 32.74 47.15 49.19
63.01 76.70 82.63 85.35 90.37 107.25
128.84 135.84 149.14 157.41 170.50 190.26
196.14 218.08 229.39 234.43 257.88 268.18
278.54 291.70 296.32 316.81 335.43 347.15
379.26 387.15 415.75 443.11 462.20 478.55
484.77 495.59 497.19 548.81 558.69 561.49
592.32 612.38 626.52 647.18 656.46 661.13
696.74 710.20 715.84 735.36 759.41 770.52
774.38 779.45 859.15 860.51 864.39 878.16
880.86 905.48 910.13 924.63 938.88 950.89
955.29 977.87 981.70 986.21 988.51 1003.25
1015.72 1016.80 1020.89 1024.58 1038.21 1039.10
1043.54 1050.21 1054.49 1063.64 1067.35 1109.08
1120.12 1129.66 1142.32 1177.79 1190.24 1197.44
1203.72 1220.31 1225.57 1239.56 1245.90 1259.12
1303.91 1323.16 1328.49 1332.36 1346.20 1352.08
1355.62 1367.78 1390.19 1396.10 1396.99 1419.60
1424.47 1428.93 1434.69 1437.56 1460.58 1478.79
1484.15 1487.46 1492.98 1494.91 1497.72 1501.76
1504.80 1508.50 1514.03 1515.79 1520.73 1545.13
1555.55 1590.16 1631.51 1644.11 1648.24 1657.46
3025.42 3035.13 3043.17 3052.44 3061.55 3063.86
3064.83 3091.95 3113.47 3118.47 3127.11 3138.75
3158.35 3167.83 3179.38 3180.57 3188.06 3193.68
3201.24 3202.43 3209.95 3210.63 3218.36 3220.15
3227.98 3244.16 3258.09

==============================
C2
==============================
21.39 31.49 51.16 68.64 71.62 82.82
88.89 101.81 109.90 125.44 130.97 159.26
170.45 200.46 209.59 235.49 257.23 275.06
284.80 303.84 324.66 340.99 379.93 406.43
420.06 428.35 444.93 461.02 468.62 481.52
495.23 549.99 558.36 582.47 609.17 624.44
655.86 661.34 707.38 718.05 735.22 748.43
762.55 777.43 782.12 849.46 861.35 869.90
888.81 890.15 911.76 921.33 943.02 952.72
957.17 975.90 984.89 986.08 994.69 1000.61
1008.13 1016.11 1020.52 1025.06 1042.78 1045.87
1055.64 1061.82 1065.13 1108.60 1119.73 1129.81
1165.47 1175.70 1192.81 1204.34 1214.72 1216.02
1222.84 1240.43 1247.89 1275.44 1299.76 1304.09
1322.35 1334.39 1346.36 1356.45 1368.97 1388.64
1390.42 1424.33 1433.67 1436.61 1468.83 1483.98
1492.91 1494.81 1499.04 1503.79 1506.50 1509.95
1514.05 1514.25 1519.75 1546.22 1557.11 1607.04
1627.13 1638.26 1653.81 3022.94 3036.67 3042.75
3052.14 3055.74 3065.76 3083.45 3107.82 3112.77
3119.11 3128.89 3170.31 3179.66 3186.11 3193.44
3199.68 3201.69 3208.74 3209.85 3218.62 3219.03
3224.99 3226.50 3230.47
==============================
N1
==============================
14.12 37.28 42.61 49.76 54.87 61.67
68.92 78.42 89.44 93.99 101.37 111.65
112.57 125.21 141.49 154.69 161.06 169.38
191.02 193.63 201.40 213.28 221.62 249.94
258.66 267.93 280.24 285.22 293.49 326.85
338.34 342.82 369.95 381.78 407.17 420.16
427.69 428.94 462.52 474.53 485.72 487.79
494.25 510.27 539.14 551.46 559.93 600.64
611.49 624.75 635.93 641.35 643.71 652.17
662.60 687.39 704.08 709.05 717.49 736.54
766.95 780.27 785.48 812.75 829.32 849.74
861.71 873.38 890.48 910.98 922.80 958.02
959.87 984.48 985.72 988.72 993.39 998.26
1012.92 1019.46 1019.70 1020.12 1025.40 1028.92
1030.56 1045.13 1052.36 1058.36 1063.47 1064.62
1079.41 1123.86 1135.53 1145.15 1161.38 1177.63
1194.29 1203.54 1220.56 1225.53 1229.81 1241.09
1242.36 1310.66 1320.96 1324.51 1340.10 1350.70
1366.19 1374.89 1388.96 1393.08 1422.19 1425.37
1426.92 1442.87 1453.96 1459.11 1466.49 1484.82
1490.60 1491.30 1495.63 1500.67 1501.59 1507.14
1516.16 1522.24 1527.15 1545.43 1586.67 1604.50
1627.71 1640.33 1641.07 1655.49 3040.73 3056.41
3062.94 3105.30 3132.27 3141.96 3144.80 3162.10
3175.89 3191.60 3199.32 3200.24 3202.96 3203.60
3209.47 3213.14 3215.38 3220.58 3222.37 3222.91
3231.29 3232.40 3251.79
==============================
N2
==============================
13.12 31.49 38.06 38.83 43.44 49.13
64.17 68.94 71.78 76.49 92.47 99.47
109.99 116.90 129.13 144.77 151.45 165.13
173.41 179.66 195.21 199.55 213.74 218.29
232.67 243.98 253.84 260.54 279.14 281.44
283.78 285.75 295.03 302.62 325.63 336.19
342.32 362.88 373.09 393.86 408.25 419.35
422.83 426.03 434.73 439.14 464.14 477.66
486.64 495.03 505.35 538.68 545.83 553.07
560.90 611.21 621.38 624.80 632.96 639.92
643.05 651.88 662.45 703.50 708.93 718.33
736.96 770.67 780.45 785.38 785.74 809.67
813.57 829.21 849.33 860.95 874.51 889.38
907.70 914.99 923.52 952.68 958.30 961.21
984.31 985.59 989.11 989.74 992.88 999.37
1012.57 1019.92 1020.04 1024.70 1029.73 1030.39
1045.05 1053.14 1053.76 1058.79 1061.68 1065.19
1076.96 1125.54 1136.20 1143.45 1161.94 1177.63
1194.02 1204.66 1221.47 1225.46 1230.55 1233.94
1240.66 1241.81 1249.72 1255.86 1311.08 1317.47
1325.59 1340.66 1351.82 1366.32 1376.84 1392.21
1409.77 1416.15 1422.29 1427.07 1427.32 1433.78
1453.01 1454.27 1459.81 1485.27 1490.30 1491.65
1492.03 1492.03 1496.57 1501.73 1501.98 1503.30
1506.19 1515.02 1515.70 1516.09 1522.71 1546.35
1548.72 1587.30 1603.33 1629.32 1641.10 1641.83
1656.94 3041.37 3045.11 3055.27 3055.30 3062.52
3106.20 3121.73 3130.07 3130.37 3135.12 3140.03
3142.19 3145.14 3154.32 3158.23 3190.46 3199.02
3199.50 3200.18 3201.82 3208.14 3211.97 3214.82
3220.18 3222.15 3222.76 3230.66 3234.50 3252.11
==============================
N3
==============================
14.28 19.18 34.86 36.95 39.20 45.01
48.10 56.27 63.61 64.85 71.39 73.23
78.34 83.17 89.86 91.11 97.31 101.93
105.59 113.62 117.80 118.93 124.56 139.65
148.86 151.60 166.64 179.58 182.68 192.13
205.25 212.03 232.27 238.80 249.66 259.11
262.12 275.68 279.69 287.14 294.80 300.12
304.55 317.95 322.58 339.80 347.53 366.83
373.85 377.34 392.29 416.55 421.54 422.57
429.52 442.64 445.49 454.51 471.49 484.40
504.11 518.46 541.79 556.91 562.36 567.10
586.71 605.07 607.43 623.70 625.75 636.07
647.47 654.82 668.27 675.71 700.27 714.49
718.59 749.02 774.87 776.20 782.85 798.66
803.97 833.34 844.15 850.52 872.12 876.69
889.23 894.80 914.04 923.90 934.49 950.02
955.22 958.81 975.73 982.03 985.85 995.29
996.87 1012.18 1014.56 1016.87 1018.52 1018.91
1024.97 1026.58 1028.82 1044.01 1056.22 1057.70
1059.43 1061.16 1064.70 1068.09 1075.29 1121.50
1121.94 1131.47 1162.10 1171.43 1193.73 1202.39
1216.66 1224.08 1228.95 1233.90 1236.41 1242.30
1245.59 1253.40 1275.05 1286.20 1295.80 1321.71
1323.03 1349.84 1350.56 1357.71 1372.23 1390.28
1391.48 1410.51 1416.27 1422.42 1425.54 1437.23
1445.19 1445.71 1466.27 1477.73 1486.13 1488.77
1490.24 1494.03 1496.44 1499.84 1503.54 1506.27
1508.44 1512.21 1514.12 1517.52 1523.03 1537.70
1539.56 1550.87 1611.57 1621.28 1628.01 1639.23
1651.67 1656.79 1690.25 1726.46 3036.24 3042.47
3045.44 3045.50 3050.50 3057.78 3106.44 3106.88
3115.36 3124.34 3127.66 3130.79 3133.77 3134.38
3136.01 3137.62 3167.94 3185.44 3186.26 3186.71
3195.17 3196.01 3197.87 3207.44 3208.82 3211.87
3220.30 3220.59 3223.54 3230.63 3232.91 3238.42
==============================
^{S}N3-TS
==============================
-211.35 11.27 15.84 23.37 33.93 37.36
44.29 45.17 49.07 53.41 56.85 64.47
71.39 76.18 80.44 83.48 88.25 97.15
97.75 100.98 106.33 107.95 111.98 117.41
120.26 126.34 130.81 136.66 142.87 146.49
154.26 159.37 165.16 171.93 180.11 190.52
191.69 195.07 206.30 216.85 228.03 236.38
236.73 247.59 255.35 259.94 268.40 278.42
279.36 282.88 287.82 292.31 298.73 305.17
306.25 326.72 335.87 345.64 352.42 354.17
369.31 382.79 384.20 397.20 412.20 414.29
418.95 419.86 431.01 450.10 475.05 478.67
487.67 497.31 513.62 524.55 540.50 556.77
557.31 562.07 598.86 605.19 609.37 613.63
628.70 644.37 647.41 656.03 662.66 665.51
675.91 707.41 713.21 717.90 725.08 754.28
760.14 770.29 783.55 786.03 803.66 815.06
826.18 827.78 862.89 863.78 867.33 879.41
891.79 899.26 913.60 925.24 925.45 934.20

942.55 951.16 952.22 954.90 967.71 971.00
983.61 985.11 988.77 997.82 1000.61 1005.93
1012.02 1019.69 1021.63 1023.01 1026.11 1032.85
1037.95 1042.98 1047.71 1051.43 1055.54 1056.61
1061.85 1063.92 1067.16 1083.83 1085.65 1100.50
1121.84 1128.22 1132.59 1135.22 1158.14 1175.48
1181.42 1191.70 1200.54 1218.65 1225.61 1226.18
1237.34 1238.72 1242.03 1245.05 1245.37 1258.03
1275.23 1287.45 1305.47 1319.25 1322.03 1325.07
1332.84 1339.28 1343.86 1345.89 1350.32 1361.04
1362.40 1373.84 1384.83 1389.31 1412.16 1412.47
1417.46 1421.52 1425.32 1430.32 1434.89 1445.32
1447.15 1448.65 1462.23 1466.93 1486.04 1489.22
1490.41 1493.37 1493.65 1497.63 1497.73 1500.31
1500.60 1504.37 1505.50 1509.58 1510.68 1513.24
1513.78 1515.65 1518.22 1519.76 1533.61 1537.32
1549.86 1597.89 1620.26 1620.54 1630.49 1647.34
1647.86 1659.20 1678.64 1708.77 2990.53 3022.92
3026.00 3035.75 3036.62 3041.93 3043.54 3049.46
3053.44 3055.95 3057.87 3065.04 3090.70 3102.38
3106.26 3106.80 3113.44 3123.80 3127.76 3131.54
3135.41 3136.44 3137.09 3144.50 3163.12 3165.34
3174.07 3182.86 3186.59 3191.49 3192.97 3193.93
3200.58 3200.87 3203.60 3205.27 3212.17 3219.77
3222.65 3222.92 3237.53 3237.95 3254.29 3294.30

==============================

^{R}N3-TS

==============================

-211.30 2.02 16.76 24.13 29.83 33.04
35.19 43.18 46.48 49.47 57.79 62.68
66.92 75.81 79.87 82.20 85.41 91.16
92.31 96.07 97.84 105.20 107.02 110.33
118.83 125.65 128.26 138.81 143.59 145.23
148.15 151.40 157.26 161.31 170.17 189.81
190.14 193.27 196.96 209.46 218.01 234.60
238.39 242.57 254.23 256.94 262.30 274.43
276.64 277.15 282.45 285.02 290.46 302.37
305.03 328.03 339.76 343.44 353.31 357.52
365.98 386.10 391.45 394.96 414.59 417.11
420.44 423.02 431.93 453.29 476.97 480.75
491.50 495.71 500.62 524.60 537.82 554.59
559.11 564.69 602.17 605.54 607.44 614.11
628.86 644.05 648.06 659.08 663.48 670.57
689.03 707.24 712.95 718.14 742.50 747.80
764.31 771.81 782.94 784.98 805.03 814.54
821.12 827.45 862.23 863.98 868.93 881.87
895.47 900.36 909.43 924.42 927.33 927.46
942.25 951.78 953.09 955.90 969.65 970.33
985.88 989.15 990.08 993.27 1001.64 1008.96
1012.42 1016.89 1019.67 1022.43 1027.24 1030.83
1032.59 1039.58 1046.76 1050.87 1051.24 1056.56
1061.03 1064.71 1067.42 1068.77 1081.68 1088.17
1122.56 1123.47 1132.50 1135.70 1158.43 1176.13
1191.95 1194.37 1200.71 1220.36 1225.33 1227.25
1237.50 1239.24 1243.39 1246.51 1247.17 1260.32
1276.44 1282.07 1311.26 1315.54 1320.97 1327.61
1333.15 1338.28 1343.03 1344.93 1350.48 1362.15
1364.85 1374.86 1383.41 1392.02 1410.82 1411.64
1415.49 1421.68 1425.18 1434.18 1435.94 1446.80
1447.82 1458.11 1463.75 1468.97 1486.59 1489.02
1490.33 1490.87 1495.65 1498.07 1499.70 1501.38
1502.44 1504.28 1505.31 1506.87 1509.72 1513.42
1514.46 1515.95 1520.42 1523.65 1533.39 1537.38
1549.23 1593.16 1616.26 1619.91 1630.61 1647.54
1649.49 1659.63 1696.02 1726.20 2988.19 3002.45
3030.53 3033.57 3039.27 3041.93 3043.42 3046.99
3051.49 3056.98 3061.08 3071.51 3090.15 3101.35
3102.17 3105.98 3115.41 3123.46 3125.61 3125.72
3127.97 3129.84 3134.14 3138.47 3145.03 3157.13
3177.92 3180.47 3182.55 3193.91 3193.99 3194.17
3195.29 3196.02 3203.15 3204.36 3215.23 3219.25
3219.71 3226.18 3236.81 3239.35 3240.70 3303.13

7. References

1. Semakul, N.; Jackson, K. E.; Paton, R. S.; Rovis, T., Heptamethylindenyl (Ind*) enables diastereoselective benzamidation of cyclopropenes via Rh(iii)-catalyzed C-H activation. *Chem. Sci.* **2017,** *8*, 1015-1020.
2. Arnold, T. A. Q.; Buffet, J. C.; Turner, Z. R.; O'Hare, D., Synthesis, characterisation, and polymerisation studies of hexamethylindenyl zirconocenes and hafnocenes. *J. Organomet. Chem.* **2015,** *792*, 55-65.
3. Yan, Z.; Chong, S.; Lin, H.; Yang, Q.; Wang, X.; Zhang, W.; Zhang, X.; Zeng, Z.; Su, Y., Design, synthesis and biological evaluation of tetrazole-containing RXRalpha ligands as anticancer agents. *Eur. J. Med. Chem.* **2019,** *164*, 562-575.
4. Farr, C. M. B.; Kazerouni, A. M.; Park, B.; Poff, C. D.; Won, J.; Sharp, K. R.; Baik, M. H.; Blakey, S. B., Designing a Planar Chiral Rhodium Indenyl Catalyst for Regio- and Enantioselective Allylic C-H Amidation. *J. Am. Chem. Soc.* **2020,** *142*, 13996-14004.
5. Wang, A.; Venditto, N. J.; Darcy, J. W.; Emmert, M. H., Nondirected, Cu-Catalyzed sp3 C–H Aminations with Hydroxylamine-Based Amination Reagents: Catalytic and Mechanistic Studies. *Organometallics* **2017,** *36*, 1259-1268.
6. Matsumoto, K.; Usuda, K.; Okabe, H.; Hashimoto, M.; Shimada, Y., Synthesis of optically active heterocyclic compounds via deracemization of 1,2-diol monotosylate derivatives bearing a long aliphatic chain by a combination of enzymatic hydrolysis with Mitsunobu inversion. *Tetrahedron: Asymmetry* **2013,** *24*, 108-115.
7. Brown, H. C.; Lynch, G. J., Solvomercuration-demercuration. 8. Oxymercuration-demercuration of methoxy-, hydroxy-. and acetoxy-substituted alkenes. *The Journal of Organic Chemistry* **1981,** *46*, 531-538.
8. Li, X.; He, S.; Song, Q., Diethylzinc-Mediated Radical 1,2-Addition of Alkenes and Alkynes. *Org. Lett.* **2021,** *23*, 2994-2999.
9. Kalyankar, K. B.; Das, S., First total synthesis of 5(S)-hydroxyrecifeiolide. *Synth. Commun.* **2020,** *50*, 322-328.
10. Karabiyikoglu, S.; Boon, B. A.; Merlic, C. A., Cycloaddition Reactions of Cobalt-Complexed Macrocyclic Alkynes: The Transannular Pauson–Khand Reaction. *The Journal of Organic Chemistry* **2017,** *82*, 7732-7744.
11. Smith, B. J.; Sulikowski, G. A., Total Synthesis of (±)-Haliclonacyclamine C. *Angew. Chem. Int. Ed.* **2010,** *49* , 1599-1602.
12. Bender, T. A.; Bergman, R. G.; Raymond, K. N.; Toste, F. D., A Supramolecular Strategy for Selective Catalytic Hydrogenation Independent of Remote Chain Length. *J. Am. Chem. Soc.* **2019,** *141*, 11806-11810.
13. Marsh, G. P.; Parsons, P. J.; McCarthy, C.; Corniquet, X. G., An Efficient Synthesis of Nitroalkenes by Alkene Cross Metathesis: Facile Access to Small Ring Systems. *Org. Lett.* **2007,** *9*, 2613-2616.
14. Yang, C.; Gao, Y.; Bai, S.; Jiang, C.; Qi, X., Chemoselective Cross-Coupling of gem-Borazirconocene Alkanes with Aryl Halides. *J. Am. Chem. Soc.* **2020,** *142*, 11506-11513.
15. Basauri-Molina, M.; Verhoeven, D. G. A.; van Schaik, A. J.; Kleijn, H.; Klein Gebbink, R. J. M., Ring-Closing and Cross-Metathesis with Artificial Metalloenzymes Created by Covalent Active Site-Directed Hybridization of a Lipase. *Chem. Eur. J.* **2015,** *21*, 15676-15685.
16. Li, Y.-G.; Li, L.; Yang, M.-Y.; He, G.; Kantchev, E. A. B., A Bulky Disulfoxide Ligand for Pd-Catalyzed Oxidative Allylic C–H Amination with 2,2,2-Trichloroethyl Tosyl Carbamate. *The Journal of Organic Chemistry* **2017,** *82*, 4907-4917.
17. Buslov, I.; Becouse, J.; Mazza, S.; Montandon-Clerc, M.; Hu, X., Chemoselective Alkene Hydrosilylation Catalyzed by Nickel Pincer Complexes. *Angew. Chem. Int. Ed.* **2015,** *54*, 14523-14526.
18. Meng, Q.-Y.; Schirmer, T. E.; Katou, K.; König, B., Controllable Isomerization of Alkenes by Dual Visible-Light-Cobalt Catalysis. *Angew. Chem. Int. Ed.* **2019,** *58* , 5723-5728.
19. Youn, S. W.; Pastine, S. J.; Sames, D., Ru(III)-catalyzed cyclization of arene-alkene substrates via intramolecular electrophilic hydroarylation. *Org. Lett.* **2004,** *6*, 581-4.
20. Cleveland, A. H.; Fronczek, F. R.; Kartika, R., Synthesis of Vicinal Dichlorides via Activation of Aliphatic Terminal Epoxides with Triphosgene and Pyridine. *The Journal of Organic Chemistry* **2018,** *83*, 3367-3377.
21. Maust, M. C.; Hendy, C. M.; Jui, N. T.; Blakey, S. B., Switchable Regioselective 6-endo or 5-exo Radical Cyclization via Photoredox Catalysis. *J. Am. Chem. Soc.* **2022,** *144*, 3776-3781.
22. Yadav, J. S.; Kumar, G. G. K. S. N., A concise stereoselective formal total synthesis of the cytotoxic macrolide (+)-Neopeltolide via Prins cyclization. *Tetrahedron* **2010,** *66*, 480-487.
23. Breitenbach, R.; Chiu, C. K. F.; Massett, S. S.; Meltz, M.; Murtiashaw, C. W.; Pezzullo, S. L.; Staigers, T., A practical synthesis of 3(S)-methyl-heptanoic acid from (S)-citronellol. *Tetrahedron-Asymmetry* **1996,** *7*, 435-442.
24. Wu, B.; Woodward, R.; Wen, L.; Wang, X.; Zhao, G.; Wang, P. G., Synthesis of a Comprehensive Polyprenol Library for Evaluation of Bacterial Enzyme Lipid Substrate Specificity. *Eur. J. Org. Chem.,* **2013,** *2013*, 8162-8173.
25. Kumar, S. M.; Prasad, K. R., Enantiospecific formal total synthesis of iriomoteolide 3a. *Chem. Asian J.,* **2014,** *9*, 3431-9.

26. Perez, S. J.; Purino, M. A.; Cruz, D. A.; Lopez-Soria, J. M.; Carballo, R. M.; Ramirez, M. A.; Fernandez, I.; Martin, V. S.; Padron, J. I., Enantiodivergent Synthesis of (+)- and (-)-Pyrrolidine 197B: Synthesis of trans-2,5-Disubstituted Pyrrolidines by Intramolecular Hydroamination. *Chem. Eur. J.* **2016,** *22*, 15529-15535.
27. Parr, R. G.; Weitao, Y., *Density-Functional Theory of Atoms and Molecules*. Oxford University Press: 1994.
28. Bochevarov, A. D.; Harder, E.; Hughes, T. F.; Greenwood, J. R.; Braden, D. A.; Philipp, D. M.; Rinaldo, D.; Halls, M. D.; Zhang, J.; Friesner, R. A., Jaguar: A high-performance quantum chemistry software program with strengths in life and materials sciences. *Int. J. Quantum Chem* **2013,** *113*, 2110-2142.
29. Becke, A. D., A new mixing of Hartree–Fock and local density-functional theories. *J. Chem. Phys.* **1993,** *98*, 1372-1377.
30. Lee, C.; Yang, W.; Parr, R. G., Development of the Colle-Salvetti correlation-energy formula into a functional of the electron density. *Phys. Rev. B* **1988,** *37*, 785-789.
31. Grimme, S.; Antony, J.; Ehrlich, S.; Krieg, H., A consistent and accurate ab initio parametrization of density functional dispersion correction (DFT-D) for the 94 elements H-Pu. *J. Chem. Phys.* **2010,** *132*, 154104.
32. Goerigk, L.; Grimme, S., A thorough benchmark of density functional methods for general main group thermochemistry, kinetics, and noncovalent interactions. *PCCP* **2011,** *13*, 6670-6688.
33. Hay, P. J.; Wadt, W. R., Ab initio effective core potentials for molecular calculations. Potentials for the transition metal atoms Sc to Hg. *J. Chem. Phys.* **1985,** *82*, 270-283.
34. Wadt, W. R.; Hay, P. J., Ab initio effective core potentials for molecular calculations. Potentials for main group elements Na to Bi. *J. Chem. Phys.* **1985,** *82*, 284-298.
35. Hay, P. J.; Wadt, W. R., Ab initio effective core potentials for molecular calculations. Potentials for K to Au including the outermost core orbitals. *J. Chem. Phys.* **1985,** *82*, 299-310.
36. Marten, B.; Kim, K.; Cortis, C.; Friesner, R. A.; Murphy, R. B.; Ringnalda, M. N.; Sitkoff, D.; Honig, B., New Model for Calculation of Solvation Free Energies: Correction of Self-Consistent Reaction Field Continuum Dielectric Theory for Short-Range Hydrogen-Bonding Effects. *J. Chem. Phys.* **1996,** *100*, 11775-11788.
37. Rashin, A. A.; Honig, B., Reevaluation of the Born model of ion hydration. *J. Chem. Phys.* **1985,** *89*, 5588-5593.
38. Dunning, T. H., Jr., Gaussian basis sets for use in correlated molecular calculations. I. The atoms boron through neon and hydrogen. *J. Chem. Phys.* **1989,** *90*, 1007-1023.
39. Ryu, H.; Park, J.; Kim, H. K.; Park, J. Y.; Kim, S.-T.; Baik, M.-H., Pitfalls in Computational Modeling of Chemical Reactions and How To Avoid Them. *Organometallics* **2018,** *37*, 3228-3239.
40. Azek, E.; Khalifa, M.; Bartholoméüs, J.; Ernzerhof, M.; Lebel, H., Rhodium(ii)-catalyzed C–H aminations using N-mesyloxycarbamates: reaction pathway and by-product formation. *Chem. Sci.* **2019,** *10*, 718-729.
41. Lipkowitz, K. B.; Cundari, T. R.; Boyd, D. B., *Reviews in Computational Chemistry, Volume 26*. Wiley: 2008.
42. Kitaura, K.; Morokuma, K., A new energy decomposition scheme for molecular interactions within the Hartree-Fock approximation. *Int. J. Quantum Chem* **1976,** *10*, 325-340.
43. te Velde, G.; Bickelhaupt, F. M.; Baerends, E. J.; Fonseca Guerra, C.; van Gisbergen, S. J. A.; Snijders, J. G.; Ziegler, T., Chemistry with ADF. *J. Comput. Chem.* **2001,** *22*, 931-967.
44. Van Lenthe, E.; Baerends, E. J., Optimized Slater-type basis sets for the elements 1–118. *J. Comput. Chem.* **2003,** *24*, 1142-1156.
45. Chong, D. P.; Van Lenthe, E.; Van Gisbergen, S.; Baerends, E. J., Even-tempered slater-type orbitals revisited: From hydrogen to krypton. *J. Comput. Chem.* **2004,** *25*, 1030-1036.
46. Lenthe, E. v.; Baerends, E. J.; Snijders, J. G., Relativistic regular two-component Hamiltonians. *J. Chem. Phys.* **1993,** *99*, 4597-4610.
47. Van Lenthe, E.; Baerends, E. J.; Snijders, J. G., Relativistic total energy using regular approximations. *J. Chem. Phys.* **1994,** *101*, 9783-9792.

8. NMR and HPLC Data

*2,4,5,6,7-pentamethyl-2,3-dihydro-1H-inden-1-one (**S1**)*:

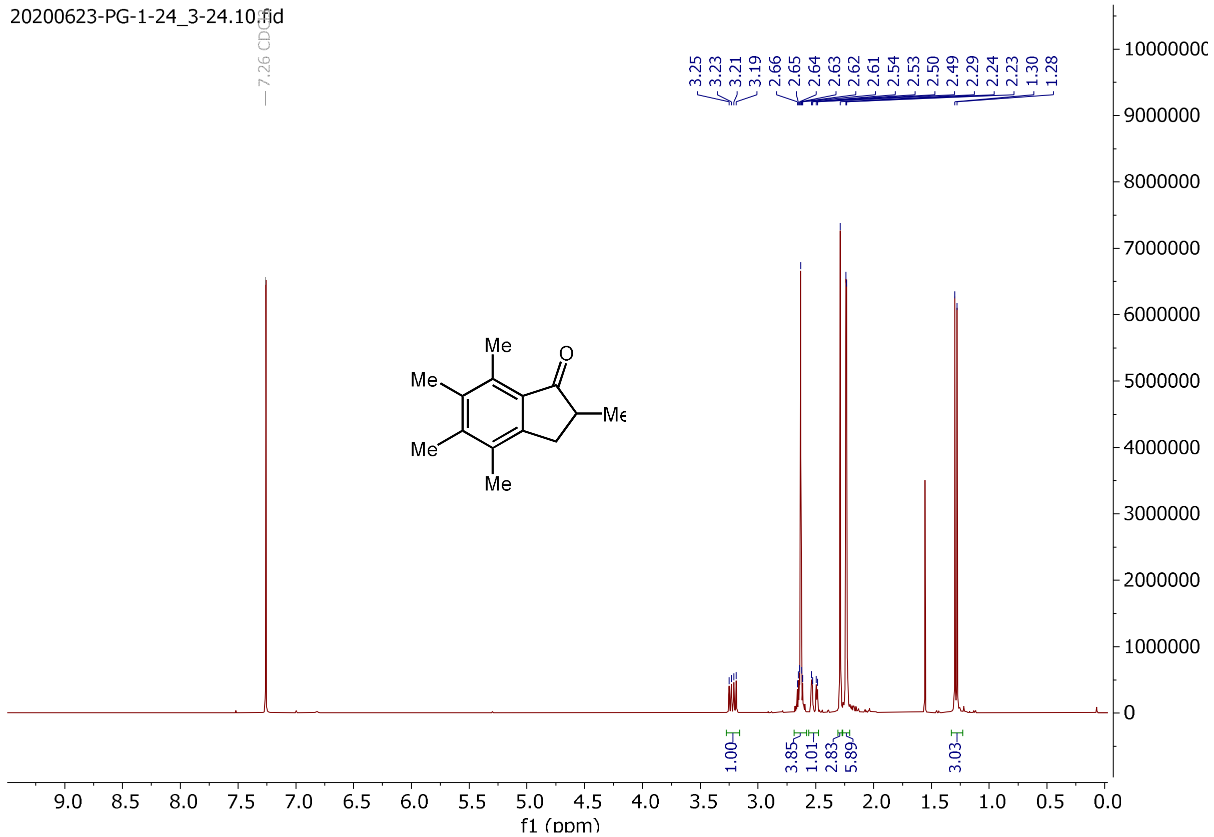

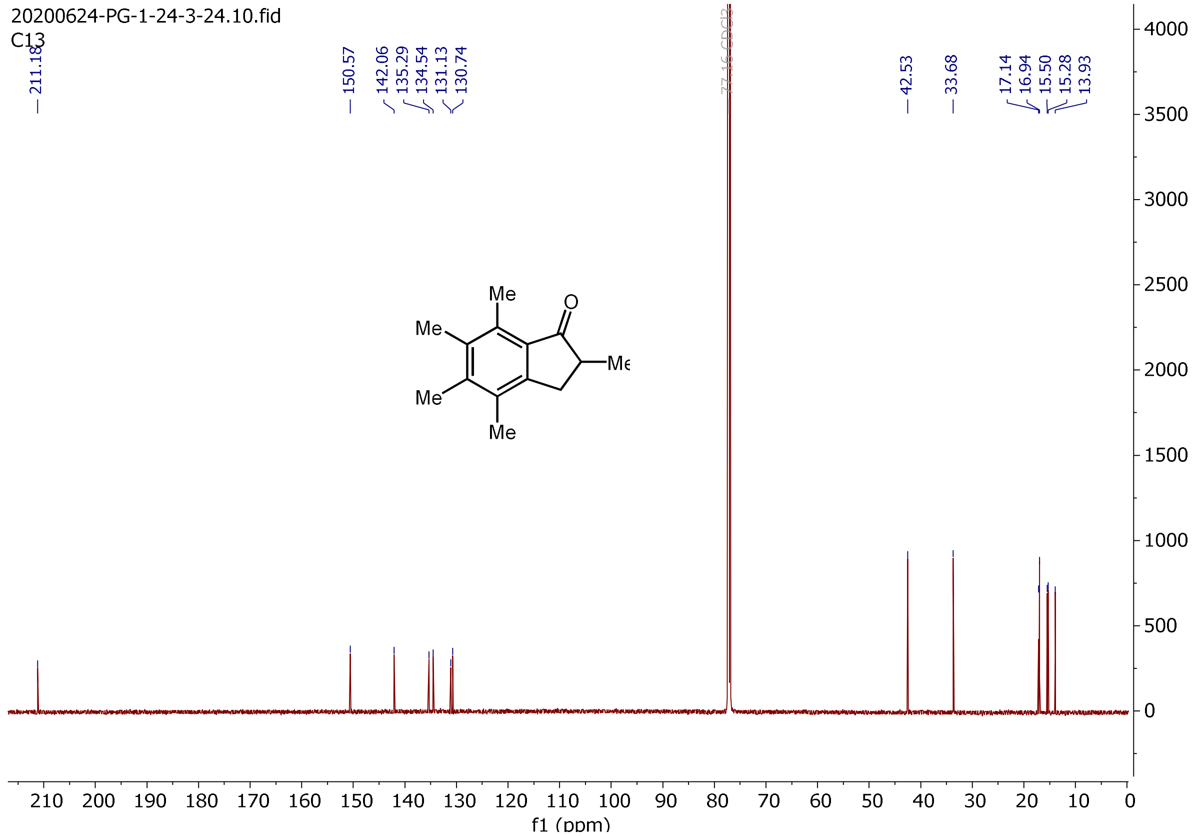

2-methyl-3-(4-(trifluoromethyl)phenyl)-1H-indene **(S3)**:

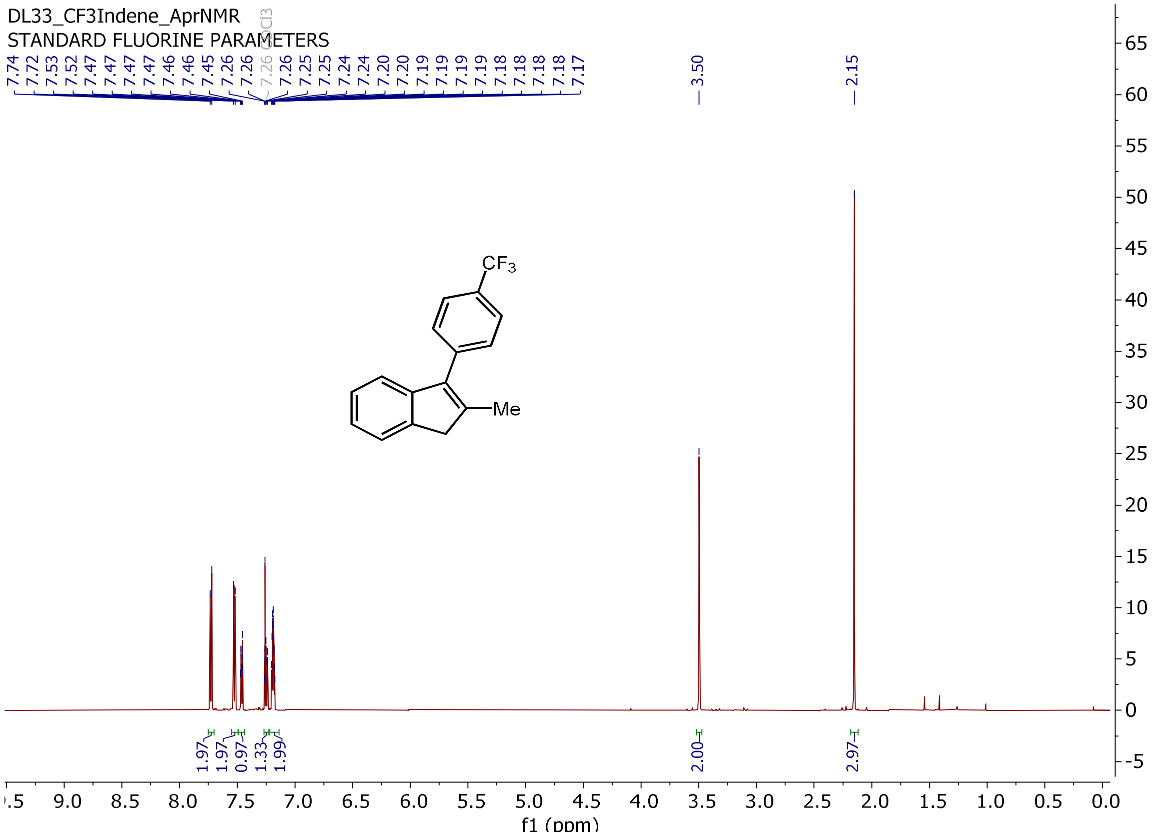

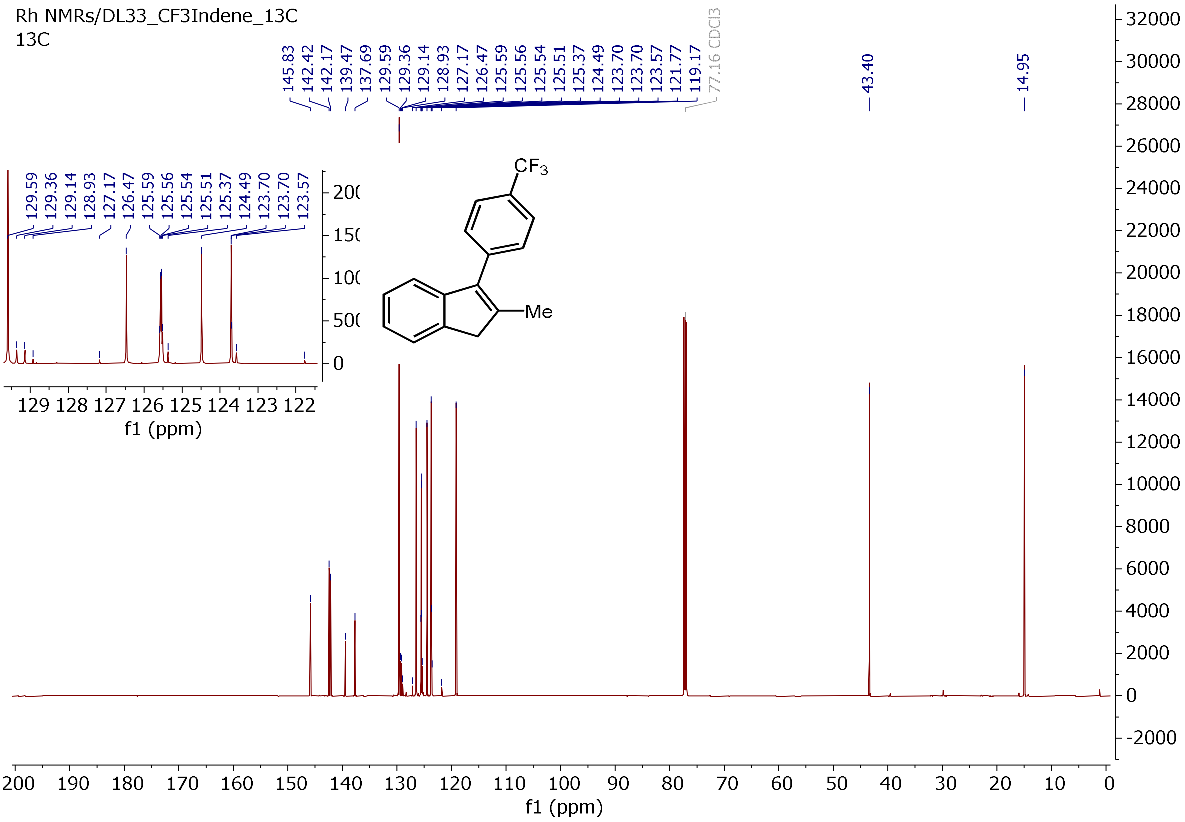

3-(4-methoxyphenyl)-2-methyl-1H-indene **(S4)**:

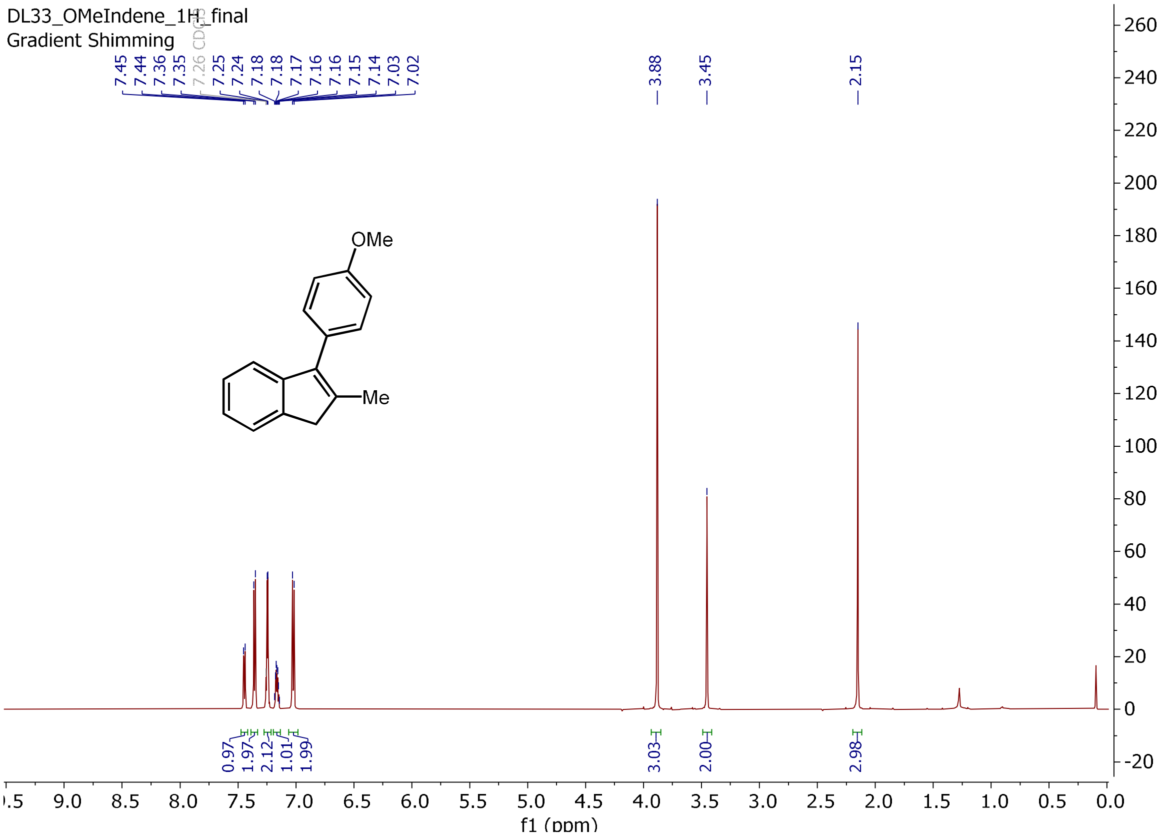

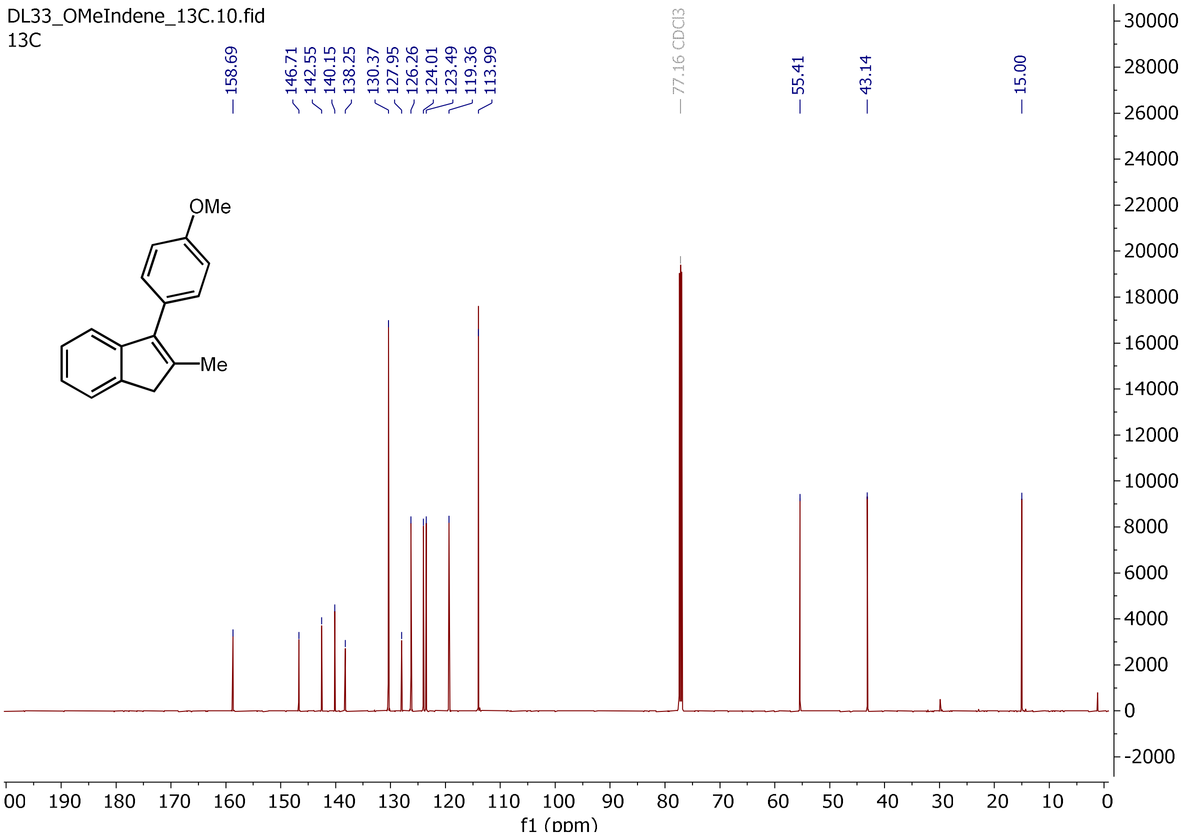

3-(4-(tert-butyl)phenyl)-2-methyl-1H-indene (**S5**):

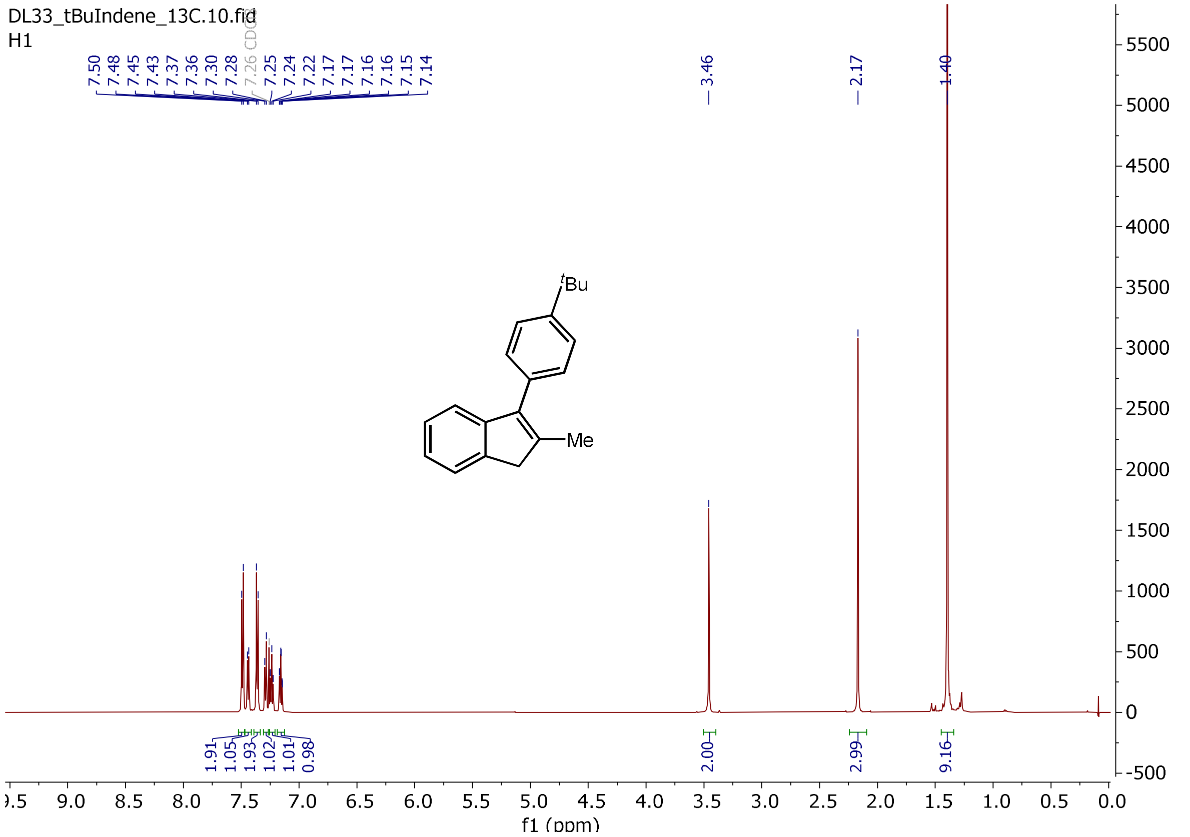

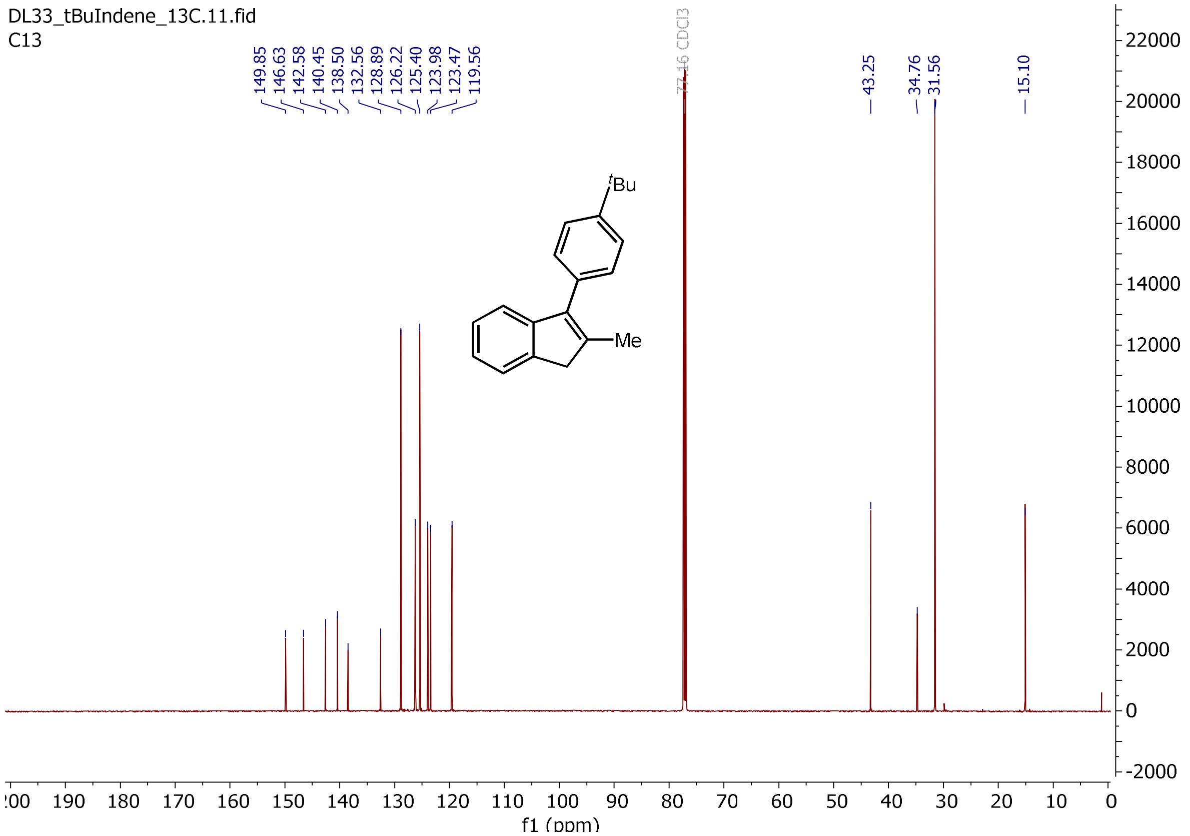

2,4,5,6,7-pentamethyl-3-phenyl-1H-indene **(S6)**:

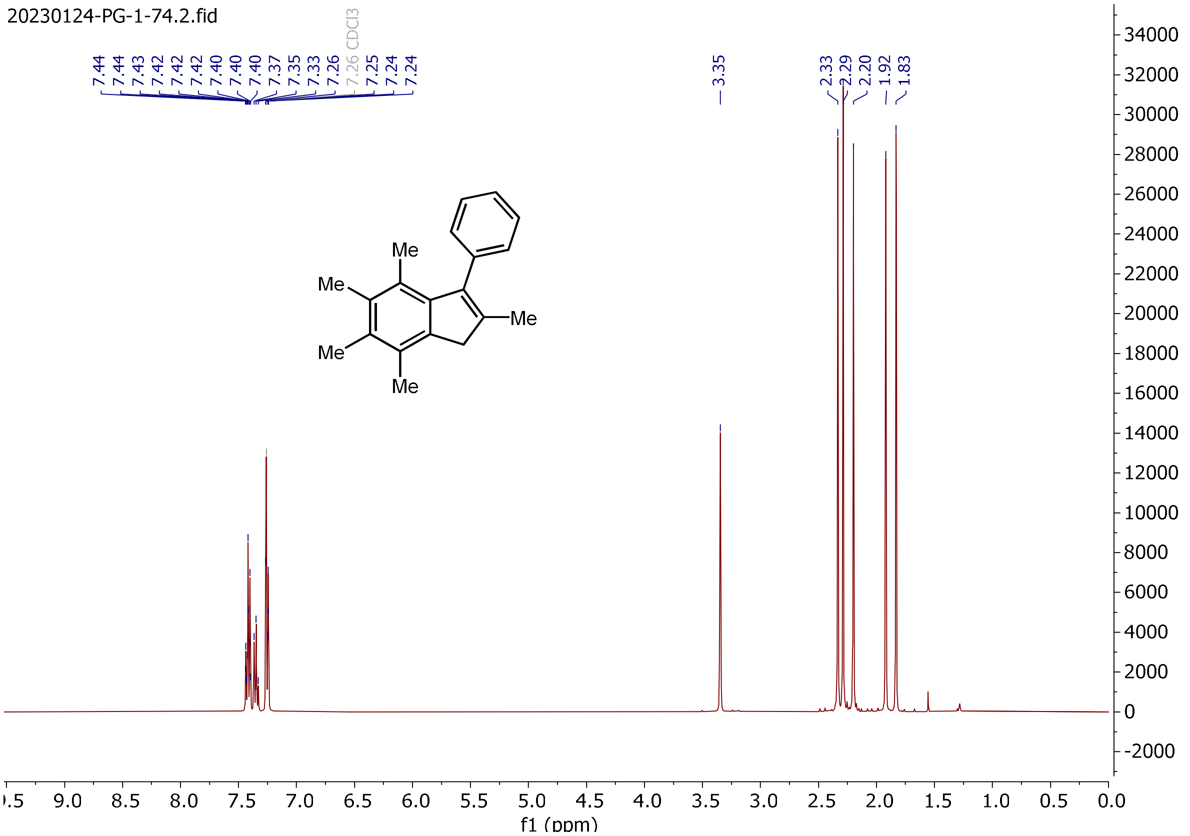

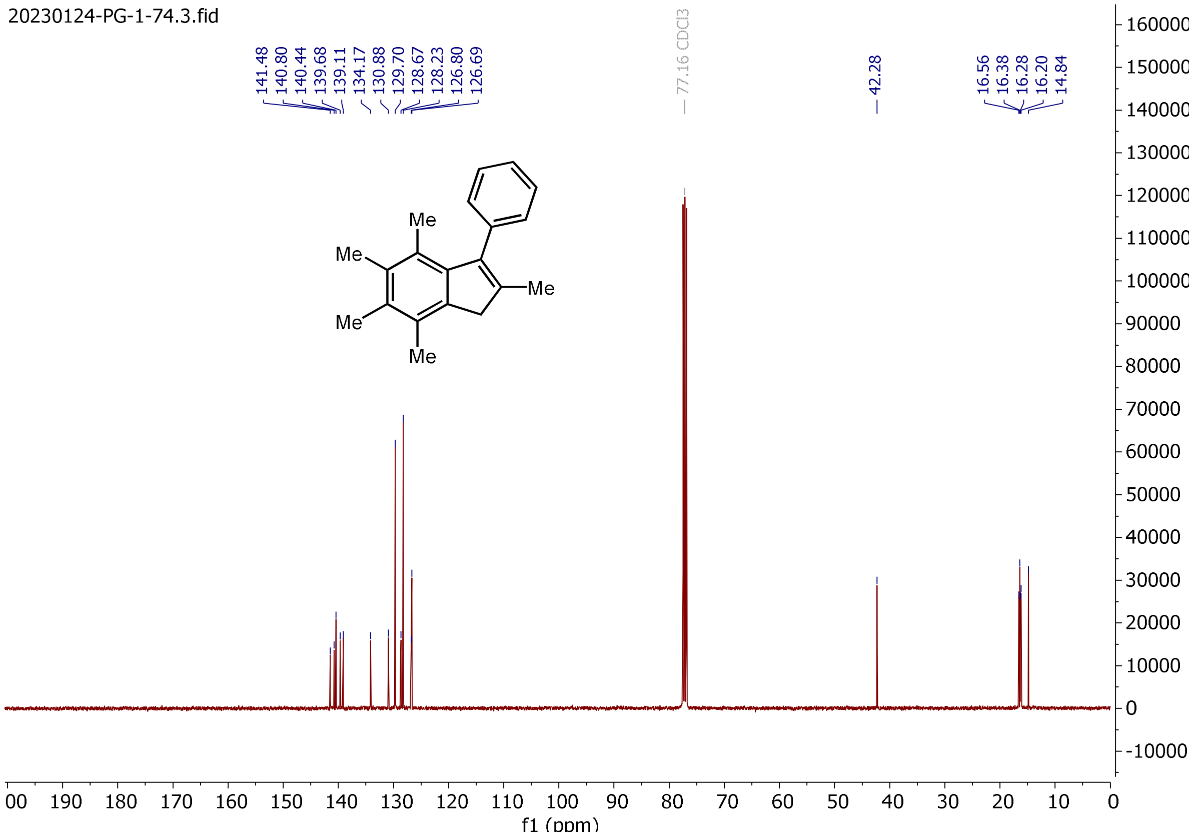

3-(4-(tert-butyl)phenyl)-2,4,5,6,7-pentamethyl-1H-indene **(S7)**:

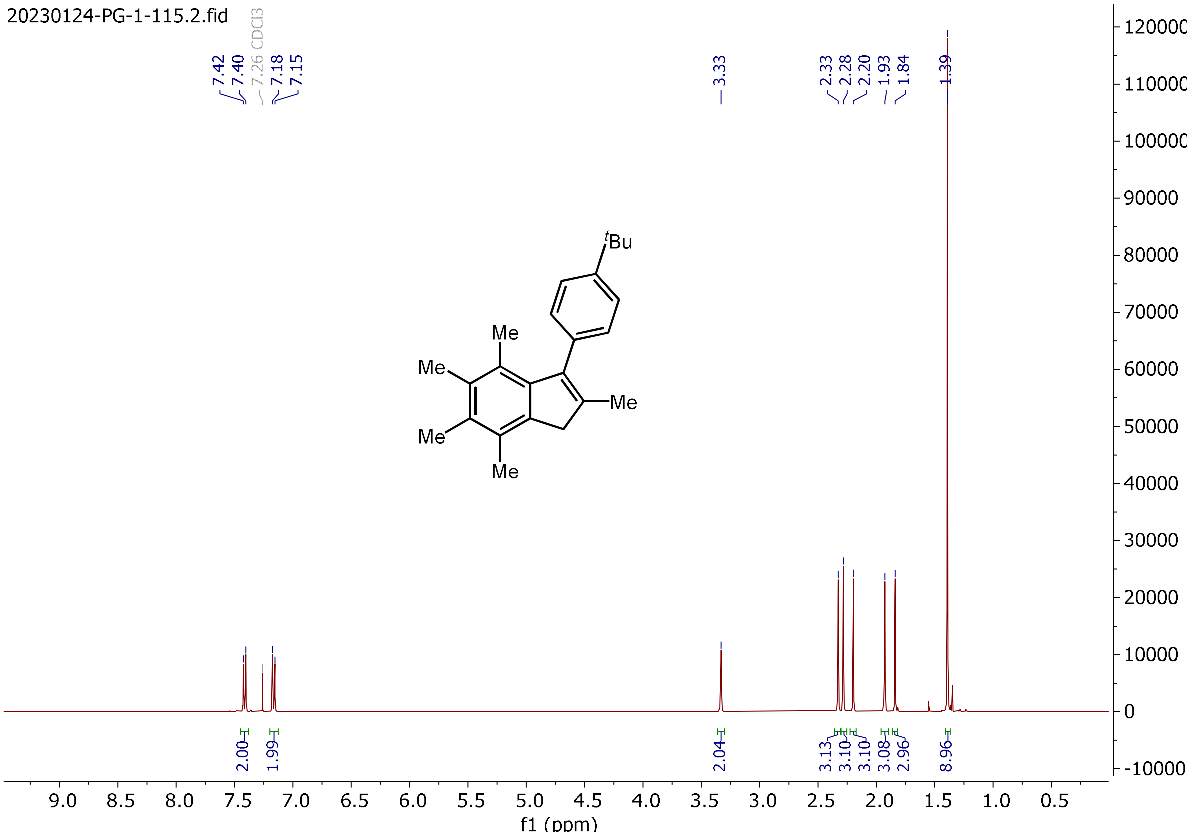

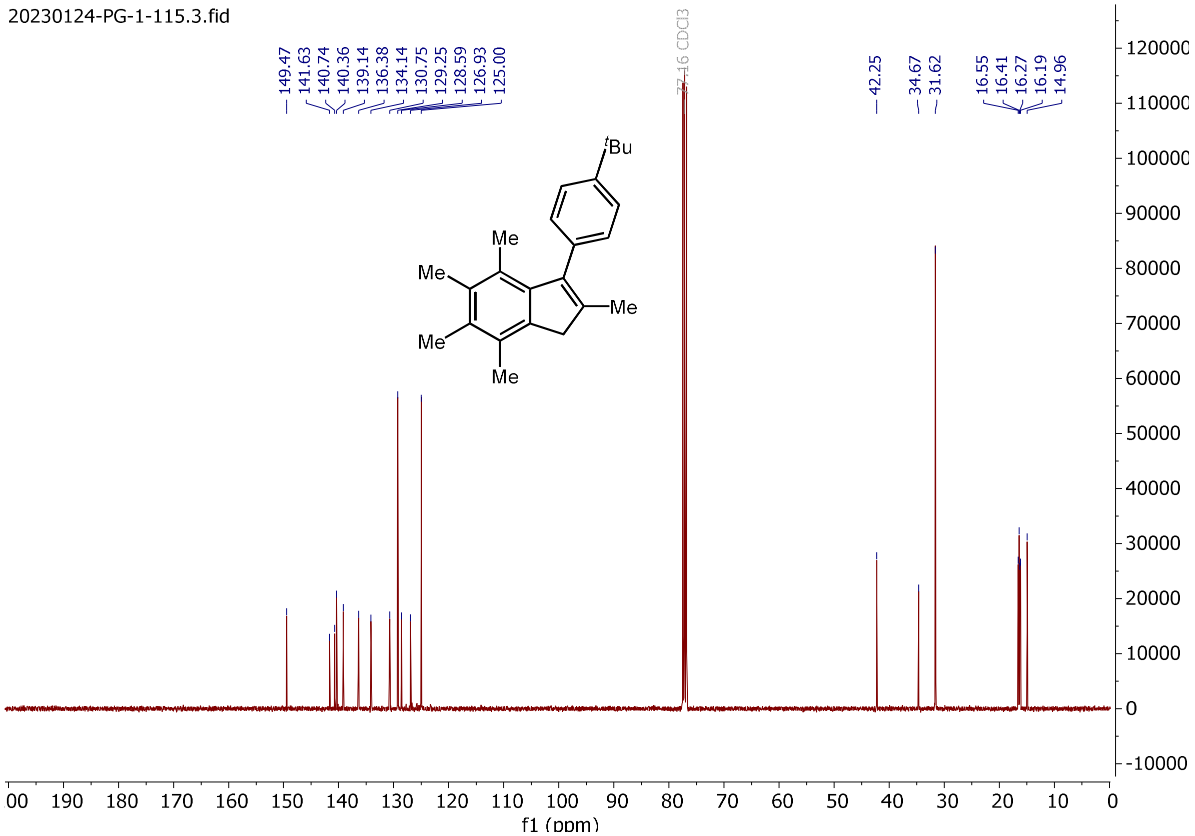

(±)-1,5-cyclooctadiene(η⁵-2-methyl-3-phenylinden-1H-yl)rhodium(I)(±-**S8**):

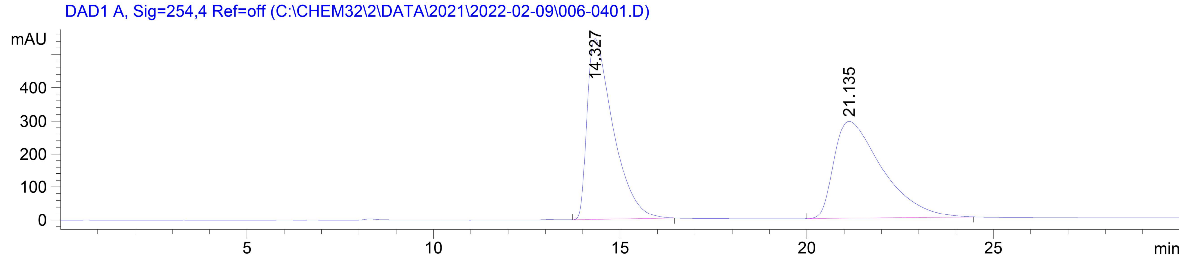

Signal 1: DAD1 A, Sig=254,4 Ref=off

Peak #	RetTime [min]	Type	Width [min]	Area [mAU*s]	Height [mAU]	Area %
1	14.327	BB	0.7227	2.65512e4	546.65851	50.0758
2	21.135	BB	1.3366	2.64708e4	293.38287	49.9242

Totals : 5.30221e4 840.04138

(S)-1,5-cyclooctadiene(η⁵-2-methyl-3-phenylinden-1H-yl)rhodium(I) ((***S***)-**S8**):

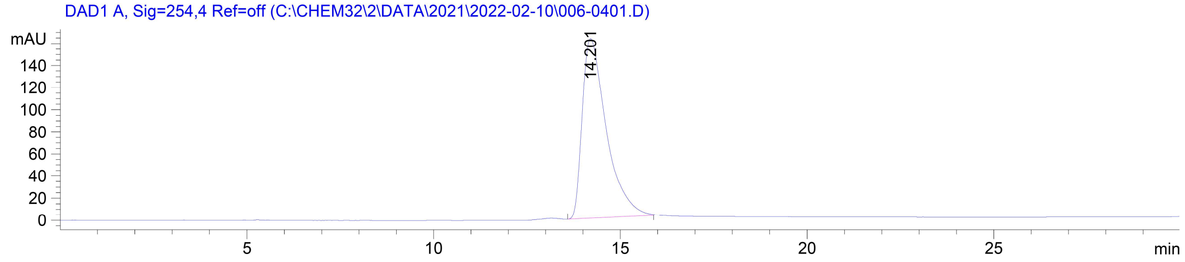

Signal 1: DAD1 A, Sig=254,4 Ref=off

Peak #	RetTime [min]	Type	Width [min]	Area [mAU*s]	Height [mAU]	Area %
1	14.201	BB	0.6687	7259.47119	162.66116	100.0000

Totals : 7259.47119 162.66116

(R)-1,5-cyclooctadiene(η^{5}-2-methyl-3-phenylinden-1H-yl)rhodium(I) (***(R)*-S8**):

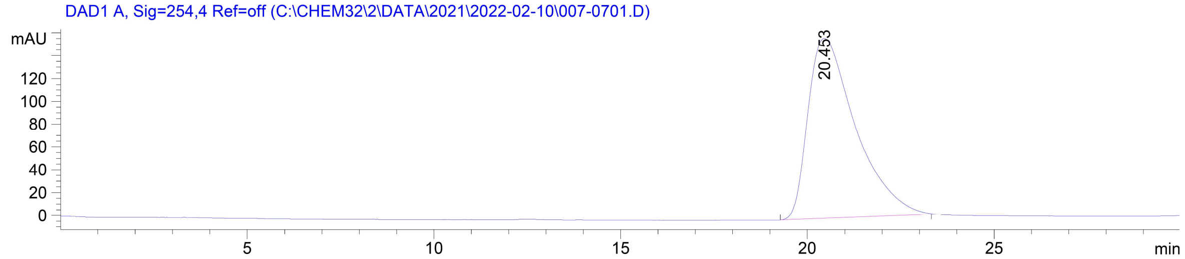

Signal 1: DAD1 A, Sig=254,4 Ref=off

Peak #	RetTime [min]	Type	Width [min]	Area [mAU*s]	Height [mAU]	Area %
1	20.453	BB	1.3022	1.39726e4	157.62514	100.0000

Totals : 1.39726e4 157.62514

(±)-1,5-cyclooctadiene(η⁵-2-methyl-3-(4-(trifluoromethyl)phenyl)inden-1H-yl)rhodium(I) (±-**S9**):

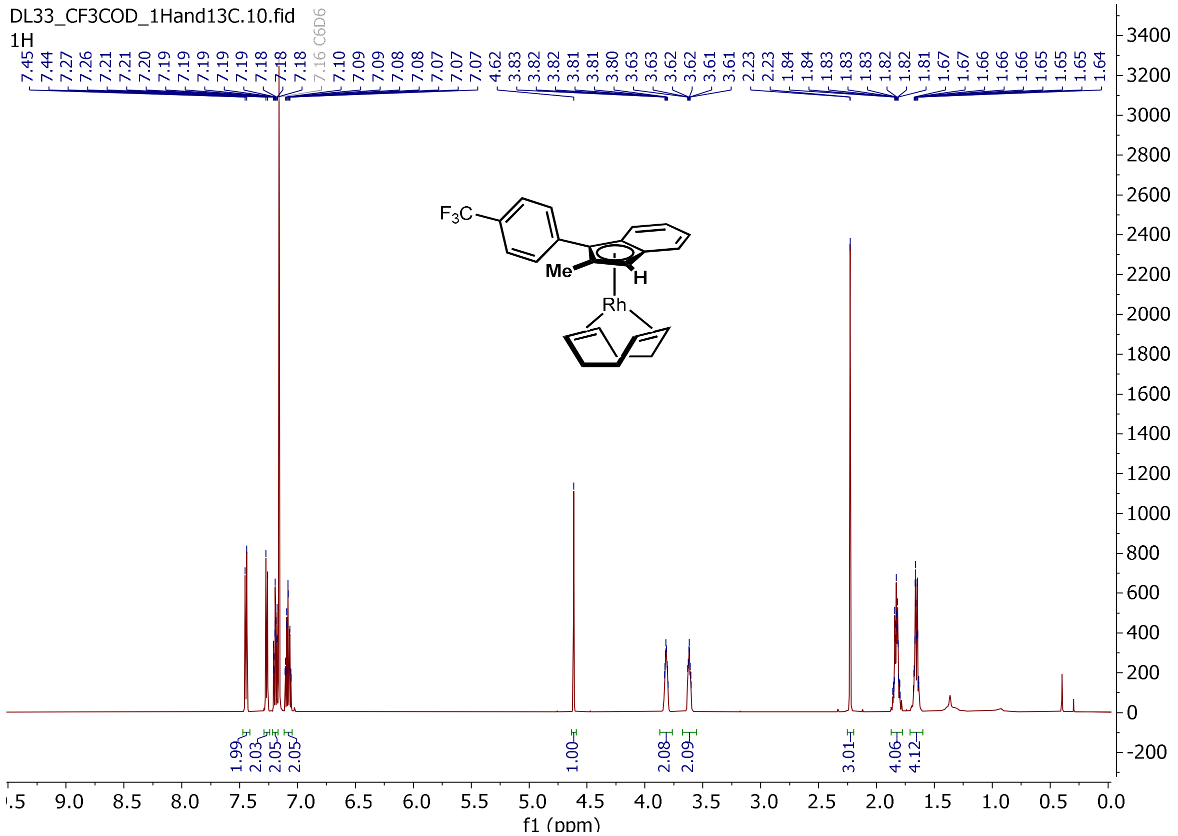

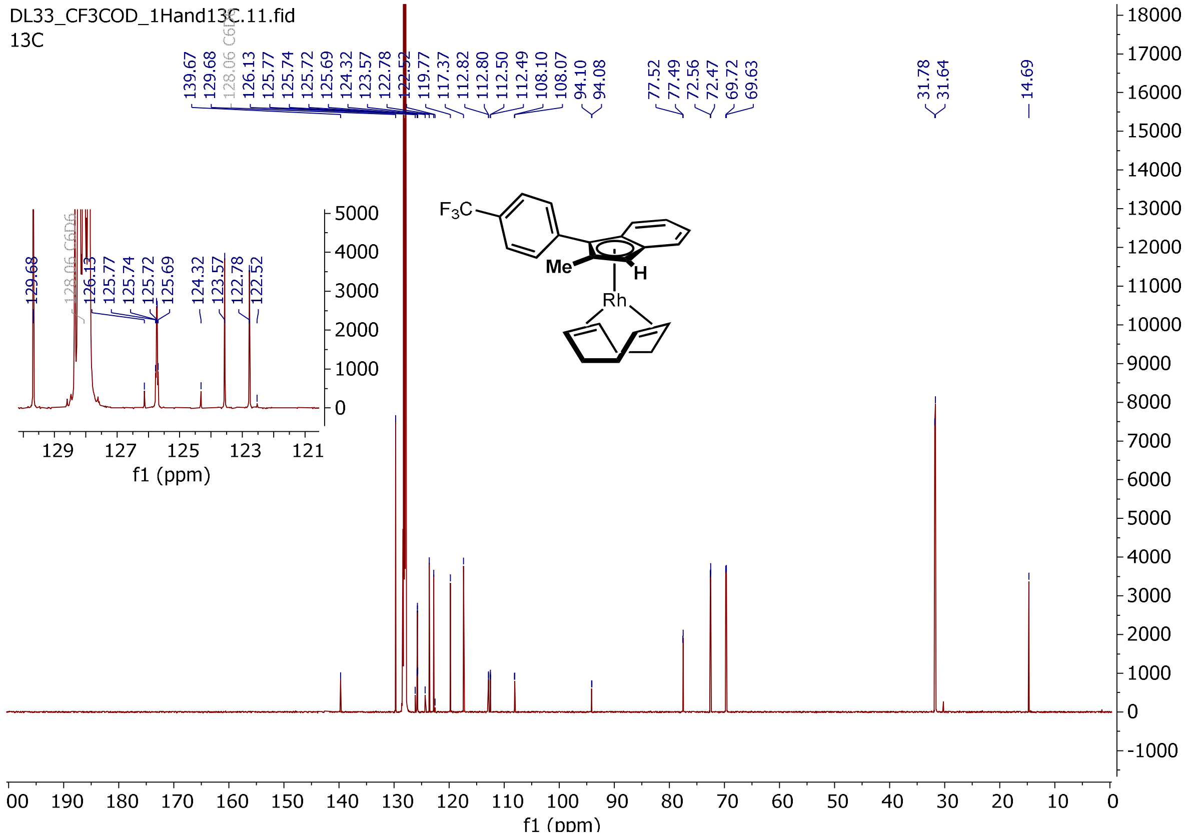

(±)-1,5-cyclooctadiene(η⁵-2-methyl-3-(4-(trifluoromethyl)phenyl)inden-1H-yl)rhodium(I) (±-**S9**):

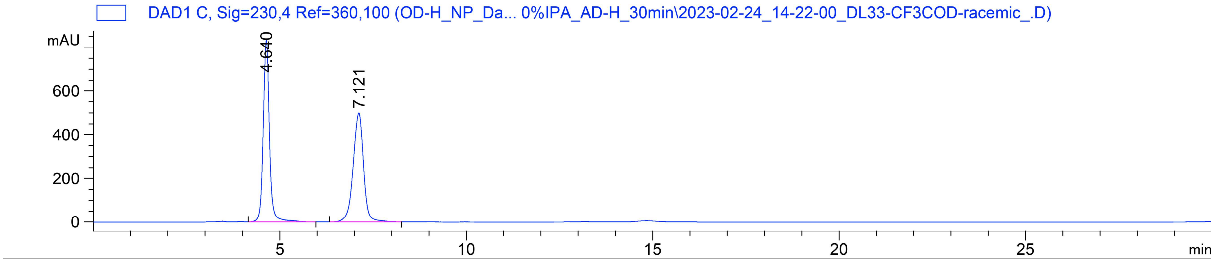

Signal 3: DAD1 C, Sig=230,4 Ref=360,100

Peak #	RetTime [min]	Type	Width [min]	Area [mAU*s]	Height [mAU]	Area %
1	4.640	BB	0.1779	9718.68945	831.37299	50.1056
2	7.121	BB	0.2947	9677.72656	499.10626	49.8944
Totals :				1.93964e4	1330.47925	

(S)-1,5-cyclooctadiene(η⁵-2-methyl-3-(4-(trifluoromethyl)phenyl)inden-1H-yl)rhodium(I) (***(S)*-S9**):

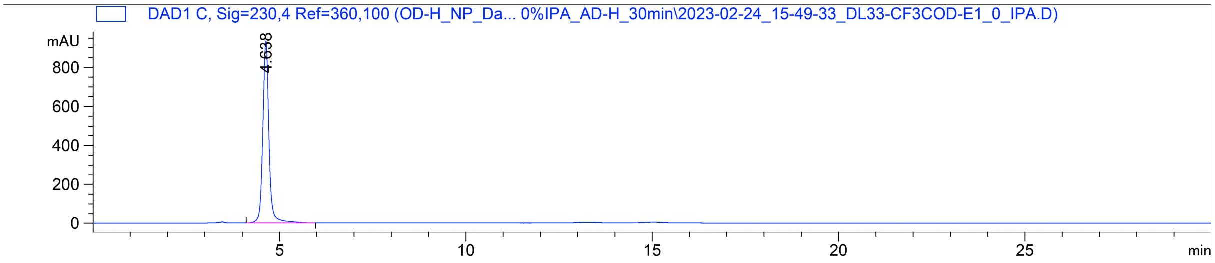

Signal 3: DAD1 C, Sig=230,4 Ref=360,100

Peak #	RetTime [min]	Type	Width [min]	Area [mAU*s]	Height [mAU]	Area %
1	4.638	BB	0.1743	1.07581e4	934.78265	100.0000
Totals :				1.07581e4	934.78265	

(R)-1,5-cyclooctadiene(η^5-2-methyl-3-(4-(trifluoromethyl)phenyl)inden-1H-yl)rhodium(I) (***(R)*-S9**):

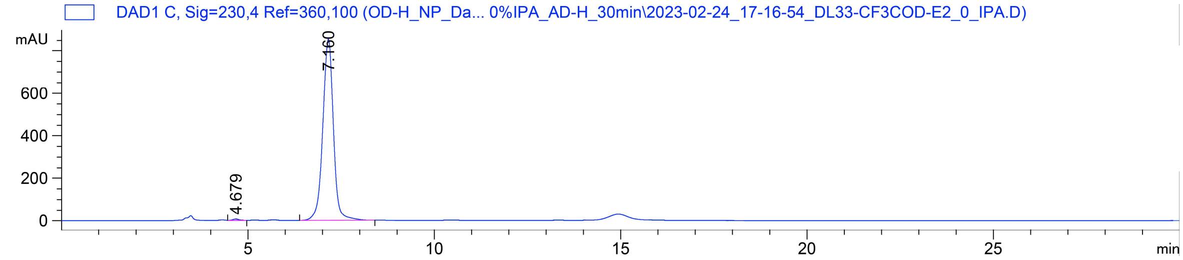

Signal 3: DAD1 C, Sig=230,4 Ref=360,100

Peak #	RetTime [min]	Type	Width [min]	Area [mAU*s]	Height [mAU]	Area %
1	4.679	VB	0.1404	74.39933	6.50820	0.4384
2	7.160	BB	0.3026	1.68954e4	854.68109	99.5616

Totals : 1.69698e4 861.18930

(±)-1,5-cyclooctadiene(η^5-3-(4-methoxyphenyl)-2-methylinden-1H-yl)rhodium(I) (±-**S10**):

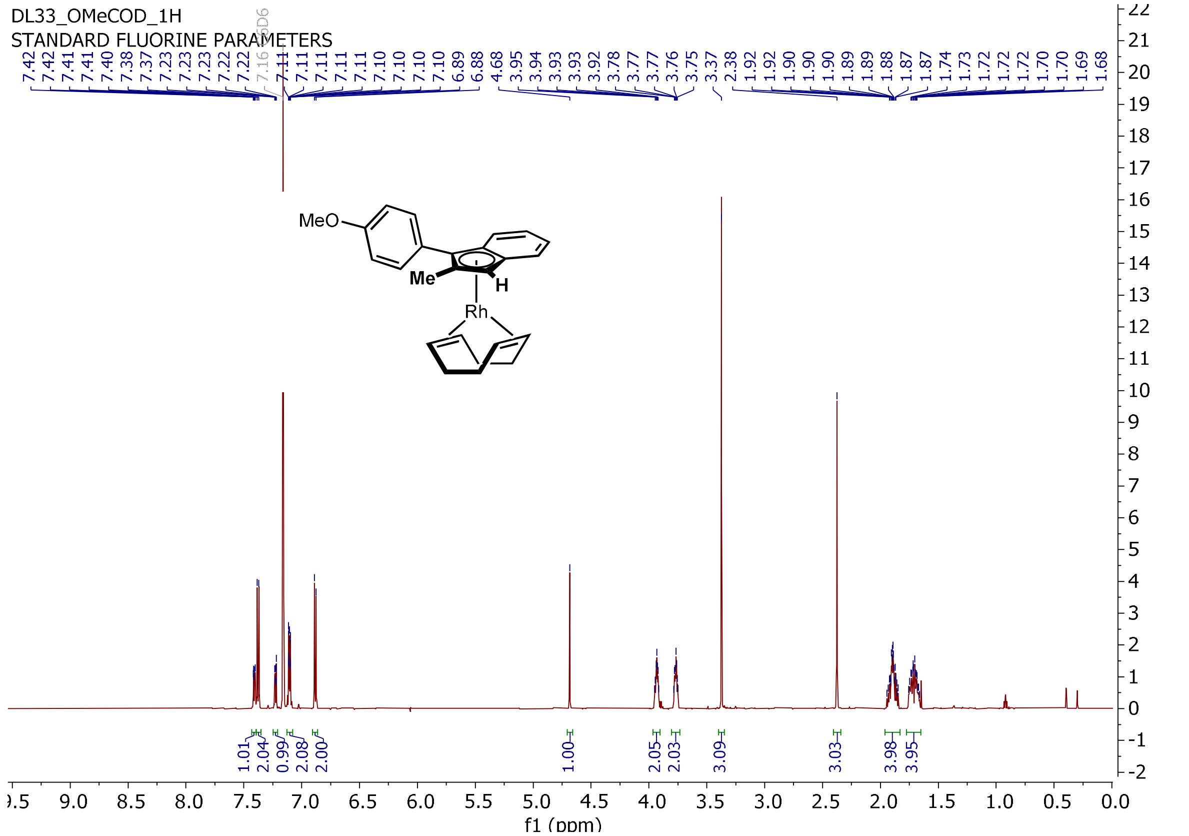

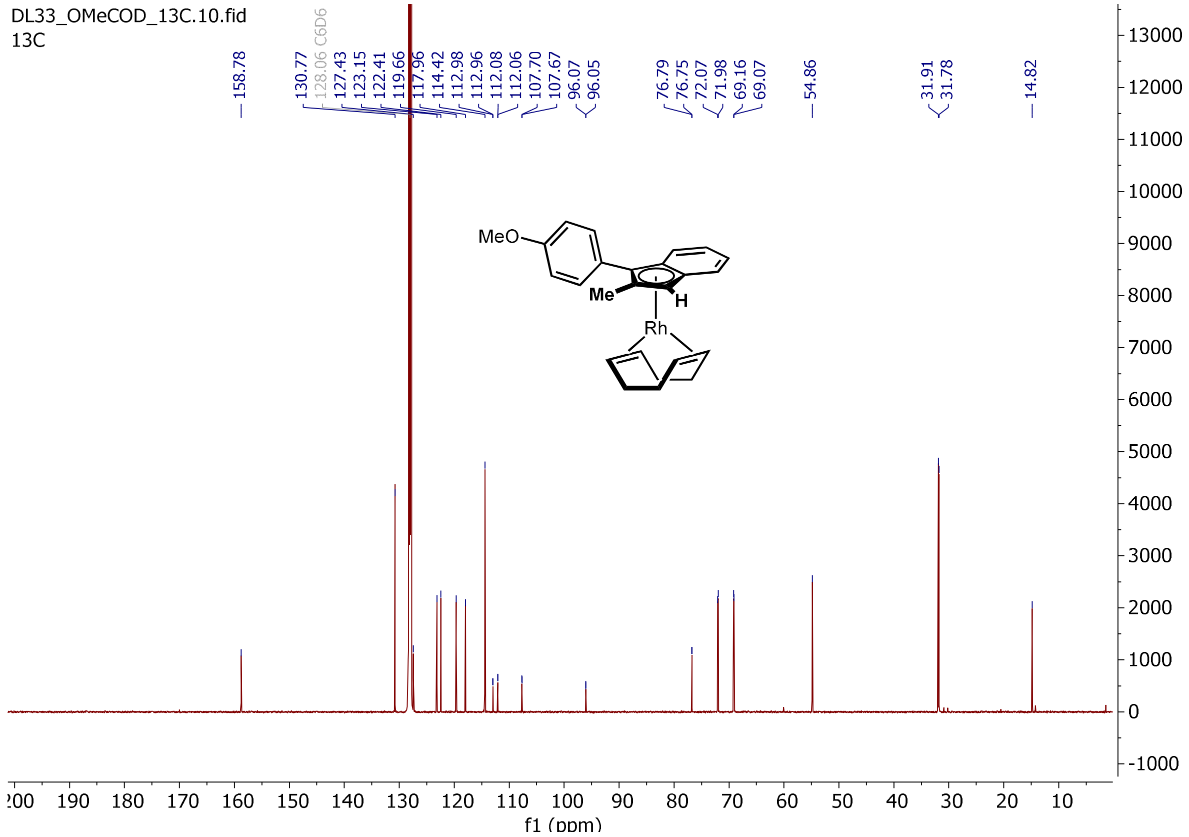

(±)-1,5-cyclooctadiene(η^5-3-(4-methoxyphenyl)-2-methylinden-1H-yl)rhodium(I) (±-**S10**):

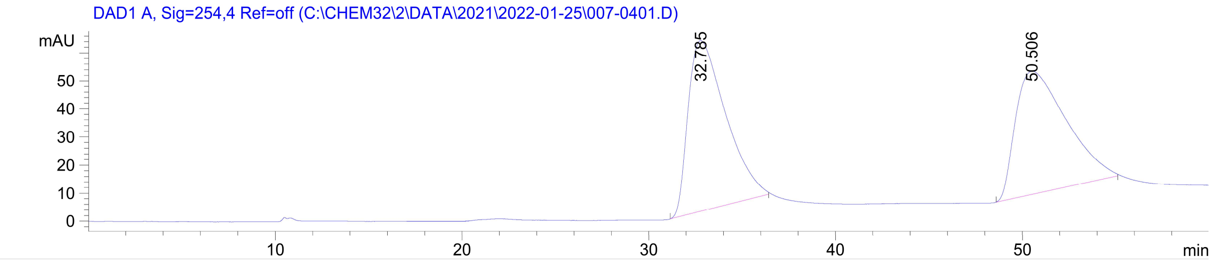

Signal 1: DAD1 A, Sig=254,4 Ref=off

Peak #	RetTime [min]	Type	Width [min]	Area [mAU*s]	Height [mAU]	Area %
1	32.785	BB	1.9017	8497.05078	60.90253	50.7854
2	50.506	BB	2.2075	8234.23340	43.87610	49.2146

Totals : 1.67313e4 104.77863

(S)-1,5-cyclooctadiene(η^5-3-(4-methoxyphenyl)-2-methylinden-1H-yl)rhodium(I) (**(*S*)-S10**):

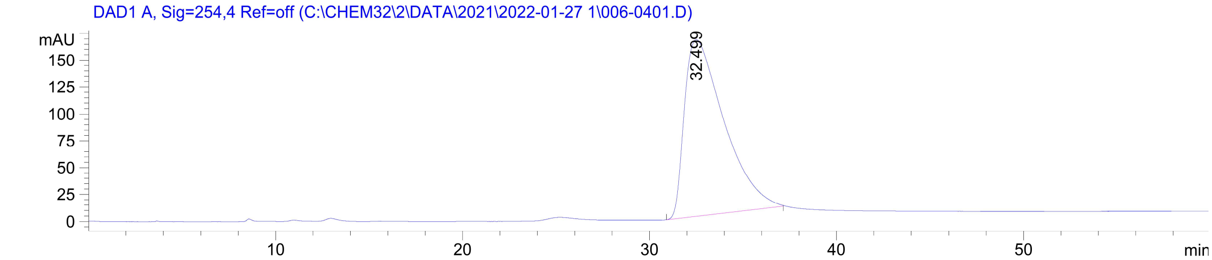

Signal 1: DAD1 A, Sig=254,4 Ref=off

Peak #	RetTime [min]	Type	Width [min]	Area [mAU*s]	Height [mAU]	Area %
1	32.499	BB	2.0821	2.42883e4	164.54539	100.0000

Totals : 2.42883e4 164.54539

(R)-1,5-cyclooctadiene(η^5-3-(4-methoxyphenyl)-2-methylinden-1H-yl)rhodium(I) (***(R)*-S10**):

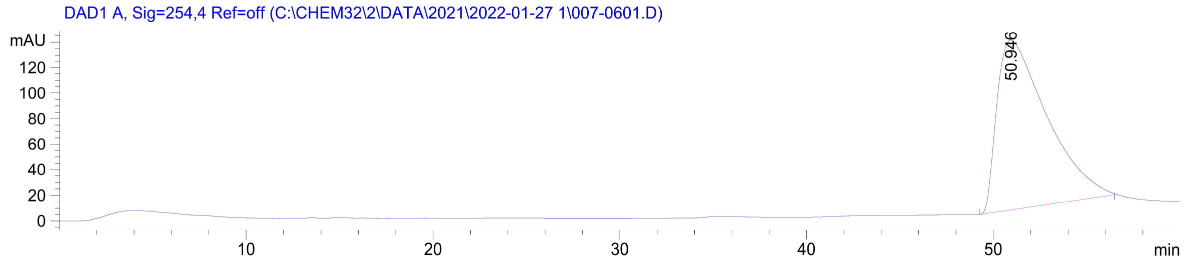

Signal 1: DAD1 A, Sig=254,4 Ref=off

Peak #	RetTime [min]	Type	Width [min]	Area [mAU*s]	Height [mAU]	Area %
1	50.946	BB	2.4440	2.47072e4	133.08168	100.0000

Totals : 2.47072e4 133.08168

(±)-1,5-cyclooctadiene(η⁵-3-(4-(tert-butyl)phenyl-2-methylinden-1H-yl)rhodium(I) (±-**S11**):

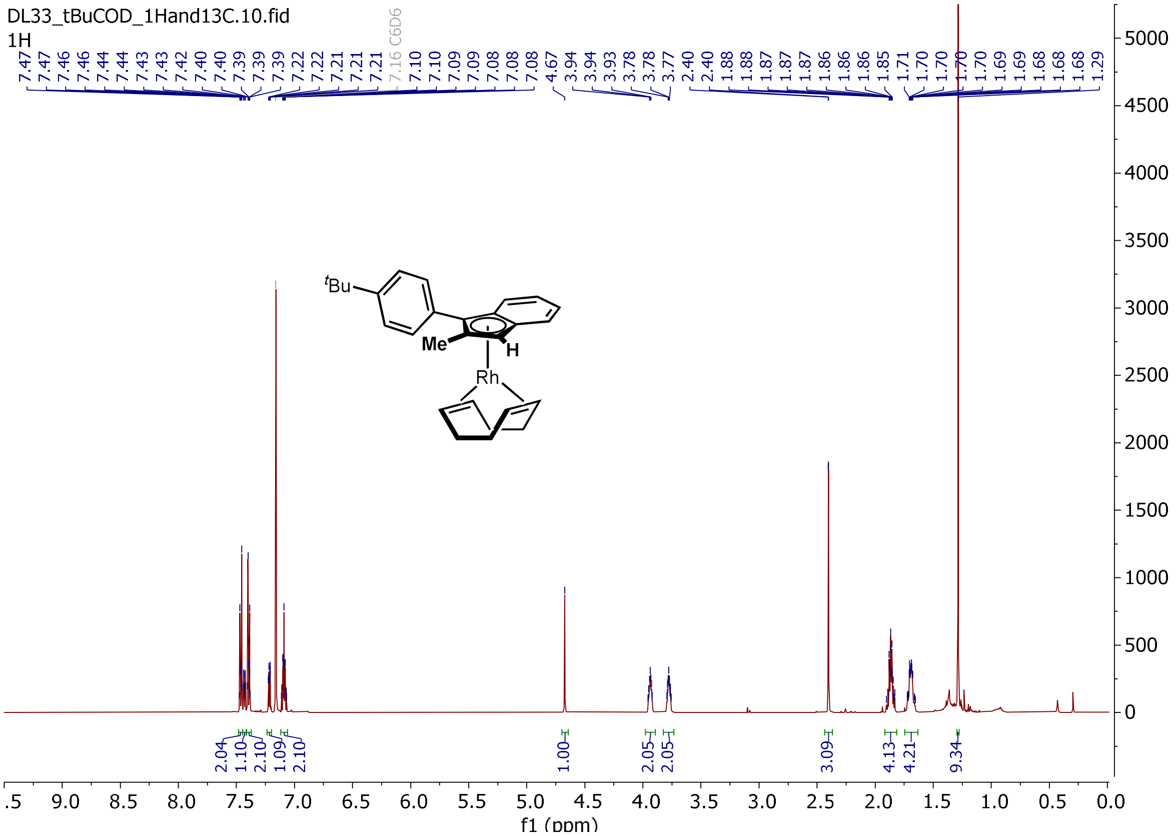

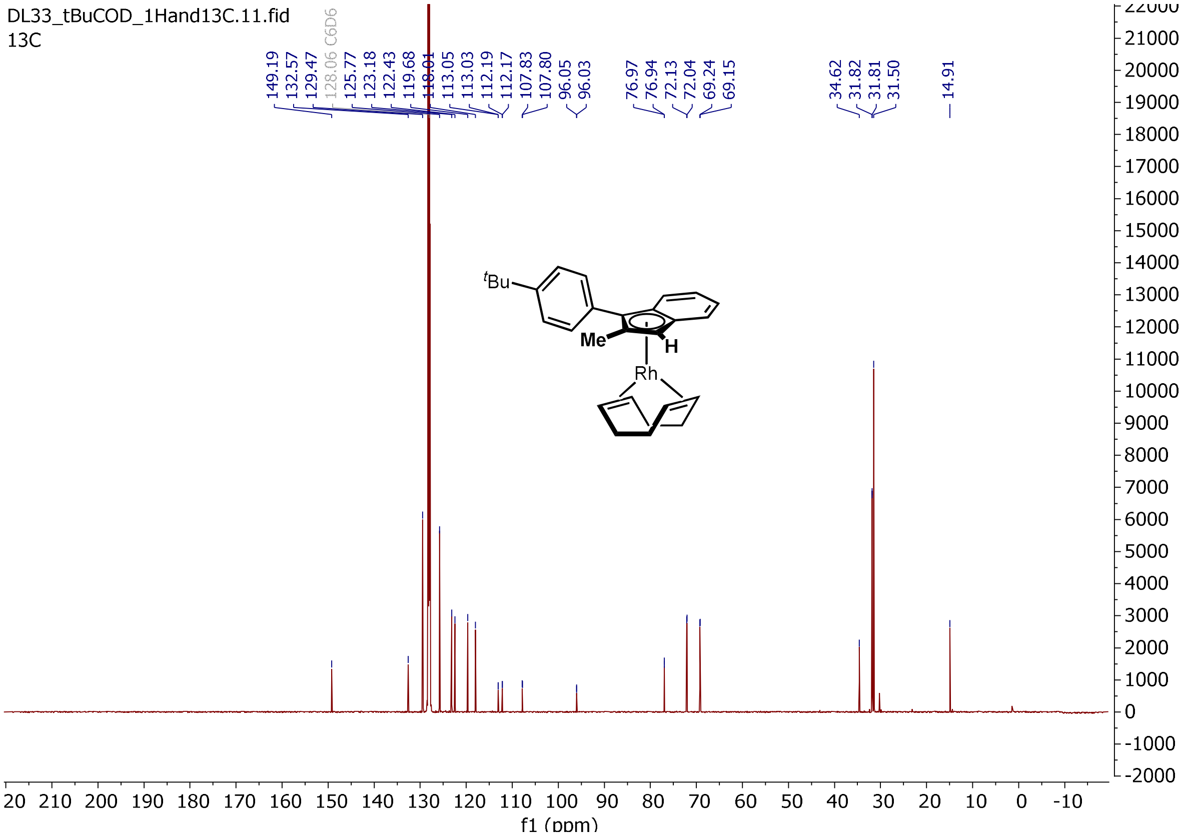

(±)-1,5-cyclooctadiene(η^5-3-(4-(tert-butyl)phenyl-2-methylinden-1H-yl)rhodium(I) (±-**S11**):

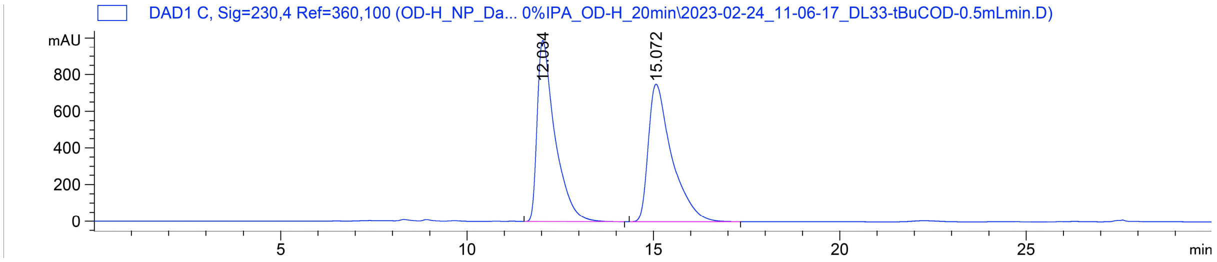

Signal 3: DAD1 C, Sig=230,4 Ref=360,100

Peak #	RetTime [min]	Type	Width [min]	Area [mAU*s]	Height [mAU]	Area %
1	12.034	BB	0.4709	3.27591e4	989.06738	49.9359
2	15.072	BB	0.5844	3.28431e4	750.77612	50.0641
Totals :				6.56021e4	1739.84351	

(S)-1,5-cyclooctadiene(η^5-3-(4-(tert-butyl)phenyl-2-methylinden-1H-yl)rhodium(I) ((***S***)-**S11**):

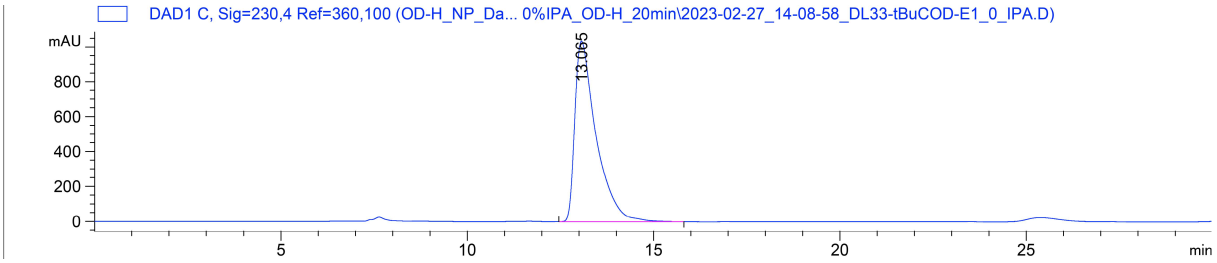

Signal 3: DAD1 C, Sig=230,4 Ref=360,100

Peak #	RetTime [min]	Type	Width [min]	Area [mAU*s]	Height [mAU]	Area %
1	13.065	BB	0.5320	4.10621e4	1035.77551	100.0000
Totals :				4.10621e4	1035.77551	

(R)-1,5-cyclooctadiene(η^5-3-(4-(tert-butyl)phenyl-2-methylinden-1H-yl)rhodium(I) (***(R)*-S11**):

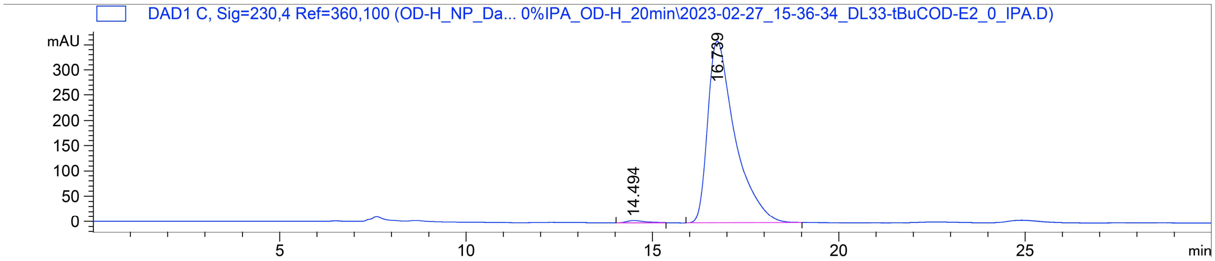

Signal 3: DAD1 C, Sig=230,4 Ref=360,100

Peak #	RetTime [min]	Type	Width [min]	Area [mAU*s]	Height [mAU]	Area %
1	14.494	BB	0.3794	147.94838	4.57352	0.8020
2	16.739	BB	0.6538	1.83004e4	360.19049	99.1980

Totals : 1.84484e4 364.76401

(±)-1,5-cyclooctadiene(η^5-2,4,5,6,7-pentamethyl-3-phenylinden-1H-yl)rhodium(I)(±-**S12**).

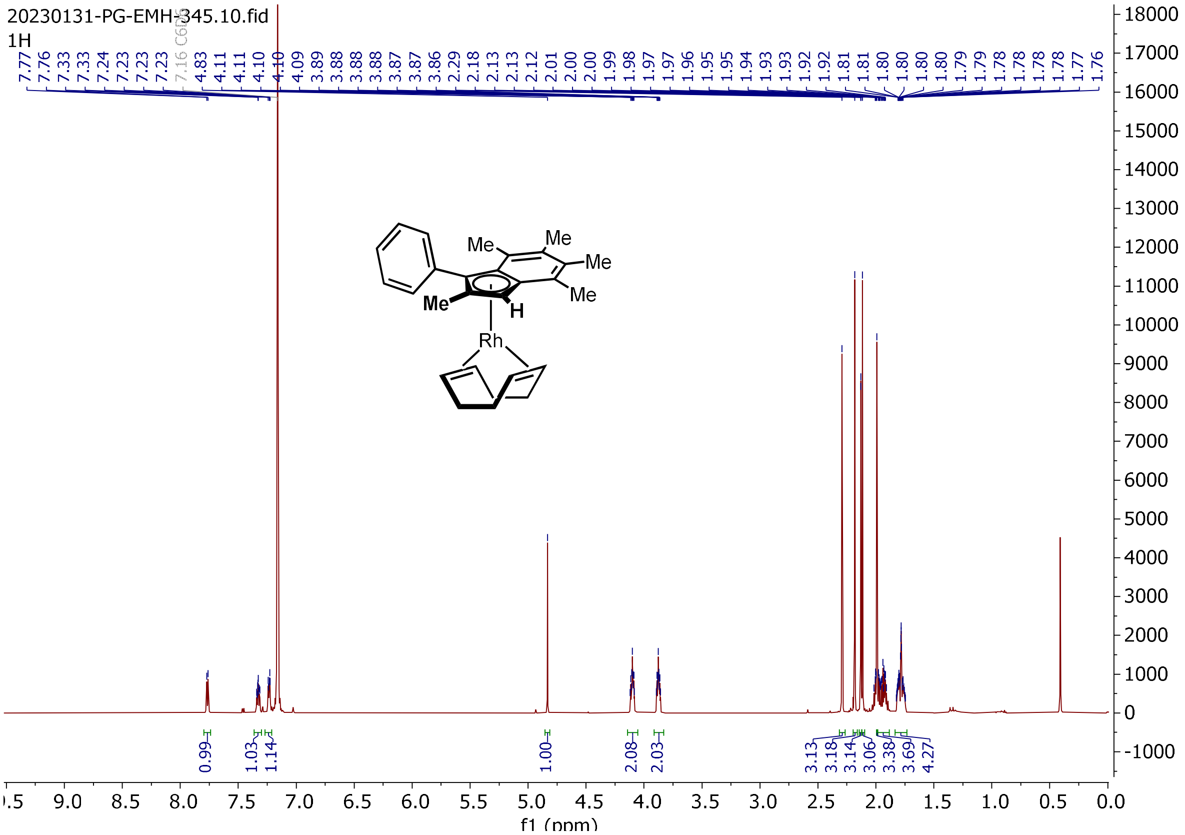

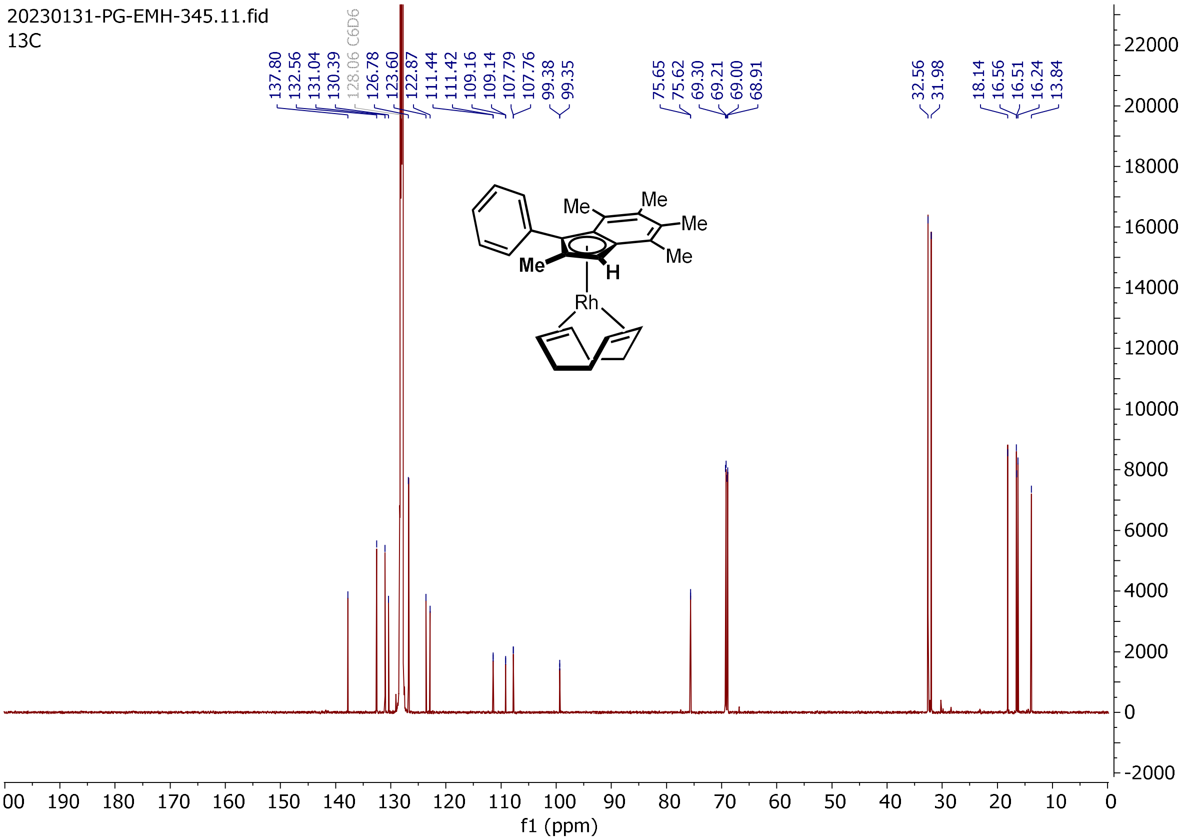

(±)-1,5-cyclooctadiene(η^5-2,4,5,6,7-pentamethyl-3-phenylinden-1H-yl)rhodium(I)(±-**S12**).

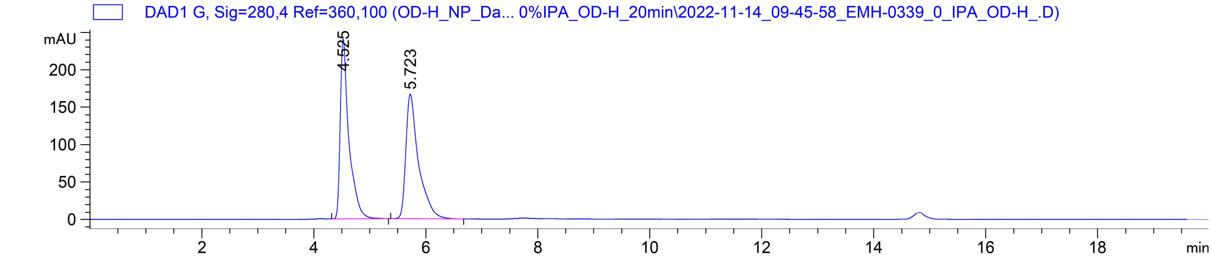

Signal 7: DAD1 G, Sig=280,4 Ref=360,100

Peak #	RetTime [min]	Type	Width [min]	Area [mAU*s]	Height [mAU]	Area %
1	4.525	BB	0.1554	2594.72900	240.36707	49.6225
2	5.723	BB	0.2273	2634.20874	166.34329	50.3775
Totals :				5228.93774	406.71036	

(R)-1,5-cyclooctadiene(η^5-2,4,5,6,7-pentamethyl-3-phenylinden-1H-yl)rhodium(I) ((***R)***-**S12**):

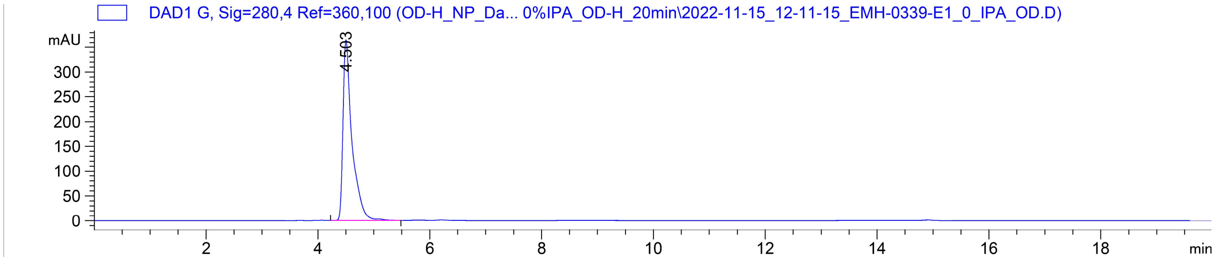

Signal 7: DAD1 G, Sig=280,4 Ref=360,100

Peak #	RetTime [min]	Type	Width [min]	Area [mAU*s]	Height [mAU]	Area %
1	4.503	BV R	0.1599	4102.95166	365.32794	100.0000
Totals :				4102.95166	365.32794	

(S)-1,5-cyclooctadiene(η^5-2,4,5,6,7-pentamethyl-3-phenylinden-1H-yl)rhodium(I) ((***S***)-**S12**):

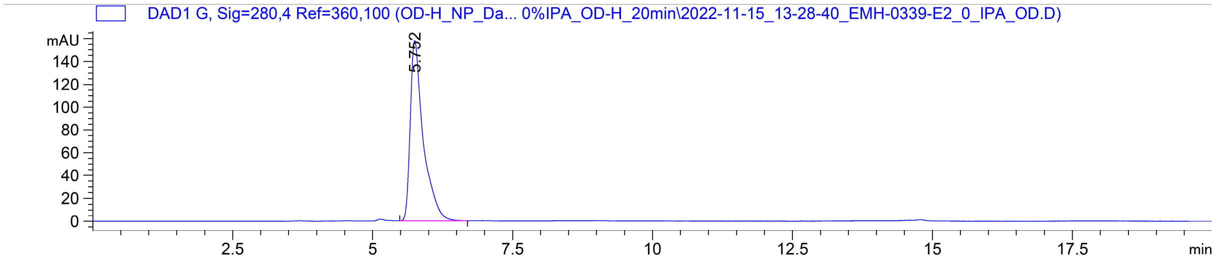

Signal 7: DAD1 G, Sig=280,4 Ref=360,100

Peak #	RetTime [min]	Type	Width [min]	Area [mAU*s]	Height [mAU]	Area %
1	5.752	BB	0.2317	2548.04932	157.92233	100.0000

Totals : 2548.04932 157.92233

(±)-1,5-cyclooctadiene(η⁵-3-(4-(tert-butyl)phenyl)-2,4,5,6,7-pentamethylinden-1H-yl)rhodium(I) (±-**S13**).

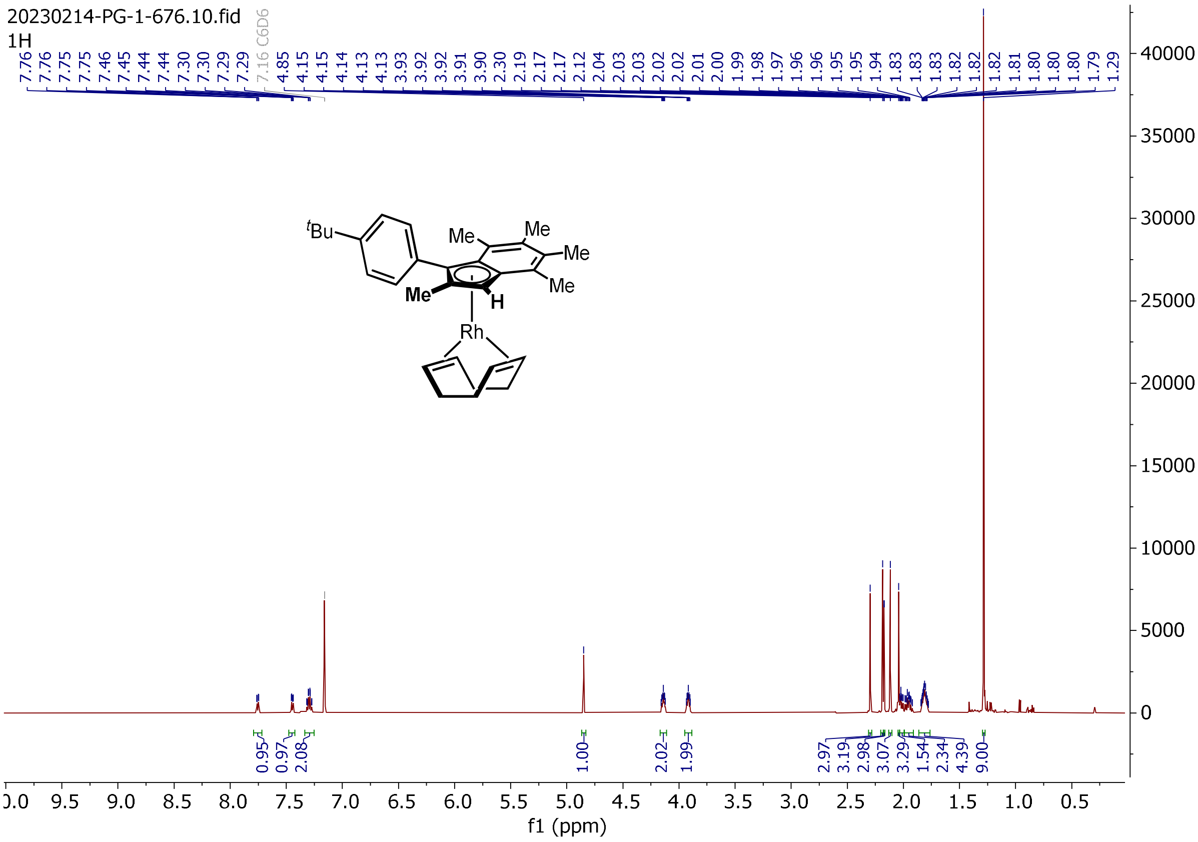

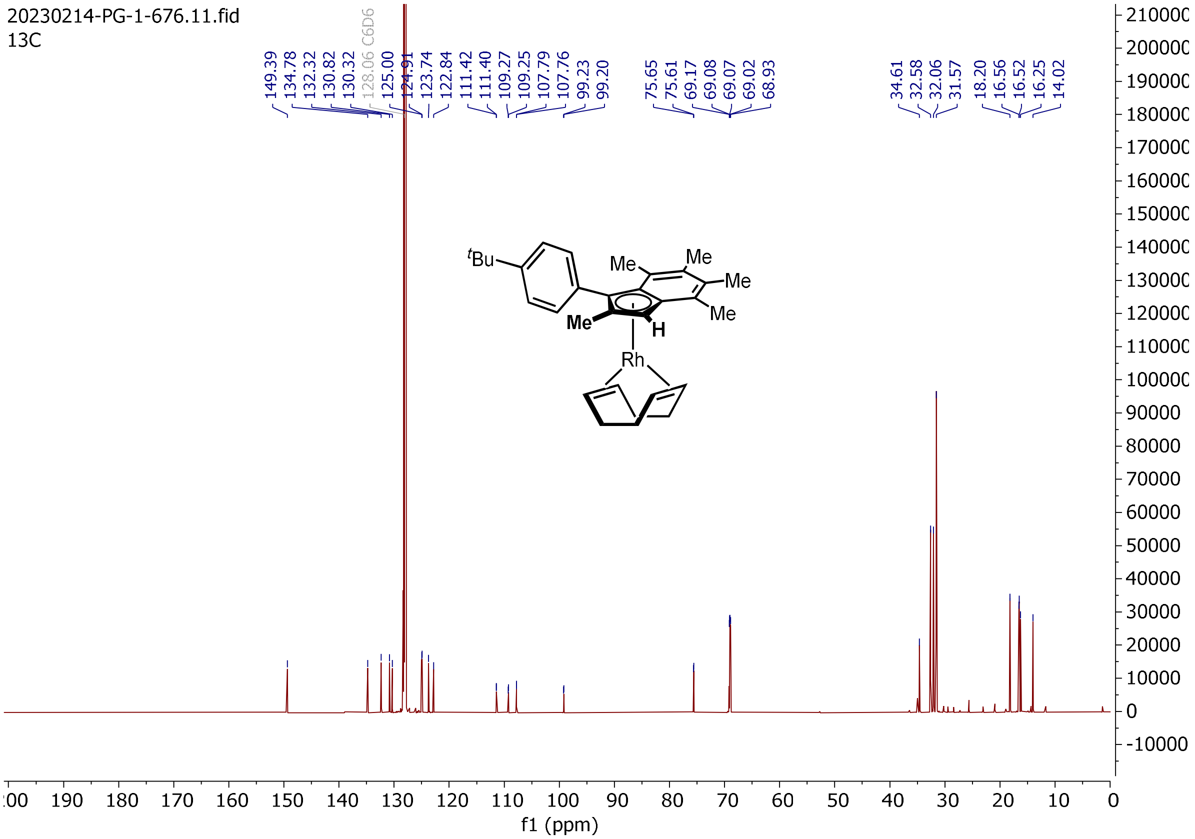

(S,S)-(η[5]-2-methyl-3-(4-(trifluoromethyl)phenyl)inden-1H-yl) rhodium(III) diiodide dimer (**(*S,S*)-S5**):

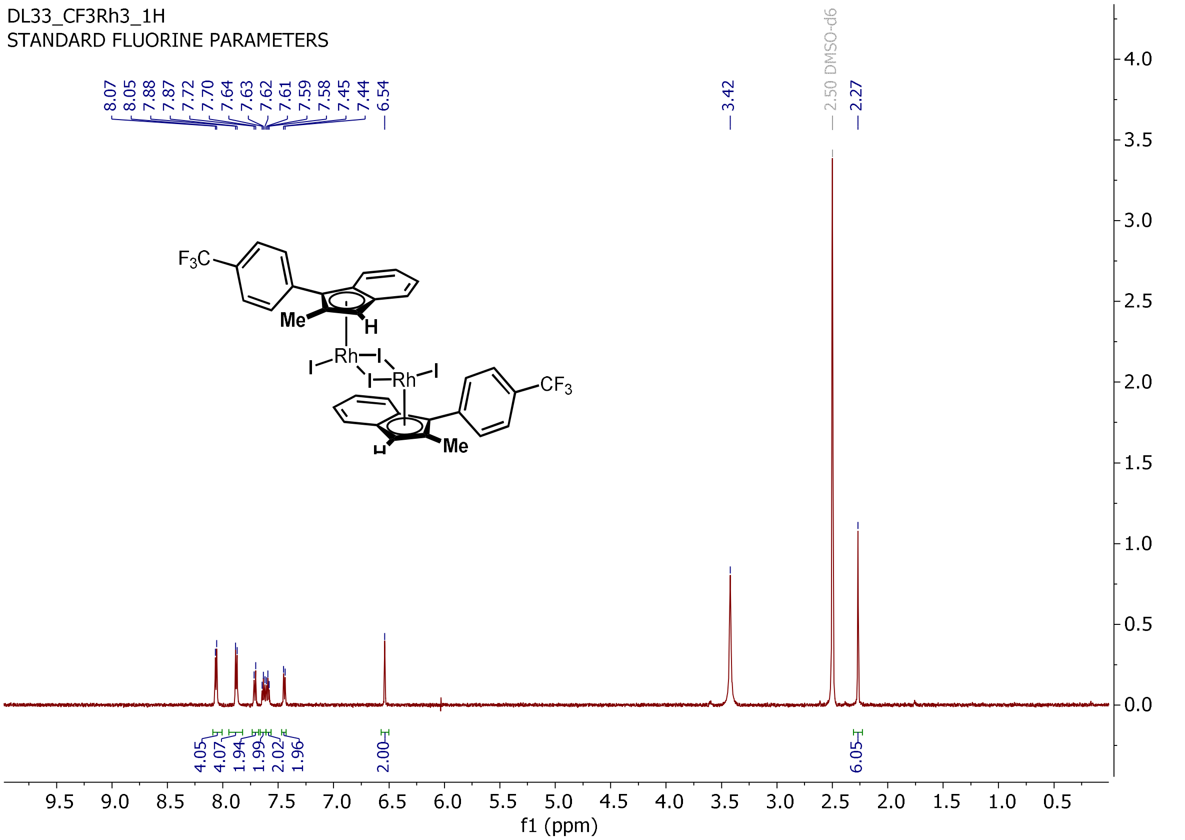

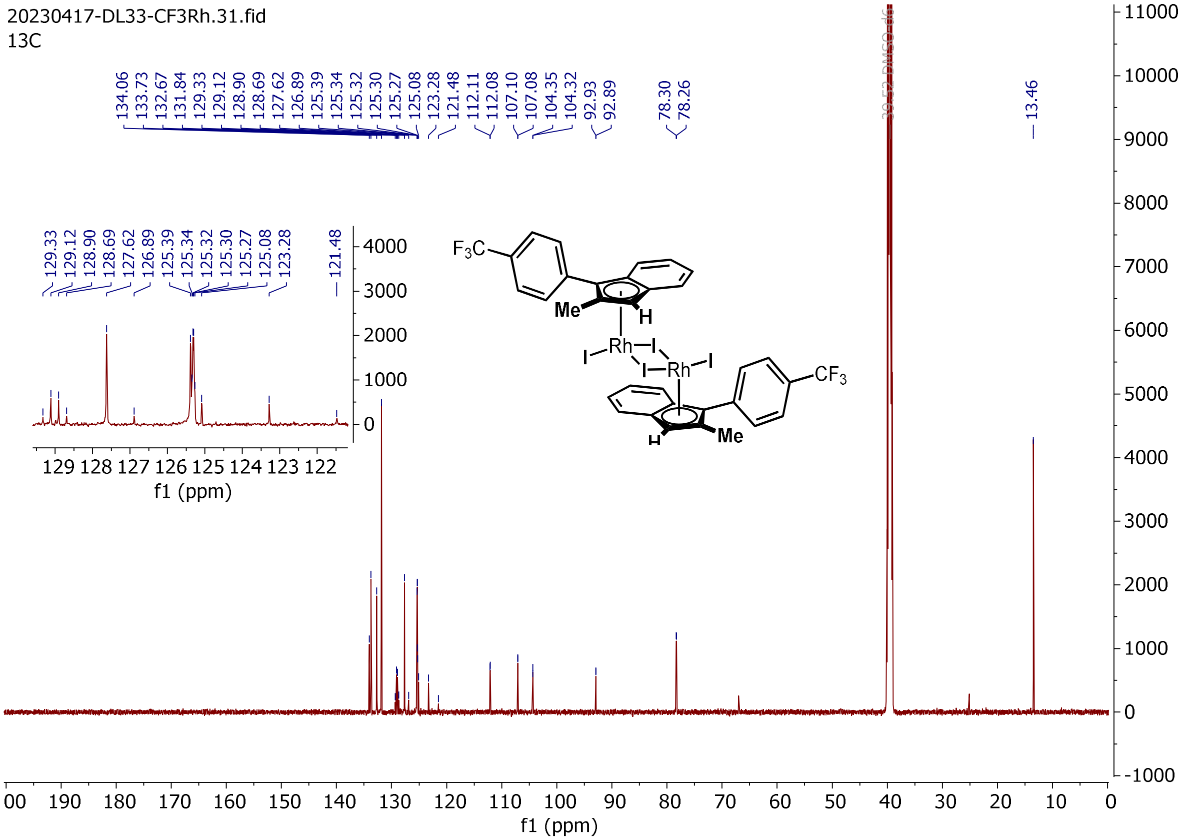

(S,S)-(η[5]-3-(4-methoxyphenyl)-2-methylinden-1H-yl) rhodium(III) diiodide dimer (***(S,S)*-6**):

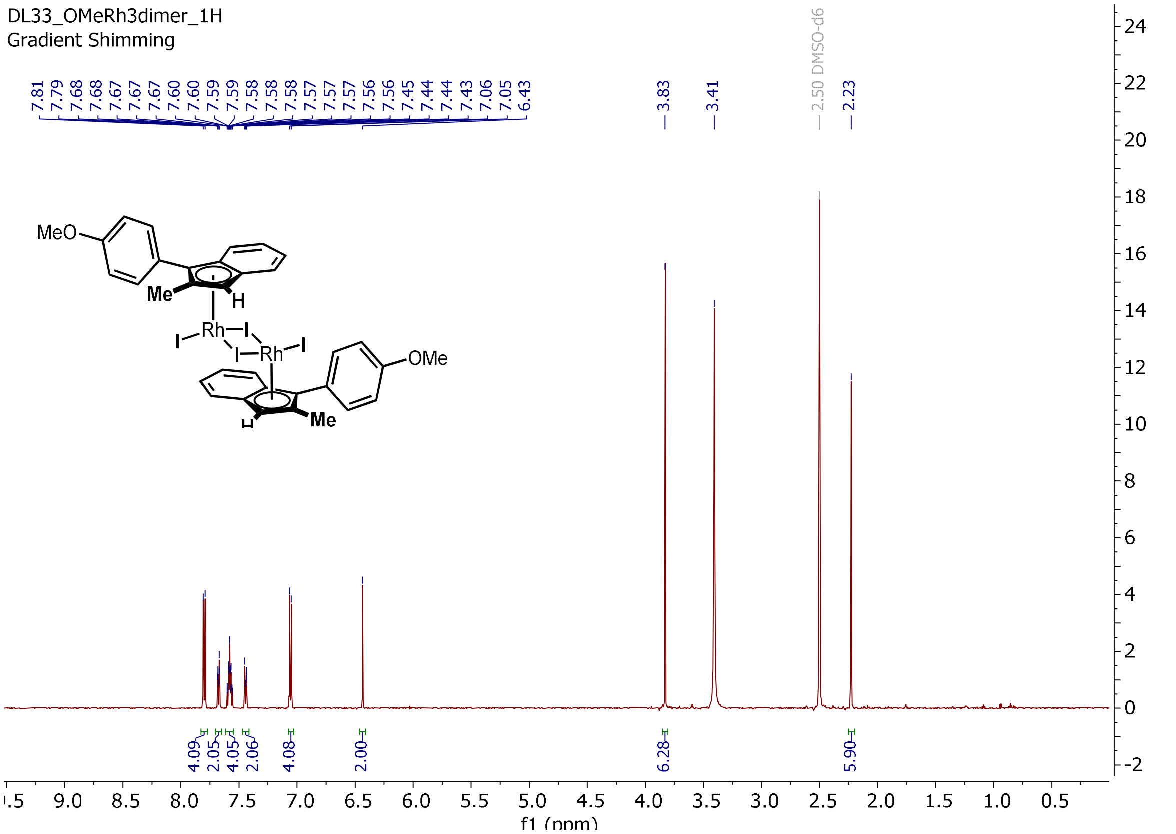

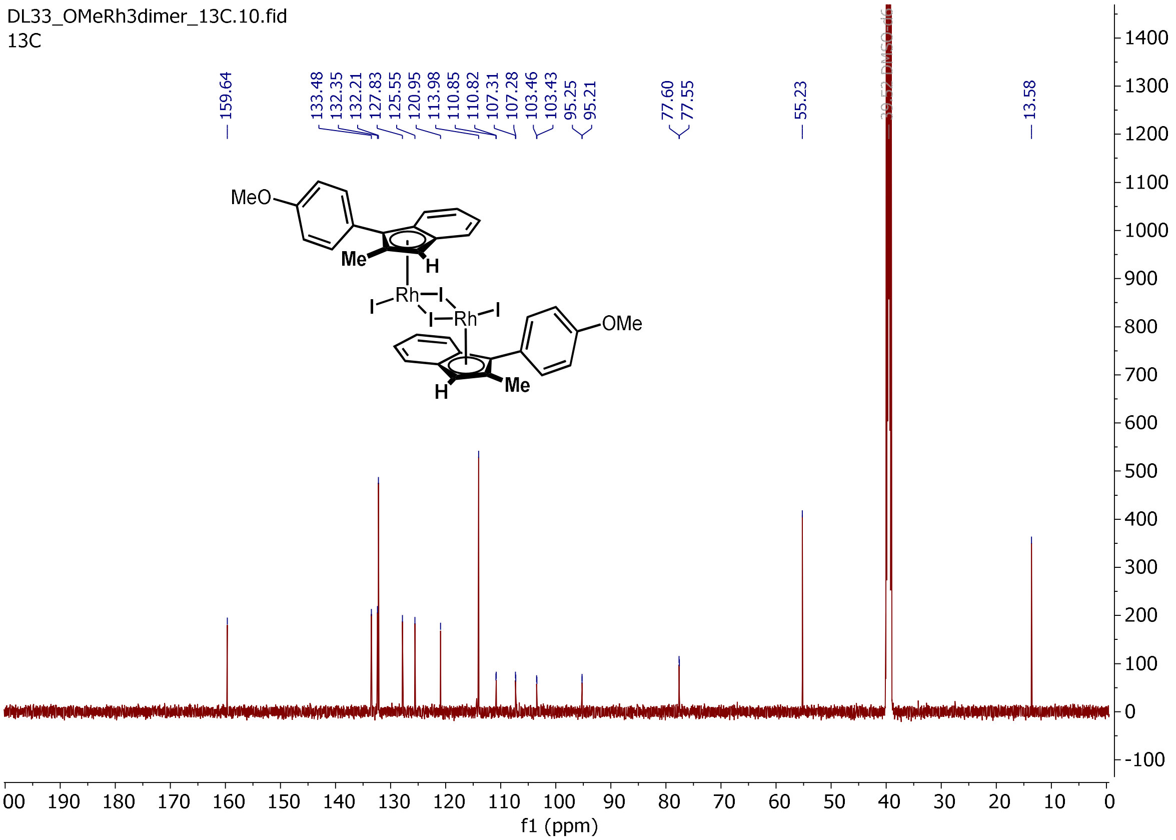

(S,S)-(η⁵-3-(4-(tert-butyl)phenyl-2-methylinden-1H-yl) rhodium(III) diiodide dimer (**(*S,S*)- 7**):

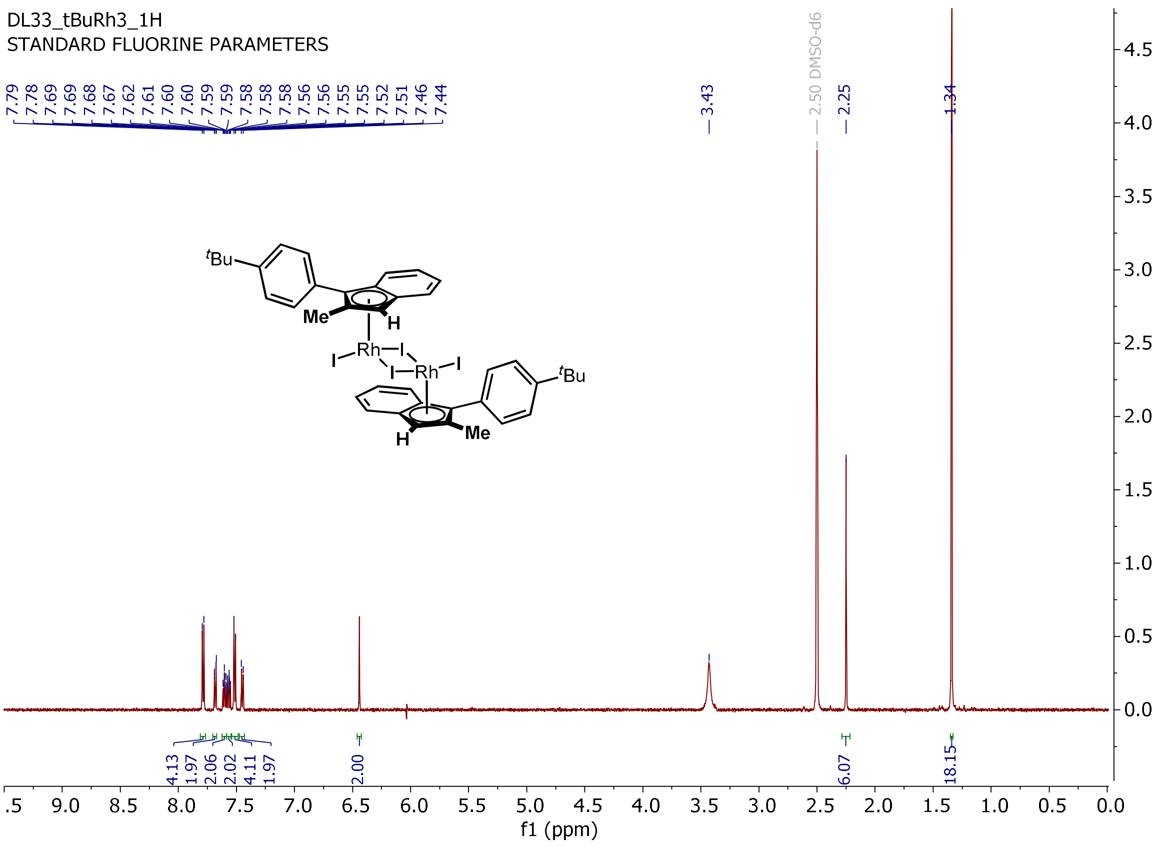

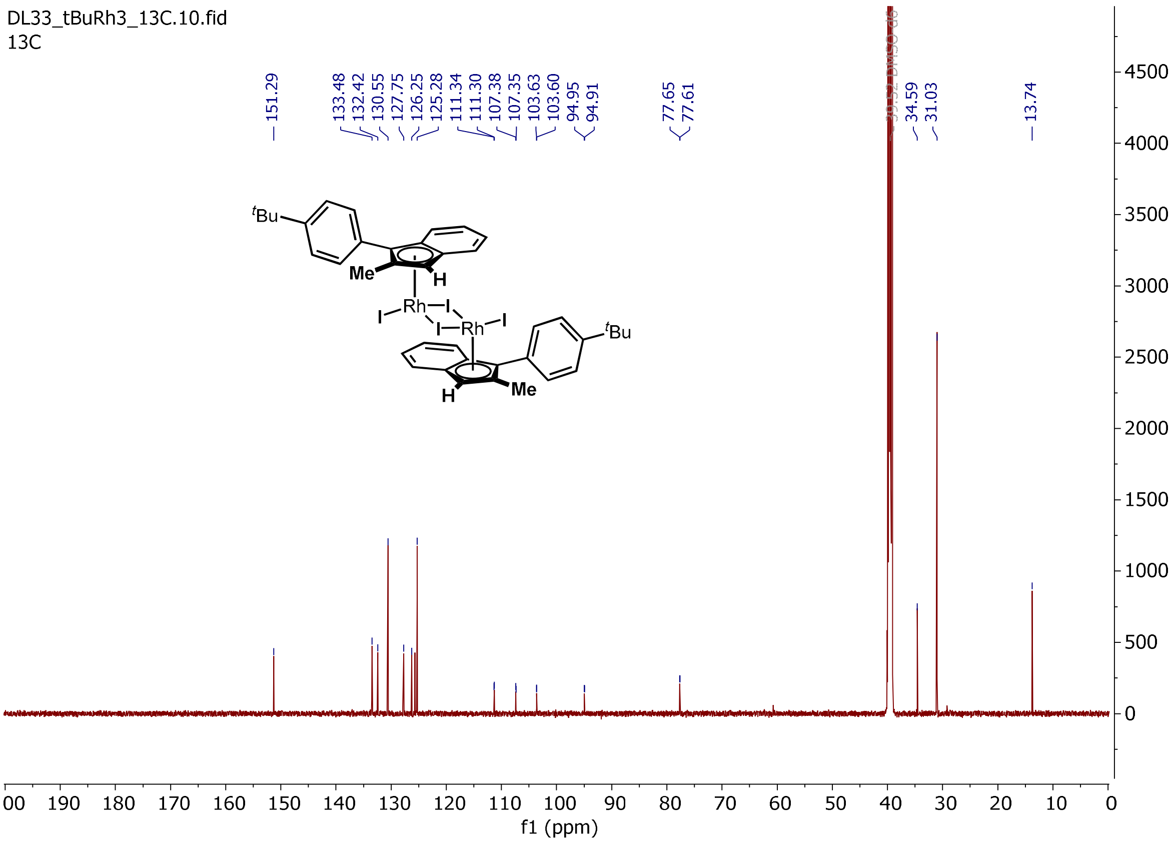

(S,S)-(η^5-2,4,5,6,7-pentamethyl-3-phenylinden-1H-yl) rhodium(III) diiodide dimer (**(*S,S*)-8**):

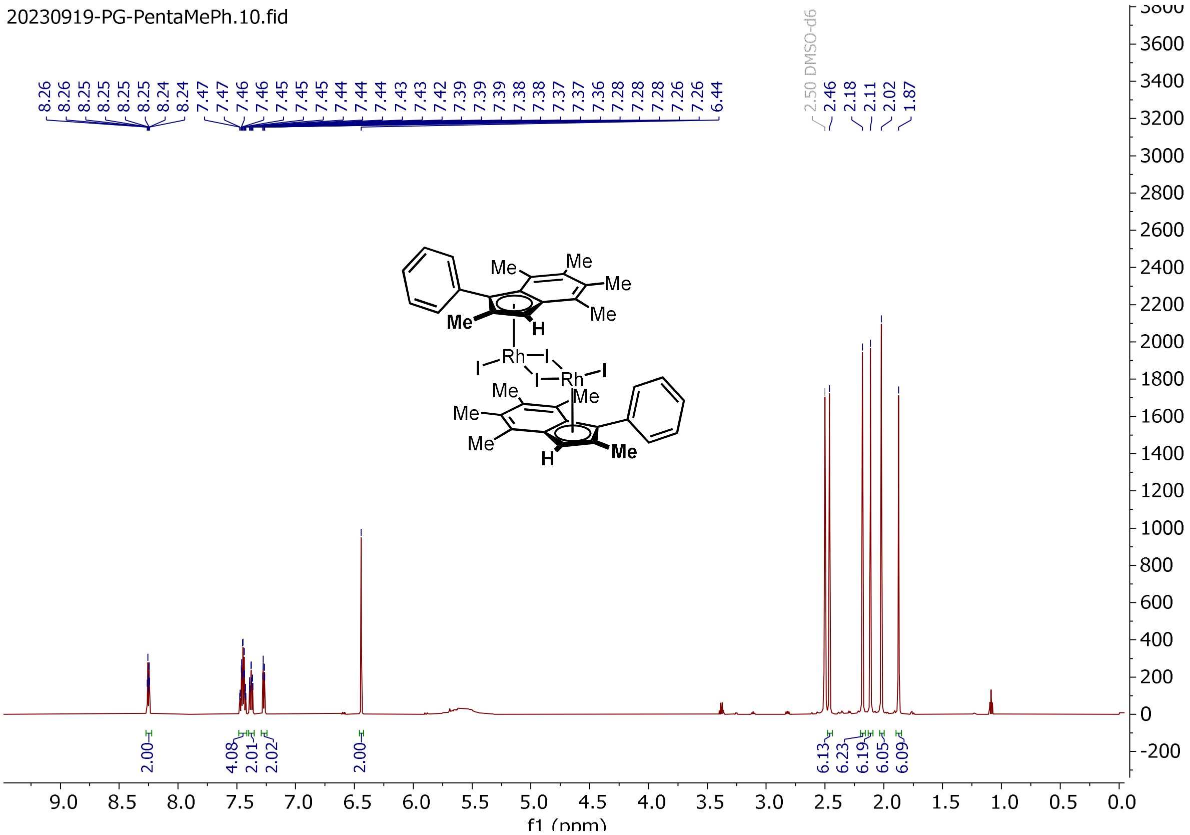

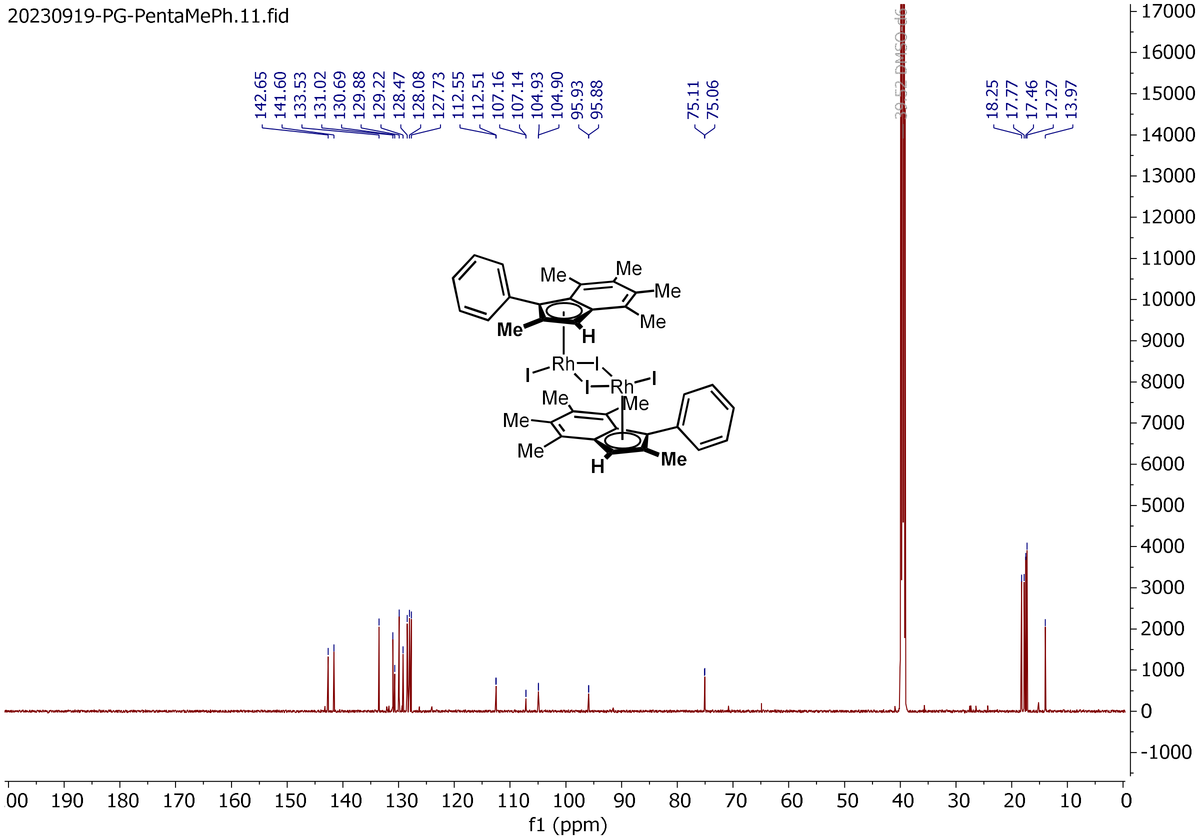

(η^5-3-(4-(tert-butyl)phenyl)-2,4,5,6,7-pentamethylinden-1H-yl) rhodium(III) diiodide dimer (**9**)

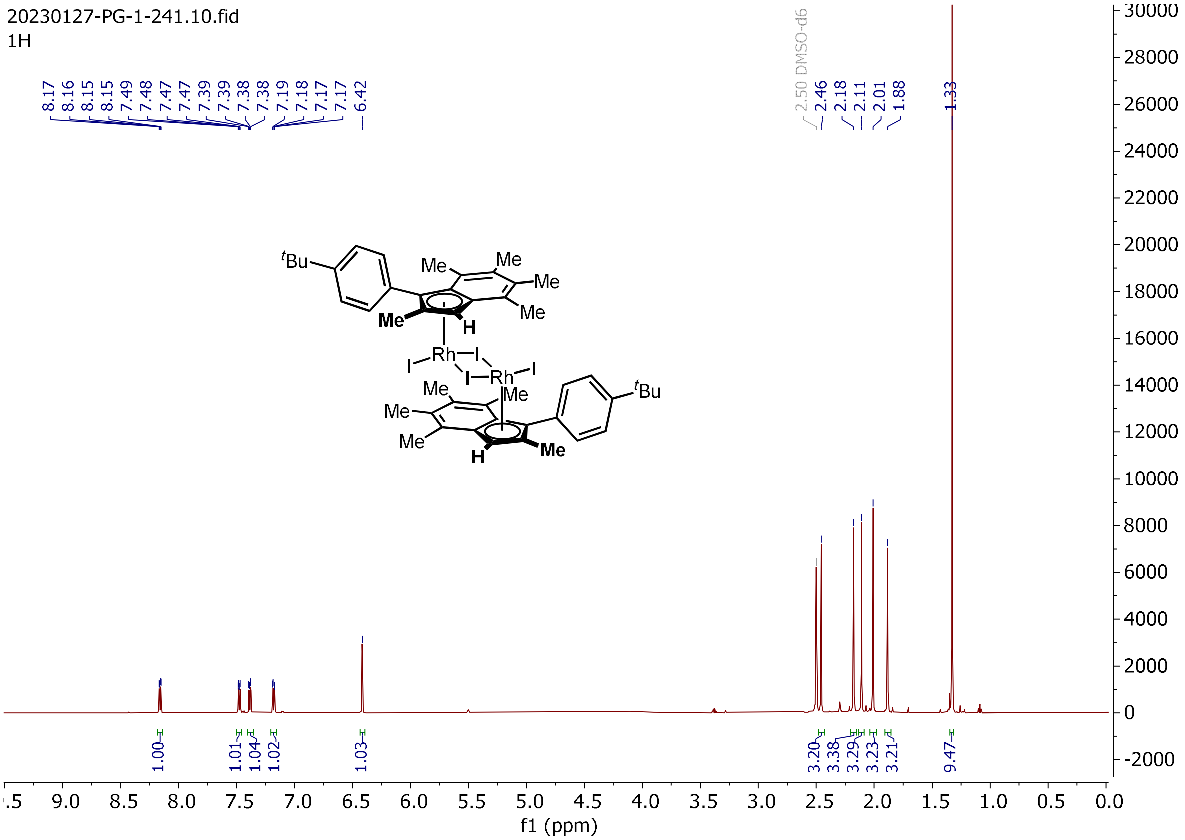

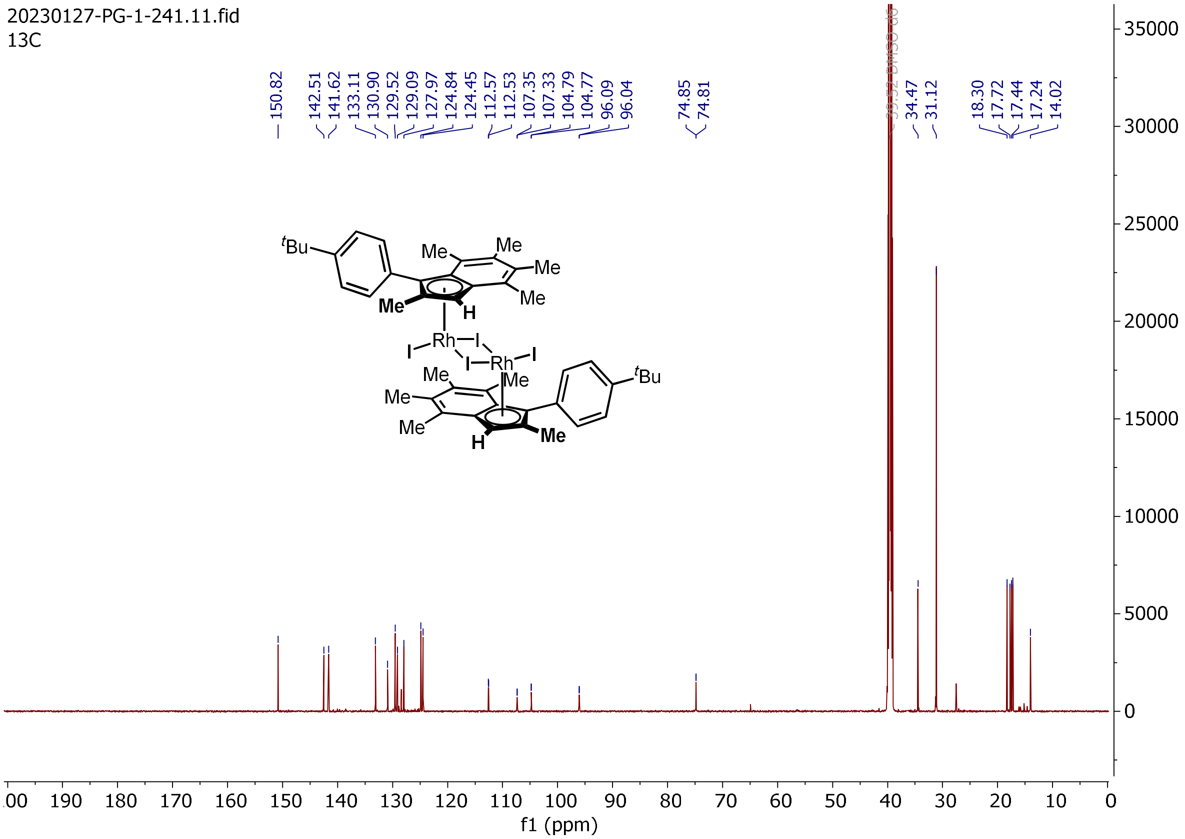

4-nitro-N-(pivaloyloxy)benzenesulfonamide (**S34**):

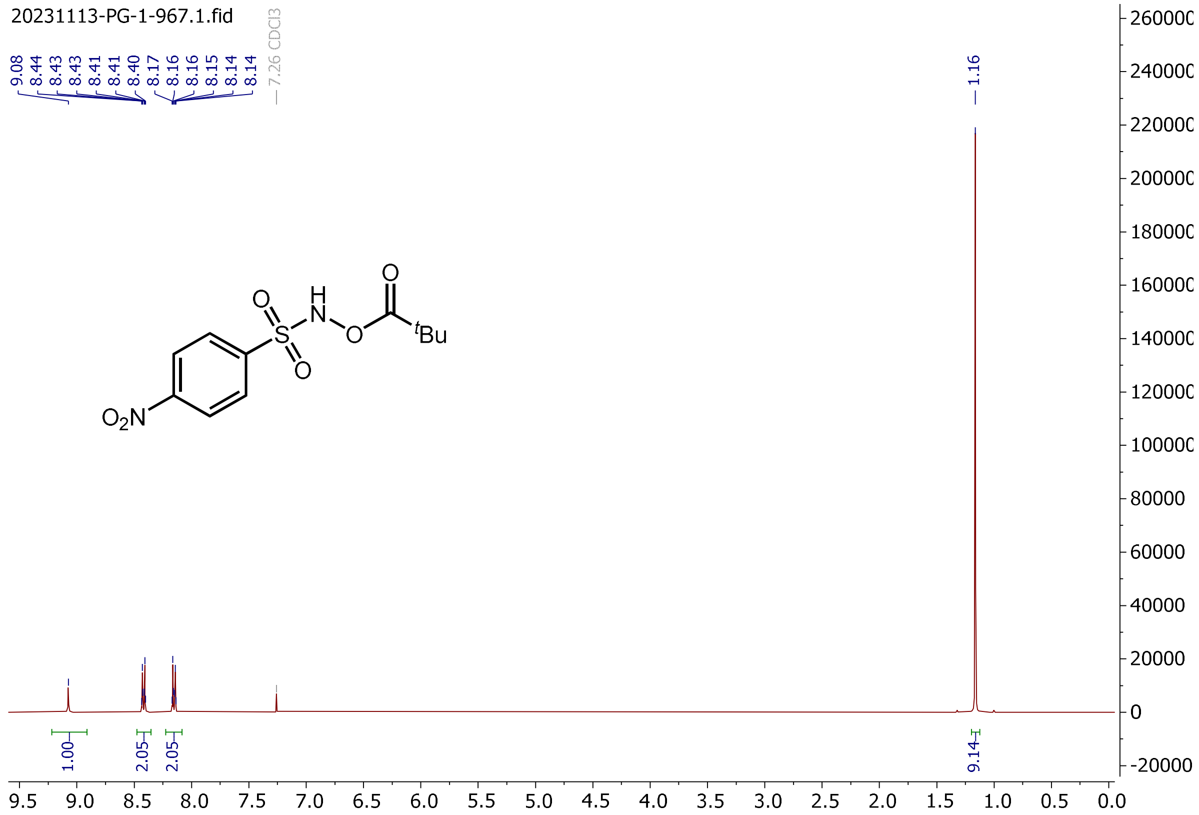

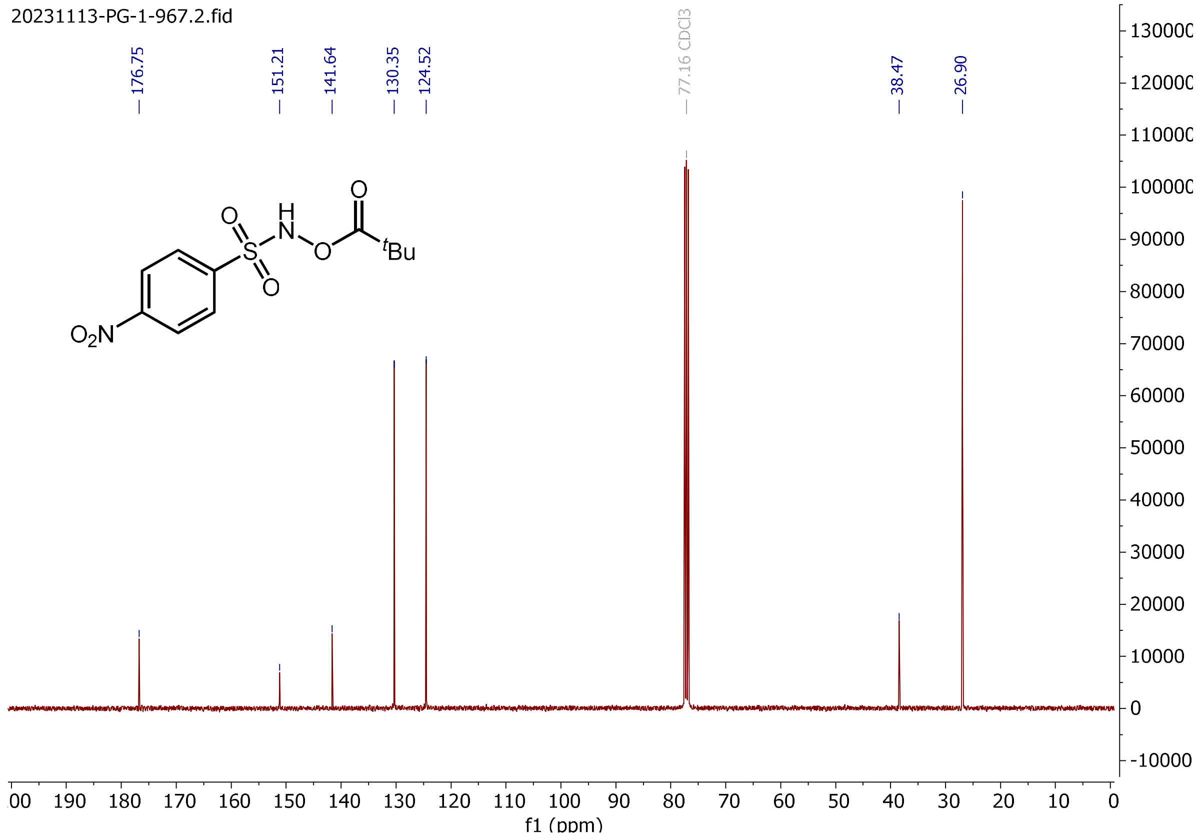

N-(pivaloyloxy)methanesulfonamide (**S35**):

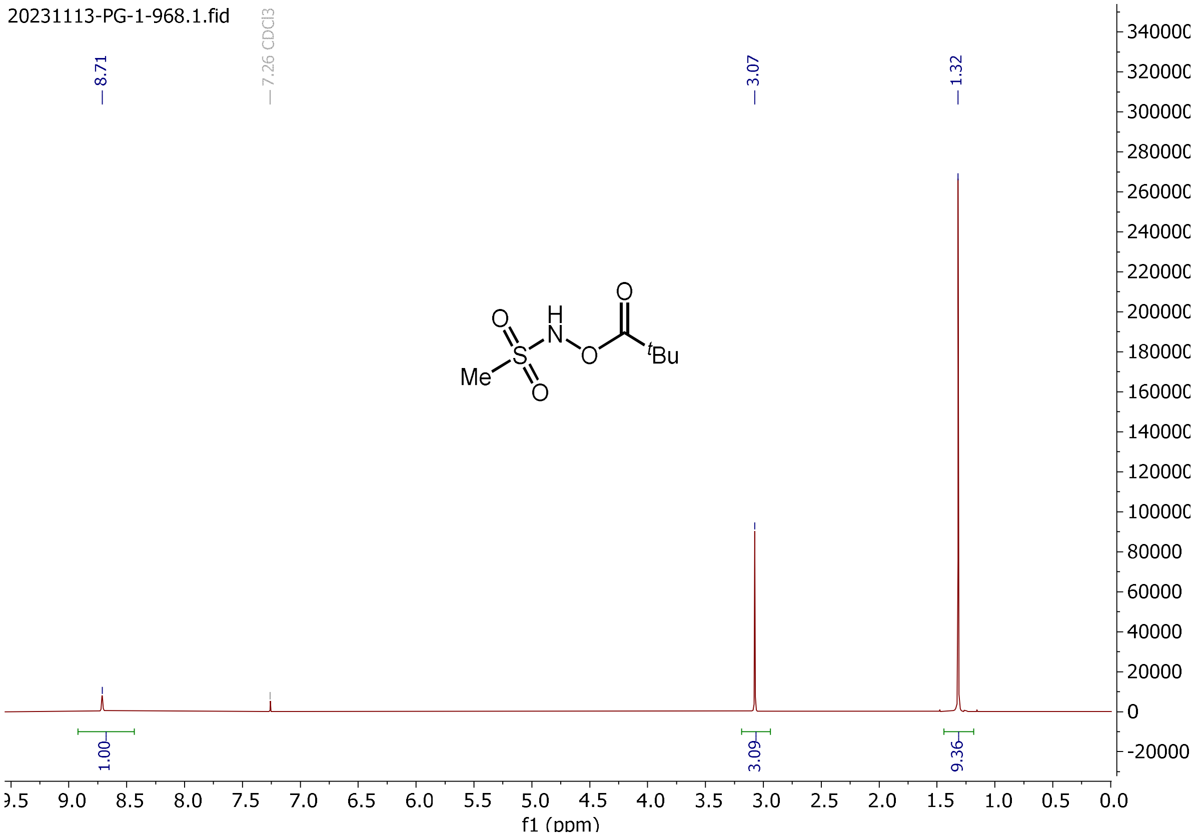

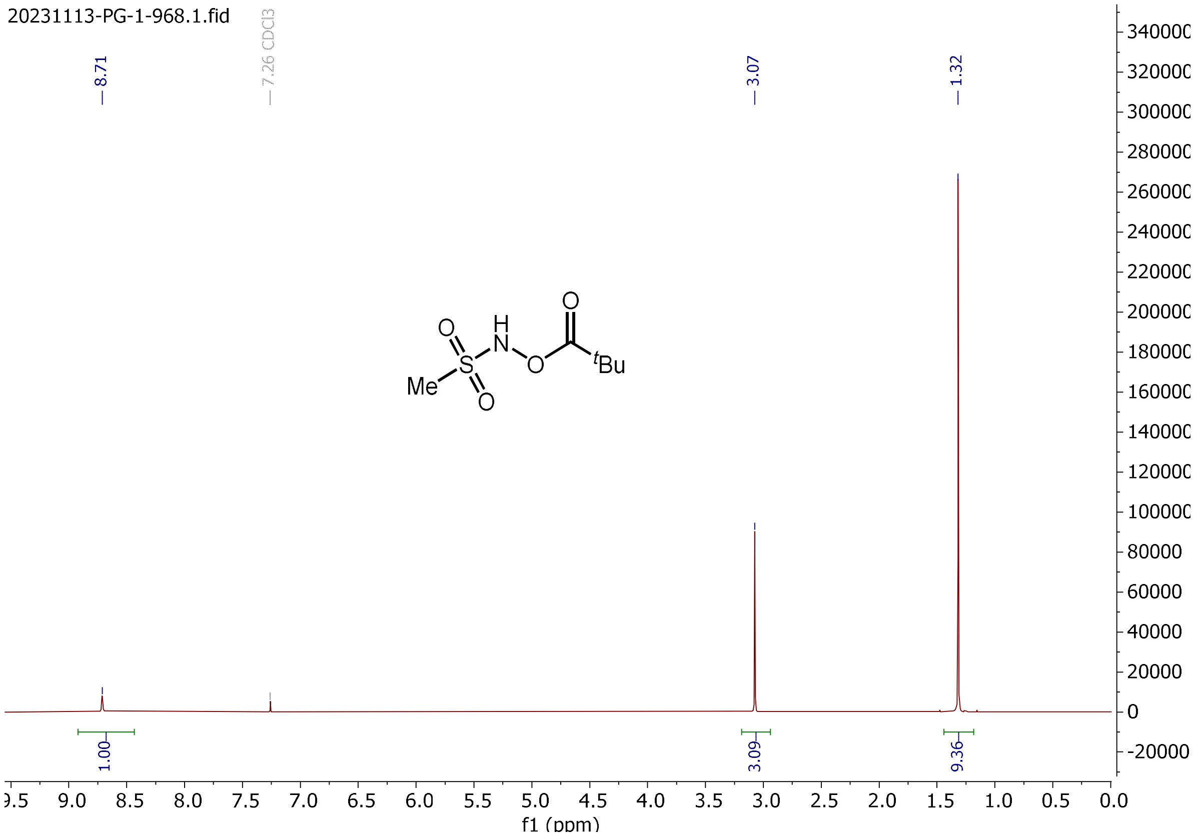

hex-5-en-1-yl morpholine-4-carboxylate (**S36**):

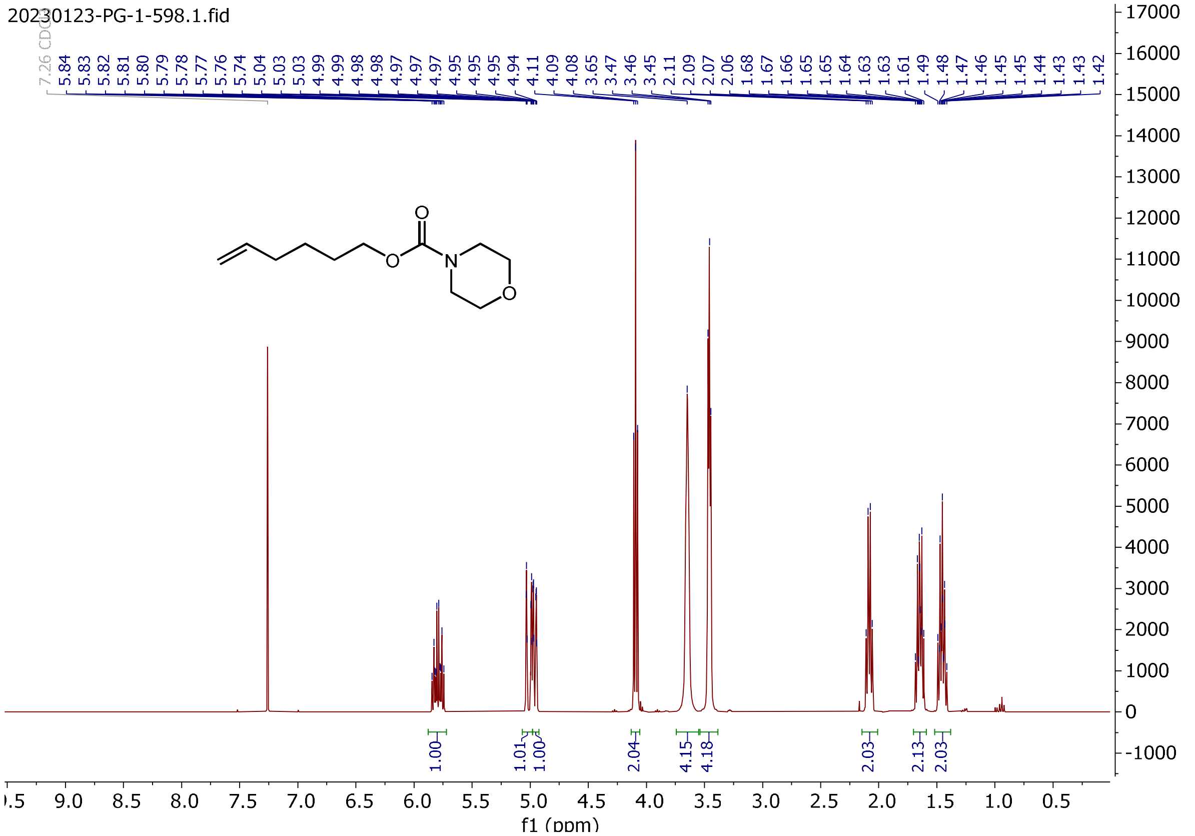

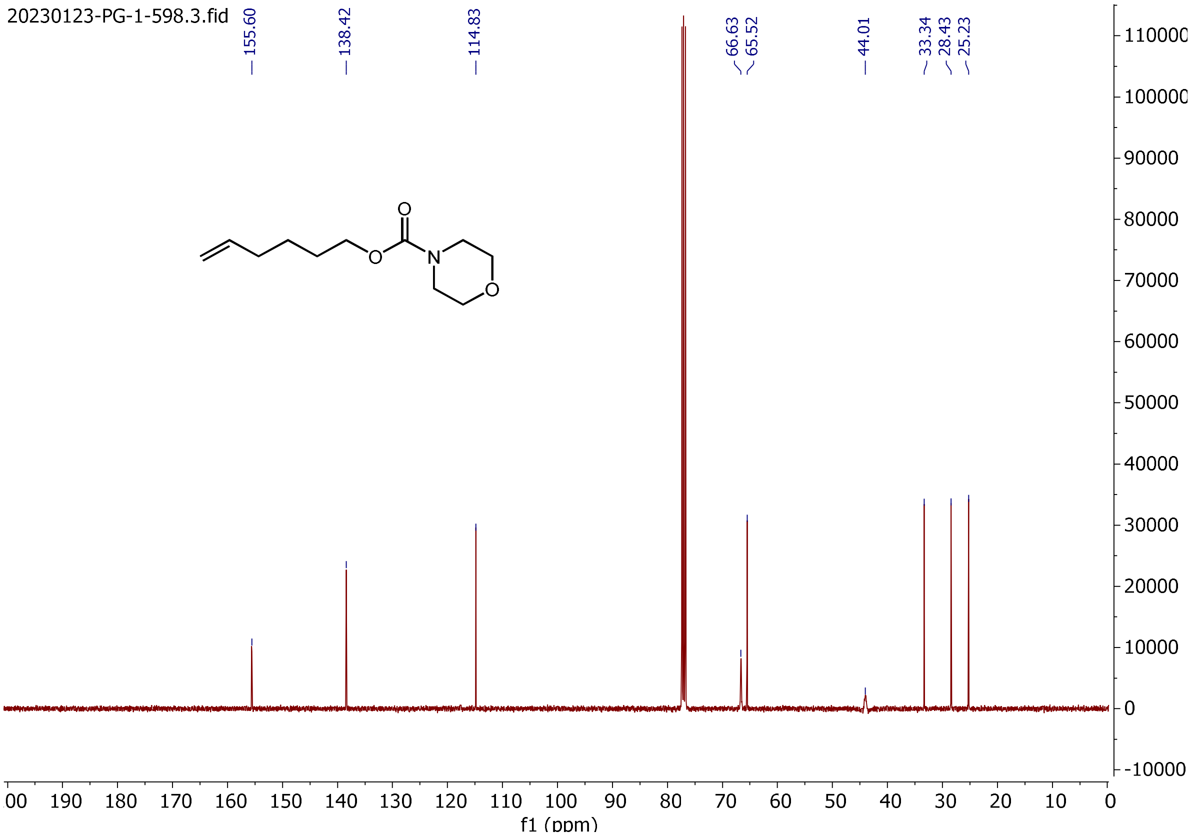

1-(hex-5-en-1-yl)indoline-2,3-dione (**S37**):

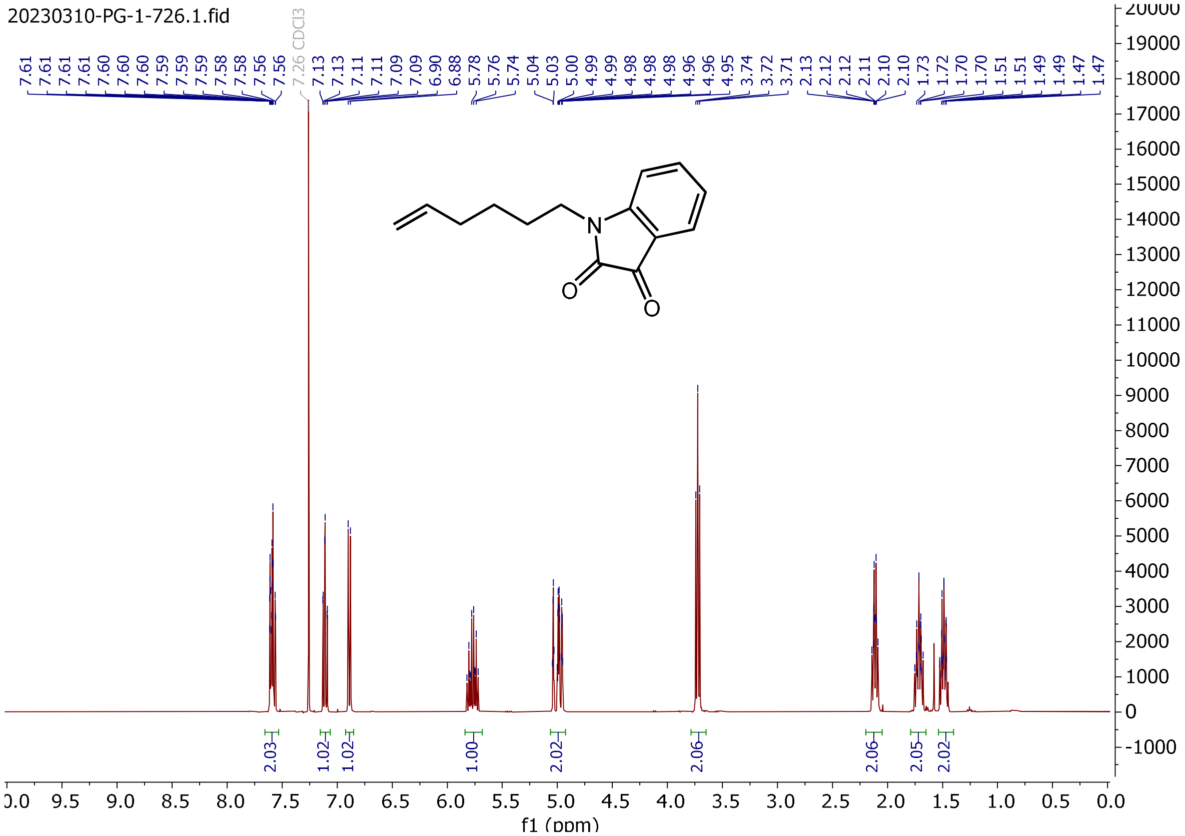

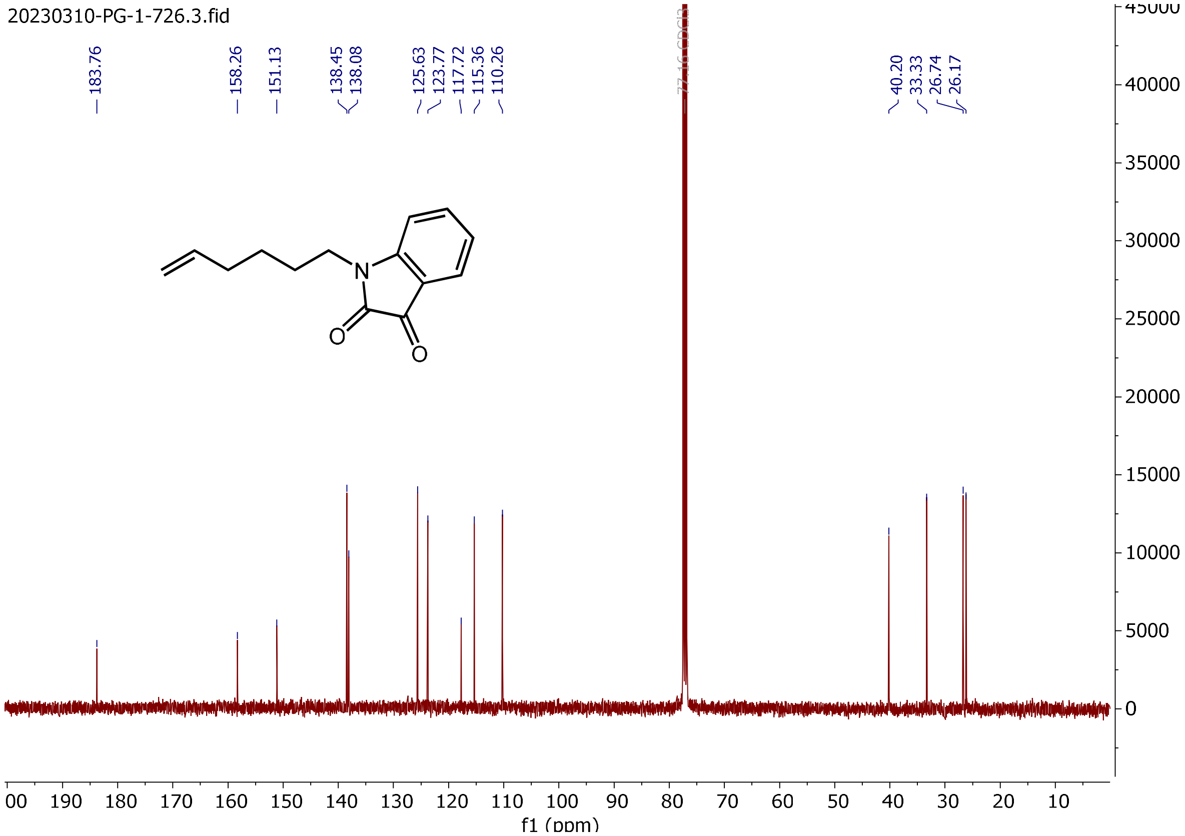

2-bromo-5-(hex-5-en-1-yloxy)pyrazine (**S38**):

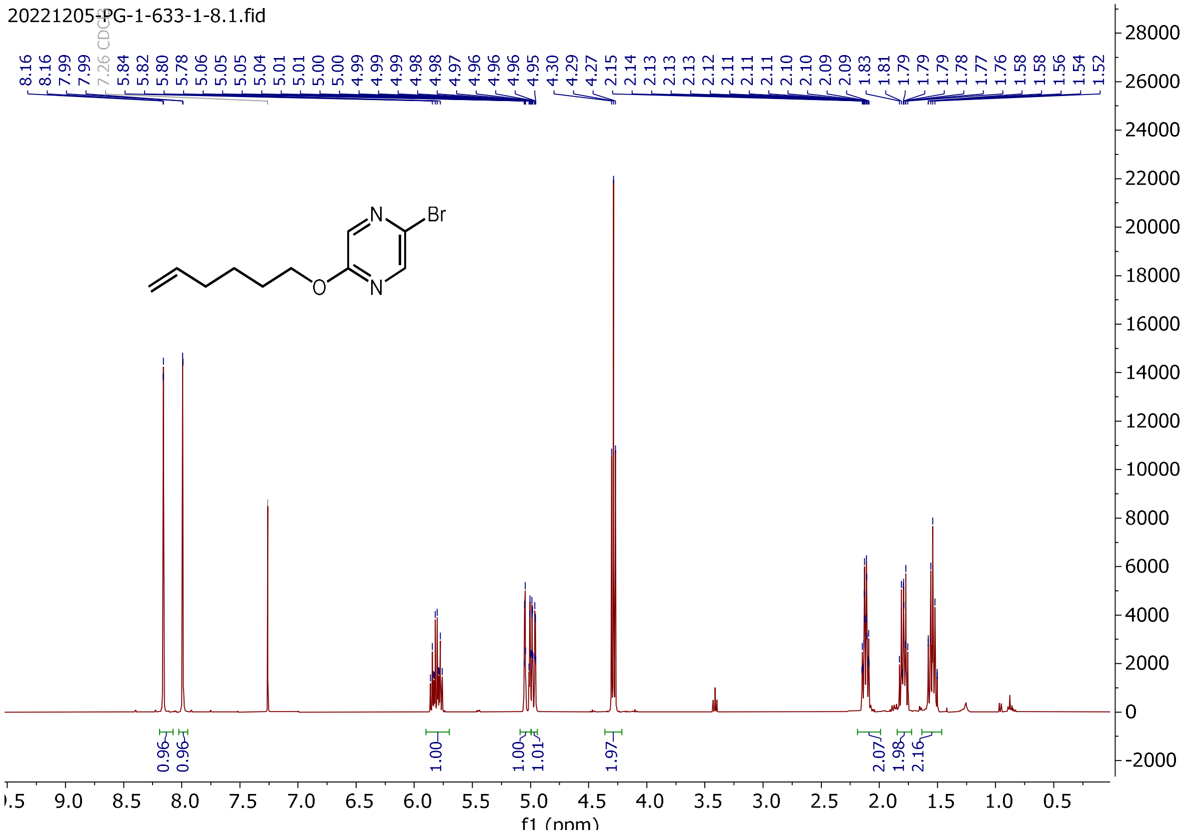

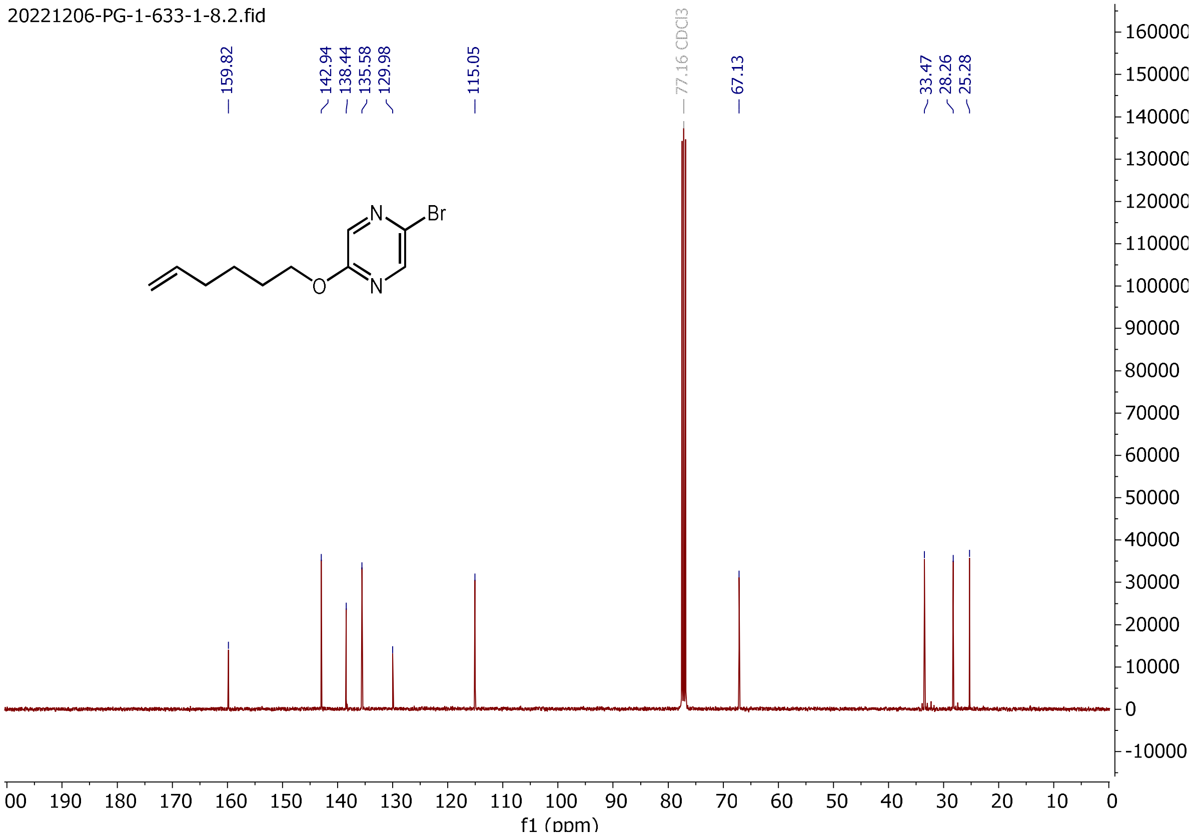

2-bromo-3-(hex-5-en-1-yloxy)pyridine (**S39**):

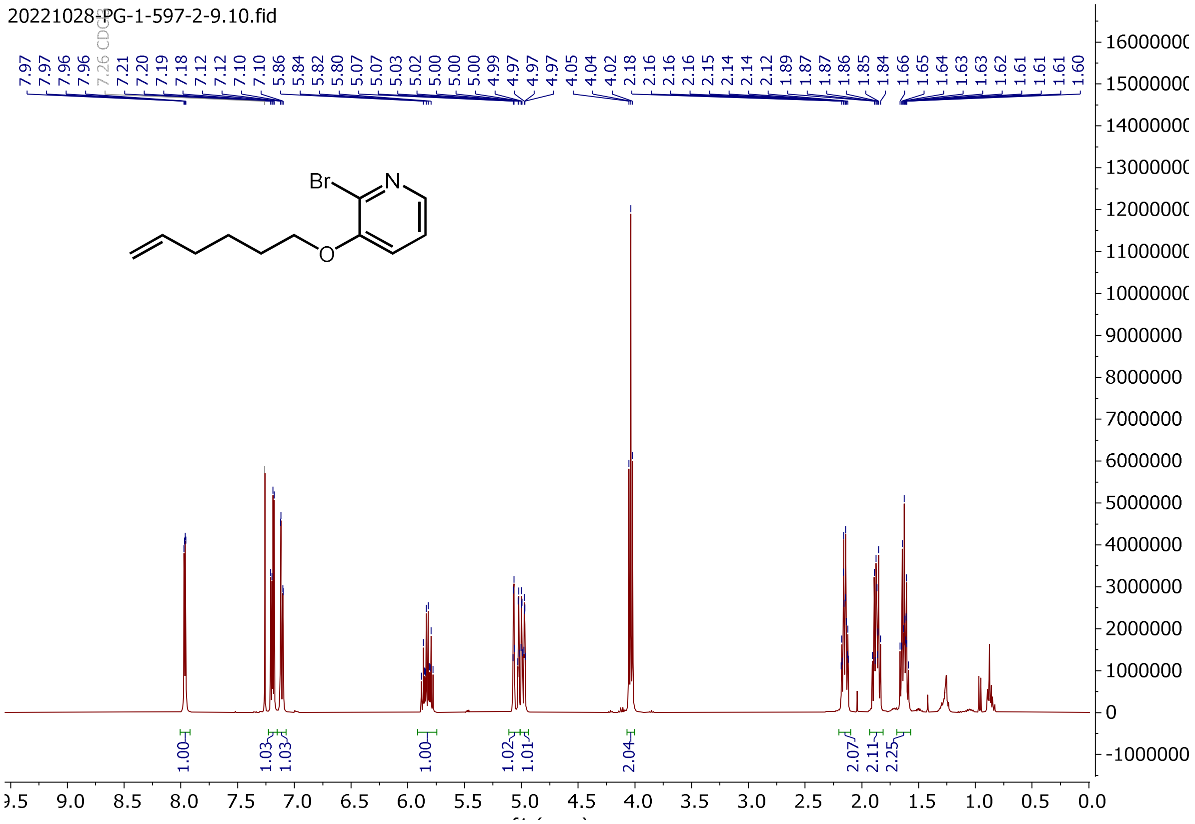

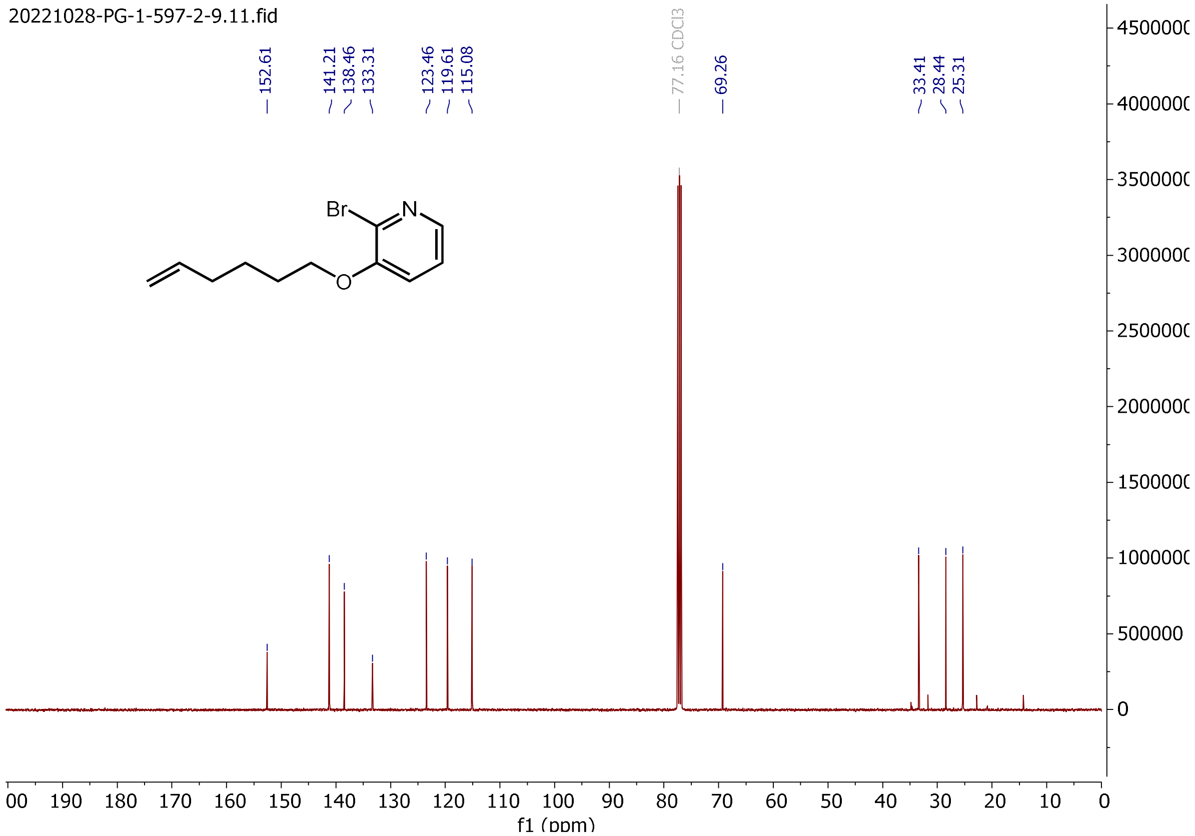

hex-5-en-1-yl (tert-butoxycarbonyl)-L-phenylalaninate (**S40**):

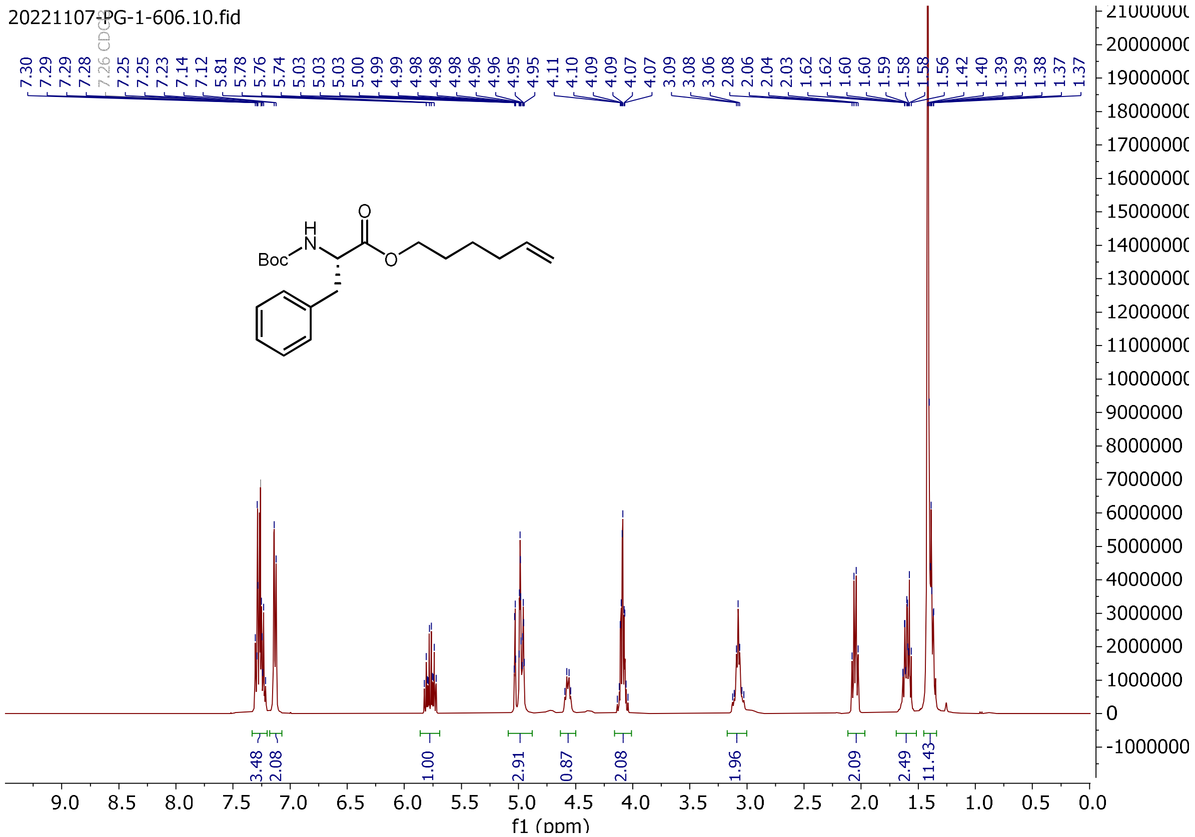

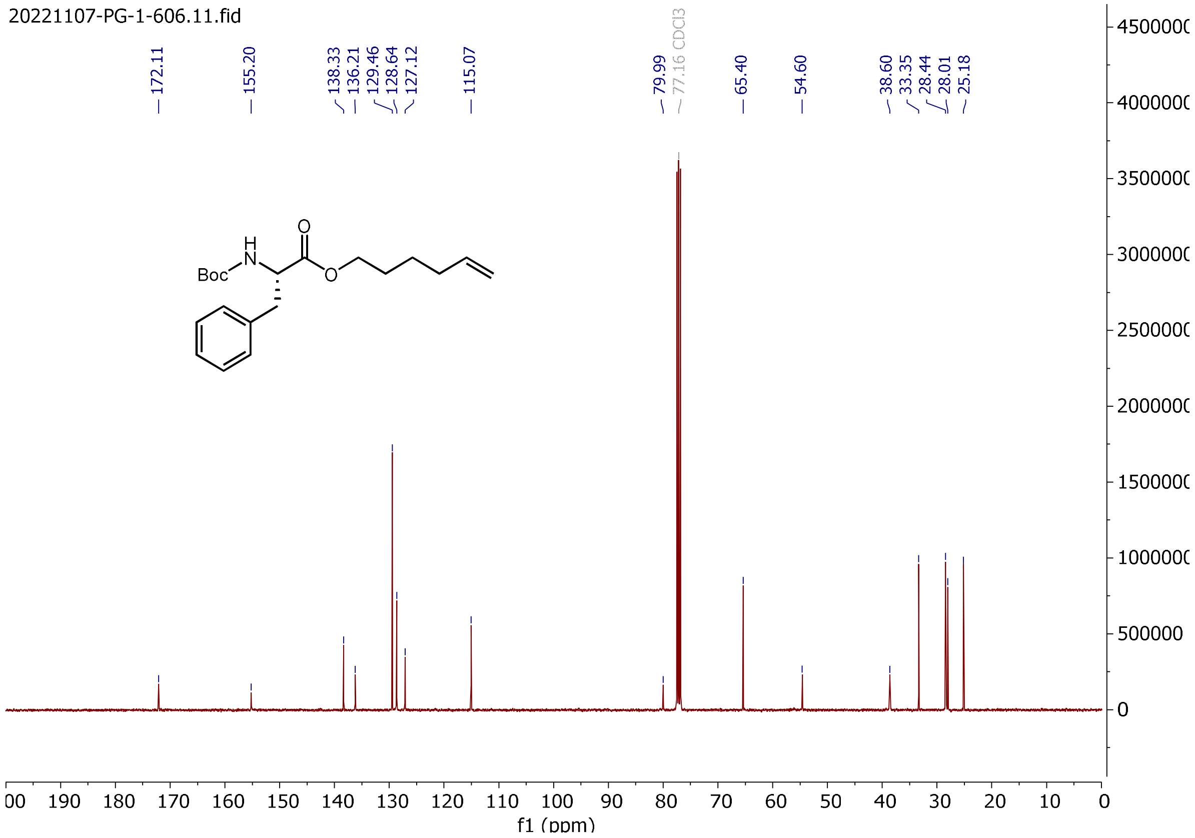

(S)-(((3-methylhept-6-en-1-yl)oxy)methyl)benzene (**S44**):

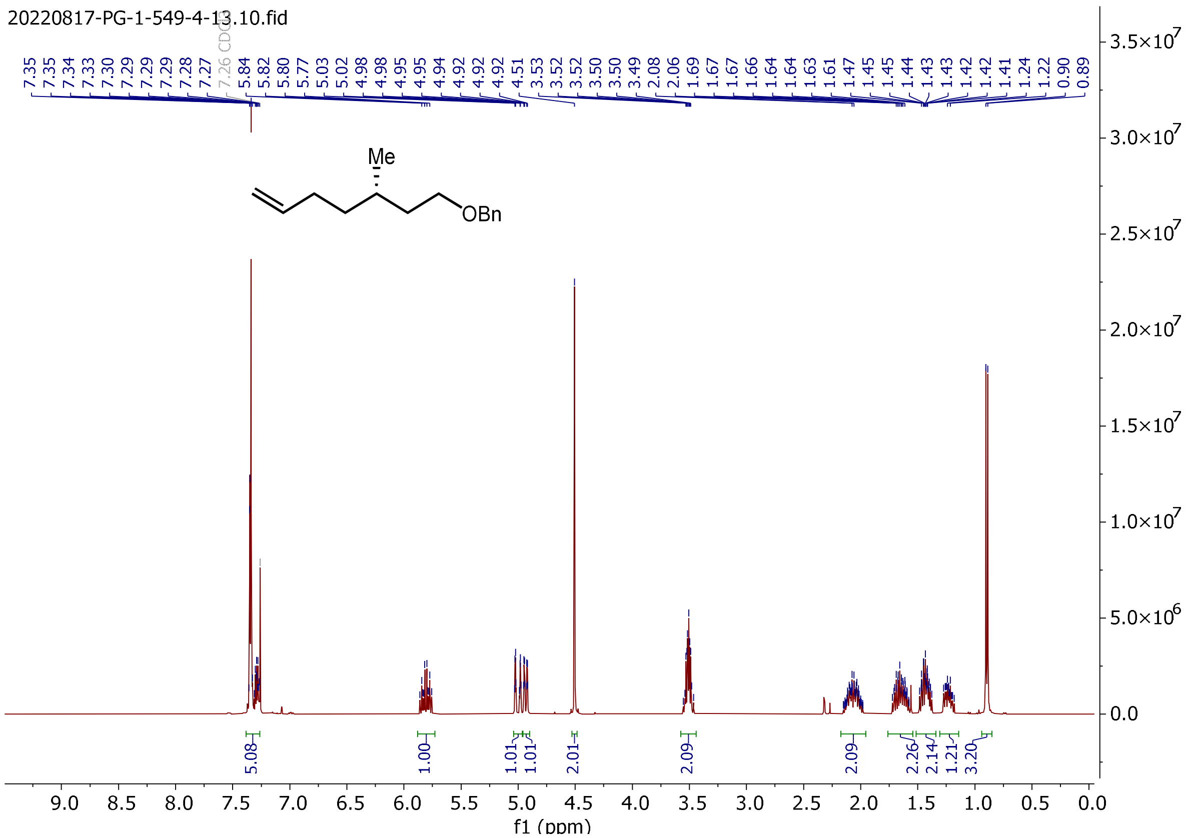

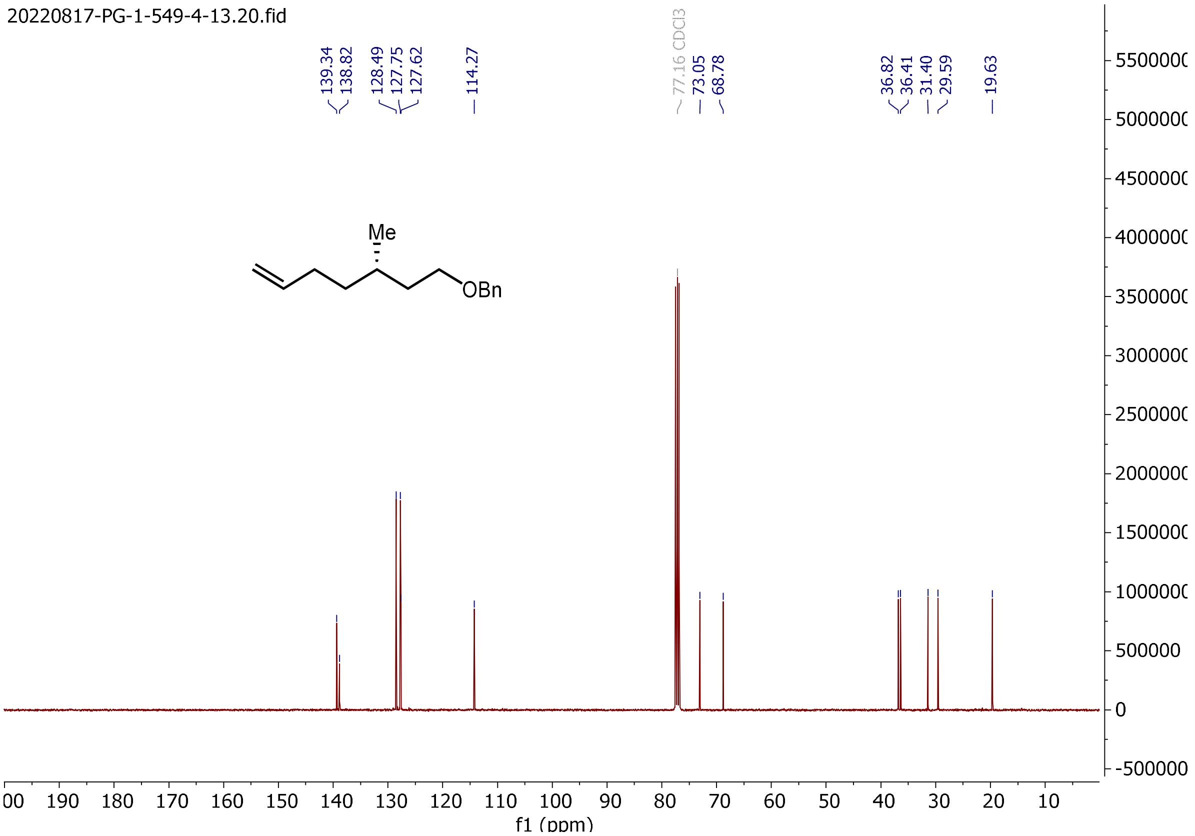

(S)-(((3-methylhex-5-en-1-yl)oxy)methyl)benzene (**S50**):

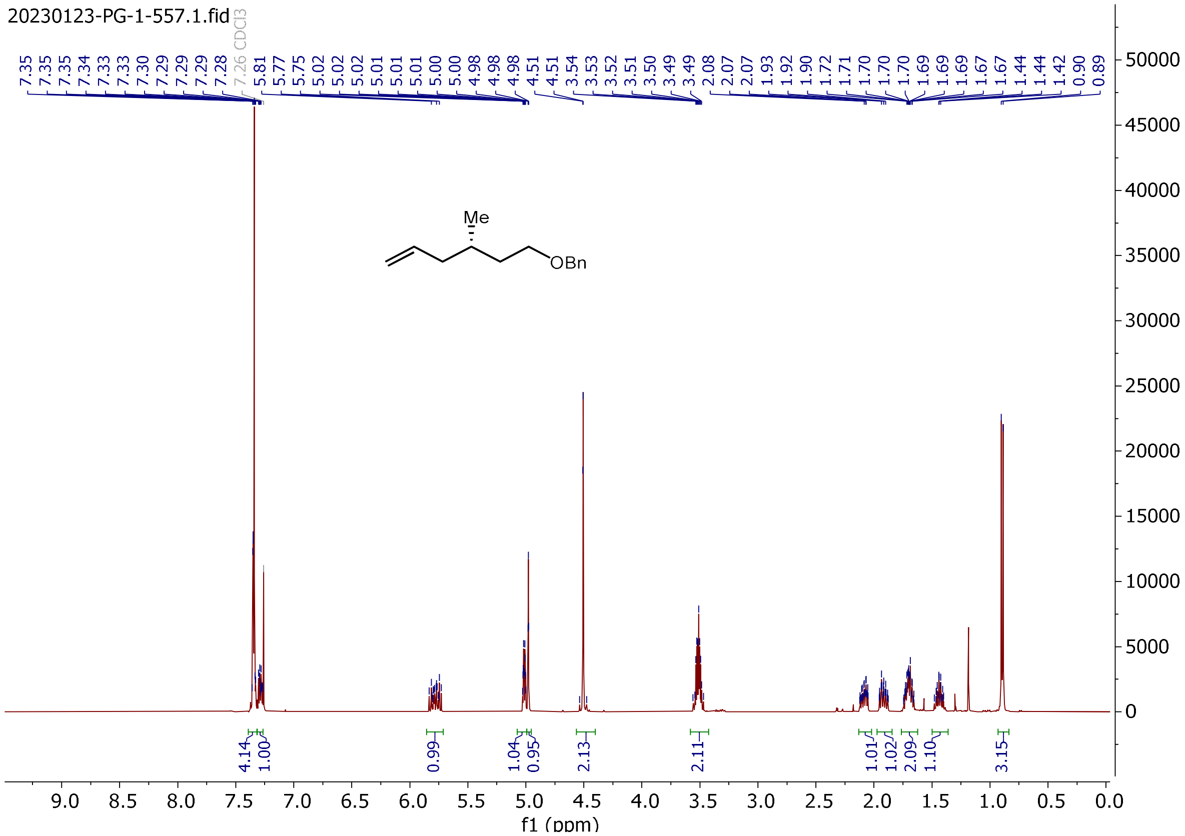

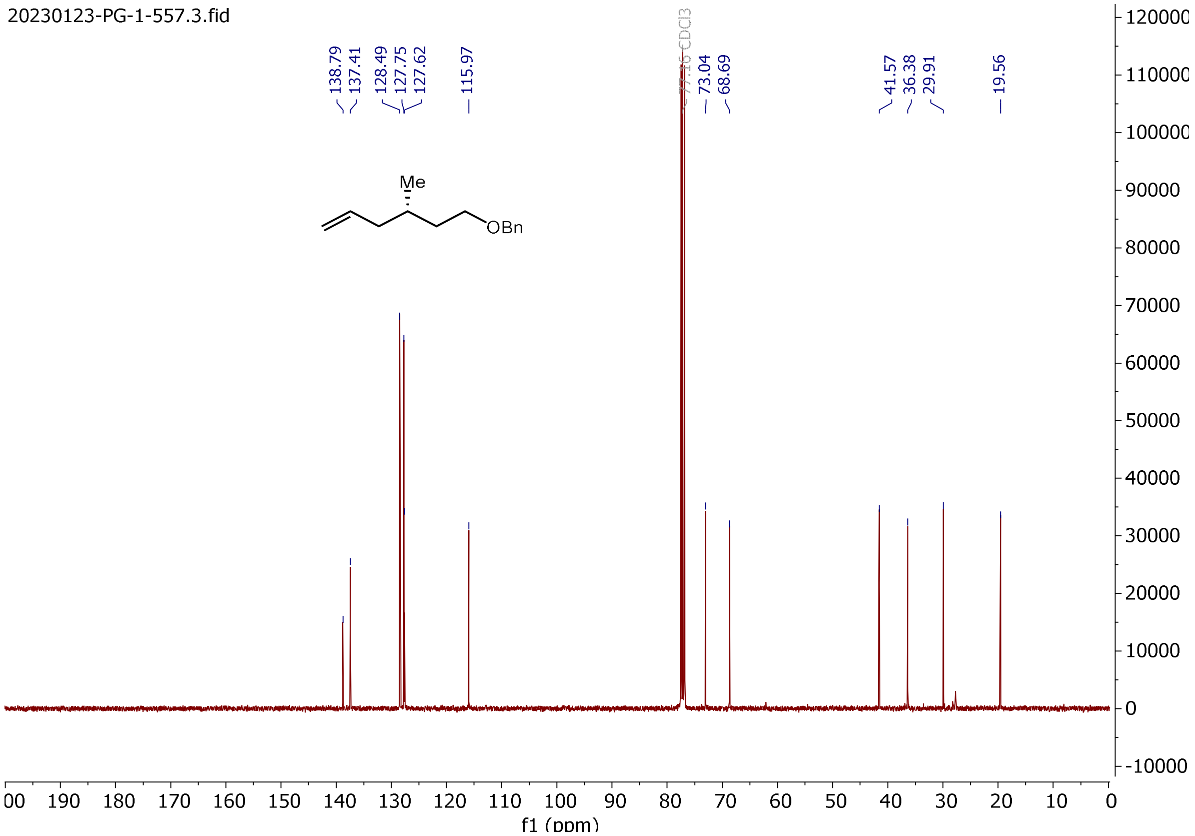

5-methylhex-5-en-1-yl benzoate (**S51**):

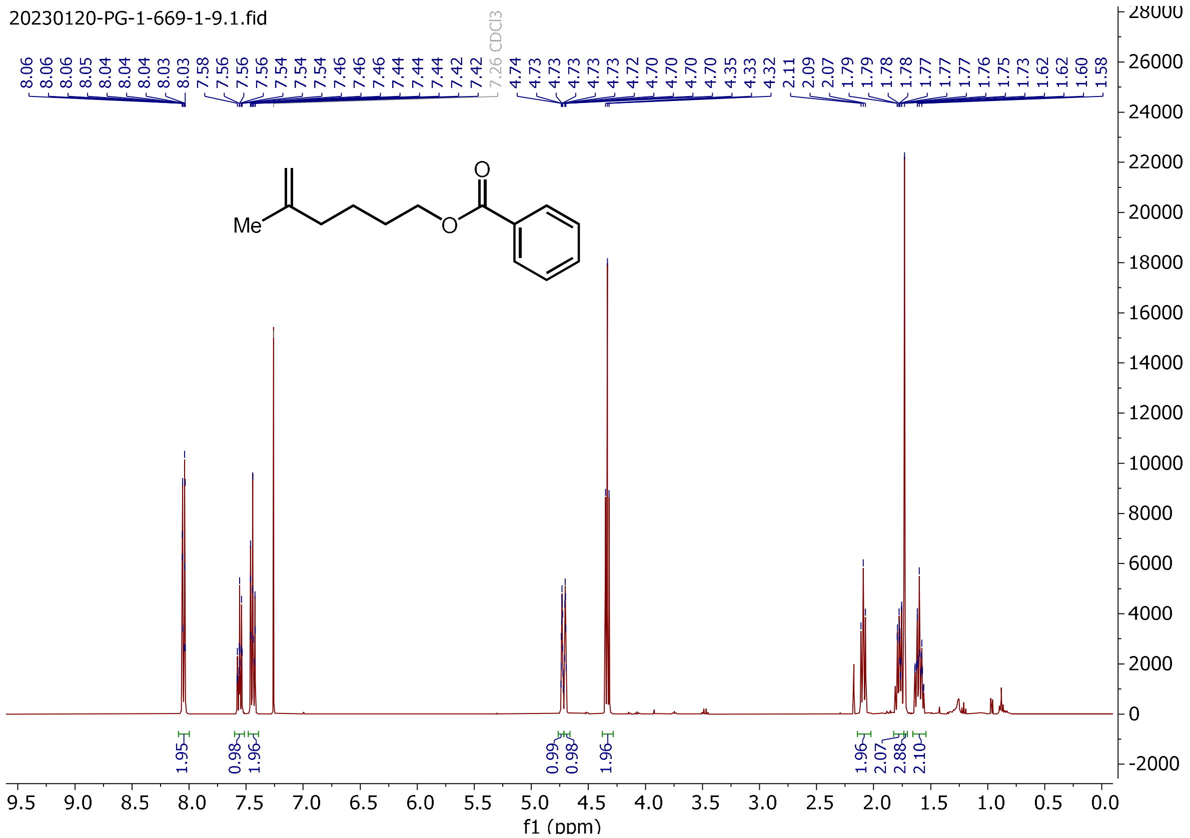

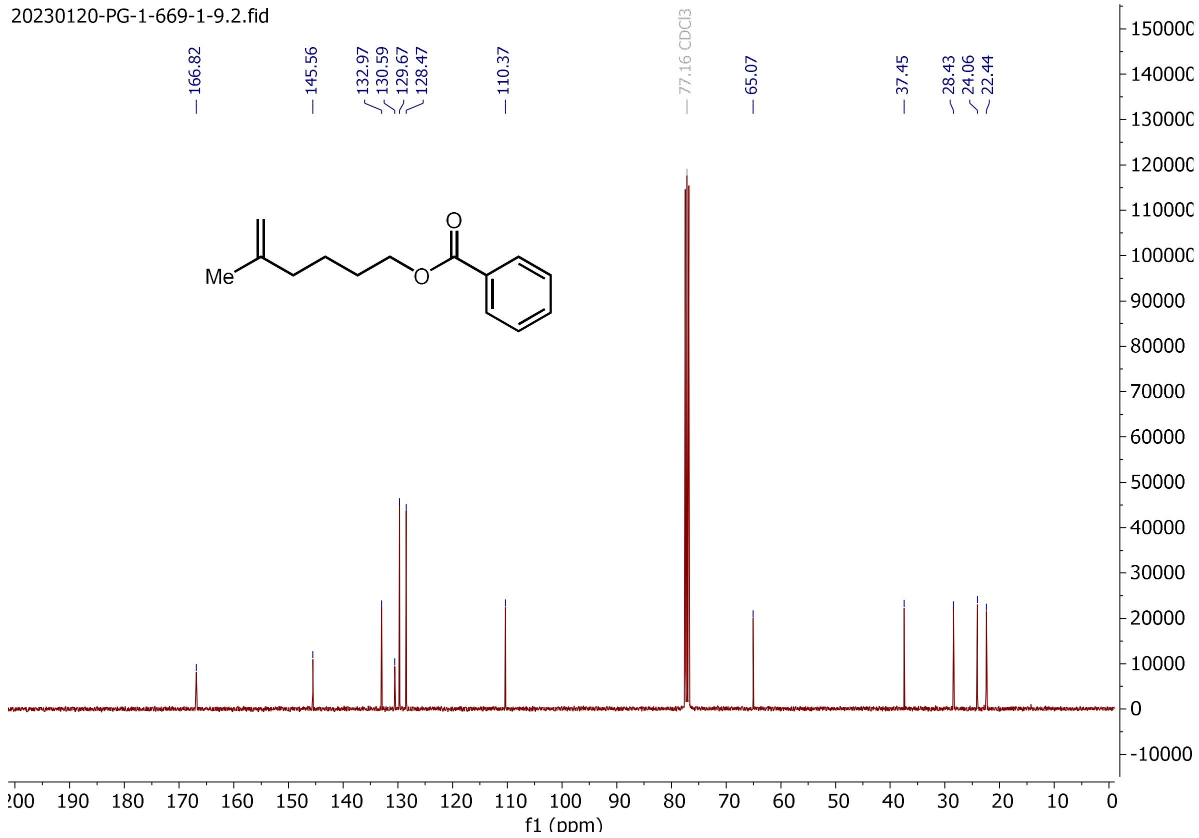

hex-5-en-1-yl acrylate (**S52**):

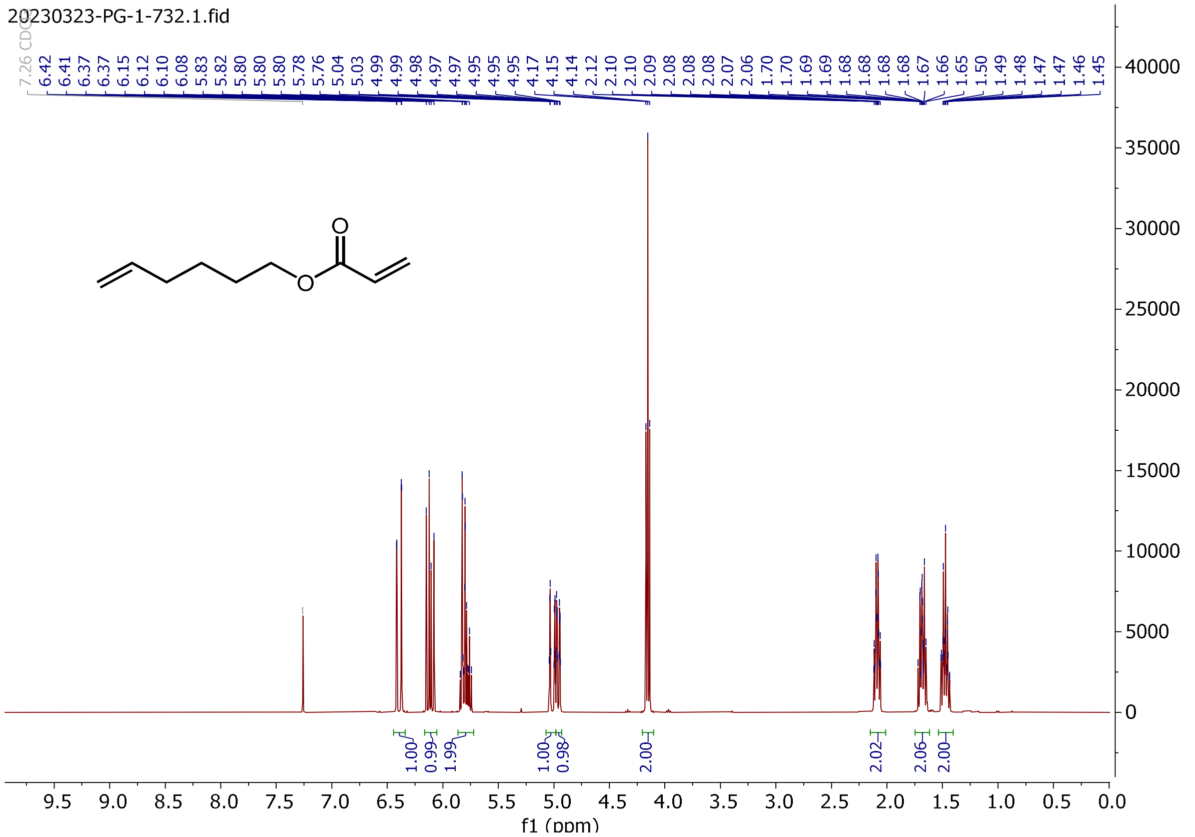

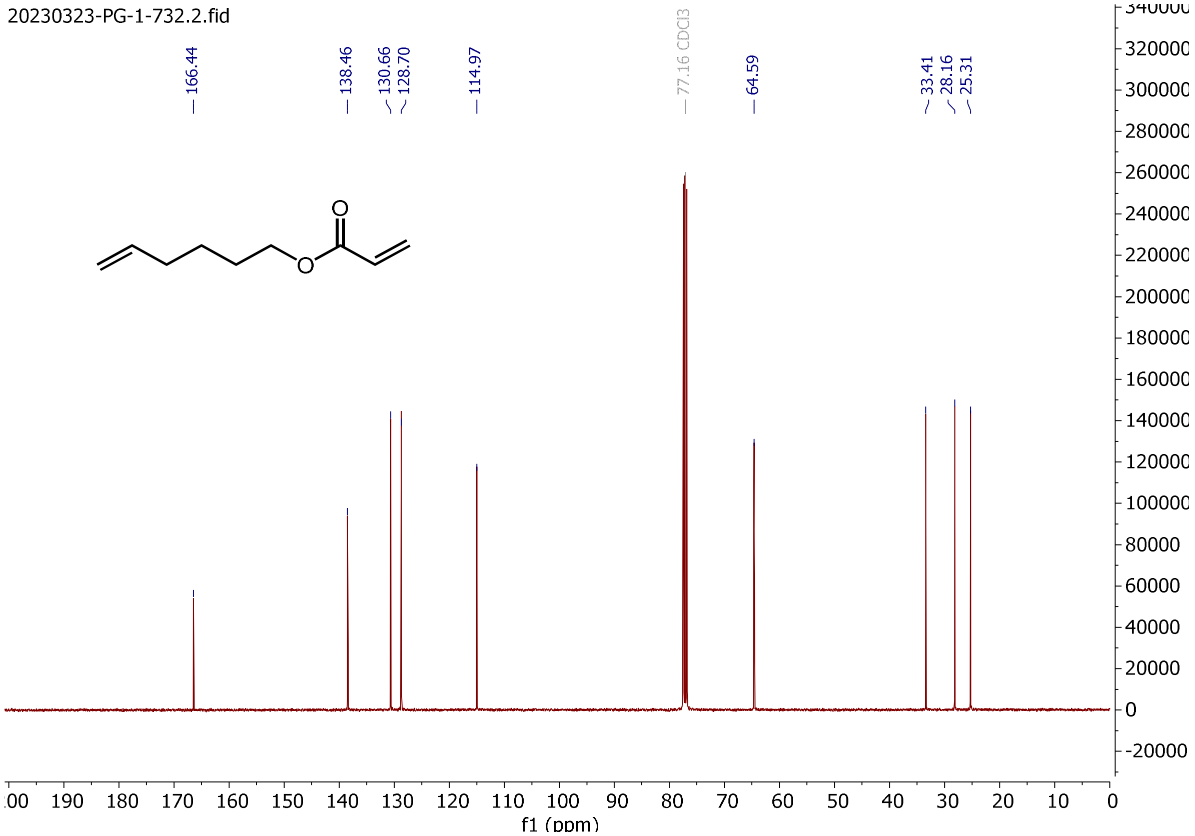

hex-5-en-1-yl 4-vinylbenzoate (**S53**):

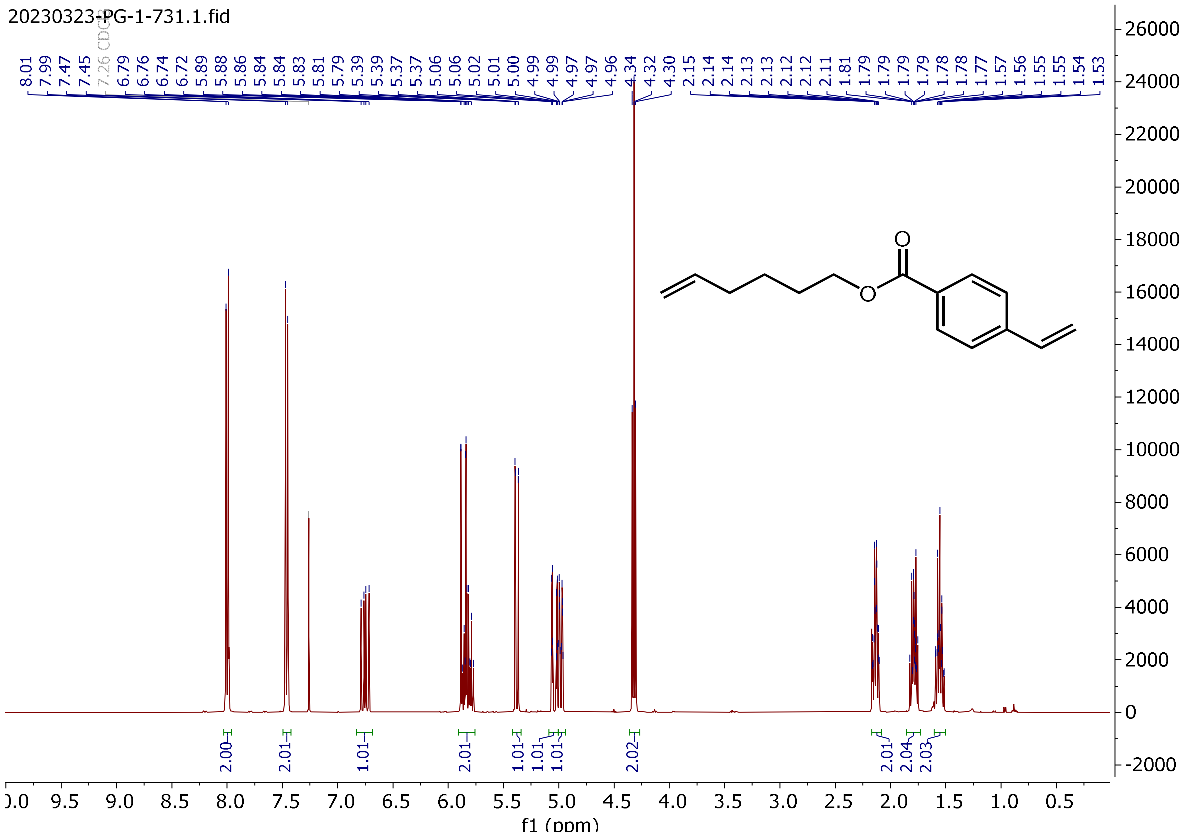

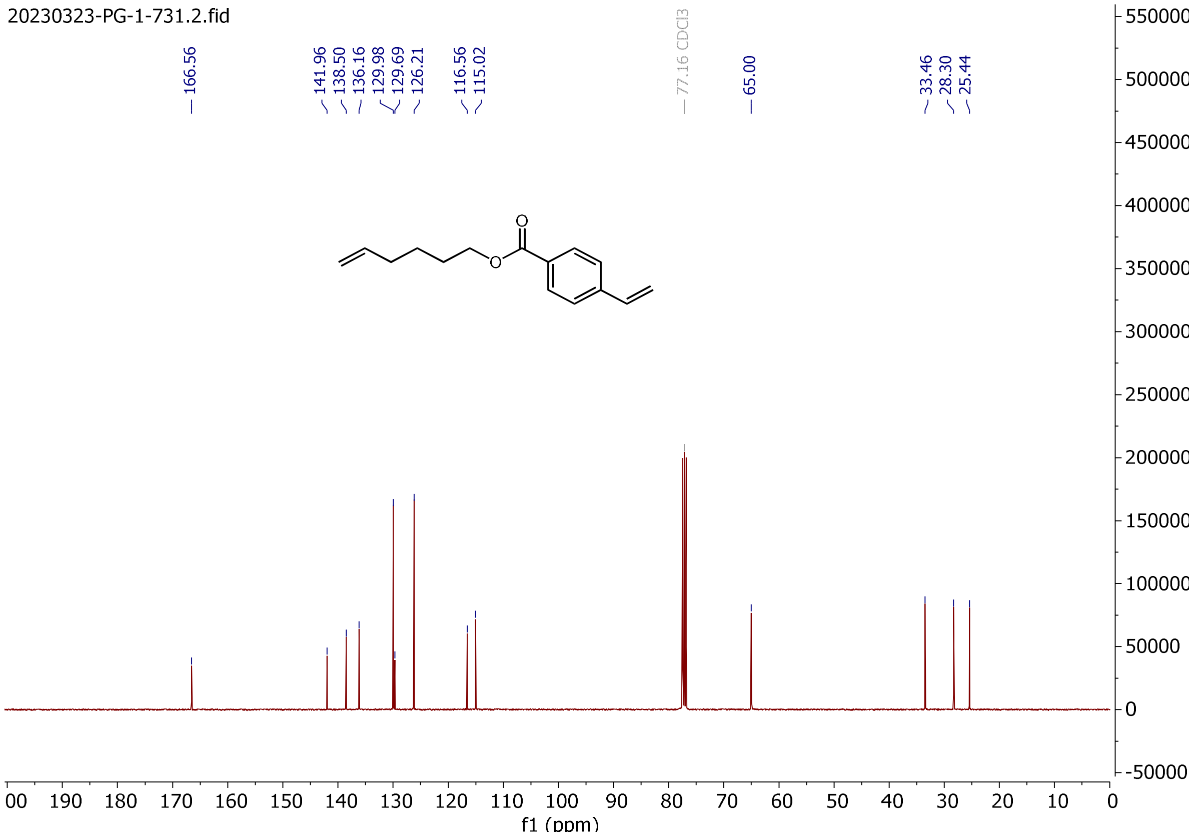

hex-5-en-1-yl cinnamate (**S54**):

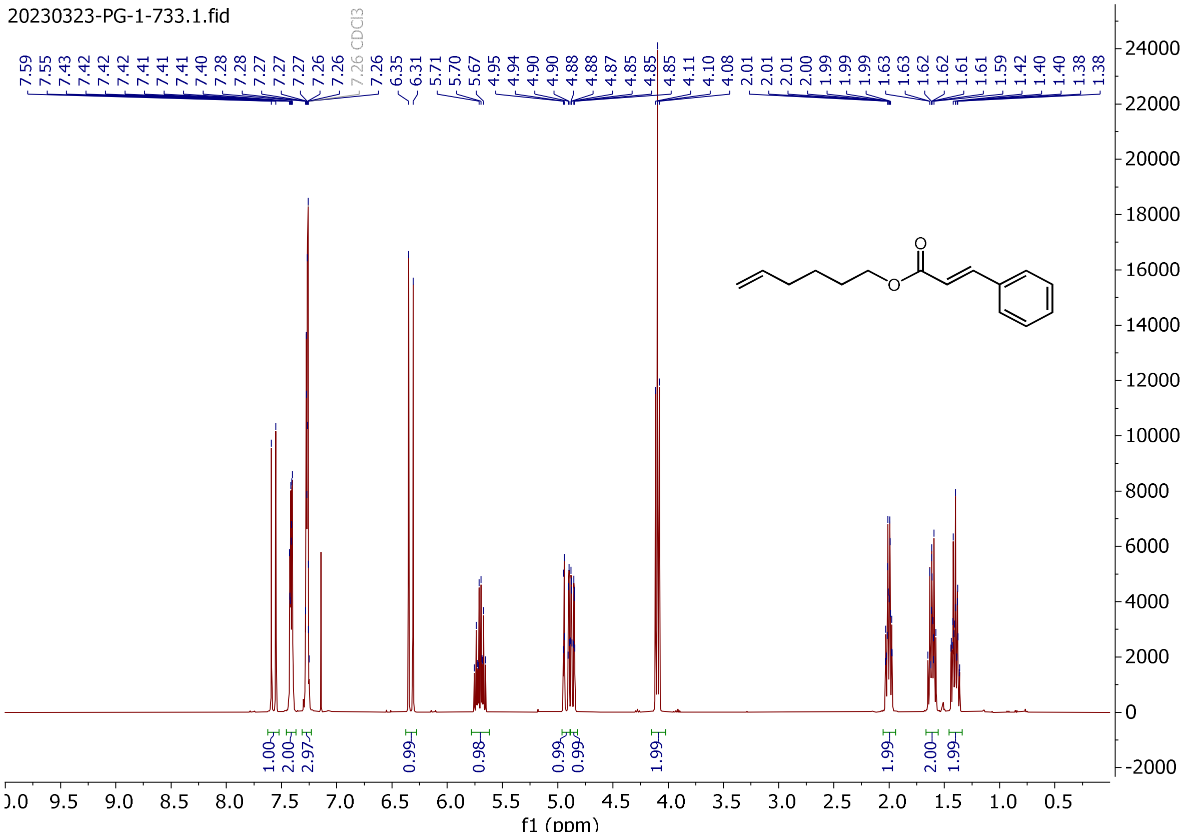

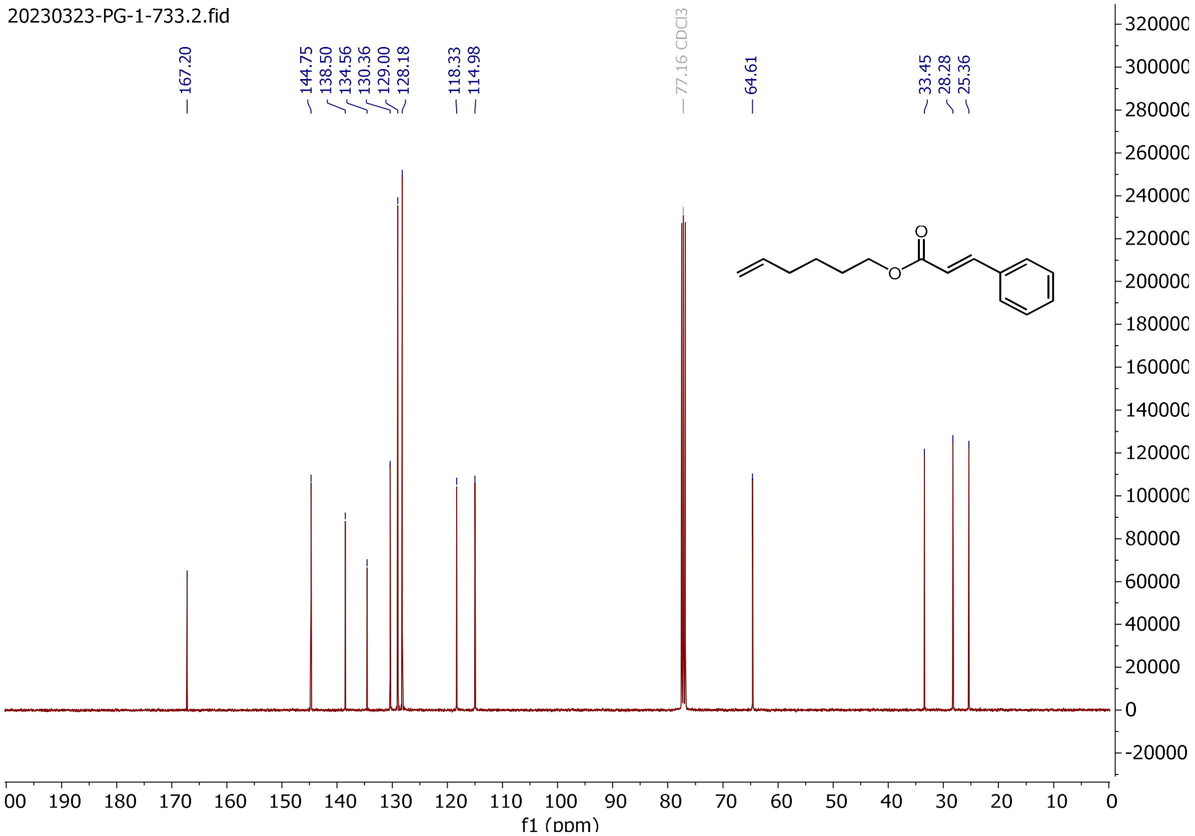

(*S*)-2-heptyl-1-tosylaziridine (**4**)

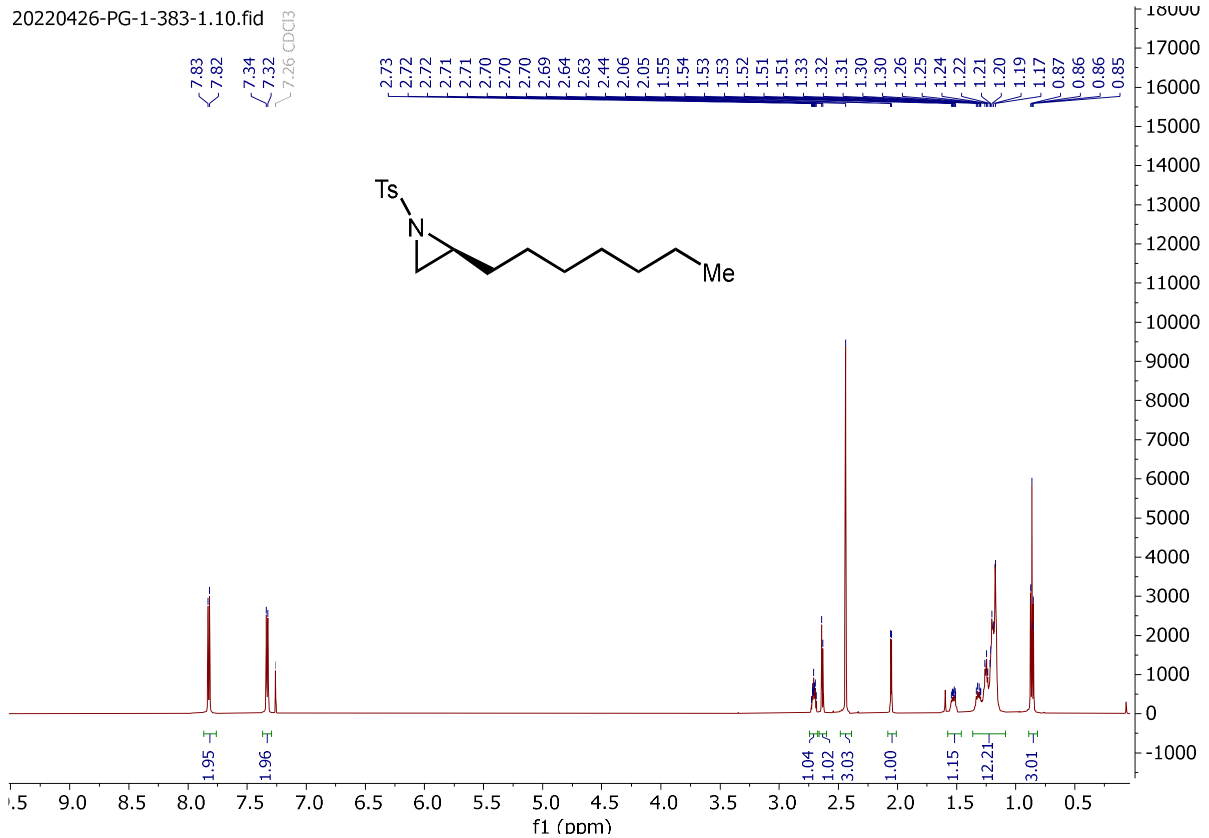

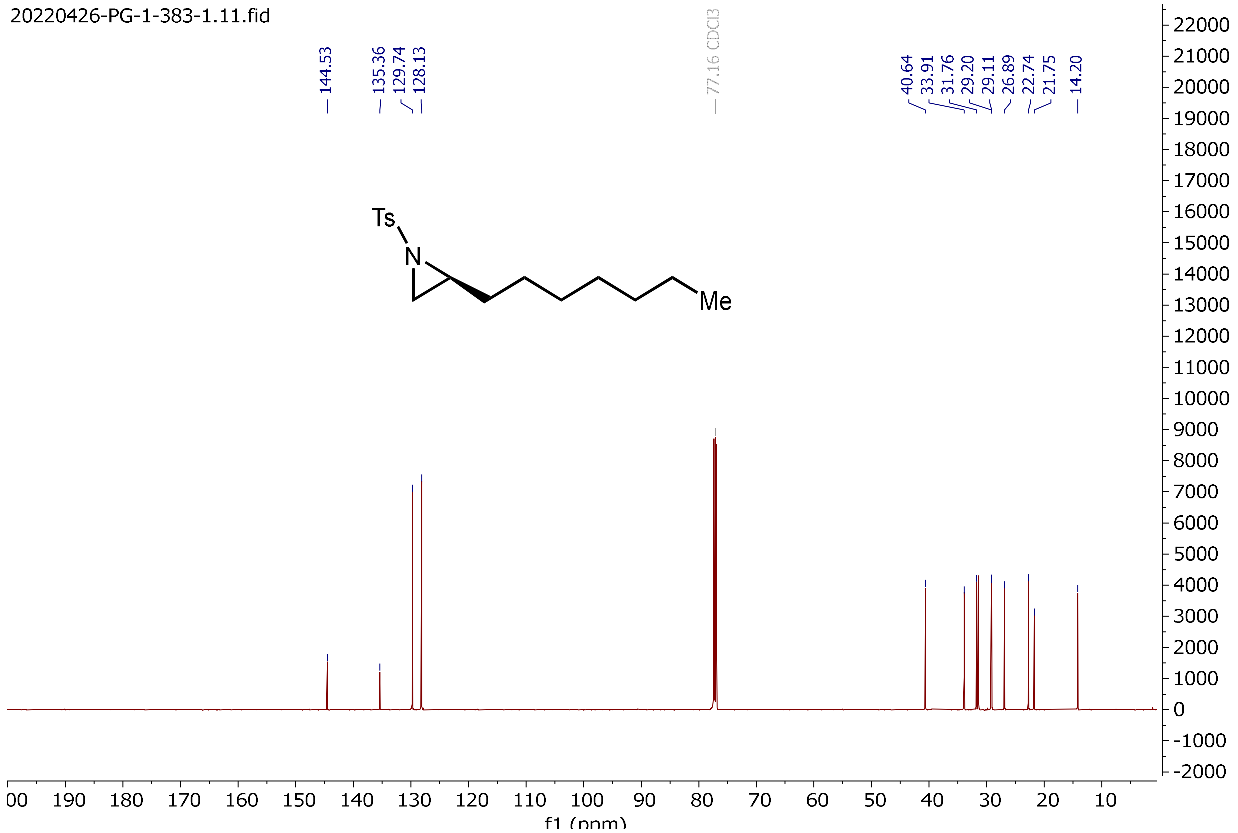

(±)-2-heptyl-1-tosylaziridine (±-**4**)

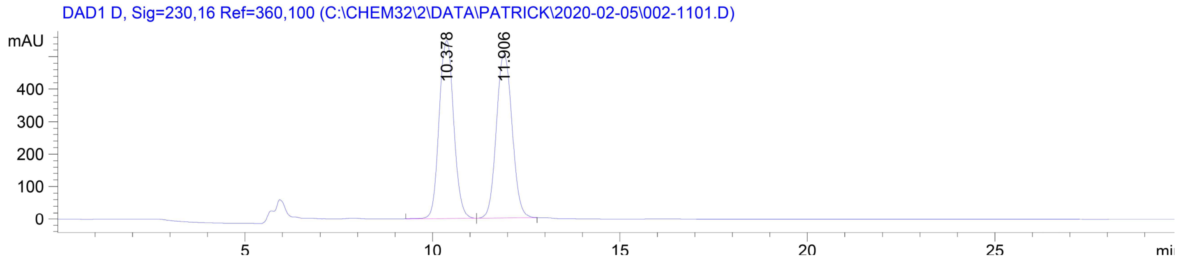

Signal 3: DAD1 D, Sig=230,16 Ref=360,100

Peak #	RetTime [min]	Type	Width [min]	Area [mAU*s]	Height [mAU]	Area %
1	10.378	BV	0.4221	1.47883e4	549.36700	49.9755
2	11.906	VB	0.4678	1.48028e4	502.37924	50.0245
Totals :				2.95912e4	1051.74625	

(*S*)-2-heptyl-1-tosylaziridine (**4**)

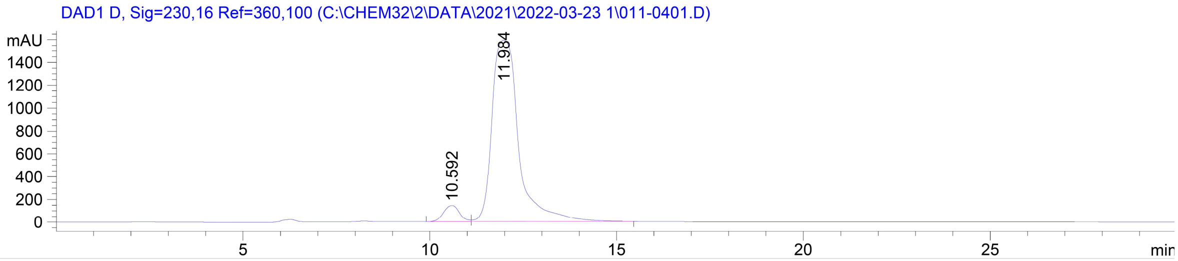

Signal 3: DAD1 D, Sig=230,16 Ref=360,100

Peak #	RetTime [min]	Type	Width [min]	Area [mAU*s]	Height [mAU]	Area %
1	10.592	BV	0.4747	4295.61133	142.89545	5.1673
2	11.984	VB	0.7675	7.88350e4	1592.51965	94.8327
Totals :				8.31306e4	1735.41510	

(*S*)-2-(4-(benzyloxy)butyl)-1-tosylaziridine (**10**)

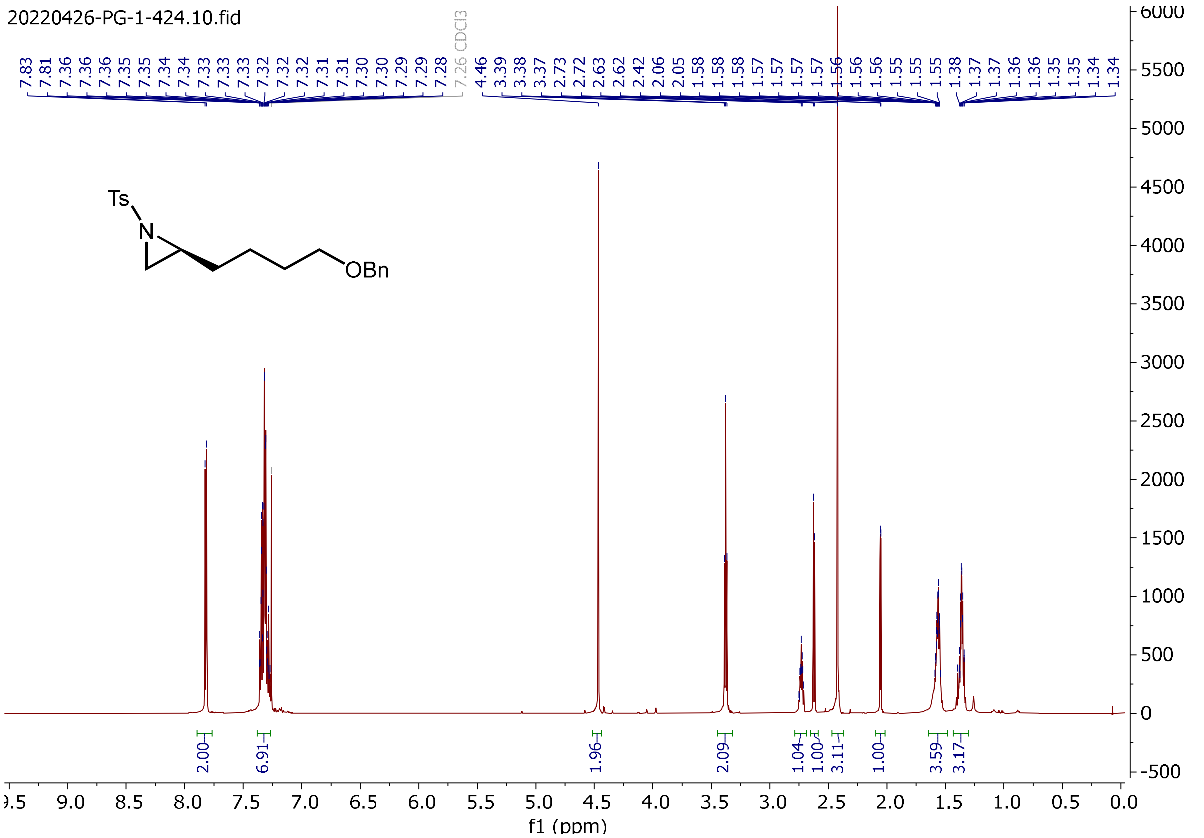

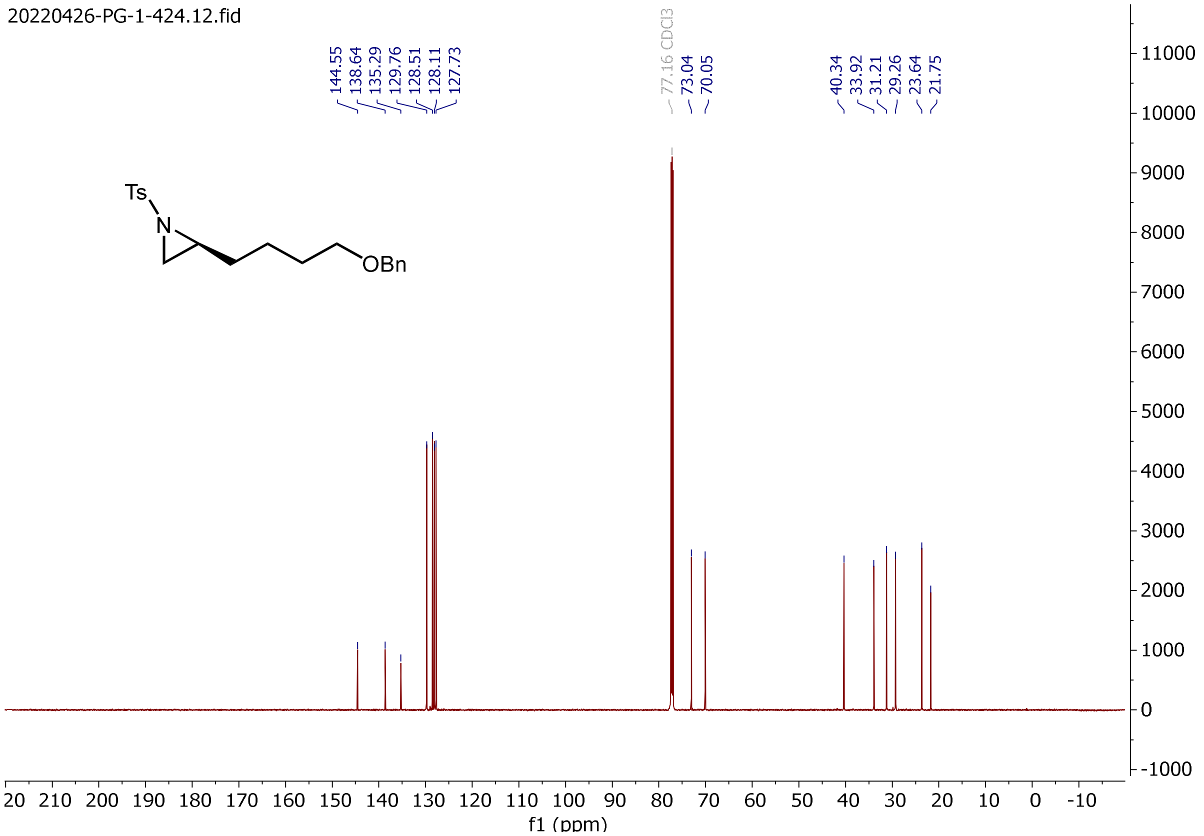

(±)-2-(4-(benzyloxy)butyl)-1-tosylaziridine (±-**10**)

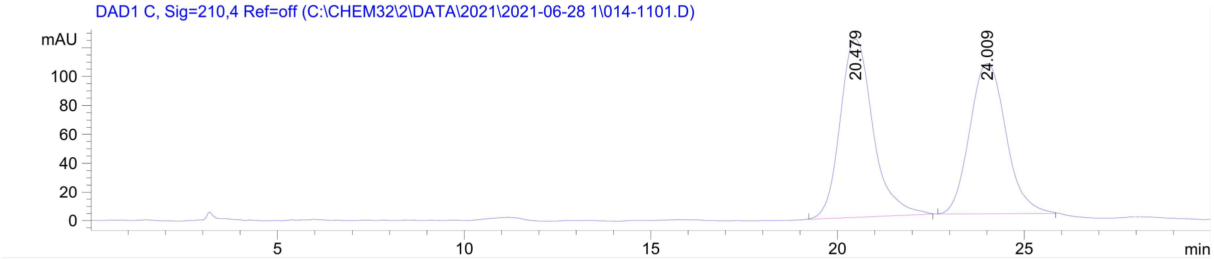

Signal 2: DAD1 C, Sig=210,4 Ref=off

Peak #	RetTime [min]	Type	Width [min]	Area [mAU*s]	Height [mAU]	Area %
1	20.479	BB	0.9443	7594.81641	123.70526	51.8482
2	24.009	BB	1.0605	7053.35010	104.35783	48.1518

Totals : 1.46482e4 228.06309

(*S*)-2-(4-(benzyloxy)butyl)-1-tosylaziridine (**10**)

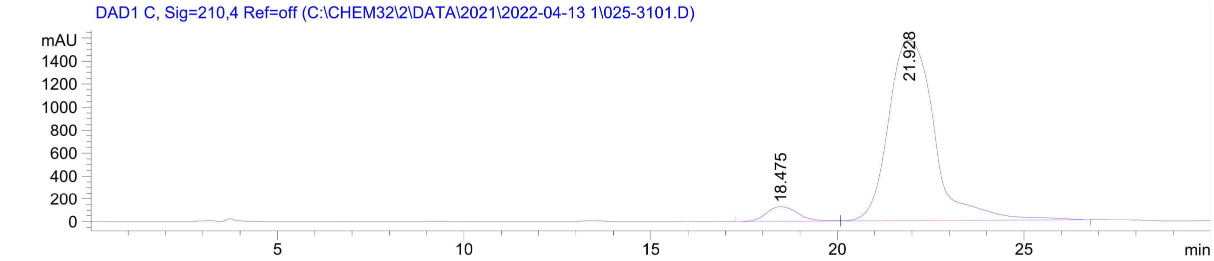

Signal 2: DAD1 C, Sig=210,4 Ref=off

Peak #	RetTime [min]	Type	Width [min]	Area [mAU*s]	Height [mAU]	Area %
1	18.475	BV	0.9394	7518.84668	127.61226	5.2736
2	21.928	VB	1.0292	1.35056e5	1573.80371	94.7264

Totals : 1.42575e5 1701.41597

(*S*)-4-(1-tosylaziridin-2-yl)butyl acetate (**11**):

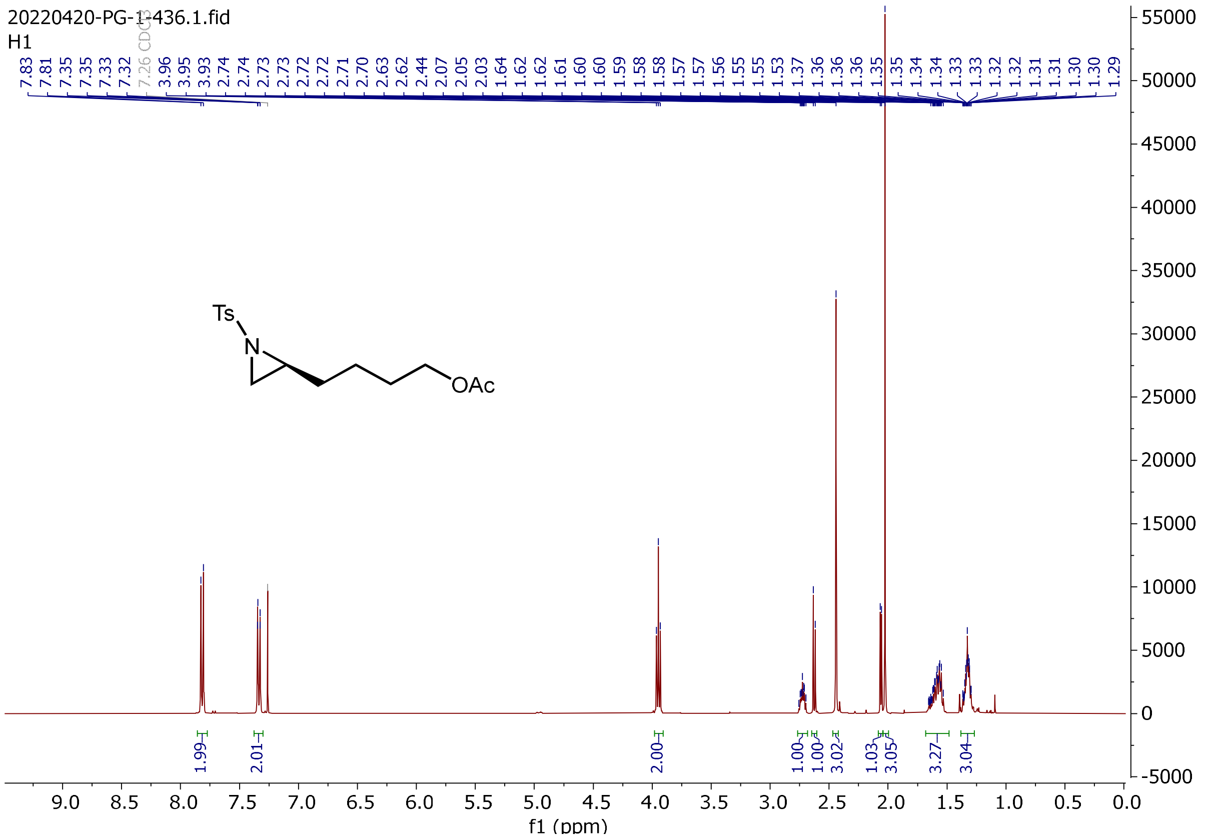

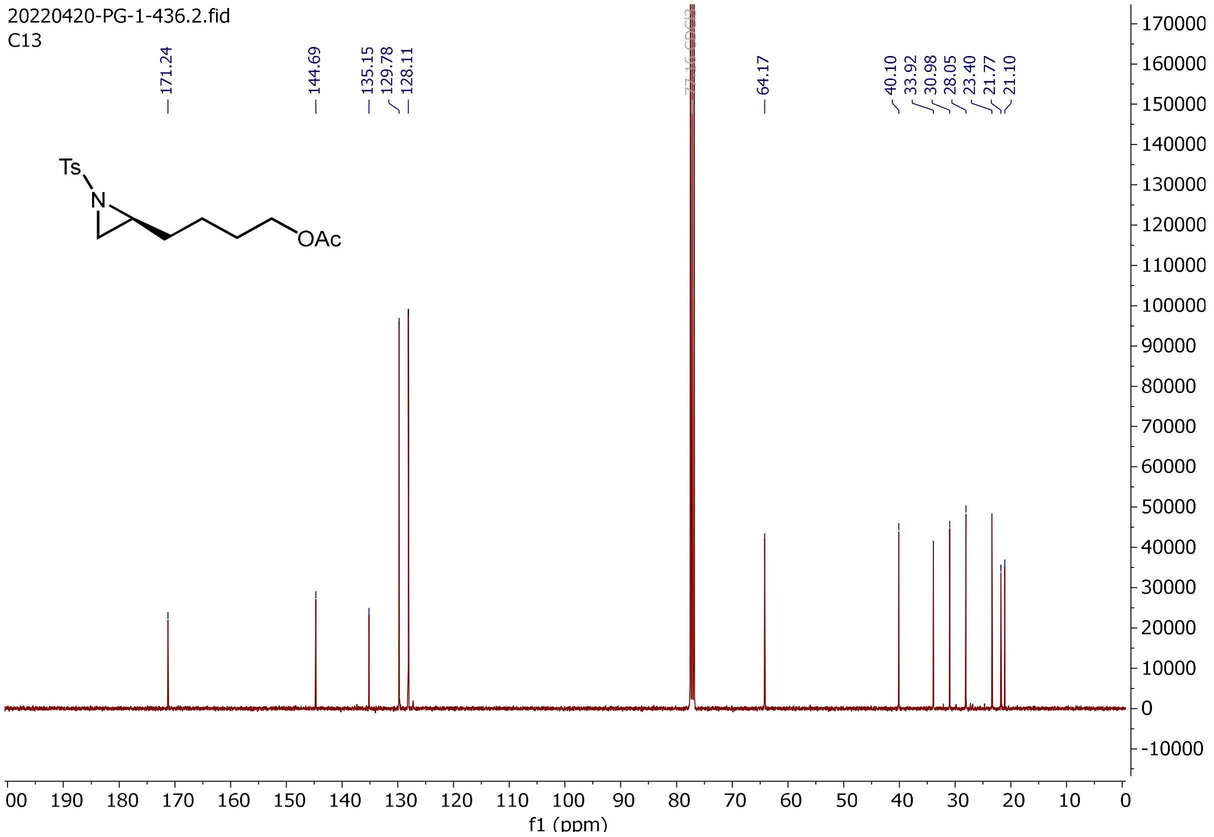

(±)-4-(1-tosylaziridin-2-yl)butyl acetate (±-**11**):

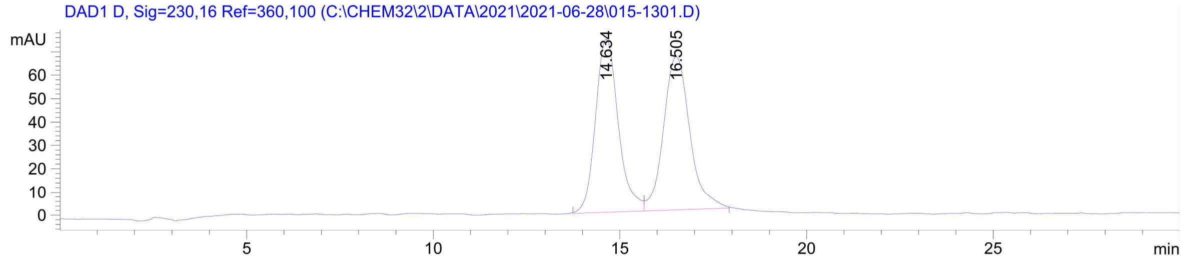

Signal 3: DAD1 D, Sig=230,16 Ref=360,100

Peak #	RetTime [min]	Type	Width [min]	Area [mAU*s]	Height [mAU]	Area %
1	14.634	BV	0.6631	3166.31494	74.01552	49.4688
2	16.505	VB	0.7644	3234.32153	65.69685	50.5312

Totals : 6400.63647 139.71237

(*S*)-4-(1-tosylaziridin-2-yl)butyl acetate (**11**):

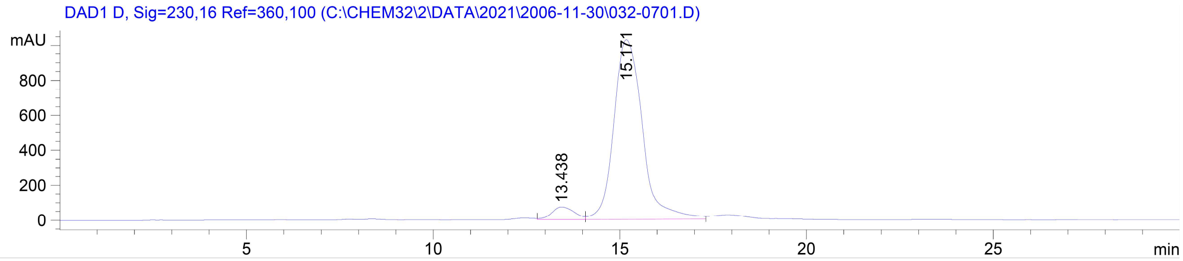

Signal 3: DAD1 D, Sig=230,16 Ref=360,100

Peak #	RetTime [min]	Type	Width [min]	Area [mAU*s]	Height [mAU]	Area %
1	13.438	VV	0.6679	3019.69897	72.20198	5.2439
2	15.171	VB	0.8474	5.45657e4	1027.76282	94.7561

Totals : 5.75854e4 1099.96480

(S)-4-(1-tosylaziridin-2-yl)butyl benzoate (**12**):

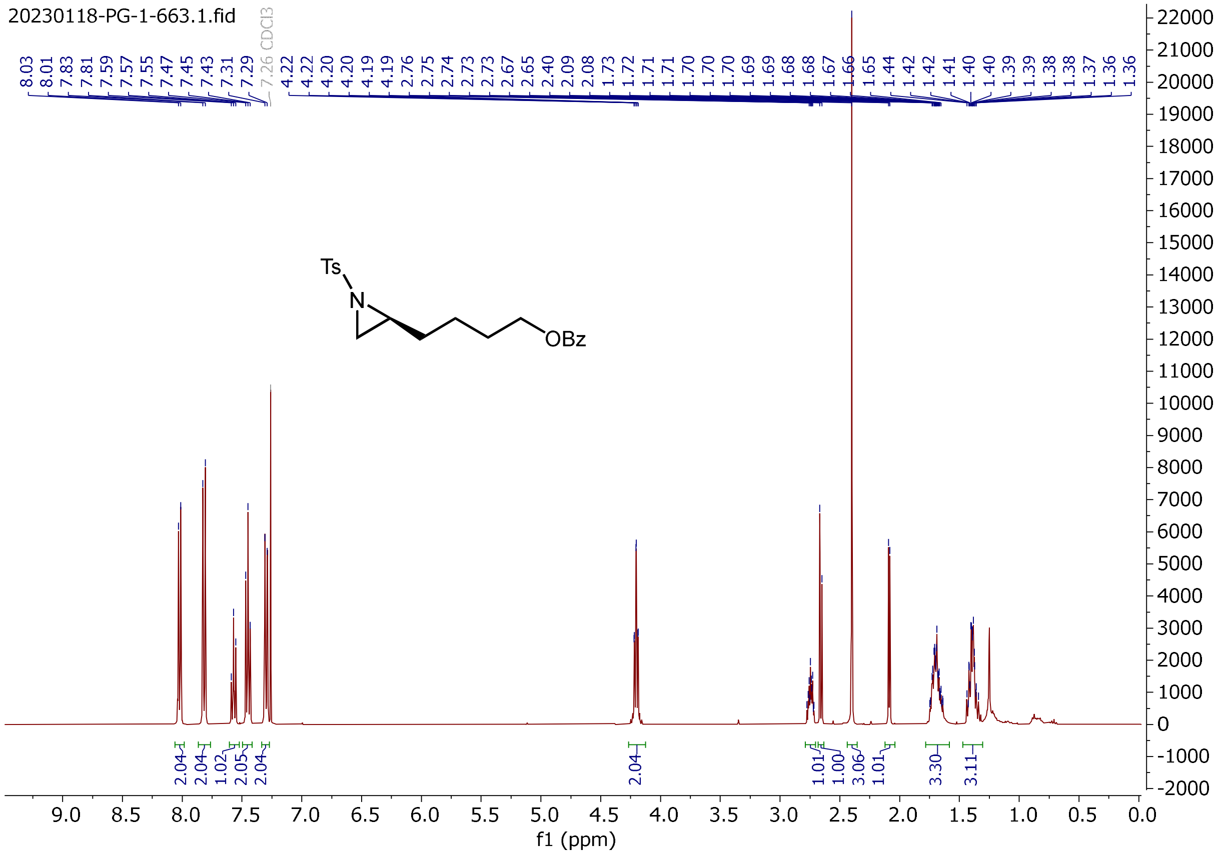

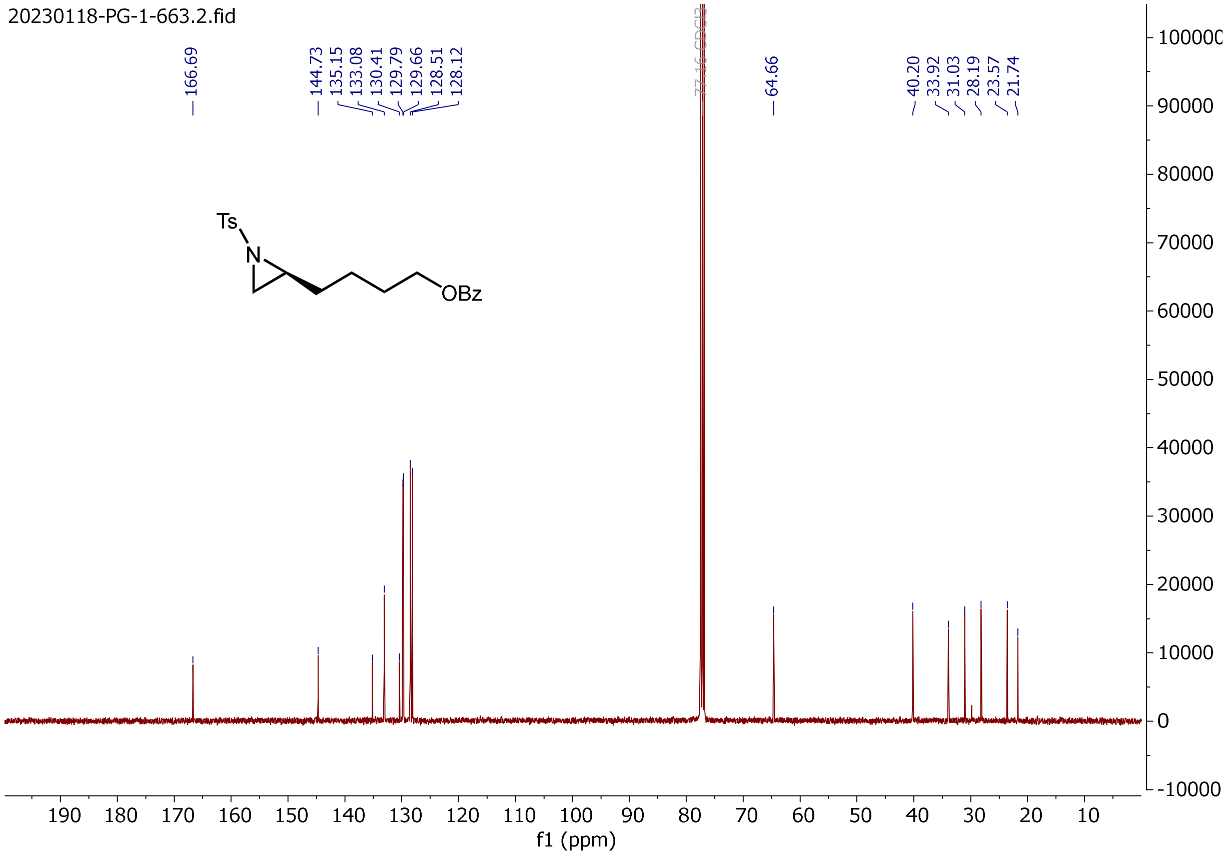

(±)-4-(1-tosylaziridin-2-yl)butyl benzoate (±-**12**):

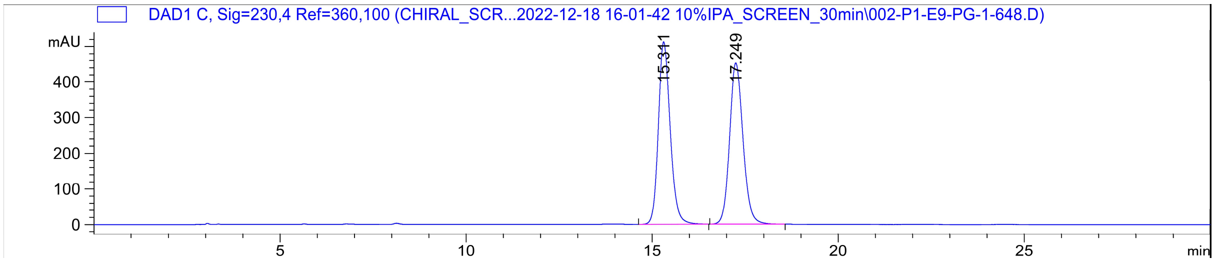

Signal 3: DAD1 C, Sig=230,4 Ref=360,100

Peak #	RetTime [min]	Type	Width [min]	Area [mAU*s]	Height [mAU]	Area %
1	15.311	BB	0.3338	1.13494e4	512.63116	50.0137
2	17.249	BB	0.3774	1.13432e4	452.59738	49.9863
Totals :				2.26926e4	965.22855	

(S)-4-(1-tosylaziridin-2-yl)butyl benzoate (**12**):

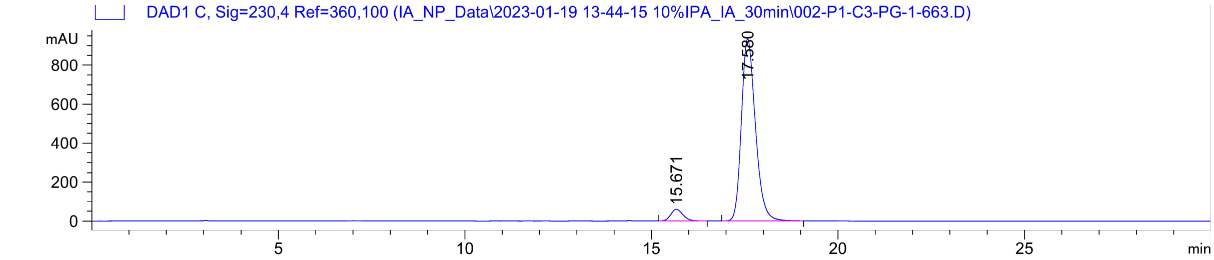

Signal 3: DAD1 C, Sig=230,4 Ref=360,100

Peak #	RetTime [min]	Type	Width [min]	Area [mAU*s]	Height [mAU]	Area %
1	15.671	BB	0.2826	1345.70996	59.34549	5.2351
2	17.580	BB	0.3925	2.43598e4	933.29297	94.7649
Totals :				2.57055e4	992.63846	

(*S*)-2-(4-((*tert*-butyldimethylsilyl)oxy)butyl)-1-tosylaziridine (**13**):

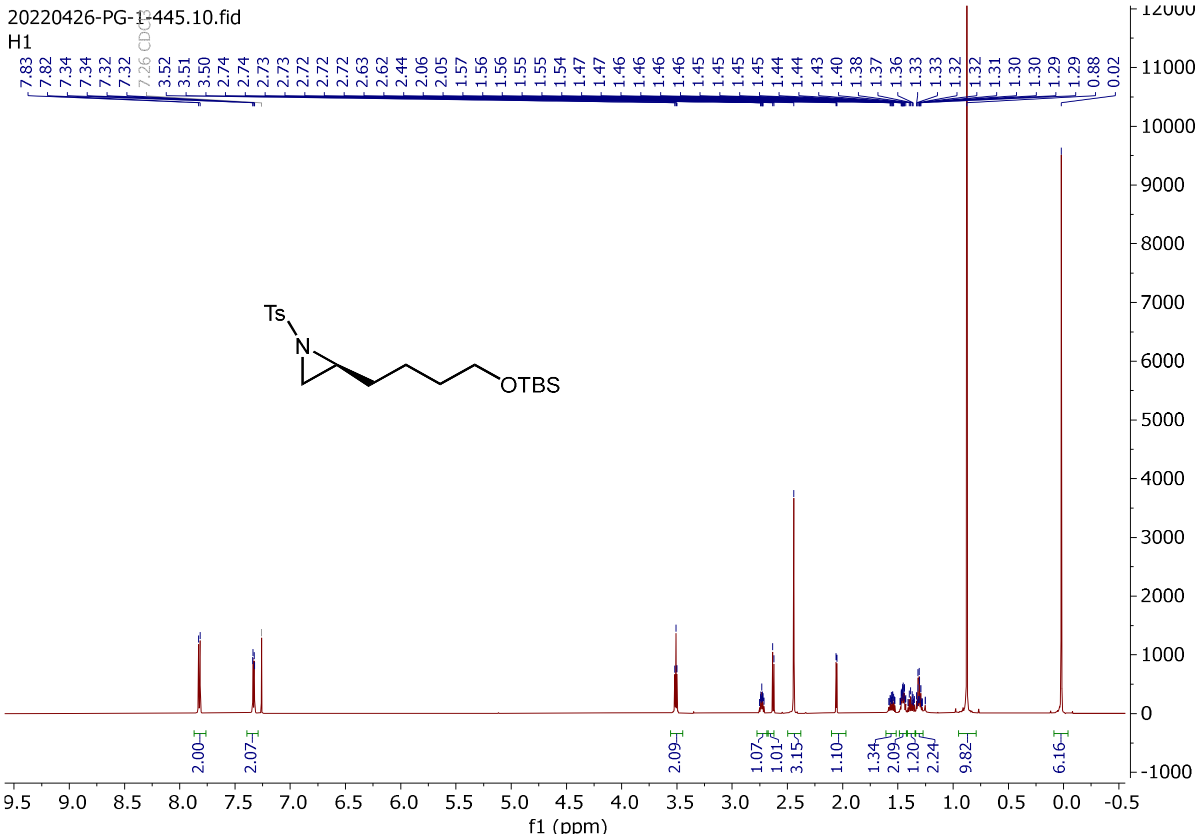

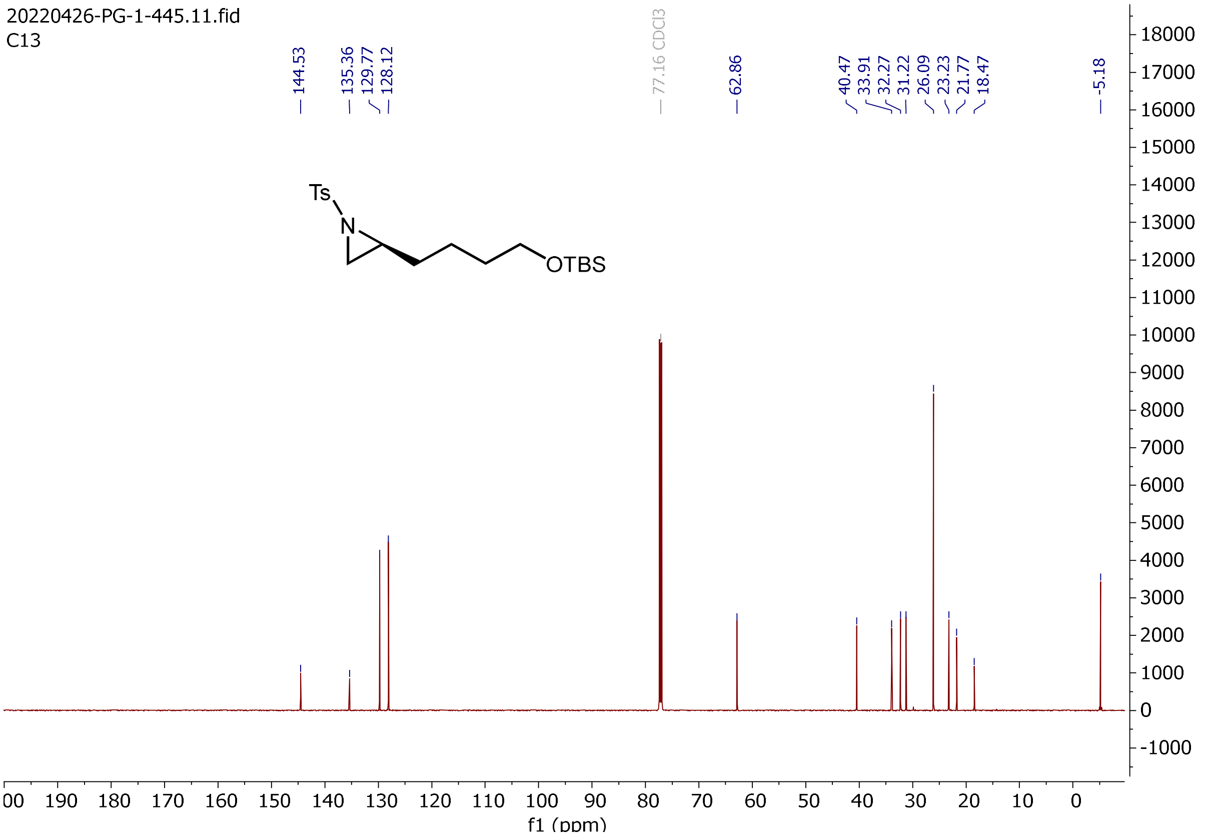

(±)-2-(4-((*tert*-butyldimethylsilyl)oxy)butyl)-1-tosylaziridine (±-**13**)

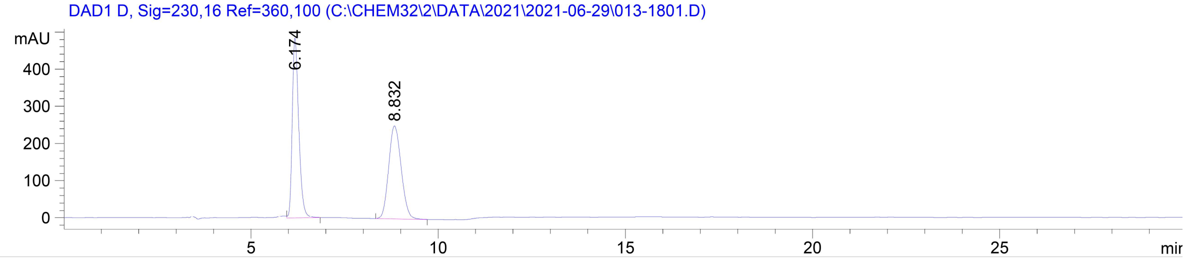

Signal 3: DAD1 D, Sig=230,16 Ref=360,100

Peak #	RetTime [min]	Type	Width [min]	Area [mAU*s]	Height [mAU]	Area %
1	6.174	VB	0.1861	5903.22070	484.65616	50.0539
2	8.832	BB	0.3657	5890.49951	251.13007	49.9461

Totals : 1.17937e4 735.78622

(*S*)-2-(4-((*tert*-butyldimethylsilyl)oxy)butyl)-1-tosylaziridine (**13**)

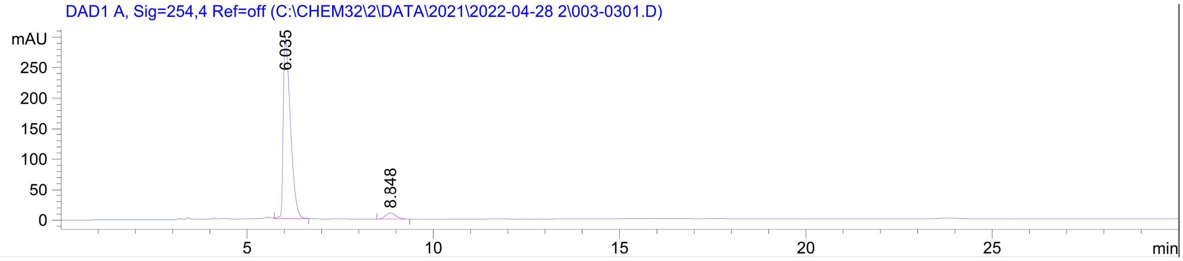

Signal 1: DAD1 A, Sig=254,4 Ref=off

Peak #	RetTime [min]	Type	Width [min]	Area [mAU*s]	Height [mAU]	Area %
1	6.035	VB	0.2058	3994.16357	295.37958	95.2330
2	8.848	BB	0.3062	199.93347	10.04337	4.7670

Totals : 4194.09705 305.42295

(*S*)-4-(1-tosylaziridin-2-yl)butyl 4-methylbenzenesulfonate (**14**):

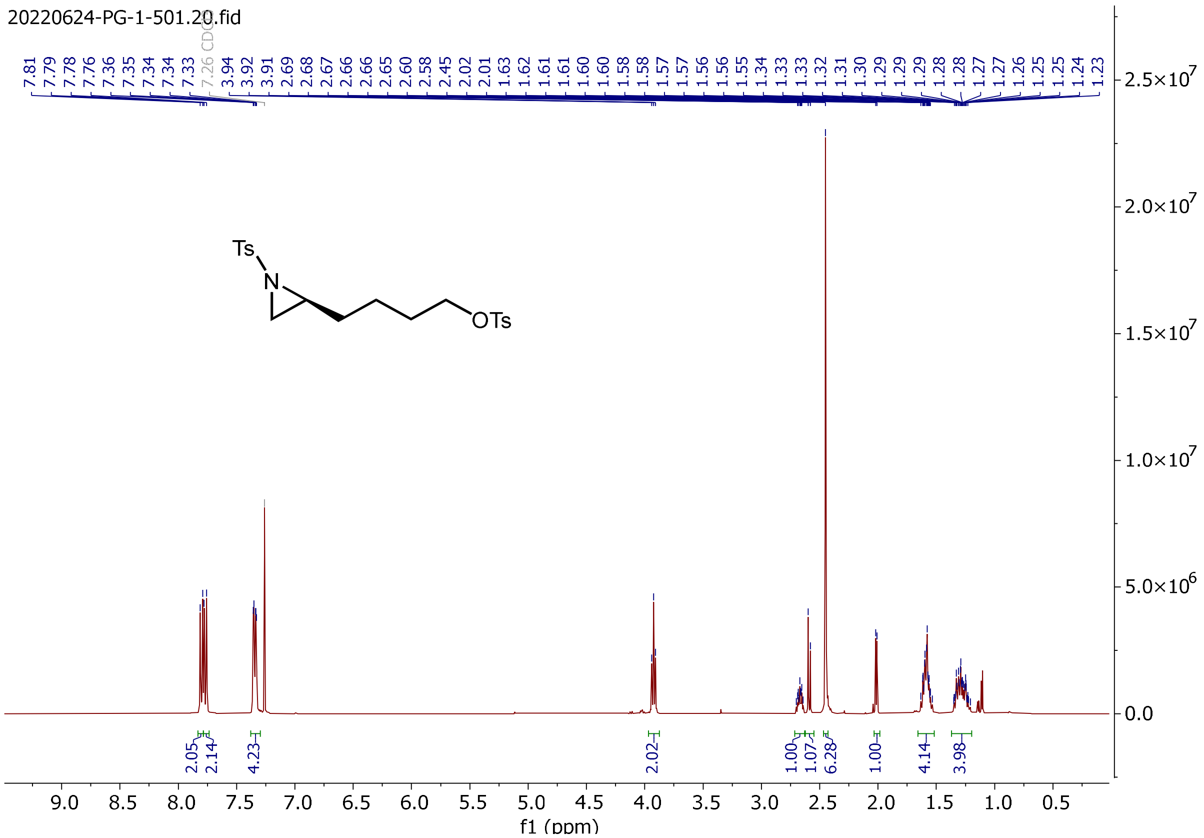

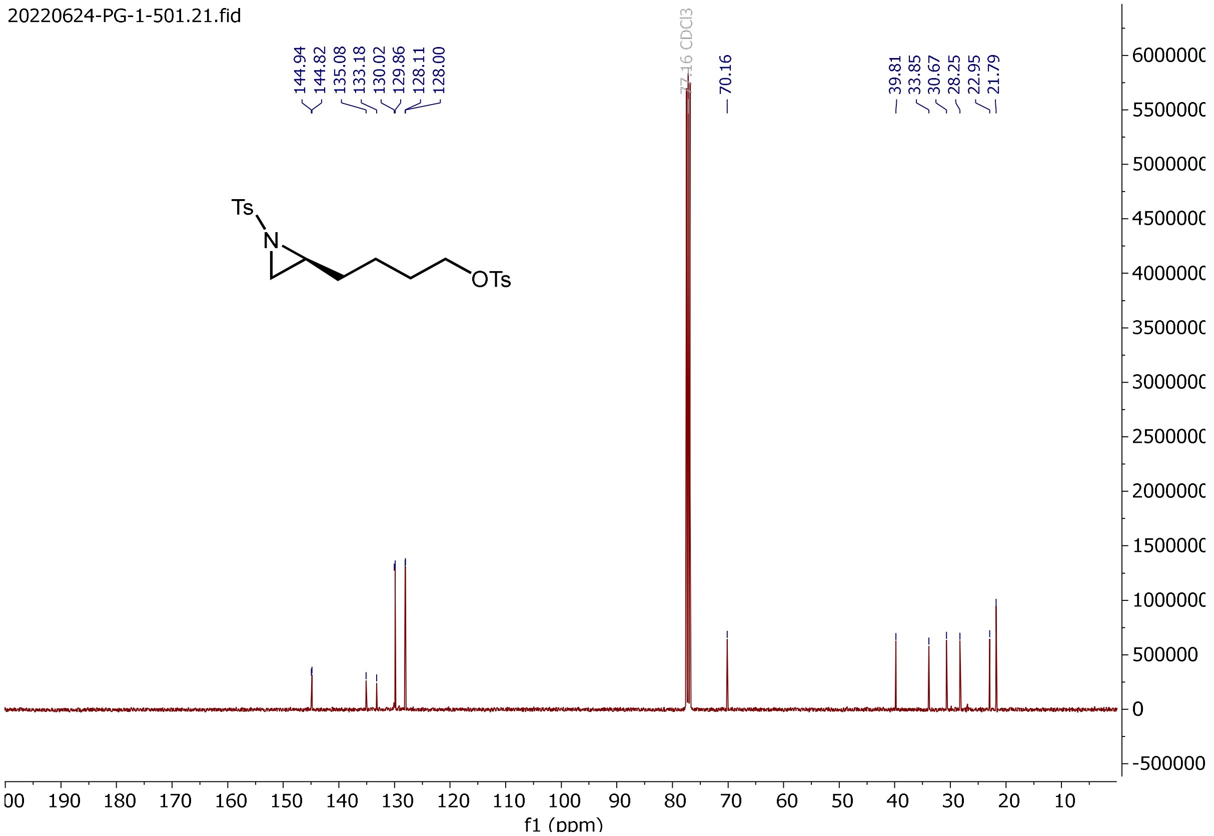

(±)-4-(1-tosylaziridin-2-yl)butyl 4-methylbenzenesulfonate (±-**14**):

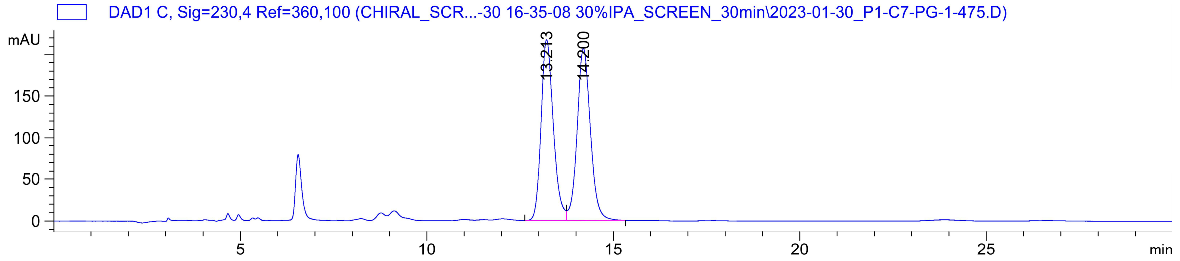

Signal 3: DAD1 C, Sig=230,4 Ref=360,100

Peak #	RetTime [min]	Type	Width [min]	Area [mAU*s]	Height [mAU]	Area %
1	13.213	BV	0.3481	4961.00391	217.13869	49.2370
2	14.200	VB	0.3607	5114.76611	206.68993	50.7630

Totals : 1.00758e4 423.82861

(*S*)-4-(1-tosylaziridin-2-yl)butyl 4-methylbenzenesulfonate (**14**):

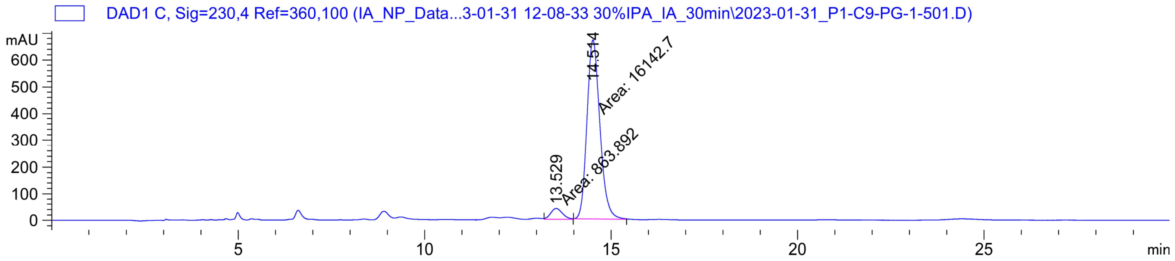

Signal 3: DAD1 C, Sig=230,4 Ref=360,100

Peak #	RetTime [min]	Type	Width [min]	Area [mAU*s]	Height [mAU]	Area %
1	13.529	MM	0.3624	863.89215	39.72470	5.0797
2	14.514	MM	0.4019	1.61427e4	669.45471	94.9203

Totals : 1.70066e4 709.17942

(*S*)-4-(1-tosylaziridin-2-yl)butan-1-ol (**15**):

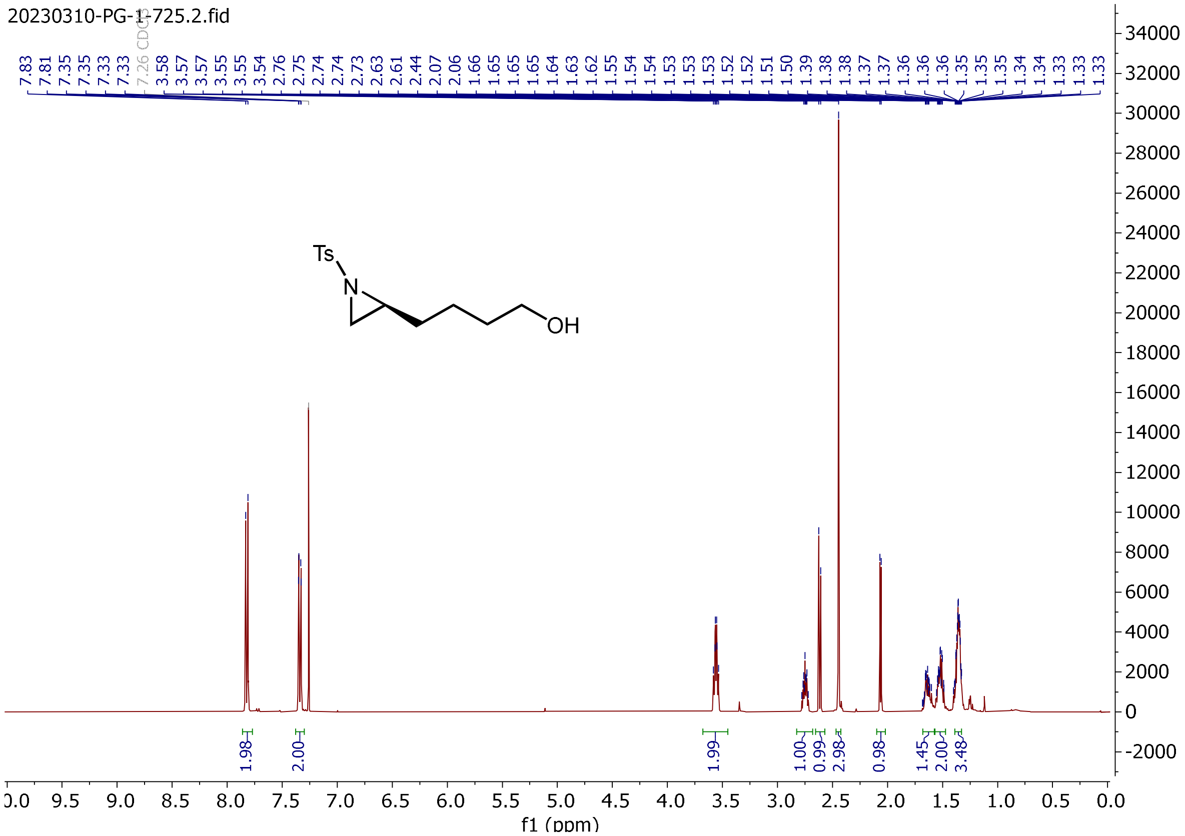

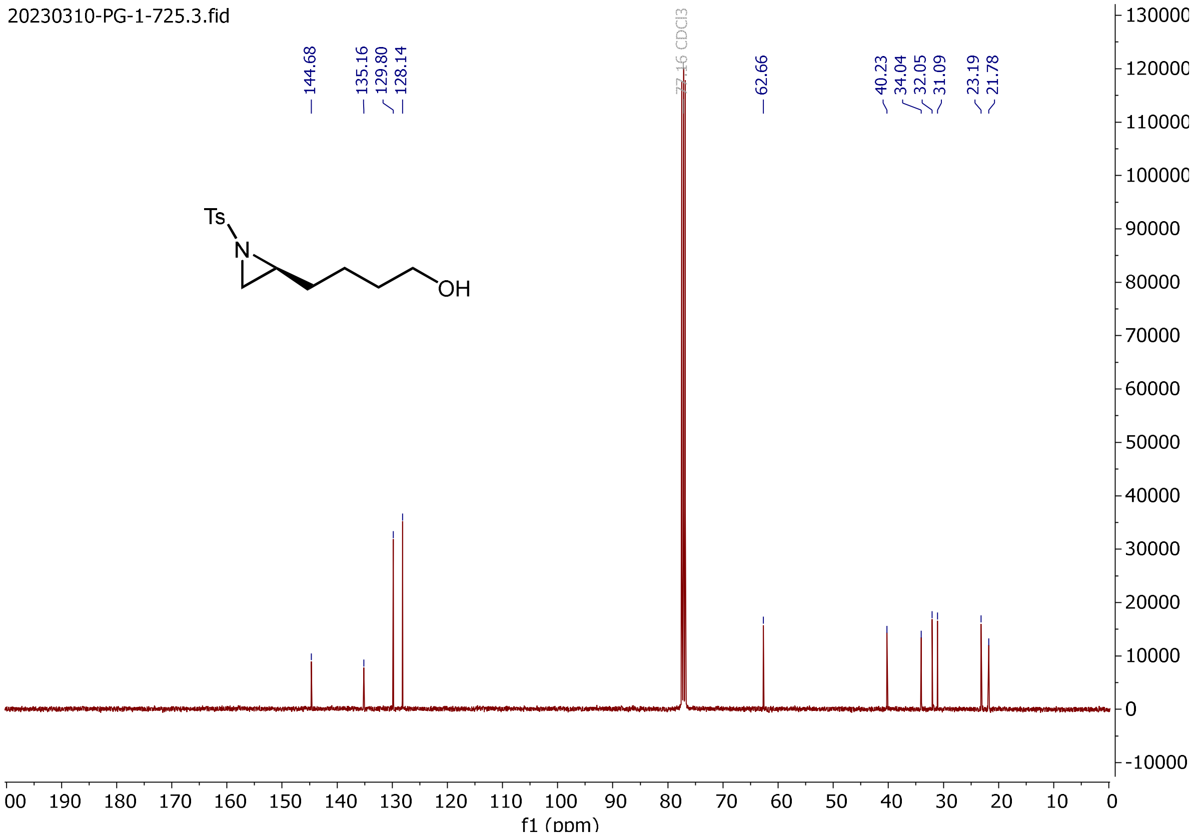

(±)-4-(1-tosylaziridin-2-yl)butan-1-ol (±-**15**):

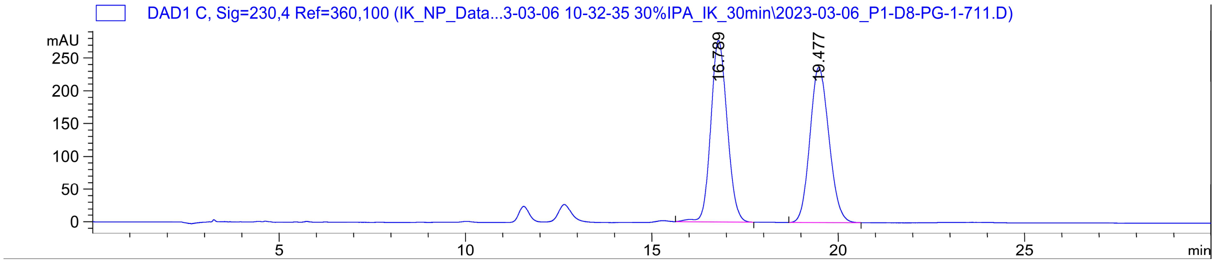

Signal 3: DAD1 C, Sig=230,4 Ref=360,100

Peak #	RetTime [min]	Type	Width [min]	Area [mAU*s]	Height [mAU]	Area %
1	16.789	VB R	0.4366	8467.90723	277.20874	50.1483
2	19.477	BB	0.4526	8417.83301	236.88470	49.8517
Totals :				1.68857e4	514.09344	

(*S*)-4-(1-tosylaziridin-2-yl)butan-1-ol (**15**):

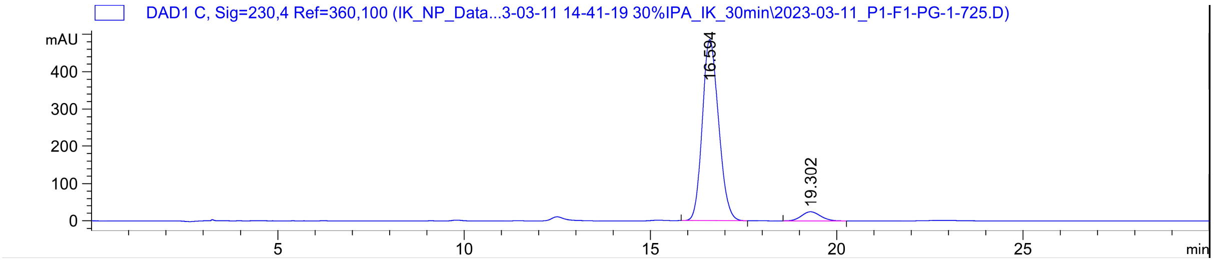

Signal 3: DAD1 C, Sig=230,4 Ref=360,100

Peak #	RetTime [min]	Type	Width [min]	Area [mAU*s]	Height [mAU]	Area %
1	16.594	BB	0.4493	1.47459e4	485.49707	94.5369
2	19.302	BB	0.4127	852.13507	24.17467	5.4631
Totals :				1.55981e4	509.67174	

(S)-2-(4-(1-tosylaziridin-2-yl)butyl)isoindoline-1,3-dione (**16**):

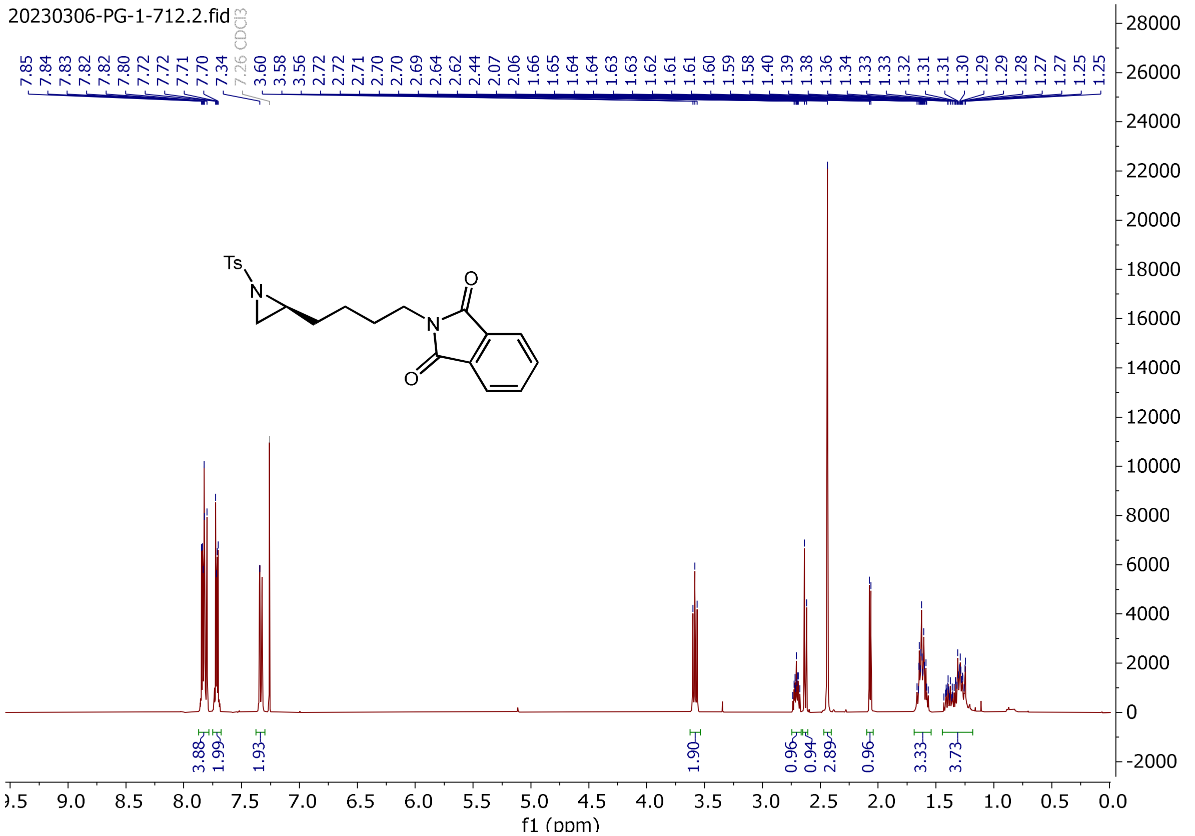

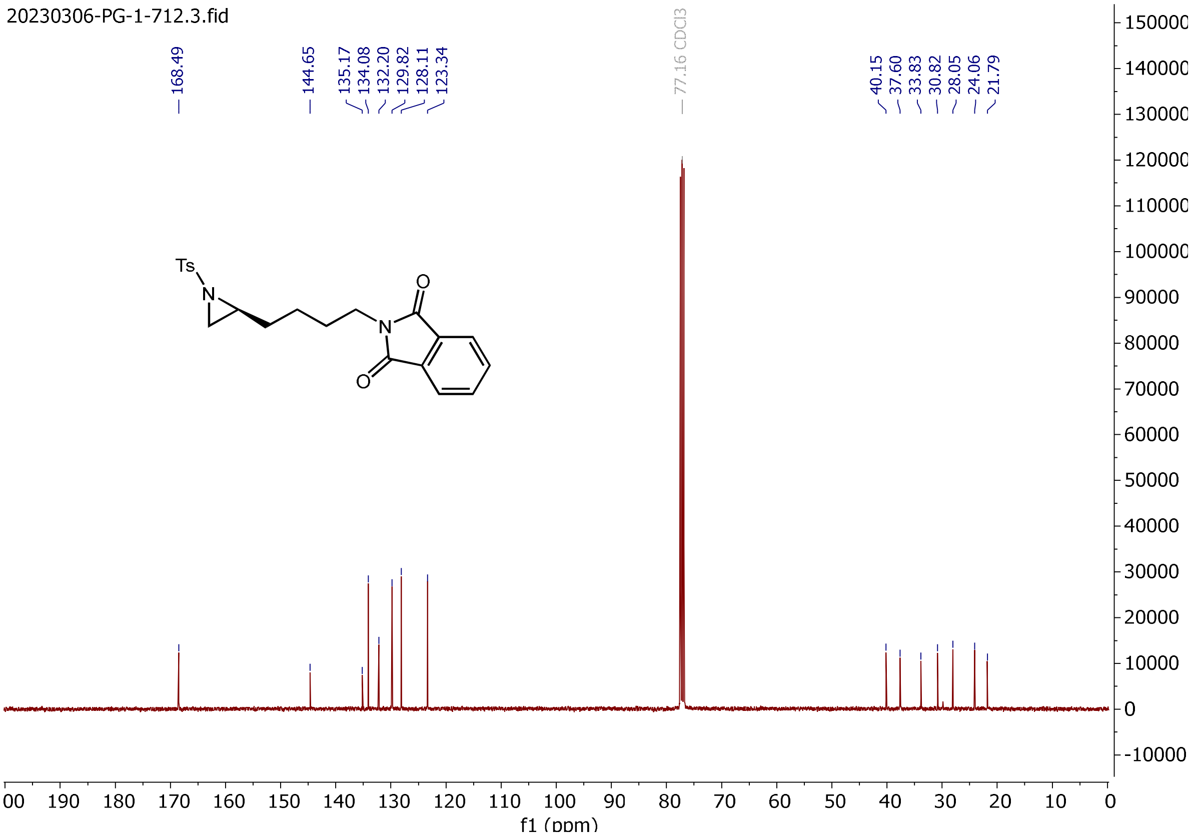

(±)-2-(4-(1-tosylaziridin-2-yl)butyl)isoindoline-1,3-dione (±-**16**):

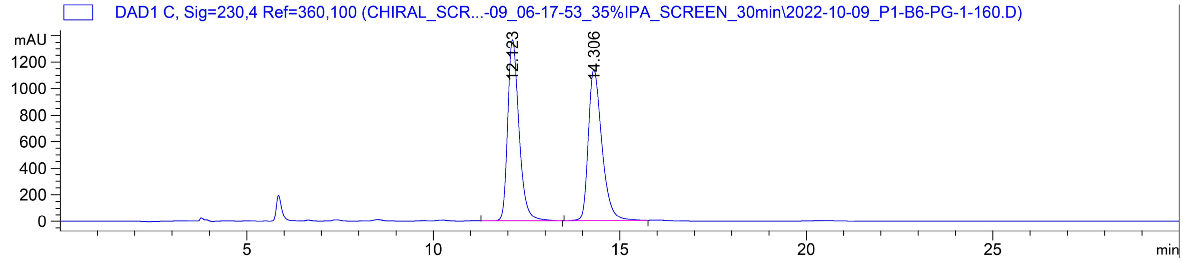

Signal 3: DAD1 C, Sig=230,4 Ref=360,100

Peak #	RetTime [min]	Type	Width [min]	Area [mAU*s]	Height [mAU]	Area %
1	12.123	BB	0.3200	2.87536e4	1366.31616	49.9879
2	14.306	BB	0.3813	2.87675e4	1138.56714	50.0121

Totals : 5.75211e4 2504.88330

(S)-2-(4-(1-tosylaziridin-2-yl)butyl)isoindoline-1,3-dione (**16**):

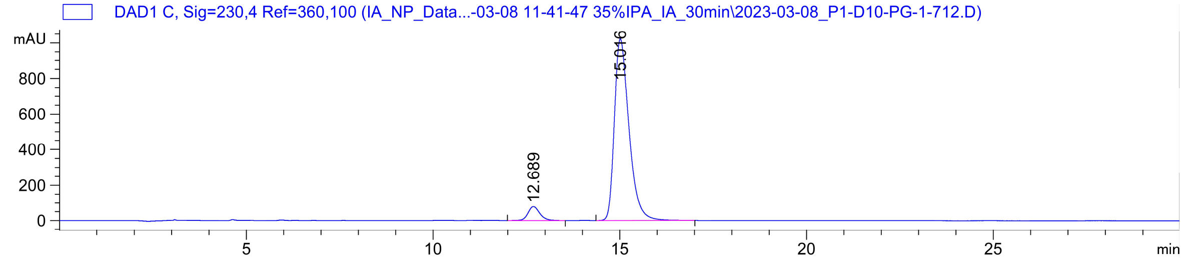

Signal 3: DAD1 C, Sig=230,4 Ref=360,100

Peak #	RetTime [min]	Type	Width [min]	Area [mAU*s]	Height [mAU]	Area %
1	12.689	BB	0.2821	1732.11353	78.28905	5.8859
2	15.016	BB	0.4030	2.76961e4	1020.82062	94.1141

Totals : 2.94283e4 1099.10967

(*S*)-N-(4-(1-tosylaziridin-2-yl)butyl)acetamide (**17**):

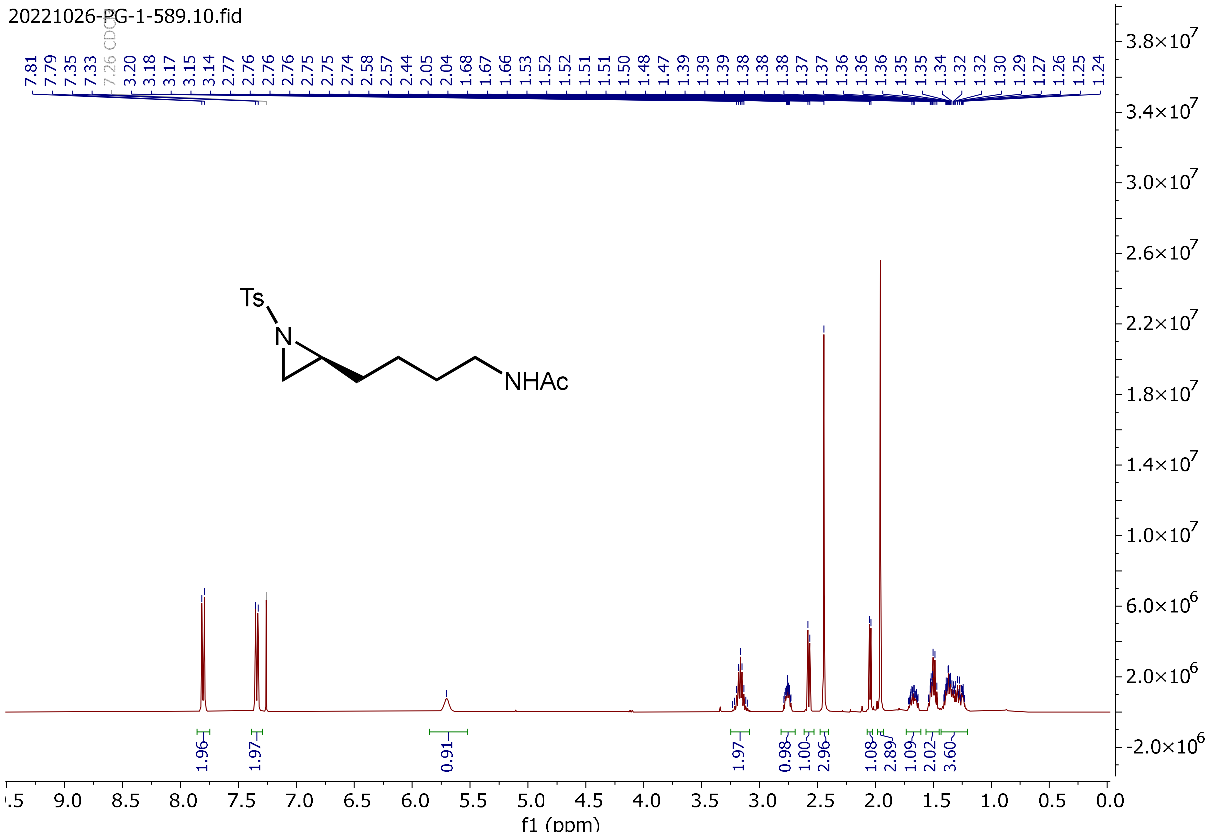

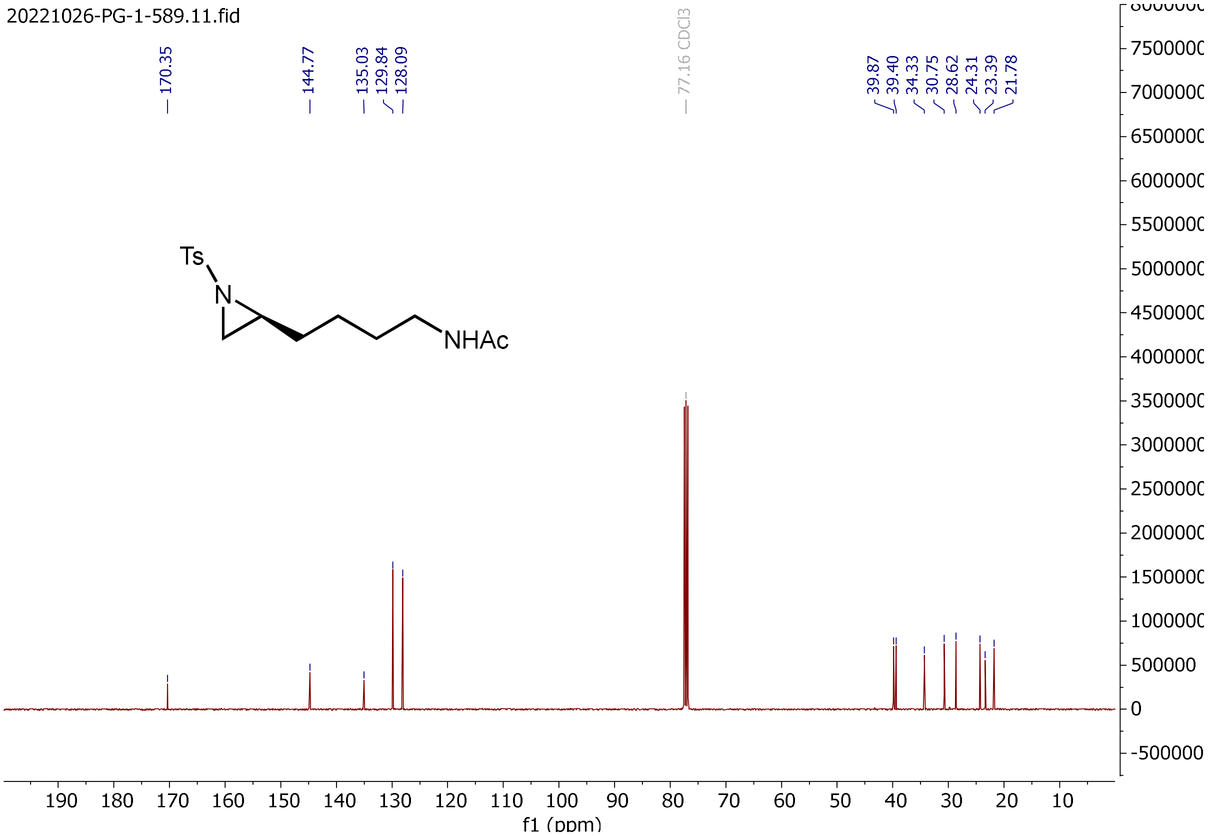

(±)-N-(4-(1-tosylaziridin-2-yl)butyl)acetamide (±-**17**):

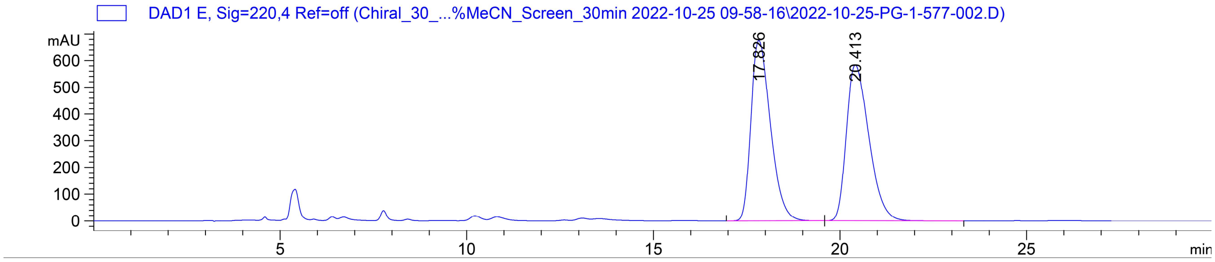

Signal 5: DAD1 E, Sig=220,4 Ref=off

Peak #	RetTime [min]	Type	Width [min]	Area [mAU*s]	Height [mAU]	Area %
1	17.826	BB	0.5576	2.44017e4	675.31604	49.9622
2	20.413	BB	0.6510	2.44386e4	583.09021	50.0378
Totals :				4.88403e4	1258.40625	

(*S*)-N-(4-(1-tosylaziridin-2-yl)butyl)acetamide (**17**):

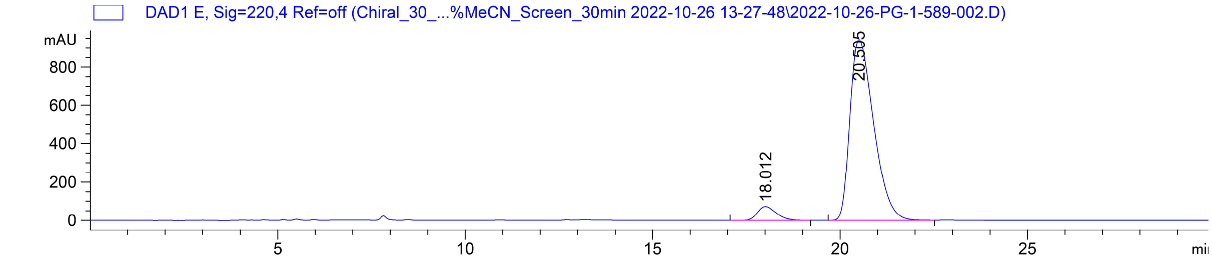

Signal 5: DAD1 E, Sig=220,4 Ref=off

Peak #	RetTime [min]	Type	Width [min]	Area [mAU*s]	Height [mAU]	Area %
1	18.012	BB	0.5342	2510.60913	72.10947	5.7082
2	20.505	BB	0.6849	4.14717e4	943.26465	94.2918
Totals :				4.39823e4	1015.37411	

(S)-2-(4-nitrobutyl)-1-tosylaziridine (**18**):

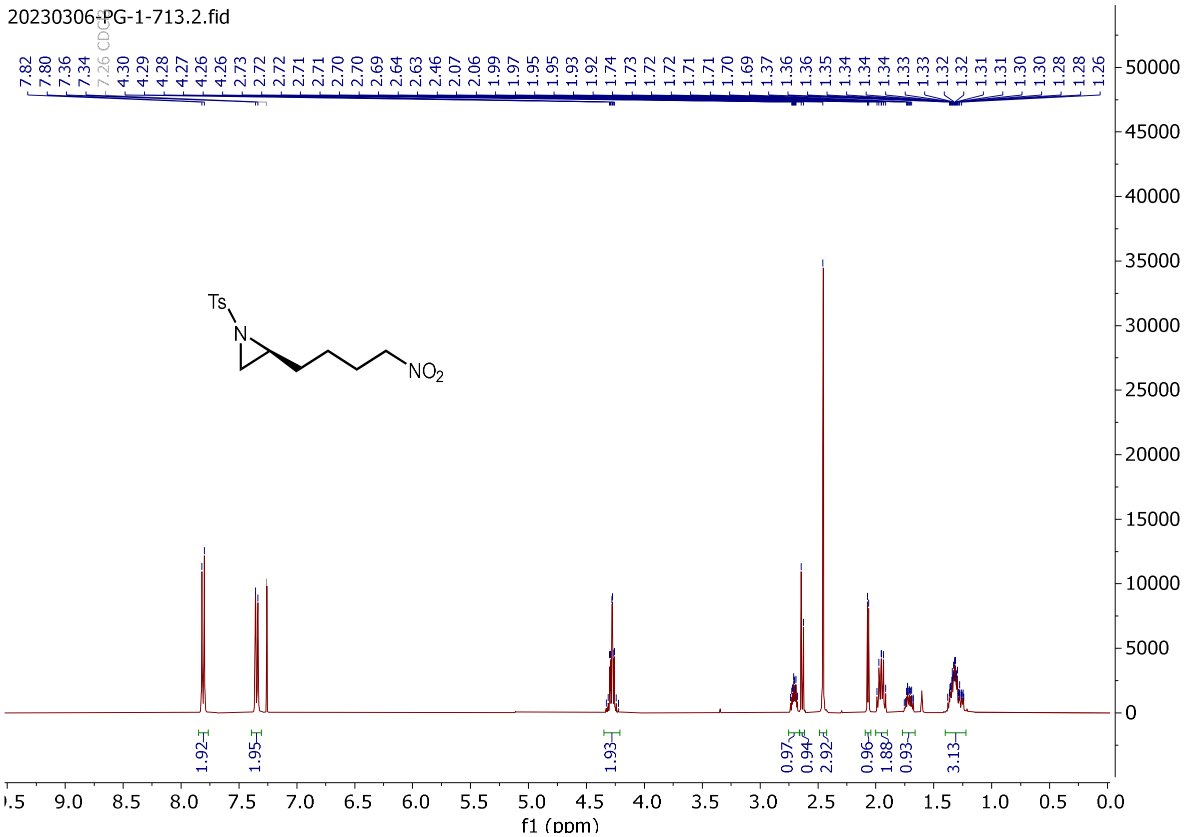

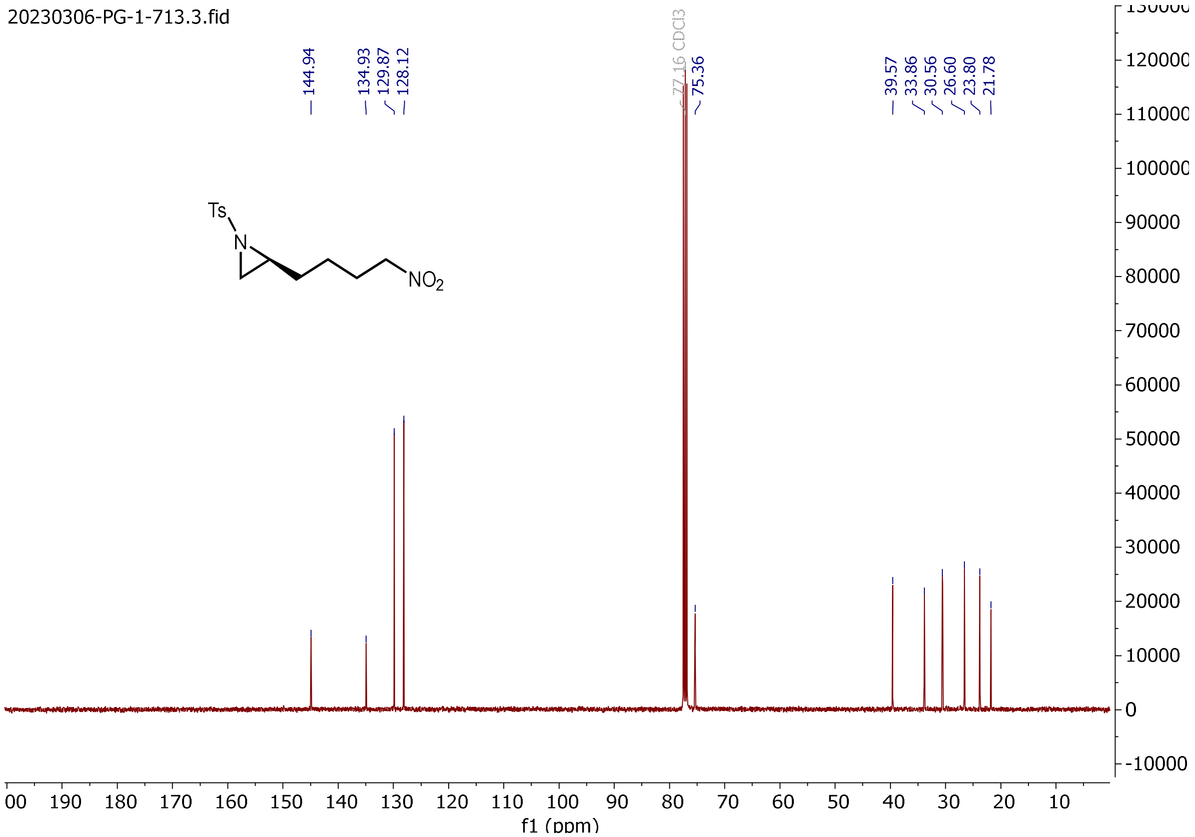

(±)-2-(4-nitrobutyl)-1-tosylaziridine (±-**18**):

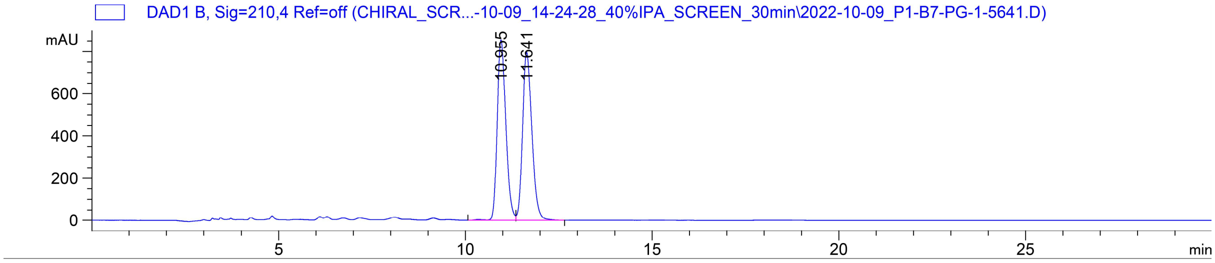

Signal 2: DAD1 B, Sig=210,4 Ref=off

Peak #	RetTime [min]	Type	Width [min]	Area [mAU*s]	Height [mAU]	Area %
1	10.955	VV R	0.2467	1.37106e4	855.09778	49.8023
2	11.641	VB	0.2678	1.38195e4	794.91217	50.1977

Totals : 2.75301e4 1650.00995

(S)-2-(4-nitrobutyl)-1-tosylaziridine (**18**):

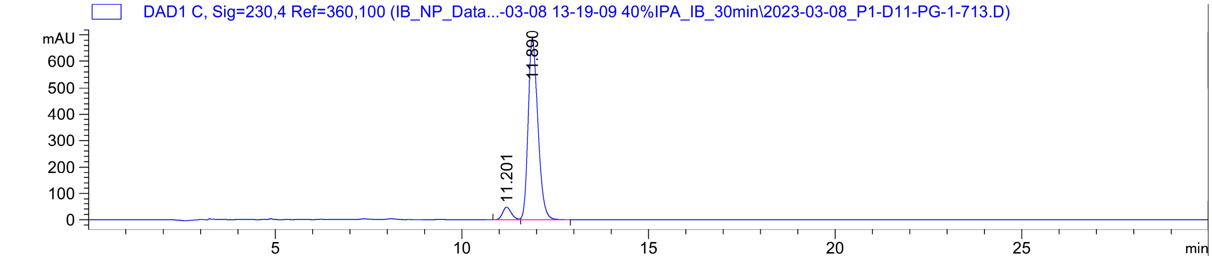

Signal 3: DAD1 C, Sig=230,4 Ref=360,100

Peak #	RetTime [min]	Type	Width [min]	Area [mAU*s]	Height [mAU]	Area %
1	11.201	BV E	0.2231	758.55176	47.05001	5.7328
2	11.890	VB R	0.2792	1.24732e4	685.73492	94.2672

Totals : 1.32318e4 732.78493

*(S)-2-(4-bromobutyl)-1-tosylaziridine (**19**)*:

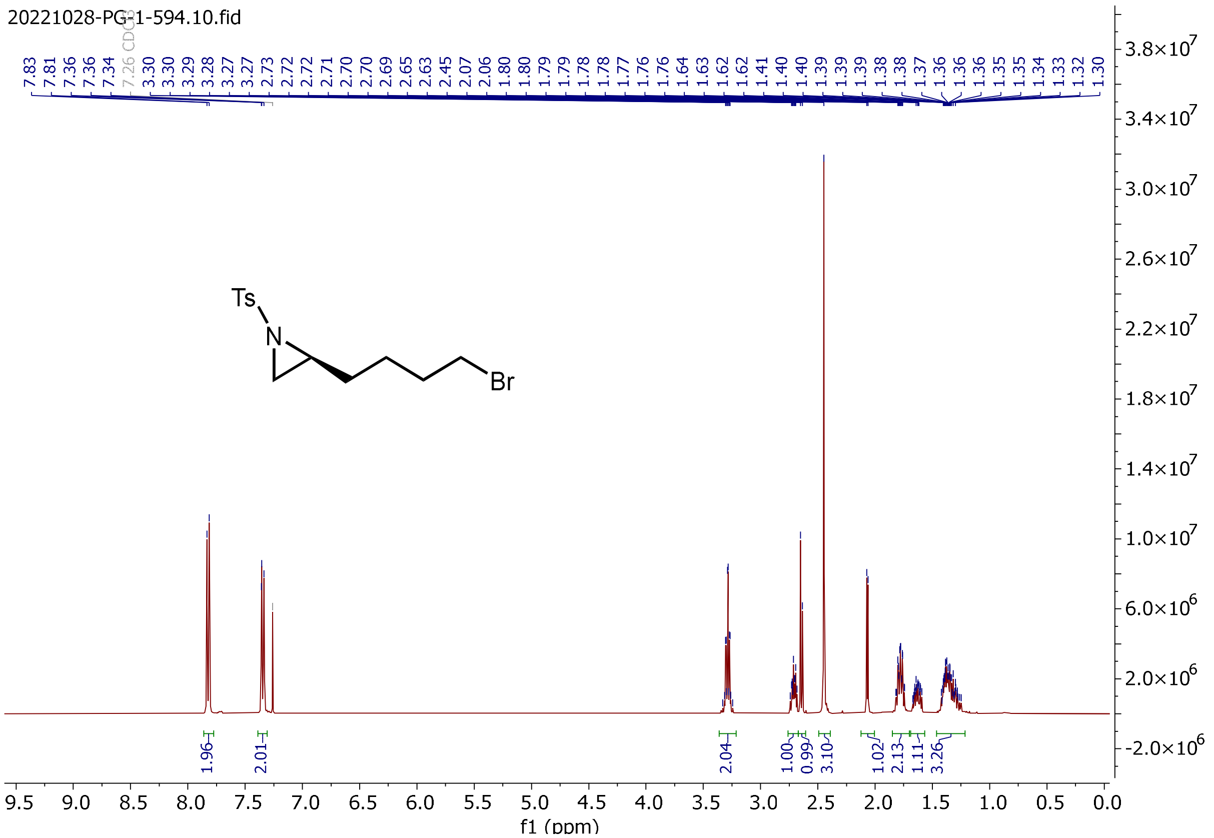

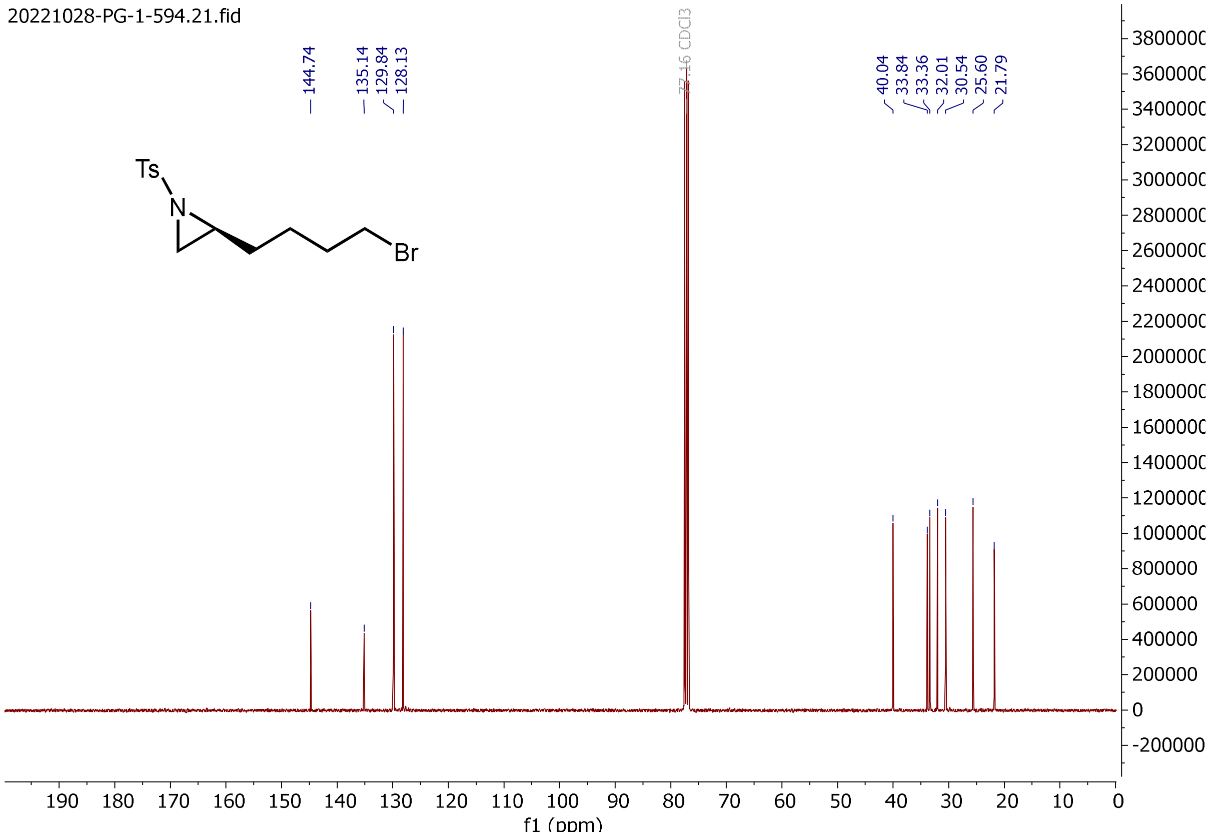

*(±)-2-(4-bromobutyl)-1-tosylaziridine (±-**19**):*

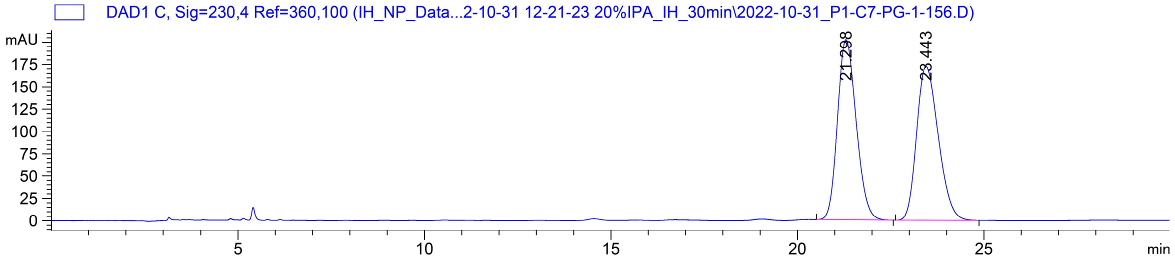

Signal 3: DAD1 C, Sig=230,4 Ref=360,100

Peak #	RetTime [min]	Type	Width [min]	Area [mAU*s]	Height [mAU]	Area %
1	21.298	BB	0.4907	7028.21191	201.85909	50.2308
2	23.443	BB	0.4848	6963.62256	172.38828	49.7692

Totals : 1.39918e4 374.24736

*(S)-2-(4-bromobutyl)-1-tosylaziridine (**19**):*

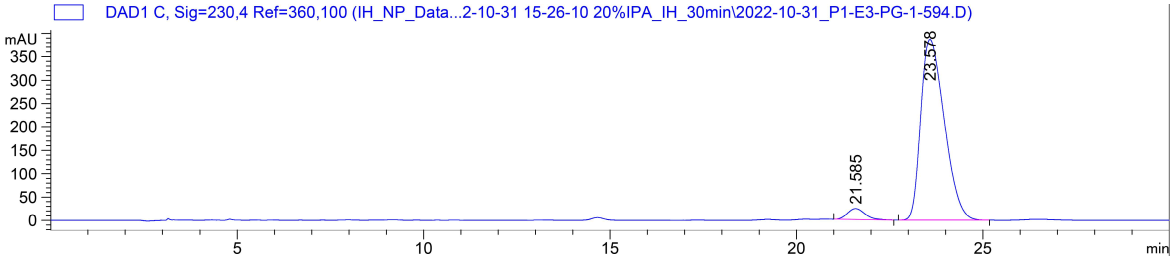

Signal 3: DAD1 C, Sig=230,4 Ref=360,100

Peak #	RetTime [min]	Type	Width [min]	Area [mAU*s]	Height [mAU]	Area %
1	21.585	BB	0.3903	769.99579	23.46990	4.3422
2	23.578	BB	0.6332	1.69627e4	386.33487	95.6578

Totals : 1.77327e4 409.80477

(S)-2-(4-(4,4,5,5-tetramethyl-1,3,2-dioxaborolan-2-yl)butyl)-1-tosylaziridine (**20**):

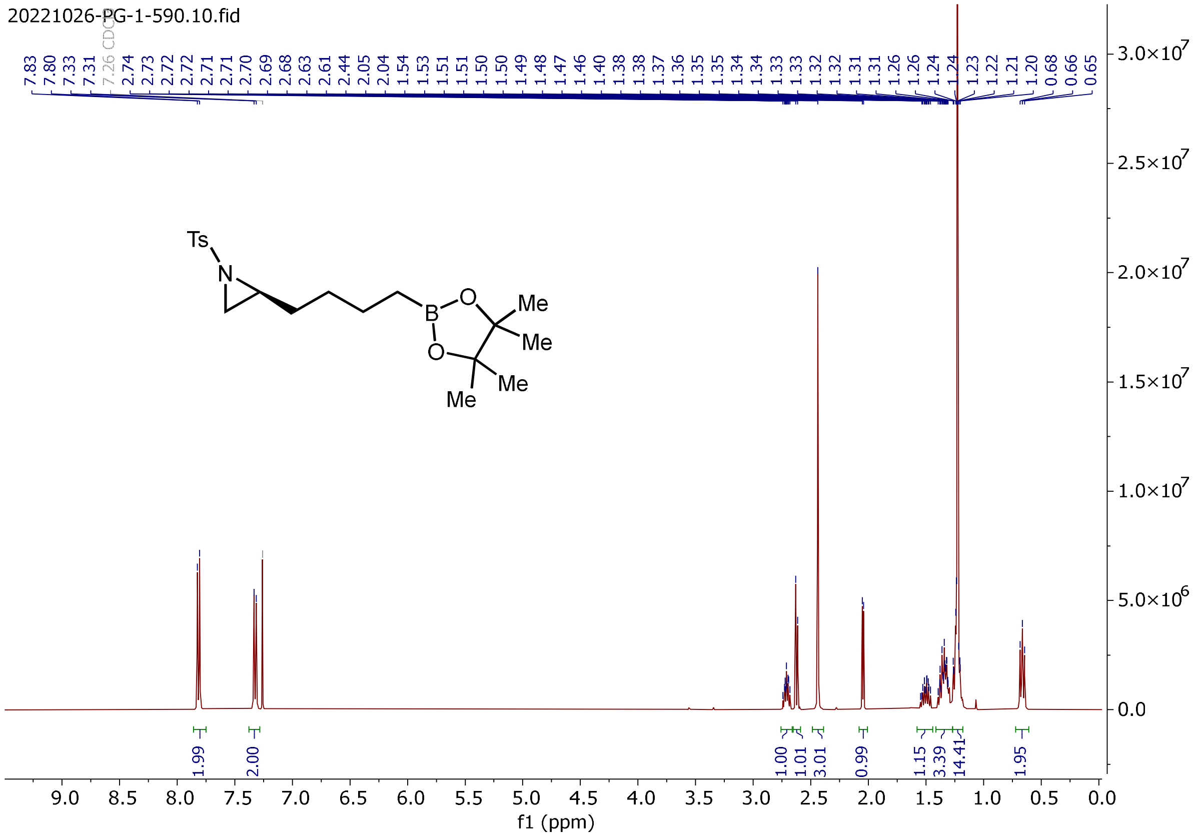

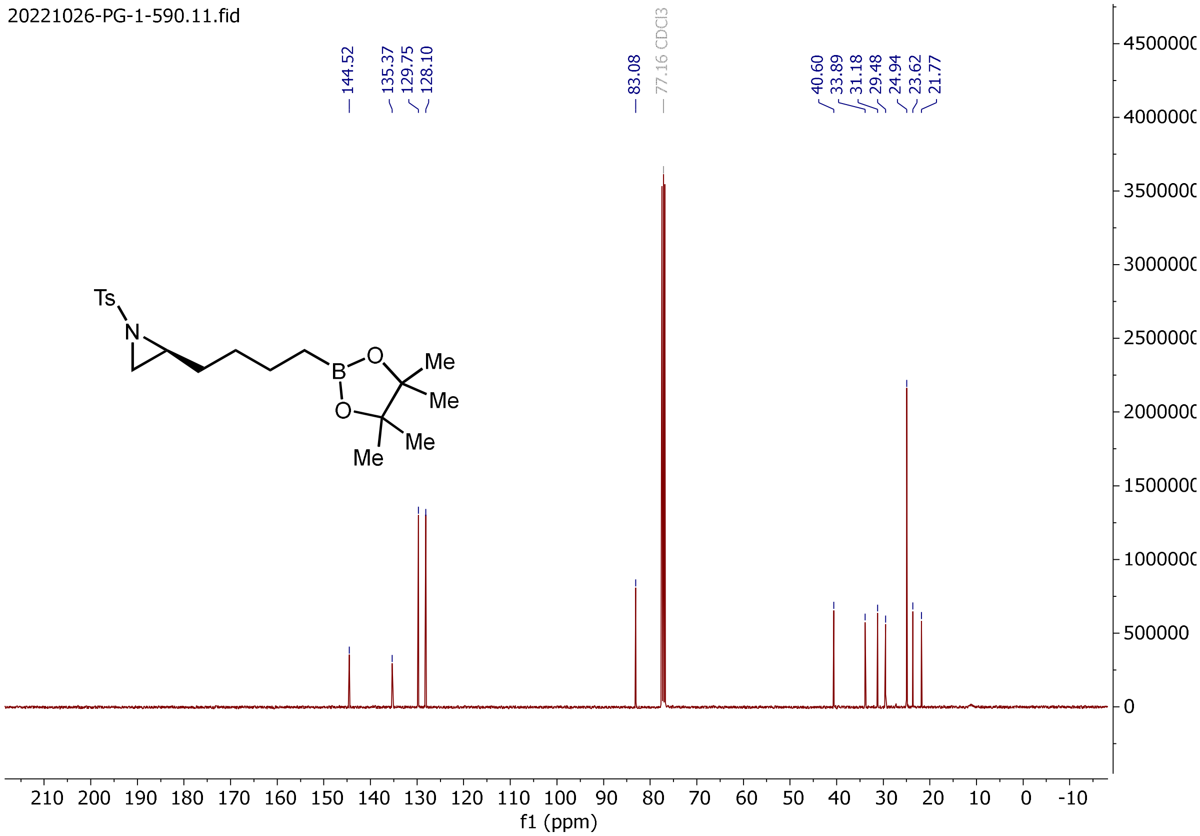

(±)-2-(4-(4,4,5,5-tetramethyl-1,3,2-dioxaborolan-2-yl)butyl)-1-tosylaziridine (±-**20**):

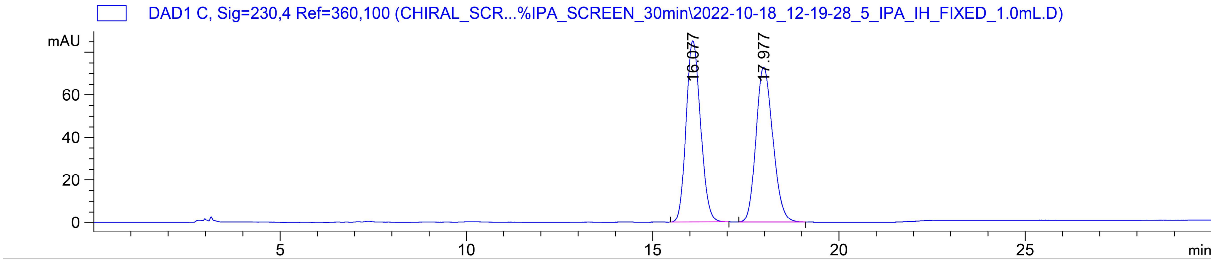

Signal 3: DAD1 C, Sig=230,4 Ref=360,100

Peak #	RetTime [min]	Type	Width [min]	Area [mAU*s]	Height [mAU]	Area %
1	16.077	BB	0.3867	2298.20679	85.32497	50.0368
2	17.977	BB	0.4201	2294.82422	72.93842	49.9632

Totals : 4593.03101 158.26340

(S)-2-(4-(4,4,5,5-tetramethyl-1,3,2-dioxaborolan-2-yl)butyl)-1-tosylaziridine (**20**):

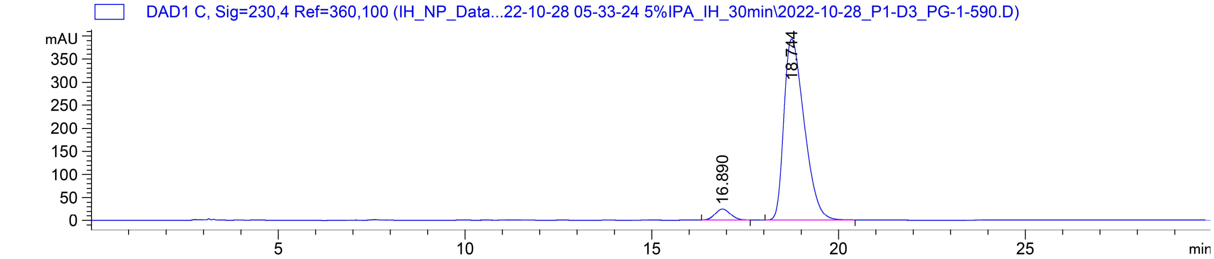

Signal 3: DAD1 C, Sig=230,4 Ref=360,100

Peak #	RetTime [min]	Type	Width [min]	Area [mAU*s]	Height [mAU]	Area %
1	16.890	BB	0.3312	688.79572	24.48606	4.4435
2	18.744	BB	0.5245	1.48123e4	392.72430	95.5565

Totals : 1.55011e4 417.21036

diethyl (*S*)-(4-(1-tosylaziridin-2-yl)butyl)phosphonate (**21**):

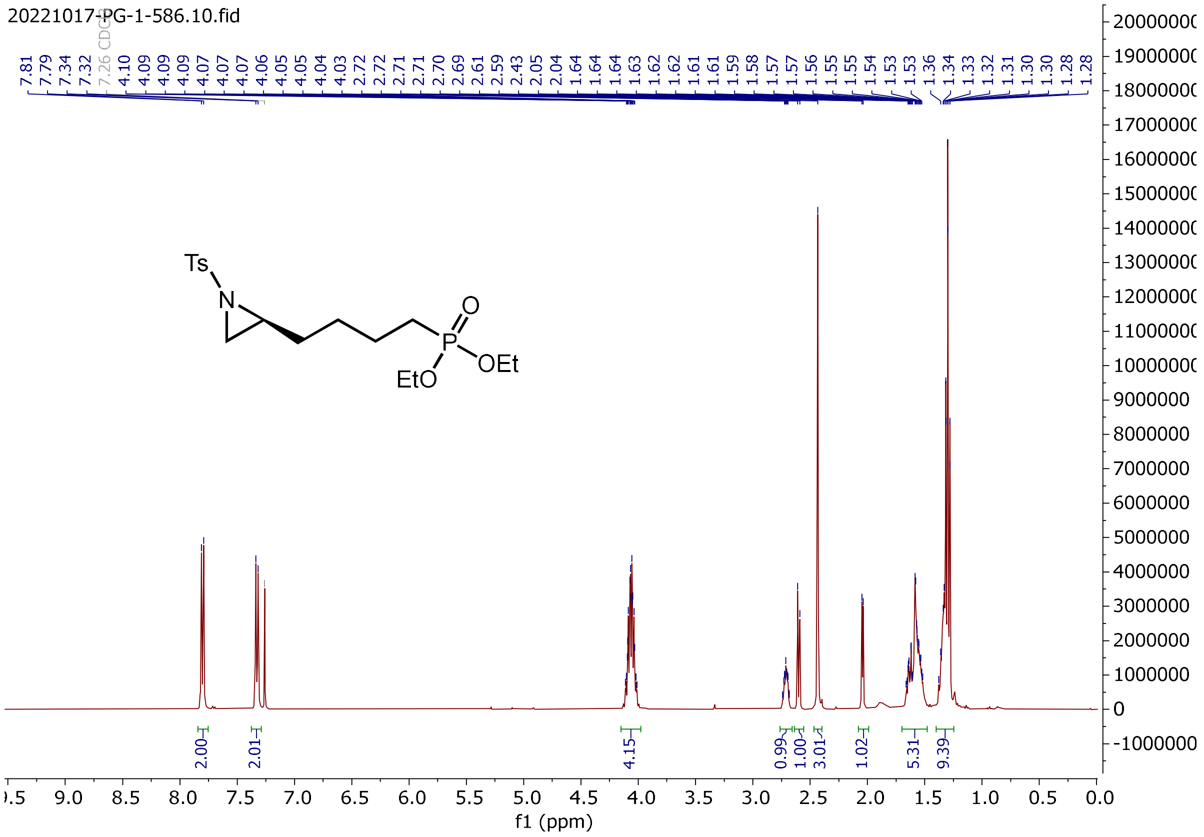

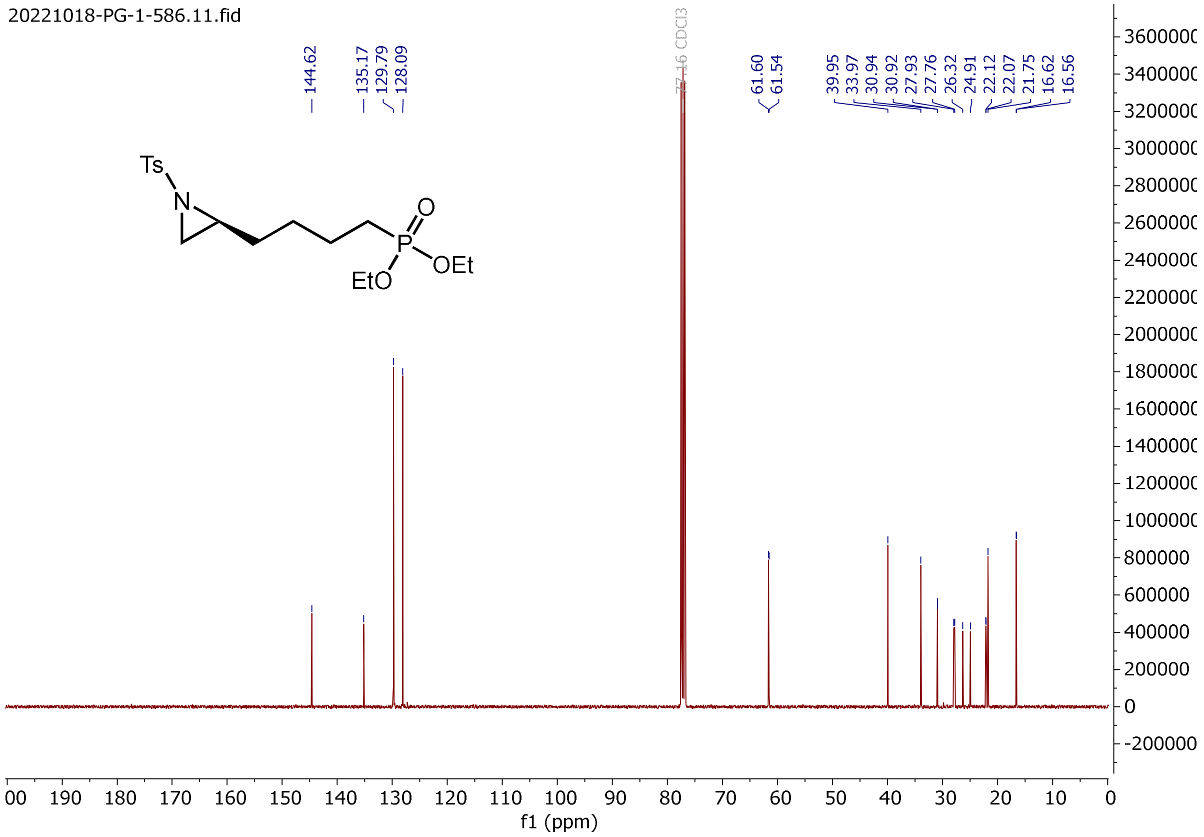

ethyl (*S*)-(4-(1-tosylaziridin-2-yl)butyl)phosphonate (**21**):

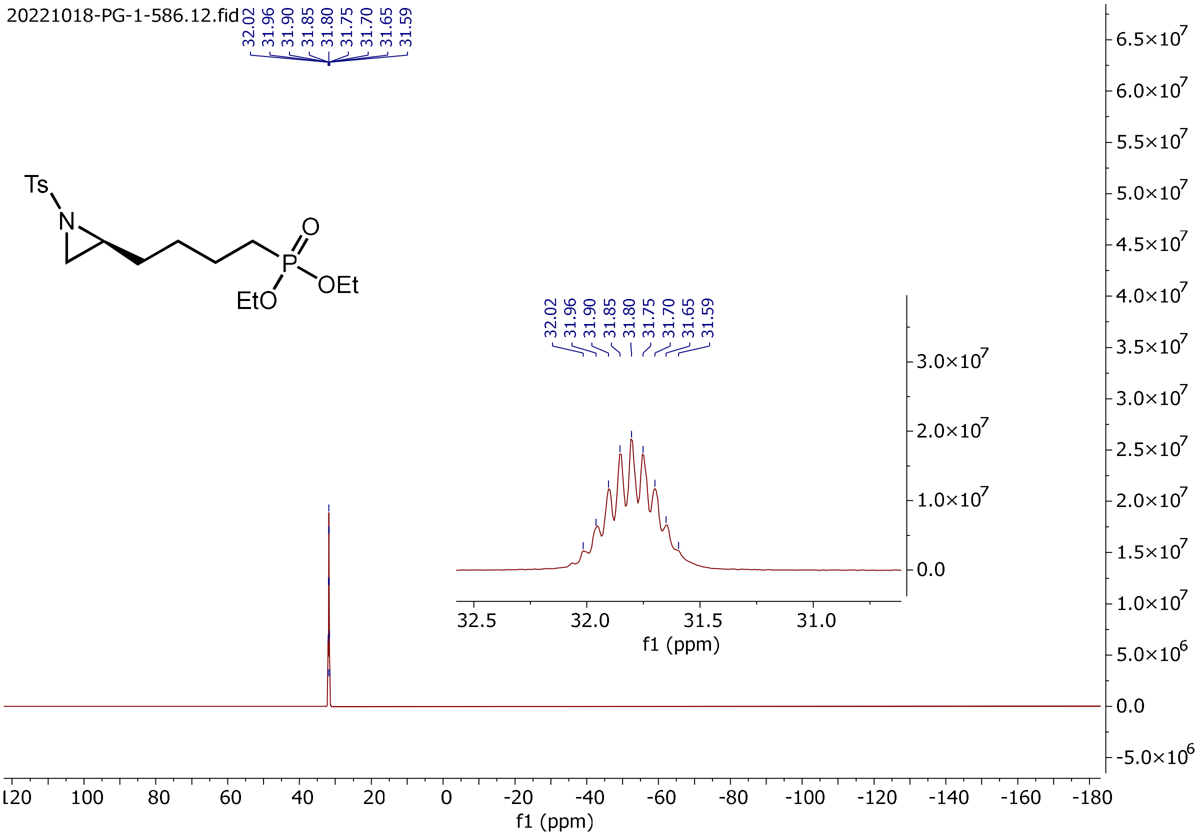

ethyl (±)-(4-(1-tosylaziridin-2-yl)butyl)phosphonate (±-**21**):

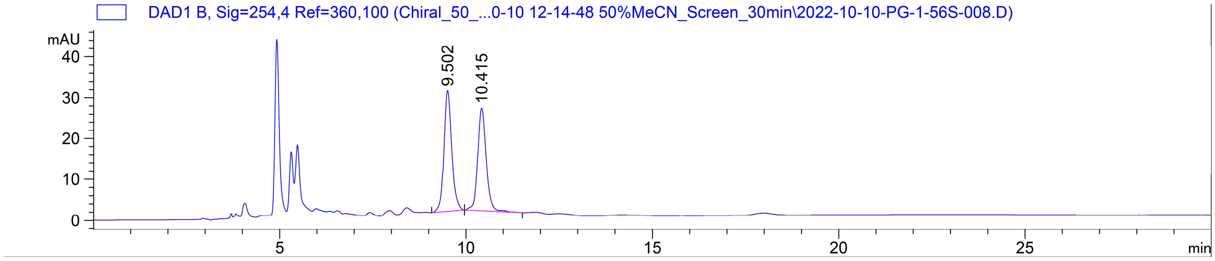

Signal 2: DAD1 B, Sig=254,4 Ref=360,100

Peak #	RetTime [min]	Type	Width [min]	Area [mAU*s]	Height [mAU]	Area %
1	9.502	BB	0.2194	429.23535	29.55379	51.9796
2	10.415	BB	0.2402	396.54083	25.11118	48.0204
Totals :				825.77618	54.66497	

ethyl (*S*)-(4-(1-tosylaziridin-2-yl)butyl)phosphonate (**21**):

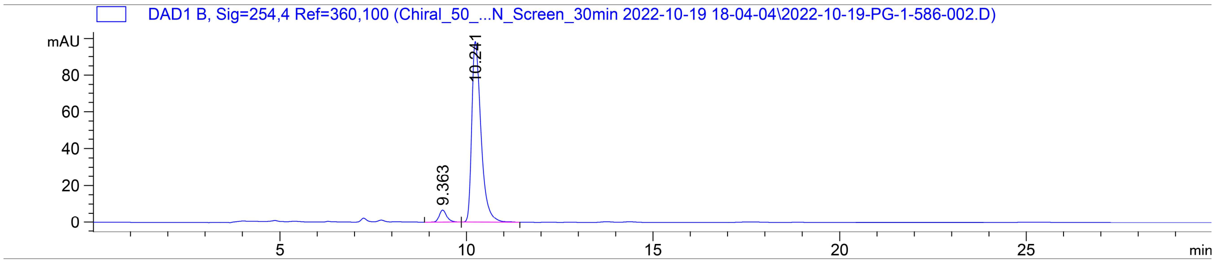

Signal 2: DAD1 B, Sig=254,4 Ref=360,100

Peak #	RetTime [min]	Type	Width [min]	Area [mAU*s]	Height [mAU]	Area %
1	9.363	BB	0.2139	95.79315	6.81980	5.4138
2	10.241	BB	0.2585	1673.62292	98.38892	94.5862
Totals :				1769.41608	105.20871	

(S)-4-(1-tosylaziridin-2-yl)butyl morpholine-4-carboxylate (**22**):

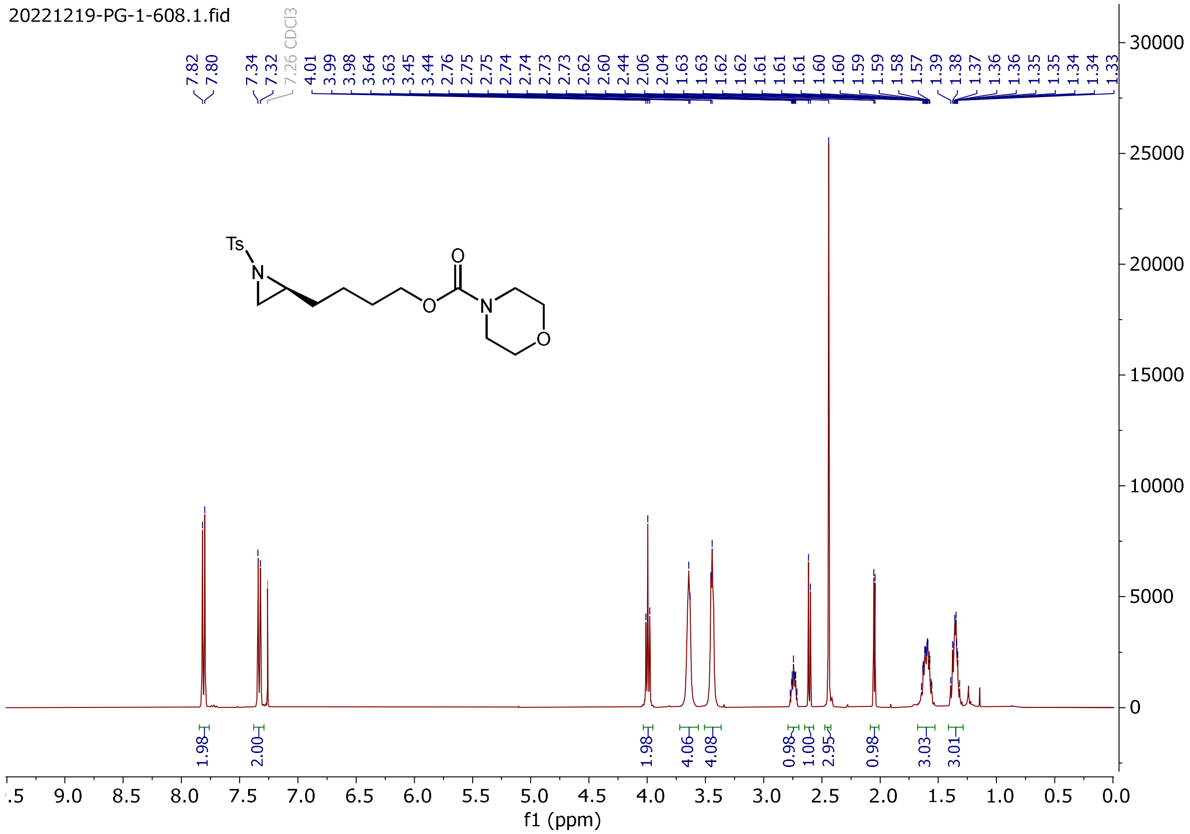

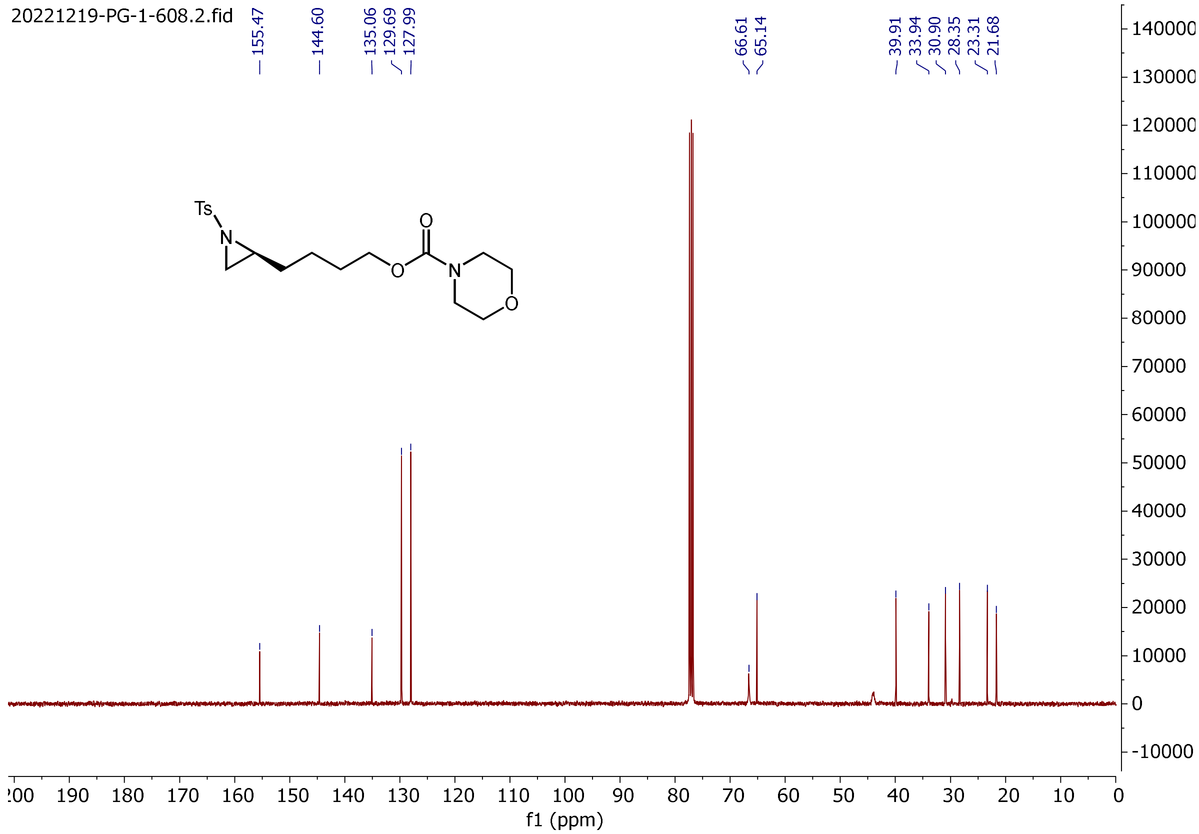

(±)-4-(1-tosylaziridin-2-yl)butyl morpholine-4-carboxylate (±-**22**):

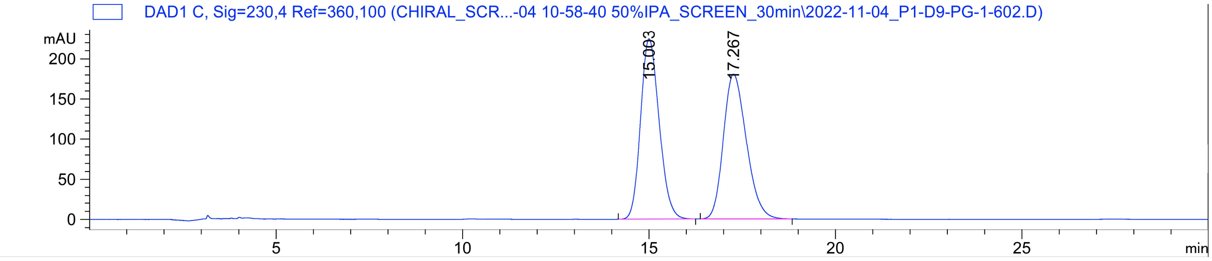

Signal 3: DAD1 C, Sig=230,4 Ref=360,100

Peak #	RetTime [min]	Type	Width [min]	Area [mAU*s]	Height [mAU]	Area %
1	15.003	BB	0.5036	7765.38818	224.21693	50.1402
2	17.267	BB	0.5204	7721.96191	180.19902	49.8598
Totals :				1.54874e4	404.41595	

(S)-4-(1-tosylaziridin-2-yl)butyl morpholine-4-carboxylate (**22**):

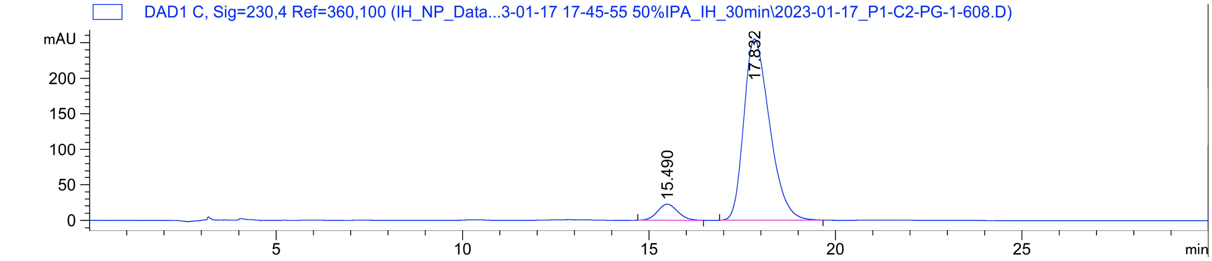

Signal 3: DAD1 C, Sig=230,4 Ref=360,100

Peak #	RetTime [min]	Type	Width [min]	Area [mAU*s]	Height [mAU]	Area %
1	15.490	BB	0.4397	822.09857	21.96504	6.3195
2	17.832	BB	0.5734	1.21868e4	254.63814	93.6805
Totals :				1.30089e4	276.60318	

(S)-1-(4-(1-tosylaziridin-2-yl)butyl)indoline-2,3-dione (**23**):

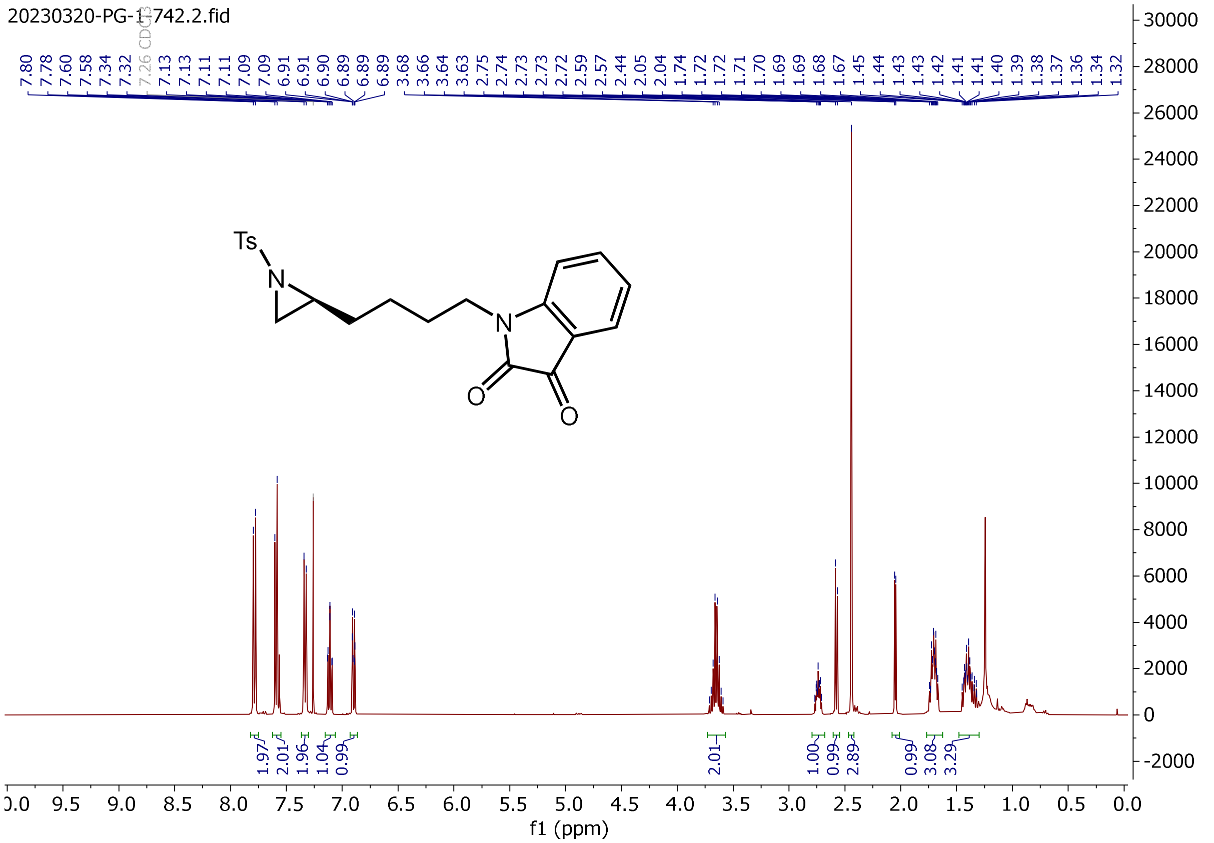

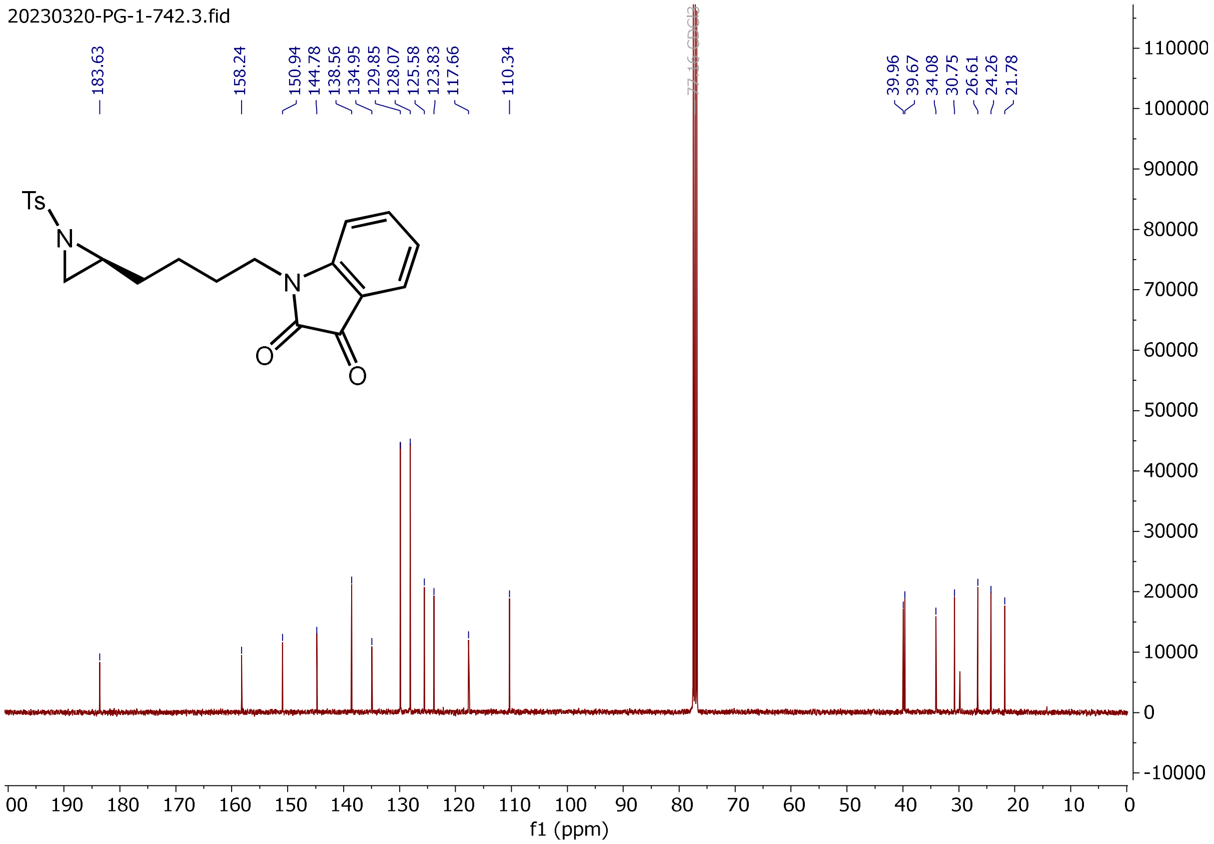

(±)-1-(4-(1-tosylaziridin-2-yl)butyl)indoline-2,3-dione (±-**23**):

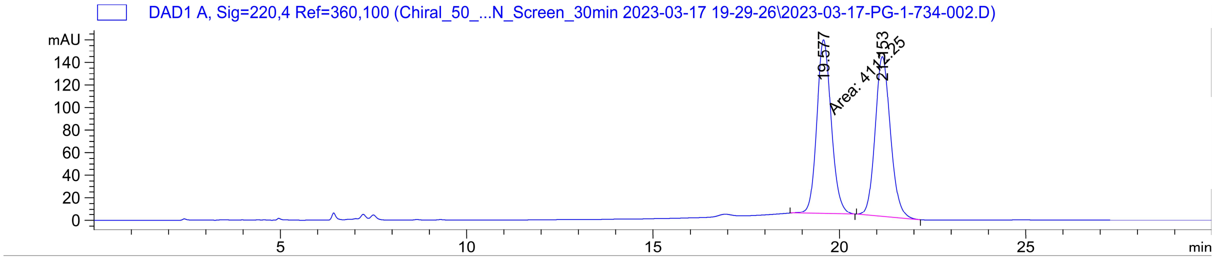

Signal 1: DAD1 A, Sig=220,4 Ref=360,100

Peak #	RetTime [min]	Type	Width [min]	Area [mAU*s]	Height [mAU]	Area %
1	19.577	MM T	0.4450	4112.25488	154.01982	50.2835
2	21.153	BB	0.4413	4065.88867	141.48030	49.7165
Totals :				8178.14355	295.50012	

(S)-1-(4-(1-tosylaziridin-2-yl)butyl)indoline-2,3-dione (**23**):

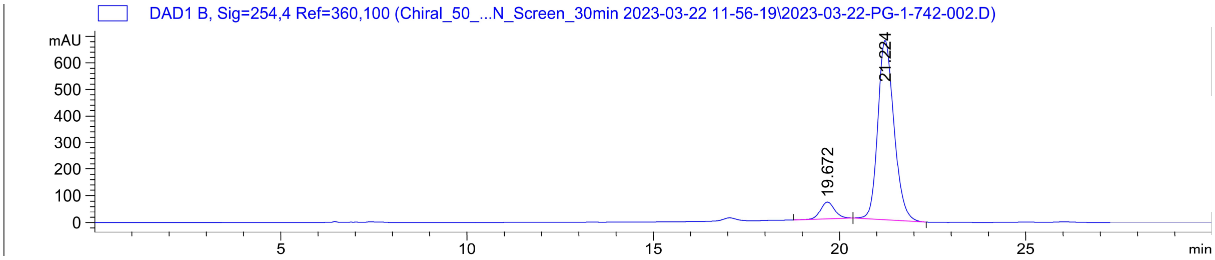

Signal 2: DAD1 B, Sig=254,4 Ref=360,100

Peak #	RetTime [min]	Type	Width [min]	Area [mAU*s]	Height [mAU]	Area %
1	19.672	BB	0.4025	1629.29834	62.43285	7.4078
2	21.224	BB	0.4644	2.03651e4	674.12158	92.5922
Totals :				2.19944e4	736.55443	

(S)-2-bromo-5-(4-(1-tosylaziridin-2-yl)butoxy)pyrazine (**24**):

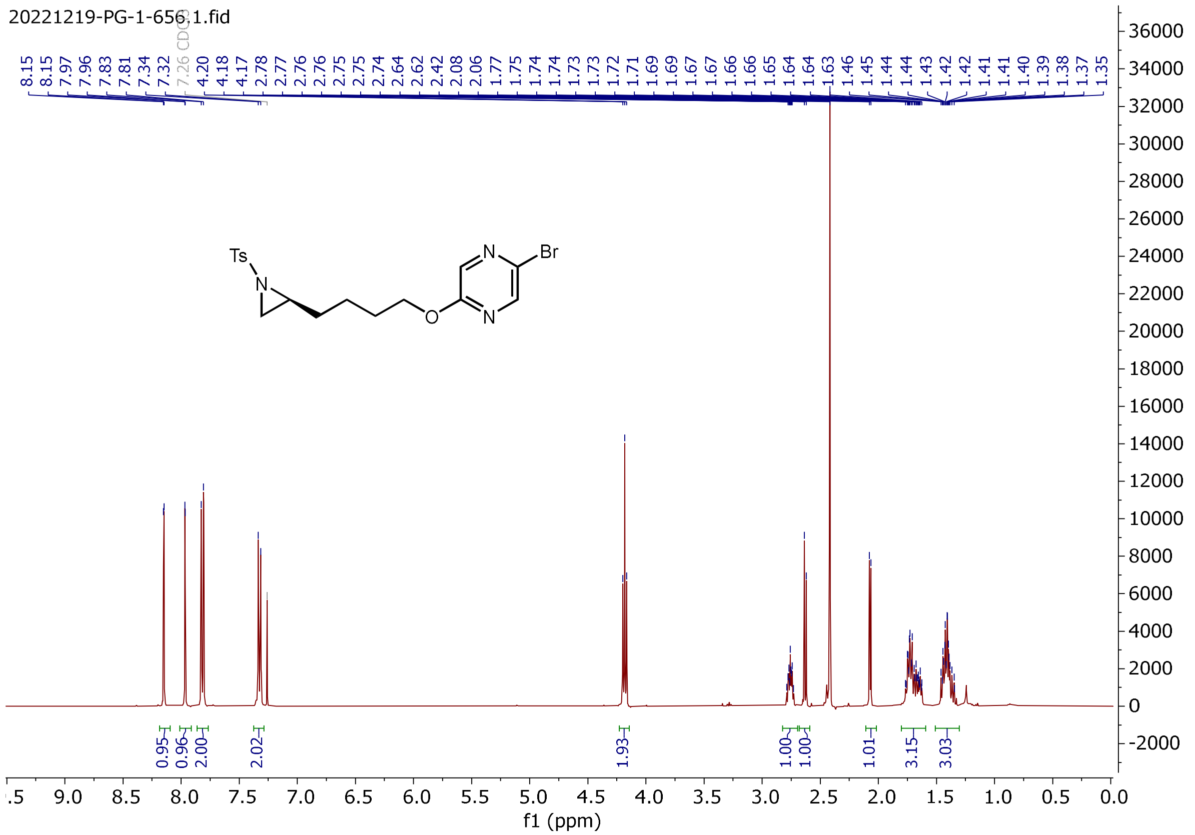

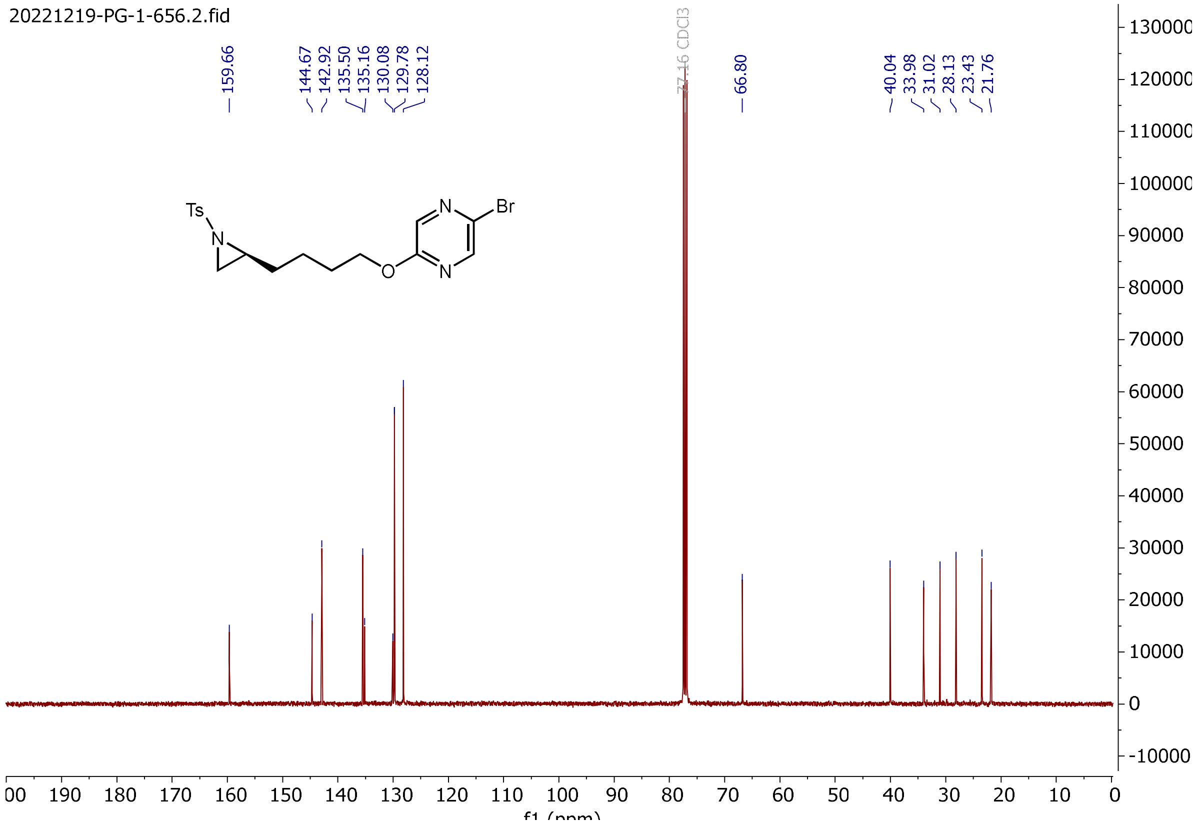

(±)-2-bromo-5-(4-(1-tosylaziridin-2-yl)butoxy)pyrazine (±-**24**):

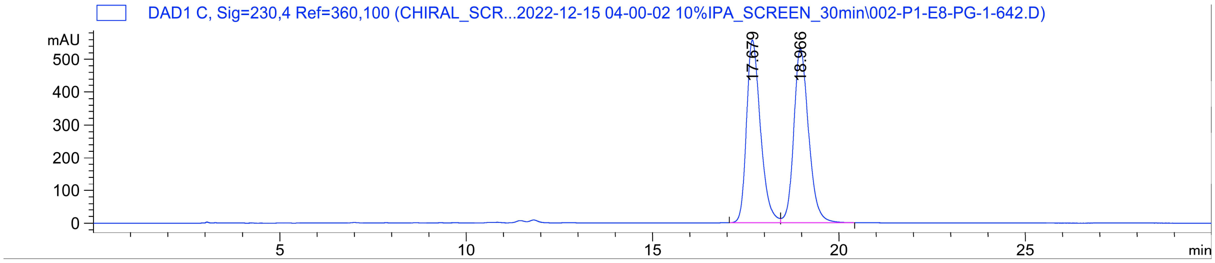

Signal 3: DAD1 C, Sig=230,4 Ref=360,100

Peak #	RetTime [min]	Type	Width [min]	Area [mAU*s]	Height [mAU]	Area %
1	17.679	BV	0.3918	1.44649e4	557.33893	49.6285
2	18.966	VB	0.4172	1.46814e4	529.94415	50.3715

Totals : 2.91463e4 1087.28308

(S)-2-bromo-5-(4-(1-tosylaziridin-2-yl)butoxy)pyrazine (**24**):

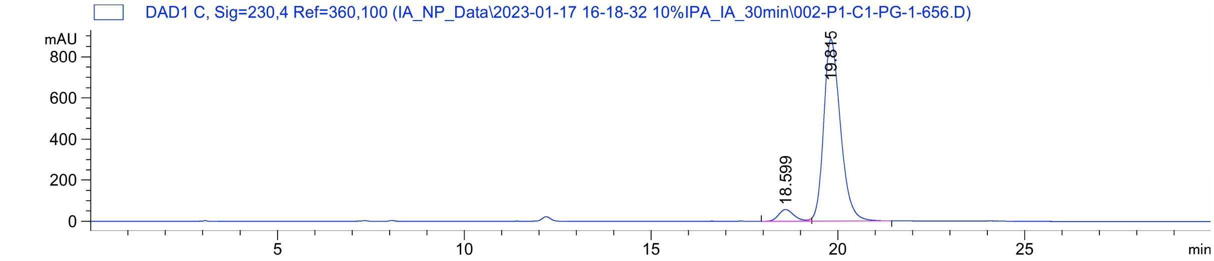

Signal 3: DAD1 C, Sig=230,4 Ref=360,100

Peak #	RetTime [min]	Type	Width [min]	Area [mAU*s]	Height [mAU]	Area %
1	18.599	BV E	0.3382	1573.57642	55.60388	5.4165
2	19.815	VB R	0.4695	2.74781e4	883.12793	94.5835

Totals : 2.90517e4 938.73181

(S)-2-bromo-3-(4-(1-tosylaziridin-2-yl)butoxy)pyridine (**25**):

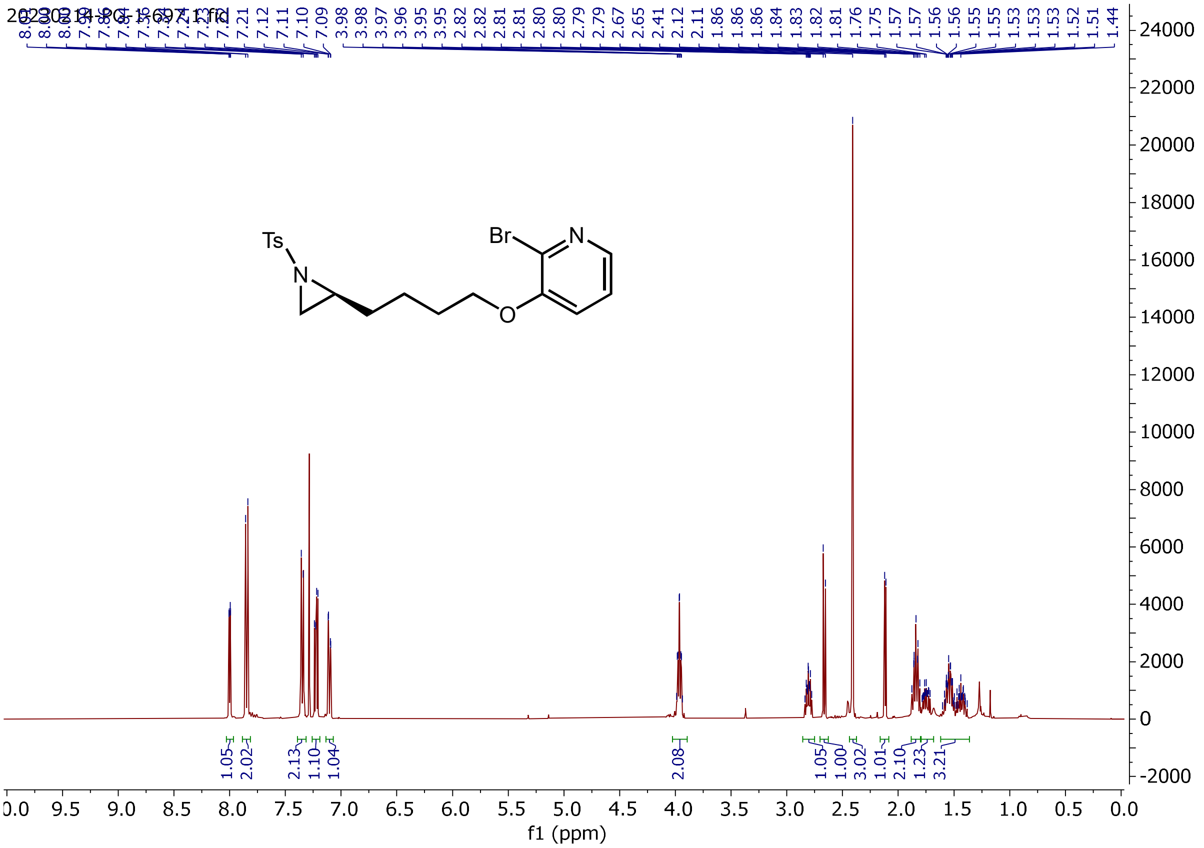

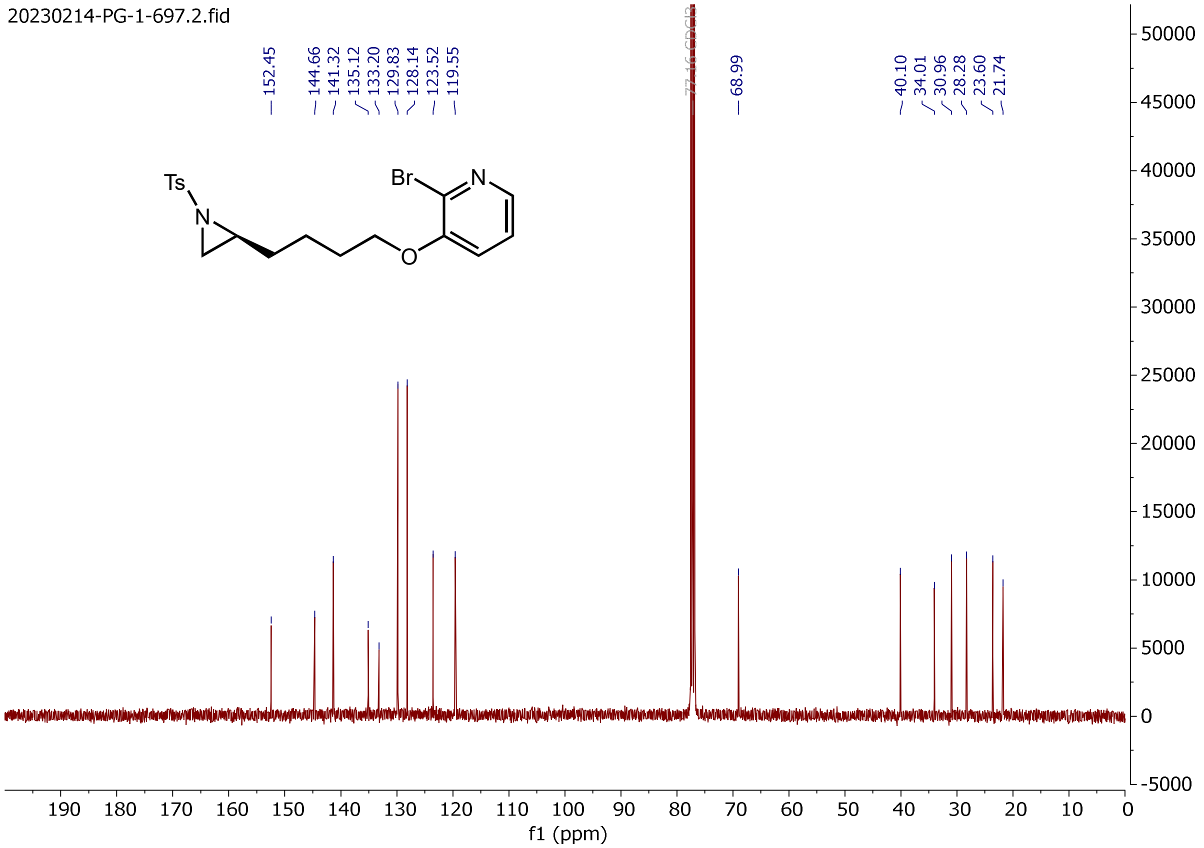

(±)-2-bromo-3-(4-(1-tosylaziridin-2-yl)butoxy)pyridine (±-**25**):

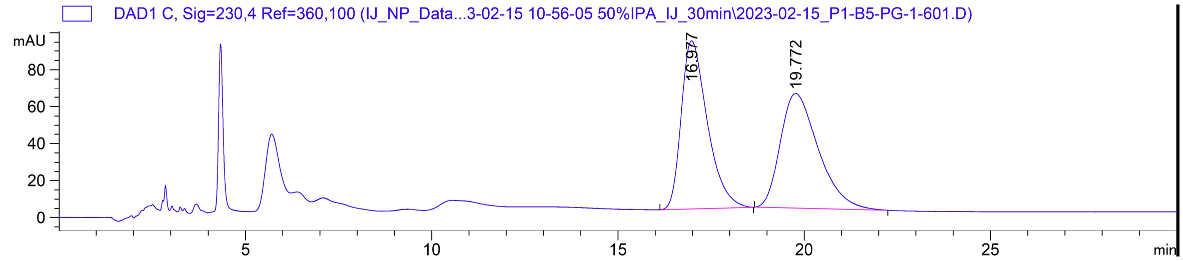

Signal 3: DAD1 C, Sig=230,4 Ref=360,100

Peak #	RetTime [min]	Type	Width [min]	Area [mAU*s]	Height [mAU]	Area %
1	16.977	BB	0.5678	4414.51611	90.95141	50.1847
2	19.772	BB	0.8302	4382.01660	61.83913	49.8153
Totals :				8796.53271	152.79054	

(S)-2-bromo-3-(4-(1-tosylaziridin-2-yl)butoxy)pyridine (**25**):

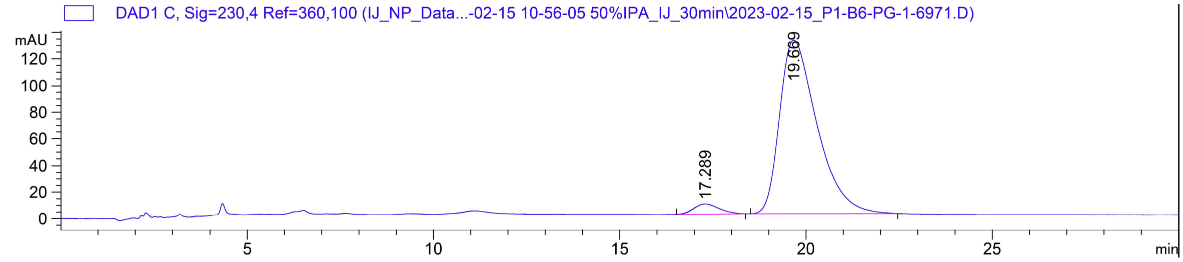

Signal 3: DAD1 C, Sig=230,4 Ref=360,100

Peak #	RetTime [min]	Type	Width [min]	Area [mAU*s]	Height [mAU]	Area %
1	17.289	BB	0.5509	361.12485	7.66805	3.8178
2	19.669	BB	0.8194	9097.79199	130.40765	96.1822
Totals :				9458.91684	138.07571	

(S)-2-(4-(((3aR,5R,6S,6aR)-5-((R)-2,2-dimethyl-1,3-dioxolan-4-yl)-2,2-dimethyltetrahydrofuro[2,3-d][1,3]dioxol-6-yl)oxy)butyl)-1-tosylaziridine (**26**):

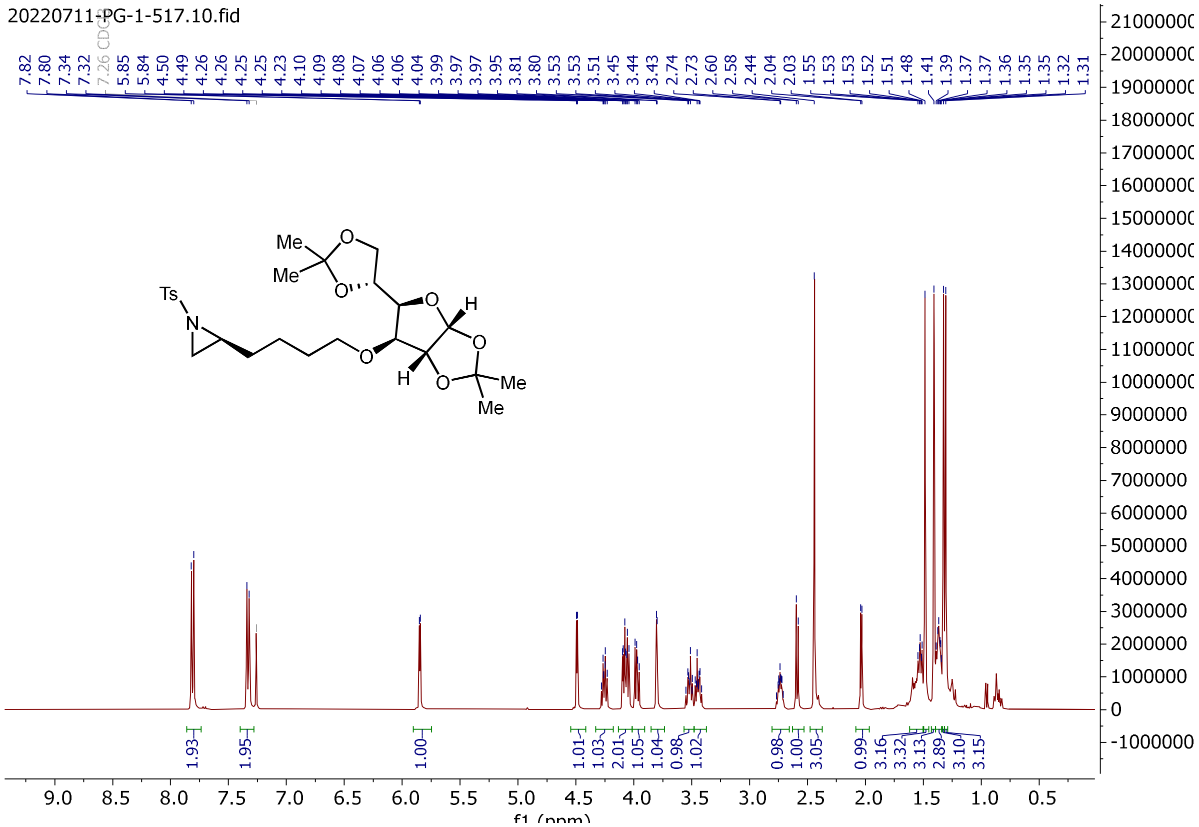

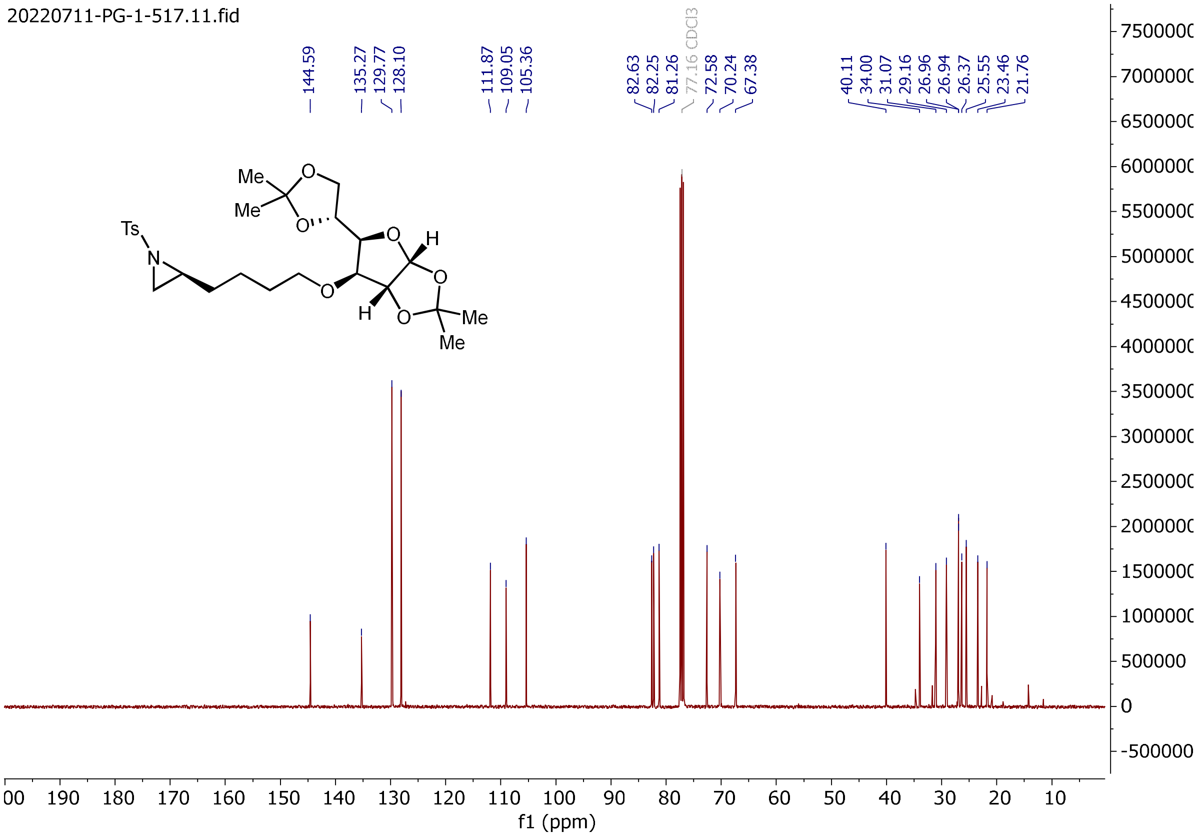

2-(4-(((3aR,5R,6S,6aR)-5-((R)-2,2-dimethyl-1,3-dioxolan-4-yl)-2,2-dimethyltetrahydrofuro[2,3-d][1,3]dioxol-6-yl)oxy)butyl)-1-tosylaziridine (**26**):

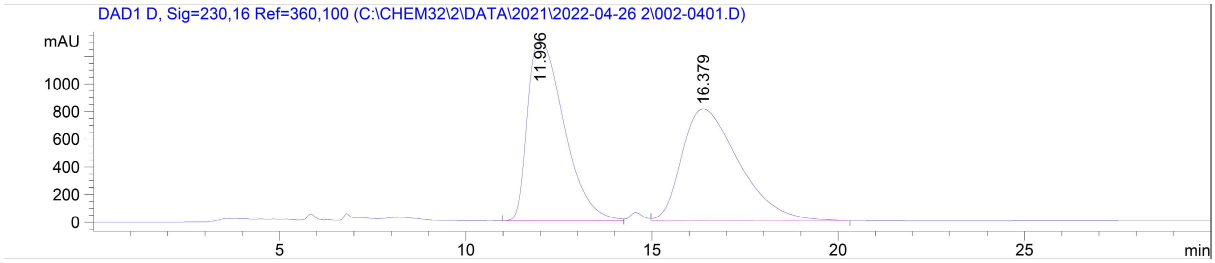

Signal 3: DAD1 D, Sig=230,16 Ref=360,100

Peak #	RetTime [min]	Type	Width [min]	Area [mAU*s]	Height [mAU]	Area %
1	11.996	VV	1.0645	8.88711e4	1301.70557	50.8187
2	16.379	VB	1.6105	8.60076e4	808.91321	49.1813
Totals :				1.74879e5	2110.61877	

(S)-2-(4-(((3aR,5R,6S,6aR)-5-((R)-2,2-dimethyl-1,3-dioxolan-4-yl)-2,2-dimethyltetrahydrofuro[2,3-d][1,3]dioxol-6-yl)oxy)butyl)-1-tosylaziridine (**26**):

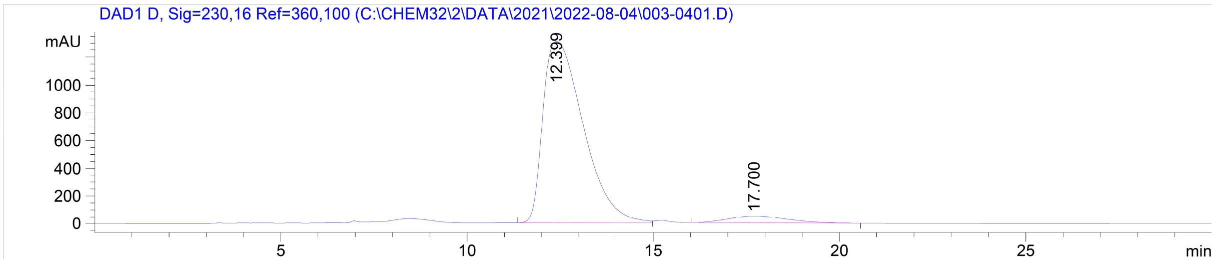

Signal 3: DAD1 D, Sig=230,16 Ref=360,100

Peak #	RetTime [min]	Type	Width [min]	Area [mAU*s]	Height [mAU]	Area %
1	12.399	BV	1.1575	9.86357e4	1308.34302	94.7218
2	17.700	BB	1.5455	5496.31592	50.75129	5.2782
Totals :				1.04132e5	1359.09430	

4-((S)-1-tosylaziridin-2-yl)butyl (tert-butoxycarbonyl)-L-phenylalaninate (**27**):

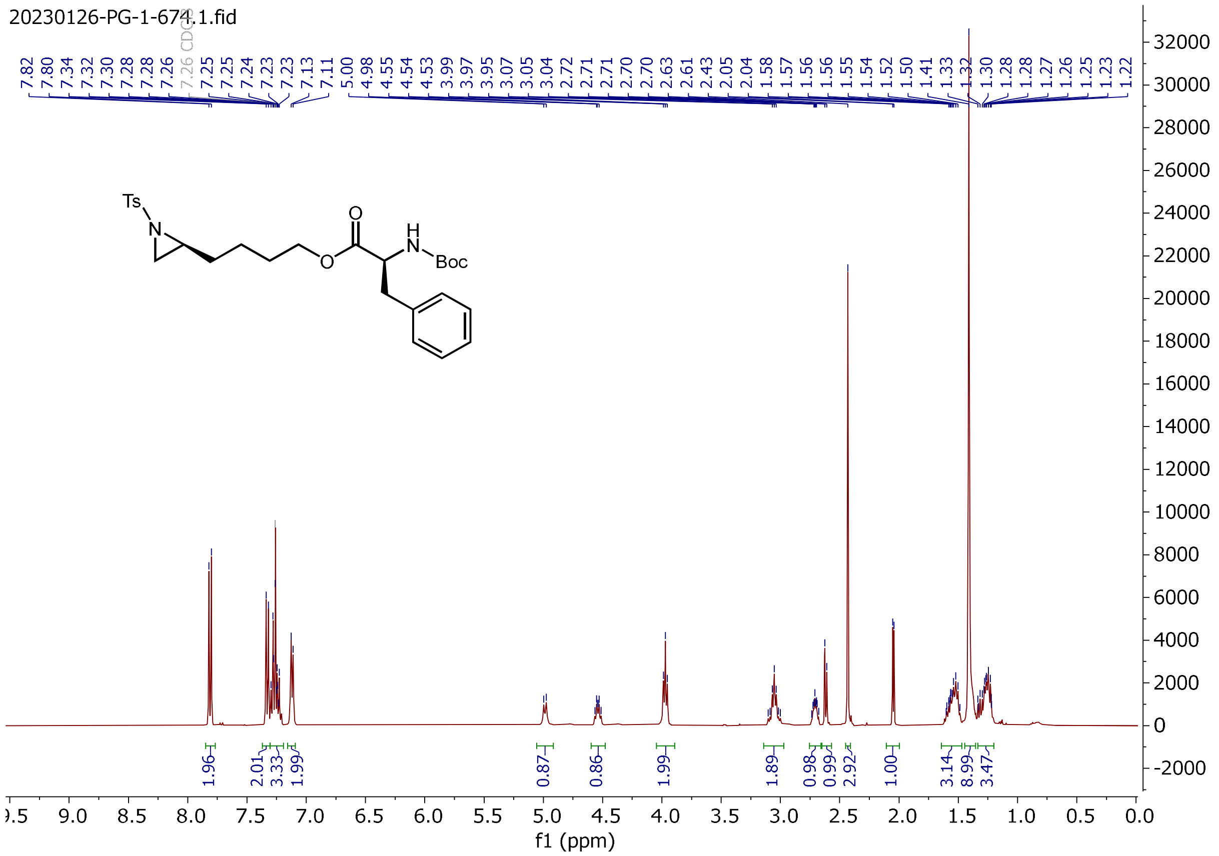

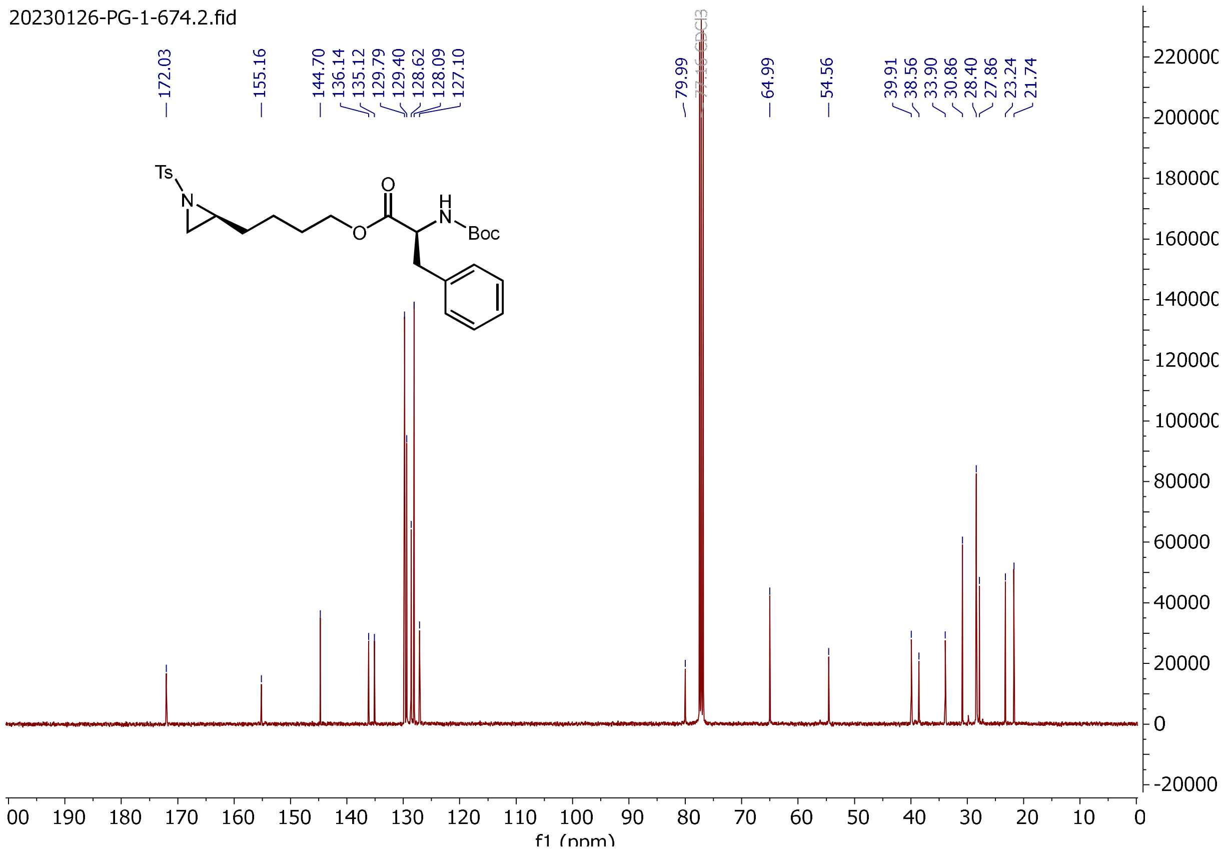

4-1-tosylaziridin-2-yl)butyl (tert-butoxycarbonyl)-L-phenylalaninate (**27**):

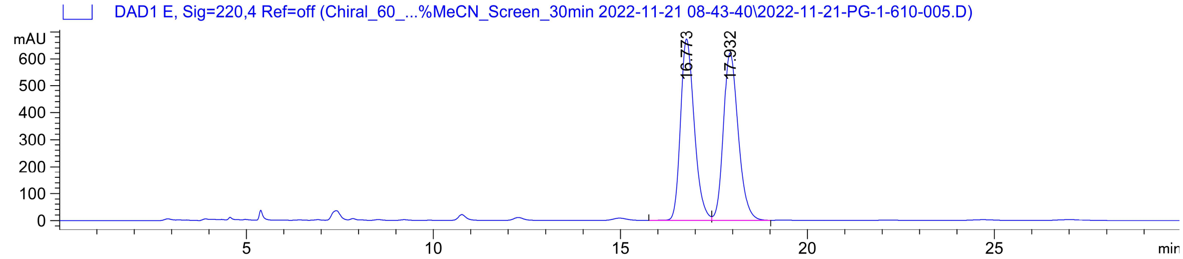

Signal 5: DAD1 E, Sig=220,4 Ref=off

Peak #	RetTime [min]	Type	Width [min]	Area [mAU*s]	Height [mAU]	Area %
1	16.773	BV	0.3897	1.70401e4	672.19922	49.9161
2	17.932	VB	0.4231	1.70974e4	621.28369	50.0839
Totals :				3.41375e4	1293.48291	

4-((S)-1-tosylaziridin-2-yl)butyl (tert-butoxycarbonyl)-L-phenylalaninate (**27**):

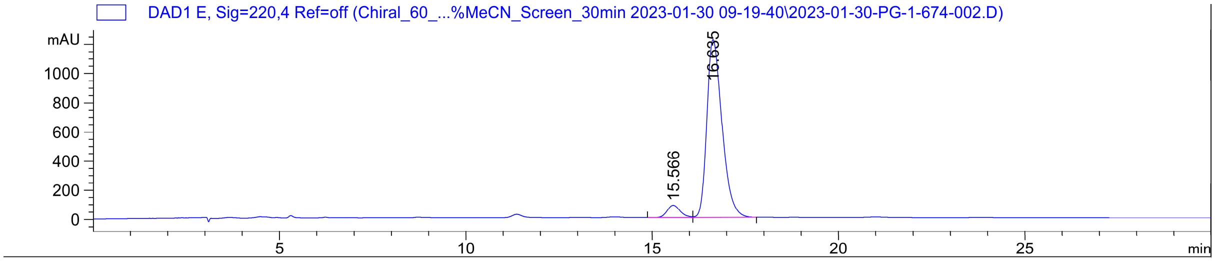

Signal 5: DAD1 E, Sig=220,4 Ref=off

Peak #	RetTime [min]	Type	Width [min]	Area [mAU*s]	Height [mAU]	Area %
1	15.566	BV	0.3742	2028.79102	83.28348	5.5634
2	16.635	VB	0.4357	3.44380e4	1218.73645	94.4366
Totals :				3.64668e4	1302.01993	

(*S*)-2-((*S*)-5-(benzyloxy)-3-methylpentyl)-1-tosylaziridine (**(*S,S*)-28**):

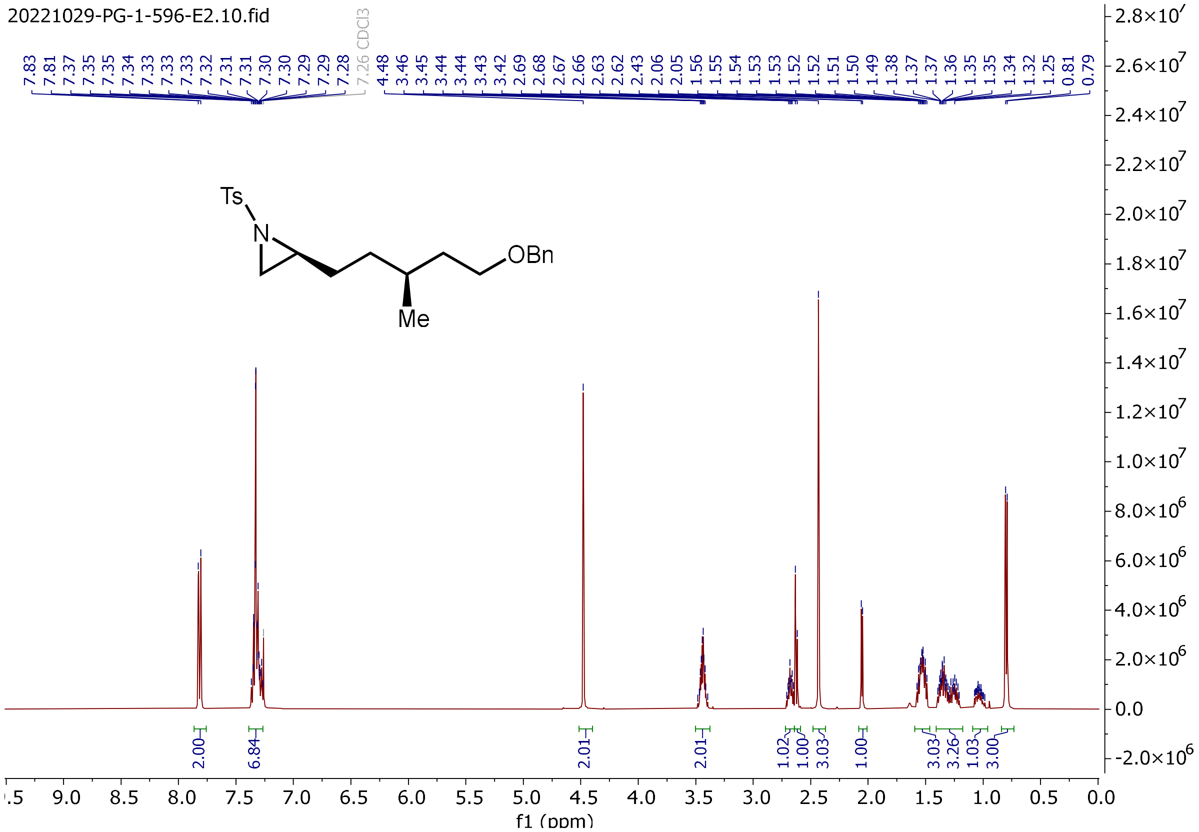

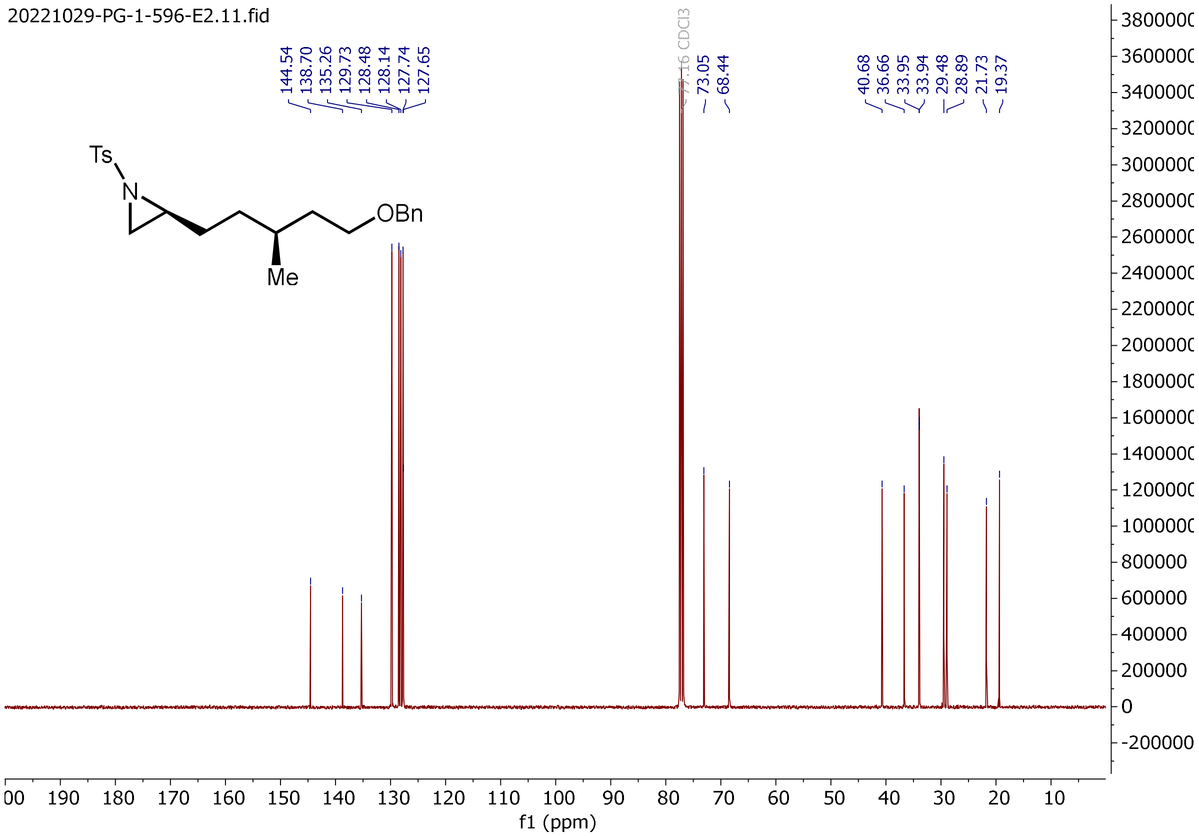

2-((S)-5-(benzyloxy)-3-methylpentyl)-1-tosylaziridine (**28**)

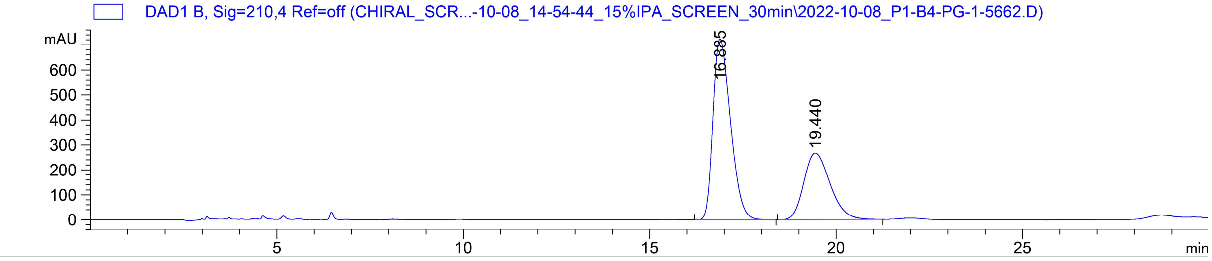

Signal 2: DAD1 B, Sig=210,4 Ref=off

Peak #	RetTime [min]	Type	Width [min]	Area [mAU*s]	Height [mAU]	Area %
1	16.885	BB	0.5058	2.36370e4	724.16583	64.3584
2	19.440	BB	0.6979	1.30901e4	266.55930	35.6416

Totals : 3.67271e4 990.72513

S)-2-((*S*)-5-(benzyloxy)-3-methylpentyl)-1-tosylaziridine (***(S,S)*-28**):

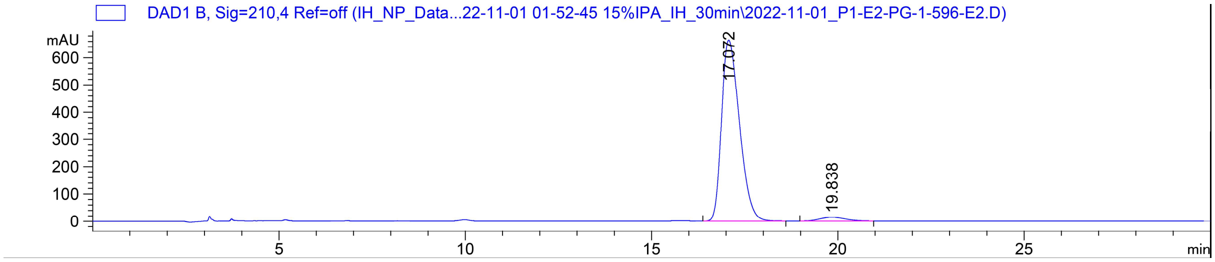

Signal 2: DAD1 B, Sig=210,4 Ref=off

Peak #	RetTime [min]	Type	Width [min]	Area [mAU*s]	Height [mAU]	Area %
1	17.072	BB	0.4746	2.21371e4	664.59760	96.9773
2	19.838	BB	0.5469	689.98444	14.75927	3.0227

Totals : 2.28271e4 679.35686

(*R*)-2-((*S*)-5-(benzyloxy)-3-methylpentyl)-1-tosylaziridine (***(R,S)*-28**):

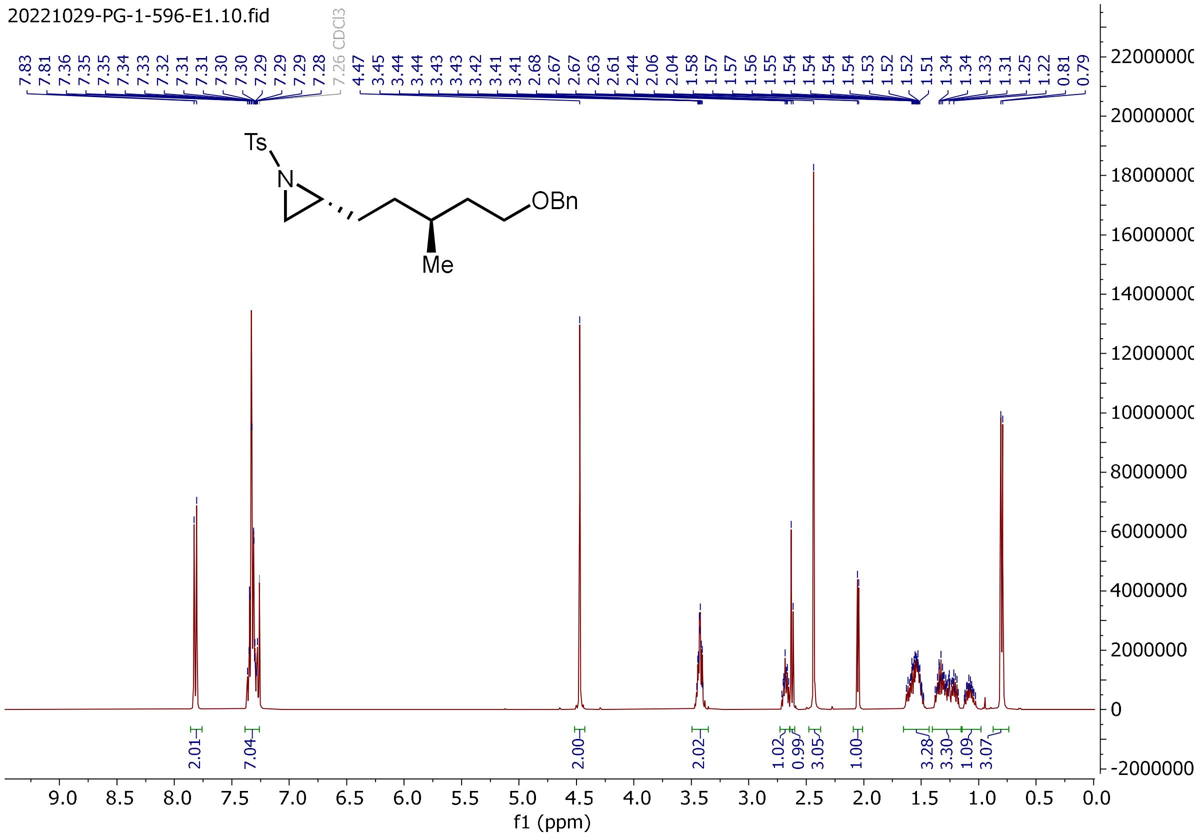

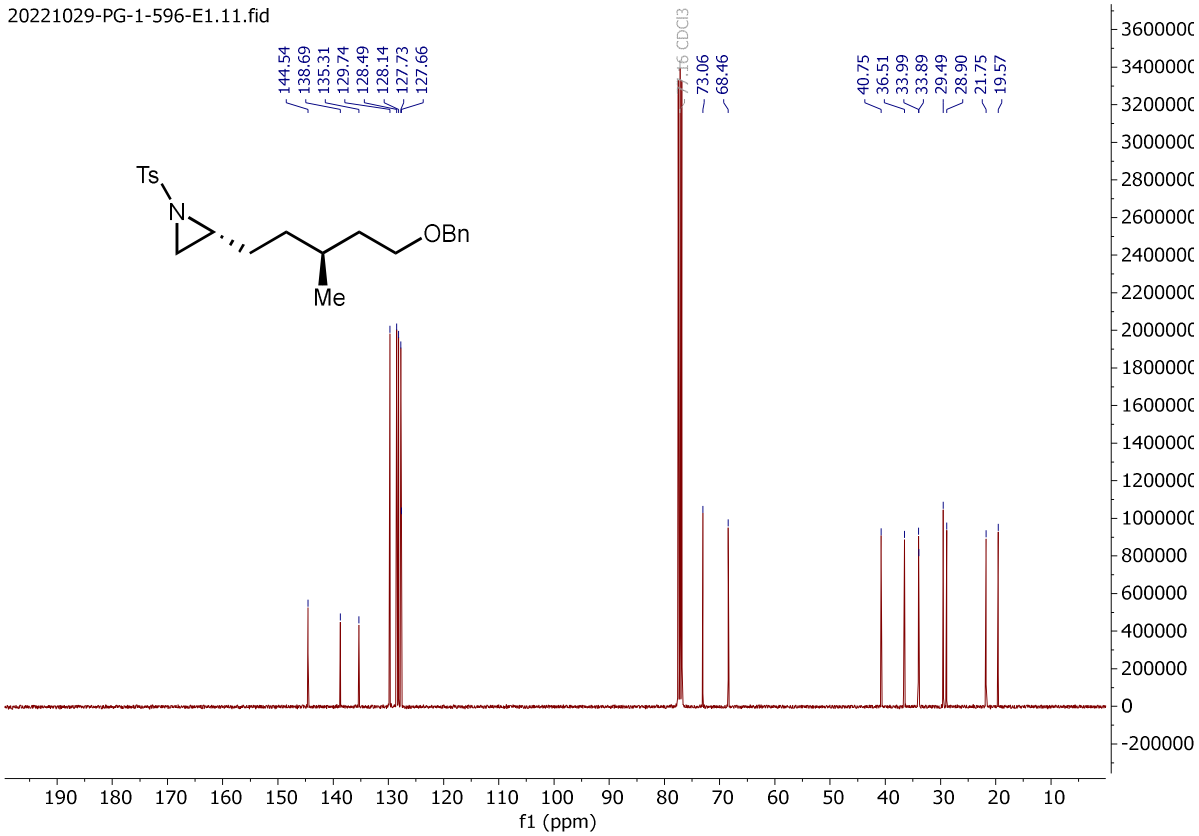

2-((S)-5-(benzyloxy)-3-methylpentyl)-1-tosylaziridine (**28**)

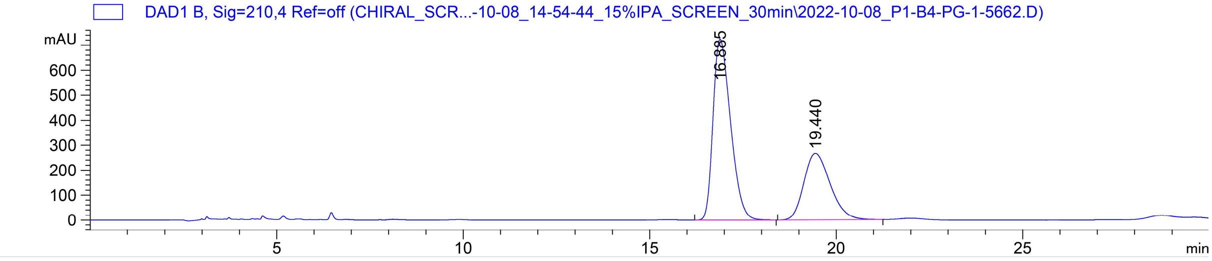

Signal 2: DAD1 B, Sig=210,4 Ref=off

Peak #	RetTime [min]	Type	Width [min]	Area [mAU*s]	Height [mAU]	Area %
1	16.885	BB	0.5058	2.36370e4	724.16583	64.3584
2	19.440	BB	0.6979	1.30901e4	266.55930	35.6416

Totals : 3.67271e4 990.72513

(*R*)-2-((*S*)-5-(benzyloxy)-3-methylpentyl)-1-tosylaziridine (***(R,S)*-28**):

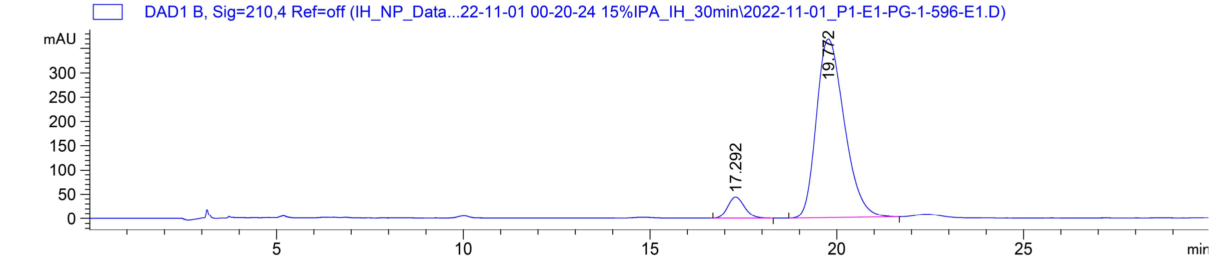

Signal 2: DAD1 B, Sig=210,4 Ref=off

Peak #	RetTime [min]	Type	Width [min]	Area [mAU*s]	Height [mAU]	Area %
1	17.292	BB	0.3666	1346.15967	43.55563	6.5820
2	19.772	BB	0.6237	1.91061e4	368.32233	93.4180

Totals : 2.04522e4 411.87796

(*S*)-2-((*S*)-4-(benzyloxy)-2-methylbutyl)-1-tosylaziridine (***(S,S)*-29**):

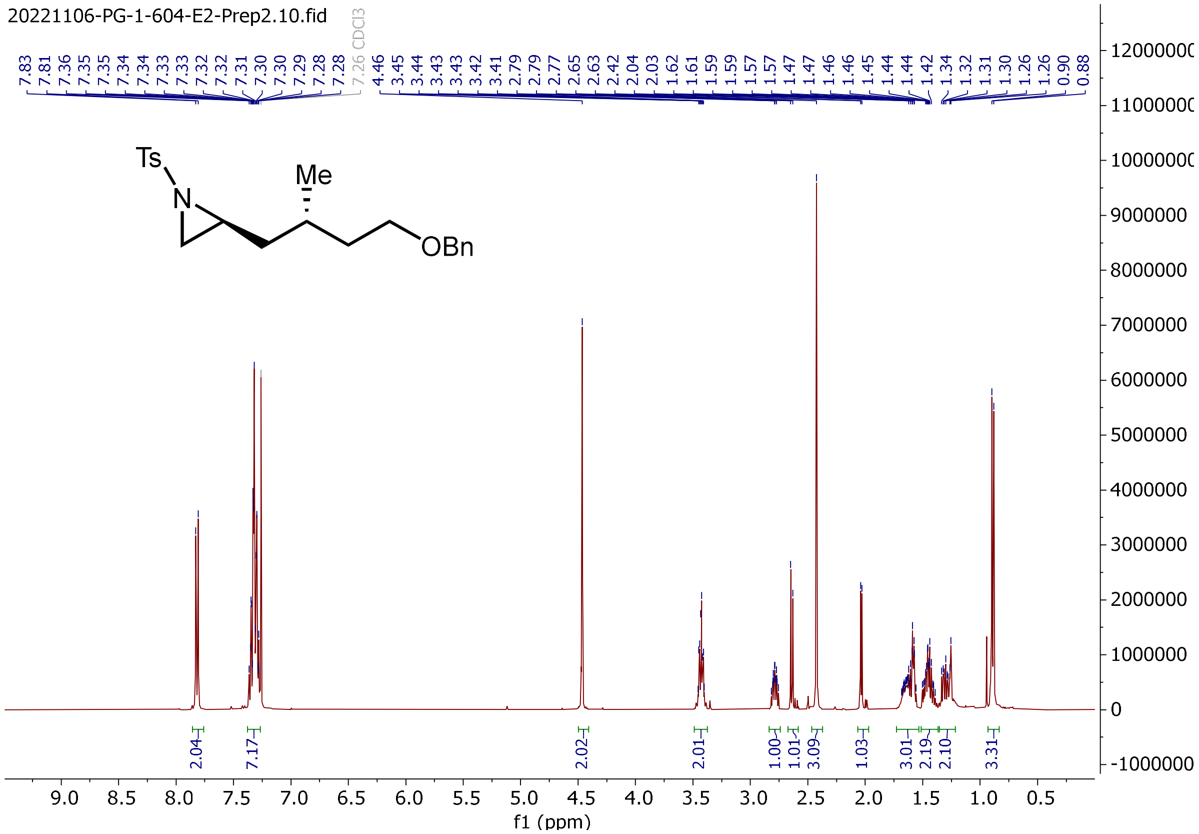

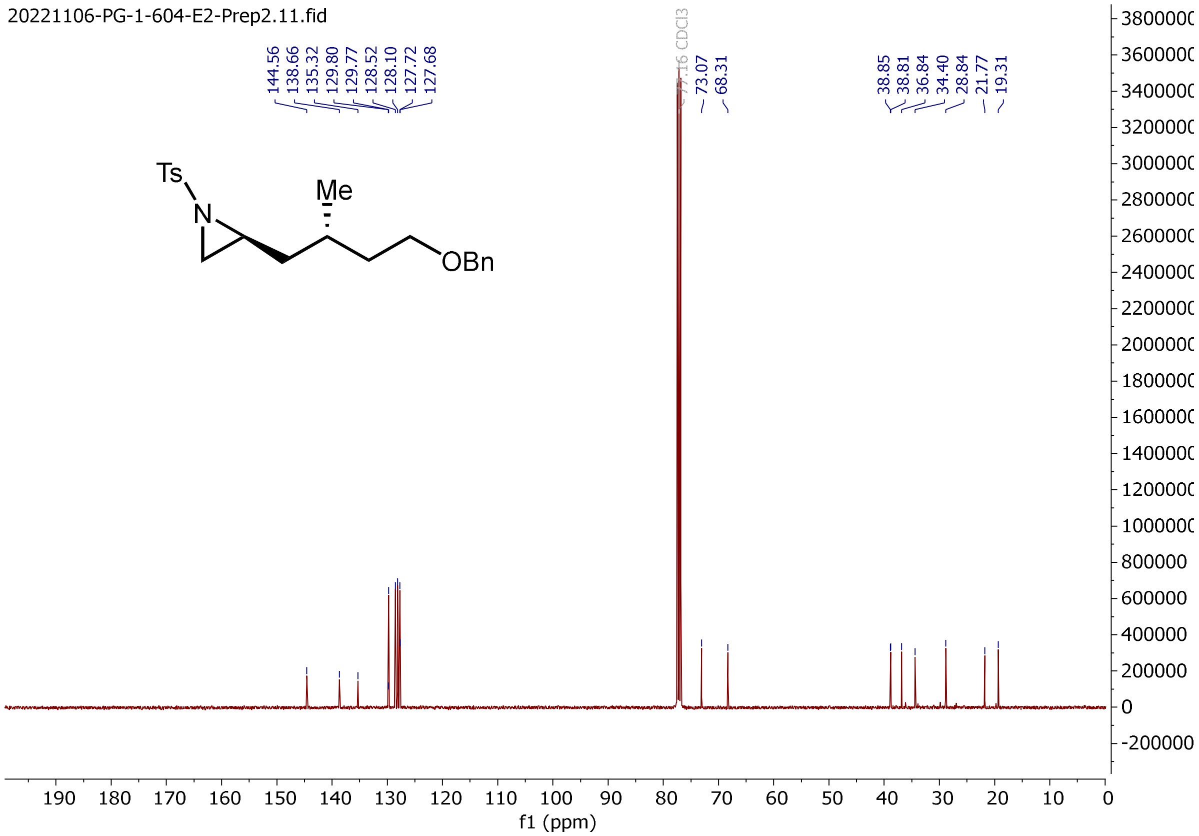

2-((S)-4-(benzyloxy)-2-methylbutyl)-1-tosylaziridine (**29**):

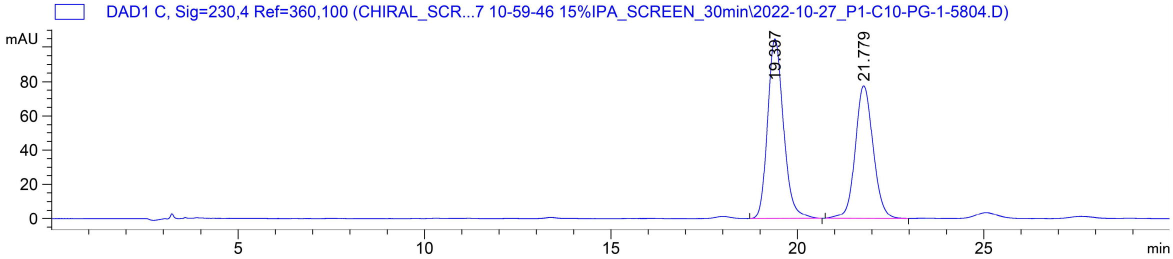

Signal 3: DAD1 C, Sig=230,4 Ref=360,100

Peak #	RetTime [min]	Type	Width [min]	Area [mAU*s]	Height [mAU]	Area %
1	19.397	BB	0.4214	3102.65259	104.80980	54.5842
2	21.779	BB	0.4107	2581.50830	77.57654	45.4158

Totals : 5684.16089 182.38634

(*S*)-2-((*S*)-4-(benzyloxy)-2-methylbutyl)-1-tosylaziridine (**(*S*,*S*)-29**):

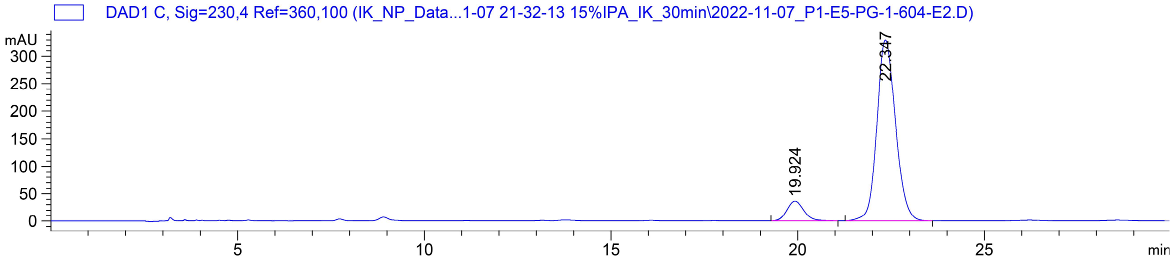

Signal 3: DAD1 C, Sig=230,4 Ref=360,100

Peak #	RetTime [min]	Type	Width [min]	Area [mAU*s]	Height [mAU]	Area %
1	19.924	BB	0.3720	1128.92102	36.03534	8.9920
2	22.347	BB	0.5147	1.14257e4	329.39688	91.0080

Totals : 1.25547e4 365.43222

(*R*)-2-((*S*)-4-(benzyloxy)-2-methylbutyl)-1-tosylaziridine ((***R,S*)-29**):

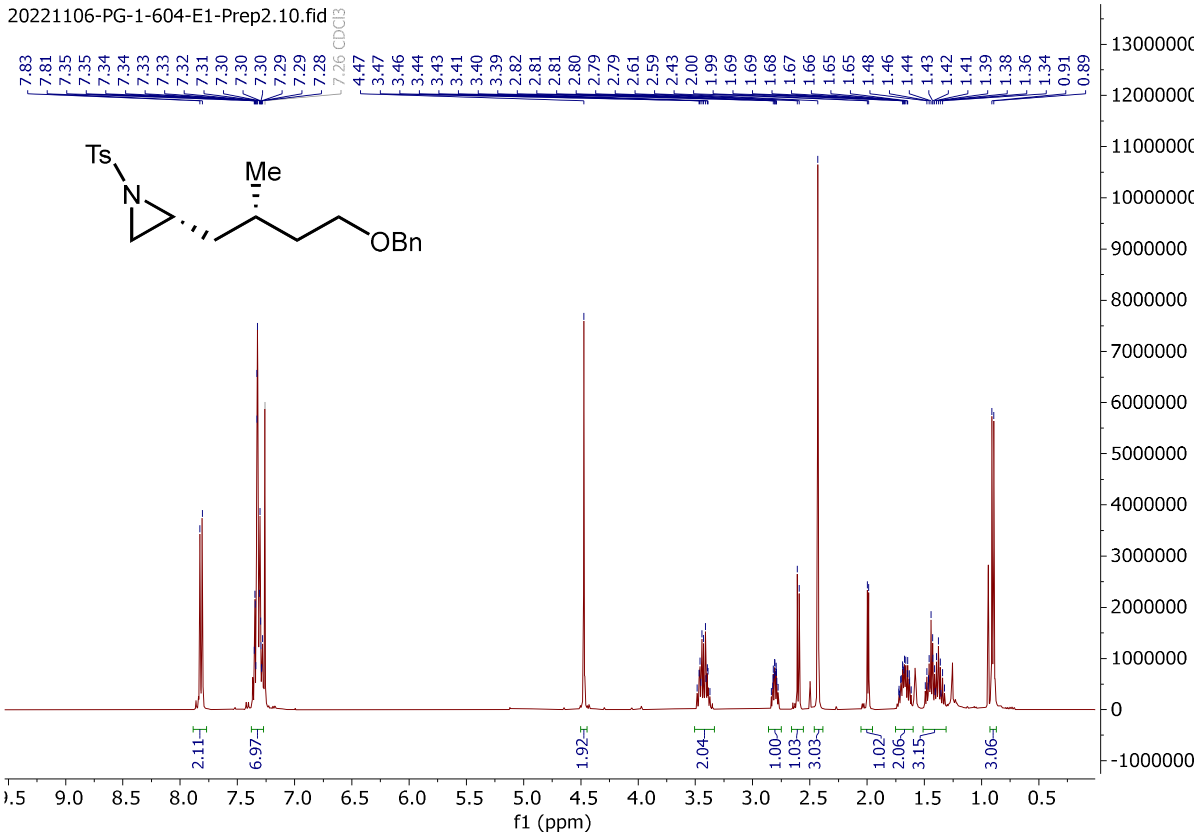

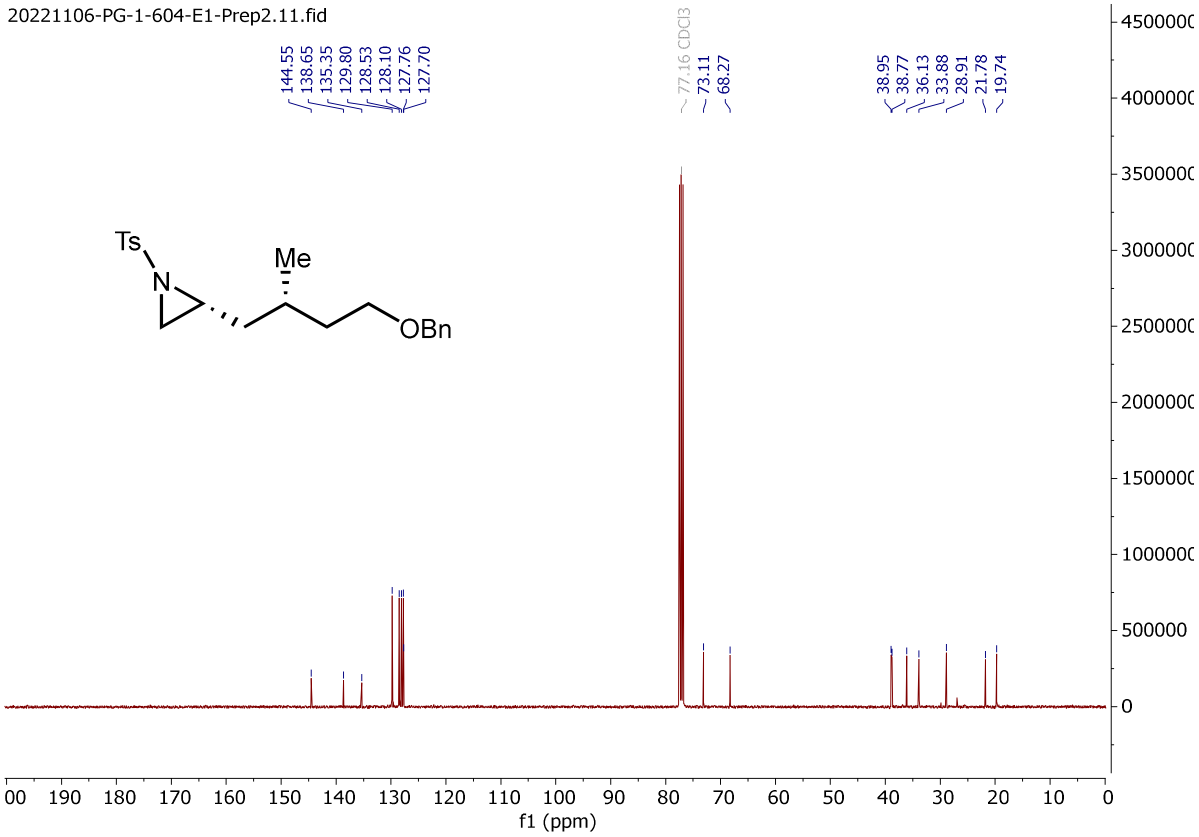

2-((S)-4-(benzyloxy)-2-methylbutyl)-1-tosylaziridine (**29**):

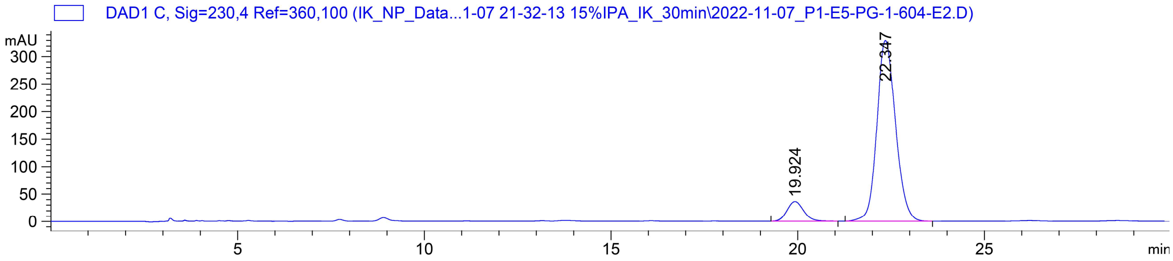

Signal 3: DAD1 C, Sig=230,4 Ref=360,100

Peak #	RetTime [min]	Type	Width [min]	Area [mAU*s]	Height [mAU]	Area %
1	19.397	BB	0.4214	3102.65259	104.80980	54.5842
2	21.779	BB	0.4107	2581.50830	77.57654	45.4158
Totals :				5684.16089	182.38634	

(*R*)-2-((*S*)-4-(benzyloxy)-2-methylbutyl)-1-tosylaziridine (**(*R*,*S*)-29**):

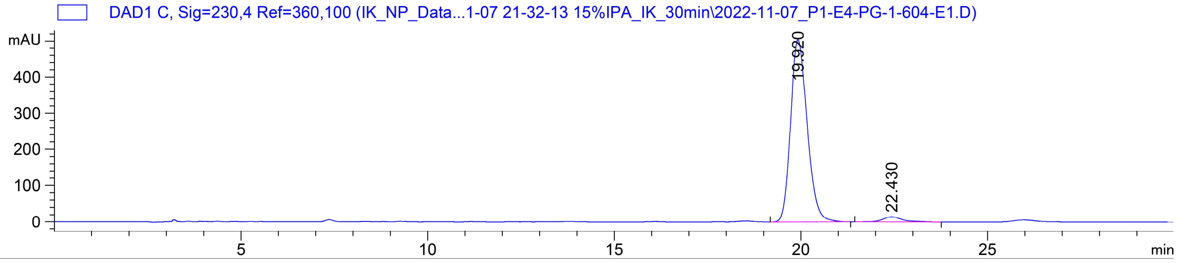

Signal 3: DAD1 C, Sig=230,4 Ref=360,100

Peak #	RetTime [min]	Type	Width [min]	Area [mAU*s]	Height [mAU]	Area %
1	19.920	BB	0.4690	1.55667e4	504.50003	96.5241
2	22.430	BB	0.4532	560.57104	14.57487	3.4759
Totals :				1.61273e4	519.07491	

(S)-2-(cyclohexylmethyl)-1-tosylaziridine (**30**):

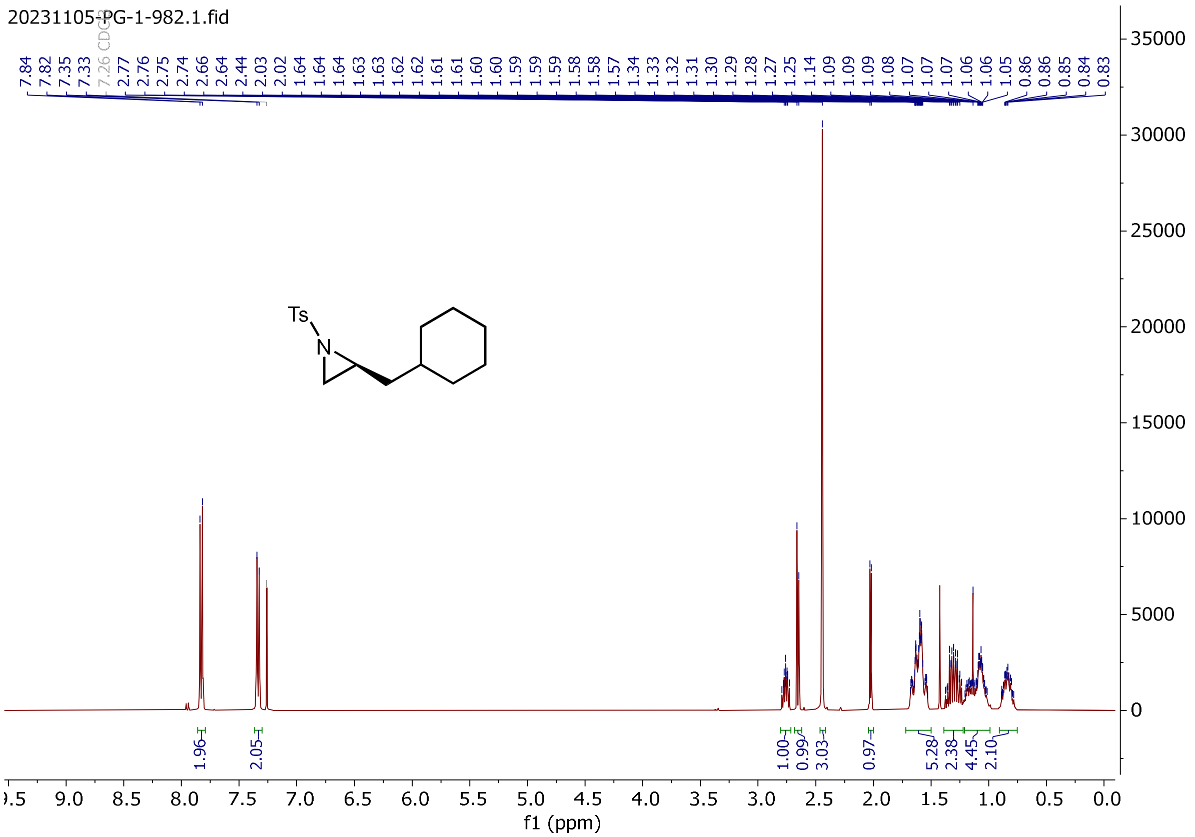

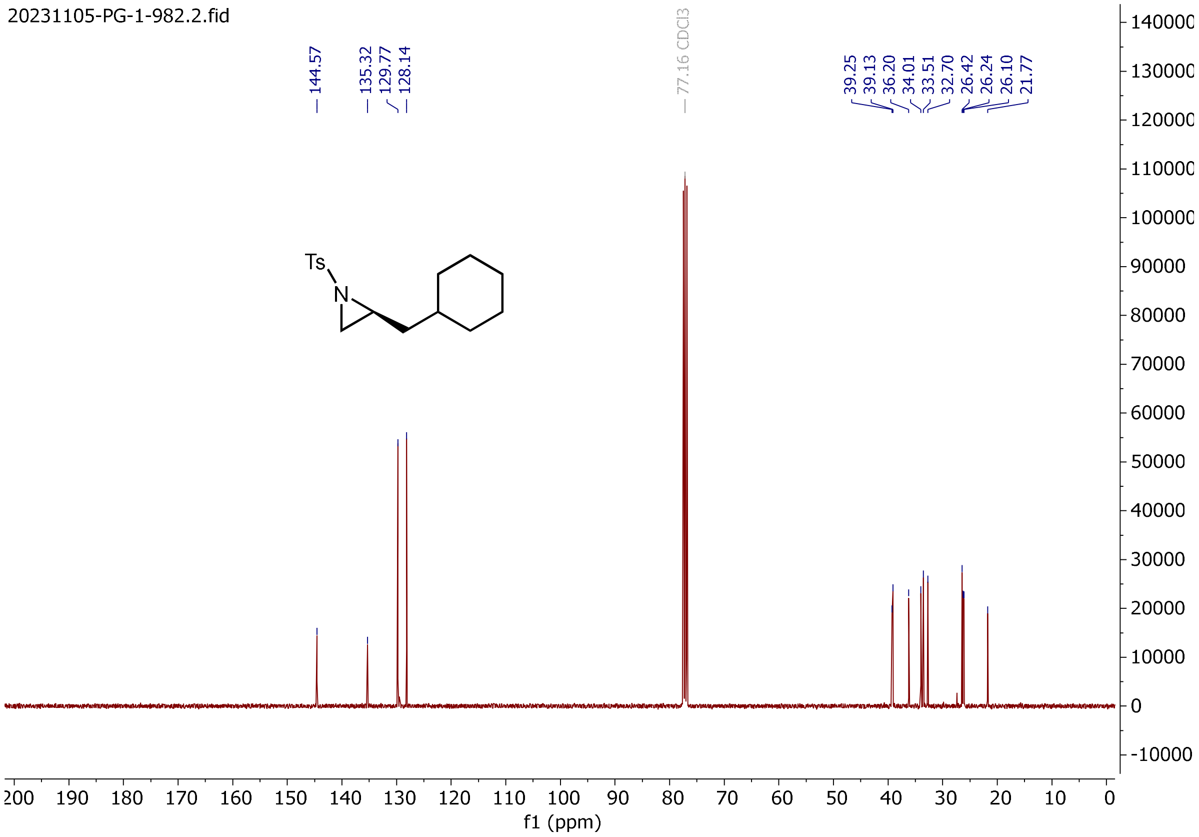

(±)-2-(cyclohexylmethyl)-1-tosylaziridine (±-**30**):

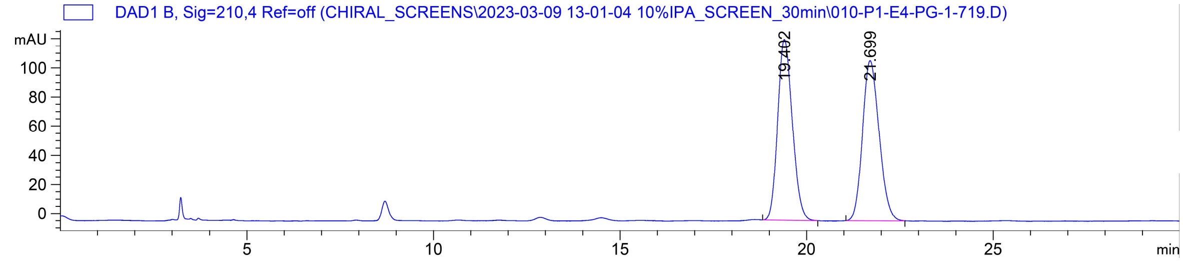

Signal 2: DAD1 B, Sig=210,4 Ref=off

Peak #	RetTime [min]	Type	Width [min]	Area [mAU*s]	Height [mAU]	Area %
1	19.402	BV R	0.3244	3395.34155	124.20193	49.8847
2	21.699	BB	0.3713	3411.03735	109.99075	50.1153
Totals :				6806.37891	234.19268	

(S)-2-(cyclohexylmethyl)-1-tosylaziridine (**30**):

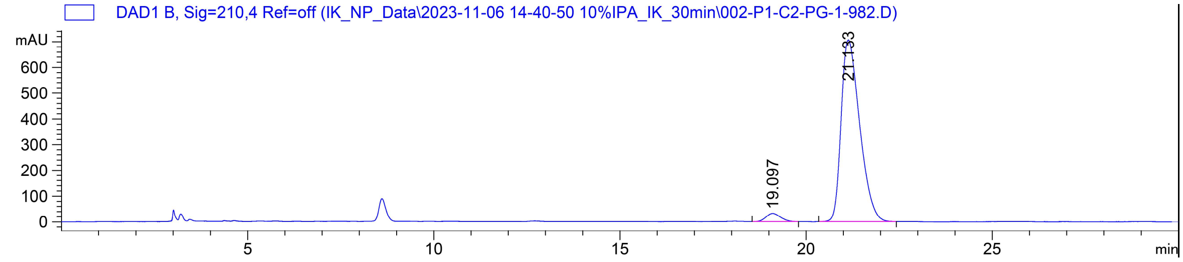

Signal 2: DAD1 B, Sig=210,4 Ref=off

Peak #	RetTime [min]	Type	Width [min]	Area [mAU*s]	Height [mAU]	Area %
1	19.097	BV R	0.3244	904.96912	32.84894	3.5732
2	21.133	VB R	0.4065	2.44217e4	706.94379	96.4268
Totals :				2.53267e4	739.79273	

(*S*)-2-cyclohexyl-1-tosylaziridine (**31**):

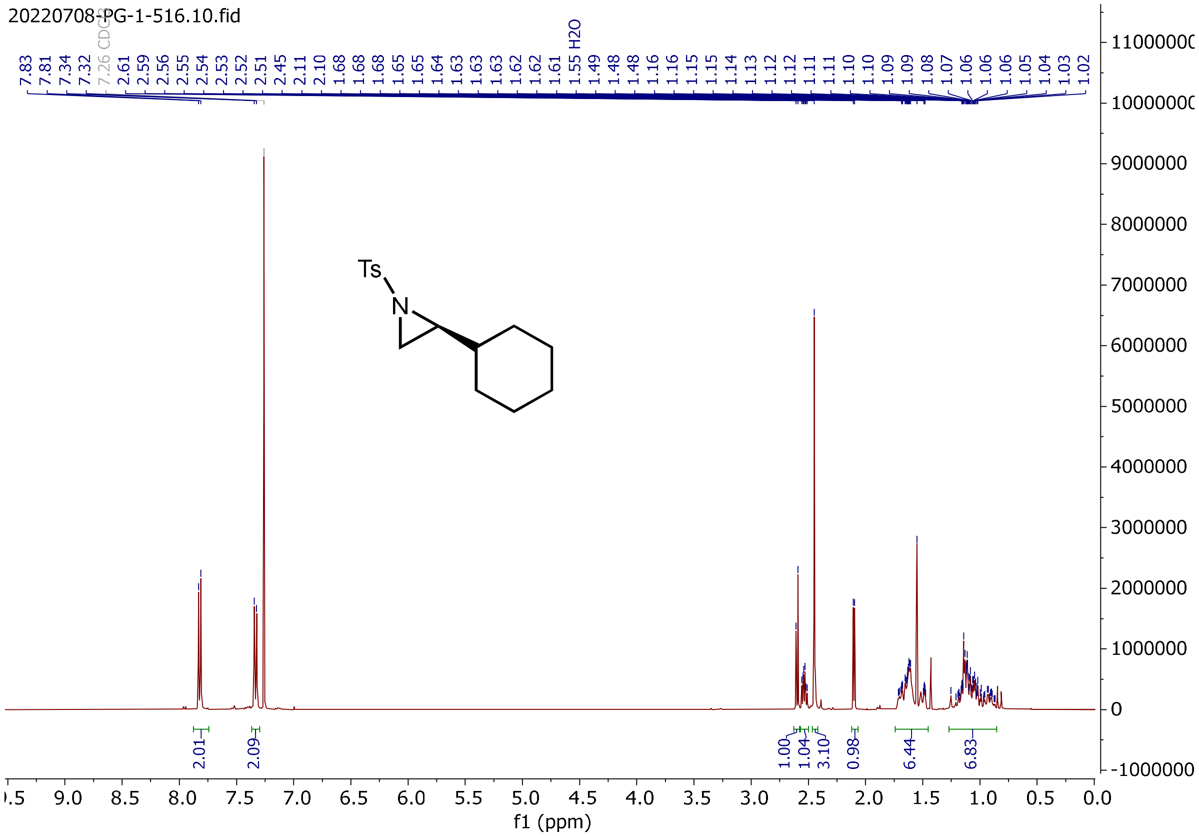

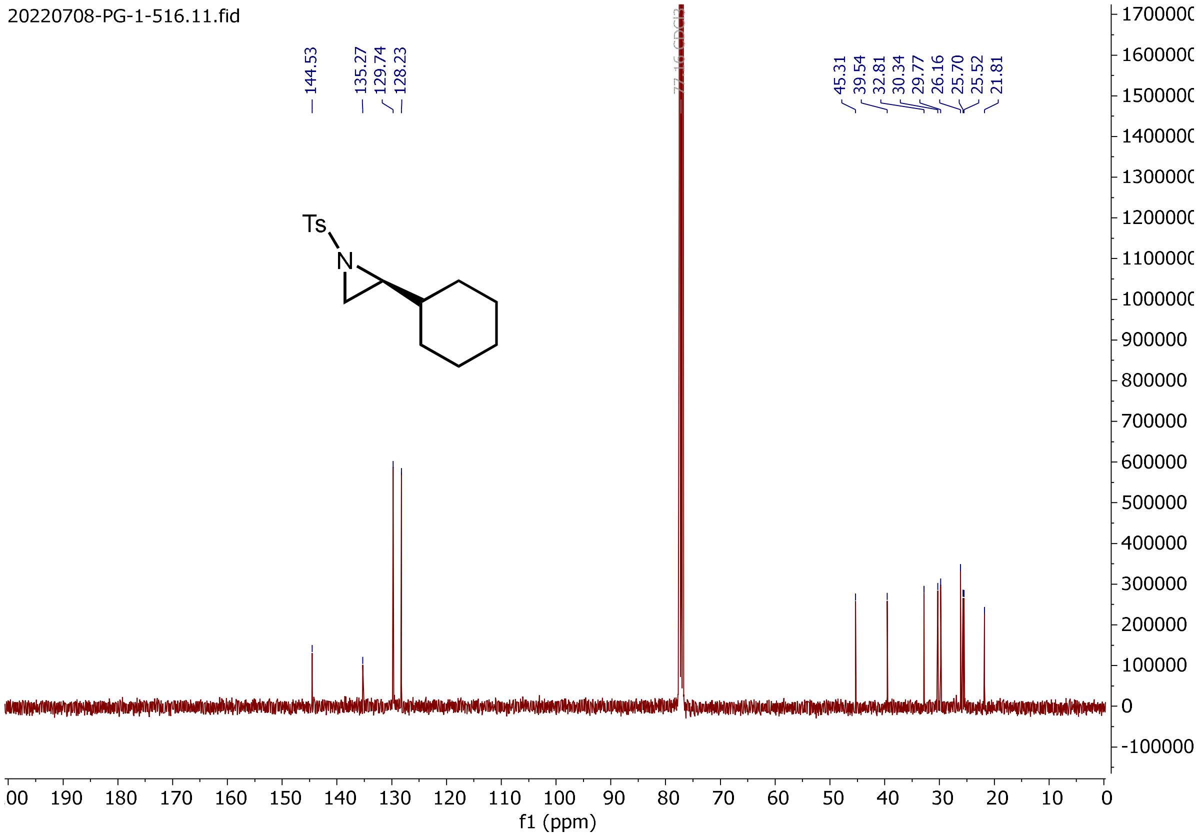

(±)-2-cyclohexyl-1-tosylaziridine (±-**31**):

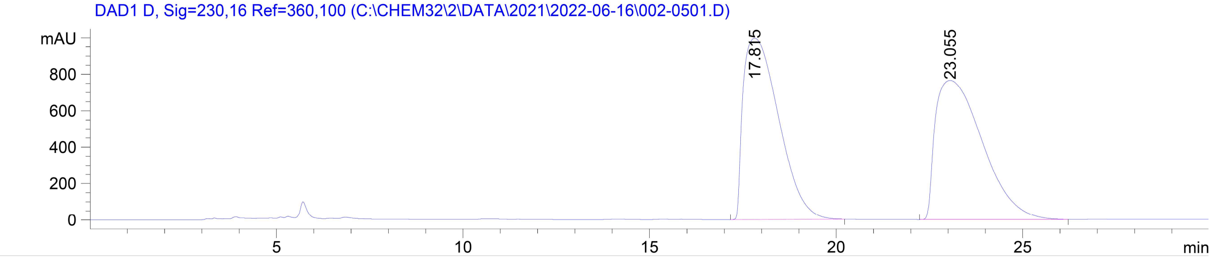

Signal 3: DAD1 D, Sig=230,16 Ref=360,100

Peak #	RetTime [min]	Type	Width [min]	Area [mAU*s]	Height [mAU]	Area %
1	17.815	BB	1.0732	6.60348e4	996.37714	49.4721
2	23.055	BB	1.4127	6.74440e4	764.86414	50.5279

Totals : 1.33479e5 1761.24127

(*S*)-2-cyclohexyl-1-tosylaziridine (**31**):

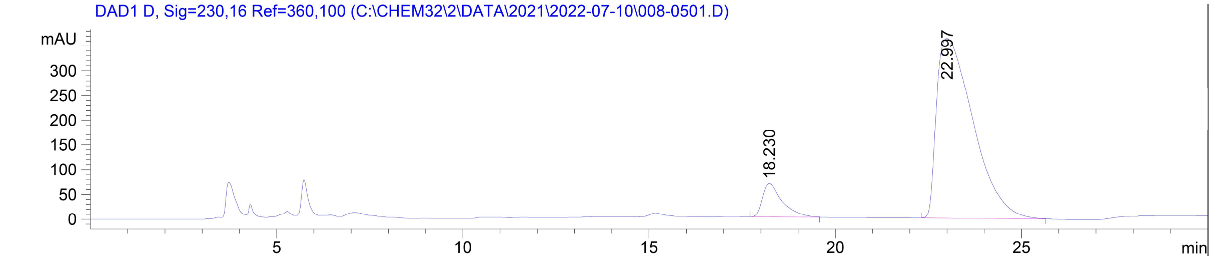

Signal 3: DAD1 D, Sig=230,16 Ref=360,100

Peak #	RetTime [min]	Type	Width [min]	Area [mAU*s]	Height [mAU]	Area %
1	18.230	BB	0.5442	2477.47339	67.18789	9.0861
2	22.997	BB	1.0859	2.47891e4	363.38428	90.9139

Totals : 2.72666e4 430.57217

3-((2S,3R)-3-methyl-1-tosylaziridin-2-yl)propyl benzoate (**32**):

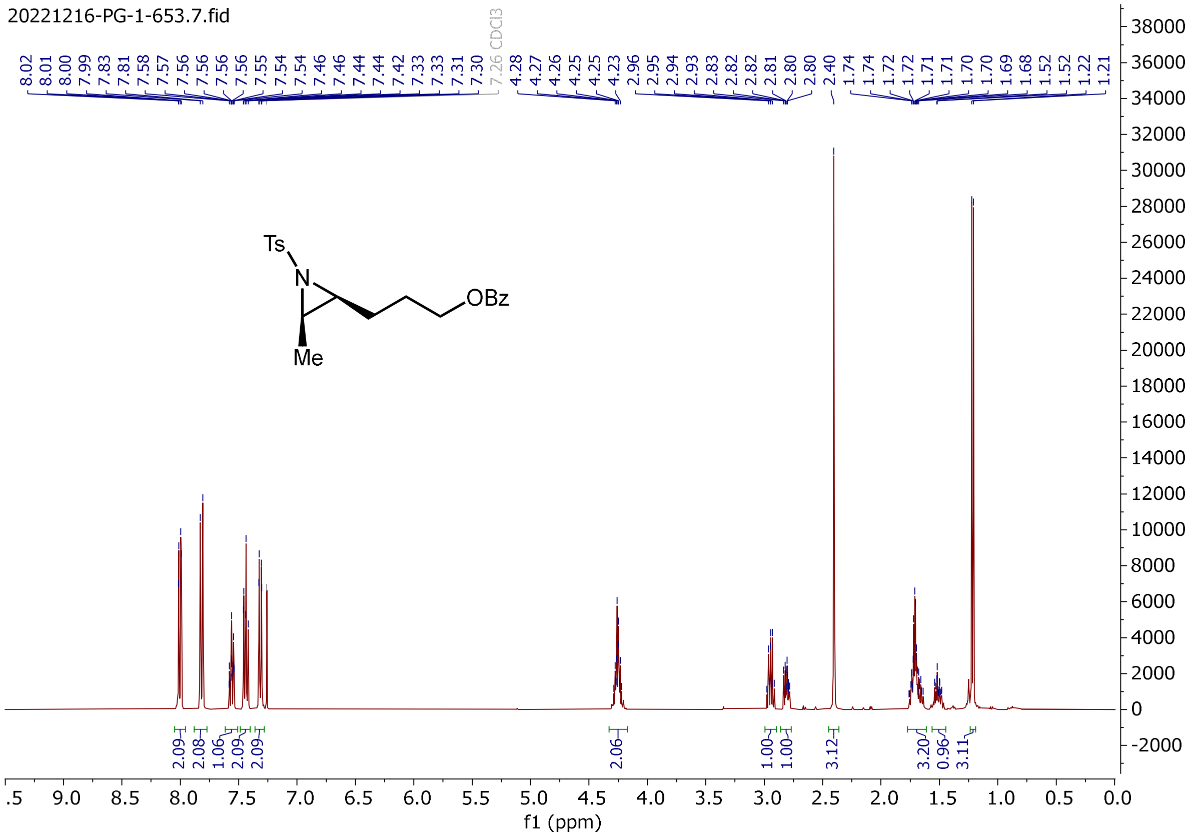

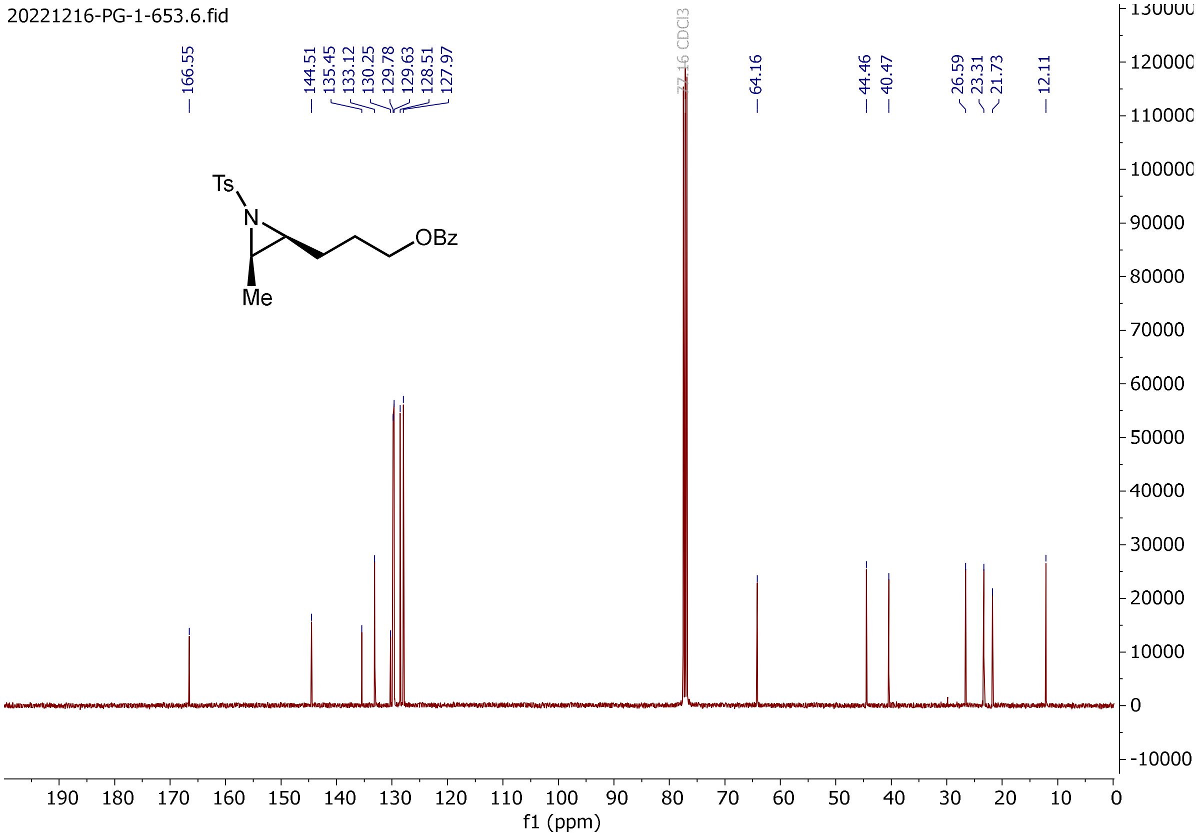

3-(3-methyl-1-tosylaziridin-2-yl)propyl benzoate (**32**):

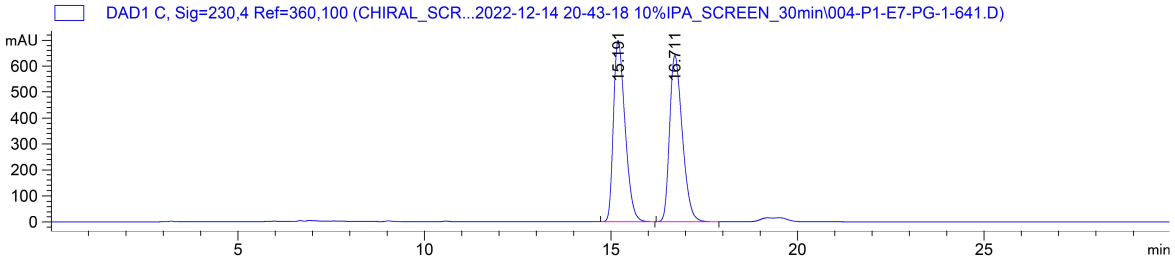

Signal 3: DAD1 C, Sig=230,4 Ref=360,100

Peak #	RetTime [min]	Type	Width [min]	Area [mAU*s]	Height [mAU]	Area %
1	15.191	BB	0.3271	1.51322e4	698.88940	49.9512
2	16.711	BB	0.3551	1.51618e4	639.47821	50.0488
Totals :				3.02939e4	1338.36761	

3-((2S,3R)-3-methyl-1-tosylaziridin-2-yl)propyl benzoate (**32**):

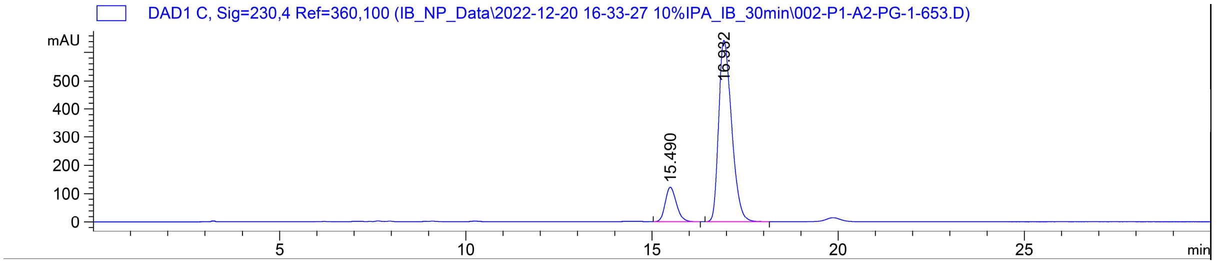

Signal 3: DAD1 C, Sig=230,4 Ref=360,100

Peak #	RetTime [min]	Type	Width [min]	Area [mAU*s]	Height [mAU]	Area %
1	15.490	BB	0.3032	2553.55298	121.48096	14.1081
2	16.932	BB	0.3674	1.55464e4	643.39404	85.8919
Totals :				1.80999e4	764.87500	

5-methyl-6-((4-methylphenyl)sulfonamido)hex-4-en-1-yl benzoate (**34**):

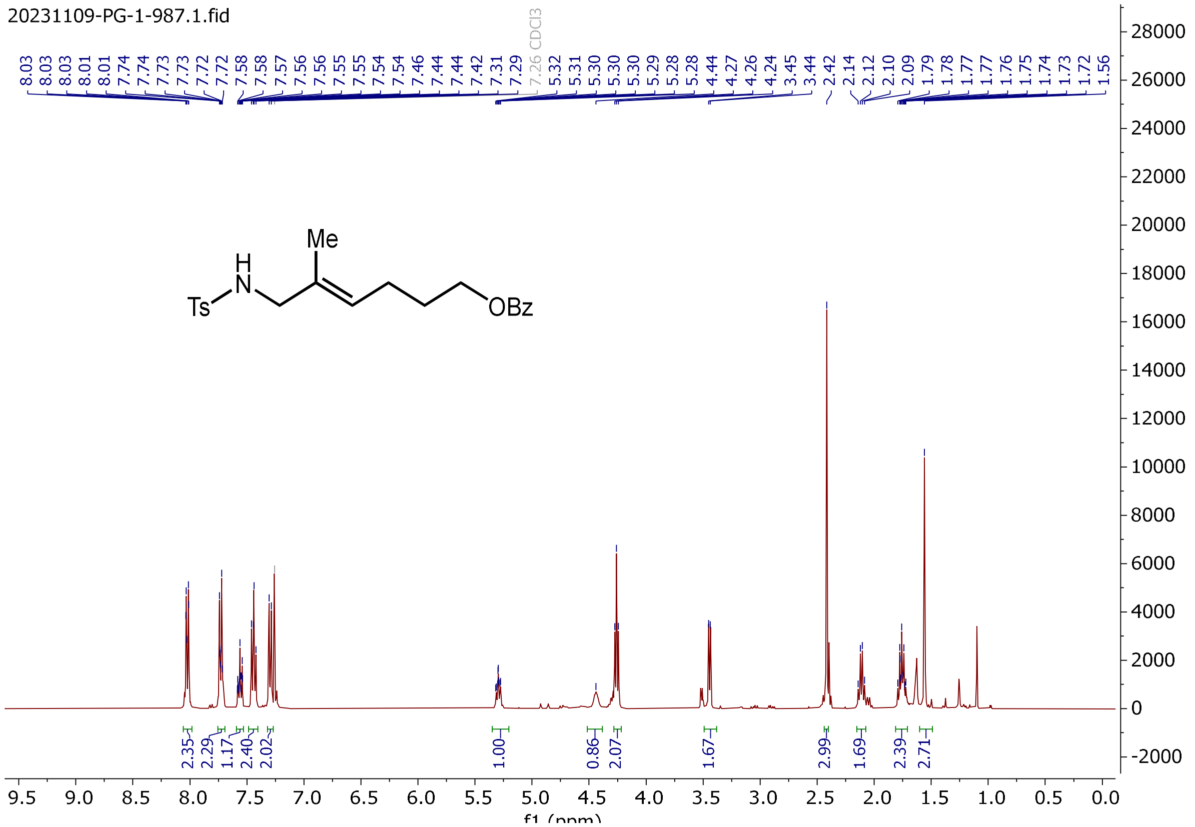

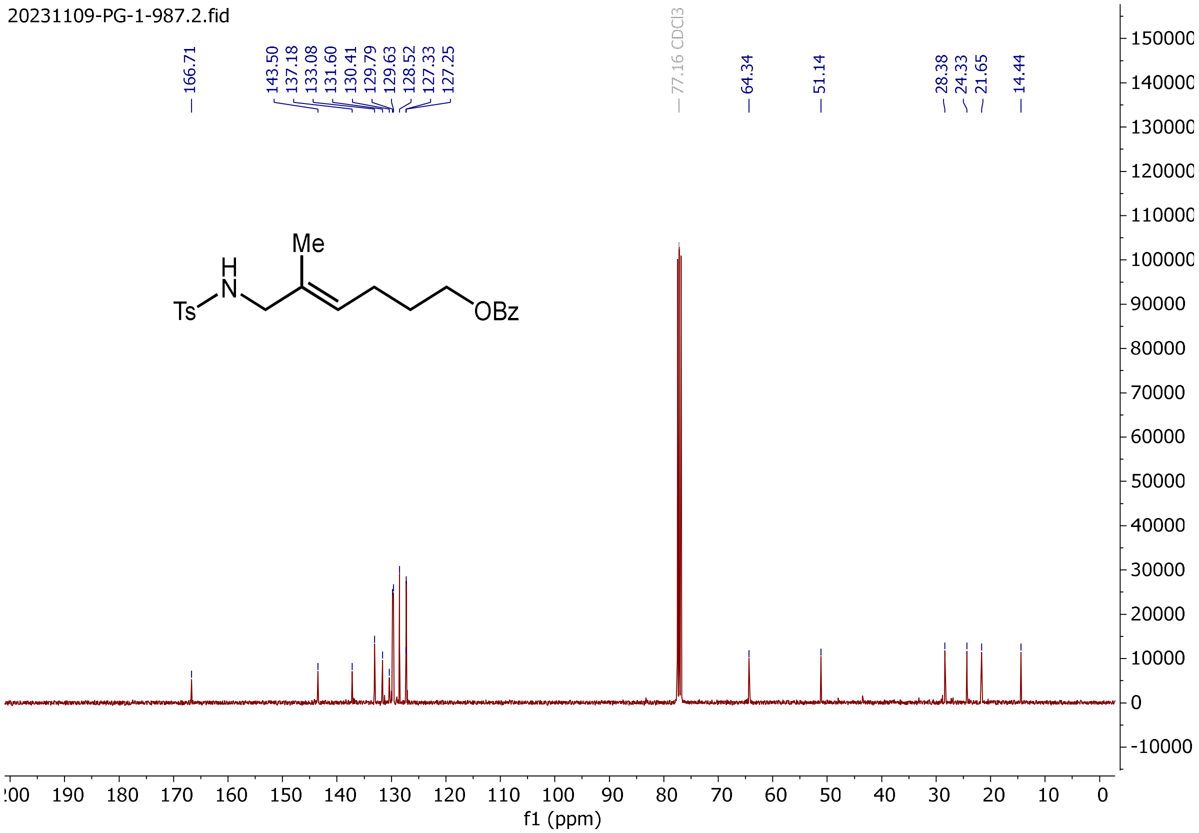

(*S*)-2-(4-phenylbutyl)-1-tosylaziridine (**35**):

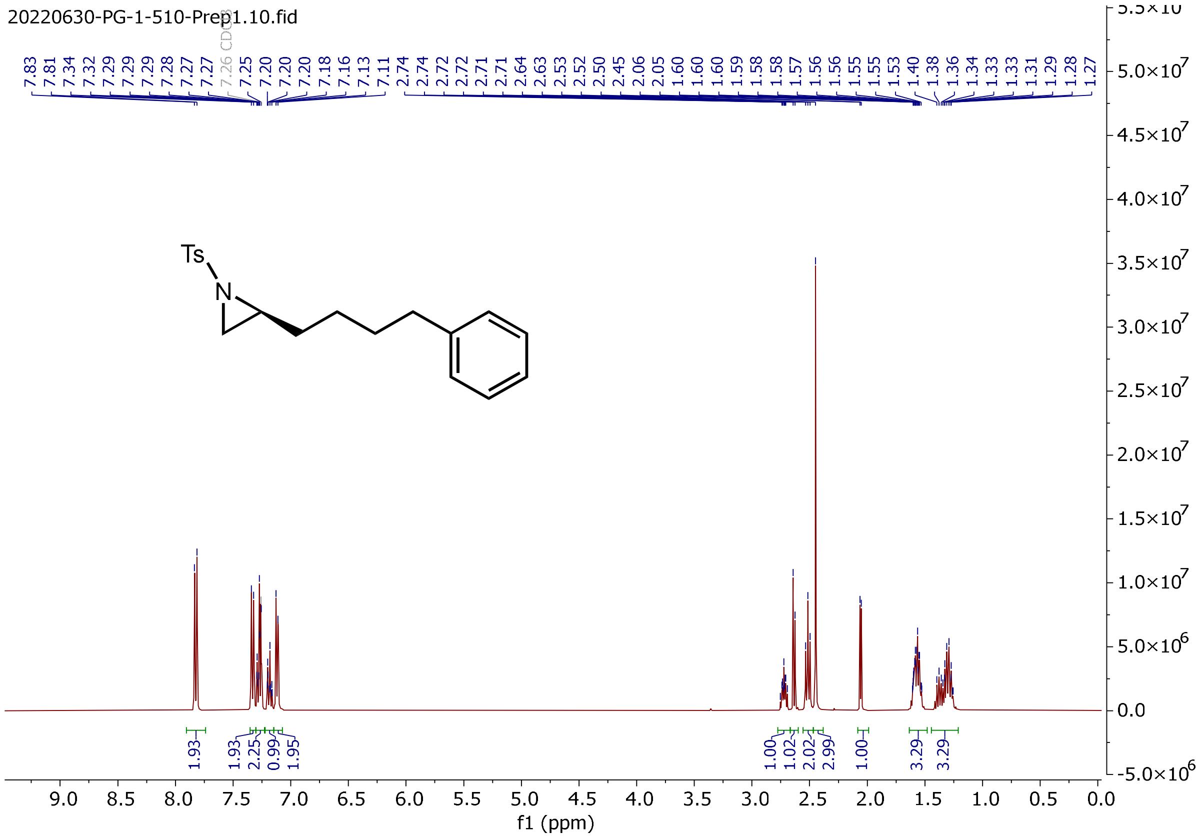

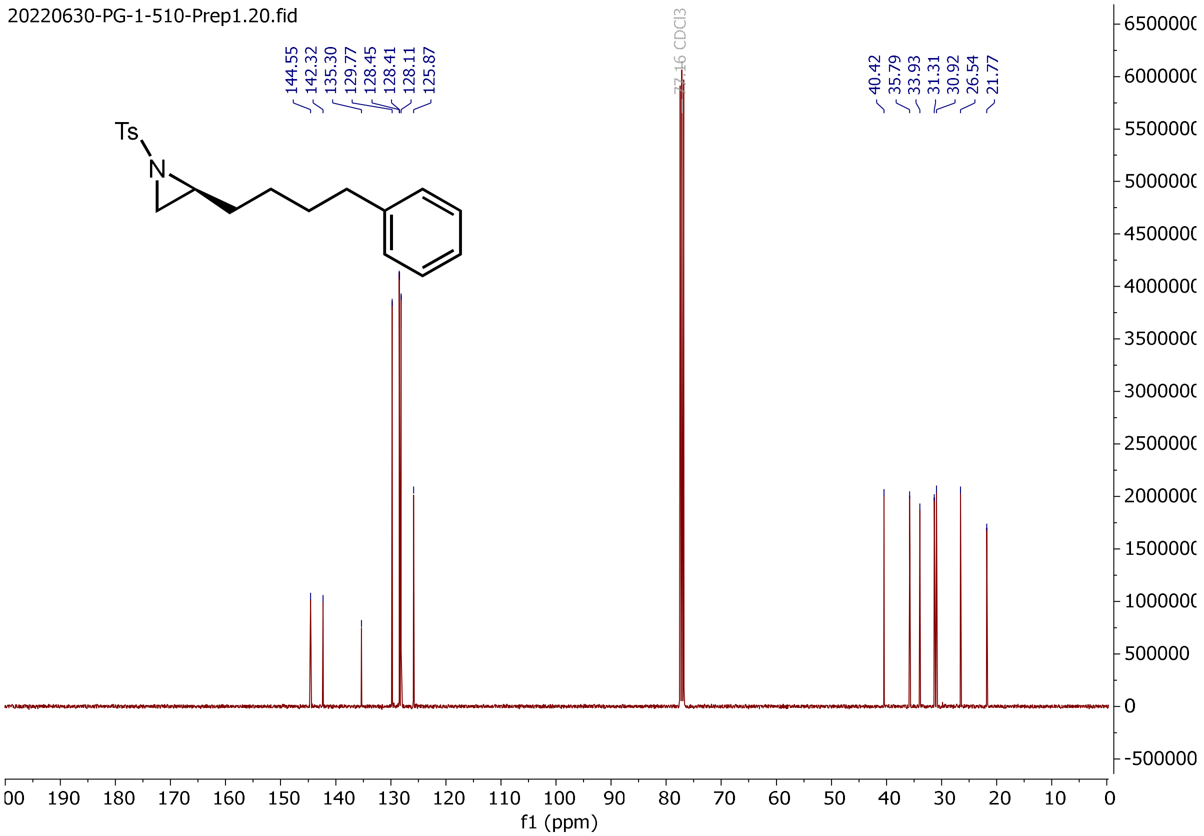

(±)-2-(4-phenylbutyl)-1-tosylaziridine (±-**35**):

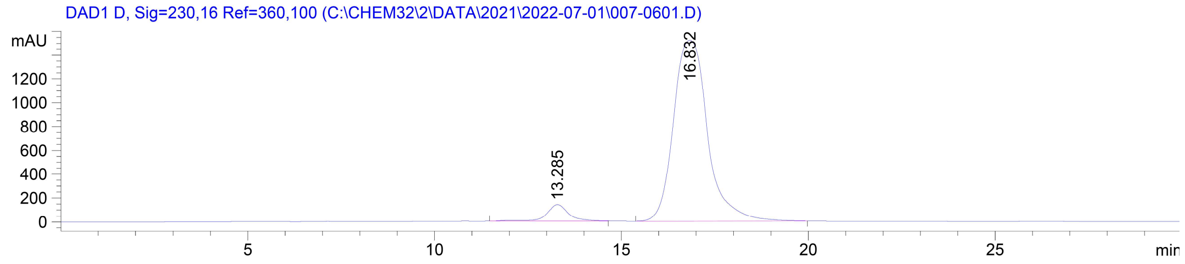

Signal 3: DAD1 D, Sig=230,16 Ref=360,100

Peak #	RetTime [min]	Type	Width [min]	Area [mAU*s]	Height [mAU]	Area %
1	12.921	BB	0.6441	4.17986e4	978.96667	50.1762
2	16.373	BB	0.7983	4.15050e4	793.39551	49.8238

Totals : 8.33036e4 1772.36218

(*S*)-2-(4-phenylbutyl)-1-tosylaziridine (**35**):

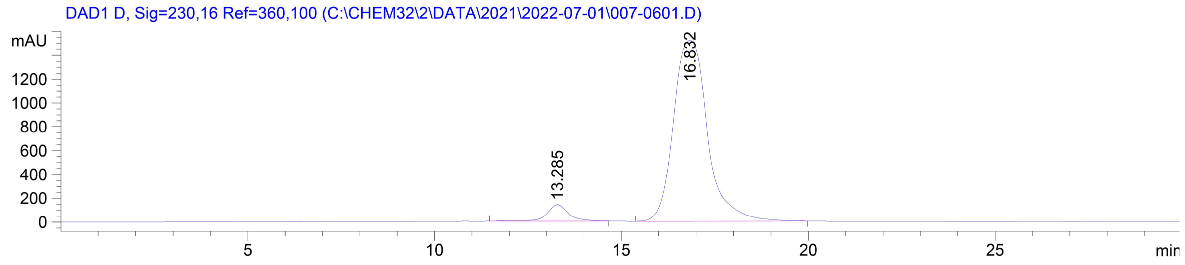

Signal 3: DAD1 D, Sig=230,16 Ref=360,100

Peak #	RetTime [min]	Type	Width [min]	Area [mAU*s]	Height [mAU]	Area %
1	13.285	BB	0.6062	5664.38184	138.18881	5.5709
2	16.832	BB	0.9873	9.60131e4	1519.34229	94.4291

Totals : 1.01677e5 1657.53110

(*S*)-2-(3-phenylpropyl)-1-tosylaziridine (**36**):

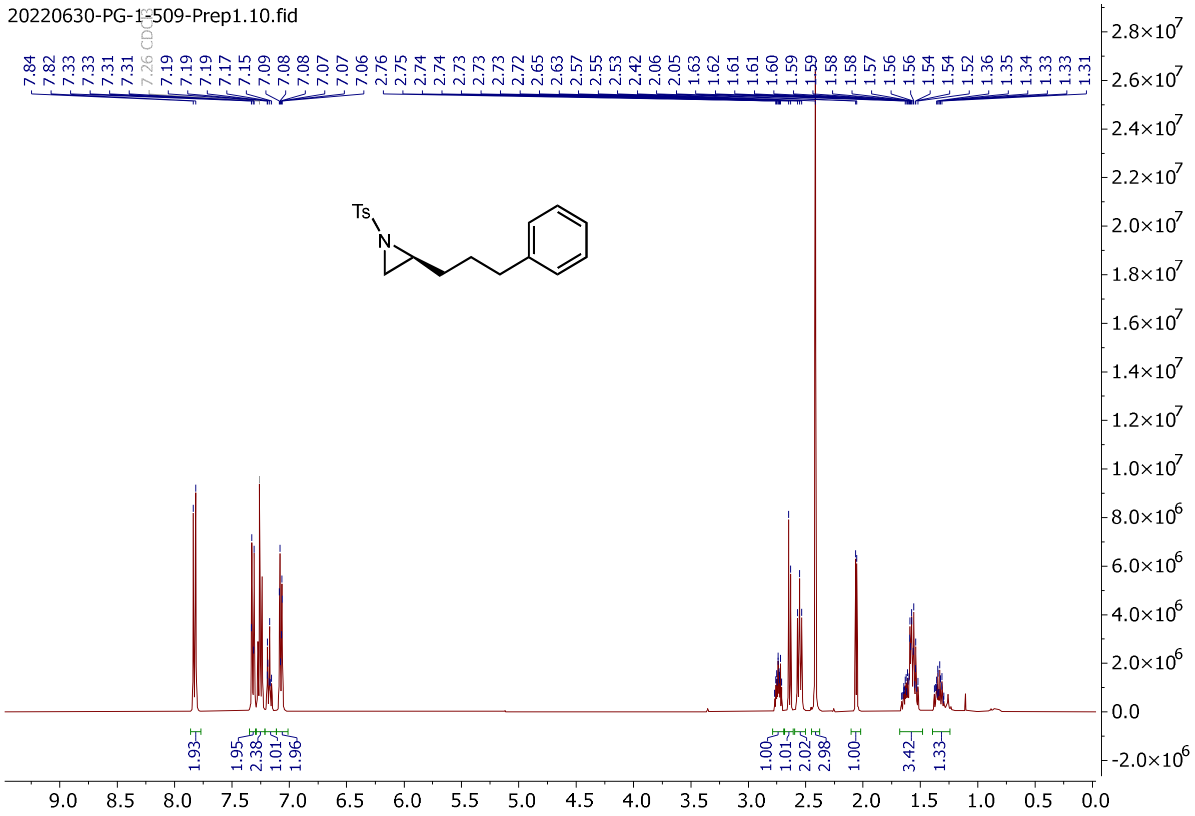

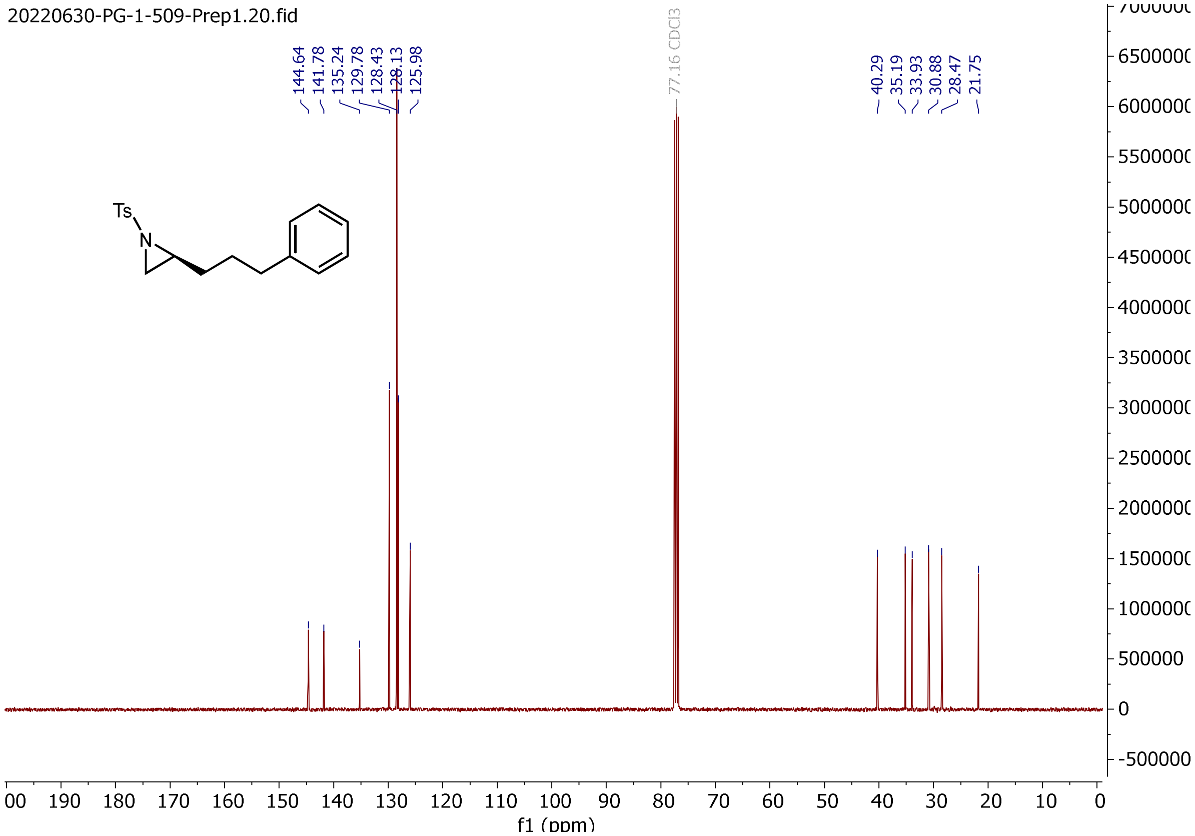

(±)-2-(3-phenylpropyl)-1-tosylaziridine (**36**):

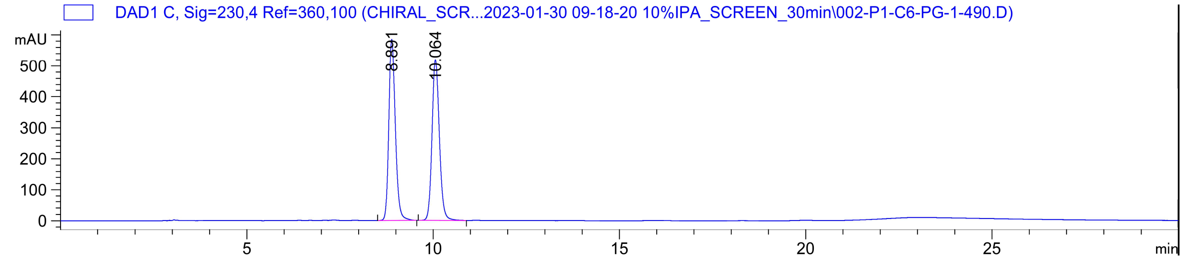

Signal 3: DAD1 C, Sig=230,4 Ref=360,100

Peak #	RetTime [min]	Type	Width [min]	Area [mAU*s]	Height [mAU]	Area %
1	8.891	BB	0.1872	7120.35449	584.38434	50.0245
2	10.064	BB	0.2102	7113.39063	519.70685	49.9755
Totals :				1.42337e4	1104.09119	

(*S*)-2-(3-phenylpropyl)-1-tosylaziridine (**36**):

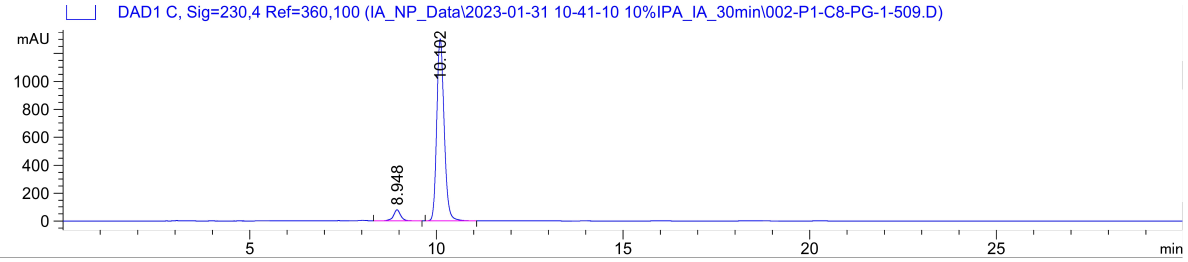

Signal 3: DAD1 C, Sig=230,4 Ref=360,100

Peak #	RetTime [min]	Type	Width [min]	Area [mAU*s]	Height [mAU]	Area %
1	8.948	BB	0.2027	1072.08289	78.33858	5.6308
2	10.102	BB	0.2135	1.79675e4	1302.09705	94.3692
Totals :				1.90396e4	1380.43562	

(*S*)-2-benzyl-1-tosylaziridine (**37**):

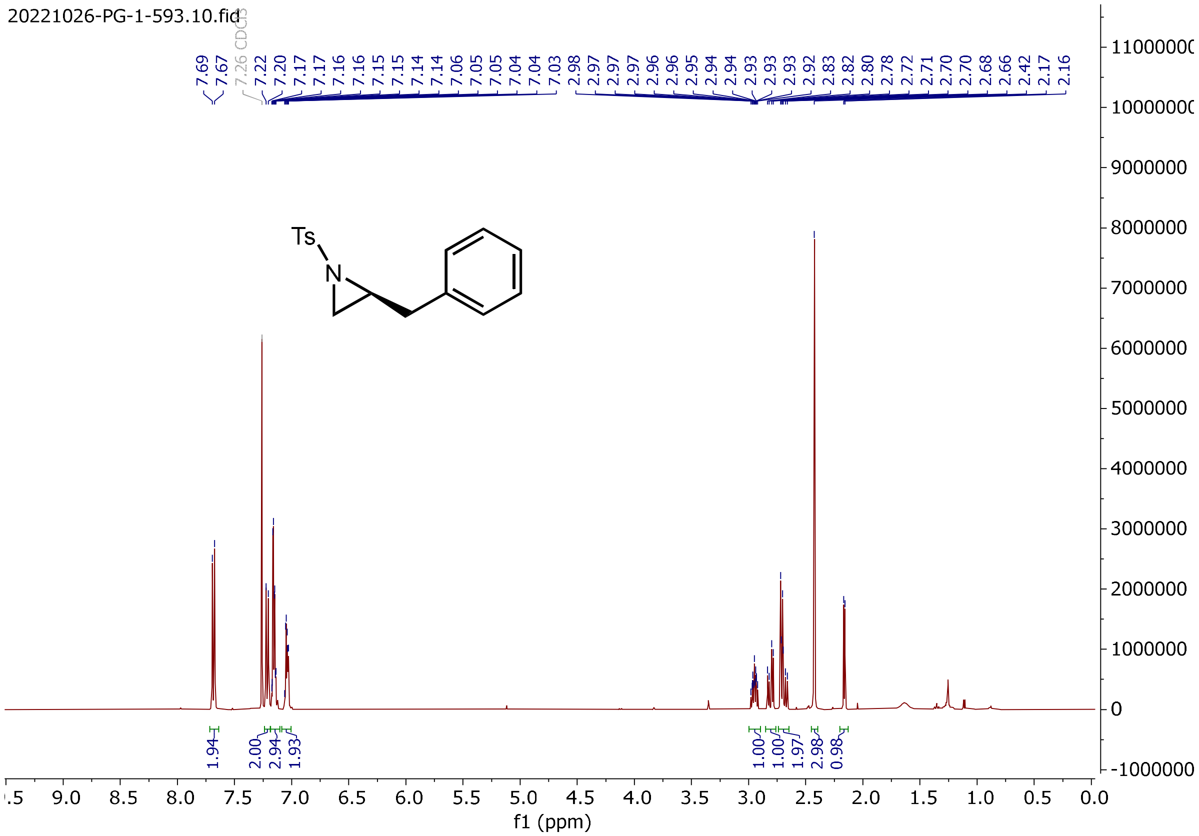

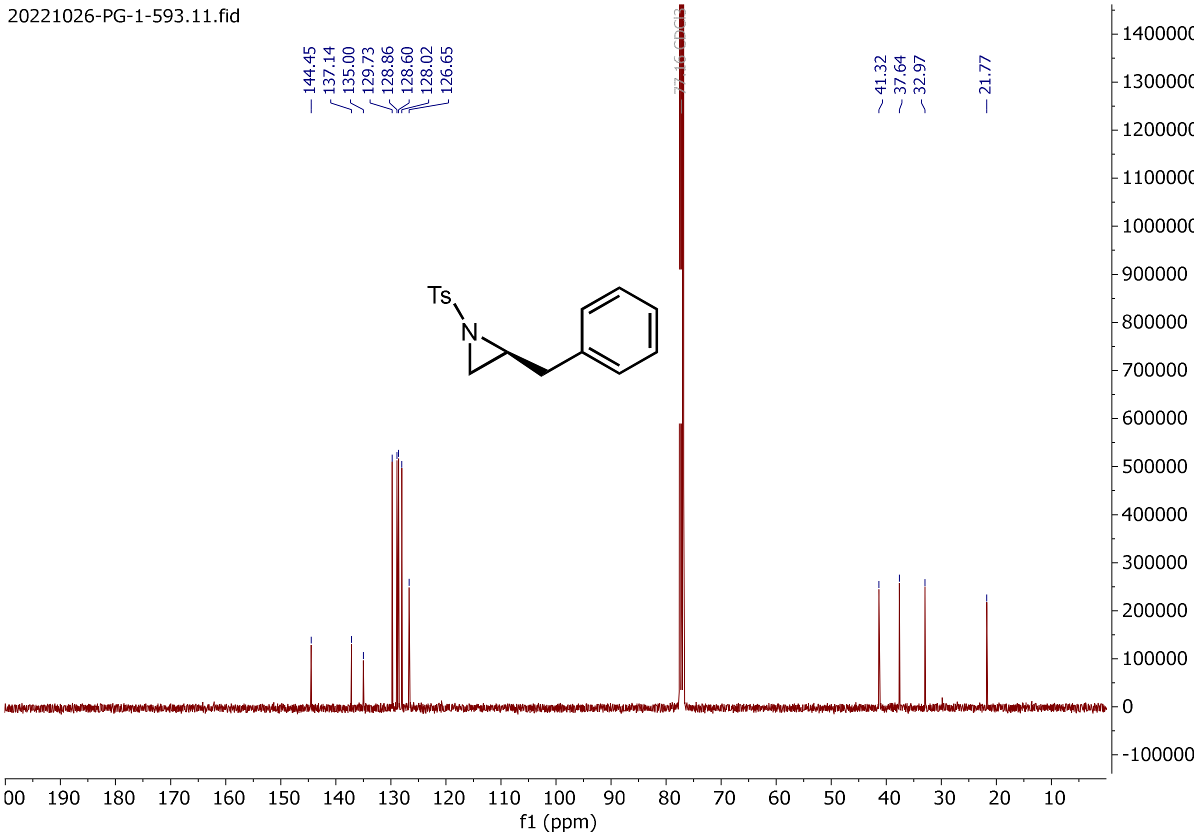

(±)-2-benzyl-1-tosylaziridine (±-**37**):

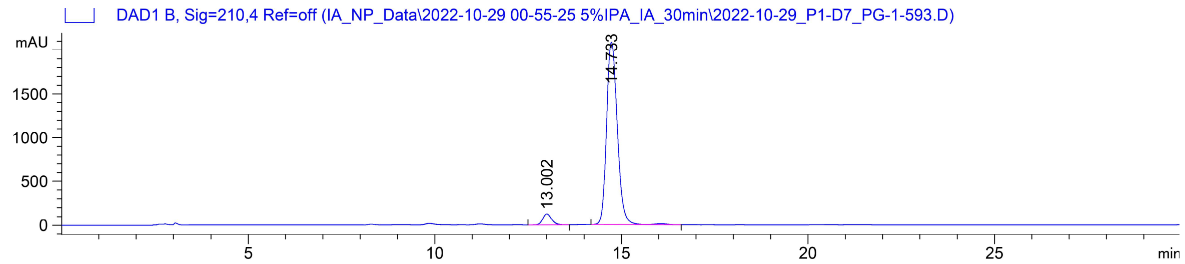

Signal 2: DAD1 B, Sig=210,4 Ref=off

Peak #	RetTime [min]	Type	Width [min]	Area [mAU*s]	Height [mAU]	Area %
1	13.002	BB	0.2471	2112.04272	127.29562	4.8152
2	14.733	BV R	0.2419	4.17496e4	2081.45728	95.1848
Totals :				4.38617e4	2208.75290	

(*S*)-2-benzyl-1-tosylaziridine (**37**):

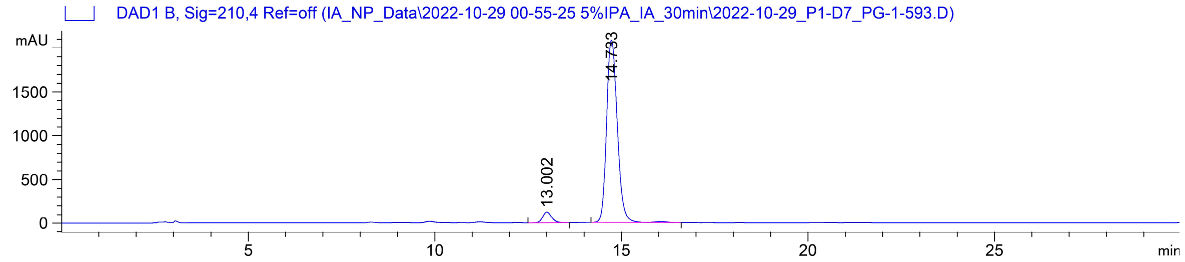

Signal 2: DAD1 B, Sig=210,4 Ref=off

Peak #	RetTime [min]	Type	Width [min]	Area [mAU*s]	Height [mAU]	Area %
1	13.002	BB	0.2471	2112.04272	127.29562	4.8152
2	14.733	BV R	0.2419	4.17496e4	2081.45728	95.1848
Totals :				4.38617e4	2208.75290	

(S)-1-((4-nitrophenyl)sulfonyl)-2-(4-phenylbutyl)aziridine (**38**):

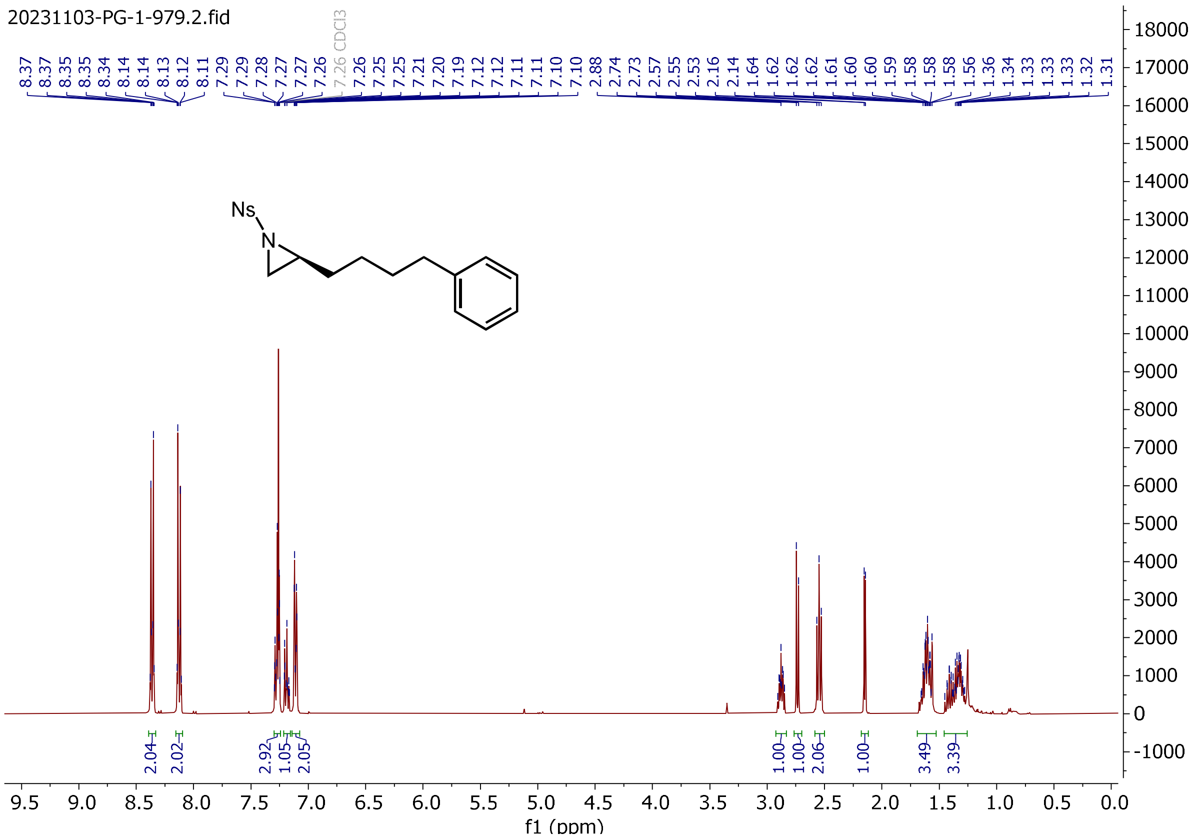

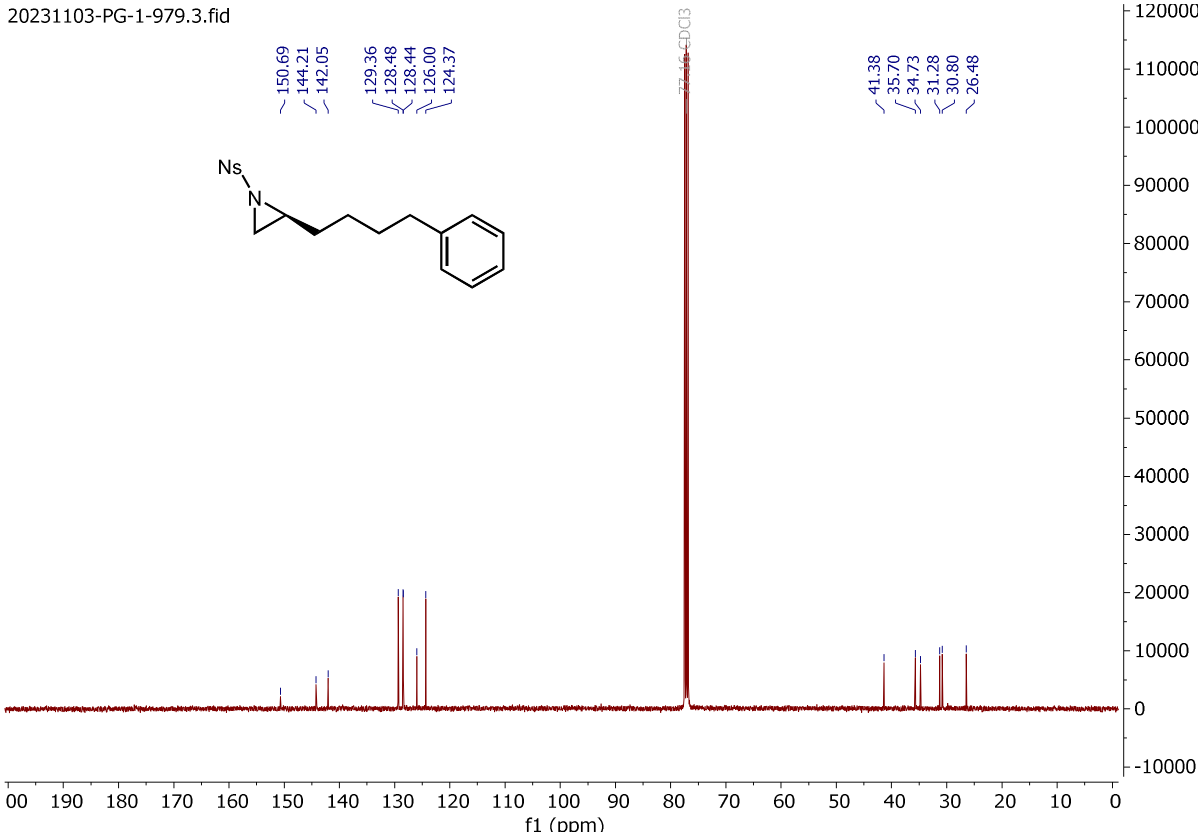

(±)-1-((4-nitrophenyl)sulfonyl)-2-(4-phenylbutyl)aziridine (±-**38**):

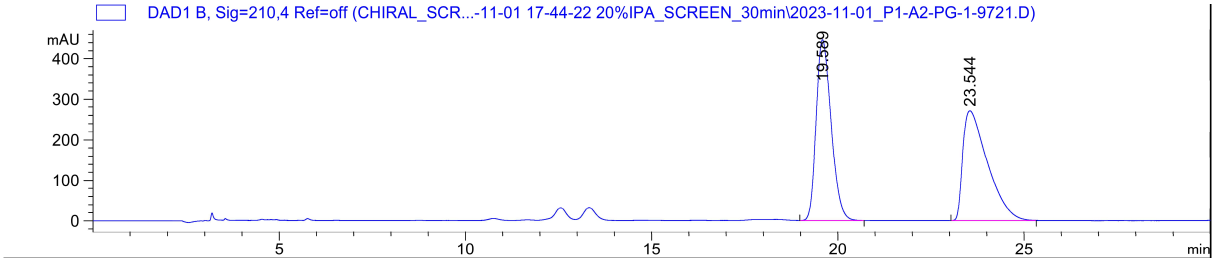

Signal 2: DAD1 B, Sig=210,4 Ref=off

Peak #	RetTime [min]	Type	Width [min]	Area [mAU*s]	Height [mAU]	Area %
1	19.589	BB	0.3499	1.24868e4	446.29572	49.9748
2	23.544	BV R	0.5451	1.24993e4	270.50516	50.0252
Totals :				2.49861e4	716.80087	

(S)-1-((4-nitrophenyl)sulfonyl)-2-(4-phenylbutyl)aziridine (**38**):

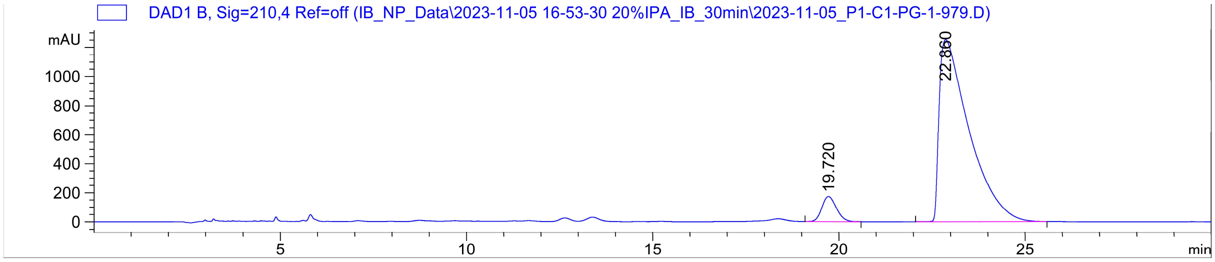

Signal 2: DAD1 B, Sig=210,4 Ref=off

Peak #	RetTime [min]	Type	Width [min]	Area [mAU*s]	Height [mAU]	Area %
1	19.720	VV R	0.3241	4633.23389	171.83258	6.2234
2	22.860	VV R	0.6527	6.98153e4	1251.65906	93.7766
Totals :				7.44485e4	1423.49164	

(S)-1-(methylsulfonyl)-2-(4-phenylbutyl)aziridine (**39**):

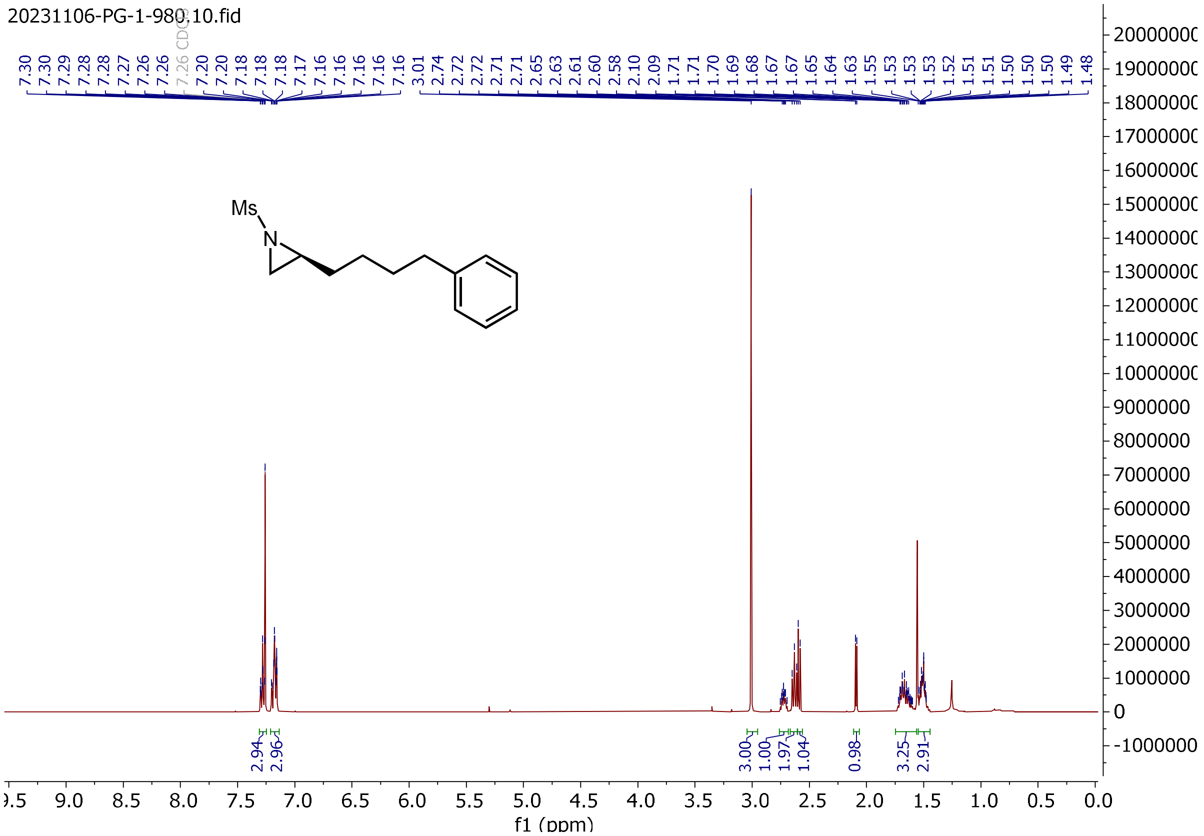

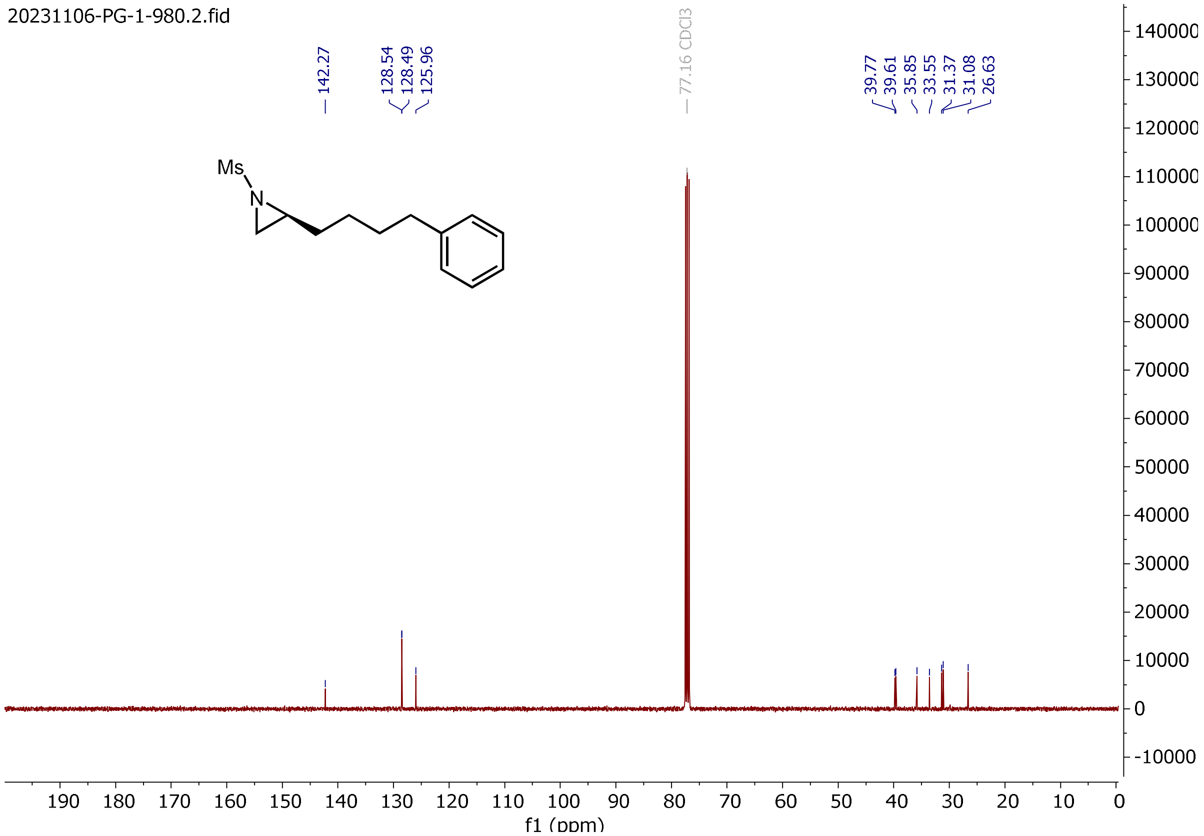

(±)-1-(methylsulfonyl)-2-(4-phenylbutyl)aziridine (±-**39**):

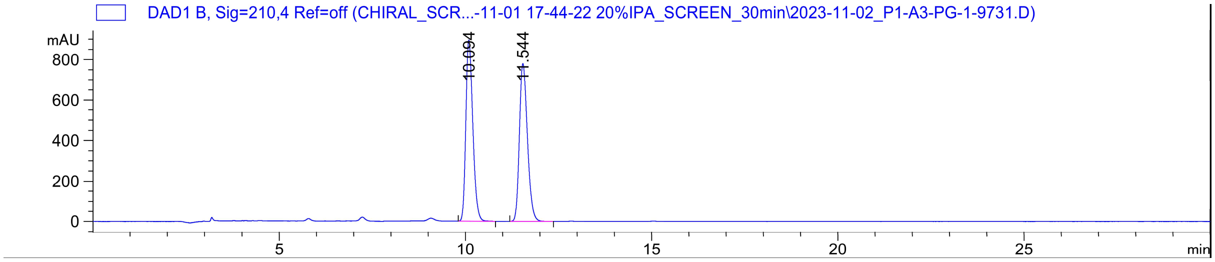

Signal 2: DAD1 B, Sig=210,4 Ref=off

Peak #	RetTime [min]	Type	Width [min]	Area [mAU*s]	Height [mAU]	Area %
1	10.094	BB	0.1957	1.16009e4	894.92395	49.9585
2	11.544	VV R	0.2199	1.16202e4	779.35724	50.0415

Totals : 2.32211e4 1674.28119

(S)-1-(methylsulfonyl)-2-(4-phenylbutyl)aziridine (**39**):

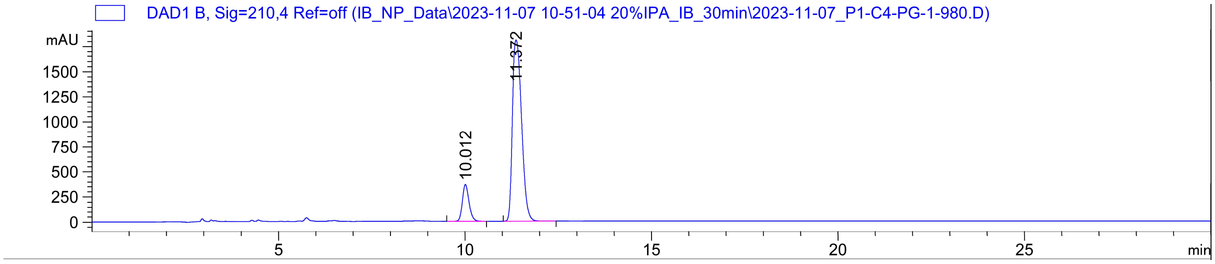

Signal 2: DAD1 B, Sig=210,4 Ref=off

Peak #	RetTime [min]	Type	Width [min]	Area [mAU*s]	Height [mAU]	Area %
1	10.012	BB	0.1892	4505.58838	361.92816	12.7795
2	11.372	BB	0.2437	3.07507e4	1805.56567	87.2205

Totals : 3.52563e4 2167.49384

(S)-2-(4-(allyloxy)butyl)-1-tosylaziridine (**40**):

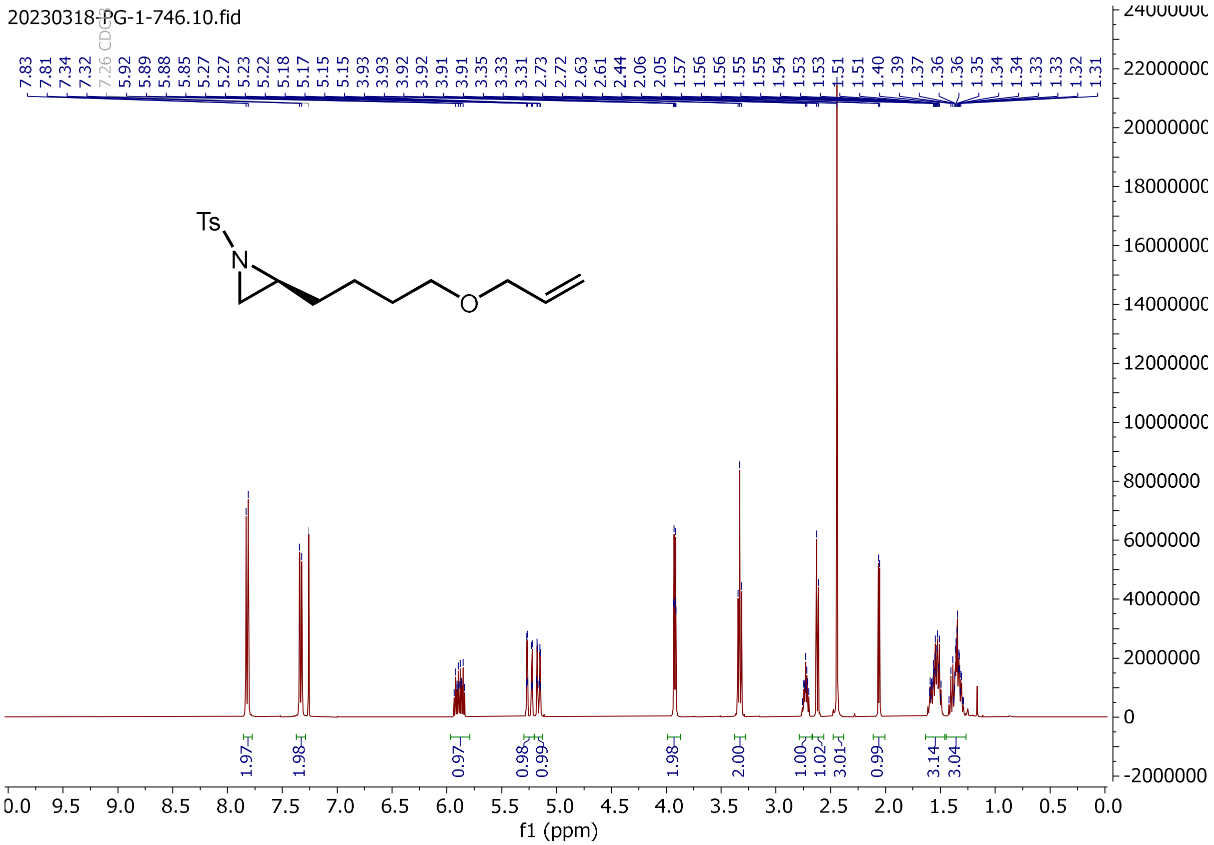

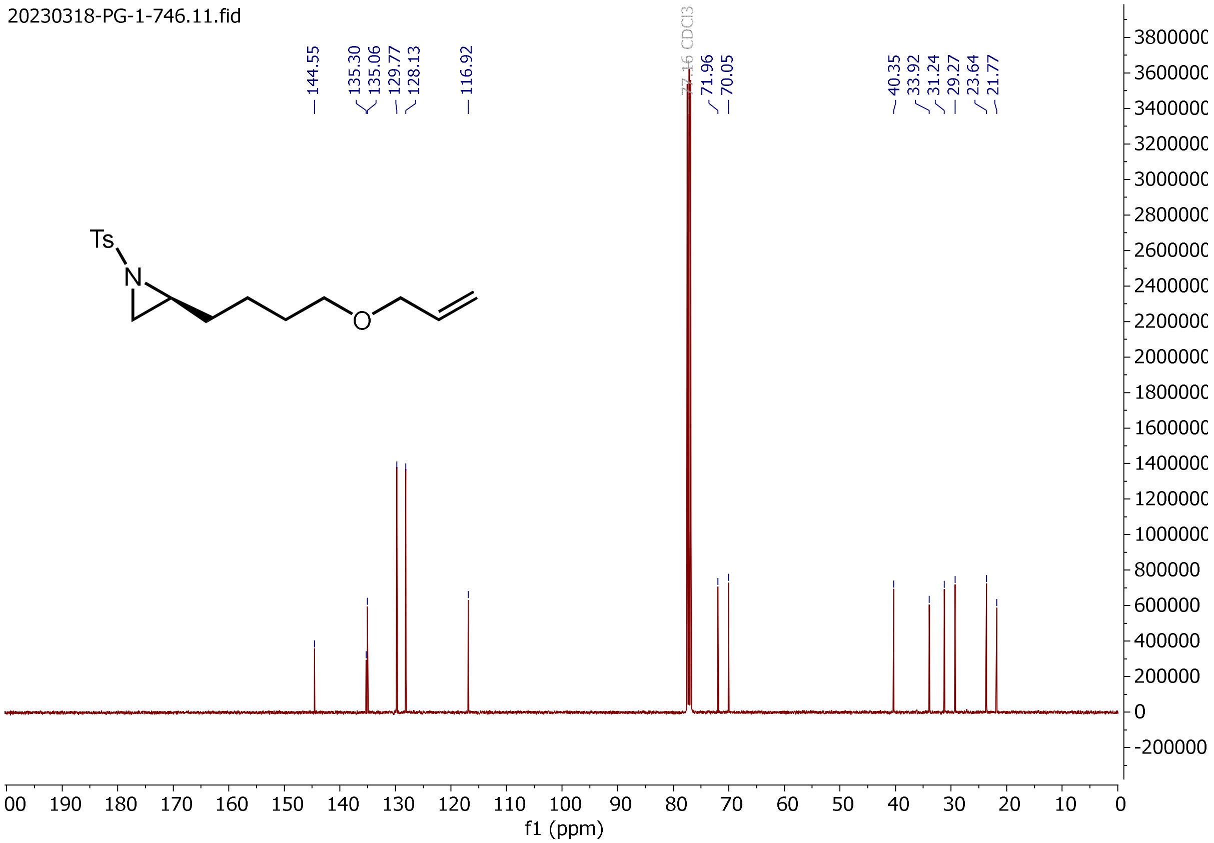

(±)-2-(4-(allyloxy)butyl)-1-tosylaziridine (±-**40**):

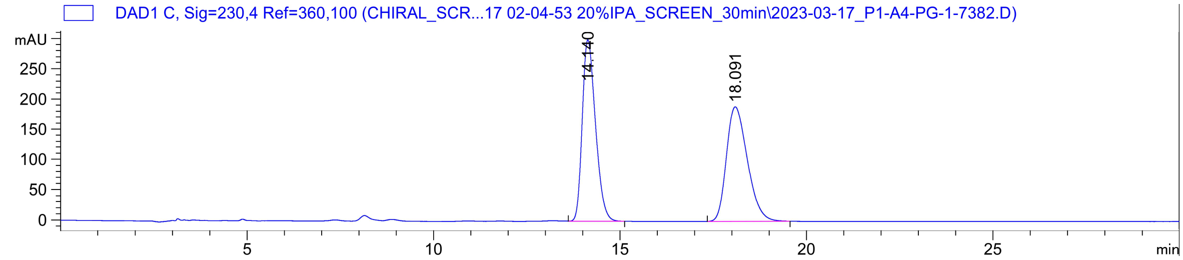

Signal 3: DAD1 C, Sig=230,4 Ref=360,100

Peak #	RetTime [min]	Type	Width [min]	Area [mAU*s]	Height [mAU]	Area %
1	14.140	BB	0.3654	7385.88965	299.78070	50.0015
2	18.091	BB	0.4703	7385.44824	189.85191	49.9985

Totals : 1.47713e4 489.63261

(S)-2-(4-(allyloxy)butyl)-1-tosylaziridine (**40**):

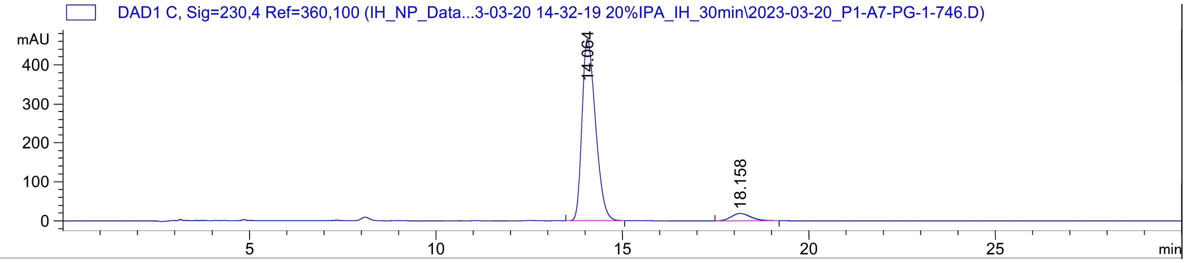

Signal 3: DAD1 C, Sig=230,4 Ref=360,100

Peak #	RetTime [min]	Type	Width [min]	Area [mAU*s]	Height [mAU]	Area %
1	14.064	BV R	0.3724	1.16835e4	464.60098	94.5954
2	18.158	BB	0.4242	667.52789	18.50953	5.4046

Totals : 1.23511e4 483.11051

(S)-4-(1-tosylaziridin-2-yl)butyl acrylate (**41**):

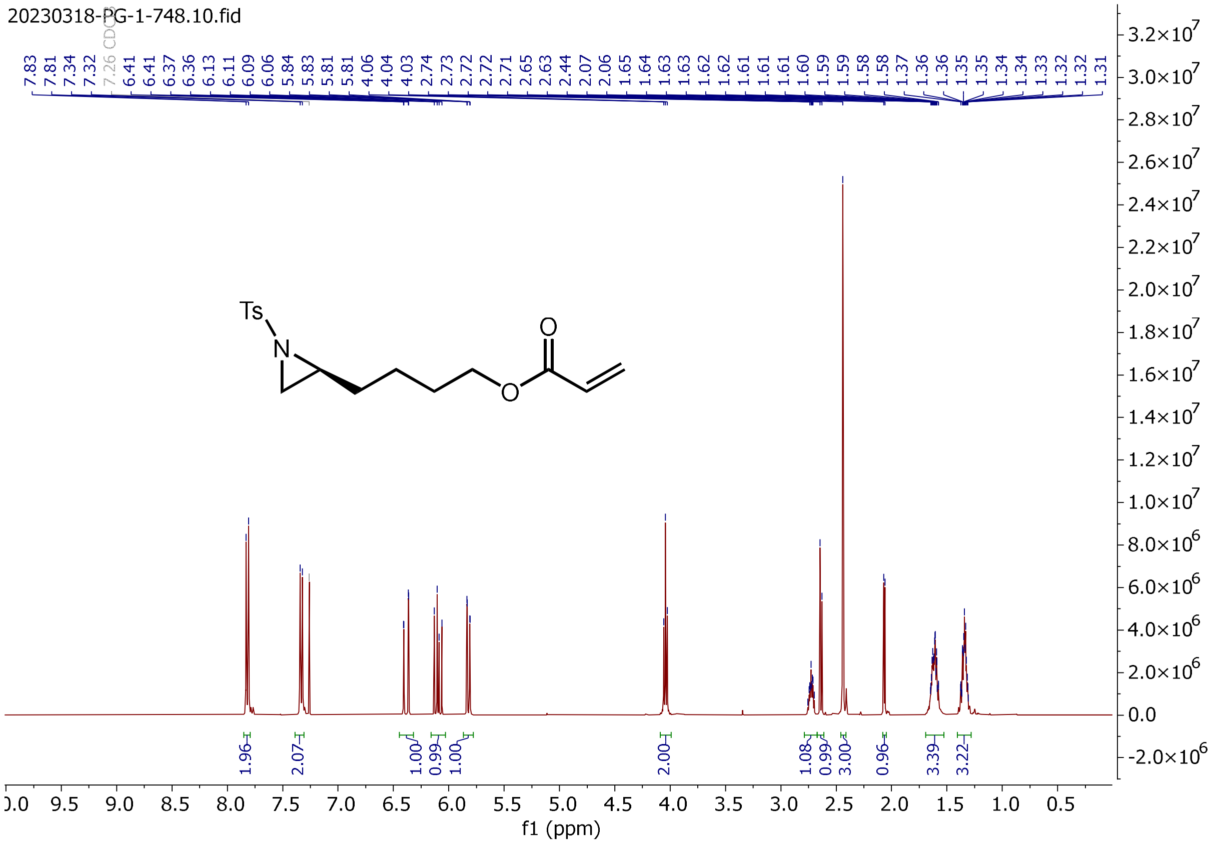

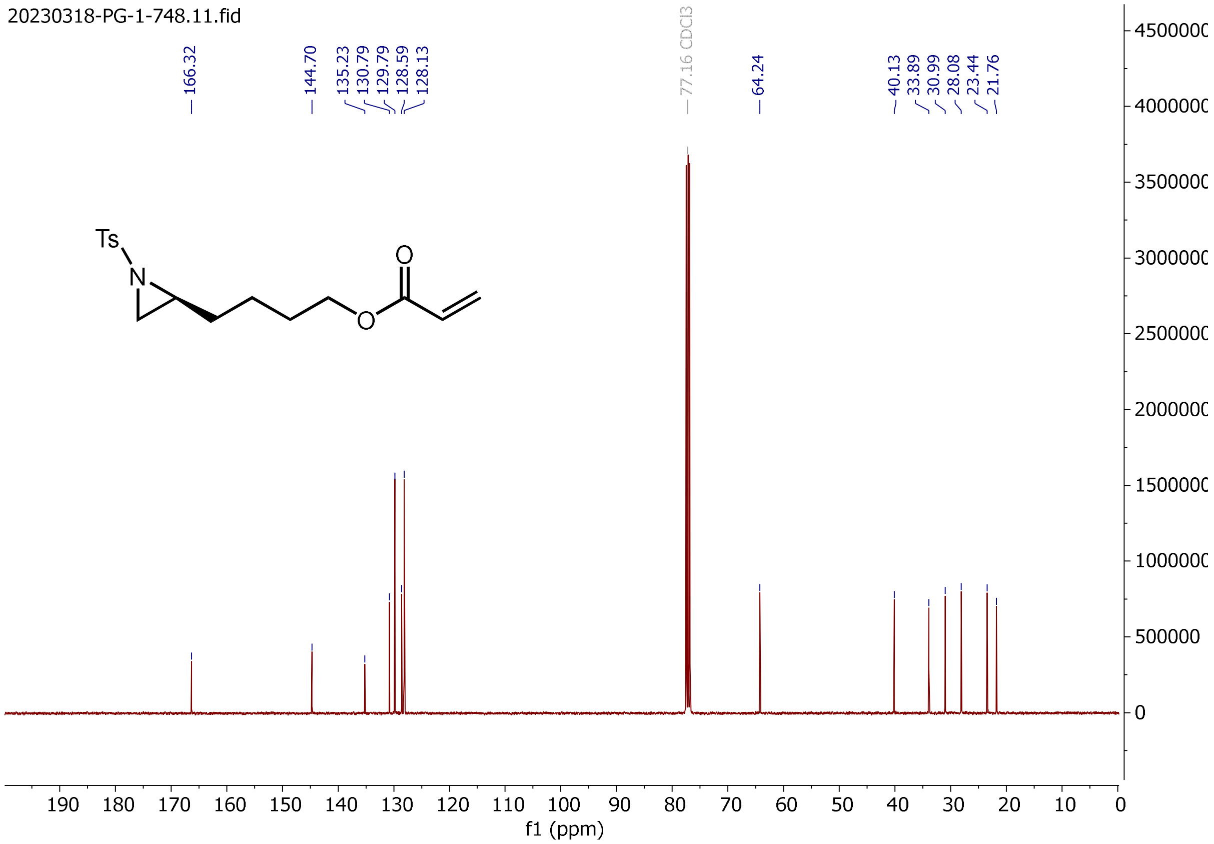

(±)-4-(1-tosylaziridin-2-yl)butyl acrylate (±-**41**):

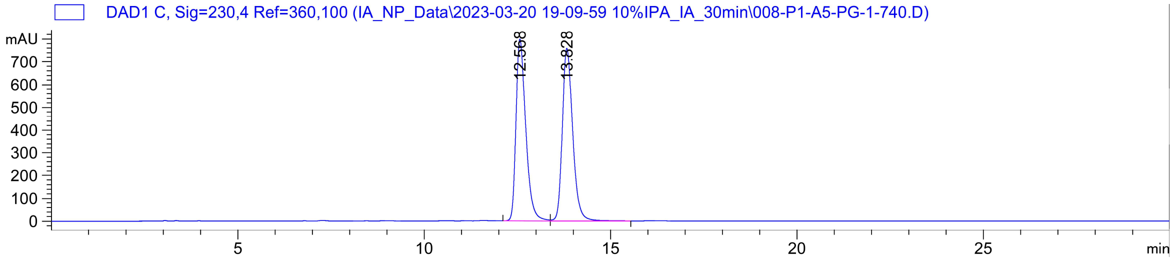

Signal 3: DAD1 C, Sig=230,4 Ref=360,100

Peak #	RetTime [min]	Type	Width [min]	Area [mAU*s]	Height [mAU]	Area %
1	12.568	BV	0.2773	1.45193e4	797.76776	49.7555
2	13.828	VB	0.2902	1.46620e4	757.92334	50.2445

Totals : 2.91812e4 1555.69110

(S)-4-(1-tosylaziridin-2-yl)butyl acrylate (**41**):

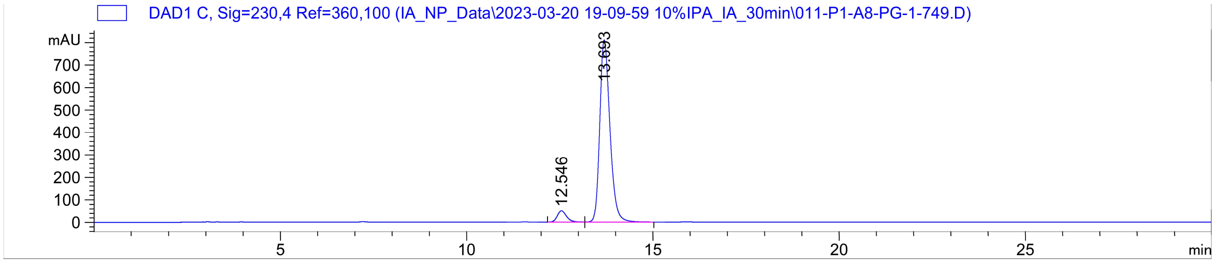

Signal 3: DAD1 C, Sig=230,4 Ref=360,100

Peak #	RetTime [min]	Type	Width [min]	Area [mAU*s]	Height [mAU]	Area %
1	12.546	BB	0.2398	851.45819	49.72660	5.2585
2	13.693	BB	0.2864	1.53406e4	810.22791	94.7415

Totals : 1.61920e4 859.95450

(S)-4-(1-tosylaziridin-2-yl)butyl 4-vinylbenzoate (**42**):

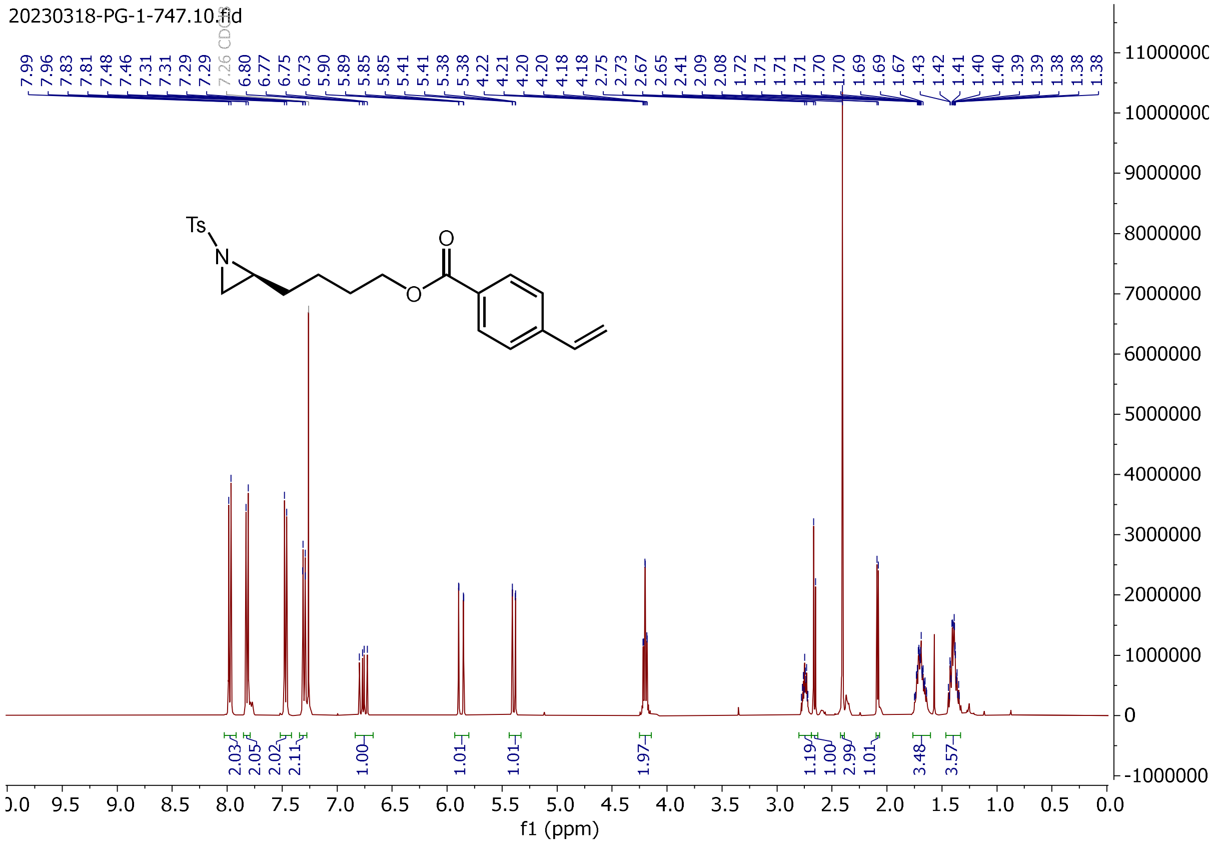

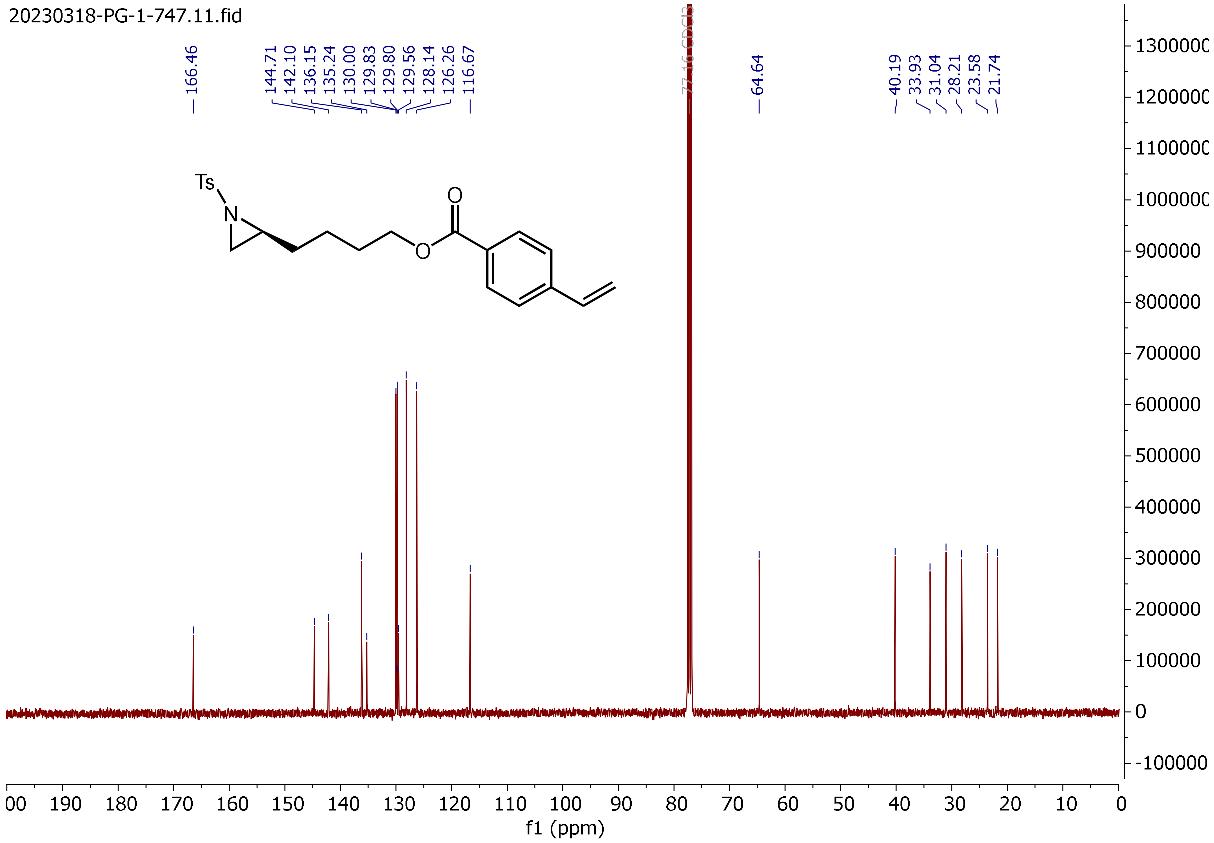

(±)-4-(1-tosylaziridin-2-yl)butyl 4-vinylbenzoate (±-**42**):

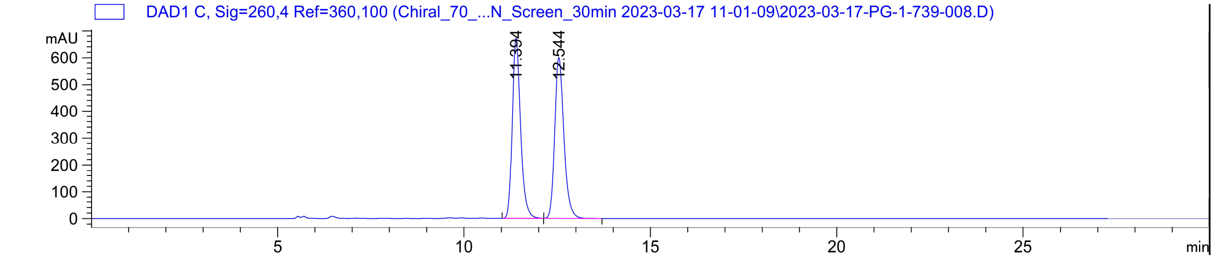

Signal 3: DAD1 C, Sig=260,4 Ref=360,100

Peak #	RetTime [min]	Type	Width [min]	Area [mAU*s]	Height [mAU]	Area %
1	11.394	BB	0.2361	1.03375e4	669.77283	50.0224
2	12.544	BB	0.2627	1.03282e4	600.28094	49.9776
Totals :				2.06657e4	1270.05377	

(S)-4-(1-tosylaziridin-2-yl)butyl 4-vinylbenzoate (**42**):

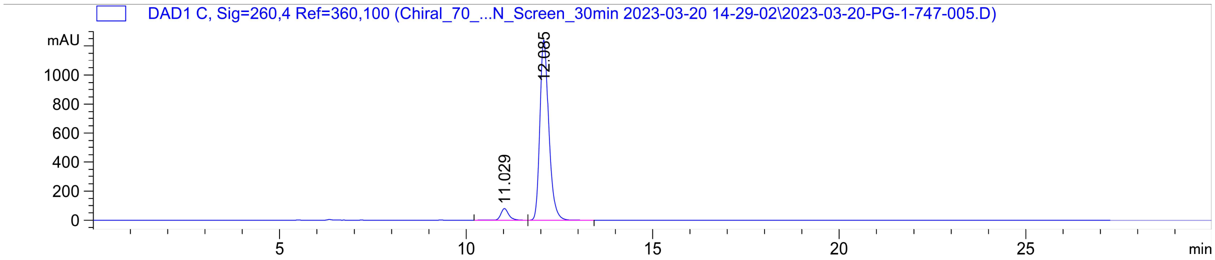

Signal 3: DAD1 C, Sig=260,4 Ref=360,100

Peak #	RetTime [min]	Type	Width [min]	Area [mAU*s]	Height [mAU]	Area %
1	11.029	VB R	0.2222	1176.72290	78.86966	5.4314
2	12.085	BB	0.2528	2.04884e4	1239.53906	94.5686
Totals :				2.16651e4	1318.40872	

(S)-4-(1-tosylaziridin-2-yl)butyl cinnamate (**43**):

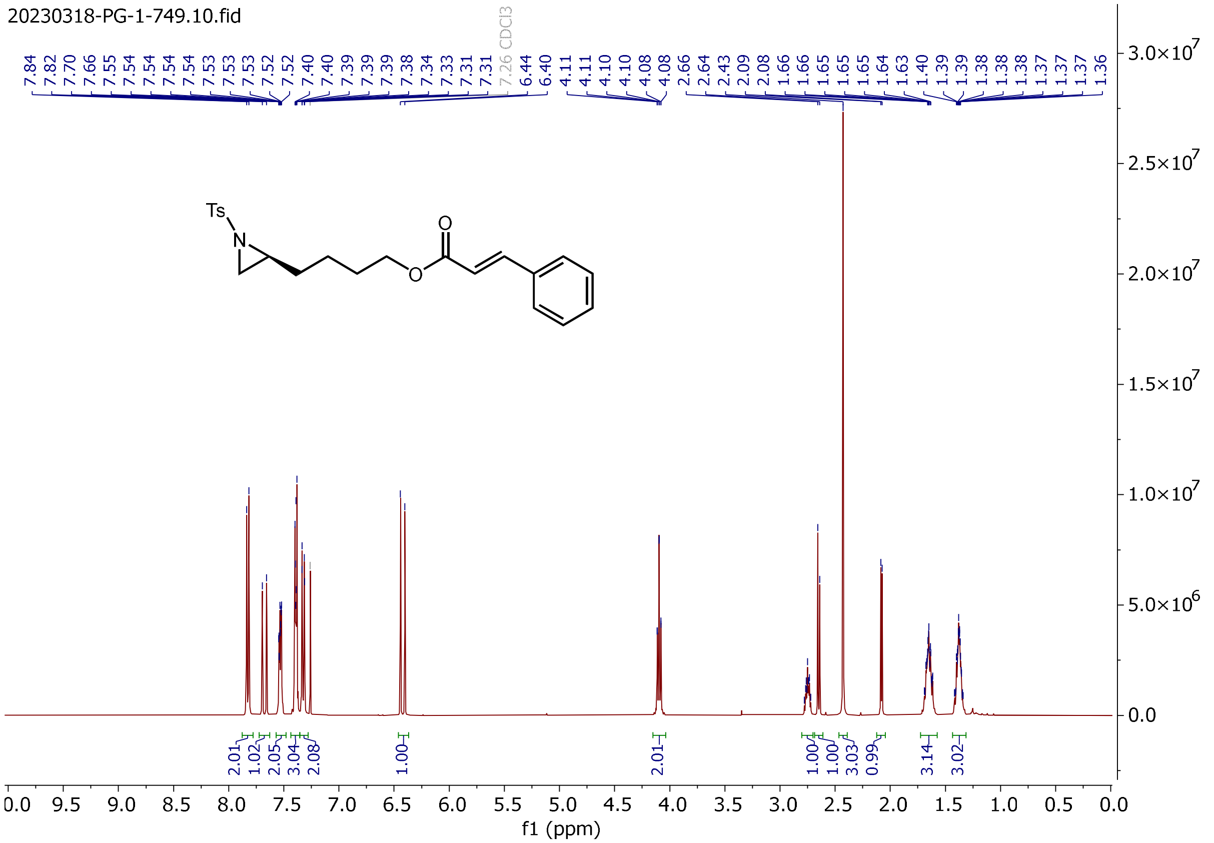

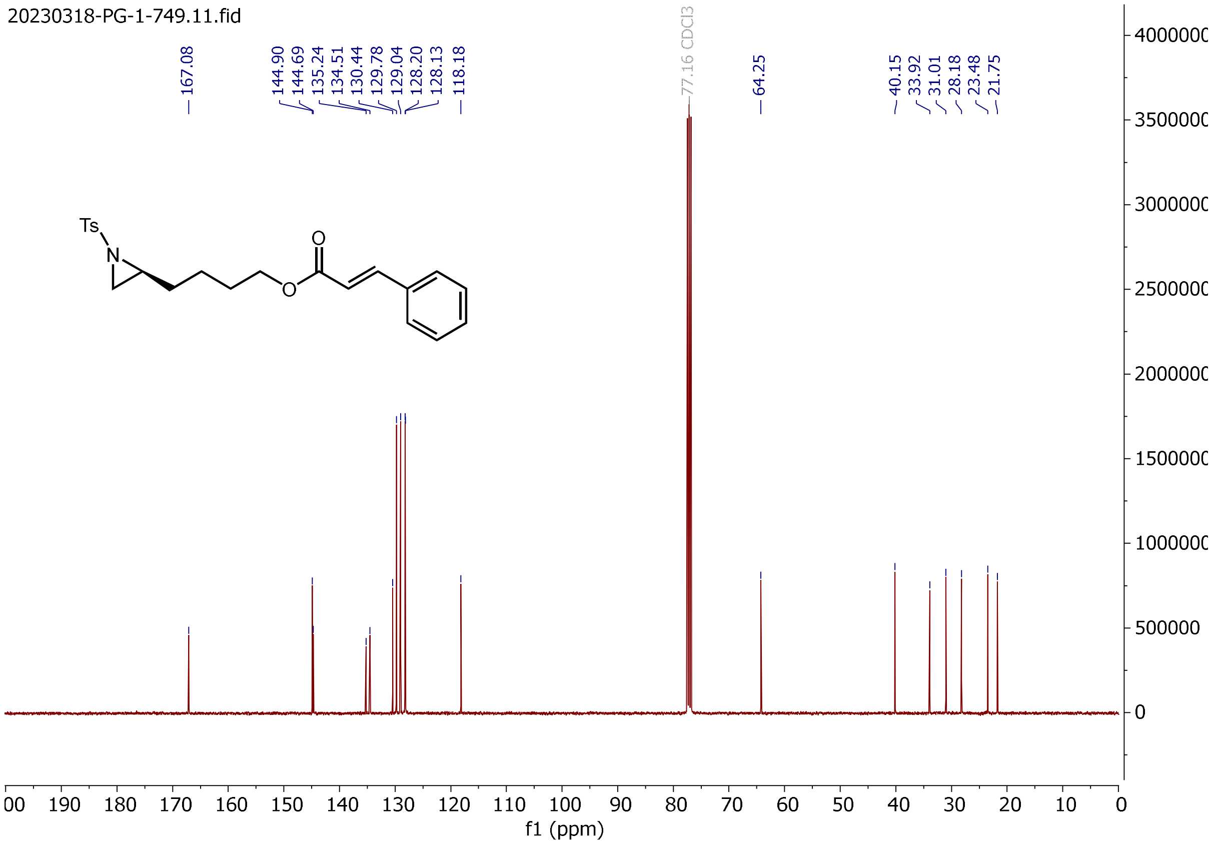

(±)-4-(1-tosylaziridin-2-yl)butyl cinnamate (±-**43**):

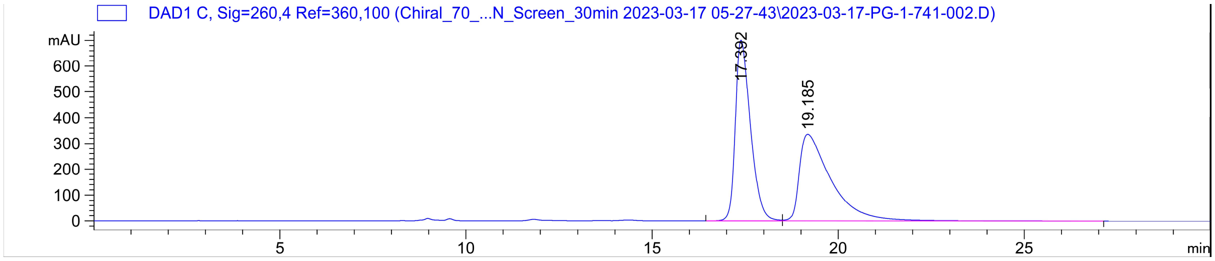

Signal 3: DAD1 C, Sig=260,4 Ref=360,100

Peak #	RetTime [min]	Type	Width [min]	Area [mAU*s]	Height [mAU]	Area %
1	17.392	BV	0.4308	1.98685e4	700.71686	49.9562
2	19.185	VB	0.8633	1.99033e4	335.88550	50.0438

Totals : 3.97718e4 1036.60236

(S)-4-(1-tosylaziridin-2-yl)butyl cinnamate (**43**):

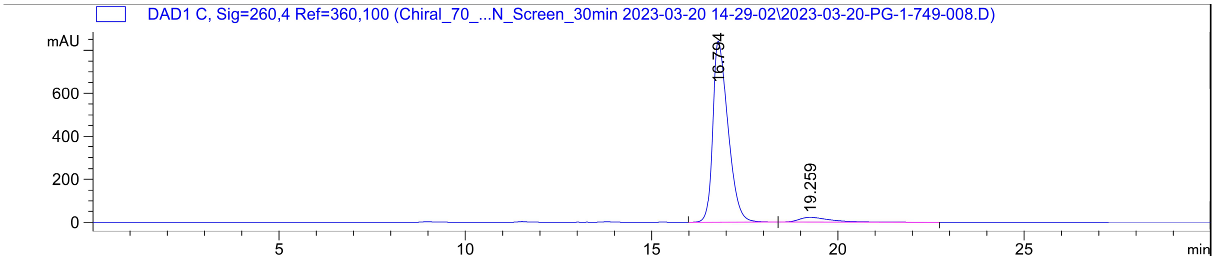

Signal 3: DAD1 C, Sig=260,4 Ref=360,100

Peak #	RetTime [min]	Type	Width [min]	Area [mAU*s]	Height [mAU]	Area %
1	16.794	BB	0.4261	2.36567e4	841.26526	94.9186
2	19.259	BB	0.8441	1266.44214	21.78959	5.0814

Totals : 2.49231e4 863.05485
